# Genome-wide analysis of core promoter elements from conserved human and mouse orthologous pairs

**DOI:** 10.1186/1471-2105-7-114

**Published:** 2006-03-07

**Authors:** Victor X Jin, Gregory AC Singer, Francisco J Agosto-Pérez, Sandya Liyanarachchi, Ramana V Davuluri

**Affiliations:** 1 Human Cancer Genetics Program, Comprehensive Cancer Center, Department of Molecular Virology, Immunology, and Medical Genetics, The Ohio State University, Columbus, OH 43210, USA

## Abstract

**Background:**

The canonical core promoter elements consist of the TATA box, initiator (Inr), downstream core promoter element (DPE), TFIIB recognition element (BRE) and the newly-discovered motif 10 element (MTE). The motifs for these core promoter elements are highly degenerate, which tends to lead to a high false discovery rate when attempting to detect them in promoter sequences.

**Results:**

In this study, we have performed the first analysis of these core promoter elements in orthologous mouse and human promoters with experimentally-supported transcription start sites. We have identified these various elements using a combination of positional weight matrices (PWMs) and the degree of conservation of orthologous mouse and human sequences – a procedure that significantly reduces the false positive rate of motif discovery. Our analysis of 9,010 orthologous mouse-human promoter pairs revealed two combinations of three-way synergistic effects, TATA-Inr-MTE and BRE-Inr-MTE. The former has previously been putatively identified in human, but the latter represents a novel synergistic relationship.

**Conclusion:**

Our results demonstrate that DNA sequence conservation can greatly improve the identification of functional core promoter elements in the human genome. The data also underscores the importance of synergistic occurrence of two or more core promoter elements. Furthermore, the sequence data and results presented here can help build better computational models for predicting the transcription start sites in the promoter regions, which remains one of the most challenging problems.

## Background

**Figure 1:**
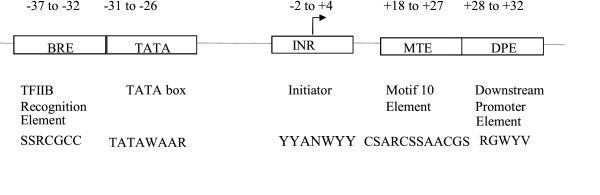
The schematic diagram of the core promoter elements.

The core promoter region is a key component in the regulation of gene transcription by RNA polymerase II, and includes DNA sequence elements that extend ~35 bp upstream and downstream of the transcription start site (TSS) [1]. Most core promoter elements are thought to interact directly with components of the basal transcription machinery, which is comprised of the multisubunit RNA polymerase II and several auxiliary factors [2-4]. For example, recent published crystal structures of apoIBD and IBD-Inr complexes present direct evidence for Inr element-mediated transcription initiation via direct binding [5]. Although no known sequence motifs are shared by all core promoters, four core promoter elements have been well-characterized in the *Drosophila *genome [6,7]: the TATA box, Initiator (Inr), downstream promoter element (DPE), and TFIIB recognition element (BRE). Core promoters possess considerable structural and functional diversity [8], and play important roles in the combinatorial regulation of gene transcription [9]. The TATA box binds to the TATA box-binding protein (TBP) subunit of the TFIID complex [10], and has a consensus sequence of TATAWAAR (degenerate nucleotides are designated according to the IUPAC code [11]. The Inr [12] has a consensus of YYANWYY in humans and TCAKTY in *Drosophila*. The DPE, mostly found in TATA-less promoters in *Drosophila *[13,14], has a consensus sequence of RGWYV. The BRE element, which is the only well-characterized element recognized by a TFIIB factor, has a consensus of SSRCGCC [15]. In addition to these four well-known elements, the motif 10 element (MTE), first discovered by computational analysis of *Drosphila *promoters [16], is a novel core promoter element that promotes transcription by RNA polymerase II when it is located precisely at positions +18 to +27 relative to the TSS [17]. The MTE has a consensus of CSARCSSAACGS, and appears to be present in both *Drosophila *and human promoter sequences [17]. These various elements and their relative positions in the promoter region are depicted in Figure 1.

Core promoter elements are highly variable, making sophisticated techniques necessary for their detection. Early work from Zhang employed both linear discriminant analysis and quadratic discriminant analysis to identify human core promoter regions [18]. Several other methods for the prediction of human promoters have been reported as well [19-21], but the bulk of research in this field of core promoter prediction has been focused on the fruitfly, *Drosophila *[22,23]. While *ab initio *techniques are improving, computational predictions can still be greatly enhanced by combining them with experimental data. Ohler's work, for example, has recently shown that *de novo *promoter predictions are greatly improved by starting with accurate transcription start sites that have been determined from a cap-trapped cDNA library, whose 5' ends were complete [16].

A comparative genomics strategy has been widely used by both experimental and computational biologists to aid regulatory element identification by examining orthologous sequences from multiple species. The studies from both Suzuki *et al*. [24] and Iwama and Gojobori [25] have identified blocks of highly conserved regions in orthologous human and mouse promoter sequences. Wasserman *et al*. [26] and Liu *et al*. [27] also have performed systematic analyses of sequence conservation in known human *cis*-regulatory elements and were able to use sequence conservation as a criterion to identify putative binding sites. Further, the diversity of the core promoter sequences suggests synergistic co-occurrence of two or more elements in order to have a level of specificity of transcription initiation in individual promoters. For example, based on current experimental evidence the MTE functions in TATA, Initiator, and DPE contexts [17]. However, the degree of conservation of individual core promoter elements in mammalian genomes and their synergistic occurrence within the core promoter regions are largely unknown. In this study, we describe a new approach that incorporates the positional weight matrices (PWMs) of the five core promoter elements with mouse-human sequence conservation information. We applied our approach to a set of 9,010 experimentally-supported human promoter sequences paired with their orthologous counterparts in mouse. In concert with the recent work of Gershenzon and Ioshikhes [28], we find strong evidence for synergism among the various core promoter elements, including a novel three-way pairing of the BRE-Inr-MTE elements.

## Results

### Core promoters regions are conserved

**Figure 2:**
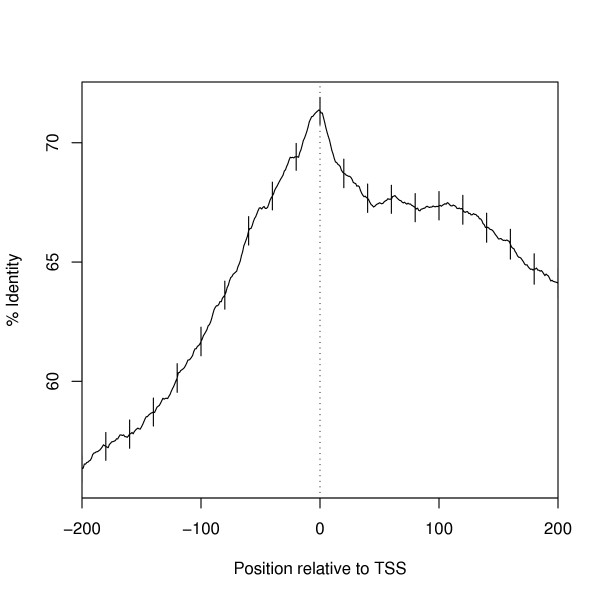
Average sequence similarity between orthologous human and mouse promoter regions is very high at the transcription start site (vertical dotted line), and drops sharply both up- and downstream of this point. Points indicate the mean percent identity in sliding 20-base windows along our dataset of 9,010 orthologous mouse-human promoter pairs. 95% confidence interval bars are plotted at every 10 bases.

The coding regions within orthologous mouse and human gene pairs tend to show high levels of sequence similarity [29], and therefore non-coding regulatory regions that lie upstream of the protein-encoding sequence should also be well-conserved between the two organisms. Indeed, a number of studies have shown that these regions do show a level of sequence similarity that is significantly higher than that of the nonfunctional regions in each genome [24,25]. Figure 2 shows that the findings of Suzuki *et al*. [24] are also true for our independently-derived dataset: there is a clear peak of sequence similarity at the transcription start site (TSS) of orthologous mouse and human sequences, which falls quickly both upstream and downstream of this point. This evidence shows that the core promoter region, which lies roughly -50 bp to +50 bp of the TSS, is preserved very well between mouse and human genes. Indeed, small "shelves" can be seen around positions -30 and -50 relative to the TSS, which we believe may correspond to the TATA and BRE elements present in many of these promoters. This level of sequence conservation indicates that comparative genomics may be a valuable tool in the identification of functional regulatory elements in orthologous core promoter regions.

### Annotation of the core promoter elements

The positions of each core promoter element are measured relative to the TSS, and have been experimentally determined [17,30]; see Figure 1. For the purpose of our analysis, we allowed these positions to vary +/-5 bp, because core promoter element placement often has some elasticity [30]. For each of the 9,010 human promoters in our dataset (see Additional File 1), we examined each target region relative to the TSS for sequences that closely matched the motif of a known promoter element. We attempted to distinguish false positives from true positives by using the scores generated by position weight matrices (see the Materials and Methods). The numbers of promoters having different core promoter elements are presented in Column 2 of Table 1.

Although all the 9,010 TSSs in our dataset are supported by full-length mRNA sequences, few have been studied in detail and it is possible that sequencing or annotation errors have skewed our results. For this reason, we downloaded the Eukaryotic Promoter Database [31] and selected promoters from within our own dataset whose TSS matched a TSS within the Eukaryotic Promoter Database (EPD). The result was a set of 624 sequences for which we can be highly confident of both active promoter activity and the exact position of the TSS. The proportion of each element found within this smaller dataset closely mirrors that of the larger dataset, with the exception of an enrichment of TATA boxes within the EPD set (the EPD is known to have a TATA bias; see [32]). The results from this EPD-derived dataset are listed in **Supplementary Table 2 **(see Additional file 2).

**Figure 3:**
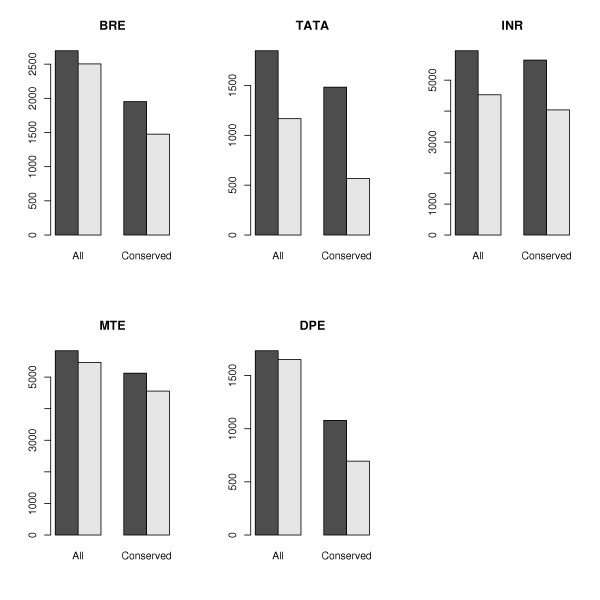
The number of core promoter elements in the real promoter sequences (dark bars) significantly exceeds the numbers found in the randomized sequences (light bars) in all cases. When we add the criterion that the element must be conserved in the mouse genome, we find that the gap between the number of elements found in the real data versus the random data widens, indicating an increase in the signal-to-noise ratio.

**Figure 4:**
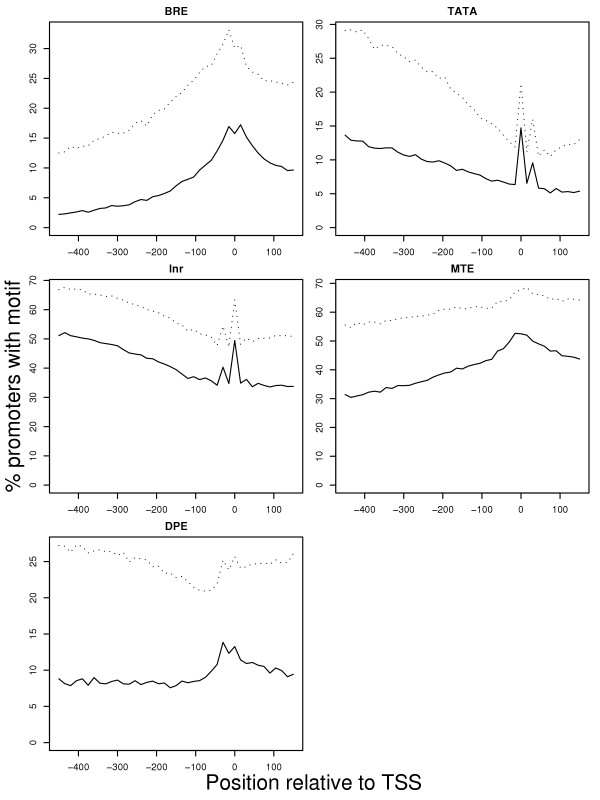
The number of each motif discovered in its expected position relative to the true TSS (position zero) represents a local maximum when compared to the sequence immediately upstream. The dotted lines show the results for a single-genome scan, while the solid lines show the results when only those motifs that are conserved in the orthologous mouse promoter are accepted.

To further evaluate the accuracy of our promoter element annotations, we bootstrapped the human promoter sequences by re-sampling the nucleotides (without replacement) and re-running our core promoter element annotation procedure (a similar technique was employed by Frith et al., [33]). These randomized promoters are obviously non-functional, but they have the same nucleotide frequencies as the original sequences and are therefore preferable to using inter-genic sequences (which tend to be much more AT-rich than mammalian promoters), or exonic sequences (which, since they are usually protein-encoding, have strongly biased nucleotide distributions). In all cases the number of elements identified within the real promoter sequences exceeded the number found in the randomized sequences, indicating a clear positive "signal" for the core promoter elements (Figure 3). Another means of displaying the motif signals compared to the background is via a sliding window analysis. We scanned for core promoter elements in the sequence immediately upstream of the TSS, and compared the abundance of these false motifs to that of the motifs identified in their correct positions relative to the TSS. For most of the motifs, we found that there is an excess at their correct positions relative to the sequence upstream of these regions (Figure 4, dotted lines). Interestingly, TATA and Inr show a marked dip in frequency upstream of the TSS, indicating motif avoidance in this region. Presumably, this is to prevent the transcriptional machinery from being confused by TATA- and Inr-like sites near the TSS.

Our results show a clear core promoter element signal within the promoter sequences, but it is important to note that our false discovery rate is very high. For example, we found 1,848 promoters with a TATA box in the real dataset, but 1,167 bootstrapped promoters also had a TATA box identified in the correct position (Table 1). Of course, we know *a priori *that the real dataset consists of functional promoters, so the situation is not as dire as it would appear at first. Nevertheless, we believed that the false discovery rate could be improved by employing a comparative genomics technique, reasoning that "real" core promoter elements would be conserved in the orthologous mouse promoter, while nonfunctional elements would not.

### Improving the signal-to-noise ratio using comparative genomics

Since we already know that there is a high level of sequence conservation surrounding the TSS in orthologous mouse and human genes (Figure 2), it is reasonable to assume that core promoter elements, which are important for transcription, will be conserved in the mouse genome. Conversely, we do not expect false positives (i.e., high scoring motifs in the DNA that are not functional promoter elements) to be conserved. For each human TSS, we found the orthologous mouse TSS and surrounding DNA from OMGProm [34], and scanned those sequences for core promoter elements. As shown in Table 1, adding the condition that each human core promoter element must also occur in the corresponding mouse promoter causes the number of predicted elements to go down, ostensibly because false positives are being eliminated. This trend is also depicted in Figure 4, where the gap between the dotted (single-genome scan) and solid (conservation criterion) lines is great in the region upstream of the TSS, but is much narrower at the TSS. Once again, we also measured the robustness of our analyses by comparing these results to those from randomized promoter sequences. For this analysis, we performed a bootstrapping technique similar to the one described in the previous section, only this time we sampled columns in the mouse-human promoter sequence alignment instead of the individual nucleotides in each sequence, thus allowing the orthologous DNA positions in the alignment to remain orthologous; in other words, the overall sequence similarity for the region is identical, but the order of bases in the alignment is scrambled. We then scanned through these randomized sequences using the exact same procedure as we had for the real alignments. While the quantity of promoter elements identified in both the real and the randomized alignments decreases, this decrease is much greater in the randomized alignments, resulting in a significantly better signal-to-noise ratio for all of the promoter elements, though it should be noted that after correcting for multiple testing, neither the Inr nor MTE elements show significant improvements (Table 1).

Our results based on human-mouse sequence conservation suggest that roughly 22% of human promoters contain the BRE element, 17% contain the TATA box, 62% contain an Initiator element, 57% contain the MTE, and about 12% contain a DPE (Table 1). We note that these numbers are reported by *promoter*, and not by *gene*. Since 1,326 genes in our dataset have two or more promoters, the proportion of each promoter element per gene is somewhat higher. For example, 22% of promoters have a TATA box, but 25% of all genes have at least one promoter with a TATA box. Similarly, 62% of promoters have an Inr, but 67% of all genes have an Inr in one or more promoters. These results are similar to those in a recent study by Gershenzon *et al*. [28]. The study from Suzuki *et al *[35] estimated that roughly a third of human promoters contained a TATA box, but our estimate is much lower at only 17%. This lower estimate suggests that the TATA box is much rarer, than was historically believed [28], but is consistent with many studies on the subject that confirm these lower estimates [28,36-38]. Interestingly, we found only 282 promoters (~3.1%) with no identifiable known core promoter elements, which suggests that although additional undiscovered core promoter elements may exist in the human genome, the current known set is sufficient to explain RNA polymerase II transcription for the overwhelming majority of genes. We identified only nine promoters that contained all five core promoter motifs, while the most common condition was for two core promoter motifs to be present. We have made the annotated 9,010 human promoter sequences available in **Supplemental Table 1 **(see Additional file 1).

A potential source of bias in these results are the dinucleotide frequencies of our negative dataset. While the randomization procedure keeps the nucleotide frequencies identical to the original sequences, we confirmed that the frequency of dinucleotides in these sequences departs significantly from the original promoters. This difference could result in unrealistic conditions in which to test the accuracy of analyses. For this reason, we performed a new randomization procedure, this time splitting up the original sequence into overlapping dinucleotides and carefully rearranging them into a new sequence. These new sequences are identical to the originals in terms of both nucleotide and dinucleotide frequencies, but the order of dinucleotides has been randomized. Interestingly, we found that our analysis of these new sequences was nearly identical to that of the original randomized set of sequences, leading to the same conclusions about core promoter element frequency and distribution (data not shown).

### Synergism between core promoter elements

**Figure 5:**
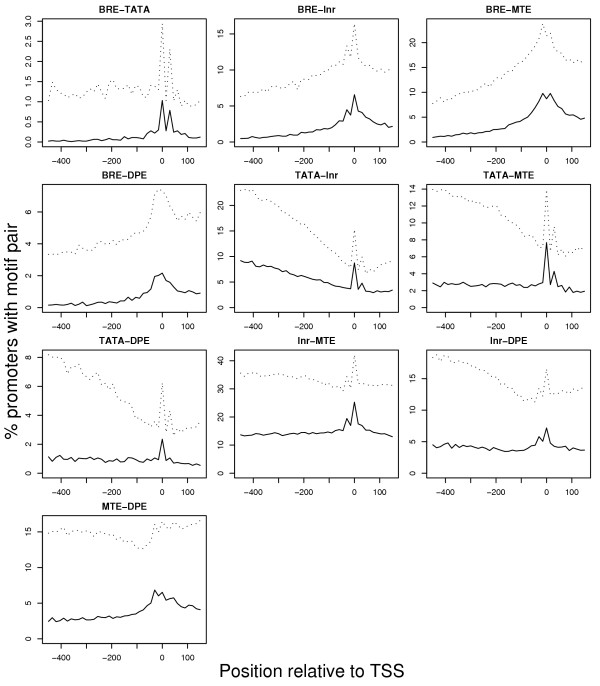
Pairs of motifs are much more likely to occur at the TSS than within sequences up- or downstream of the TSS. The dotted and solid lines are as described in the legend for **Figure 4**.

As we reported above, the most common condition in any given promoter is for two core promoter elements to be present, and it is extremely rare for all five to be present. This pairing is quite striking, as shown in Figure 5, where a sharp peak in pair frequency is observed at the TSS position, but not at sites up- or downstream of the TSS. This peak is very pronounced for all possible motif pairs – especially when the criterion that the pair must also be present in the orthologous mouse promoter is applied to reduce the false positive rate. A number of these combinations are of particular interest. For example, the high proportion of promoters with TATA-MTE, MTE-DPE, and expecially Inr-MTE confirms the findings of previous studies of the MTE [17]. Similarly, the TATA and Inr elements are known to interact [39], and this is also demonstrated by our results, which show that 10% of promoters have both of these elements. Also significant peaks exist for both the BRE-TATA and TATA-DPE combinations, a very small proportion of promoters have these particular combinations. The DPE is thought to occur mostly in TATA-less promoters [14], and our results confirm that this is generally true. The avoidance of BRE-TATA pairing is also an interesting trend, and is consistent with the findings of [28], who found that BRE tended to be found in TATA-less promoters. We note that peaks roughly 20–30 bp either upstream or downstream exist for some pairs. We attribute this to two factors. For one, it is possible that these auxiliary peaks represent true TSSs from multiple-TSS genes. However, we believe that a greater contribution to this artifact comes from the similarity of the TATA and Inr PWMs. A TATA box may be mistaken for an initiator element or *vice versa *(Table 1), causing an additional spurious peak ~30 bp upstream of the true TSS whenever a TATA box exists in a promoter, or at ~30 bp downstream of the TSS when an Inr is present.

The simple motif counting exercise above reveals interesting trends, but in order to work in a synergistic manner, two core promoter elements must not only be present but must also be within a narrowly-defined distance from each other. For example, we have defined position of the first T of the TATA element as occurring between -41 and -31 of the TSS, and the first A in the Inr element as occurring between -5 and +5 of the TSS. However, in order to work together, these two elements must be 26–30 bases apart [40]. Because of the flexibility in our scanning process, it is quite possible for a particular promoter to have both elements, but for the two elements to be spaced in such a way that they could not possibly work synergistically: a TATA at position -41 cannot work together with an Inr identified at position +5, since they have more than 30 bases between them. We might ask the question: Given that a high-scoring TATA and a high-scoring Inr element are present in a particular promoter, what is the probability that they are spaced in such a way that they can work synergistically? To address this question, we compare this conditional probability as measured in the real data sets to those measured in the bootstrapped data sets. Table 2 shows that the following pairs are more likely to occur at a synergistic distance than within the randomized dataset: TATA-MTE, BRE-Inr, Inr-MTE, and BRE-MTE. Again, the robustness of these results may be improved by comparison to the mouse genome. We can ask the following: Given that two elements are present in a human promoter, and are also present in the orthologous promoter in mouse, what is the probability that the elements are at a synergistic distance in both genomes? In this case, the results between the real promoters and randomized promoters are strikingly different (Table 2), and show that there is evidence for a synergistic effect between virtually all possible pairs of promoters, save the BRE-DPE combination. Surprisingly, when we correct the p-values for multiple testing, we find that the TATA-Inr combination becomes insignificant – which is striking because these two elements are known to work synergistically [39]. It is probable that this strange result is due to the lack of specificity in our ability to detect Inr elements (see Table 1). This problem is alleviated somewhat by looking for three-way synergistic effects, since the addition of a third component further reduces the false positive rate of detection. Out of the ten possible three-way interactions, two produced highly significant results: properly aligned BRE-Inr-MTE combinations are nearly four times as likely in the real dataset than in the randomized data (p = 4.1 × 10^-6^), and the TATA-Inr-MTE combination is about 50% more likely in the real data versus the random data, which is also significant (p = 0.0042). How can we interpret the cases where two promoter elements are present but are not spaced appropriately? We have identified two possible explanations: First, false positives are always possible and not all of the promoter elements may be real. Second, the elements may be real, but the promoter has a "loose" TSS [28,41], meaning the core promoter elements work independently and drive slightly different TSSs.

## Discussion

TATA and Inr elements have position weight matrices (PWMs) represented in the TRANSFAC database ([42]; TATA_01 and TATA_C for TATA box, CAP for Inr), but to the best of our knowledge, no PWMs previously existed for the BRE, DPE, and MTE elements in mammalian genomes. The PWMs we have constructed are not based on experimentally-verified data, but we feel that it is better to use our less-than-perfect matrices than simple motif consensus searches when attempting to identify core promoter elements because of the additional information that they contain. We should also point out that our PWMs for the TATA and Inr elements are slightly different from those in the TRANSFAC database [42] in that when building ours, we only considered sequences from mammalian species. Moreover, our TATA matrix was built using the latest annotated sequences from the GenBank database [43]. In order to directly compare our Inr and TATA matrices to those in TRANSFAC, we used a simple Nelder-Mead simplex algorithm to optimize the core and matrix scores for each PWM such that the false positive rate (as judged on our negative control set of "shuffled" promoter sequences) was fixed at 5% and the number of motifs found in the true promoter sequences was maximized. We found that TRANFAC's MATCH program [44] reports 11.2% of the promoters have an Inr and only 11.0% have a TATA box. Our own matrices performed similarly, with 10.6% and 12.5%, respectively. When we applied the conservation criterion – that the motif also had to be present in the orthologous mouse sequence – the results improved for both sets of PWMs, but more so for ours: MATCH finds 11.2% and 12.3% for Inr and TATA, respectively, while we find 13.2% and 14.2% using our matrices. Thus, we conclude that our matrices perform very slightly but significantly (p < 0.0002) better than those from TRANSFAC when conservation in the mouse genome is taken into consideration. Repeating the analysis at various false positive rate cut-offs produced identical trends.

Using our PWMs, we have analyzed a set of 9,010 pairs of orthologous human and mouse promoters. Our analysis is unique in that we have used human and mouse sequence conservation as a tool to distinguish true promoter elements from those that appear to be functional but in fact are not. We have presented data for human promoter sequences that have been annotated with the aid of mouse sequences, but the reverse procedure – analyzing mouse promoters using orthologous human sequences – is also possible and we provide these annotations as **Supplementary Table 3 **(see Additional file 3). This research also marks the first time that all five core promoter elements experimentally confirmed by previous benchwork studies [1,6,9,10,14] have been studied in mammalian genomes. Notably, the MTE element has not previously been analyzed on a wide scale outside of the *Drosophila *genome.

Our results are generally in line with those of similar recent studies [28,36], in that we estimate that ~17% of promoters contain a TATA box, which is far less than was historically believed to be the case in humans, although many recent studies suggest that previous estimates of ~30% are too high [28,37,38]. We found only a small fraction (~3%) of promoters with no identifiable core promoter elements, but it is quite probable that our methodology lacks the sensitivity to identify all of core promoter elements present in our dataset; in other words, these apparently element-less promoters may constitute false negatives. In order to investigate this, we examined the number of human promoters lacking visible core promoter elements whose mouse orthologues also lacked core promoter elements. Only 56, or less than 1% of the promoters in our dataset met this condition. Therefore, we believe that the current set of five core promoter elements is sufficient to explain RNA polymerase II-driven transcription. Despite this, we fully expect that novel core promoter elements remain to be discovered in mammalian genomes.

Experimental studies have shown that core promoter elements can work in concert when they are spaced in such a manner that both can bind to the transcriptional machinery simultaneously. Lim *et al*. [17] showed that in *Drosophila*, the MTE works in pair wise conjunction with TATA, Initiator or DPE, when it is positioned exactly at +18 to +27 relative to the A_+1 _of the Inr. This same principle could, in theory, apply to any two core promoter elements that are appropriately spaced relative to each other, and indeed this notion was recently confirmed [28]. Our independent results also support this model, with the caveat that the BRE-DPE combination is not found with significantly greater frequency in the real promoter sequences than in randomized sequences (Table 2). We have extended this analysis to demonstrate that two three-way combinations are also highly favorable in human promoters: BRE-Inr-MTE and TATA-Inr-MTE. It is interesting to note that these two combinations have anchor points on either side of the TSS, and within the TSS itself, so from a structural point of view these particular combinations ought to position the RNA polymerase II complex very efficiently. The modest synergism of TATA-Inr-MTE is supported by laboratory results published by Lim *et al*. [17], but the BRE-Inr-MTE combination is novel. Although BRE-Inr-DPE and TATA-Inr-DPE also have anchor points before, at, and after the TSS, we find that these are no more likely in the real data than in the random data.

## Conclusion

We have shown that DNA sequence conservation can greatly improve the identification of functional motifs in the human genome. The data also underscores the importance of synergistic occurrence of two or more core promoter elements. We believe that the sequence data and results presented here can help build better computational models for predicting the transcription start sites in the promoter regions, which remains a very difficult problem. Of course, by themselves, the methods in this paper are inadequate for *de novo *core promoter detection because of the very high false positive rate. However, traditional *de novo *promoter detection methods like FirstEF [19] and DragonGSF [21], rely upon DNA sequence characteristics such as di- or trinucleotide frequencies, and also suffer from a high false positive rate. We believe that in combination, however, these two methods – core promoter element prediction and *ab initio *promoter prediction – may help eliminate each other's false positives, improving the overall performance of a combined approach.

## Methods

### Promoter sequence retrieval

The transcription start site (TSS) is a central part of promoter annotation and is a key element to the determination of the core-promoter region. We have recently developed a promoter database of orthologous mammalian genes, called OMGProm [34], in which the TSS annotation is experimentally supported in at least one species. The methodology by which these data were gathered is described elsewhere [34], but in short, we collected full-length mRNA/5'UTRs from GenBank [43], and DBTSS [41], and experimentally determined promoters and first-exons from GenBank [43]. Many genes have more than one TSS annotated. To ensure that each TSS was truly distinct and had its own core promoter, we imposed a distance of 50 bp between neighboring TSSs, and took the most 5' TSS in cases where this distance was not met. We found that, for genes with more than one TSS, 68% have neighboring TSSs in excess of 100 bp apart, and 17% are more than 1 kb apart. In total, there are 10,922 pairs of experimentally supported human promoter sequences paired with mouse counterparts. Of these 10,922 pairs of orthologous promoters, 9,010 are conserved well enough that their TSSs are within a base pair of each other after ClustalW [45] alignment. The requirement of high sequence similarity biases our dataset towards those genes that have a high degree of evolutionary constraint, but it is a necessary condition of the methods we employ to identify core promoter elements. Because of this, it is unclear whether our results from this highly conserved dataset can be extrapolated to promoters that show a significant evolutionary departure between the mouse and human orthologs.

From the OMGProm database, we further selected 4,100 pairs of the mouse and human orthologous promoter sequences in which the TSS annotation for *both *species are experimentally supported as well as aligned in the ClustalW aligned formats. From this set, a 5' flanking region of 200 bp, from -100 to +100 bp relative to the TSS of the human and mouse promoter sequences were retrieved for building positional weight matrices for core elements. We also used the whole dataset of 9,010 pairs in the OMGProm database as a genome-wide test dataset to identify conserved core promoter elements.

### Defining positional weight matrices for the core promoter elements

With the exception of the TATA box, few core promoter elements have been experimentally characterized, making it impossible to construct their positional weight matrices (PWMs). For this reason, to build our PWMs we used a two-pass approach, where we first scanned through a dataset of 4,100 real human and mouse orthologous pairs of promoter sequences using consensus motifs defined in various papers [10,12-16], and then created PWMs from those regions matching the consensus plus 1 flanking bases on each side, which we then used for the second scan through the entire dataset. These flanking bases were added with the hope that some additional information might exist in these sites, but our results showed that they are, in fact, uninformative and we therefore eliminated these flanking sites from the PWMs – BRE PWM (Table 3), TATA PWM (Table 4), Inr PWM (Table 5), MTE PWM (Table 6), DPE PWM (Table 7). We only accepted those putative elements where the sequence was 80% identical between mouse and human, and the same element was identified in both the mouse and human in the same position relative to the TSS.

The position weight matrix commonly used for identification of the TATA box was compiled 15 years ago [46], from a small number of experimentally-verified sequences from a diverse set of organisms. Since then, large-scale sequencing efforts have vastly increased the potential pool of TATA elements from which to build PWMs, but surprisingly, the number of experimentally-verified elements has not increased substantially. Nonetheless, we decided that some useful information may be contained in the hand-annotated TATA boxes within GenBank entries. We first retrieved a total of 965 mammalian promoter sequences from GenBank that had annotated TATA elements ~-30 bp from the TSS. After extending 25 bases in each side of TATA box with a total of 50 bp for each promoter sequence, we ran the MEME program [47] on this dataset. Interestingly, the MEME result shows a 21-base consensus motif from 897 promoter sequences with a very low E-value (5.9e-950). The position-specific probability matrix from the MEME output was then used as our matrix for the TATA element.

When constructing PWMs, it is important to include enough sequence information to have high sensitivity, but not so much that the core motifs of the elements are lost in the noise. For each PWM, we defined two scores: core score for the central five bases of the core consensus, and the PWM score for the whole matrix. The core and PWM scores, ranging from 0 to 1, reflect the closeness of predicted sites to consensus sequences. PWMs (Tables 3, 4, 5, 6, 7) are more sensitive than pure consensus sequences and we used them to scan the test dataset of promoter sequences to identify the core promoter elements. In order to distinguish true core promoter motifs from false positives, it is necessary to choose score cut-offs. Ideally, this would be accomplished by measuring the scores generated by a PWM on a set of true positives, and compare these to scores generated on a set of non-promoter sequences, to measure the false positive rate. Cut-offs could then be chosen in such a way that the true positive rate is maximized while trying to limit the false positive rate. Unfortunately, with the exception of the TATA box, not enough true positives are known for each of the binding sites for us to test the sensitivity of the PWM.

The cutoffs for the core and matrix scores for each core promoter element were determined by iteration over a wide range of values and optimizing each element's "signal" in its correct position relative to the TSS relative to the "noise" (false positives) in the surrounding sequence within the promoter. We subsequently analyzed the sensitivity and specificity of the PWMs. First, we checked the scores generated by the original training sequences used to build the matrices, and all of them were detected at these cutoffs. Because, by definition, these training sequences all matched the consesus sequence for each motif, this is likely to be an overestimate of the sensitivity of our methods. Next, we applied the PWMs to randomized promoter sequences (see the Results, second subsection), and analysed the performance over a range of matrix- and core-score cutoff values. In general, the PWMs have a low specificity, however, we found that the scores we employ in our analyses are an optimal balance of being able to detect all of the true positive set, while minimizing the hits in the negative set.

## Abbreviations

Transcription Start Site (TSS), Initiator (Inr), Downstream core Promoter Element (DPE), TFIIB recognition element (BRE), Motif Ten Element (MTE), TATA Box-binding Protein (TBP), Positional Weight Matrix (PWM), Eukaryotic Promoter Database (EPD), DataBase of Transcriptional Start Sites (DBTSS), Orthologous Mammalian Gene Promoter database (OMGProm).

## Authors' contributions

VJ and GS designed the methods and performed computational analysis. FA generated the datasets. GS performed statistical analysis assisted by SL. RD formulated and coordinated the research. All authors read and approved the final manuscript.

## Supplementary Material

Additional File 1 Supplementary Table 1: Composition of orthologous human-mouse core promoters; Alignment of Human and Mouse sequences with highlighted conservation of core promoter elements, in which the TSS of human promoter is experimentally supported. Click here for file

Additional File 2 Supplementary Table 2; Enumeration of core promoter elements in EPD with and without considering conservation in the mouse genome. Click here for file

Additional File 3 Supplementary Table 3: Composition of orthologous mouse-human core promoters; Alignment of Mouse and Human sequences with highlighted conservation of core promoter elements, in which the TSS of mouse promoter is experimentally supported. Click here for file

## Figures and Tables

**Table 1:** Enumeration of core promoter elements in the human genome, with and without considering conservation in the mouse genome

Motif (core, PWM score cutoffs)	Number of promoter elements found in 9,010 promoter sequences		Number of promoter elements that are conserved in the orthologous mouse promoters		Significance of increase in signal-to-noise ratio (one-tailed)^a^
		
	Real sequences	Randomized sequences	Real sequences	Randomized sequences	
BRE (N/A^b^, 0.81)	2696 (29.9%)	2503 (27.8%)	1952 (21.7%)	1476 (16.4%)	1.90 × 10^-6^
TATA (0.73, 0.58)	1848 (20.5%)	1167 (13.0%)	1483 (16.5%)	567 (6.3%)	1.70 × 10^-16^
INR (0.72, 0.62)	5949 (66.0%)	4527 (50.2%)	5648 (62.7%)	4040 (44.8%)	0.02
MTE (0.79, 0.53)	5833 (64.7%)	5464 (60.6%)	5123 (56.9%)	4555 (50.6%)	0.03
DPE (0.92, 0.92)	1733 (19.2%)	1650 (18.3%)	1078 (12.0%)	695 (7.7%)	2.90 × 10^-11^

^a^One-tailed Fisher's exact test of the real:randomized ratio in columns 4 and 5 versus columns 2 and 3. The p-value in the BRE row, for example, indicates that 1952/1476 is significantly greater than 2696/2503.

^b^The "core score" was found to be uninformative for the BRE element, so only the PWM score was used in this case.

**Table 2:** Conditional probabilities that two core promoter elements are spaced in a synergistically-favorable manner, given that both elements are present

Core promoter element pairs	p(synergy | both elements present)		One-tailed significance of synergy	p(synergy | both elements present and conserved in mouse counterparts)		One-tailed significance of synergy
				
	Real sequences	Randomized sequences		Real sequences	Randomized sequences	
TATA-MTE	0.75	0.57	2.3 × 10^-17^	0.72	0.45	2.3 × 10^-17^
BRE-Inr	0.54	0.46	4.0 × 10^-5^	0.45	0.31	7.2 × 10^-8^
TATA-Inr	0.68	0.69	0.67	0.67	0.60	0.01
BRE-DPE	0.43	0.42	0.46	0.32	0.24	0.10
Inr-DPE	0.48	0.47	0.20	0.42	0.33	0.00
MTE-DPE	0.45	0.49	0.98	0.33	0.24	0.00
Inr-MTE	0.53	0.46	1.9 × 10^-8^	0.44	0.34	3.9 × 10^-17^
BRE-TATA	0.45	0.38	0.08	0.46	0.08	0.00
BRE-MTE	0.31	0.27	0.00	0.2	0.12	5.4 × 10^-7^
TATA-DPE	0.5	0.45	0.11	0.45	0.22	0.00

**Table 3:** BRE PWM with defined core elements in bold

	1	2	3	4	5	6	7
A	0	0	26	0	0	0	0
C	51	50	0	74	0	74	74
G	23	24	48	0	74	0	0
T	0	0	0	0	0	0	0
Con	**S**	**S**	**R**	**C**	**G**	**C**	**C**

**Table 4:** TATA PWM with defined core elements in bold

	1	2	3	4	5	6	7	8	9	10	11	12	13	14	15	16	17	18	19	20	21
A	224	228	186	146	213	67	756	0	865	670	770	444	282	131	182	164	185	184	141	179	165
C	226	203	239	229	310	84	0	5	0	0	0	15	99	274	272	273	273	264	286	283	304
G	301	291	333	302	225	71	0	8	2	0	119	224	437	394	301	337	275	303	328	276	275
T	146	175	139	220	149	675	141	884	30	201	8	214	79	98	142	123	164	146	142	159	153
Con	N	N	N	N	N	**T**	**A**	**T**	**A**	**A**	**A**	**D**	**W**	N	N	N	N	N	N	N	N

**Table 5:** Inr PWM with defined core elements in bold

	1	2	3	4	5	6	7
A	0	0	506	81	143	0	0
C	248	388	0	223	0	193	320
G	0	0	0	102	0	0	0
T	258	118	0	100	363	313	186
Con	**Y**	**Y**	**A**	**N**	**W**	**Y**	**Y**

**Table 6:** MTE PWM with defined core elements in bold

	1	2	3	4	5	6	7	8	9	10	11	12
A	2	14	51	5	1	1	6	25	7	3	1	10
C	20	24	2	3	55	24	26	0	5	50	3	20
G	35	18	5	43	0	31	26	33	39	2	52	27
T	1	2	0	7	2	2	0	0	7	3	2	1
Con	**S**	**V**	**A**	**G**	**C**	**S**	**S**	**R**	**G**	**C**	**G**	**S**

**Table 7:** DPE PWM with defined core elements in bold

	1	2	3	4	5
A	514	0	585	0	214
C	0	0	0	549	303
G	481	995	0	0	478
T	0	0	410	446	0
Con	**R**	**G**	**W**	**Y**	**V**

## References

[B1] Butler JE, Kadonaga JT (2002). The RNA polymerase II core promoter: a key component in the regulation of gene expression. Genes Dev.

[B2] Hochheimer A, Tjian R (2003). Diversified transcription initiation complexes expand promoter selectivity and tissue-specific gene expression. Genes Dev.

[B3] Woychik NA, Hampsey M (2002). The RNA polymerase II machinery: structure illuminates function. Cell.

[B4] Hampsey M (1998). Molecular genetics of the RNA polymerase II general transcriptional machinery. Microbiol Mol Biol Rev.

[B5] Schumacher MA, Lau AO, Johnson PJ (2003). Structural basis of core promoter recognition in a primitive eukaryote. Cell.

[B6] Burke TW, Kadonaga JT (1997). The downstream core promoter element, DPE, is conserved from Drosophila to humans and is recognized by TAFII60 of Drosophila. Genes Dev.

[B7] Corden J, Wasylyk B, Buchwalder A, Sassone-Corsi P, Kedinger C, Chambon P (1980
). Promoter sequences of eukaryotic protein-coding genes. Science.

[B8] Levine M, Tjian R (2003). Transcription regulation and animal diversity.. Nature.

[B9] Smale ST (2001). Core promoters: active contributors to combinatorial gene regulation. Genes Dev.

[B10] Goldberg ML (1979). Sequence analysis of Drosophila histone genes..

[B11] IUPAC http://www.chem.qmul.ac.uk/iubmb/misc/naseq.html.

[B12] Smale ST, Baltimore D (1989). The 'initiator' as a transcription control element. Cell.

[B13] Kadonaga JT (2002). The DPE, a core promoter element for transcription by RNA polymerase II.. Exp Mol Med.

[B14] Burke TW, Kadonaga JT (1996). Drosophila TFIID binds to a conserved downstream basal promoter element that is present in many TATA-box-deficient promoters.. Genes & Dev.

[B15] Lagrange T, Kapanidis AN, Tang H, Reinberg D, Ebright RH (1998). New core promoter element in RNA polymerase II-dependent transcription: sequence-specific DNA binding by transcription factor IIB. Genes Dev.

[B16] Ohler U, Liao GC, Niemann H, Rubin GM (2002). Computational analysis of core promoters in the Drosophila genome. Genome Biol.

[B17] Lim CY, Santoso B, Boulay T, Dong E, Ohler U, Kadonaga JT (2004). The MTE, a new core promoter element for transcription by RNA polymerase II.. Genes Dev.

[B18] Zhang MQ (1998). Identification of human gene core promoters in silico. Genome Res.

[B19] Davuluri RV, Grosse I, Zhang MQ (2001). Computational identification of promoters and first exons in the human genome.. Nat Genet.

[B20] Hannenhalli S, Levy S (2001). Promoter prediction in the human genome. Bioinformatics.

[B21] Bajic VB, Tan SL, Suzuki Y, Sugano S (2004). Promoter prediction analysis on the whole human genome. Nat Biotechnol.

[B22] Reese MG (2001). Application of a time-delay neural network to promoter annotation in the Drosophila melanogaster genome. Comput Chem.

[B23] Levitsky G, Katokhin AV (2001). Computational analysis and recognition of Drosophila melanogaster gene promoters. Mol Biol.

[B24] Suzuki Y, Yamashita R, Shirota M, Sakakibara Y, Chiba J, Mizushima-Sugano J, Nakai K, Sugano S (2004). Sequence comparison of human and mouse genes reveals a homologous block structure in the promoter regions. Genome Res.

[B25] Iwama H, Gojobori T (2004). Highly conserved upstream sequences for transcription factor genes and implications for the regulatory network. Proc Natl Acad Sci U S A.

[B26] Wasserman WW, Palumbo M, Thompson W, Fickett JW, Lawrence CE (2000). Human-mouse genome comparisons to locate regulatory sites. Nat Genet.

[B27] Liu Y, Liu XS, Wei L, Altman RB, Batzoglou S (2004). Eukaryotic regulatory element conservation analysis and identification using comparative genomics. Genome Res.

[B28] Gershenzon NI, Ioshikhes I (2005). Synergy of human Pol II core promoter elements revealed by statistical sequence analysis. Bioinformatics.

[B29] Waterston RH, Lindblad-Toh K, Birneys E, Rogers J, Abril JF (2002). Initial sequencing and comparative analysis of the mouse genome. Nature.

[B30] Smale ST, Kadonaga JT (2003). The RNA polymerase II core promoter. Annu Rev Biochem.

[B31] Perier RC, Junier T, Bonnard C, Bucher P (1999). The Eukaryotic Promoter Database (EPD): recent developments. Nucleic Acids Res.

[B32] Zhang MQ (1998). A discrimination study of human core-promoters. Pac Symp Biocomput.

[B33] Frith MC, Li MC, Weng Z (2003). Cluster-Buster: Finding dense clusters of motifs in DNA sequences. Nucleic Acids Res.

[B34] Palaniswamy SK, Jin VX, Sun H, Davuluri RV (2004). OMGProm: a database of orthologous mammalian gene promoters. Bioinformatics.

[B35] Suzuki Y, Tsunoda T, Sese J, Taira H, Mizushima-Sugano J, Hata H, Ota T, Isogai T, Tanaka T, Nakamura Y, Suyama A, Sakaki Y, Morishita S, Okubo K, Sugano S (2001). Identification and characterization of the potential promoter regions of 1031 kinds of human genes. Genome Res.

[B36] Bajic VB, Choudhary V, Hock CK (2003). Content analysis of the core promoter region of human genes. In Silico Biol.

[B37] Basehoar AD, Zanton SJ, Pugh BF (2004). Identification and distinct regulation of yeast TATA box-containing genes. Cell.

[B38] Fukue Y, Sumida N, Nishikawa J, Ohyama T (2004). Core promoter elements of eukaryotic genes have a highly distinctive mechanical property. Nucleic Acids Res.

[B39] Emami KH, Jain A, Smale ST (1997). Mechanism of synergy between TATA and initiator: synergistic binding of TFIID following a putative TFIIA-induced isomerization. Genes Dev.

[B40] O'Shea-Greenfield A, Smale ST (1992). Roles of TATA and initiator elements in determining the start site location and direction of RNA polymerase II transcription. J Biol Chem.

[B41] Suzuki Y, Yamashita R, Sugano S, Nakai K (2004). DBTSS, DataBase of Transcriptional Start Sites: progress report. Nucleic Acids Res.

[B42] Wingender E, Chen X, Fricke E, Geffers R, Hehl R, Liebich I, Krull M, Matys V, Michael H, Ohnhauser R (2001). The TRANSFAC system on gene expression regulation.. Nucleic Acids Res.

[B43] Benson DA, Karsch-Mizrachi I, Lipman DJ, Ostell J, Wheeler DL (2004). GenBank: update. Nucleic Acids Res.

[B44] Kel AE, Gossling E, Reuter I, Cheremushkin E, Kel-Margoulis OV, Wingender E (2003). MATCH: A tool for searching transcription factor binding sites in DNA sequences. Nucleic Acids Res.

[B45] Thompson JD, Higgins DG, Gibson TJ (1994). CLUSTAL W: improving the sensitivity of progressive multiple sequence alignment through sequence weighting, position-specific gap penalties and weight matrix choice. Nucleic Acids Res.

[B46] Bucher P (1990). Weight matrix descriptions of four eukaryotic RNA polymerase II promoter elements derived from 502 unrelated promoter sequences. J Mol Biol.

[B47] Bailey TL, Gribskov M (1997). Score distributions for simultaneous matching to multiple motifs.. J Comput Biol.

