# Supplementary material for: Genome-wide analysis of core promoter elements from conserved human and mouse orthologous pairs
Source: BMC Bioinformatics. 2006 Mar 7;7:114. doi: 10.1186/1471-2105-7-114 (PMC1475891; doi:10.1186/1471-2105-7-114)
Supplement: Additional File 1 — Supplementary Table 1: Composition of orthologous human-mouse core promoters; Alignment of Human and Mouse sequences with highlighted conservation of core promoter elements, in which the TSS of human promoter is experimentally supported. [file 1471-2105-7-114-S1.zip › Jin_CorePromoters_Human.html]

 
   
   Composition of orthologous human-mouse core promoters 
   Entries are in the following format:  Organism: symbol (GeneID, Accession)   
   Hover cursor over elements to see their scores  
  1 Human: ACTA1 (58, NM_001100) 
Mouse: Acta1 (11459, NM_009606) 
 
           |-50      |-40      |-30      |-20      |-10      |1        |11       |21       |31       |41       
Human     CGGAGGGAATGCCCGCGGGCTA  TATAAAA  CCTGAGCAGAGGGA  CAAGCGG C   CACCGCAGCGGACAGCGCCAAGT  GAAGCCTCGCTTC  CCCTCCGCGGCGA
                                 (TATA+)                 (INR-)                              (MTE+)                 
Mouse     TGGGGAGAGCTCCCGGGACTA  TATAAAA  ACCTGTGCAAGGGGACAGGCGG   T CACACGG  ACGTGAAGCCTCACTTCCTACCCTCGGCACCCAGGGCCAGAG
                                (TATA+)                         (INR+)                                            
    2 Human: ACTN2 (88, NM_001103) 
Mouse: Actn2 (11472, AW556942) 
 
           |-50      |-40      |-30      |-20      |-10      |1        |11       |21       |31       |41       
Human     ACGTGACGGGC  GGCGCGGC    TATTAAG  CCGCGCGGCAGCTGCTCGCAGCCG   G AGCTGGT  GCTTCGCCCGAGAC  CCAGCGCCCAGGC  GTGTCGCCCCGAGAG
                       (BRE-)   (TATA+)                           (INR-)                     (MTE+)                   
Mouse     NNNNNNNNNNN  NNNNNGGG  C  TATTAAT  CCGCGCGGCGCTGCCTGCA  GGCG T GCT  GGTACTTCGCCGGAG  ACTCCGCGCCCAG  GCGTGCCGCCCCAGGAAC
                       (BRE-)    (TATA+)                      (INR-)                      (MTE-)                      
    3 Human: ACTN2 (88, AJ295982) 
Mouse: Actn2 (11472, AW556942) 
 
           |-50      |-40      |-30      |-20      |-10      |1        |11       |21       |31       |41       
Human     TTCTCATGTAGAGTGGTC  ATTATGA  TGTTGAAGTAAAAGAGTTGGTT  ATA T TTTC  ACCTTTGGATGCTAGGTTACCCATGAATGCAAAAAGGCCACTGAG
                             (TATA+)                         (INR+)                                               
Mouse     CTCCTAGGGGGTTGCTAGCAC  TGTCCTA  TGTGAGCAGAGGCTTGG  TCACG T GT  CATCCTTTGGATGATGAGTTCCTGCG  GGTCTG  TGGCAAGCAAGAAGA
                                (TATA-)                    (INR+)                             (DPE-)                
    4 Human: ADAR (103, BX538232) 
Mouse: Adar (56417, AK089451) 
 
           |-50      |-40      |-30      |-20      |-10      |1        |11       |21       |31       |41       
Human     TCCTCCCACGCCCG  GCGCGCCA  ATGATGTCACCAATCTGCGACCAGA  CCA T TGAT  TCCCGACTGAAGGTAGAGAAGGCTACGTGGTGGGGGAGGGTGGGG
                          (BRE+)                             (INR+)                                               
Mouse     TCCCTCCCACGCCG  GCGCGCCA  GTGATGTCACCAATCTGCGCCCTAA  CCA T TGAT  TCCTGACTGAAGGTGGAAG  ACTACGCGTTGGG  ACTAGCCGGGAAG
                          (BRE+)                             (INR+)                          (MTE-)                 
    5 Human: ADORA1 (134, AK127752) 
Mouse: Adora1 (11539, AF133099) 
 
           |-50      |-40      |-30      |-20      |-10      |1        |11       |21       |31       |41       
Human     CCCGGTGTG  CGGAGCCC  GATTGTCACTCAGCTCCTGCGCCGGGGGCGACA G AGCCGCAGGCGCCCGAGT  CGAGTCCCAGCCA  GCTACCATCCCTCTGGAG
                     (BRE+)                                                           (MTE+)                      
Mouse     CCCGGTGTG  CGGAGCCC  GATTGTCACTCAGCTCCCGCGCCGGGGGCGACG A AGGCGCCGGCGCCAGAGTGG  AGTCTCGGCTGGC  TACCACCCCTTGGACA
                     (BRE+)                                                             (MTE-)                    
    6 Human: ADORA3 (140, NM_000677) 
Mouse: Adora3 (11542, M94152) 
 
           |-50      |-40      |-30      |-20      |-10      |1        |11       |21       |31       |41       
Human     TGAAACACCCCTGAAGAG  GGTTGCT  TATCTTGATGGAACTCAAAAAGCC  A A AAAGCT  GCAGGCAGAGGCGTTGAGGA  CATCTG  TTTGGGGAACTAAGAGC
                             (TATA-)                           (INR-)                       (DPE-)                  
Mouse     TTGAAACACTCTAAAGA  GGAGTGC  T  TATCTT  GATGGAGCTCGAAA  GTTCT C TG  GGGCCCAGGGGACAGCA  GAAGCCCTGACTC  TGTTTGCCTGGGGAAGT
                             (BRE-)  (TATA-)                 (INR+)                        (MTE+)                     
    7 Human: PARP1 (142, NM_001618) 
Mouse: Parp1 (11545, AF126717) 
 
           |-50      |-40      |-30      |-20      |-10      |1        |11       |21       |31       |41       
Human     CGCCGGCCCCGCCCCGTG  GACGCGGG  TTCCGTGGGCGTTCCCGCGGCCAG G CATCAGCAATCTATCAG  GGAACGGCGGTGG  CCGGTGCGGCGTGTTCGGT
                              (BRE-)                                                 (MTE+)                       
Mouse     TGAGGCCCCGCCCT  CCGCGCGC  GAGCTTGGGGGGGCGGTCCCGCCGCTAG G CA  TCAGTAAT  CTATCC  TGAGCGGAGGCGG  CTCTGCACGCTTGCGGGGGA
                          (BRE+)                                   (INR+)             (MTE+)                        
    8 Human: ADSS (159, NM_001126) 
Mouse: Adss (11566, AK028060) 
 
           |-50      |-40      |-30      |-20      |-10      |1        |11       |21       |31       |41       
Human     GGCTGGAGGGTGGC  GGCCCGGC  GGGGCGGGGGCGGGGCCGGCCTCTGGCT C   CTTCTTCC  TCTGCATGTGGCTGGCGGCCGCAGAGCAGTTCAGTTCGCTC
                          (BRE-)                                 (INR+)                                           
Mouse     TGGAGAGAGGGT  GGCGGCCC  TCGGGGCGGGGGCGGGGCCGGCCTCTGGCT C   CTTCTTCC  TCCGCATGTGGCTG  GTGGCGGCGCAGC  AGTTGGGTTCACGT
                        (BRE-)                                   (INR+)                     (MTE+)                  
    9 Human: AGT (183, M24685) 
Mouse: Agt (11606, AK005354) 
 
           |-50      |-40      |-30      |-20      |-10      |1        |11       |21       |31       |41       
Human     CCATCCCCACCCCTCAGC  TATAAAT  AGGGCATCGTGACCCGGCCGGGG  GA A GAAGC  TGCCGTTGTTCTGGGTACTACAGCAGAAGGTAAGCCGGGGGCCC
                             (TATA+)                          (INR-)                                              
Mouse     TCCATCTCCACCCCTTGAG  TATAAAT  AAGCCTGCTTGGCTCACCAGGGGA T A  GCTGTGCT  TGTCTA  GGTTGGCGCTGAA  GGTAAGCAGAGCACTCTTGTC
                              (TATA+)                             (INR-)             (MTE-)                         
    10 Human: AGT (183, NM_000029) 
Mouse: Agt (11606, AK005354) 
 
           |-50      |-40      |-30      |-20      |-10      |1        |11       |21       |31       |41       
Human     GCCTAAGCAAGACTCTCCCCTGCCCTCTGCCCTCTGCACCTCCGGCCT  GC A TGTCC  CTGTGGCCTCTTGGGGGTA  CATCTC  CCGGGGCTGGGTCAGAAGG
                                                            (INR+)                      (DPE-)                    
Mouse     GCAGCTGGTGACGTGAGGTTCAGCTCCTGCAAGTGAGCCCCCTTCCTG  GC A TGCCC  AGAGAGGCTTA  CGAGTGCATCACG  AGGGGGCTTTCATCCCAAGG
                                                            (INR+)                  (MTE+)                        
    11 Human: AK2 (204, CR596376) 
Mouse: Ak2 (11637, AK006763) 
 
           |-50      |-40      |-30      |-20      |-10      |1        |11       |21       |31       |41       
Human     CAGTGTGAGCGGCGAGTGGGACGTGCGTGGCGTGCGTGCGTTGACCTGGG A A  GCACTGGA  CCTGTGAGGCGTGC  GAACTG  GTGGCAGTGAGAGACTTCGG
                                                                (INR-)                 (DPE-)                     
Mouse     ATGCGGCGAGCGACCAGTGTGAGCCCGGAGTAGGATGTACGCTGGCCTG  G A AGCGCT  AGACCTGTGAG  GTGTGCGGCTGGC  TGTTGGAGTGAAGCTTTGG
                                                             (INR-)                  (MTE-)                       
    12 Human: AK3L1 (205, NM_203464) 
Mouse: Ak3l1 (11639, BY125224) 
 
           |-50      |-40      |-30      |-20      |-10      |1        |11       |21       |31       |41       
Human     GCGCGGCCGGCCGGGGCT  CCGCGACT  GGGCGGGCGGAGGCGGCGCCGCCC T GCAGGATCCGGGCTGGAAGCCTC  TCGGAGTGCTGCC  TCCAGCCAAGGTG
                              (BRE+)                                                       (MTE-)                 
Mouse     TAAGAAGCAGCGAGTCC  CGCGCGGC  CAGCCCGGCAGAGGCGGCGCCGCCC A GCGGGATCCGGGCTAAGTC  TGGCCGCGCTCCT  TTCCACCGAGGTGTGTG
                             (BRE-)                                                    (MTE-)                     
    13 Human: ALDH9A1 (223, NM_000696) 
Mouse: Aldh9a1 (56752, BB871736) 
 
           |-50      |-40      |-30      |-20      |-10      |1        |11       |21       |31       |41       
Human     CCCGCCCCTC  CCGCGGCC  CGTCAGCCTCTGCCGCGGAGCTGCGTCCG  CCA C TCAT  GTTTCTCCGAGCAGGCC  TGGCCGCGCTCTC  CCCGCTTCTTCGCAG
                      (BRE+)                                 (INR+)                        (MTE-)                   
Mouse     CCCCCTCCAGTAGTAATCTGCTGGGGCTTCCGGACGCTTTGCACGCGTA  G C AGATGA  TCCTCGGT  GCAGTCGGTTCTG  TGC  TGACCT  CGCTCCTCCGCAT
                                                             (INR+)               (MTE-)        (DPE-)              
    14 Human: ALPL (249, NM_000478) 
Mouse: Akp2 (11647, NM_007431) 
 
           |-50      |-40      |-30      |-20      |-10      |1        |11       |21       |31       |41       
Human     CGGGCCCCGCGGCCGCCTT  TATAAGG  CGGCGGGGGTGGTGGCCCGGGCCG C GTTGCGCTCCCGCCACTC  CGCGCCCGCTATC  CTGGCTCCGTGCTCCCAC
                              (TATA+)                                                 (MTE-)                      
Mouse     GGGTGCTCGG  CCAGGCCG  CCTT  CATAAGC  AGGCGGGGGAGGTGGCCGCC  A G AGTACG  CTCCCGCCACTGCGCT  CCTTAGGGCTGCC  GCTCGCGAGCCGGA
                      (BRE+)       (TATA+)                       (INR-)                       (MTE-)                  
    15 Human: AMPD1 (270, M37920) 
Mouse: Ampd1 (229665, AK086147) 
 
           |-50      |-40      |-30      |-20      |-10      |1        |11       |21       |31       |41       
Human     AATCAAATTTCCGGGGGCAAAGC  AAAATAA  TAAAATGAATGTCAGG  ATAT T TTA  TAGTGTCAGTCAG  TCACCCCACAGTC  TCCTCTCTCTTCTTTTCTAC
                                  (TATA-)                   (INR+)                    (MTE+)                        
Mouse     GGTCAGATTACAGGGGGCAAAAC  AAAATAA  TAAAATGAGTGTCCTG  GTAT T TCA  GAGAGTCATTCCG  CCATCCCCACAGT  CTCTCTCCTTCCTCTGCTGT
                                  (TATA-)                   (INR+)                    (MTE+)                        
    16 Human: AMPD2 (271, AK025706) 
Mouse: Ampd2 (109674, AK004759) 
 
           |-50      |-40      |-30      |-20      |-10      |1        |11       |21       |31       |41       
Human     GGGTCGGCAGGCGGA  GGCGGGGC  CAGGCGGGGGCGGGGCCAAGGGCCGCA G AGCCTGGCGCGGAG  CCGGCGAGATTTT  GGTGGGGTCTCACCTGTTGCGT
                           (BRE-)                                                 (MTE-)                          
Mouse     GCGTGGCCGAACGG  GGCGTGGC  CGCGGACTTGGCGGGGCCTGGGGTACGC G   AGATTGGC  GCGCAGCCCGGAA  GGTTTCGGCGGGG  TCGCACCTGTTGCGT
                          (BRE-)                                 (INR-)                    (MTE-)                   
    17 Human: AMPD2 (271, U16271) 
Mouse: Ampd2 (109674, AK034844) 
 
           |-50      |-40      |-30      |-20      |-10      |1        |11       |21       |31       |41       
Human     ATTTGGCGACGGTGGAATTTTCCCGGGACAGGCCCCTCCCTCCTGGGCTG G GATTCTCGGTTCTTCCCCCTCC  TCCCAGAGCTGGG  CTACCCCGGGAGCT
                                                                                        (MTE-)                  
Mouse     TCTGCCGGTCAGTGGAATTTTCCCAAGACAGGCCCCTCCCTCCTGGGCT  C G AATTCT  CAGTTCTGCCTCC  TCCCGGAGCTGGG  CTGTCCCTAGAGCAGGG
                                                             (INR+)                    (MTE-)                     
    18 Human: AMPD2 (271, NM_004037) 
Mouse: Ampd2 (109674, AK004759) 
 
           |-50      |-40      |-30      |-20      |-10      |1        |11       |21       |31       |41       
Human     AATCGTGGCCAGGGCCTCTTCCGCCTGCGGAGCCGCTGCTTCCTGCATCA G   TCACTCCC  GCTGGGGGC  GGGGCGGAGGAAG  GGGTTGGATGTGGCAGAGC
                                                               (INR+)                (MTE+)                       
Mouse     AAATCGTGGCCAGAGCCTCTTCCGCCTGCGGGGCGCTGCTTCCTGCATCA G   TCACTCCC  GCTGGGGGC  GGGGCGGAGGAAG  GGGTTGGATGTGGGAGAGC
                                                               (INR+)                (MTE+)                       
    19 Human: AMY2A (279, M18670) 
Mouse: Amy2 (11723, BG270172) 
 
           |-50      |-40      |-30      |-20      |-10      |1        |11       |21       |31       |41       
Human     GTAAAATGTGCTTCTTACAGGAA  TATAAAT  AGTTTCTGGAAAGGACACTG A   CAACTTCA  AAGCAAAATGAAGTTCTTTCTGTTGCTTTTCACCATTGGGT
                                  (TATA+)                        (INR+)                                           
Mouse     ATTTGCATTATGTATATTCAATG  TATGATT  CATTGCTAGAACAGA  TGTCT A CA  ACTTCAGGGCAGGCAAAATAAGATTTTTTTGTGTGTGTGTGTTGTAT
                                  (TATA-)                  (INR-)                                                 
    20 Human: AMY2B (280, NM_020978) 
Mouse: Amy2 (11723, BG270172) 
 
           |-50      |-40      |-30      |-20      |-10      |1        |11       |21       |31       |41       
Human     AAATGTGCTTCTTACAGGAA  TATAAAT  AGTTTCTGGAAAGGACACTGA  CA A CTTCA  AAGCAAAATGAAGTTCTTTCTGTTGCTTTTCACCATTGGGTTCT
                               (TATA+)                        (INR+)                                              
Mouse     GTACTTTTTGTAGAAA  TATAAAT  AGGCGCTAGAGAGAAAGAACACTGA  CA A CTTCA  AAGCAAAATGAAGT  TCGTTCTGCTGCT  TTCCCTCATTGGGTTCT
                           (TATA+)                            (INR+)                     (MTE-)                     
    21 Human: APOA2 (336, CR595207) 
Mouse: Apoa2 (11807, M79361) 
 
           |-50      |-40      |-30      |-20      |-10      |1        |11       |21       |31       |41       
Human     AGGGGGTGGGTAAACAGACAGG  TATATAG  CCCCTTCCTCTCCAGCCAGGG C AGGCACAGACACCAAGGACAG  AGACGC  TGGCTAGGTAAGATAAGGAGGC
                                 (TATA+)                                             (DPE+)                       
Mouse     GGTGGGGGTGGGGGAGTATATGTG  TATATAG  CCCCTACCTCCAGTCAAGC C C  AGAGTAGA  CGGGAAGGACT  GCAGCACAGAATC  GGTATGCTGAGGGAAC
                                   (TATA+)                        (INR-)                  (MTE+)                    
    22 Human: FASLG (356, D38122) 
Mouse: Fasl (14103, AK040139) 
 
           |-50      |-40      |-30      |-20      |-10      |1        |11       |21       |31       |41       
Human     GAGAAAGAGAAAGACAGAGGTGTTTCCCTTAGCTATGGAAACTCTA  TAAG A GAG  ATCCAGCTTGCCTCCTCTTGAGC  AGTCAG  CAACAGGGTCCCGTCCT
                                                          (INR-)                          (DPE+)                  
Mouse     AGAAGAGAACAGGAGAAAGGTGTTTCCCTTGACTGCGGAAACTTTATAAA G A  AAACTTAG  CTTCTCTGGAGC  AGTCAG  CGTCAGAGTTCTGTCCTTGACA
                                                                (INR+)               (DPE+)                       
    23 Human: ARF1 (375, M84327) 
Mouse: Arf1 (11840, AI048172) 
 
           |-50      |-40      |-30      |-20      |-10      |1        |11       |21       |31       |41       
Human     GTGCCCCGCGCCCGGA  GGCGCTGA  CGTGGCCGCCGTCAGAGCCGCCAT  CT T GTGGG  AGCAAAACCA  ACGCCTGGCTCGG  AGCAGCAGCCTCTGAGGTGAG
                            (BRE-)                            (INR-)                 (MTE-)                         
Mouse     GGCGTGCCCCGCGGCA  GGCGCTGA  CGTGGCTGCCGTCAGCGCCGCCAT  CT T GTGGG  AGCGAAACCA  ACGCCTGGCTGGG  AGCAGCCGCCGCGGAGGTGAG
                            (BRE-)                            (INR-)                 (MTE-)                         
    24 Human: RHOC (389, AK094474) 
Mouse: Rhoc (11853, AK011599) 
 
           |-50      |-40      |-30      |-20      |-10      |1        |11       |21       |31       |41       
Human     GCCCTCCCCGCCGC  GGCGCTGG  AGGAGGGCGGGGCGGGGCCCTGGGG  TCA G TCTG  AGCCTCCGGCACCGG  CCGCGCAGCTGGA  GGCGGCGGAGCGGAAGG
                          (BRE-)                             (INR+)                      (MTE-)                     
Mouse     GATGGACCCGCCCCAGAGCGGTGCTGGAGAGTGGGGCGGGGCCCGGG  TCA G TCTC  AGCCTCCGGCACTGG  CCGCGCAGCTGGA  GGCGGTGGAGCCCAAGG
                                                           (INR+)                      (MTE-)                     
    25 Human: ARNT (405, BC028362) 
Mouse: Arnt (11863, AK040475) 
 
           |-50      |-40      |-30      |-20      |-10      |1        |11       |21       |31       |41       
Human     GGTCAGGCGCCGCTTCTGGGGAG  TGGCCTT  TCTTTTCCCCTCCCTCCCGG T TCGGTGGCGGCGGC  TCCTCCCACTGGG  GGGGGGGGTGGCGCGGCGGCGG
                                  (TATA-)                                         (MTE-)                          
Mouse     GGCGGTAAGGTGCCTAATCTGCGGAGTGGCTCTTCCCTCCCCTCCC  CCAG C TCG  GTGGCGGCTGCCCCTC  CCACCGAGGGTGG  CGCAGGGACGGTGCCAT
                                                          (INR+)                       (MTE+)                     
    26 Human: ARNT (405, Y18859) 
Mouse: Arnt (11863, AK040475) 
 
           |-50      |-40      |-30      |-20      |-10      |1        |11       |21       |31       |41       
Human     GGGTCTCCCCGCCGCAGGGGCTGGGATGCTGGGGGCTGCTGAAGCCG  CCA T CTTG  GATTCCGCG  GTAGCGGAGGCGG  CGGTCAGGCGCCGCTTCTGGGGA
                                                           (INR+)                (MTE+)                           
Mouse     TTTGGGGCGTGTCTTCTGCCCAGGATGGGGAGGGGGGTGGGCGTCCG  CCA T CTTG  GATTCCGCG  GTAGCGGTGGCGG  CGGTAAGGTGCCTAATCTGCGGA
                                                           (INR+)                (MTE+)                           
    27 Human: SERPINC1 (462, U11270) 
Mouse: Serpinc1 (11905, BF385364) 
 
           |-50      |-40      |-30      |-20      |-10      |1        |11       |21       |31       |41       
Human     TCTGCCCCACCCTGTCCTCTGGAACCTCTGCGAGATTTAGAGGAAAGAA  C C AGTTTT  CAGGCGGAT  TGCCTCAGATCAC  AC  TATCTC  CACTTGCCCAGCC
                                                             (INR+)                (MTE-)       (DPE-)              
Mouse     GCTCCCTCCCCCACCTGGCCTCTGGACCTCTCAGATTTAGGGGAAAGAA  C C AGTTTT  CGGAGTGATCGTCTCAGTCAGCAC  CATCTC  TGTAGGAGCATCG
                                                             (INR+)                           (DPE-)              
    28 Human: ATF3 (467, CR614862) 
Mouse: Atf3 (11910, BC019946) 
 
           |-50      |-40      |-30      |-20      |-10      |1        |11       |21       |31       |41       
Human     GCCGCCAGCCTGAGGGC  TATAAAA  GGGGTGATGCAACGCTCTCCAAGCC  A C AGTCGC  ACGCAGCCAGGCGCGCA  CTGCACAGCTCTC  TTCTCTCGCCGCC
                            (TATA+)                            (INR+)                        (MTE-)                 
Mouse     CCTGCCAACGCGAGGGC  TTTAAAA  GGGGTGATGCAACGCGCTCCCAGCC  A C AGTCTC  ACTCAGCGAGACGCCGC  GCACGGTGCTTCC  CCAGTGGAGCCAA
                            (TATA+)                            (INR+)                        (MTE-)                 
    29 Human: ATP1A1 (476, NM_000701) 
Mouse: Atp1a1 (11928, AK078983) 
 
           |-50      |-40      |-30      |-20      |-10      |1        |11       |21       |31       |41       
Human     GCGGGGCCAGCACGCA  GGTTGCA  TATTTTAGGAAGTGAGGAGGAGGCGCG G GCTGGAGCTGCGGCGGGGTCT  GGGGCGCAGAGCA  GCGGCGGGAGGAGGC
                           (TATA-)                                                       (MTE+)                   
Mouse     GCGGAGCCATCACGCA  GGTTGCA  TATTTTAGGAAGTGAGGAGGAGGCACG G GCTCGAGCTGCGGCTGGGTCT  GGGGCGCGGAGCC  TCGGCGGGAGGAGGC
                           (TATA-)                                                       (MTE+)                   
    30 Human: ATP1A1 (476, NM_000701) 
Mouse: Atp1a1 (11928, AK078983) 
 
           |-50      |-40      |-30      |-20      |-10      |1        |11       |21       |31       |41       
Human     GAGCTGCGGCGGGGTCTGGGGCGCAGAGCAGCGGCGGGAGGAGGCGGA  CA C GTGGC  AACAGCGGTA  GCAGCCCGGGCGG  CGGCAGCAACAGCGGCGGCGG
                                                            (INR-)                 (MTE+)                         
Mouse     GAGCTGCGGCTGGGTCTGGGGCGCGGAGCCTCGGCGGGAGGAGGCGGA  CA C GTGGC  AGCGGCGGCA  GCAGCGGCGGCAG  CAGCGGCGGCCTCGGTCCGGG
                                                            (INR-)                 (MTE+)                         
    31 Human: ATP1A2 (477, Y07494) 
Mouse: Atp1a2 (98660, D90049) 
 
           |-50      |-40      |-30      |-20      |-10      |1        |11       |21       |31       |41       
Human     GGGAGAGGGGGAGAAGG  ACCTATT  TAAAGCTACCCTGTTGCTTTGGCTTT C TCTGTCTGCCAGG  GTCTCCGACTGTC  CC  AGACGG  GCTGGTGTGGGCTTG
                            (TATA-)                                              (MTE-)       (DPE+)                
Mouse     GAGTGAGGGGGAGAGGG  ACCTATT  TAAAGCTACCCTGTTGCTC  AGACTGT C   TCTGTCTGTCTGCCAGG  GTCTCCAGCTGCC  CCAGACAGGCGGTGTGGTC
                            (TATA-)                      (INR-)                        (MTE-)                       
    32 Human: ATP1B1 (481, M25161) 
Mouse: Atp1b1 (11931, AK010677) 
 
           |-50      |-40      |-30      |-20      |-10      |1        |11       |21       |31       |41       
Human     GCGCACGGCCGCCGG  GGCGCGG  T  ATATAG  TAAAGGTAGGGCGGGCGCAG  C C AATTCC  TCGGCTCCTGGCG  GGAGTGCCGGTGG  CGCCCCGCAGTCCGCTT
                           (BRE-)  (TATA+)                       (INR+)                    (MTE+)                     
Mouse     CGGATGGTGGA  GGCGCGG  T  ATATAG  TAAAGGTAGGGCGTGCGCCCTCAG  C C AATTTC  TCGGCTCCTGG  CCTGCGCGCCCGT  GTGCCGAGCCCGCGCTCCA
                       (BRE-)  (TATA+)                           (INR+)                  (MTE-)                       
    33 Human: ATP1B1 (481, NM_001677) 
Mouse: Atp1b1 (11931, BQ714103) 
 
           |-50      |-40      |-30      |-20      |-10      |1        |11       |21       |31       |41       
Human     GTCCCTCCCTCCGC  CCCCGCCC  CGCCGCCGCCGCCGCCGCCGCCTCCCCC T CCTCCTGCTCCTGCCTTGG  CTCCTCCGCCGCG  CGTCTCGCACTCCGAGA
                          (BRE+)                                                       (MTE-)                     
Mouse     GCGCCCTCCTC  CCTCGTCC  CTCCTCCCTCCGCCCCCGCCGCACCTCCCCC T CCTCCCGCTCTGC  CTAGGCTGCTCCG  CGGCGCGCCTCGCACTCGGAGAG
                       (BRE+)                                                    (MTE-)                           
    34 Human: ATP2B4 (493, BX537444) 
Mouse: Atp2b4 (381290, BF731559) 
 
           |-50      |-40      |-30      |-20      |-10      |1        |11       |21       |31       |41       
Human     GTGAGCAAGAGTCTGGCCCGAGTTAGCTAGAGCTGTTGCCATGACAA  GCA T TTTC  TTAAGAAAGCTCTGCTGTTG  AGATCG  TCCAAAGCTGGGGGCTGGG
                                                           (INR+)                       (DPE+)                    
Mouse     GGGAGCCCCAGTCTGGCCGGAGTTGGCTGGAGCTGTTGCCATGACAA  GCA T TTTC  TTAAGACAGCTCTGCTGTTGAGAC  TGTCCT  AGGCTGGGGGGTGGG
                                                           (INR+)                           (DPE-)                
    35 Human: ATP2B4 (493, M25874) 
Mouse: Atp2b4 (381290, BF731559) 
 
           |-50      |-40      |-30      |-20      |-10      |1        |11       |21       |31       |41       
Human     TATCCAGGAAGCTCTCCTCTTCCTCCTCCTGACGTCTACTACT  ACAGTTG C   TGGTTGTTGCTAAGGTTGCTGCCATGGTAACATGCACATCCTGTTTACA
                                                       (INR+)                                                   
Mouse     ATCCAGGGAGCCCTCCTCTTTTTCCTGACGCCAGCCTACCATCG  TAATTG C T  GATTGTTGCTAAGGTTGCTTCCATGGTAACATGCTTATCCTGTTTACA
                                                        (INR-)                                                  
    36 Human: ATP5F1 (515, NM_001688) 
Mouse: Atp5f1 (11950, AI037721) 
 
           |-50      |-40      |-30      |-20      |-10      |1        |11       |21       |31       |41       
Human     TCTCGCGATAATTTTT  TTTAAAA  ATCTCCCAAGGAAAGTTGAAGGA  AGAG T ACA  AAATTTTCATCTCGCGAGACTTGTGAGCGGCCATCTTGGTCCTGCC
                           (TATA+)                          (INR-)                                                
Mouse     GCGATAGTGGGGAGGG  GGCGGGG  A  AAAAGC  CGAGAACTTCCGAG  AAAAAG C G  AGCTGATCTCTT  CTCGCGAGATGTG  CGAGCGGCCATCTTGCTCCTGCC
                            (BRE-)  (TATA+)                 (INR-)                   (MTE-)                           
    37 Human: ATP6V0B (533, BC053601) 
Mouse: Atp6v0b (114143, AF356007) 
 
           |-50      |-40      |-30      |-20      |-10      |1        |11       |21       |31       |41       
Human     ACCGGCGTGGGGTCACGTGGTACCGGCGCATCACGTGGGCGAGT  TAGGTG A C  GCTGCGGGGCGGGCGGACAGACTGCG  GGACGG  ACGGTGGACGCTGGGA
                                                        (INR-)                             (DPE+)                 
Mouse     TGTGCGCGGGGGTCACGTGGGGTCGGCGCATCACGTGCGCCGGT  CAGGTG G C  GCTGCGGGGCGGGCCGACAGACTGCGGGGC  GGACGG  TGGACGCGGTTT
                                                        (INR-)                                 (DPE+)             
    38 Human: BCL9 (607, NM_004326) 
Mouse: Bcl9 (77578, AL363114) 
 
           |-50      |-40      |-30      |-20      |-10      |1        |11       |21       |31       |41       
Human     GGGGGGCGCAGAGAAGGGAGGGAGAATGTCTTGTTTGTGGCTTGTCAG  CA A TTGCT  TGAGACAAGCTTCCATGTGTGAAAGCTACTTGGCATGAATGCCT
                                                            (INR-)                                              
Mouse     GGGGGGTGCGGAGAAGGGAGGGAGAATGTCTTGTTTGTGGCTTGTCAG  CA A TTGCT  TGAGACAAGCTTCCATGTGTGAAAGCTACTTGGCATGAATGCCT
                                                            (INR-)                                              
    39 Human: BRDT (676, AY338951) 
Mouse: Brdt (114642, NM_054054) 
 
           |-50      |-40      |-30      |-20      |-10      |1        |11       |21       |31       |41       
Human     CCGCCCGCGGGGGGTCTTC  TATAAAA  GCGCCTGTCGCGCGACCG  CCATTA C A  ACAAAAACTGGCGGCGAGGAACTGCGGA  GAACTG  TTGCCCTGCACCGC
                              (TATA+)                     (INR+)                               (DPE-)               
Mouse     GGGTGAGTCCCATAA  AGGCGCCA  GTCGCTGGCTGCTGACAGCCG  CCATCC G G  GCGAGAGCTGGC  GGCCACCGGTGGA    GAACCG  GGAGCGACGGTCCTGCA
                           (BRE+)                         (INR+)                   (MTE-)     (DPE-)                  
    40 Human: C1QG (714, CR597625) 
Mouse: C1qg (12262, BC043945) 
 
           |-50      |-40      |-30      |-20      |-10      |1        |11       |21       |31       |41       
Human     AGGGGAGGGGGCCCAGCCCTGCTTTGGGCAATCCTTGCTCTGA  CCACTCA G   ACACCGTGTCCTCTTGCCTG  GGAGAGGGGAAGC  AGATCTGAGGACATCT
                                                       (INR+)                           (MTE+)                    
Mouse     GTGGGGGAGGGGGCTCTCCCTGCTCTGGGCGGTTCTTCTCTCACAGCTCT G AA  ACAGTATG  TTCCTGTCTGGGAGAACAGGA  CGTCTC  TGTGATTAGGCC
                                                                 (INR+)                        (DPE-)             
    41 Human: C4BPA (722, M62448) 
Mouse: C4bp (12269, M17122) 
 
           |-50      |-40      |-30      |-20      |-10      |1        |11       |21       |31       |41       
Human     GTTACGAAGAATGAGGACTAGCAAAGAGGAAGGAGCTTAGGTAA  ACAGTG C T  GCTTTATTTCTGCTGTTAATCATTCATTGGGCCCGTCAAAAGTTTCTG
                                                        (INR-)                                                  
Mouse     CACATGATGAAGAATGAGGGCTGGGAGAGAAGGAGATTAAATAA  ACACTG C T  GCTCTAGTCTGTTAATCAT  TCACCGGGACAGA  GGAGACTGTCTCCCTG
                                                        (INR+)                          (MTE+)                    
    42 Human: C8A (731, M16974) 
Mouse: C8a (230558, AB077295) 
 
           |-50      |-40      |-30      |-20      |-10      |1        |11       |21       |31       |41       
Human     CTGTGCAAATCAATGTGTATCTGGGTGAGTTTCCAACATCAGATA  GATCT T AC  AGGTCCCAGCCTGTAGACATCTTTTACTCCAATTTCCTGAATAGATA
                                                         (INR-)                                                 
Mouse     TTGAATGGGAACTGGGGGACTTTT  GACCATT  TTAAACATCCGGTAG  ACAA T TTG  GGTCCCAGCCTGGATAT  ATCTATGGCTTCT  AAGGTAGGCTCTTCTC
                                   (TATA-)                  (INR+)                        (MTE-)                    
    43 Human: CA6 (765, NM_001215) 
Mouse: Car6 (12353, AF079834) 
 
           |-50      |-40      |-30      |-20      |-10      |1        |11       |21       |31       |41       
Human     TAGCCAGCTGGGCAGATCAAT  TGTAAAT  TGTTATTTACAGTTTCTGAGCC T T  TAAAAGAA  GAGCTTCGCCGGAATCAGTCTTCATTACAGATGTGCAGCA
                                (TATA+)                           (INR-)                                          
Mouse     GCGGATCAGCCAGCTGGA  CCTGGTA  AATTGTTATTTACAGTTTCTGG  GCA T TTAA  AAGAAGTGCAGCCCAGAACC  TGTCCT  GCATTCAGGGCTACAGCAT
                             (TATA-)                         (INR+)                       (DPE-)                    
    44 Human: CACNA1E (777, NM_000721) 
Mouse: Cacna1e (12290, BY269288) 
 
           |-50      |-40      |-30      |-20      |-10      |1        |11       |21       |31       |41       
Human     TCTTTTTTTTTTTTTTCCTTCTTGAGGAATGGAGCTTCGCAGAGGTT  GCA T TTAG  ATTCAACAGTTC  ACAGCGGCGGGCT  GCTGCTGCTGCCTCTCCGAA
                                                           (INR+)                   (MTE+)                        
Mouse     TCTTCTTCTTCTTTTTTTTTCTTGAGGAATGGAGCTTCGCAGAGGTTGCC T   TTAGATTC  AACAGTTCA  GAGCAGGGCTGAA  GAGCCTGCAGGGGTCCCCA
                                                               (INR+)                (MTE-)                       
    45 Human: CACNA1S (779, L33798) 
Mouse: Cacna1s (12292, AF343753) 
 
           |-50      |-40      |-30      |-20      |-10      |1        |11       |21       |31       |41       
Human     AAGTCACTGGCTGGGCTGGGTGGAATGACAGCTGCCTGGCCTCAGAAGCT C AGGCCCGGCAGCGG  GGAGCCGAGTGGA  GGCTAATTTTACTTGCTGGGAG
                                                                                (MTE+)                          
Mouse     ATGTCACCGGCTGGACTGGAAGGAATGACAGCTGCCTGGCCGCAGGAGCC T GGGCTGGGCAGCAGGGGCTAAT  TTTACTCGCTGGG  AGCAGAGAGAGTAA
                                                                                        (MTE-)                  
    46 Human: CAPN2 (824, J04700) 
Mouse: Capn2 (12334, AK053832) 
 
           |-50      |-40      |-30      |-20      |-10      |1        |11       |21       |31       |41       
Human     GCCCAGCAGGCCG  GGAGCGGC  TGAGGCCACACCCCGCGGGCCGGG  CCGCT T CC  CTCCGGTGAATCATCGCTC  GCAGCGGCGGCGC  CCGCAGTGGCCGCAG
                         (BRE-)                            (INR+)                          (MTE+)                   
Mouse     GAGCAGGCCTGGGGAGGC  GGAGGCCA  CACCCCGCGCACGGCCCAG  GCGCT T CC  CGCCGGTGAATCATCCCCGCA  GCAGCGGCTCCCG  CAGTCCGCTGCAG
                              (BRE+)                       (INR+)                            (MTE+)                 
    47 Human: CAPN2 (824, BC007686) 
Mouse: Capn2 (12334, AK053832) 
 
           |-50      |-40      |-30      |-20      |-10      |1        |11       |21       |31       |41       
Human     CGCGCCCCAGCCGAGCGC  AGCGCGGA  GTCGCCCCGACCTTTCTCTGC  GCA G TACG  GCCGCCGGGACCGCAGC  ATGGCGGGCATCG  CGGCCAAGCTGGCGA
                              (BRE-)                         (INR+)                        (MTE+)                   
Mouse     CCCCAGCCGAGTG  CCGTGCCC  CGCCTCTCCGCGACCCCTTTCTCCGT  GCA G TGCG  CCAACCGGATCGCTACC  ATGGCGGGCATCG  CGATAAAGCTGGCCA
                         (BRE+)                              (INR-)                        (MTE+)                   
    48 Human: CAPZA1 (829, BX648738) 
Mouse: Capza1 (12340, BC016232) 
 
           |-50      |-40      |-30      |-20      |-10      |1        |11       |21       |31       |41       
Human     ATCTTGCCGCTATCGC  AGGCGCCA  GGAGCTGGGAGGGGAGAAGGACAGGA A ACCGGGAGTCGGGA  ATCCTGGGATAGT  GGCGGACCTGAAGTTGGCCTCT
                            (BRE+)                                                (MTE-)                          
Mouse     ATCTTGCGGCTCCGGCA  GGCGCCGG  GACCTGGGAGGGGAGAAGGGCCGGA A ACCCGGAGTCGGGA  ATCCTGGGATAGT  GGC  GGACTT  GAAGCTGGCCTCT
                             (BRE-)                                               (MTE-)        (DPE-)              
    49 Human: CAPZA1 (829, U56637) 
Mouse: Capza1 (12340, BC016232) 
 
           |-50      |-40      |-30      |-20      |-10      |1        |11       |21       |31       |41       
Human     CATCACTCGGCTTTCTTCCCGGCCTGCCTCGCGCCCGTAGCCGGGCTGGG C CA  GAACAGCC  CAAGATGGC  CGACTTCGATGAT  CGTGTGTCGGATGAGGA
                                                                 (INR-)                (MTE-)                     
Mouse     GCCACTCAGCTTCCTTCCCGGCCTGCCTCGCGCCGGCCCGCCGGGCTGGG   C CAGAGCA  GCCAAG  ATGGCCGACTTTG  A  GGATCG  GGTGTCGGATGAAGAG
                                                              (INR+)             (MTE-)      (DPE+)                 
    50 Human: CAPZB (832, NM_004930) 
Mouse: Capzb (12345, AK007209) 
 
           |-50      |-40      |-30      |-20      |-10      |1        |11       |21       |31       |41       
Human     GGCCGGGGCGGGGAAGGAAGGTGGCGGCGGCCCGGCGCGGGGGGAGGGGG G TGCTGACCCGGATGTTCACTCCTGGGCACCCGGGGAAGTGGAAGCGCCG
                                                                                                              
Mouse     GGAAGGAAGGTGGTGGCTGCCCGGCGCGGGGGGGTGGGGGGGGGAGGGGG G CGCTGACCCGGATGTTCACTCTTGGGCACCCGGGGAAGTGGAAGCGCAG
                                                                                                              
    51 Human: CASQ1 (844, NM_001231) 
Mouse: Casq1 (12372, NM_009813) 
 
           |-50      |-40      |-30      |-20      |-10      |1        |11       |21       |31       |41       
Human     CCCTACCCCAGCTAACCTCTTCTGGACCAGGAGAGCCAACCCAGATC  CCA C TACC  TCCATGAGTGCTACAGACA  GGATGG  GGCCCAGAGCTGTGCCGGGT
                                                           (INR+)                      (DPE+)                     
Mouse     CCCTATTTCAGCTAACCTCCTCTGGACCAGGAGAGCAGGCCCAGATT  GTA C TACC  TCCATGAGAGCTACCGACA  GGATGG  GGGCCAGAGCAGTGTCCGAG
                                                           (INR+)                      (DPE+)                     
    52 Human: CASQ2 (845, AK129891) 
Mouse: Casq2 (12373, AK048509) 
 
           |-50      |-40      |-30      |-20      |-10      |1        |11       |21       |31       |41       
Human     CGTGTGTGTGTGTGTGTGCCTC  TGCTCTT  TGTCCTGAGCCCACGATTCCA   G AGCTGGC  TGGACCCAAGGAGGTGAAG  AGTCAC  TTTTCAGCCCCAGGAAG
                                 (TATA-)                        (INR-)                      (DPE+)                  
Mouse     CGCATGTGTGCATTTGAGCCTC  CACAGTT  TGTTCTGAGCCCACGA  CTACA C AG  CTGCAGGACCAAGGAGGTGAAAGCAGCCTCTTTGTTCCCTCACCAAC
                                 (TATA-)                   (INR+)                                                 
    53 Human: RUNX3 (864, Z38104) 
Mouse: Runx3 (12399, AF169246) 
 
           |-50      |-40      |-30      |-20      |-10      |1        |11       |21       |31       |41       
Human     CCGCCCCCGGGGGAAG  CCGCGCCG  TCTCCGCCTGCCCGGCGCCCTGACGG C CGCTGTTATGCGTATTCCC  GTAGACCCAAGCA  CCAGCCGCCGCTTCACA
                            (BRE+)                                                     (MTE+)                     
Mouse     GCCTTCCCGCCTCCCGCTTCCCGCGGCTGCGGCCGCCCGCGCCCTGACGG C CGCGGCATGCGTATTCCCGT  AGACCCGAGCACC  AGCCGCCGCTTCACTC
                                                                                      (MTE+)                    
    54 Human: CD1D (912, NM_001766) 
Mouse: Cd1d1 (12479, M63695) 
 
           |-50      |-40      |-30      |-20      |-10      |1        |11       |21       |31       |41       
Human     TGAAACCTACTGAAGTGAGCGGCGGCGCCAGGATTCCTGGGACCCCGA  CC T CTTTG  CAGCTCGC  ACAGCTAAGGGCG  AGGGCGCCCTTCGGCAGAAGCAG
                                                            (INR+)               (MTE+)                           
Mouse     TGTTTAAAAGTGGTTTGCGTAGCGGTTCTGAGTAAAGCTGCACTCCAACC C T  CAACTACC  AGGGG  GTTTGGCTTTGTC  AAAGAGTAATTTACCAATCGTG
                                                                (INR-)            (MTE-)                          
    55 Human: CD3Z (919, NM_000734) 
Mouse: Cd3z (12503, J04967) 
 
           |-50      |-40      |-30      |-20      |-10      |1        |11       |21       |31       |41       
Human     CCTGGGGAGGTAGCTGCAG  AATAAAA  CCAGCAGAGACTCCTTTTCTCCTA A CCGTCCCGGCCACCGCTGC  CTCAGCCTCTGCC  TCCCAGCCTCTTTCTGA
                              (TATA+)                                                  (MTE-)                     
Mouse     GCCACAGAACAAAGCCAGCAGAGACTCCATCAGCGCCTCCTTTTCT  CCTC A TCC  TCCCAGGCATAGCTGC  CTCTGCCTCTGCC  TCTGGGTACCATCCCAG
                                                          (INR+)                       (MTE-)                     
    56 Human: TNFRSF8 (943, M83554) 
Mouse: Tnfrsf8 (21941, AK044269) 
 
           |-50      |-40      |-30      |-20      |-10      |1        |11       |21       |31       |41       
Human     AAATCCGG  GGCGGGCC  A  TTCAAAC  AGCTAAGGGAGGCGTCTCCT  AGTGTG C C  TTTTCCTGAGTCATCTCTGCACGTGTTTGCCCCCTTTTTTCTTCGCTG
                    (BRE-)    (TATA+)                       (INR-)                                                  
Mouse     CAAATCCGGGGCGGATCA  TTCAAAA  CGCAGGGGAGCCCCCTGCGTGCGTG C   GCTGTGGC  TAGCCCTGAG  TCATCTCTGCACG  TGGTGGCCCCTTCTCTCT
                             (TATA+)                             (INR-)                 (MTE+)                      
    57 Human: TNFRSF8 (943, M83554) 
Mouse: Tnfrsf8 (21941, AK044269) 
 
           |-50      |-40      |-30      |-20      |-10      |1        |11       |21       |31       |41       
Human     CCCCTTTTTTCTTCGCTGCTTGTAGCTAAGTGTTCCTGGAACC  AATTTGA T   ACGGGAGAACTAAGGCTGAAACCTCGGAGGAACAACCACTTTTGAAGTG
                                                       (INR-)                                                   
Mouse     CCCTTCTCTCTTAGGCTTCACAGGCAGGCAGATCGCTGCATCACAGGTGG   T AAGAGGG  TGGGGGAGGGAAG  GATTAGGGCTGAA  CCCGAAGAAGGGTTAG
                                                              (INR-)                    (MTE-)                    
    58 Human: CD34 (947, M81104) 
Mouse: Cd34 (12490, AK044121) 
 
           |-50      |-40      |-30      |-20      |-10      |1        |11       |21       |31       |41       
Human     GCGGAGGGGGCGGGAAGAGCGCGTCCTGGCCAAGCCGAGTAGTGTCTT  CC A CTCGG  TGCGTCTCTCTA  GGAGCCGCGCGGG  AAGGATGCTGGTCCGCAGG
                                                            (INR+)                   (MTE+)                       
Mouse     CACCCCTGCGCAGGGCGGAGGAGCGCGTCCCGCGCCGGGCCGCCTCCTGC A CCGAGCGCATCTCC  GGAGCGGTACAGG  AGAATGCAGGTCCACAGGGACA
                                                                                (MTE+)                          
    59 Human: CD53 (963, NM_000560) 
Mouse: Cd53 (12508, BC021310) 
 
           |-50      |-40      |-30      |-20      |-10      |1        |11       |21       |31       |41       
Human     TGTCGTCACAGCATGA  TCATATT  TTTTCACCCTTCACTTCTCCTTTTACA   C AAATAGC  CCCGGATATCTGTGTTACCAGCCT  TGTCTC  GGCCACCTCAAG
                           (TATA-)                              (INR-)                           (DPE-)             
Mouse     TGTCGTCACAGCATGA  TTGTATT  TTTTCTCTCTTCACTTCTCCTTTTACA   C AAATAGA  CATAGA  CTTCTGGGTTACA  GGCTGTGCTGGCCACCTAAAAGA
                           (TATA-)                              (INR-)             (MTE-)                           
    60 Human: CDA (978, L27943) 
Mouse: Cda (72269, AK008793) 
 
           |-50      |-40      |-30      |-20      |-10      |1        |11       |21       |31       |41       
Human     GCTGCGTACCTGAGAGCCTGCGGTCTGGCTGCAGGGACACACC  CAAGGGG A   GGAGCTGCAATCGTGTCTGGGGCCCCAGCCCAGGCTGGCCGGAGCTCCT
                                                       (INR-)                                                   
Mouse     TGGGAGCCAGCCTCCAGGTCTCCCTCTAGCGCAGGGACACACCCCGGG  GG A GGAGC  CACCTCTTCTTGGCCAGTGCGGGC  AGACGG  CCAGCACCGGTTTC
                                                            (INR-)                           (DPE+)               
    61 Human: CDC20 (991, NM_001255) 
Mouse: Cdc20 (107995, AK011723) 
 
           |-50      |-40      |-30      |-20      |-10      |1        |11       |21       |31       |41       
Human     CGAACCGAGACGACTCCAGGACGCTGAGGCAGCGCAGGCCCCACCCGGCC C CGCCCTGCCCCGCCCTGTC  CCGGCCGGCTTTC  CAGTACTAGTCCTCTGG
                                                                                     (MTE-)                     
Mouse     GGCCCAACCCAGAGAGATGGCCGGCAGAGAGCGGATCCCGAGGAC  TAACT G TG  CGGGACCCGCCCCCGTGTTCC  GATTCGGGCTCCG  ATTCGGGCAGGGA
                                                         (INR-)                            (MTE-)                 
    62 Human: CDC42 (998, NM_001791) 
Mouse: Cdc42 (12540, AK051543) 
 
           |-50      |-40      |-30      |-20      |-10      |1        |11       |21       |31       |41       
Human     CAGTCCAGGCACCGCCTTGACCCGCCCCAGCCTCTTCCCCTTCCCTGTTC C TC  CCACTTCC  GCGGGCACCCAACTGTG  CGTCTC  CTGCGCGCTGACGTCA
                                                                 (INR+)                    (DPE-)                 
Mouse     GCCAAGGGGCGGAGC  CCGAGCCG  CCCTCAGCCCCTTCTCTCTCTTTGTCC C TC  CCACTTCC  GCGGGCACCCAACCATG  CGTCCC  CTGCGTGCTGACGTCA
                           (BRE+)                                  (INR+)                    (DPE-)                 
    63 Human: CDC42 (998, AY673602) 
Mouse: Cdc42 (12540, AK051543) 
 
           |-50      |-40      |-30      |-20      |-10      |1        |11       |21       |31       |41       
Human     CCCAACTGTGCGTCTCCTGCGCGCTGACGTCAGGTGCGTGCCCCTGTCCG G CAGCCGAGGAGACCCC  GCGCAGTGCTGCC  AACGCCCCGGTGGAGAAGCT
                                                                                  (MTE-)                        
Mouse     CCCAACCATGCGTCCCCTGCGTGCTGACGTCAGGCGCGTGCCCCTGTCCG G CAGCCGAGGAGGCCCC  GCGCAGTGCTGCC  AACGCCCCGGCGGAGAAGCT
                                                                                  (MTE-)                        
    64 Human: CDC42 (998, AL121737) 
Mouse: Cdc42 (12540, AK081384) 
 
           |-50      |-40      |-30      |-20      |-10      |1        |11       |21       |31       |41       
Human     CGGGGGAGGGGAGT  GGCGTTGC  GGCCGCGGCGGGGCGGTGGCAGGAGG  GC A GCTCG  CTGGCTGC  TCGCGGCGGTGCC  TGTCCGAGCTCCCCTCCGCGCCG
                          (BRE-)                              (INR+)               (MTE-)                           
Mouse     GGGAGCGGAACTGCGGCCGCAGGGCCGGGCCGGGCCGGGGCCGAGG  TGAC G GAC  CGGGCGGCTCTG  CGCCTGCGCCCCT  CGGCGGTCTCCAGCCCCGCGC
                                                          (INR-)                   (MTE-)                         
    65 Human: CDKN2C (1031, AK091170) 
Mouse: Cdkn2c (12580, BC027026) 
 
           |-50      |-40      |-30      |-20      |-10      |1        |11       |21       |31       |41       
Human     TGTCACACCCAGTCCCAAGCTCCCTGACGTCTGCATCCTCAGACTCCG  TA A CACAG  AAGTAACCAGACTGCCATGGTAGACATGGGAGAGACGAGGAAGC
                                                            (INR+)                                              
Mouse     CACTCCCTACCTGGAACC  TCCTATT  TCTGAGTCTGCAATCTCAGGC  ACAA A CAC  TGAGGTATCCAGCAGACAATAATA  TGTCTG  GAACATAGAAACTCAG
                             (TATA-)                        (INR+)                           (DPE-)                 
    66 Human: CDKN2C (1031, AY094608) 
Mouse: Cdkn2c (12580, BC027026) 
 
           |-50      |-40      |-30      |-20      |-10      |1        |11       |21       |31       |41       
Human     GGGCGGGGCGTGGG  CGGCGCCC  GGCCTTCCCGCTCCCGCGGCGCTG  CAAC T CTG  CCGAGCCTCCTT  AAAACTCTGCCGT  TAAAATGGGGGCGGGTTTTTC
                          (BRE+)                            (INR+)                   (MTE+)                         
Mouse     GGGCGGGGCGTG  GGCGGGGC  CCGGCCTTCCCGCTCCCGCGGCGCCC  TAAC T CGG  CGGAGCCTCCTTAAAA  CTCTGCCGTTAAA  ATGGGGGCGGGTTTTTC
                        (BRE-)                              (INR+)                       (MTE-)                     
    67 Human: CDKN2C (1031, AK021794) 
Mouse: Cdkn2c (12580, BC027026) 
 
           |-50      |-40      |-30      |-20      |-10      |1        |11       |21       |31       |41       
Human     ATGGGGGCGGGTTTTTCAACT  CAAAAAG  CGCTCAATTTTTTTCTTTTCAA   A AAAAGCT  GATGAGGTCGGAAAAAAGGGAGAAGAAACCGGCACCCTCTCT
                                (TATA+)                         (INR-)                                            
Mouse     ATGGGGGCGGGTTTTTCAACT  CAAAAAG  CGCTCAATTTTTTTCTTTTCAA   A AAAAGCT  GATGAGGTCGGAAAAAAGGGATAAGAAACCGGCACAGTACCT
                                (TATA+)                         (INR-)                                            
    68 Human: CHI3L1 (1116, Y08374) 
Mouse: Chi3l1 (12654, NM_007695) 
 
           |-50      |-40      |-30      |-20      |-10      |1        |11       |21       |31       |41       
Human     TTGACACATAGCTCAGTTCC  CATAAAA  GGGCTGGTTTGCCGCGTCGG  GGA G TGGA  GTGGGACAGGTATATAAAGGAAGTACAGGGCCTGGGGAAGAGGCC
                               (TATA+)                       (INR-)                                               
Mouse     GTTTACACACACACAGTTCC  TGTAAGG  AGGGCAGTTTGGGGGGCGGG  GGA G TGAG  TGAGATGGGATGGGTATAAGAAGG  AGATGT  GGAGCCTAAGGAAGA
                               (TATA+)                       (INR-)                           (DPE+)                
    69 Human: LYST (1130, L77889) 
Mouse: Lyst (17101, L77884) 
 
           |-50      |-40      |-30      |-20      |-10      |1        |11       |21       |31       |41       
Human     CCCGTTACCAAACGAAAAAGCAACGCAAGGGCTTCTAAGAAGCCATC  CCA A TGAC  CTTTTGGCTTTGAGA  AGAGCAGTCCTCA  TACCAGAGTGTTTGGGG
                                                           (INR-)                      (MTE+)                     
Mouse     ACCACCAGCGGGTGAAATGGCAGCATGGGGCTTTCAGAAAGCCATTCCCA A GG  ACGATTTG  GCTTGGAGA  GGAGCAGGAAGAG  TGCCAGAGTTGCTGATG
                                                                 (INR+)                (MTE+)                     
    70 Human: CHRM3 (1131, AF331832) 
Mouse: Chrm3 (12671, NM_033269) 
 
           |-50      |-40      |-30      |-20      |-10      |1        |11       |21       |31       |41       
Human     GCCGCTTGGGCAGGTG  CCGCGGCC  GCTGCCCGGAGGCGGCATGTGACGCG C GGCCGCAGCTGCCCGCGG  GCGGAGCGCTCTC    AGACCC  CGGAGCGCACAC
                            (BRE+)                                                    (MTE-)     (DPE+)             
Mouse     GTCGCCCGGGCAGGTG  CCACGGCT  GCTGCCGAGAGGCGGCATGTGACGCG C GGCCGCAGCTGCCCGCGGGC  GGAGCGCGCTCGG  ACCCGGGAGCGCAACC
                            (BRE+)                                                      (MTE+)                    
    71 Human: CHRNB2 (1141, U62437) 
Mouse: Chrnb2 (11444, AK051742) 
 
           |-50      |-40      |-30      |-20      |-10      |1        |11       |21       |31       |41       
Human     CCCCCCTTTTTTTTCCTGGGACCCAGCGTTTCCGCCTCCGGGGC  GCAGAC T C  CTCCCCCTCACCGTCCCAATTGTATTCCCTGGAAGAGCAGCCGGAAAA
                                                        (INR+)                                                  
Mouse     ACCACCTTTTTTTCCCTGGGATCCCGCGCTTCCGCCTCCGGGGCGG  AGAC T CCT  CCCCTAGTAGTTCCACTTGTGTTCCCTAGAAGAGCAGCCGGGACCG
                                                          (INR+)                                                
    72 Human: CKS1B (1163, NM_001826) 
Mouse: Cks1b (54124, AB025409) 
 
           |-50      |-40      |-30      |-20      |-10      |1        |11       |21       |31       |41       
Human     TGTCCCGCCTCGAGCCAAACGGACCAATAGGAAGTGCGGGCGGCG  TGTTT G AA  AGCGAGGCCAAAGTGGGTG  GGAGCGCGTGCTG  TTGGGAGTTGCTTGG
                                                         (INR-)                          (MTE+)                   
Mouse     TGTCCCGCCTTCGGCCGCAGAGACCAATAGGAAGTGCGGGCGGTG  TGTTT G AA  AGCGAGGCCAAAGAGGGTG  GGAGCGCGTGCTG  CTGGGAGTTGTCTGG
                                                         (INR-)                          (MTE+)                   
    73 Human: CLCN6 (1185, AL707709) 
Mouse: Clcn6 (26372, NM_011929) 
 
           |-50      |-40      |-30      |-20      |-10      |1        |11       |21       |31       |41       
Human     TCTCAGTCCCAGAGCTCGAGAGGGCCACGTGACCGTCCCGGGG  CCAGTCA C   GTGAGGCGCAGATCCTGGCTG  GGAGGGGGTTGGT  AGAGGGGTCCAGAGT
                                                       (INR+)                            (MTE-)                   
Mouse     CTCCGGACCCAGAGCTCGAGAGGGCCACGTGACCCTCCCGGGG  CCAGTCA C   GTGAGGCGCGGATCCTGGGTGG  GAGGGGGGTTGGC  CTAGGTGGTCCGGA
                                                       (INR+)                             (MTE-)                  
    74 Human: CLCNKA (1187, BC035373) 
Mouse: Clcnka (12733, BC037077) 
 
           |-50      |-40      |-30      |-20      |-10      |1        |11       |21       |31       |41       
Human     CTAGAATGCCTTCTG  GGCATGGG  CTCCTGGCTGCAAGCTGTCCAGCACGG C CC  CGTTTGAT  CCCCAC  ACAGCTGGGAGGG  TGGAGGGGCTCTTCCCATGT
                           (BRE-)                                  (INR-)             (MTE+)                        
Mouse     GCTCCGGCTTCCACGATGTCTCTGGCCACTGCTGTCTCTTCCCAGCAGAG C   CCTCTTTG  ATCTTAAC  ACAACTCAAAAAG  AGGAGCTTTGAGGCCAGTTT
                                                               (INR+)               (MTE+)                        
    75 Human: CLCNKA (1187, NM_004070) 
Mouse: Clcnka (12733, BC037077) 
 
           |-50      |-40      |-30      |-20      |-10      |1        |11       |21       |31       |41       
Human     GTGATGATTGGTAGAG  CATATAC  CTGTCCAGGTGCACTGGAGCTGGGAG  C A AAGGAG  GCTCTGTGAGAGGAGGGCCAGTTCAGCCGCAGCAGGAGGACTG
                           (TATA+)                             (INR-)                                             
Mouse     GTGTTGATTGGTGGAGG  GCACACCT  GTCCAGGTGTACAGGAGTT  ACAAAC A A  AGAAGCTGTGTTGGAGGAGGGCCTGTTGGGGCTTCAGCAGGGCACACT
                             (BRE+)                       (INR+)                                                  
    76 Human: CLCNKB (1188, NM_000085) 
Mouse: Clcnkb (56365, BC019983) 
 
           |-50      |-40      |-30      |-20      |-10      |1        |11       |21       |31       |41       
Human     AGGACATTCTAAGTGTTCGC  CATAAAC  CCTGGGCCCAGGCGGG  AAAGGGG G   GAGGATGTTGATTGTTGGAACACACACCTGTCCAGGTGCAGGGGAGCTG
                               (TATA+)                   (INR-)                                                   
Mouse     CAGTTTCTCAAGGACAT  TCTAAAT  GGCTTCCTTAACCCCAGGAGGGGAGG G G  AGGGTGTT  GATTGGTGGAGGGCACACCTGTCCAGGTGTACAGGAGTTA
                            (TATA+)                               (INR-)                                          
    77 Human: PLK3 (1263, AY764184) 
Mouse: Plk3 (12795, BC063051) 
 
           |-50      |-40      |-30      |-20      |-10      |1        |11       |21       |31       |41       
Human     CCAGACGCCGGGGAGGGGGCAGGC  GTTCATT  GATAAAACGCTGGGCTCCC C TGGGCGCCAGCGCAGCGTAGC  AAATCCAGGCAGC  GCCACGCGCGGCCGG
                                   (TATA-)                                               (MTE+)                   
Mouse     GAAACACCGGGGAGGGGGCAGGG  GTTCATT  GATAAAAACGTCGGACTC  CC C CTTGC  CCTGGCGCGATC  GCAGCGTAGAGAA  ACCAGGCAGCGCCACGCGC
                                  (TATA-)                     (INR+)                   (MTE+)                       
    78 Human: PLK3 (1263, U56998) 
Mouse: Plk3 (12795, BC063051) 
 
           |-50      |-40      |-30      |-20      |-10      |1        |11       |21       |31       |41       
Human     CCCCTTCC  AGCGTGCG  GCCGCCGCGCCCGCTCCCCCGGCCGGGCCCGGGC C GCCTCCGAGTGCCTTGCGCG  GACCTGAGCTGGA  GATGCTGGCCGGGCTA
                    (BRE-)                                                              (MTE-)                    
Mouse     CCCCTTCCCG  CGCGCGGC  TGCCCCGTCCGCGCCCCCCGCCGGCCCGGGGC C GCCTGCCAATGCC  TCCCCGCGATCG  G  AACCT  GAGGTGCTGGCCGGGCCA
                      (BRE-)                                                     (MTE-)    (DPE-)                   
    79 Human: CNN3 (1266, NM_001839) 
Mouse: Cnn3 (71994, AK051628) 
 
           |-50      |-40      |-30      |-20      |-10      |1        |11       |21       |31       |41       
Human     GGAGGCGGCCCTCCATTGGTCGGGCCCGGCGGCCAATCCTCGAAGGCCCA G GCGGAGGCGGGGGCGGCCTGTGG  GGAACCGAGGTGC  GGGCGGCGAGCGA
                                                                                         (MTE+)                 
Mouse     AGGAGGCGGCGCTCATTGGTCGGGCTCGCCGGCCAATCCCTGGAGGCCCG G GGGTGGGTGGGGGG  AGACCAGGCTGGG  GGCAGAGCGTGCGGAGCCGCGG
                                                                                (MTE-)                          
    80 Human: CNR2 (1269, X74328) 
Mouse: Cnr2 (12802, AK037898) 
 
           |-50      |-40      |-30      |-20      |-10      |1        |11       |21       |31       |41       
Human     AGCTGGCTT  GGGGTGGC  ACTCAACAGGTGCTCTGAGTGGCACCCACGGCC A GGTCCTGGGAGAGGACAGAAAA  CAACTG  GGACTCCTCAGCCCCCGGCAG
                     (BRE-)                                                           (DPE-)                      
Mouse     AACCCCAGGCT  GGAGCTGC  AGCTCTTGGGACCTACGTGGGGGTCCCTGCT G GGTCTCCAGATCTGGATACAGAATAGCCA  GGACAA  GGCTCCACAAGACC
                       (BRE-)                                                                (DPE+)               
    81 Human: COL11A1 (1301, NM_001854) 
Mouse: Col11a1 (12814, AK081304) 
 
           |-50      |-40      |-30      |-20      |-10      |1        |11       |21       |31       |41       
Human     GCCCGGAGCCACTCGTCCAGCCCACTGACGGCATGAAGCCTTTAGGG  GCA C ACAG  TACTCTCAGCTTGTTGGTG  GAAGCCCCTCATC  TGCCTTCATTCTG
                                                           (INR+)                          (MTE+)                 
Mouse     TGCGCGAGCCTCTTGTCCAGCCCACCCACAGCATGAAGCCTTTAGGGGTG C   TCAGTGCT  TTCGGCTTTTTTTCTTGAAACCCTTCATCTGCCCTCAGTCT
                                                               (INR+)                                           
    82 Human: COL16A1 (1307, BC064839) 
Mouse: Col16a1 (107581, BB857359) 
 
           |-50      |-40      |-30      |-20      |-10      |1        |11       |21       |31       |41       
Human     CCCCCACCC  GGAGTGGC  CGGCAGTCCCCGGCAGGCGTGCGCCGGGCAGCA G ACAGCCGGGAGAGCT  CGAGCGCCAGGAA  GCTGGGGACCCGTGACCGTCA
                     (BRE-)                                                        (MTE+)                         
Mouse     CGGCAGTTCCTGCAAGCA  GGCGTGTG  AGTGTGCGCGCACGCTGCGCT  GCA C TCAG  TCTAGAGAACTCAGGACG  TGGAAAAGCTGGG  GTCAGCGCAGACTG
                              (BRE-)                         (INR+)                         (MTE-)                  
    83 Human: COL16A1 (1307, NM_001856) 
Mouse: Col16a1 (107581, BB857359) 
 
           |-50      |-40      |-30      |-20      |-10      |1        |11       |21       |31       |41       
Human     GGCGGGCCTGGGAGCTCGGCCAGACCCGGGGCGGGGCTGTGCACCCC  GCA G TCTA  GCCGCCTCTGGTGCCGCT  GGAGCCCCCTCTA  GGCCCTCCTCCTCC
                                                           (INR+)                         (MTE+)                  
Mouse     GGCGGGCCCG  GGCGCAGG  GCCAGGCCAGGAGCGCGGCGGTGCAGG  CCAAT G GC  CAGTGCCTCCGCA  GCCGCCGAACCCT  CGC  GGTCTC  CCTCCTCCTCCA
                      (BRE-)                               (INR-)                    (MTE+)        (DPE-)             
    84 Human: COPA (1314, NM_004371) 
Mouse: Copa (12847, AK043479) 
 
           |-50      |-40      |-30      |-20      |-10      |1        |11       |21       |31       |41       
Human     TGGCCCGGAGCCTAAGAGACCGGAAGTTCGTGTTTCCAGGCGCTTCCGGA A ACCGCGGGAGAGGGTCGCTGACGTGGAGGCGTCCGAAGGGCAGCAGGGT
                                                                                                              
Mouse     GTGGCCCAGCGCTCCAGAGACCGGAAGTCCTTGTCCGAGGAGCTTCCGGG A GCCGCGACCCGGGA  GTAGCTGACGTGG  AAGCCTTGGGAGTGCCACCGGG
                                                                                (MTE+)                          
    85 Human: COPA (1314, U24105) 
Mouse: Copa (12847, AK043479) 
 
           |-50      |-40      |-30      |-20      |-10      |1        |11       |21       |31       |41       
Human     CAGGAAGAGGCCTAGAGAAGGGAGTGAAGCGACACGACGTGCTGCGGGGG A   GAAGGGAC  CTTCAGGTCCAGGCAAAGGGG  GAACTT  CTGTCGTGGGAACG
                                                               (INR-)                        (DPE-)               
Mouse     AGAAAGGTTGAAGAGAATCAGGCTGGAAACCCAGAAAAGGGCTGGAGA  GA A CTAAG  GAAATGCAGGTCCAGGCAAAGGAG  GAACTT  ATGTCATGGGAACG
                                                            (INR-)                           (DPE-)               
    86 Human: CPT2 (1376, U09642) 
Mouse: Cpt2 (12896, NM_009949) 
 
           |-50      |-40      |-30      |-20      |-10      |1        |11       |21       |31       |41       
Human     CTGCTTTCCTATTAAGCACCATGCACTGTGCATAACATGTTATCTTTATC   T CATATCT  AGAATTTTGGGAGGTACTTTGAATCATTACGATCTATCTACT
                                                              (INR+)                                            
Mouse     ACACGGACCTAGTATG  AGTAAAG  TATTCTGCCTAACAAATTATATTGGTG A AA  TAAGTATG  ACTATTGTTCAGTAAATCTA  GAACTG  TGGAGGCGTTTGT
                           (TATA+)                                 (INR-)                       (DPE-)              
    87 Human: CPT2 (1376, NM_000098) 
Mouse: Cpt2 (12896, NM_009949) 
 
           |-50      |-40      |-30      |-20      |-10      |1        |11       |21       |31       |41       
Human     GGGCGGAGCCGATGGCCTTACAGGGGCCGGAAGTGGCCTGCGGGCGGA  GA A GTGCC  TCAGGAGTCCTGACGCAG  TGTCTT  GGGCGCTAACGGCGGCGGCG
                                                            (INR-)                     (DPE-)                     
Mouse     GGGCGGGGCCACCGCCTATCGGTGCCCGGAAGTGCCCCTGTGGGCGGAGT T   GAACTGGA  CGTTAAGTCTCTACGGG  TGTCCC  GGGCCTGAGGGCAGCGGC
                                                               (INR-)                    (DPE-)                   
    88 Human: CRABP2 (1382, NM_001878) 
Mouse: Crabp2 (12904, M87538) 
 
           |-50      |-40      |-30      |-20      |-10      |1        |11       |21       |31       |41       
Human     GGGCGGTTCCGTACAGGG  TATAAAA  GCTGTCCGCGCGGGAGCCCAGG  CCA G CTTT  GGGGTTGTCCCTGGACT  TGTCTT  GGTTCCAGAACCTGACGACCCG
                             (TATA+)                         (INR+)                    (DPE-)                       
Mouse     CGCCTCATGCACCAGCTGGG  TATAAAA  GCCTAGAGCTCTGGCGCCCAGCG   G CTGTGCA  ATTGTCCT  CCACGCGGCTTGG  TCCCCAGGTGGAAGGATCTGT
                               (TATA+)                          (INR-)               (MTE-)                         
    89 Human: CRP (1401, AF449713) 
Mouse: Crp (12944, X13588) 
 
           |-50      |-40      |-30      |-20      |-10      |1        |11       |21       |31       |41       
Human     TTGGATATAAATCCAGGCAGGAGGAGGTAGCTCTAAGGCAAGA  GATCTAG G   ACTTCTAGCCCCTGAACTTTCAGCCGAATA  CATCTT  TTCCAAAGGAGTG
                                                       (INR-)                                 (DPE-)              
Mouse     GCTATAGTTATAAATCTGAGGATGGGCTGGGCCCGAGGCAAGCGTTCCA  G G ACTCCT  TGTCCTTGATCTTTC  AGACAA  AACACTGTCCTCTTAGTCCAGA
                                                             (INR+)                  (DPE+)                       
    90 Human: CRYZ (1429, NM_001889) 
Mouse: Cryz (12972, NM_009968) 
 
           |-50      |-40      |-30      |-20      |-10      |1        |11       |21       |31       |41       
Human     GAAGGCAGGCGATGGACAGGCGCAAAGGGGCCTCACCCGGTCAA  GGAGTA G G  ATGCGGCCAGCGCA  GGAGCTGGTGGTG  AAAAACTGATCTCGCATGTTC
                                                        (INR-)                     (MTE+)                         
Mouse     TTAGCATTGCACAGCCTCCCCCGGCCCGGGCCTCACCCCGTCCA  GAAGGA G G  ATGCGCCCTGCGCA  GGAGCTGGTGGTG  AAAAACTCTTCCCGCGAGTTC
                                                        (INR-)                     (MTE+)                         
    91 Human: CSF1 (1435, NM_172211) 
Mouse: Csf1 (12977, BC025593) 
 
           |-50      |-40      |-30      |-20      |-10      |1        |11       |21       |31       |41       
Human     TCAGGCCTGGAGGGAAAGTCCCTTGGGACGATCATAGAGCGCTA  GCACTG A A  TCAGCCTGGAGAGCGCGGAAGGAAAGGGTCGGTCCGCAGAGGGCGCGG
                                                        (INR-)                                                  
Mouse     TTGAGGGAAAGTCCCTAGGGGCCAGCATTAGACCGAGAACAATCACACG  A G ATTGAC  AAGAGTGG  CGAGCACAGAAGG  AAAGGCAGTGTCTTGTCCAGAG
                                                             (INR-)               (MTE+)                          
    92 Human: CSF1 (1435, X06106) 
Mouse: Csf1 (12977, BC025593) 
 
           |-50      |-40      |-30      |-20      |-10      |1        |11       |21       |31       |41       
Human     ACCACATGCCCCCCAGT  CCTCTCT  TAAAAGGCTGTGCCGAGGGCTGG  CCA G TGAG  GCTCGGCCCGGGGAAAGTGAAAGTTTGCCTGGGTCCTCTCGGCGC
                            (TATA-)                          (INR-)                                               
Mouse     ACATGCCCCGCCAGCCC  GCCCGCT  TAAAAGGCTGTGCCGAGGGCTGGCCA G CGAAGCTCGGCCAGGGGAAAGTGAAAGTTTGCCTCGGTGCTCTCGGTGT
                            (TATA-)                                                                             
    93 Human: CSF3R (1441, NM_000760) 
Mouse: Csf3r (12986, AK089305) 
 
           |-50      |-40      |-30      |-20      |-10      |1        |11       |21       |31       |41       
Human     TCGGAAGGTGTTGCAATCCCCAGCCCCCGGGCCTGTCAGAGGCTGAG  CCA T TAAC  GACAGAGCTCGGGGAGA  GAAGCTGGACTGC  AGCTGGTTTCAGGAA
                                                           (INR+)                        (MTE+)                   
Mouse     AAGCCAGGAA  GGTGTCGC  AATTCTGGGTCCCCCCCACCCCCCGCCTG  TCA T TAAC  GACGGGGCTAG  AAAGAGAAGTTTC  GG  GAACTT  GCCCCAAGAGGAG
                      (BRE-)                                 (INR+)                  (MTE+)       (DPE-)              
    94 Human: CSRP1 (1465, M33146) 
Mouse: Csrp1 (13007, AK087436) 
 
           |-50      |-40      |-30      |-20      |-10      |1        |11       |21       |31       |41       
Human     CACGCCTTCCGC  GGAGCGG  A  ACAAAA  CGGCGCGCAGGCCGGGCGCACCCA G CCGCCACTTCCGAGAGC  GCCTGCCGCCCCT  GCGCCGCCGAGCCAGCTGC
                        (BRE-)  (TATA+)                                                (MTE-)                       
Mouse     AAACGCCTTCCGGAGGGGGA  AACAAAA  CGGCGCGCACTCCGGCGCAG  CCA G TCAC  CGCTTCCCTG  AGAGCTGTCGCCC  GTGCGCCTCCCAGCAGCTGCCA
                               (TATA+)                       (INR+)                 (MTE+)                          
    95 Human: CTPS (1503, NM_001905) 
Mouse: Ctps (51797, BC006698) 
 
           |-50      |-40      |-30      |-20      |-10      |1        |11       |21       |31       |41       
Human     CCGCCCCGCGCCCT  CCAGGCCC  GGCCCGCCCTCCAACCTCTGCGTGC  GCA C AGCC  TAGAGCCCGCCTCCGTGAAAGACTGCCGGGCGCATGCGGTCGGGG
                          (BRE+)                             (INR+)                                               
Mouse     CTAGCCCCAC  CCTCCCCT  GGGCCCGCCTCTCCCCGCCCTCTGCGTGCGCA C CGCCTAGAGCCCGCCTCTCAGAA  AGACGG  CTGGCGCATGCGGCCCGGCC
                      (BRE+)                                                           (DPE+)                     
    96 Human: CTSE (1510, AJ250716) 
Mouse: Ctse (13034, BY057666) 
 
           |-50      |-40      |-30      |-20      |-10      |1        |11       |21       |31       |41       
Human     TTTACTCTG  GGCATGGC  TAATGACTCCAGGGCCTTATCATTCGGCCCTC  A G ACTGGG  CTGGGCAGGTCTGAG  AGTTAG  GGAAAGTCCGTTCCCACTGCCC
                     (BRE-)                                    (INR-)                  (DPE+)                       
Mouse     GTTTACTCTG  GGCATGGC  TATTGACTCCAGACCTTATCATTGGGTCCTC  A G ACTGGG  CAGGGCAGGTCTGGGCACCAGGAAGAGCCAGTGCCCCTGCCCT
                      (BRE-)                                   (INR-)                                             
    97 Human: CTSK (1513, U20280) 
Mouse: Ctsk (13038, AJ006033) 
 
           |-50      |-40      |-30      |-20      |-10      |1        |11       |21       |31       |41       
Human     CCTCTGTTTCCCTGCCAAATGGAAGAGTTTTCCCTAACTACATTCTTCTG C AG  GATGTGGG  GGCTCA  AGGTTCTGCTGCT  ACCTGTGGTGAGCTTTGCTC
                                                                 (INR-)             (MTE-)                        
Mouse     CAACCTGTGAATTTCTGCTAGTGGAAATCTCCCCTAATTATTCCTTCCGC C AG  GATGTGGG  TGTTCA  AGTTTCTGCTGCT  ACCCATGGTGAGCTTTGCTC
                                                                 (INR-)             (MTE-)                        
    98 Human: CTSS (1520, CR612707) 
Mouse: Ctss (13040, AK028366) 
 
           |-50      |-40      |-30      |-20      |-10      |1        |11       |21       |31       |41       
Human     TGTGACAAGTTCCAA  TTTCTTT  TCAAGTCAATTGAACTGAAATCTCCTTG T TGCTTTGAAATCTTAGAAG  AGAGCCCACTAAT  TCAAGGACTCTTACTGT
                          (TATA-)                                                      (MTE+)                     
Mouse     ATGTGACAAGCTCCG  ATTTCTT  TTTAGAGTCATTTCAAGCAGCCTCCTGC T GCCAGGGACATCTTTGGAG  TGAGCACCACACT  TCAGGATGACCATGAGG
                          (TATA-)                                                      (MTE+)                     
    99 Human: CTSS (1520, S39127) 
Mouse: Ctss (13040, AK028366) 
 
           |-50      |-40      |-30      |-20      |-10      |1        |11       |21       |31       |41       
Human     CCTCAGCCTCCCAAAGTG  CTGGGAT  TATAGGCATGAGCCACTGGGC  TCAG A CAA  GGGCTCTTCTTGATGGCTTACTGTATCCACTTTGTCCCCAAGACCA
                             (TATA-)                        (INR+)                                                
Mouse     GGAAAACTTTGTCCTGTTCTCTGCTGAGGTCATTCTATTGATGGCCTCTA C   CATGGAGC  CCTCTGG  GCAGCTCATTACA  CACAAACTGTCCCAAGGTGAG
                                                               (INR-)              (MTE+)                         
    100 Human: CYP2J2 (1573, AF272142) 
Mouse: Cyp2j6 (13110, AK084025) 
 
           |-50      |-40      |-30      |-20      |-10      |1        |11       |21       |31       |41       
Human     AGGCGGGGCGGGGAC  CGTCGCCT  GCTGGGACCGCCGCCTGCTTGGACCGC A   GAAGAGCA  GGAGGACGTC  TGAGCCATGCTCG  CGGCGATGGGCTCTCTGG
                           (BRE+)                                (INR-)                 (MTE+)                      
Mouse     CCCTTGTTGTTAGCACCTGGGACTTGCCGCAGCTCAGACCTTCATTCC  TC A GACGA  ACCAGCTGCAGTCCTAGC  CATGCTCGCTGCT  ACCGGCTCCTTGT
                                                            (INR+)                         (MTE-)                 
    101 Human: CYP4A11 (1579, AF208532) 
Mouse: Cyp4a10 (13117, AK002528) 
 
           |-50      |-40      |-30      |-20      |-10      |1        |11       |21       |31       |41       
Human     CATTATTGCAATGT  AGTAAAG  CAGAATGCAAATTCCTTGAGGTCA  GCATT C AA  GTCTGTTTTCCTC  GCAGCGGTGCCCA  CACCCCTAGCATACTGCCTGG
                         (TATA+)                           (INR+)                    (MTE+)                         
Mouse     ATTTAGTAAAACTTTGCAAGA  AAAGATT  AAAAATAAGTGGAAGA  ATATTT A A  GGATATTATAATTGTTCTGCTTAATTTATGCATGCAGTACAGGTGTGG
                                (TATA-)                   (INR+)                                                  
    102 Human: DAB1 (1600, AK095513) 
Mouse: Dab1 (13131, AY174225) 
 
           |-50      |-40      |-30      |-20      |-10      |1        |11       |21       |31       |41       
Human     GCTAGGTGGCGAAATGCTGAGGGGCTCCCCTATTCGGGGGAATGCGGTAG T   GATGTATA  AGGTATGAAAGATGTGGCCTGCAGAGAGCCGGCACTATCTC
                                                               (INR-)                                           
Mouse     GTTAGGTGGCGAAATGCTGATGGGCTCTCCTATTCGGGGGAATGCGGTAG T   GATGTATA  AGGTATGAAAGATGTGGCCTGCAGAGAGCCGGCACTATCTC
                                                               (INR-)                                           
    103 Human: DAF (1604, NM_000574) 
Mouse: Daf1 (13136, AK030285) 
 
           |-50      |-40      |-30      |-20      |-10      |1        |11       |21       |31       |41       
Human     TGTCCCACCCTTGGTGACGCAGAGCCCCAGCCCAGACCCCGCCCAAAGCA C   TCATTTAA  CTGGTATTGC  GGAGCCACGAGGC  TTCTGCTTACTGCAACTC
                                                               (INR+)                 (MTE+)                      
Mouse     GGCTGCCCCACCCAGGGTGACGAGGGCCCTGCCCCGCCCCGCCACAGCTG C   TCAATTAA  CTGCGGCTCAAA  ACAGCTCCAGCCG  CAGCCGGGCAAGGTCT
                                                               (INR+)                   (MTE+)                    
    104 Human: DBT (1629, NM_001918) 
Mouse: Dbt (13171, AK075831) 
 
           |-50      |-40      |-30      |-20      |-10      |1        |11       |21       |31       |41       
Human     CTGATAGG  CCGCGCCG  AGGAGAGTCTCTCCAGTTTGCGGCACTGTTG  TCA T TTCC  GGGGTAAGATGGCT  GCAGTCCGTATGC  TGAGAACCTGGAGCAGGA
                    (BRE+)                                   (INR+)                     (MTE+)                      
Mouse     GGATTGGTCGCCTGGAGGAGGCGTCTTTCCCAGCTGTGTGCTGGTGG  CTA T TTCC  GGAGTAAAATGGCT  GCAGCGCGTGTAC  TGAGGACCTGGAGCCAGA
                                                           (INR+)                     (MTE+)                      
    105 Human: DDOST (1650, D29643) 
Mouse: Ddost (13200, D89063) 
 
           |-50      |-40      |-30      |-20      |-10      |1        |11       |21       |31       |41       
Human     GCCTTATCGCCAAAGCTGCGGCTCTGGACGCCCAGCCGCGGCGTATCCCG A   TCACTTCC  GGGTAGTGCTCCACGGGCACGAGCCGCGATTGGGCTACCGT
                                                               (INR+)                                           
Mouse     CTTGGTCCGTCCCCGCAGCGGCGTGGGCCGCGGGGCCACGGAGTACCCGG A   CCGCTTCC  AAGTGGTA  CTCCGCCGCTGCG  AGCGGCCATTGGGCGGCCGC
                                                               (INR+)               (MTE-)                        
    106 Human: DDOST (1650, D29643) 
Mouse: Ddost (13200, D89063) 
 
           |-50      |-40      |-30      |-20      |-10      |1        |11       |21       |31       |41       
Human     TAGGGTTCAAGGAATCCACCTCCCACCAGGGCACTTCCGGCGGCGCTCTC C GCGCCTTATCGCCAA  AGCTGCGGCTCTG  GACGCCCAGCCGCGGCGTATC
                                                                                 (MTE-)                         
Mouse     ATAAGGTACAGAGACTCGGCCTCCGGAGGGCCACTTCCGGTGGCGTTCCC T G  CTTCTTGG  TCCGTCCCC  GCAGCGGCGTGGG  CCGCGGGGCCACGGAGTA
                                                                (INR+)                (MTE+)                      
    107 Human: DHX9 (1660, NM_001357) 
Mouse: Dhx9 (13211, NM_007842) 
 
           |-50      |-40      |-30      |-20      |-10      |1        |11       |21       |31       |41       
Human     TCTGCGCATGCGTACGCTCGCTGGCCCCGCCCCCTAGCGCCGCGGTCGGA G   CCATTTCG  CCGATTCCTCCATGCG  AGTTGC  TGTGCGTTTCTCTGTTGTC
                                                               (INR+)                   (DPE+)                    
Mouse     ACCGCGCA  GGCGCGAG  TCGCCGACTCCGCCCCCTGGGTCCTGCAGTCGGA G   CCATTTCG  CCGCTGCTT  CCATGCGGGTGAG  GTGTGTTCTTTCGCCGTTC
                    (BRE-)                                       (INR+)                (MTE-)                       
    108 Human: DFFA (1676, NM_004401) 
Mouse: Dffa (13347, AK039227) 
 
           |-50      |-40      |-30      |-20      |-10      |1        |11       |21       |31       |41       
Human     CTACATTTCCC  GGCGTGCC  CTGGTCTCACGTGTGATTTGCTGCGGAAC  GA A CTACA  TCTCCCGGCAGGCTGC  GGAAGGGGGTCGA  GTAGAAGGACCGCCG
                       (BRE-)                                 (INR-)                       (MTE-)                   
Mouse     TAGAACTACATCTCCC  GGCGTGCC  TTGGTCTCGCGTAACTTGCTGAGA  GA A CTACA  TCTCCCGTCAG  GCAGCGGGAGGAA    GGTTGT  CAAAGCTTAGGGAA
                            (BRE-)                            (INR-)                  (MTE+)     (DPE+)               
    109 Human: DHCR24 (1718, AF261758) 
Mouse: Dhcr24 (74754, AK129036) 
 
           |-50      |-40      |-30      |-20      |-10      |1        |11       |21       |31       |41       
Human     GCGAGGCGGCGGGCGATCCCGGGCTCCCCGGGCTGTGGGCTACAGGCGCA G AGCGGGCCAGGCGC  GGAGCTGGCGGCA  GTGACAGGAGGCGCGAACCCGC
                                                                                (MTE+)                          
Mouse     GAGGCGGCGGGCCCGACTTACAGGCTCCCGGGGCGGTGGCGGCAGGCGCT G AGCCGGCGGAGCGC  GGAGCGGGCGAAG  G  AGACGG  GCGGCGCGAGCCCGC
                                                                                (MTE+)      (DPE+)                
    110 Human: DHCR24 (1718, NM_014762) 
Mouse: Dhcr24 (74754, AK129036) 
 
           |-50      |-40      |-30      |-20      |-10      |1        |11       |21       |31       |41       
Human     CATTGGTCGCCGCCCGGGTCTCGGCCCACCGAACCTCGGCGACCCGAG  CC A ATCGC  GAGGCGGCGGGCG  ATCCCGGGCTCCC  CGGGCTGTGGGCTACAGG
                                                            (INR+)                    (MTE-)                      
Mouse     CATTGGTCGCCGCCTGGGTCTCGGCCCACCGAACCTTGGCGACCCAAG  CC A ATCGC  GAGGCGGCGGGCCCGA  CTTACAGGCTCCC  GGGGCGGTGGCGGCA
                                                            (INR+)                       (MTE-)                   
    111 Human: DIO1 (1733, NM_000792) 
Mouse: Dio1 (13370, NM_007860) 
 
           |-50      |-40      |-30      |-20      |-10      |1        |11       |21       |31       |41       
Human     TTCAGCTCCGCCCAGTGTTGCCTGGCTGCAGCCTCTCTGCCCATA  GAACT C AG  AGCTTACTCTGGCTTTGCCG  AGATGG  GGCTGCCCCAGCCAGGGCTGT
                                                         (INR-)                       (DPE+)                      
Mouse     AGCTCCACCCAGTGCTGTCTGGCTGGGGCCTCTCCTTCAGTCTGCAGGC  T C AGAGCC  ACTTCTGCCCCGTGCTG  AGATGG  GGCTGCCCCAGCTATGGCTG
                                                             (INR+)                    (DPE+)                     
    112 Human: DPH2L2 (1802, CR600358) 
Mouse: Dph2l2 (67728, BC014746) 
 
           |-50      |-40      |-30      |-20      |-10      |1        |11       |21       |31       |41       
Human     TAAAACCCGGAAGTAACCTCCCCGGTAGTCCCACGTGTAGCGGAGAAACA   G TAGTTAG  GATGGC  TGAAGGGGATACT  CACCGGCTGAAGGCCGACTGTGA
                                                              (INR+)             (MTE-)                           
Mouse     TCCTTGAACCCGGAAGTAATCTCCGTAGGTCCATGTGTAGTGGAGAGCCG G G  AATTTGGA  AGCGTACGGTTTG  GATCCT  AGGACCCGAGGACAGGTTGGT
                                                                (INR-)                (DPE-)                      
    113 Human: DPT (1805, NM_001937) 
Mouse: Dpt (56429, AK019890) 
 
           |-50      |-40      |-30      |-20      |-10      |1        |11       |21       |31       |41       
Human     GAAGCCACATGTCAGCGGCAGCTA  TATAAAA  CGGTCAGACCAGCAATCTT   A GAGTGAC  ATTGTTTGCCA  AAATCCCAGGCAG  CATGGACCTCAGTCTTCT
                                   (TATA+)                      (INR-)                  (MTE+)                      
Mouse     CTGGGAACCACATGTCAGCGGC  AGCTGTA  TAAAGTCAGCCCAGC  AAACTG A G  GGTGACATTTTTTTTGCCA  GAAGCCGGGCAGT  ATGGACCTCACTCTTC
                                 (TATA-)                  (INR-)                          (MTE+)                    
    114 Human: DR1 (1810, BC035507) 
Mouse: Dr1 (13486, AK075713) 
 
           |-50      |-40      |-30      |-20      |-10      |1        |11       |21       |31       |41       
Human     TCGGTCCGGGAGGGAGGGGGAAATCTGAGGGAACGAGGCTTCCGGTGGCG C   AAAGGGTG  TCGGGAGCGGCTTCCTGCAAACCTTCCCTGGCATCTGGAGG
                                                               (INR-)                                           
Mouse     ATCCATCGAGGAGGGCGGGTGGGACCTGGGGAGTTCGACTTCCGGTGGCG C   AAAGCGTG  CCGGGATCAGCTTGCGGCAAACCTCCTTCCCCGGCCCGTAG
                                                               (INR-)                                           
    115 Human: DR1 (1810, M97388) 
Mouse: Dr1 (13486, AK075713) 
 
           |-50      |-40      |-30      |-20      |-10      |1        |11       |21       |31       |41       
Human     CTGCGTTCGGGCTACGCGGCCACGGCGGCAGCCACTGCGACTC  CCACTGT G   CCTGGCTCTGTCCATATTAGTTCCCAGGCGGCCGTCGCCGTTCCAGCAG
                                                       (INR+)                                                   
Mouse     TCGCACAGCCTGCTCTCGGCCACACGCTTGGCCGCTGCGGCTCCC  GCAGT G TT  CGGCCCGGTCCACTTGGTTTCCCAGGCAGCCGGCGTGCGCTCAGCTG
                                                         (INR-)                                                 
    116 Human: DVL1 (1855, NM_182779) 
Mouse: Dvl1 (13542, NM_010091) 
 
           |-50      |-40      |-30      |-20      |-10      |1        |11       |21       |31       |41       
Human     CCCTGAGCGCGCGGGCT  CCGCGCCG  CCGCCGCGCCATGGCGGAGACCA  AG A TTATC  TACCACATGGACGAGGAGG  AGACGC  CGTACCTGGTCAAGCTGCC
                             (BRE+)                           (INR-)                      (DPE+)                    
Mouse     ACGCCGCGCGGGTT  CCGCGTCG  CCCCTGCCGCGCCATGGCGGAGACCAAA A T  CATCTACC  ACATGGACGAGGAGG  AGACGC  CGTACCTGGTCAAGCTGCC
                          (BRE+)                                  (INR-)                  (DPE+)                    
    117 Human: ECE1 (1889, AF055469) 
Mouse: Ece1 (230857, NM_199307) 
 
           |-50      |-40      |-30      |-20      |-10      |1        |11       |21       |31       |41       
Human     GGTCGGGCTCGCGGGTGGCGGCGATTTCGAGGTGCCTGGATGCAGCTGGA C CTGAGCGTCCCGGGACACTGAGGTC  GGTCTG  GGGCTCGCCTTGGGGCAC
                                                                                       (DPE-)                   
Mouse     CCGTGGGCAGCTAGGCCAGGTCGATTCAGAGACGTCAAGGTTCAGCTG  GA C TTGAG  CTCACACCCCGCATAGT  CCGAGTGGCTTTG  GGATTCGAGAACAT
                                                            (INR-)                        (MTE-)                  
    118 Human: ECE1 (1889, NM_001397) 
Mouse: Ece1 (230857, NM_199307) 
 
           |-50      |-40      |-30      |-20      |-10      |1        |11       |21       |31       |41       
Human     GCCCCGCCCCTCCGGCACTCGGCGCCGAAGCCGCGAGCTCGCCCGCTG  GA G CTGAG  CGCGCCGCCTGGGCCAG  GCAGCCGAGCCGT  CCGAGCAGCTGGGC
                                                            (INR-)                        (MTE+)                  
Mouse     GCACTTCTGTATTGGTTCGCGGCGGGCCCCGCCCTCTGGCCTGGGCGCC  C C AGCCTC  GCGCGCCGCCCCGGCAG  CTAGCCCTGAGGT  TCGAGCGTAGAGC
                                                             (INR+)                        (MTE+)                 
    119 Human: ECM1 (1893, AK097205) 
Mouse: Ecm1 (13601, BI739978) 
 
           |-50      |-40      |-30      |-20      |-10      |1        |11       |21       |31       |41       
Human     AGGAGGAGCAGCTGGGACTGAGTCATGGCAGGAAGCTGAGGAGGGCGGGA G A  TCACACCA  GACAATTATAAAAGAAGAGCT  GGTCCT  GAAGCTCACAACC
                                                                (INR+)                        (DPE-)              
Mouse     AGAGGAGGAGCAGCAGGGACTGAGTCATGGCAGGAAACAGGGGGGCGG  GA G ATAGC  ATATAAAGAGAGACAG  ACAGCAGGCCTTG  AAGTTCACAACGGCA
                                                            (INR-)                       (MTE+)                   
    120 Human: ECM1 (1893, U65933) 
Mouse: Ecm1 (13601, BI739978) 
 
           |-50      |-40      |-30      |-20      |-10      |1        |11       |21       |31       |41       
Human     TTGCCTGAGAGGACCCACCTCTGAGTGTCCAGTGGTCAGTTGCCCCAGGA T GGGGACCACAGCCAGAGCAGCCTT  GGTCTT  GACCTATTTGGCTGTTGCT
                                                                                      (DPE-)                    
Mouse     CCTGTGACAACCAGCTTCTGGGTGACCAGTGACCAGTTCTTGCCCCAGGA T GGGGACCGTATCCAGAGCAGCCTT  GATCTT  GGCCTGCTTGGCTCTTGCT
                                                                                      (DPE-)                    
    121 Human: EDG1 (1901, NM_001400) 
Mouse: Edg1 (13609, AK004591) 
 
           |-50      |-40      |-30      |-20      |-10      |1        |11       |21       |31       |41       
Human     GAGGAGGGGTTCTT  TTTTTTT  TTTTTTTTTTTTTTTTTGCTTCTGCC  CCA G ATCT  TTCCTGGACAGTGCGTCTCAGCAGTTC  AGATCC  GGGGGCCCCCAG
                         (TATA-)                             (INR+)                              (DPE+)             
Mouse     GGGCGGAGGGGCTCCTATTCAT  TTGGTTT  TATTTTAGTCACTCTGCC  CCA G ATCT  TTCTGGGAGGCTGCTTCTTAGCAGCCC  AGACCC  TGAGGGGCCCCC
                                 (TATA-)                     (INR+)                              (DPE+)             
    122 Human: EDG1 (1901, NM_001400) 
Mouse: Edg1 (13609, AK004591) 
 
           |-50      |-40      |-30      |-20      |-10      |1        |11       |21       |31       |41       
Human     TTCCTGAAGAAACCACCCAGCCTTGGCGCGGCGCTGGGTGACTTCGCGTA G CAGGCAGGGAACTGGCCGCGG  CGAGCGGGACTGG  CCATTGGAGTGCTCC
                                                                                       (MTE+)                   
Mouse     TTCCTAGAGGAGCCACCCAGCCTCGGCGGGGCGCTCAGAGACTTCGTCTT G CA  AAAGTGGT  GCGG  GCAGCGCAGGCAG  GGCTGGCCATTGGAGTGCACCG
                                                                 (INR-)           (MTE+)                          
    123 Human: PHC2 (1912, AJ419231) 
Mouse: Phc2 (54383, AI227386) 
 
           |-50      |-40      |-30      |-20      |-10      |1        |11       |21       |31       |41       
Human     CTCTAGTAAGCGTCTGTCAAAACATTCACTTCTGCCTCTTCCCTCCTAG  C A TCTGCC  TGCCCTTCTGCCATC  CGAGCGCCCTGAC  TGCGCCACACTGCAG
                                                             (INR-)                      (MTE+)                   
Mouse     TTCCACAAACATTTGCTAAGCT  TTTCATT  TCTTTTCTCTTCCTTCCTAGC A T  CCACCTGC  TCTTCTG  CCACCTGAGCACC  C  TGACTG  TACCACCCCATAG
                                 (TATA-)                          (INR+)              (MTE+)      (DPE-)              
    124 Human: LGTN (1939, NM_006893) 
Mouse: Lgtn (16865, NM_010709) 
 
           |-50      |-40      |-30      |-20      |-10      |1        |11       |21       |31       |41       
Human     CCCAGCGGCAACGGCCACGAAGCTGCGCGGCCCTGGTTTCCAGCCGGGC  C C TTTTCG  CGGCCGGGCCC  CAGCATGGCTGCC  CCCACGGCTGAGGGCCTGG
                                                             (INR+)                  (MTE-)                       
Mouse     CGTCAGTTCCGGGCCAGCATCAGGGCTAGGGCCGGGTTCCTAGCAGGCTG T   TTTTTGGC  GCCGAGTCT  CAGCATGGCTGCC  TCCAGGGTTGAGGGTCCGG
                                                               (INR-)                (MTE-)                       
    125 Human: EFNA1 (1942, AK057845) 
Mouse: Efna1 (13636, AK009144) 
 
           |-50      |-40      |-30      |-20      |-10      |1        |11       |21       |31       |41       
Human     GCCGCGCGCCGGGCGG  GGCGAGGG  CGGGGCGGGGTGTCGGGCGGCGCCGG C C  CAAAAGGC  GGAGTCGCTAGGCGAAGGGGCC  AGATCT  GTGAGCCCAGCG
                            (BRE-)                                (INR-)                         (DPE+)             
Mouse     GCAGCGCGCGTCGGGCGG  GGCGAGGG  CGGGGCTGGTGTTGGCGGCGCCAG C C  CAAAAGGC  GGAGTCGA  GCAGCGAAGAGGC  CAGATTTGTGATCGTGGTG
                              (BRE-)                              (INR-)               (MTE+)                       
    126 Human: EPHA2 (1969, NM_004431) 
Mouse: Epha2 (13836, CF164571) 
 
           |-50      |-40      |-30      |-20      |-10      |1        |11       |21       |31       |41       
Human     ACGTCACGCAGGGCATG  AATGAAC  AGGAGTCGGTTCTCACCCAACTT  CCA T TAAG  GACTCGGGGCAGGAGGGGCAGA  AGTTGC  GCGCAGGCCGGCGGGCG
                            (TATA+)                          (INR+)                         (DPE+)                  
Mouse     CGTCAGCGCAGGGCATG  AATGAAC  AGGAGCGGGTTCTCACCCAACTT  CCA T TAAG  GACTTGGAGCAGGAGGGTAGA  AGTTGT  CTCTGTCGGCGGGCGGGC
                            (TATA+)                          (INR+)                        (DPE+)                   
    127 Human: ELF3 (1999, NM_004433) 
Mouse: Elf3 (13710, AY456682) 
 
           |-50      |-40      |-30      |-20      |-10      |1        |11       |21       |31       |41       
Human     GTTACTGCAGGGGACACAC  TATAAAG  CCCTGAGCTCAGGGAGGAGCTCCC T CCAGGCTCTATTT  AGAGCCGGGTAGG  GGAGCGCAGCGGCCAGATACCTC
                              (TATA+)                                            (MTE+)                           
Mouse     CAGGTTACAACCGGGGAC  GTACGCCG  AAGACCTGGAGGGGAGGAGCTCCT G CTTTGCTCTATTT  AGAGCGGGTGGGG  GCAGCGCCCTGGCCACACTCATC
                              (BRE+)                                             (MTE+)                           
    128 Human: ELK4 (2005, NM_021795) 
Mouse: Elk4 (13714, NM_007923) 
 
           |-50      |-40      |-30      |-20      |-10      |1        |11       |21       |31       |41       
Human     CTGCCGCGTGCGGG  GGCGGGGA  GGGCGGGGCGCCAGGAGCCGCGGCGGCG G   GAGATGCG  GGCGGCTGCGGGCAC  CCGGCGGGCTCGG  CTTGGCCGCCGCC
                          (BRE-)                                 (INR-)                      (MTE-)                 
Mouse     CGCCGTCCCGGCCGCGTG  TGCGAGGG  CGGGGCTCGAGACCTGCGGCAGCG G   GAGATGCG  GGCGGCTGCGG  GCACGCGGCTGGC  TCGGCTCGGCCGCCGCT
                              (BRE-)                             (INR-)                  (MTE-)                     
    129 Human: ENO1 (2023, M14328) 
Mouse: Eno1 (13806, BC085098) 
 
           |-50      |-40      |-30      |-20      |-10      |1        |11       |21       |31       |41       
Human     GAGCTGAGGG  GGCGTGCC  CCGGAGGCGGGAAGTGGGTGGGGCTCGCCTTA G CTAGGCAGGAAGTCGGCGCGG  GCGGCGCGGACAG  TATCTGTGGGTACCC
                      (BRE-)                                                             (MTE+)                   
Mouse     CGCGGAGGGAG  GGCGGGCC  CGAAAGGGGAGAACTGGGCGGGGCTTAACGA G CAGGAAAGGAAGACAGAGT  GGGAGGCGCTTAG  TGCTGCTCCGGTACAGG
                       (BRE-)                                                          (MTE-)                     
    130 Human: ENSA (2029, NM_004436) 
Mouse: Ensa (56205, AK019170) 
 
           |-50      |-40      |-30      |-20      |-10      |1        |11       |21       |31       |41       
Human     ATCTACCTCACTCCCAC  TGGCTCT  CATAGGAAGGGGCGGTCGCAACGTCA C GGGCAAGGGCCGCCATTTTGAC  TGAGCAACCCTAG  TGACAGGAGCCGAA
                            (TATA-)                                                       (MTE+)                  
Mouse     GTCAAGCCCACTCTCAT  TGGCTCT  CATAGGAGGGGGCGGTCTCAACGTCA C GGGCTTGAGCCGCCATTTTGAC  TGAGCAACCATAG  TGACAGGAGCCGGA
                            (TATA-)                                                       (MTE+)                  
    131 Human: EPB41 (2035, NM_004437) 
Mouse: Epb4.1 (269587, BC068138) 
 
           |-50      |-40      |-30      |-20      |-10      |1        |11       |21       |31       |41       
Human     CAGAGGGCCTAGCG  GGCGGGGC  GGGGCCTCACGATGGAGTTGCCCTGTCA G TG  CAGGTGGA  GGCC  CCGGCGGGGCAAA  GTGGCAGGAACCTCTTAAAGGG
                          (BRE-)                                   (INR-)           (MTE+)                          
Mouse     CAGGACCTCCCGA  GGGGAGGG  CGGGGCCTCGCATCAGAGTTGCCCTG  TCA G TGCA  GGTGGAGGCCC  CCGGCGGGGCAAA  GTGGCAGGAACCTCTTAAAGG
                         (BRE-)                              (INR-)                  (MTE+)                         
    132 Human: EPHB2 (2048, L41939) 
Mouse: Ephb2 (13844, BB869147) 
 
           |-50      |-40      |-30      |-20      |-10      |1        |11       |21       |31       |41       
Human     GCTCCCGC  CCGGGCCG  TCCGGGCCCCGCGGCGCCGCGGCCCGAGGCCCCG G   GAAGCGCA  GCCATGGCTC  TGCGGAGGCTGGG  GGCCGCGCTGCTGCTGCT
                    (BRE+)                                       (INR-)                 (MTE-)                      
Mouse     GCGCGCGCTCCAGCCCG  GGGGTCCC  CGCGGCGCCGCGGCCGGAGGCTCCG G GCAGCGCAGCAATGGCCG  TGCGCAGGCTGGG  GGCCGCGCTGCTGCTGCT
                             (BRE-)                                                   (MTE-)                      
    133 Human: EPRS (2058, X72396) 
Mouse: Eprs (107508, X54327) 
 
           |-50      |-40      |-30      |-20      |-10      |1        |11       |21       |31       |41       
Human     TTATCTCGTGCTTATATTC  TAGAAAT  GTGATATTCACAGATGTGAATTCT   A TACTTCG  CTACTTGGCTAGAGTTG  CAACTA  CAGCTGGGTTATATGGCTC
                              (TATA+)                           (INR+)                    (DPE-)                    
Mouse     TTACATAATGCTTCAATTCTAGGACTGTGGCATTCACTGACGTGAAT  TCA A TCCT  GCGCTACCTGGCTAGAATTGCAACTA  CGTCTG  GGCTGTATGGGAC
                                                           (INR+)                             (DPE-)              
    134 Human: EPRS (2058, NM_004446) 
Mouse: Eprs (107508, AK049657) 
 
           |-50      |-40      |-30      |-20      |-10      |1        |11       |21       |31       |41       
Human     TCGCACCGC  GGCGTGCG  CGGTGTCGGCAGTAGCTGCGGCGCAGGGGCGGA G CGAAGGCTGCGGC  GGCGTCGGGTACG  CGCACACGTTGCATCTTCTTCCT
                     (BRE-)                                                      (MTE-)                           
Mouse     CGTGTCGTAGCGT  GCGCACCG  ACTCCGGCGGTGGCGGCCCGTGGGGA  GGA G TCAG  GGCTGCGGTGCCTT  CGGTCGCGCGCAC  ACGTTGCATCTCCTTGCT
                         (BRE+)                              (INR-)                     (MTE-)                      
    135 Human: EPS15 (2060, NM_001981) 
Mouse: Eps15 (13858, NM_007943) 
 
           |-50      |-40      |-30      |-20      |-10      |1        |11       |21       |31       |41       
Human     CGCCTCCTCCCCGCC  CCGAGCCC  CAGTCAGCCCGTCTTCCTTCCCC  TCCC T TGC  ATGATGGAAA  CACCATGGCTGCG  GCGGCCCAGCTCTCTCTGACACA
                           (BRE+)                           (INR+)                 (MTE-)                           
Mouse     GCCTCCTTC  CCGCCCCG  GGCCCCCGTCCGTCCGTCCTTCCTTCCCCTCCC G   TGCATGAT  GGAAA  CACCATGGCTGCG  GCAGCCCAGCTCTCCCTGACACA
                     (BRE+)                                      (INR-)            (MTE-)                           
    136 Human: EXTL1 (2134, NM_004455) 
Mouse: Extl1 (56219, BY018364) 
 
           |-50      |-40      |-30      |-20      |-10      |1        |11       |21       |31       |41       
Human     GGGAGGGGAGGAGCCAGGCTGCTGCTAAGGAAAGAGATCAGGATGGG  CCA G TTGC  CGACTGGAGAGGGATGGGAGG  AGATAC  CATAGGGTTTGGGGGGCT
                                                           (INR+)                        (DPE+)                   
Mouse     CGGGGGTGTGGCCAGGCTGCTGCTGCTGCGAAAGAGAGCTGGAC  CGACTT G A  TACCTTCTGGAGTGGG  ACCTGGAGCTGCC  GGCTGGACCTGTGGGGCAG
                                                        (INR+)                       (MTE-)                       
    137 Human: EXTL1 (2134, AF083623) 
Mouse: Extl1 (56219, BY018364) 
 
           |-50      |-40      |-30      |-20      |-10      |1        |11       |21       |31       |41       
Human     TGCTGGCAGAGGCCTCCCAGCTTCCCTAGCCCTGACTGTGGGTGG  CCACA T GC  AGTCGTGGAGGAGAAGAAAGT  CCCTGTGGCTGGC  ACTGTCAGCCTCC
                                                         (INR+)                            (MTE-)                 
Mouse     TGCTACAGAAATTCCTC  ACTTCCT  TCTAGTCCTAGCTGTTGGTGG  CCACA T GC  TGTGGAGAAGAAAGT  CCTTCTGGCTGGC  GTTGTCAGCCTTCTGGCTC
                            (TATA-)                        (INR+)                      (MTE-)                       
    138 Human: EXTL2 (2135, NM_001439) 
Mouse: Extl2 (58193, AF200973) 
 
           |-50      |-40      |-30      |-20      |-10      |1        |11       |21       |31       |41       
Human     GCCTTTTAGGCCGACAGGAAGTGGAGCGTCACCGAAAGGGAGGGGCGG  CC A CTCGG  GGACTGTCCCTTGC  TCCAGGCGCTCAC  TTTGCGGGCGGCACTTT
                                                            (INR+)                     (MTE-)                     
Mouse     TTTTGCCTAACAGGAAGTGGAGCGTCACCCAAAGCGAGGGAGGGGTGG  CC A CTCAG  GAACTGTCCCCTGC  TCCAGGCACTCAC  TTTGCAGGCGGCGCATT
                                                            (INR+)                     (MTE-)                     
    139 Human: EYA3 (2140, NM_172098) 
Mouse: Eya3 (14050, NM_210071) 
 
           |-50      |-40      |-30      |-20      |-10      |1        |11       |21       |31       |41       
Human     GAGGTGTGGATTGTGAGGTGACTCTGGCCGCTTACTTCCGGTTCCTAGCG A TGCGCATCCGGGTCACG  CTAACGCCGCGGT  TTCCTCCGCTCGATTGGTT
                                                                                   (MTE+)                       
Mouse     ACGGGGTGTGGATTGTTAGGCGGTCCCGCATCTACTTCCGGTGGCTAGGG A TGCGCATCCGGGTCACG  CTAACGCCGCGGT  TTCCGCCACTCTGTTGGTT
                                                                                   (MTE+)                       
    140 Human: F3 (2152, NM_001993) 
Mouse: F3 (14066, BC024886) 
 
           |-50      |-40      |-30      |-20      |-10      |1        |11       |21       |31       |41       
Human     GCGGGCGCCG  GGGGCGGG  CAGAGGCGCGGGAGAGCGCGCCGCCG  GCCCTT T A  TAGCGCGCGGGGCACCGGCTCCCCAAGACTGCGAGCTCCCCGCACCCC
                      (BRE-)                              (INR+)                                                  
Mouse     TCGGCGGAGGC  GGCGCGGG  GTGGAGAGGAGCCGGTGTCCGCGCG  GCCCTT T A  TAACGCACCCCGCGC  CGACCCCGGCAGC  CTGGGTACAGCCGGTACCCA
                       (BRE-)                             (INR+)                      (MTE+)                        
    141 Human: F5 (2153, M14335) 
Mouse: F5 (14067, NM_007976) 
 
           |-50      |-40      |-30      |-20      |-10      |1        |11       |21       |31       |41       
Human     TAGTTTGGTTGCTCTCCCTAATACCTCTGGTCACTGGGAGCTGTGATCTC A   CCAAACCC  CTGCCAGGA  AAAGCCCCAGAAA  AAGCGGAGGGAGTGAGAGC
                                                               (INR+)                (MTE+)                       
Mouse     TCTGGCATTCTCTCT  AGTAAAT  GCCTGTGGCCACAGGGGGGAGTGACCT  C A ACAAGC  CCCTGCCTACACTAGGC  ATAGAGGGAGGTA  GGGTTAGACAGCC
                          (TATA+)                              (INR-)                        (MTE+)                 
    142 Human: FAAH (2166, AF098010) 
Mouse: Faah (14073, BC052321) 
 
           |-50      |-40      |-30      |-20      |-10      |1        |11       |21       |31       |41       
Human     GGTTTTGCGGC  GGAGCGGG  CGGGCTGCGCGTGCGGCGGCTTCAACTGTCG C GGTAGGCAGCAGCAGG  CTGAAGGGATCAT  GGTGCAGTACGAGCTGTGGG
                       (BRE-)                                                       (MTE-)                        
Mouse     GGTTTTGCAGCGGAGCTGTTGGTGTGCGCGTGCCGAGTCCTCTCGGGTGG C GGTCGGCTGCAGGAGA  TCATGGTGCTGAG  CGAAGTGTGGACCGCGCTGT
                                                                                  (MTE-)                        
    143 Human: FABP3 (2170, NM_004102) 
Mouse: Fabp3 (14077, NM_010174) 
 
           |-50      |-40      |-30      |-20      |-10      |1        |11       |21       |31       |41       
Human     TTCGGGAGCCGGG  GGCGTGGG  CCACGTCTCCTCATGTGATGCGAGGG  CTA T TTAA  AGCGGCAGCCCGGGCAG  GGAGCCGCCGTCG  GAGCCCTTGCACGCC
                         (BRE-)                              (INR+)                        (MTE+)                   
Mouse     TTTCGGGAGCGAGG  GGTGTGGG  CCACTTTCATCATGTGATGCGAGGG  CTA T TTAA  AGAGGCTGT  CCAGCCGGGAGCT  GCGGTTCTCAGTGCCTGCTCGCC
                          (BRE-)                             (INR+)                (MTE+)                           
    144 Human: FCER1A (2205, AB059236) 
Mouse: Fcer1a (14125, J05018) 
 
           |-50      |-40      |-30      |-20      |-10      |1        |11       |21       |31       |41       
Human     CTTCTGACCTTGGCAATAGGAAGGAGAAGGAAGCACTTTCCAG  ATAGTGA T   CTTTAAAAGGCATGAAAGGACTGAGTCTACTCAGAGAAAGCAGGTGAAA
                                                       (INR-)                                                   
Mouse     GTGTCTGACCTTGGCAGTGGGAACCGGAAGGAGGTGCATCCTGGAA  GAAA T CTT  TACAAAGTTCTGGAAAGAACTGAGTCTGGAAACAAACAGATCAACA
                                                          (INR-)                                                
    145 Human: FCER1A (2205, L14075) 
Mouse: Fcer1a (14125, J05018) 
 
           |-50      |-40      |-30      |-20      |-10      |1        |11       |21       |31       |41       
Human     TCTGCTTTTTGGTTTT  AAGCCTA  TATTTGAAGCCTTAGATCTCTCCAGC  A C AGTAAG  CACCAGGAGTCCATGAAGA  AGATGG  CTCCTGCCATGGAATCCC
                           (TATA-)                             (INR-)                      (DPE+)                   
Mouse     CTTCTCCTTTGTGG  TTTCAAG  CCTATTTTCGAAGCCATAGCTCTCTGGT  G C AGTTAG  CACCTGAAGGTGCAGGGGCGAT  GGTCAC  TGGAAGGTCTGCCCA
                         (TATA+)                               (INR+)                         (DPE+)                
    146 Human: FCER1G (2207, NM_004106) 
Mouse: Fcer1g (14127, BC034163) 
 
           |-50      |-40      |-30      |-20      |-10      |1        |11       |21       |31       |41       
Human     TGTGCAGGAAGGGGAAGGGGCCAAAGCATGGGGGAAGGCGTGGCAG  GAAG A GGG  GGACTCTGTGGTCAGGGA  ACTGCTCGCTGAG  CACAGCTGCACAGTG
                                                          (INR-)                         (MTE-)                   
Mouse     AGTACAGGAAGGGGAAGGGGCCAAAGTGTGGGCGAAGGCGTGGCAG  GAAG G AGG  GAACTGTGGTCAG  GGAACTGTTCGTG  GGCACAGCTGCGCAGTTCTG
                                                          (INR-)                    (MTE+)                        
    147 Human: FCER1G (2207, M33196) 
Mouse: Fcer1g (14127, BC034163) 
 
           |-50      |-40      |-30      |-20      |-10      |1        |11       |21       |31       |41       
Human     GACTCTGTGGTCAGGGAACTGCTCGCTGAGCACAGCTGCACAGTGCTGTC A GAACGGCCGATCTCCAGCCCA  AGATGA  TTCCAGCAGTGGTCTTGCTCTT
                                                                                   (DPE+)                       
Mouse     GGGAACTGTGGTCAGGGAACTGTTCGTGGGCACAGCTGCGCAGTTCTGTC A GCGCAGCGCGATCACCAGCTC  CCAGCGCCGCAGC  CCCCAGCGCACCCAG
                                                                                       (MTE+)                   
    148 Human: FCGR1A (2209, CR591922) 
Mouse: Fcgr1 (14129, M61171) 
 
           |-50      |-40      |-30      |-20      |-10      |1        |11       |21       |31       |41       
Human     GGAGAGATGGGCTAACAGG  TATGAGC  ATGGGAAAAGCATGTTTCAAG  AAT T TGAG  ATGTATTTCCCAGA  AAAGCAACATGAT  GAAAATGGTCAGAAAAGG
                              (TATA+)                        (INR-)                     (MTE+)                      
Mouse     TCTGAGGATAACCTGGGCTACAGGAGACCTGTCAACAGAGGGGAAA  AAAC T AAG  GATGTATTTCCCAGAAAAG  GGAACAGTACCAT  AATGCAAGCAGGCA
                                                          (INR-)                          (MTE+)                  
    149 Human: FCGR1A (2209, X14355) 
Mouse: Fcgr1 (14129, M61171) 
 
           |-50      |-40      |-30      |-20      |-10      |1        |11       |21       |31       |41       
Human     TATTTCCCAGAAAAGCAACATGATGAAAATGGTCAGAAAAGGCAATTTCC T T  CCTCTTTT  CTAATTTGGCTCT  GGAGCCACCAGCA  GAACCTCTTCAATA
                                                                (INR+)                    (MTE+)                  
Mouse     CAGAAAAGGGAACAGTAC  CATAATG  CAAGCAGGCAGAAATGGCAACTTCC T T  CCTCTTTT  CTAATCTTGGTCC  CCAGCCCTGAGTA  GAACCTTCCAGTGT
                             (TATA+)                              (INR+)                    (MTE+)                  
    150 Human: FCGR1A (2209, X14355) 
Mouse: Fcgr1 (14129, M61171) 
 
           |-50      |-40      |-30      |-20      |-10      |1        |11       |21       |31       |41       
Human     TTTGGCTCTGGAGCCACCAGCAGAACCTCTTCAATATCTTGCATGTT  ACA G ATTT  CACTGCTCCCACCAGCTTGG  AGACAA  CATGTGGTTCTTGACAACT
                                                           (INR+)                       (DPE+)                    
Mouse     ATCTTGGTCCCCAGCCCTGAGTAGAACCTTCCAGTGTCTTGCACA  TTACA T GA  TTCTTACCAGCTTTGGAGATG  ACATGTGGCTTCT  AACAACTCTGCTA
                                                         (INR+)                            (MTE-)                 
    151 Human: FCGR2B (2213, CR592864) 
Mouse: Fcgr2b (14130, AK089266) 
 
           |-50      |-40      |-30      |-20      |-10      |1        |11       |21       |31       |41       
Human     GCCTGTGATAAAACA  GAACATT  TCTTTTTCACTTCCCCTTTCAGACTCCA G   AATTTGTT  TGCCCTCTAGGGTA  GAATCCGCCAAGC  TTTGAGAGAAGGCT
                          (TATA-)                                (INR-)                     (MTE+)                  
Mouse     AGCCCAGGATTAAACA  GACCGTT  TCTTTTCACTTCCCCATTTGGACTTC  A C AATTTT  GTCTGTTCTCTGGGTAC  TATCTG  CCAAGCCGGGAGGGAAGCCT
                           (TATA-)                             (INR+)                    (DPE-)                     
    152 Human: FCGR2B (2213, M90736) 
Mouse: Fcgr2b (14130, AK089266) 
 
           |-50      |-40      |-30      |-20      |-10      |1        |11       |21       |31       |41       
Human     AGAGAAGGCTGTGACTGCTGTGCTCTGGGCGCCAGCTCGCTCCAG  GGAGT G AT  GGGAATCCTGTCATTCTTACC  TGTCCT  TGCCACTGAGAGTGACTGGG
                                                         (INR-)                        (DPE-)                     
Mouse     TGCCAAGCCGGGAGGGAAGCCTGTGCCTGCAGCTGACTCGCTCCA  GAGCT G AT  GGGAATCCTGCCGTTCCTACT  GATCCC  CATGGAGAGCAACTGGACTG
                                                         (INR-)                        (DPE-)                     
    153 Human: FGR (2268, NM_005248) 
Mouse: Fgr (14191, AK036476) 
 
           |-50      |-40      |-30      |-20      |-10      |1        |11       |21       |31       |41       
Human     CCCGCCGGCCGTGACTGC  AATAAGA  GAAGTCCGAGGCGGCTTCCTCCTCC C TGCCCAGCAGGGGCGGCGG  TCAGAGGCGGGCA  GCACCCCAGTTCTCCCC
                             (TATA+)                                                   (MTE+)                     
Mouse     CTCCTCCGCAGTCACCAA  AATAAGA  GAAGTCCGAGACGGCTTCCTCCTCC C TCCCCAGAGTAGGTAGTAGGG  GCAGAGGCGGGTA  GCACCTCACTTTACC
                             (TATA+)                                                     (MTE+)                   
    154 Human: FHL3 (2275, CR604728) 
Mouse: Fhl3 (14201, AF114382) 
 
           |-50      |-40      |-30      |-20      |-10      |1        |11       |21       |31       |41       
Human     GGGCGGGC  CCGCGCTC  ACTTCCTCGGGGAGGGGTTGGGGCCGCGCTC  CCA C TCGC  CCACACGGTCCGTCTGC  AGTCGG  CAGCCTCGCCGGCAGCTCGCTC
                    (BRE+)                                   (INR+)                    (DPE+)                       
Mouse     GGGCGGGGCGGGCCCGAGCACTTCCTCGGAGGCTTAGGGCCGCGCGC  CCA C TCGC  CCACACAGTCAGTC  GCAGCCGGCTACC  TCGCCGCTGCTCTCTCGG
                                                           (INR+)                     (MTE-)                      
    155 Human: FMO1 (2326, NM_002021) 
Mouse: Fmo1 (14261, AK042457) 
 
           |-50      |-40      |-30      |-20      |-10      |1        |11       |21       |31       |41       
Human     ATAAACAACGGCTAACCTTGGGTT  TCAAATT  TAACATTCCTTATCTC  TTA G ACCA  GGTTTATCCACTGT  GCTGGGGACTCCC  AAGCCAGCACTGGCTCAT
                                   (TATA-)                   (INR+)                     (MTE-)                      
Mouse     ACAGCTAACCTTCGGTTC  CAATTTT  TTTTCCTAACGCTTCTTATCTC  TTA G ACCA  GGCTCATCCTGTGTGCAGG  GAACTC  CCAAACACTGGCTCAAAACA
                             (TATA-)                         (INR+)                      (DPE-)                     
    156 Human: FMO2 (2327, NM_001460) 
Mouse: Fmo2 (55990, AF184981) 
 
           |-50      |-40      |-30      |-20      |-10      |1        |11       |21       |31       |41       
Human     TCTCACAGCCACCTC  CAACTCT  TAAAAACGCTTCCAACTGCCTCCCAGC  A C ACAACC  AAGGGAGAAAACTATTCTGTCAAAG  AGACGG  TGCCAAAAGGCA
                          (TATA-)                              (INR+)                            (DPE+)             
Mouse     CCCACTTCTCACAGCCTCCTC  CATCTCT  TAAATACTCCCATCTCTCC  GCA C ACAA  CCCTCAGGG  GGAACTCTTCTGC  CAAGA  GGACAG  TGCTAAGAAGCA
                                (TATA-)                      (INR+)                (MTE+)          (DPE+)             
    157 Human: FMO4 (2329, AL031274) 
Mouse: Fmo4 (226564, NM_144878) 
 
           |-50      |-40      |-30      |-20      |-10      |1        |11       |21       |31       |41       
Human     TACTTTCTGCTGTCACTAGCATAA  CAGAAAA  GACGAATGGCTTTTGTTTT   A AACTGAG  GGCACTTTTACAAAAGT  CATCTT  TCCATTTATTTTTCTTTCA
                                   (TATA+)                      (INR-)                    (DPE-)                    
Mouse     AACTCCCTGTTGTCCATAGCCTGG  CAGAAGA  AGCCACCGGTTTTTGTGTT   A AACTGAG  AGTTTGACCAAAG  TCAGCAAAGTGAG  CACTGTTTCCACTTCA
                                   (TATA+)                      (INR-)                    (MTE+)                    
    158 Human: FMOD (2331, BC035281) 
Mouse: Fmod (14264, AK054183) 
 
           |-50      |-40      |-30      |-20      |-10      |1        |11       |21       |31       |41       
Human     CACGCACACTCTCAGTAGACTCTTTCACTCCTCTCTCTCTTCCTCTC  TCA C ACGT  TCTCCAACCCAAGGAGGCC  AGACAG  AGGGACGTGGTCACTCTCTG
                                                           (INR+)                      (DPE+)                     
Mouse     ACAGACTTGCACACTCTCCGTAGGCGCTCACTCCTCTCTTCCCTCTCTG  C C ACATTC  TCCAACCCAAGGAGACC  AGACAG  AAGGACGTGGTCACTCTGAA
                                                             (INR+)                    (DPE+)                     
    159 Human: FRAP1 (2475, NM_004958) 
Mouse: Frap1 (56717, AF152838) 
 
           |-50      |-40      |-30      |-20      |-10      |1        |11       |21       |31       |41       
Human     TTTTCAGTCCATCTTCTCCCTATACCTGTCGATTGGTCCTCAGGGCTGGG A ACCCTCCTTCCCTC  GCTCCCGGCTTAG  A  GGACAG  CGGGGAAGGCGGGCG
                                                                                (MTE-)      (DPE+)                
Mouse     GTTCCTCCCCATCTTCGAGCTCGGCCCGCTGATTGGTTGTAGCGCTTACG A GCCCGCCCTCCCCAC  CTCCCTGGCCTAG  A  AGACAG  CGGGGAAGGCGGGT
                                                                                 (MTE-)      (DPE+)               
    160 Human: NR5A2 (2494, AF319643) 
Mouse: Nr5a2 (26424, AF239709) 
 
           |-50      |-40      |-30      |-20      |-10      |1        |11       |21       |31       |41       
Human     CTTATCTCTTTTGCT  GTCACTT  TATAACAGGGTCCTCTTATCAACCTGCA T   AGAGTCAT  GTGATGAGTC  AAAGCCTATCAAA  AGTTTCCATTCTTCTGAT
                          (TATA-)                                (INR-)                 (MTE+)                      
Mouse     CTTATCTCTTTTGCT  GTCACTT  TATAACAGGGTCCTCTTATCAACCGGCA C AGGGTCATGTGACCAGTT  GAAGCCTATTGAA  AGCTTCCTTTCTTCTGAT
                          (TATA-)                                                     (MTE+)                      
    161 Human: NR5A2 (2494, U93553) 
Mouse: Nr5a2 (26424, AF239709) 
 
           |-50      |-40      |-30      |-20      |-10      |1        |11       |21       |31       |41       
Human     TTCTGATTAGAAACCAT  CATGAAA  CTGGATACATGGTTTACAGCAGG  TCA C TAAT  GTTGGAAAAAGTAC  AGAGTCCAGGGAA  AGACTTGCTTGTAACTTT
                            (TATA+)                          (INR+)                     (MTE+)                      
Mouse     TTCTGATTAGAAACCAT  CATGAAA  CTGGAGACATGGTTTACAGCAGGTC  A T ACATGC  TGGAAAAAGTGC  AGAGTCCAGGAGA  ACACGGACTGGAGCCTTT
                            (TATA+)                            (INR+)                   (MTE+)                      
    162 Human: FUCA1 (2517, NM_000147) 
Mouse: Fuca1 (71665, AK020610) 
 
           |-50      |-40      |-30      |-20      |-10      |1        |11       |21       |31       |41       
Human     CTTTTTCCTTCCGGGCCAATCGTTAGTCAGAGTGGGCGGAGCCGCCCGCG G GCACCTGCGCGTTAAGAGTGGG  CCGCGTCGCTGAG  GGGTAGCGATGCGG
                                                                                        (MTE-)                  
Mouse     CCCCTCTCCTT  CCAAGCCA  ATCGTTACTCTGAGAGGCGGGGCTACTCGCC G GCGCTTGCGTGTTAAGA  GGGGGGCGGTGCG  GGCCCGGAAGCAAGGATGT
                       (BRE+)                                                        (MTE-)                       
    163 Human: FY (2532, NM_002036) 
Mouse: Dfy (13349, AF016697) 
 
           |-50      |-40      |-30      |-20      |-10      |1        |11       |21       |31       |41       
Human     GTAAGGCTTCCTGATGCCCCCTGTCCCTGCCCAGAACCTGATGGCCCTCA   T TAGTCCT  TGGCTCTTATCTTG  GAAGCACAGGCGC  TGACAGCCGTCCCAG
                                                              (INR+)                     (MTE+)                   
Mouse     TATATATACACACACACACCCTGGCTCTGCCTAGAACCCGAGAGAAG  TCA T TAGC  CCTGGGCACTTATCTT  GGAGCCACAGCTG  CTGACAGAGTCCAGGC
                                                           (INR+)                       (MTE+)                    
    164 Human: FY (2532, Y14873) 
Mouse: Dfy (13349, AK010883) 
 
           |-50      |-40      |-30      |-20      |-10      |1        |11       |21       |31       |41       
Human     TTATCTTGGAAGCACA  GGCGCTGA  CAGCCGTCCCAGCCCTTCTGTCTGCG G GCCTGAACCAAACGGTGCCATGGGGAAC  TGTCTG  CACAGGGTGAGTATG
                            (BRE-)                                                          (DPE-)                
Mouse     CTTATCTTGGAGCCACAGCTGCTGACAGAGTCCAGGCCCTGTACTTCTCT G CCCTGAGCCTGCAGTGCCATGGGGAAC  TGTCTG  TATCCGGTGAGTGTGG
                                                                                         (DPE-)                 
    165 Human: GABRD (2563, NM_000815) 
Mouse: Gabrd (14403, NM_008072) 
 
           |-50      |-40      |-30      |-20      |-10      |1        |11       |21       |31       |41       
Human     GGCCGCCGGGAGCCAAGTTTGCGCGGACCCCGTCCCGAGCCCGCCGCGGC C ATGGACGCGCCCGCCCGGCTGC  TGGCCCCGCTCCT  GCTCCTCTGCGCGC
                                                                                        (MTE-)                  
Mouse     TTGCTTGCGCTGGGGCTAGCTGGACCTGTCCCGCGCACAGCCCGCAAGGC C ATGGACGTTCTGGGCTGGCTGC  TGCTGCCGCTCCT  GCTGCTCTGCACGC
                                                                                        (MTE-)                  
    166 Human: GALE (2582, L41668) 
Mouse: Gale (74246, BE653500) 
 
           |-50      |-40      |-30      |-20      |-10      |1        |11       |21       |31       |41       
Human     CTTCCTGCGACCCTGGAGTCCCAATCGTGCTGCCATTTTCCTTCTGCCCA   G GACTCTC  CAGTCCTCAGTCACCTTGGACAAAGAAGTGTGGATCCTCAGA
                                                              (INR+)                                            
Mouse     CCTTTTTCTGTATCCTAGCCTC  CCATTCT  TAAGCGATGTTCTTCTGCCCG   A GACTGTC  CATACTCCAGACATGGAGGCAGGAAGGGTGGGTCTTCAAAAG
                                 (TATA-)                        (INR-)                                            
    167 Human: GALE (2582, AL031295) 
Mouse: Gale (74246, BY788290) 
 
           |-50      |-40      |-30      |-20      |-10      |1        |11       |21       |31       |41       
Human     CTCCTAGCTCGGCC  CCGCTCCG  CGCTACAGCCCAAAGCCCAGCCTGAGAC C CAGCCGCCCTCGTATGCCCA  CGGCCTAGCTCCC  CTTCCCTCCAACAGAC
                          (BRE+)                                                        (MTE-)                    
Mouse     TCGGCTCC  CCGCTCCT  GCTCCCAGCCCAGACCCGAAGTCCAGAGCTAAGC T CCGCCTCGCCCAGGCTGCTGAGACCCAG  CATCCG  CTCTCCTGCCTTGTA
                    (BRE+)                                                                  (DPE-)                
    168 Human: GALE (2582, BM807767) 
Mouse: Gale (74246, BE653500) 
 
           |-50      |-40      |-30      |-20      |-10      |1        |11       |21       |31       |41       
Human     CCCAGTTCCCCGCTGCCCGGGCTGGGGCGGCCGGGGTTCATTCATTGCCG G CCTGAGGACTCCTAGCT  CGGCCCCGCTCCG  CGCTACAGCCCAAAGCCCA
                                                                                   (MTE-)                       
Mouse     TCGCGTTTTCCGCAACCGTAGCGGGGGCCACCCGAGCCCATTCATTCGGG G CTCGGCTCCCCGCTCCTGCTC  CCAGCCCAGACCC  GAAGTCCAGAGCTAA
                                                                                       (MTE+)                   
    169 Human: GALE (2582, NM_000403) 
Mouse: Gale (74246, BE653500) 
 
           |-50      |-40      |-30      |-20      |-10      |1        |11       |21       |31       |41       
Human     TTGGGGGCGTGGAGG  GGCGGGGT  CGGGGCCGGAGTGGGCCGTCA  GCACTT A A  AGGGCCCGCGGCTCGGGCGT  AGGAGGCGGTGCC  TCTGCAGCAAGCGTG
                           (BRE-)                         (INR+)                           (MTE-)                   
Mouse     GTTTCCTGGAG  GGCGTGGA  GGGGCGGGGTCGGGCTGGGCTGTCAGGTCTT   A AAGGGGC  CGCGGCGCTGGTCGC  GGGAGGCGGTGCC  TGGAGTCAGCGCTG
                       (BRE-)                                   (INR-)                      (MTE-)                  
    170 Human: GALNT2 (2590, NM_004481) 
Mouse: Galnt2 (108148, NM_139272) 
 
           |-50      |-40      |-30      |-20      |-10      |1        |11       |21       |31       |41       
Human     CTCCGCTCCTCCCCCGGCCCCCACCGCGCCCGCGGCCGGCCCAGGCA  GCA C TCGC  GAGCAGCGG  CGGCCCCGCCGGC  GGCCG  AGTTGG  GAGAATGCGGCG
                                                           (INR+)                (MTE-)          (DPE+)             
Mouse     TCCCGCGCCG  CCGCCCCG  AGCTCCGCCCGCCGCGCGCTCCGCCCGCCGCG C CG  CCAGGCTG  CCCG  GCGGCCGCCCAGA  GCGA  GGATGC  GGCGGCGCTCGC
                      (BRE+)                                       (INR+)           (MTE+)         (DPE+)             
    171 Human: GBA (2629, NM_000157) 
Mouse: Gba (14466, AK082767) 
 
           |-50      |-40      |-30      |-20      |-10      |1        |11       |21       |31       |41       
Human     TGACGCTCCTAGTCATCACATGACCCATCCACATCGGGAAGCCGGAA  TTA C TTGC  AGGGCTAAC  CTAGTGCCTATAG  CTAAGGCAGGTACCTGCATCCTT
                                                           (INR+)                (MTE+)                           
Mouse     GACCGGCTTAGTCATCATGTGACTCTGTCCGCCCCTTTAAGCCGGAAG  GA A CTTGC  TGTGCTAGTCGGT  TCCAAGAGCTATG  ATATCTGTCTGTGTTTTA
                                                            (INR+)                    (MTE-)                      
    172 Human: GBA (2629, M18916) 
Mouse: Gba (14466, AK082767) 
 
           |-50      |-40      |-30      |-20      |-10      |1        |11       |21       |31       |41       
Human     ACCCCATTCTCCATGCAAATCTGTGTTCTAGGCTCTTCCTAAAGTTGTCA C CC  ATACATGC  CCTCCAGAGTTTTATAGGGCATATAATCTGTAACAGATG
                                                                 (INR+)                                         
Mouse     ACTCAGCTGTCCATACAATCCTTGTTAGAATTCTCTTCCTAAAGGTGTCA   T CCTTTAG  GCCTCCCCAAAATTCACAGGGTATGCAATGAATGACTAACCT
                                                              (INR+)                                            
    173 Human: GJA4 (2701, M96789) 
Mouse: Gja4 (14612, AF216832) 
 
           |-50      |-40      |-30      |-20      |-10      |1        |11       |21       |31       |41       
Human     ACCCCGCCCCTCTGCGCTA  TTTAAGG  CGCCCCCGCCGCTCGTGCGGTCCA G CAGGGCTCCCGCGGGCGTCACTCCGGCCATCGTCCCCACCTCCACCTGG
                              (TATA+)                                                                           
Mouse     AAACCCGCAGCTGCGCGCTA  TTTAAGG  CGTCCTGCGGGAGGCGCGGTCCT G CAGGATTCCCGCAGGCGCAGCTTCGGCCACGGTCGTCCCCTCTACCTGG
                               (TATA+)                                                                          
    174 Human: GJA5 (2702, AY165050) 
Mouse: Gja5 (14613, AF025766) 
 
           |-50      |-40      |-30      |-20      |-10      |1        |11       |21       |31       |41       
Human     GAGGGAAGGCGACAGATACG  ATTAAAA  AGACGGTGGAAGAGGAA  CAACTG A C  AGGCTCAAGAGCAAAAAGCGTGGGC  AGTTGG  AGAAGAAGCAGCCAGAG
                               (TATA+)                    (INR-)                            (DPE+)                  
Mouse     AGGGAGGGCAATGGGATACCG  GTTAAAA  AGAGGGTGGGCGGGAG  CAACTA A C  AGGCTCAAGAGCAAATAACAGTGGGC  AGTTGA  ACAGCAGCCAGAGCCT
                                (TATA+)                   (INR-)                             (DPE+)                 
    175 Human: GJA8 (2703, U34802) 
Mouse: Gja8 (14616, CV557810) 
 
           |-50      |-40      |-30      |-20      |-10      |1        |11       |21       |31       |41       
Human     GGCTTTTAGGCAGCAAAGGAAAGAGACGAAAGTTGCCATTTTGCTGCTGA G CGCCAAGAGAGAA  AGAGCACATATTT  CTCCGTGGGACACTCCTTGTATT
                                                                               (MTE+)                           
Mouse     GGCTTTTAGGTGGCAAAGGAAAGACACCAAAGTTGCCATTTTACTGCGGA G CAGCAAGAGAGAAAG  ACAGCACCAGTTT  CTCCGTGGGACGCTACTCAGA
                                                                                 (MTE+)                         
    176 Human: GJB3 (2707, NM_024009) 
Mouse: Gjb3 (14620, AF288667) 
 
           |-50      |-40      |-30      |-20      |-10      |1        |11       |21       |31       |41       
Human     CGAGTCCTGATTGCAGTCGGACCTGCCGCCGCGGCACTTAACAGTTTGC  A G AGTGCT  TCCCGCCCC  TGATCTCATTGGA  GCCTTCGGACAGCCCAGCCCA
                                                             (INR-)                (MTE+)                         
Mouse     GGCGTGTCATTTATTGCCATCCCTACTGCCACGGCACTTAACAGTTTGC  A A GGTGTT  TCACACCCTAATG  TCATTGCAGTCCT  TGCAACAGCCCTGCCAG
                                                             (INR-)                    (MTE+)                     
    177 Human: GJB3 (2707, NM_024009) 
Mouse: Gjb3 (14620, NM_008126) 
 
           |-50      |-40      |-30      |-20      |-10      |1        |11       |21       |31       |41       
Human     TGAAGCTCCTCCCTGGGGTGTGTCAGGAGGGACTGGGCAGACAGCACCCA A GGGTAGAAGATGGGGGCCCA  GGAGGGAGCTGCA  CCCTGGTGGGCAGCCA
                                                                                      (MTE-)                    
Mouse     TGAGTCTGTCTCGGGGTGTGTCT  TAGAAGG  GACTAGGTAGCCTGCACC  AA A ATGGG  GAGCCCCAGACGTG  GCATCCTTAGGGG  GTACCGGGTGGAAGGAA
                                  (TATA+)                     (INR-)                     (MTE+)                     
    178 Human: GCLM (2730, NM_002061) 
Mouse: Gclm (14630, AF149054) 
 
           |-50      |-40      |-30      |-20      |-10      |1        |11       |21       |31       |41       
Human     TTCTCGGCTACGATTTCTGCTTAGTCATTGTCTTCCAGGAAACAGCTCCC   T CAGTTTG  GAATCAG  CTCTCCCGCTGCG  GCCGCAGTAGCCGGAGCCGGAG
                                                              (INR+)              (MTE-)                          
Mouse     TTCTCGGGCGAGGTTTCTGCTTAGTCATTGTCTTCCAGGAAACAGCTCCC   T CGTTTCA  GCGGCG  GCGGCGGTGGCGG  CCGCAGAAGCCGCAGCGACCGTC
                                                              (INR+)             (MTE+)                           
    179 Human: GLUL (2752, NM_002065) 
Mouse: Glul (14645, AY044241) 
 
           |-50      |-40      |-30      |-20      |-10      |1        |11       |21       |31       |41       
Human     GGGGGCGGCC  CCGGGCCG  CG  GATAAAG  GGTGCGGGGCTGCTGGCGGCTCT G C  AGAGTCGA  GAGTGGGAGA  AGAGCGGAGCGTG  TGAGCAGTACTGCGGCC
                      (BRE+)     (TATA+)                            (INR-)                 (MTE+)                     
Mouse     GGGGGCGGGCACAAGCTGTC  AATAAAA  AGTACCGAGCAGCCAGCGACCCT G C  AGAGCGGA  GAATGGGAGTAGAGCAGAG  TGTCTG  AACAGCACGCTCACC
                               (TATA+)                            (INR-)                      (DPE-)                
    180 Human: GNAI3 (2773, BC025285) 
Mouse: Gnai3 (14679, AK187104) 
 
           |-50      |-40      |-30      |-20      |-10      |1        |11       |21       |31       |41       
Human     ATCCGGTTCTTCTGGGCGCTAAGGGAGCTGACGGAGAGGGCCACCGCCCA G   CAATAGAC  GGTGCCTCAGCCTGC  CGAGCCGCAGTTT  CCGTGGTGTGAGT
                                                               (INR-)                      (MTE+)                 
Mouse     ATCCGGTTCTTCCGGGAGCTAGGGGAGCTGACGGAGAAGGCCACCGCCCA G CA  GAAGACCC  GTCTCCGCCGGT  GTGTGGCGATTCC  CGCGGTGTGTGTGA
                                                                 (INR+)                   (MTE-)                  
    181 Human: GNAT2 (2780, NM_005272) 
Mouse: Gnat2 (14686, BC085102) 
 
           |-50      |-40      |-30      |-20      |-10      |1        |11       |21       |31       |41       
Human     TGACCTATCACATTGCTAGA  TATAAAG  GCTACAATCCCTAGACTAA  GAAG T AGG  TCTCCAGTTGAAGTAGGGAGTCTCAGTCAATGTAGGCAGAGTACAA
                               (TATA+)                      (INR-)                                                
Mouse     TGATCTGTCATTCCGCTAGA  CATAAAG  GGGACAATCCCCGGATTAGGAAG G A  GCTCTCCA  GCTCGGGTAAGGAGTCTCAAGGCAAGGTAGGCAAGCACCA
                               (TATA+)                            (INR+)                                          
    182 Human: GNB1 (2782, AK122949) 
Mouse: Gnb1 (14688, BC013058) 
 
           |-50      |-40      |-30      |-20      |-10      |1        |11       |21       |31       |41       
Human     CGTGCGCGTGCCCGTCG  GGCCTGGC  GAGCGCGCGCGACGCGCGGCGTGCG T GGTGACGCGAGCGCGTGGGGTT  TTCCGAGGCTGCG  GGCAGGGGCCCGCC
                             (BRE-)                                                       (MTE-)                  
Mouse     GGGAAGGAGG  GGCGGGGC  GGAAAGAGCGGCACGCGCACGCCGCCCGTGCG C GGTGACGCGACGTGGGGTT  TCCCGAGGCTGCG  GGCAGGGGCCGCCGCGC
                      (BRE-)                                                           (MTE-)                     
    183 Human: GPX7 (2882, AK027683) 
Mouse: Gpx7 (67305, NM_024198) 
 
           |-50      |-40      |-30      |-20      |-10      |1        |11       |21       |31       |41       
Human     CGCCAGAGCCGCCTCCTC  CCGCCCCC  CGCGCTAGATCCCCCCGCCCCGTC T TTGCCCTCGCGACGCCGCCACCTCCGGAACAAGCCATGGTGGCGGCGAC
                              (BRE+)                                                                            
Mouse     AGCGGGCCGCCTCCTC  CCGCCCCA  CACGGCGGGGTCCCCTCCGCCTTGTC T TTGCCCTCGCCAAGTCGCCACCTCGGGAGTGAACCATGGTTGCAGCTGT
                            (BRE+)                                                                              
    184 Human: GSTM2 (2946, CR605552) 
Mouse: Gstm3 (14864, L13451) 
 
           |-50      |-40      |-30      |-20      |-10      |1        |11       |21       |31       |41       
Human     CTCACAAACGCTGAG  CCCCGCCC  CGCTGAGGCCTGTCTGCAGAAT  CCACA G CA  ACCAGCACCATGCCCATG  ACACTGGGGTACT  GGAACATCCGCGGGGT
                           (BRE+)                          (INR+)                         (MTE-)                    
Mouse     CTTAAAATTCTCCAGCTCAACCTACCTGAAGCCAGTCTGAGAAGA  CCACA G CA  TCAGCGCCATGCCTATG  ACACTGGGCTATT  GGAACACCCGCGGAGTG
                                                         (INR+)                        (MTE-)                     
    185 Human: GSTM3 (2947, J05459) 
Mouse: Gstm5 (14866, AK028098) 
 
           |-50      |-40      |-30      |-20      |-10      |1        |11       |21       |31       |41       
Human     TGTCGGGTATAAAGCCCCTCCCGCTCACAGTTTCCCTAGTCCTCGAAGGC T CGGAAGCCCGTCACCATGTCGTGCGAGT  CGTCTA  TGGTTCTCGGGTACT
                                                                                          (DPE-)                
Mouse     GTCCAGTATAAAGTTAGCCGCCCACAGTCCATCGCTGTATCCCC  GAAGGG G C  TAAGATCGCCCAAAATG  TCATCCAAGTCTA  TGGTTCTGGGTTACTGGG
                                                        (INR-)                        (MTE+)                      
    186 Human: GSTM5 (2949, NM_000851) 
Mouse: Gstm1 (14862, CA469386) 
 
           |-50      |-40      |-30      |-20      |-10      |1        |11       |21       |31       |41       
Human     TGTCCCCTCCTGGGCCTCTCAAAGTCTGAGCCCCGCTCCGCTGATGCCTG T CTGCAGAATCCGCACCAA  CCAGCACCATGCC  CATGACTCTGGGGTACTG
                                                                                    (MTE+)                      
Mouse     CTGTATAAAGTCACAACTCCAAACACACAGGTCAGTCCTGCTGAAG  CCAG T TTG  AGAAGACCACAGCA  CCAGCACCATGCC  TATGATACTGGGATACTGG
                                                          (INR+)                     (MTE+)                       
    187 Human: GTF2B (2959, X59268) 
Mouse: Gtf2b (229906, NM_145546) 
 
           |-50      |-40      |-30      |-20      |-10      |1        |11       |21       |31       |41       
Human     AACTCATCTCT  GCGCGTCT  CTTCGCCACATTCGCTTCCTGCTTTC  GGTGT G TC  TGTTGTGTCTTGTTGCGGGCACCGC  AGTCGC  CGTGAAGATGGCGTCT
                       (BRE+)                              (INR-)                            (DPE+)                 
Mouse     AGCCCGGCTCGG  GGCGTCTA  GGAGCCAGTGCTGCTTCCTGCTTCGGACG  G A GCTGTT  GGCGGAGCCGCGAAGATGGCGT  CGACCA  GCCGGTGAGTGCAAC
                        (BRE-)                                 (INR-)                         (DPE-)                
    188 Human: GUCA2A (2980, Z68182) 
Mouse: Guca2a (14915, AK075814) 
 
           |-50      |-40      |-30      |-20      |-10      |1        |11       |21       |31       |41       
Human     CTCAGTAACCTGCCCTC  TTTAAAA  GTCCCGCCGCTTCCCCCTGGCATCCA   G AACAGCC  ACCCCTCTCTC  GGGCACTGCTGCC  ATGAATGCCTTCCTGCTC
                            (TATA+)                             (INR-)                  (MTE-)                      
Mouse     TGAGTAACCCCAACCTC  TTTAAAA  GCCCCACTGTTTACCCCAG  GCACTAG T   ACTGGCCTGTTCTCTGCATT  GCATACTGCTACC  ATGAATGCCTGTGTGC
                            (TATA+)                      (INR+)                           (MTE-)                    
    189 Human: GUCA2B (2981, Z70295) 
Mouse: Guca2b (14916, NM_008191) 
 
           |-50      |-40      |-30      |-20      |-10      |1        |11       |21       |31       |41       
Human     CAGGACTCCTGAGCCTGACC  TATAAGG  AGGTGCTAGGCAGGGAC  ACAGAT G G  GAGACGGTGGACAGCGGCAGGG  GGAACCCAGGGAG  CGCGATGGGCTGC
                               (TATA+)                    (INR+)                             (MTE+)                 
Mouse     CCAGAGCTCTGGACCCTGGCC  TATAAGG  GGCTGCTGGCCCAGAGG  CAGGT G GC  AGGCAGGTGGACAGCAGAG  GAAGCAGGAACCC  AGAGGTGTGAGCTTG
                                (TATA+)                    (INR-)                          (MTE+)                   
    190 Human: GUK1 (2987, BC006249) 
Mouse: Guk1 (14923, AA144893) 
 
           |-50      |-40      |-30      |-20      |-10      |1        |11       |21       |31       |41       
Human     CGGTGCGGCGCTGTCACGTAGGTTCAGTGGGCGGAAGAGGTGGCCCCGGA T GCTGCGGCGCCCGC  TGGCCGGGCTGGC  TGCGGCCGCCCTGGGCCGGGCC
                                                                                (MTE-)                          
Mouse     TCCGGTGGGCCG  GCGCGCCG  CTGTGACGTAGTCCTGCGGGCGGTCCCTGA T GCTGCGGCGCCCGC  TGGTCGGGCTGGC  GGTGGCCGCCCTGGGTCGGGTC
                        (BRE+)                                                    (MTE-)                          
    191 Human: H3F3A (3020, D28384) 
Mouse: H3f3a (15078, AK037900) 
 
           |-50      |-40      |-30      |-20      |-10      |1        |11       |21       |31       |41       
Human     CGCGGGGGCGGGGC  GGCGTGTG  TTGGGGGATAGCCTCGGTGTCAGCCATC T T  TCAATTGT  GTTC  GCAGCCGCCGCCG  CGCCGCCGTCGCTCTCCAACGCC
                          (BRE-)                                  (INR+)           (MTE+)                           
Mouse     CCAGTTAGGACCTTGGGATTTGG  TATAAGG  ATATCTTTAAGAAGA  GCATT T CA  GAATTGTGTTC  GCAGCCTTCGCCG  CGCCGCCGCCGCTCTCCAACGCC
                                  (TATA+)                  (INR+)                  (MTE+)                           
    192 Human: H3F3A (3020, M77656) 
Mouse: H3f3a (15078, BC002268) 
 
           |-50      |-40      |-30      |-20      |-10      |1        |11       |21       |31       |41       
Human     CCGCCGCGCCGCCGTCGCTCTCCAACGCCAGCGCCGCCTCTCGCTCGCCG A GCTCCAGCCGAAGGAGAAGGGGGGTAAGTTTCCCCGTCTGCCCGCTTCC
                                                                                                              
Mouse     TCGCCGCGC  CGCCGCCG  CTCTCCAACGCCAGCGCCGCCTCTCCCTTGCCG A GCTCCAGCCGAAGGAGAAGGGGGGGTAAGTAAGGAGGTGTCCATACCAT
                     (BRE+)                                                                                     
    193 Human: HDAC1 (3065, NM_004964) 
Mouse: Hdac1-ps (15181, BF607595) 
 
           |-50      |-40      |-30      |-20      |-10      |1        |11       |21       |31       |41       
Human     GGGGACGGGC  GGGGCGGG  CCGGAGGCCCGCCCCCTCCCCCCTGGGTCGGA C GCTGAGCGGAGCCG  CGGGCGGGAGGGC  GGACGGACCGACTGACGGTAGG
                      (BRE-)                                                      (MTE+)                          
Mouse     GATCCCGGGGGTG  GGCGGGGC  TTCGAGGCCGGCCCCTCCCCCTGGTCGGA C GCCGTAAGGAGCCGCGGGCGGG  CGGGCGGACAGAC  TGACGGGAGGGCCG
                         (BRE-)                                                           (MTE+)                  
    194 Human: HDGF (3068, D16431) 
Mouse: Hdgf (15191, AK017863) 
 
           |-50      |-40      |-30      |-20      |-10      |1        |11       |21       |31       |41       
Human     GGGGGTGGAGGAAGA  GGCCTCGC  GCAGAGGAGGGAGCAATTGAATT  TCAA A CAC  AAACAACTGCA  CGAGCGCGCACCC  ACCGCGCCGGAGCCTTGCCCCG
                           (BRE-)                           (INR+)                  (MTE+)                          
Mouse     GTGGAGGAAGC  GGCCTCGC  TCGCGCCGAGGAGGGAGCAATTGAATT  TCAA A CAC  AAACAACCACACGAG  CTAGCGCTCCCGG  AGCCGTGTGTTGCCCACC
                       (BRE-)                               (INR+)                      (MTE+)                      
    195 Human: CFH (3075, NM_000186) 
Mouse: Cfh (12628, J02891) 
 
           |-50      |-40      |-30      |-20      |-10      |1        |11       |21       |31       |41       
Human     TATTTACTTTGCAAAAGTTTCTGATAGGCGGAGCATCTAGTTT  CAACTTC C   TTTTGCAGCAAGTTCT  TTCCTGCACTAAT  CACAATTCTTGGAAGAGGAG
                                                       (INR+)                       (MTE-)                        
Mouse     GTTGCTCATGGGCGGAGCAATCCTGATTTCCTAAACTGACTTT  CAACTTC C   CTTTGAAGCAAGTC  TTTCCCTGCTGTG  ACCACAGTTCATAGCAGAGAGG
                                                       (INR+)                     (MTE-)                          
    196 Human: HKR3 (3104, NM_005341) 
Mouse: Hkr3 (100090, AK027912) 
 
           |-50      |-40      |-30      |-20      |-10      |1        |11       |21       |31       |41       
Human     GGGCCGGAGCTAGAAGCTCTACGCTTCCGAGGCGCACCTCCTGGCCTGC  A C GCTTTG  ACGTCACGTCCGGCGCGG  AGACGG  TGGAGTCTCCGCACTGTCG
                                                             (INR+)                     (DPE+)                    
Mouse     GGCGGGACCCGAGGACATTGCGCCACGGAGGCGTCCGGTTCTTGCCTGCG C GC  CTGTATGT  CACATCCGGCG  CGGCGGCGGTGGC  GTCTCCGCGCTGTGG
                                                                 (INR+)                  (MTE-)                   
    197 Human: MR1 (3140, NM_001531) 
Mouse: Mr1 (15064, AF068691) 
 
           |-50      |-40      |-30      |-20      |-10      |1        |11       |21       |31       |41       
Human     CTTGTGTGTCACCAAGAGGTTCTCAGAAGGGACCTGTCAGTTTTTGGT  TA A AAGAA  CCCGGAAA  GAGAAGGACTATG  GGG  GAACTG  ATGGCGTTCCTGTT
                                                            (INR-)               (MTE-)        (DPE-)               
Mouse     AGTACAAGTTCTCAAAAGATTCATCAGGGGTCTGTCACTGTCTGAGGTTA A A  AAAAAAAA  TCGGAAAAGCAAA  GGACTT  CAGCACGGGTTGATGATGCTC
                                                                (INR-)                (DPE-)                      
    198 Human: HLX1 (3142, NM_021958) 
Mouse: Hlx1 (15284, AF172318) 
 
           |-50      |-40      |-30      |-20      |-10      |1        |11       |21       |31       |41       
Human     TGAGCGCTCGGATCGAGGTCCTACCCCGGGCCTGACTCGAAAGCTCCTGC C A  AAACTTTG  GGAGTTTTT  AGAGACGAGTTTT  TTTTTTTTTCTATTACTT
                                                                (INR+)                (MTE+)                      
Mouse     TGAGCGCACGGATCAAGGTCCTACCCGGGGTCTGACTCGAAAGCTCCTGC C A  AAACTTTG  GGAGTTTTT  AGAGAGGAGTTTT  TTTTTTTTTTTTAATTTT
                                                                (INR+)                (MTE+)                      
    199 Human: HLX1 (3142, U14325) 
Mouse: Hlx1 (15284, AF172318) 
 
           |-50      |-40      |-30      |-20      |-10      |1        |11       |21       |31       |41       
Human     CCACCCAGTCCGGCTGGACTGCGGCAGCCGCGCGGCTCACCCCGGCAG  GA T GTTCG  CAGCCGGGCTGGCTCCCTTCTACGCCTCCAACTTCAGCCTCTGG
                                                            (INR-)                                              
Mouse     GCCCACCCACTTCCCCACTCACCCAGGACCCCGCCGGGATCCTGCCAG  GA T GTTCG  CAGCCGGCCTGGCTCCCTTCTATGCTTCCAACTTTAGTCTCTGG
                                                            (INR-)                                              
    200 Human: HMGCL (3155, CR593217) 
Mouse: Hmgcl (15356, AA125224) 
 
           |-50      |-40      |-30      |-20      |-10      |1        |11       |21       |31       |41       
Human     ACCAGACGAGCCATCGGTCACGCGGGGCCCAGCTGGACTGCCGCGGGGGA T TCTGGGCCAAGATG  GCAGCAATGAGGA  AGGCGCTTCCGCGGCGACTGGT
                                                                                (MTE+)                          
Mouse     ACCACGCCCACAGAGGGATCGGCGGTCACGTGGTGCCCAGTGGGTTGGCC T CC  GGAGTGAA  GATGGCGTCAGT  GAGGAAGGCTTTC  CCGCGGAGGCTGGT
                                                                 (INR-)                   (MTE-)                  
    201 Human: HMGCS2 (3158, U81851) 
Mouse: Hmgcs2 (15360, NM_008256) 
 
           |-50      |-40      |-30      |-20      |-10      |1        |11       |21       |31       |41       
Human     AGGCAGGAGTCTTGGGGCTT  TATAAAG  TCCTGCCGGGCACCACTGGGCA  T C TCTTTC  AAGGTTT  CTGCTGGGTTTCT  GAACTGCTGGGTTTCTGCTTGCT
                               (TATA+)                         (INR+)              (MTE-)                           
Mouse     GCTTCCAGAGCCTGGAGTCT  TATAAAG  CCCAACCCAGAATCGTTGG  GCAT C TCT  CCCAAGGGCTGTGG  ACTGCTGGCTTTC  TGTTGATACCTTAGAGATG
                               (TATA+)                      (INR+)                     (MTE-)                       
    202 Human: HNRPU (3192, AK126868) 
Mouse: Hnrpu (51810, BM250848) 
 
           |-50      |-40      |-30      |-20      |-10      |1        |11       |21       |31       |41       
Human     GTGCTCGC  TCGCGCCA  GGCGAGTCTCCGCGTCTCCCTCGCGAACTCGGTG A AA  GGAATTGG  CGCCGTTCGACA  CCAGGCGGATCCG  CTCTGCAGCACGAA
                    (BRE+)                                         (INR-)                   (MTE-)                  
Mouse     TGCTCGCT  CGCGCCGG  GCGAGTCTCCGCGTCTTGCCTCGCGAACTCGGTG A AA  GGAATTGG  CGCCGTTCGACA  CCAGGCGGGTCCG  TTCTGCAGCAGCAC
                    (BRE-)                                         (INR-)                   (MTE-)                  
    203 Human: HPCA (3208, BC001777) 
Mouse: Hpca (15444, AB015199) 
 
           |-50      |-40      |-30      |-20      |-10      |1        |11       |21       |31       |41       
Human     CCGCCCGCCCCTCCTG  CCGCCCCT  GCCGCGCGCGCCGCAGCAGCGCCGCC T TCCCTGCGCAGTCGGTGTCT  CCGCGTCGCTGGG  TGAGTGGGGGCAGCTC
                            (BRE+)                                                      (MTE-)                    
Mouse     CGCCCGCCCCTCC  GGCCGCCT  CTGCCGCCGCGCGCCGCAGCAGCGCCGCC T TCTCTGCGCAGTCGGTG  TCTCGGCGTTGCT  GGGTGAGTGGGGGCCGCTC
                         (BRE+)                                                      (MTE-)                       
    204 Human: HSD3B1 (3283, NM_000862) 
Mouse: Hsd3b6 (15497, AY046511) 
 
           |-50      |-40      |-30      |-20      |-10      |1        |11       |21       |31       |41       
Human     TTGGGCCAGAAGCCACAGTG  CATAAAG  CTTCAGACTTGCCACAG  GAAATG A G  GTGAGAAGTACGTCCACTCTT  CTGTCCAGCTTTT  AACAATCTAACTAA
                               (TATA+)                    (INR-)                            (MTE-)                  
Mouse     GATGAGTCAGAAGACCAGAG  ATTAAAA  CACTAGTTCTGATCTGA  GGGCTG A G  GAGACCAGCATCCAGACTCTCC  CATCTG  ACTTTTAACAATTTAACAGG
                               (TATA+)                    (INR-)                         (DPE-)                     
    205 Human: HSD3B2 (3284, NM_000198) 
Mouse: Hsd3b1 (15492, NM_008293) 
 
           |-50      |-40      |-30      |-20      |-10      |1        |11       |21       |31       |41       
Human     GGAGACTTCTCCCAGTTTGG  TTTAAGT  TCACAGATTGCAGATC  CCAGACA G   CTGGTATCAACTGACCAGTGTTCTGTTAAGGCTAAAGCCAAGACTCTTT
                               (TATA+)                   (INR+)                                                   
Mouse     GTTGTTTGTTTGTTTTTGCAG  TTGAAAC  TCACACACTGAACCCCAAGACA T   CTTGTGAA  CTGGTGG  CTGTTTTGCTGAT  AGAAAACCTAAGTCTTTACTA
                                (TATA+)                          (INR-)              (MTE-)                         
    206 Human: HSD3B2 (3284, M77144) 
Mouse: Hsd3b1 (15492, NM_008293) 
 
           |-50      |-40      |-30      |-20      |-10      |1        |11       |21       |31       |41       
Human     CTGAGACACAAGCCACAGAG  CATAAAG  CTCCAGTCCTTCCTCCAGGGATG A G  GCAGTAAG  GACTTGGACTCCT  CTGTCCAGCTTTT  AACAATCTAAGTTA
                               (TATA+)                            (INR-)                    (MTE-)                  
Mouse     GATAAGTCAGCAGACTAGAG  ATTAAAA  CACTAGTCCTGATCTGAGGGCTG A   GGAGATCA  GCATCCAGACACTCT  CATCTG  ACTTTTAACAATTTAACAGG
                               (TATA+)                           (INR+)                  (DPE-)                     
    207 Human: HSD11B1 (3290, NM_005525) 
Mouse: Hsd11b1 (15483, NM_008288) 
 
           |-50      |-40      |-30      |-20      |-10      |1        |11       |21       |31       |41       
Human     GGCTAGCACTGCCTGAGACTACTCCAGCCTCCCCCGTCCCTGATGTC  ACA A TTCA  GAGGCTGCTGCCTGCTTAGGA  GGTTGT  AGAAAGCTCTGTAGGTTC
                                                           (INR+)                        (DPE+)                   
Mouse     GGCTAGTGCTGCCTGAGACTACTCCAGCCTCCCCCGTCCCTGATGTC  ACA A TTCA  GAGGCTGCTGCCTGCCTGGGA  GGTTGT  AGAAAGCTCTGCAGGTTT
                                                           (INR+)                        (DPE+)                   
    208 Human: ID3 (3399, NM_002167) 
Mouse: Id3 (15903, M60523) 
 
           |-50      |-40      |-30      |-20      |-10      |1        |11       |21       |31       |41       
Human     GAGGCGGGCCATTTTG  AATAAAG  AGGCGTGCCTTCCAGGCAGGCTCTA  TA A GTGAC  CGCCGCGGCGAGCG  TGCGCGCGTTGCA  GGTCACTGTAGCGGGAC
                           (TATA+)                            (INR-)                     (MTE-)                     
Mouse     CTTGGCGGTCTGTTTTG  AATAAGG  GGGTGTGTCCTAGAGAGGACTCTA  TA A GAGTC  GGCCGCTGCAGGCG  TGCGCGCACTGTT  TGCTGCTTTAGGTGTCT
                            (TATA+)                           (INR-)                     (MTE-)                     
    209 Human: ID3 (3399, AL021154) 
Mouse: Id3 (15903, M60523) 
 
           |-50      |-40      |-30      |-20      |-10      |1        |11       |21       |31       |41       
Human     AACCAGTGTTGGGCTAAAGGCGGACTGGCAGGGGGCAGGGAAGCTCAAA  G A TCTGGG  GTGCTGCCAGGAA  AAAGCAAATTCTG  GAAGTTAATGGTTTTGA
                                                             (INR-)                    (MTE+)                     
Mouse     ACCTCAGAGCTGTGGGTTCGAACCAGTATTGGGGGGAGGGGACCTCAGA  G A ACTTGG  GCGCCTCCA  GAAAAGGCATATT  CTTAAAAGTTAATGGTTTTAA
                                                             (INR-)                (MTE+)                         
    210 Human: IFI16 (3428, AF208043) 
Mouse: Ifi204 (15951, NM_008329) 
 
           |-50      |-40      |-30      |-20      |-10      |1        |11       |21       |31       |41       
Human     GAAAGCTAAAAACACTGGTGGGAGGAGTCTCCACATTGTTTCCTACT  CCA T TTTC  TCTGGGGCA  ATAGCAGAATAGG  AGCAAGCCAGCACTAGTCAGCTA
                                                           (INR+)                (MTE+)                           
Mouse     GGAAATCGAAAGCTATGAACAGTGCTGGGAGGAGCCCAATTTCATCT  TAA G TCTC  CCCAGGAAG  ACAGCACACTGTA    AGACAT  CCAGCCCTGCTCAAGCT
                                                           (INR-)                (MTE+)     (DPE+)                  
    211 Human: CYR61 (3491, NM_001554) 
Mouse: Cyr61 (16007, AA611841) 
 
           |-50      |-40      |-30      |-20      |-10      |1        |11       |21       |31       |41       
Human     GCGGCGCCTCCGCCGGCCCG  TATAAAA  GGCGGGCTCCGGGGCGCCTGG  GC A GACCG  CGAGCGAGAGCGCCCC  CGAGCAGCGCCCG  CGCCCTCCGCGCCTT
                               (TATA+)                        (INR+)                       (MTE+)                   
Mouse     ACGCGGCGCCTAGGCAGGCAT  TATAAAG  GGCGGGCTCCCGGCGCTGGG  GC A GACCG  TGAGCGAGAGCGCCCCA  GAGAAGCGCCTGC  AATCTCTGCGCCTC
                                (TATA+)                       (INR+)                        (MTE-)                  
    212 Human: IL6R (3570, X12830) 
Mouse: Il6ra (16194, ENSMUST00000081578) 
 
           |-50      |-40      |-30      |-20      |-10      |1        |11       |21       |31       |41       
Human     TCCTGTTTTAGGCTGGCAGCTCACAGTCCCTCTCTGGTTATTTTCAGGTC T G  TGTGTGCC  ACAGAGAGGAAGGGGGCAACCACAGTGGGAACCGCTTTCC
                                                                (INR-)                                          
Mouse     CCTTCCTGTTTCAGGCCAGCAACTTGTCTCTCTGCAATTATTTTCAGGTC T C  TGTTTACC  ACAGAAAGGAAGGAGGCAGCACAATGAGAGTCTGTTGGAA
                                                                (INR-)                                          
    213 Human: IL10 (3586, BC022315) 
Mouse: Il10 (16153, M37897) 
 
           |-50      |-40      |-30      |-20      |-10      |1        |11       |21       |31       |41       
Human     AAGGGGGACAGAGAGGTGAAGGTCTACACATCAGGGGCTTGCTCTTGCAA A A  CCAAACCA  CAAGACAGACTTGCAAAAGAAGGCATGCACAGCTCAGCAC
                                                                (INR+)                                          
Mouse     AAAGGGGGACCAAGAACAGGAGGT  CTACATT  TAGAGACTTGCTCTT  GCAC T ACC  AAAGCCACAAG  GCAGCCTTGCAGA  AAAGAGAGCTCCATCATGCCTG
                                   (TATA-)                  (INR+)                  (MTE+)                          
    214 Human: TNFRSF9 (3604, L12964) 
Mouse: Tnfrsf9 (21942, BY209098) 
 
           |-50      |-40      |-30      |-20      |-10      |1        |11       |21       |31       |41       
Human     CCGCACTCCTGTGTTTGACCTGAAGTCCTCTCGAGCTGCAGAAGCCTGAA G ACCAAGGAGTGGAAAGTTCTCCG  GCAGCCCTGAGAT  CTCAAGGTCTGTC
                                                                                         (MTE+)                 
Mouse     CACAGGTCCTGTGTTTGACCTGTGGTCTTGTGGAGCGGCAGAACCTCACC   G AGCTGCC  AAGGAAGCAGAAC  GCTCCTCGCTGCC  CTGAGATCGAAAGGTT
                                                              (INR-)                    (MTE-)                    
    215 Human: ILF2 (3608, NM_004515) 
Mouse: Ilf2 (67781, AF458249) 
 
           |-50      |-40      |-30      |-20      |-10      |1        |11       |21       |31       |41       
Human     AGCCAATCGCACCGGGT  TTTATTG  GGACACGCCGGCAAGACGCCTCT  TCA G TTGT  CTGCTACTCAGAGGA  AGGGGCGGTTGGT  GCGGCCTCCATTGTTCG
                            (TATA+)                          (INR+)                      (MTE-)                     
Mouse     AGCCTATCGCGCAGGCGTTTGTTGGGACACGCCGGCCAGACGTTTCTCAG   C CAGCCTA  GTTCTTCCCAGCGA  AGAGCGGTTGTGT  GGCCTCCATTGTTCA
                                                              (INR+)                     (MTE+)                   
    216 Human: JAK1 (3716, NM_002227) 
Mouse: Jak1 (16451, AK081552) 
 
           |-50      |-40      |-30      |-20      |-10      |1        |11       |21       |31       |41       
Human     TCTCTCCCTTTTCCAACCCCCCCGCCATCTATCACATGGCAGAGAT  AGAA T AAA  AACAGAAAAATGGCGACGGTCACGTTGTGGCGAGCCTTGCTGCGTC
                                                          (INR-)                                                
Mouse     TTTCCACCCCCCCCCCCCCTTCCCCAATCTATCACATGGCAAAGCT  AGAA T AAA  AACAGAAAAATGGCGACGGTCACGTTGTGGCGAGCCTTGCTGCGTC
                                                          (INR-)                                                
    217 Human: JUN (3725, NM_002228) 
Mouse: Jun (16476, NM_010591) 
 
           |-50      |-40      |-30      |-20      |-10      |1        |11       |21       |31       |41       
Human     GGGGTTGACTGGTAGCA  GATAAGT  GTTGAGCTCGGGCTGGATAAGGGCTC   A GAGTTGC  ACTGAGTGTGGCTGAA  GCAGCGAGGCGGG  AGTGGAGGTGCGC
                            (TATA+)                             (INR-)                       (MTE+)                 
Mouse     GGGATTGACTGGTAGCA  GATAAGT  GTTGAGCTCAGGCTGGATAAGGACTC   A GAGTTGC  ACTGAGTGTGGCAG  AGACAG  CCTGGCAGGAGAGCGCTCAGGC
                            (TATA+)                             (INR-)                 (DPE+)                       
    218 Human: KCNA2 (3737, NM_004974) 
Mouse: Kcna2 (16490, NM_008417) 
 
           |-50      |-40      |-30      |-20      |-10      |1        |11       |21       |31       |41       
Human     GGCTCCCCCTCTCGCTGCTGCCAAGCCGCCTCGCAGCTAGGCAGGCGGAG C GT  TTATTCTG  TGCTCCCTGCCGAGG  AGATGT  GAGGGATTTTCTCTTGGG
                                                                 (INR+)                  (DPE+)                   
Mouse     GGCTCCCCCTCTATGTGAAGCCAATCCGCCCCGCAGTCAGGCAGCTTGAG   C GTTTGTT  CTGTGCTTCCCTGCCG  AGATGT  GAGGGATTTTCTCTTGGGAA
                                                              (INR-)                   (DPE+)                     
    219 Human: KCNJ9 (3765, NM_004983) 
Mouse: Kcnj9 (16524, AF403130) 
 
           |-50      |-40      |-30      |-20      |-10      |1        |11       |21       |31       |41       
Human     TGCCGTGGAAACAAGAGAGGAGCAGGGGAGCCTGGGAAGTAGGGATGAC  A C AGATAG  CAAGTCCTAGTCAG  AGCTGCCGCTACA  TTTAGGAGAAACAGCG
                                                             (INR+)                     (MTE-)                    
Mouse     TGCCATAGAAACGAGAGAGGAGCAGGGGAACCTGGGAAGTGGGGATGAC  A C AGATAC  CAAGTCCTAGTCTG  AGCTGCCGTTACA  TTCAGGAGAAACAGCA
                                                             (INR+)                     (MTE-)                    
    220 Human: KCNJ10 (3766, U52155) 
Mouse: Kcnj10 (16513, AK048864) 
 
           |-50      |-40      |-30      |-20      |-10      |1        |11       |21       |31       |41       
Human     CCAGCTCTGCCCCCGGCCGGCCCGACCCCGGCCCCGGCCCCCGGACAAGC C CT  TATCTGAT  CCCA  GCTCCGGGTTTAA  GAGTCCTGGCCCGGCCCGTCGC
                                                                 (INR-)           (MTE-)                          
Mouse     CCAACTCTGCCCCCGGCCGGCCCGACCCCGGCCCCGGCCCCCGGACAAAC C CT  TATCTGAT  TCCA  GCTCCGGGTTTAA  GAGTCTTGGCCCTGCCTGTCGC
                                                                 (INR-)           (MTE-)                          
    221 Human: KCNJ10 (3766, AF482709) 
Mouse: Kcnj10 (16513, AK048864) 
 
           |-50      |-40      |-30      |-20      |-10      |1        |11       |21       |31       |41       
Human     CCTGCCCGGCCCCCCT  CCGGGCCC  CTCCATTCACACAAAGTTTGGCTCCG G CGCTGCGGAGGGAG  GGGGCGGCCCGGC  CCGGCCCAGCTCTGCCCCCGGC
                            (BRE+)                                                (MTE+)                          
Mouse     GCCCTCCCGAC  CCCCGCCG  GGCCCCTCCATTCACACAAAGTTTGGCTTCG   G CACTGCA  GAGGGAG  GGGGCGGCCCGGC  CCGGCCCAACTCTGCCCCCGGC
                       (BRE+)                                   (INR+)              (MTE+)                          
    222 Human: KCNK1 (3775, U90065) 
Mouse: Kcnk1 (16525, BC003729) 
 
           |-50      |-40      |-30      |-20      |-10      |1        |11       |21       |31       |41       
Human     GGGCCGCGCTCCGGCCGGTCTGCGGCGTTGGCCTTGGCGGCGGCGGTGGA G   AAGATGCT  GCAGTCCCTGGCCG  GCAGCTCGTGCGT  GCGCCTGGTGGAGC
                                                               (INR-)                     (MTE+)                  
Mouse     CGAAACCAGGAAAG  GGAGCGGC  GGCGGGAGGCGCGGAGCGCGGGCGCCGG G   AAGATGCT  GCAGTCCCTGGCCG  GCAGCTCGTGCGT  GCGCCTGGTGGAGC
                          (BRE-)                                 (INR-)                     (MTE+)                  
    223 Human: KCNN3 (3782, NM_002249) 
Mouse: Kcnn3 (140493, NM_080466) 
 
           |-50      |-40      |-30      |-20      |-10      |1        |11       |21       |31       |41       
Human     GTGGGCTTGGCTTACAATGTAACAGTGGCAGGAGGAGGCGAGCGAAG  CTA T TGAG  CCAGCGAGGAGT  GAAGCTGAGCCTG  GCCTCACACGCTCCTAGAGG
                                                           (INR-)                   (MTE+)                        
Mouse     GTGGGCTT  GGCTTGC  C  ATATAA  CAGTGTCAGGAGGAGGAGAGCGAAG  CTA T TGAG  CCAGCGAGGAGT  GAAGCTGAGTCTG  CTTCATACACTCCTAGAGGA
                    (BRE-)  (TATA+)                            (INR-)                   (MTE+)                        
    224 Human: LAD1 (3898, BC071890) 
Mouse: Lad1 (16763, AK083526) 
 
           |-50      |-40      |-30      |-20      |-10      |1        |11       |21       |31       |41       
Human     AAGGGGAG  GGGCGCCA  GGTGAGGCGGCGGCCGGCGGGGCGCGGGCGGCCA C GCGGGGCTCCTGCAGCATGGCTG  TCAGCAGGAAGGA  CTGGTCCGCGCTG
                    (BRE+)                                                                 (MTE+)                 
Mouse     GGGGAGGGGCCGCAGG  TGAGCGGC  GGAGCAGGGAGCTCGGCACGCGGCGG C CGTGGGGCTCGGCAGCATGTCGG  TCAGCAGAAAGGA  CTGGTCCGCGCTG
                            (BRE-)                                                         (MTE+)                 
    225 Human: LAMB3 (3914, L25541) 
Mouse: Lamb3 (16780, BY023161) 
 
           |-50      |-40      |-30      |-20      |-10      |1        |11       |21       |31       |41       
Human     ACACAGCAAGGTATGTTACCTGTCTTTACCTGCCTGGTGCGGGCCGGATG C GGGAGGAGGACTGT  ATCTCTGGATGCC  TGGGGCCTGGTTTCAGGGCCTG
                                                                                (MTE-)                          
Mouse     GCTCAGGGTGAGGTAGGTTTTCCGCCTTACCTGTCCTGTGGGGGTGGATG G AGCAGGTCTGGGGC  CAAGGCCGATTGC  AGGGCCCATGCAGCACTCCTCT
                                                                                (MTE-)                          
    226 Human: LAMB3 (3914, U17744) 
Mouse: Lamb3 (16780, NM_008484) 
 
           |-50      |-40      |-30      |-20      |-10      |1        |11       |21       |31       |41       
Human     CCCTTGCAGCCCCCACAGGCAGGTGGGCATTGTGGAGCTCACTACAA  GAA C TCTG  GGACGGACCGA  CCAACCCACTTGC  CCAGTCCCGTCCTGGGAGGTG
                                                           (INR+)                  (MTE+)                         
Mouse     GCTGTCTGTTTCCAGCACCTTCCAGCCCTCTAGACACCTAAGGGCAGGAA G CCTCCAAGACCAACCCACTTGCCTAGTCC  TGTCCT  GAGGGTGGGGGTGC
                                                                                           (DPE-)               
    227 Human: LAMB3 (3914, NM_000228) 
Mouse: Lamb3 (16780, BY023161) 
 
           |-50      |-40      |-30      |-20      |-10      |1        |11       |21       |31       |41       
Human     GGGTGTGACGGCTGCCAGATTCCTGAGACCCGCCCTGCGGTGGGG  CTACA C CC  AGCCAGGGAGTCTCCAG  AGGTGAGGCTGTT  GTTTAAAAACCTGGAGC
                                                         (INR+)                        (MTE-)                     
Mouse     ATGGGCGTGGCAGCTGCCAGATTCCTGAGGCCGCCAGCAGTGGGG  CTACA C CC  AGCCAGGGAGTCTCCAG  AGGTGAGGCTGTT  GTTTAAAACTGTGGAGC
                                                         (INR+)                        (MTE-)                     
    228 Human: LAMC1 (3915, M55217) 
Mouse: Lamc1 (226519, J03749) 
 
           |-50      |-40      |-30      |-20      |-10      |1        |11       |21       |31       |41       
Human     CCCACCCCACCCTGCCGACCCCACCCCCTGCTCCTTCCTCCCCGGGGGCG C   GCACTCGG  GCACGCGCTCGGA  AGTCGG  GGGTCGGCGCGGAGTGCAGGCT
                                                               (INR+)                (DPE+)                       
Mouse     ATCCCACCCCGCCGGGCAGACCCCACCCCCTTCTCCTTCCTCCTGGGGC  G C GCTCTC  GAGTGCGCGCTCGGA  AGTCGG  GGGTCGGCGCACAGTGCAGGCT
                                                             (INR+)                  (DPE+)                       
    229 Human: LAMC2 (3918, U31178) 
Mouse: Lamc2 (16782, BY074363) 
 
           |-50      |-40      |-30      |-20      |-10      |1        |11       |21       |31       |41       
Human     AGTCAGGTAGAAGAGTC  GATAAAA  CCACCTGATCAAGGAAAAGGAAG  GCA C AGCG  GAGCGCAGAGTGAGAACCA  CCAACCGAGGCGC  CGGGCAGCGACCC
                            (TATA+)                          (INR+)                          (MTE+)                 
Mouse     AGTCAGGGAGAAGAGTC  GATAAAA  CACCTGATCGAGGGAAAAGGAAG  GCA C AGCA  GAGCGAAGAC  TCGGCGCCCGGCA  GGCACCCGCAGCGGCGGAGCGC
                            (TATA+)                          (INR+)                 (MTE+)                          
    230 Human: STMN1 (3925, M31303) 
Mouse: Stmn1 (16765, AK014108) 
 
           |-50      |-40      |-30      |-20      |-10      |1        |11       |21       |31       |41       
Human     CGCGGAGGTCACGTGCCTCTGTTTGGCGCTTTTGTGCGCGCCCG  GGTCTG T T  GGTGCTCAGAGTGTGG  TCAGGCGGCTCGG  ACTGAGCAGGTGGGTGCGG
                                                        (INR-)                       (MTE-)                       
Mouse     GGTCTGGGTCACGTGCCGCTGTTTGGCGCTTTTGTGCGCGCCCA  GGTCTG T T  GGTGCTCAGAGTGTGG  CCAGGCGGCTCGG  ACCGAGCAGGTGGGTGCAG
                                                        (INR-)                       (MTE-)                       
    231 Human: LBR (3930, NM_002296) 
Mouse: Lbr (98386, BC042522) 
 
           |-50      |-40      |-30      |-20      |-10      |1        |11       |21       |31       |41       
Human     GCGCCGCGA  GGCGGGGC  CGGGGAGGCGCGCGCCCGGCTTGCGGGACTGCG G GGCGCGAGAGCGCGGCCGG  GTAGCGTCGCGCG  CGTGGATCTGCCGCCGG
                     (BRE-)                                                            (MTE+)                     
Mouse     GAGGGACCAATGGGGAGCTCAGGGCAGCCTCGCTAGGGGCGGGACCTCGG G GGCGCGAGAGCGCGGCCGGCT  GTGTCGCGCGCGG  AGTCGCCGCTGGCTG
                                                                                       (MTE-)                   
    232 Human: LCK (3932, NM_005356) 
Mouse: Lck (16818, AK088001) 
 
           |-50      |-40      |-30      |-20      |-10      |1        |11       |21       |31       |41       
Human     GCGGAGCCCTCCGGAGGAGGCAGGAAGTCAGGGTGGGACGTGGGCGCGGG G AGACAGGTGGTGGCTACGACGGC  GAAGGGAGCTGAG  ACTGTCCAGGCAG
                                                                                         (MTE-)                 
Mouse     GCTGCAGAGCCCTCAGGAGACAGGAAGTCAGGGTGGAACGTGGGCGCGAG G AGACAGGTGGTGACTACGACGGC  GAGGGGAGCTGAC  ACCGGGTCCAGGC
                                                                                         (MTE-)                 
    233 Human: LGALS8 (3964, NM_006499) 
Mouse: Lgals8 (56048, AA062254) 
 
           |-50      |-40      |-30      |-20      |-10      |1        |11       |21       |31       |41       
Human     CGCGCGAGCCGGGAAGTGAACGAGGACTGACTCCTGTCGCTTCCCGTAGC C GCCCACGGACGCC  AGAGCCGGGAACC  CTGACGGCACTTAGCTGCTGACA
                                                                               (MTE+)                           
Mouse     GTCGTTCTCGCGGAAACG  GCCGCGGG  CTGACCCCGCCCTCTGCCTGTGGC G GCTCCGGGAACCGGACGGCGTCCGTCAGGGGACACAAGTCGGAGCAAAG
                              (BRE-)                                                                            
    234 Human: LGALS8 (3964, CR606435) 
Mouse: Lgals8 (56048, AA062254) 
 
           |-50      |-40      |-30      |-20      |-10      |1        |11       |21       |31       |41       
Human     GCCGAGTCATTTGACTGCAGT  GTTAAAC  ATTGCAAAGCGCAAGT  CATGTG A C  TTCCTTTGACCGTACGTGAAACTTAAGTGATGGCTGCTTGTGATGCAT
                                (TATA+)                   (INR-)                                                  
Mouse     GCTGCGTCATTCTATCCCAAC  GTTAAAC  ACGGGGAAACGCAATTTCTCTG   G GTTTGGG  AATCGTCCTAAACCTA  AGCCACGGCTTGT  TGTGACTGACGGT
                                (TATA+)                         (INR-)                       (MTE-)                 
    235 Human: LMNA (4000, L12399) 
Mouse: Lmna (16905, AK004619) 
 
           |-50      |-40      |-30      |-20      |-10      |1        |11       |21       |31       |41       
Human     CCCTTTCCGGGACCCCTGCCCCGCGGGCAGCGCTGCCAACCTGCCGGCCA T GGAGACCCCGTCCCAGCGGCGCG  CCACCCGCAGCGG  GGCGCAGGCCAGC
                                                                                         (MTE+)                 
Mouse     CCCTTCTCGGGACCCCTGCCCAGCGGGCAGCACTGTCACCCTGCCGGCCA T GGAGACCCCGTCACAGCGGCGCG  CCACCCGCAGTGG  GGCGCAGGCCAGC
                                                                                         (MTE+)                 
    236 Human: LMX1A (4009, AY078391) 
Mouse: Lmx1a (110648, AK044944) 
 
           |-50      |-40      |-30      |-20      |-10      |1        |11       |21       |31       |41       
Human     AAACGCAGTTGG  GGGGCGGA  GGC  CTAAGTA  CATAACGTGTTGACTT  CAAG T GAA  ATCAGATCAGCC  AGAGCAGTTCGCT  G  TGACTG  ATCTCTCCTCCCAC
                        (BRE-)      (TATA-)                   (INR-)                   (MTE+)      (DPE-)               
Mouse     GAACGCAGTTAG  GGGGCGGA  GGC  CTTAGCA  CATAACGCGTTGACTT  CAAG T GAA  ATCAGAGCCGTC  CGAGCAGTTCACG  GTGGCTCTTCTCTCTCCTCCC
                        (BRE-)      (TATA-)                   (INR-)                   (MTE+)                         
    237 Human: LOR (4014, M94077) 
Mouse: Lor (16939, M34398) 
 
           |-50      |-40      |-30      |-20      |-10      |1        |11       |21       |31       |41       
Human     GGGAGAGCTCTATA  TATAACC  TCAGGAGATCAGTGCTCCTCACATTGCCA   G CATCTTC  TCTCCTCACTCACCCT  TCCTGGTGCTTTG  GGTAAGTGTGGTT
                         (TATA+)                                (INR+)                       (MTE-)                 
Mouse     GCAGGACAAGAGTATAAAACACAGGAGCACCAGTGTCCCTCACATCAGCA   T CACCTCC  TTCCCTCACTCATCTT  CCCTGGTGCTTCA  GGTAAGTGTGGGC
                                                              (INR+)                       (MTE-)                 
    238 Human: LY9 (4063, AF244129) 
Mouse: Ly9 (17085, AF244130) 
 
           |-50      |-40      |-30      |-20      |-10      |1        |11       |21       |31       |41       
Human     AAGCTGTGACACGTGCGGAAGCTGTGGTAAGTGCATCCTCCTTCAGTC  TC A GTTCT  GAAAATAGATCATCATGGTGGCACCAAAGAGTCACACAGATGAC
                                                            (INR+)                                              
Mouse     TGCACTCACATACTTCAGGAAGTTGTGGTAAGTGCATCTCCTTT  AATTTG A A  ATCTGGAAGTACACCATCATGGCG  GATCTA  AAGAGATATTGGTGTGAC
                                                        (INR-)                           (DPE-)                   
    239 Human: MAGOH (4116, AF067173) 
Mouse: Magoh (17149, AF007862) 
 
           |-50      |-40      |-30      |-20      |-10      |1        |11       |21       |31       |41       
Human     AGAAGGAACCCCT  CCACGCAG  CGACGACGGGACCTGACGTCACTTCCGCG C GCCGCCGCAAACGTCAGTGTCGGGCGC  AGACGG  CGGCAGTGCGGCTTGC
                         (BRE+)                                                            (DPE+)                 
Mouse     CCGGAGAGTGAGAAACTCTTAGTGTCGCGGTGGCAGACGTCATTTCCGCG C GCCACCATTACGCCAGTATAAGACGT  AGACCG  CAGGCCGTGGTAGTGTC
                                                                                        (DPE+)                  
    240 Human: MCL1 (4170, NM_021960) 
Mouse: Mcl1 (17210, BG072263) 
 
           |-50      |-40      |-30      |-20      |-10      |1        |11       |21       |31       |41       
Human     ACCCCGTAGGACTGG  CCGCCCT  AAAACCGTGATAAAGGAGCTGCTCG  CCA C TTCT  CACTTCCGCTTCCTTCCAGTAAGG  AGTCGG  GGTCTTCCCCAGTTT
                          (TATA-)                            (INR+)                           (DPE+)                
Mouse     CGCCTGCGTCAGCACGGCCCTAAGGCGGCGGCAGGGAACGGCCTTCC  TCA C TCCT  GACTTCCGCCTGCCTCC  GGTCTG  GAGTCGCGGCCTTCCCCGCTCC
                                                           (INR+)                    (DPE-)                       
    241 Human: MDM4 (4194, NM_002393) 
Mouse: Mdm4 (17248, NM_008575) 
 
           |-50      |-40      |-30      |-20      |-10      |1        |11       |21       |31       |41       
Human     CCTCCCATTGCTCCATCTATGGTTTCCGGAGGCCTCACCGGAAGCCCT  CG T GTGAG  GCCGTGTGGGAGGCCGGA  AGTTGC  GGCTTCATTACTCGCCATTT
                                                            (INR-)                     (DPE+)                     
Mouse     GTCCTCCAGGGTCCGCTCTATGGTTCCCCCGGCCTCCCCGGAAGCTCTTG C GAACGCTGGGTTTGAGAGGCCGG  AAGTGGTGCTGCC  GTTGCTCGCAGTT
                                                                                         (MTE-)                 
    242 Human: MEF2D (4209, L16794) 
Mouse: Mef2d (17261, BC011070) 
 
           |-50      |-40      |-30      |-20      |-10      |1        |11       |21       |31       |41       
Human     CGCGGACGCCGGCCT  CATTTAT  TATTCTCCCCGCCCGGAGCTGCGGCTTC C C  GGTGTTGA  AGATCCCCCGGACCAGGGGCGAGGGCTACCCGCTCTTTGC
                          (TATA-)                                 (INR-)                                          
Mouse     GGCCGCGTGGACG  CGGCGCCT  TCGCTGTTTCCCGTCGGAGCTGCGGCTTC G CG  TAACCGAG  GATTCGGC  GGACCGGGCCGAG  GCTCCGGGCGCCGTGACA
                         (BRE+)                                    (INR-)               (MTE+)                      
    243 Human: MFAP2 (4237, BC015039) 
Mouse: Mfap2 (17150, ENSMUST00000071977) 
 
           |-50      |-40      |-30      |-20      |-10      |1        |11       |21       |31       |41       
Human     GCGGGCGCCAGCCCCGTCGGGGGCCCGGAGGGGGACTCGGAGCGGGC  CAA G GGGC  GGCTCCGGCGGGCGGACTC  GGAGCGGGCGGCG  GAGTGACCCGGAC
                                                           (INR-)                          (MTE+)                 
Mouse     GGGCTAGGGGCG  GGCGTGGG  CCTTGGGGGCCGTAGGGGGGCTCTGGCTGC G GGGCGGCACTTTCAGCCGCACT  AGAGCACAGCGCA  GAGGCCCCAACAGG
                        (BRE-)                                                            (MTE+)                  
    244 Human: MGST3 (4259, NM_004528) 
Mouse: Mgst3 (66447, AK003246) 
 
           |-50      |-40      |-30      |-20      |-10      |1        |11       |21       |31       |41       
Human     TTGGGGGCGTGG  CCGCGCGC  TGGTCGGCTGGGGCGGGGCCTGCTTCTGGC C T  CATCTAGC  CCCGCCCCAGGCG  AGGGCGCCGCACC  CACACCGCGCTGCG
                        (BRE+)                                    (INR-)                    (MTE+)                  
Mouse     CCCGAGGGCGTGG  CCGCGCGG  GGCGGGGCGGAGTGGGGCGGGTCCTGCGG G A  CAGCTAGA  GCCGCACCTAGGCA  CTGCTGTGCTTCT  CAGGTCTGTACCA
                         (BRE+)                                   (INR-)                     (MTE-)                 
    245 Human: MOV10 (4343, NM_020963) 
Mouse: Mov10 (17454, AK004542) 
 
           |-50      |-40      |-30      |-20      |-10      |1        |11       |21       |31       |41       
Human     CCTGCCCTTTGCTCTCCTGCTTTTCACTGAGTCCCGAGGCTCTGGAGGGT G GGCGTGAGGGTAGCCCT  GGCTGAGGCTCGG  A  GGTCCT  CCCGGGCTGTTC
                                                                                   (MTE-)      (DPE-)             
Mouse     GCTCTGCCCTTTGCTCTG  CAGCTTT  TCTATGTCCCGAGGCTCTGG  AGAGT G GG  CGTGGGAGTAGCC  CTAGCTGAGGCTC  CAA  GGTCCT  CCGGGCTGTTTA
                             (TATA-)                       (INR-)                    (MTE+)        (DPE-)             
    246 Human: MOV10 (4343, NM_020963) 
Mouse: Mov10 (17454, NM_008619) 
 
           |-50      |-40      |-30      |-20      |-10      |1        |11       |21       |31       |41       
Human     CTCATCTCAGGGCCGCCAACTTCCAGCTGCAGCGGCGACTTTCAGTT  TCA T TTCC  ACGGACCCTCCTGCCTGGGCCGCAGCCGCCGCCGCGATGCCCAGT
                                                           (INR+)                                               
Mouse     CTCACCCCAGACCAGCCAAC  TCTAAAC  CGCGGCGGCGACTTTCAGTT  TCA T TTCC  GCGGACCCTCCTGCCTGGGCCGCAGCCGCCGCCGCCGCGATGCCT
                               (TATA+)                       (INR+)                                               
    247 Human: MPL (4352, U68159) 
Mouse: Mpl (17480, AK041166) 
 
           |-50      |-40      |-30      |-20      |-10      |1        |11       |21       |31       |41       
Human     CTGACAGGAACCTGAGGGGCTGGCCTGGGAGGGGATTGGGGCCCAGCTTC C T  GAAGGGAG  GATGGGCTAAGGCAGGCACACAGTGGCGGAGAAGATGCCC
                                                                (INR-)                                          
Mouse     CTGACAGGAACCTGAGGGGCTGGCCCCGGGGGGGATTGGGGCCCAGCTTC C T  GAAGGGAG  GATGGGCTAAGGCAGGCACACAGTGCCGGAGAAGATGCCC
                                                                (INR-)                                          
    248 Human: MPZ (4359, L24894) 
Mouse: Mpz (17528, NM_008623) 
 
           |-50      |-40      |-30      |-20      |-10      |1        |11       |21       |31       |41       
Human     CACTTTCTCAACCCCACAGATGCTCCGGGCCCCTGCCCCTGCC  CCAGCTA T   GGCTCCTGGGGCTCCCTCAT  CCAGCCCCAGCCC  TATCCTGGCTGTGCTG
                                                       (INR+)                           (MTE+)                    
Mouse     CCGCTCTCT  CCACCCCA  CAGACACTCTGGGCCTTTGCCCTACC  CCAGCTA T   GGCTCCCGGGGCTCCCTCCT  CCAGCCCCAGCCC  TATCCTGGCTGCCCTG
                     (BRE+)                              (INR+)                           (MTE+)                    
    249 Human: MPZ (4359, D14583) 
Mouse: Mpz (17528, NM_008623) 
 
           |-50      |-40      |-30      |-20      |-10      |1        |11       |21       |31       |41       
Human     ATGCTGGGGCCCTAGGGGAT  TTTAAGC  AGGTTCCAGGAACCCCCCGT  TCA G TTCC  TGGTCCCCCACTTTCTCAACCCCAC  AGATGC  TCCGGGCCCCTGCC
                               (TATA+)                       (INR+)                            (DPE+)               
Mouse     CATGCTGGGGATCCAGGGGAT  TTTAAGC  AGGTTCCAGAAAACACAGC  TCA G TTCC  TTGTCCCCCGCTCTCTCCACCCCAC  AGACAC  TCTGGGCCTTTGCC
                                (TATA+)                      (INR+)                            (DPE+)               
    250 Human: MTF1 (4520, BC014454) 
Mouse: Mtf1 (17764, AK030284) 
 
           |-50      |-40      |-30      |-20      |-10      |1        |11       |21       |31       |41       
Human     CACCTTCCTCCGGAAAGAGGAAGCGGAAGTGACGTTAGGGGAAGGTGGGG   G CAATCAT  GGTGCCGCTGGGGAGGGGAGAAGCTGCTGCTGCCGCCGTTGC
                                                              (INR+)                                            
Mouse     CTCCTTCCTCCGGAAAGCGGAAGCGGAAGTGACGCTAGGGACAGGTGGGG   G CAATCAT  GGTGCCGCTTGGGAGGGGAGAAGCTGCTGCTGCCGCCGTTGC
                                                              (INR+)                                            
    251 Human: MTHFR (4524, AF257484) 
Mouse: Mthfr (17769, AF404272) 
 
           |-50      |-40      |-30      |-20      |-10      |1        |11       |21       |31       |41       
Human     GAGCTCAAGCCTCTTCCTTTGTCGCAGCTCCGCCCAGTTGAACACACCCG C TGGGGAAGGTGCCTCTGTTCCCTCCCCACGCACTCTGGGCCTGAGCTGA
                                                                                                              
Mouse     GCTTCAGCTCGGGGCTCTTTGTCACAGCTCCGCCCAGTTGGGCACACCCT C CAGGAAAGGTTCCTTCCCCACTCACTCTG  GGTCTG  AGCAGATCGGGATG
                                                                                           (DPE-)               
    252 Human: MTHFR (4524, AY046565) 
Mouse: Mthfr (17769, AF404272) 
 
           |-50      |-40      |-30      |-20      |-10      |1        |11       |21       |31       |41       
Human     AGTGGCAGAGGCTTATTTTGAGAGAGTGGCAGCACCTGGCCCTTTGGCGC   T CAGTGAA  TGTTGGCTATCACCGTGTGCCAAACTCTGGGGATACCCCAGG
                                                              (INR-)                                            
Mouse     TGGCAGCGAGGCATTTTGAGATTGGTGTGGAGGTCTTGGCAGTTTTGAGC   C CAGTGAG  TACTGCCTGTTTTACTACTCTGCCAGGCACTGGCGATGCCAC
                                                              (INR-)                                            
    253 Human: MTHFR (4524, AY046561) 
Mouse: Mthfr (17769, AF404270) 
 
           |-50      |-40      |-30      |-20      |-10      |1        |11       |21       |31       |41       
Human     CCGGGGCACTGCGGTCCC  TGGCGCCC  ACTGCGTCCCGCTGCGCACGGGGG T CCGCCGGGACCTTTCTGGGA  GTCGTAGGCTTAG  TATCCCAGTGCTTGGC
                              (BRE+)                                                    (MTE-)                    
Mouse     CCATTAGTTT  CAACGCCC  AGGTGTCCATAGCATCCTGCAGTTCACATG  CA T CTATC  ACAACTCTTCCGGGA  GTGGTGGGCTCAG  TGTTTGTCAGTCTTAT
                      (BRE+)                                  (INR-)                      (MTE-)                    
    254 Human: MTR (4548, NM_000254) 
Mouse: Mtr (238505, AK037599) 
 
           |-50      |-40      |-30      |-20      |-10      |1        |11       |21       |31       |41       
Human     GGCAGACCAATCACAGGCCGGAAGGGGCGGGACCTCCCACGCCGAGGAT  A G ATTGAG  CGCAGAAC  TAACCGCGCTCTG  AAA  GGTTCT  AAATGTCTGCGGG
                                                             (INR-)               (MTE-)        (DPE+)              
Mouse     GAGGGCCAATCACATGCCGGGAAGGGGGCGGGCCCTCCTGCCGGAGGCT  A G ATTGAG  CGGAGAG  CTAACGGCGCAGT  GGCGGCGGCGGCCGTCTCTACGT
                                                             (INR-)              (MTE+)                           
    255 Human: MTX1 (4580, NM_002455) 
Mouse: Mtx1 (17827, AK086135) 
 
           |-50      |-40      |-30      |-20      |-10      |1        |11       |21       |31       |41       
Human     CCCGTCACCTAAGCGACAGCCTGCGAGCCTCTCTTCCCCTCCCCCACCCA A GCCCCAGCCCGGCCTCCGC  TCCGGCCGCCGCC  ACCGCCCCTGTTTTGTT
                                                                                     (MTE-)                     
Mouse     CCGTTACGAAGGCAACTGCCCGCGAGCCGCACCTCCCCTTCCC  CCACTCA G   GCCCCAGCCGGGCC  TCAGCTCGGGCGC  CGCCACCGCCCCTGTTTTGTTT
                                                       (INR+)                     (MTE+)                          
    256 Human: MTX1 (4580, NM_002455) 
Mouse: Mtx1 (17827, AK086135) 
 
           |-50      |-40      |-30      |-20      |-10      |1        |11       |21       |31       |41       
Human     GCGGTGGCGG  GGGGCGGG  CCCAGGCAGGGGAGGGCAGAAGCACACAAG  GA A GTGTT  TCCGGGACAGAGGGTGGGCA  AGATGG  CGGCGCCCATGGAGCTGT
                      (BRE-)                                  (INR-)                       (DPE+)                   
Mouse     GCGGGGACCA  GGGGCGGT  TCCTGGCGAGAGAAGGCCGACGCATACGAG  AA G GTGCT  TCCGGGTC  AGAGAGCGTACA  A  GATGG  CGGCGCCCATGGAGCTGT
                      (BRE-)                                  (INR-)               (MTE+)    (DPE+)                   
    257 Human: MUC1 (4582, M35093) 
Mouse: Muc1 (17829, M64928) 
 
           |-50      |-40      |-30      |-20      |-10      |1        |11       |21       |31       |41       
Human     GCAGCCAGCGCCTGCCTGAATCTGTTCTGCCCCCTCCCCACCCATTTCAC C ACCACCATGACACCGGGCACCCAGTCTCCTTTCTTCCTGCTGCTGCTCC
                                                                                                              
Mouse     ACGGAGCGCCAGCCTTGAG  TTTGTTT  TCTAGCCCCTTCCCGCCTGTTCAC C ACCACCATGACCCCGGG  CATTCGGGCTCCT  TTCTTCCTGCTGCTACTTC
                              (TATA-)                                                (MTE-)                       
    258 Human: MUC1 (4582, Z17325) 
Mouse: Muc1 (17829, M64928) 
 
           |-50      |-40      |-30      |-20      |-10      |1        |11       |21       |31       |41       
Human     GGGCGGGCGG  GGAGTGGG  GGG  ACCGGTA  TAAAGCGGTAGGCGCCTGTGCC C GCTCCACCTCTCAAGCAG  CCAGCGCCTGCCT  GAATCTGTTCTGCCCCCT
                      (BRE-)      (TATA-)                                               (MTE+)                      
Mouse     CTGTGCCTAG  GGCGGGAG  GGGG  ACTGGTA  TAAAGCAGCAGGCACCAGC  CC T GTTCC  ACCTCACACAC  GGAGCGCCAGCCT  TGAGTTTGTTTTCTAGCCCC
                      (BRE-)       (TATA-)                      (INR+)                  (MTE+)                        
    259 Human: MUTYH (4595, U63329) 
Mouse: Mutyh (70603, AK077546) 
 
           |-50      |-40      |-30      |-20      |-10      |1        |11       |21       |31       |41       
Human     CGCTCCAGGCTTTTGCAGCCGGAGCCGCGGTGTACAACGGAACTT  GTAGT C TC  CTCGTGGCTAGTT  CAGGCGGAAGGAG  CAGTCCTCTGAAGCTTGAGGA
                                                         (INR+)                    (MTE+)                         
Mouse     CAACAGTTCGGGTTTTGCAGTTGCACGCGCTGCACACTGGGAGTT  GTAGT C TA  AGATAGCTGAGGCAAGA  AGAACTACAGGCC  TGTCTCCGAGCTACCGG
                                                         (INR+)                        (MTE+)                     
    260 Human: MYOC (4653, Z97171) 
Mouse: Myoc (17926, AF049794) 
 
           |-50      |-40      |-30      |-20      |-10      |1        |11       |21       |31       |41       
Human     TGAAGGGCTGGCTCCCCAGTATA  TATAAAC  CTCTCTGGAGCTCGGGCATG A GCCAGCAAGGCCAC  CCATCCAGGCACC  TCTCAGCACAGCAGAGCTTTCC
                                  (TATA+)                                         (MTE+)                          
Mouse     CACAGGGCTGGGTCCCCAGGATA  TATAAAT  GTCTTTGGACTTCAGGCTTG A GCCAGCAGGGCCAC  CCATCCAGACACC  TTGCAGGAGAACTTTCCAGAAG
                                  (TATA+)                                         (MTE+)                          
    261 Human: NBL1 (4681, NM_005380) 
Mouse: Nbl1 (17965, CB194227) 
 
           |-50      |-40      |-30      |-20      |-10      |1        |11       |21       |31       |41       
Human     CGCCGCGC  GCCCGCCC  GGGGCCGCAGACAGCGCGCAGCGCAGCCCAGCCG A GCGTCGCGGGGCC  GCCCCCCGCCCTG  CCGGCCGCCTCGCCGAGCCTCCT
                    (BRE+)                                                       (MTE-)                           
Mouse     CGCCTCGC  GCCCGCCC  GGGGCCGCAGACGGCCAGCAGCGCAGCCCAGCCG A GCCTCGCGGGGCCGCC  CCAGCCCGAGGAC  CCCGGGCCTGCGACCCCCGC
                    (BRE+)                                                          (MTE+)                        
    262 Human: NDUFS2 (4720, BC008868) 
Mouse: Ndufs2 (226646, AK078474) 
 
           |-50      |-40      |-30      |-20      |-10      |1        |11       |21       |31       |41       
Human     ACCCCACAATCAGCCCTCAGCTGTCTCTTTCCCTTTTTGGTGGGACAGAG C CCCAGGAGAGGCAGAGAGTGAGGGAAAGGGCCTGGCCGGCATGCACAGA
                                                                                                              
Mouse     TCTTTTTTTTTTTTTTTTTTTTT  TTTTTTT  TTTTTTTTTGTGGGACAGAG C CCCAGGGGAGGTCAGGACTGGGGGAAAG  GGTCTG  GACAGCAGTCACAGA
                                  (TATA-)                                                   (DPE-)                
    263 Human: NDUFS2 (4720, NM_004550) 
Mouse: Ndufs2 (226646, AK078474) 
 
           |-50      |-40      |-30      |-20      |-10      |1        |11       |21       |31       |41       
Human     GTGAGAAGCCAAGAAGGA  GGCGCGCT  GGAGTTACTTCCGCCCGGTTCTCC T TCCCGCAGTCTGCAGCCGGAGTA  AGATGG  CGGCGCTGAGGGCTTTGTGC
                              (BRE-)                                                   (DPE+)                     
Mouse     AAGGGAAGCCCACCAGGG  GGCGCGCG  GGAGCGACTTCCGCCCG  GCTCTCC T   TCCTACAGTCTGCAGCCCGGTAA  AGATGG  CGGCCCTGAGGGCGCTGCGC
                              (BRE-)                     (INR+)                          (DPE+)                     
    264 Human: NDUFS5 (4725, AF047434) 
Mouse: Ndufs5 (170658, AK037635) 
 
           |-50      |-40      |-30      |-20      |-10      |1        |11       |21       |31       |41       
Human     GAGGGAAGGTCGGCTTCC  GGCGTCGC  GCTGCAAACGTCTCGCTGAGGTCA   T CCTTTAC  GGCAGGCGT  CCGCGTCGCTAGC  TAGTCGTTCTGAAGCGGCGG
                              (BRE-)                            (INR+)                (MTE-)                        
Mouse     GGCCGGTGGTTGATTTAC  GGCGTCGC  TCTGGAGATGTCGTGCTGCGGGCG   T CCTTTAC  GGCAGGCGTCTGCG  TCCTCCCGCAGCC  GGCGGTCGGGAATTG
                              (BRE-)                            (INR+)                     (MTE-)                   
    265 Human: NEK2 (4751, NM_002497) 
Mouse: Nek2 (18005, AK077627) 
 
           |-50      |-40      |-30      |-20      |-10      |1        |11       |21       |31       |41       
Human     AGCGGCCCACCGCCTGCC  AATAGAC  TGCGAGATCAGGGCCCGCGACGG  TT A AACGG  GGCCCAAGGCAGGG  GTGGCGGGTCAGT  GCTGCTCGGGGGCTTCT
                             (TATA+)                          (INR+)                     (MTE+)                     
Mouse     GCACCTTCGCTGTGGACC  AATAGAA  TGCGAGGCCGAAGCCCGCGA  CGGTT G AC  CCGGTGGCCGGTTGCTCGC  CGACTC  AGGCAGGCAGACGAGGGGCTCG
                             (TATA+)                       (INR-)                      (DPE-)                       
    266 Human: NFIA (4774, AB037860) 
Mouse: Nfia (18027, BC075702) 
 
           |-50      |-40      |-30      |-20      |-10      |1        |11       |21       |31       |41       
Human     TGGAGTGTAGGGAAACTCTAG  GCGGGGT  TAAAGTTCAGCTCATGGAGCG  G C AATAGC  GCTGGCTGGCTGGCTGC  AGTTGA  GCCGACTTGGAAATGTGAAC
                                (TATA-)                        (INR-)                    (DPE+)                     
Mouse     TGGAGTGTAGGGAAACTCTAG  GCGGGGT  TAAAGTTCAGCGCGT  GGAGCGG C   AAGAGCGCTGGCTGCTGCAGTTGAGCCG  AGTTGG  AAATGTGAACGCAAG
                                (TATA-)                  (INR-)                               (DPE+)                
    267 Human: NFYC (4802, Z74792) 
Mouse: Nfyc (18046, AK011327) 
 
           |-50      |-40      |-30      |-20      |-10      |1        |11       |21       |31       |41       
Human     CACCGCACGATACTGGGAGTCCAGGCGCCAAGGGAGGGGGAAGG  GAAAAG G G  GAAACGGTGCAAACGGCGTGGCCGC  CATCTT  GCTTGTGCCCCCGCTTC
                                                        (INR-)                            (DPE-)                  
Mouse     GCGCACTACGCGTTGCTG  GGAGTCGA  GGCGCGGAGTGGGGGGAGGGGCAG G GG  AGACGGTA  CAA  ACGGCGCGGCCGC    CATCTT  GTTTGCGCCCCCGCTCC
                              (BRE-)                               (INR-)          (MTE+)     (DPE-)                  
    268 Human: NFYC (4802, Z70024) 
Mouse: Nfyc (18046, AK011327) 
 
           |-50      |-40      |-30      |-20      |-10      |1        |11       |21       |31       |41       
Human     TCCGTTCTCCGTGACGCACACTTCCCCCTCCCCTCCGCCGCGCCTGGGCC T CT  GCATTGCC  CGACTCCGTA  GGAGCGCGGGGGC  GGCTCCTGCTCTTCCT
                                                                 (INR+)                 (MTE+)                    
Mouse     CCGGTTTTTCGTGAC  GCACGCTT  CCCCCTCCCCTCCGCCGCGCCCGGGCC T CT  GCATTGCC  CGACGGCGCA  GCAGCGCGGGGGC  GGCTGCTTTCTTCGCT
                           (BRE+)                                  (INR+)                 (MTE+)                    
    269 Human: NGFB (4803, NM_002506) 
Mouse: Ngfb (18049, K01759) 
 
           |-50      |-40      |-30      |-20      |-10      |1        |11       |21       |31       |41       
Human     GGCTAAGAATTCAAGAAGCCTGTGTGAGAGCAGCTCGGCGCTCCG  GCACA G CA  GAGAGCGCTGGGAGCCGGAGG  GGAGCGCAGCGGT  GAGTCAGGCTGCC
                                                         (INR+)                            (MTE+)                 
Mouse     GGCTAAATACAGTCAGGAAGCCT  GTTAAAG  AAGCTCTGTGCTCCAGCACG G C  AGAGAGCG  CCTGGAGCCGGAGG  GGAGCGCATCGGT  GAGTCAGGCTTCT
                                  (TATA+)                         (INR-)                     (MTE+)                 
    270 Human: NID (4811, X82245) 
Mouse: Nid1 (18073, BC052939) 
 
           |-50      |-40      |-30      |-20      |-10      |1        |11       |21       |31       |41       
Human     CGGGAGGCGGGAGGAGAGGGGGCTGCCAGGGGCGTCCGGTTACATCCCCG C CTTCCTCTGTCCTGGCCGCG  GGACCGGGTTTGC  GGGACCGCAGTTCGGG
                                                                                      (MTE-)                    
Mouse     GAGGCGGGAGGTGAGGAGGGACTGCGGAGGGGCGTAGGGTTATATCTGCG C CTCCTCCGCCTGAGCTGCTAAA  CCGCCGGGATTGT  GGGACCACAGTTGG
                                                                                        (MTE-)                  
    271 Human: NIT1 (4817, NM_005600) 
Mouse: Nit1 (27045, AK032189) 
 
           |-50      |-40      |-30      |-20      |-10      |1        |11       |21       |31       |41       
Human     CCATCTTCGCCGCCTGCTGGGTTTCCGGCCGGCGCAGCTCAGCCCCCGCC T TGCGTCACGTCCGG  CCTGCGAGTTACC  GCCCACTCGCTGCGGCGCTTCT
                                                                                (MTE-)                          
Mouse     GCTGTCCGCCATCTTCCCCACCTGCAAGCTTTCCGGCCTGCGCACTC  TCA C TGCG  TCACATCCGG  CCGGGGGGTTACC  GCCCACTTGCTCCTGCGCTCCT
                                                           (INR+)                 (MTE-)                          
    272 Human: NOTCH2 (4853, NM_024408) 
Mouse: Notch2 (18129, NM_010928) 
 
           |-50      |-40      |-30      |-20      |-10      |1        |11       |21       |31       |41       
Human     GCGGAAGGACACACGAGGCTGCTTCGTTGCACACCCGAGAAAGTTTCAG  C C AAACTT  CGGGCGGCGGCT  GAGGCGGCGGCCG  AGGAGCGGCGGACTCGGG
                                                             (INR+)                   (MTE+)                      
Mouse     GAAGGACACGCGGGCCGCCCAGCCGCGCGGCGCAGCCAGAAACTTTCAG  C C AAACTT  CGGGCGG  CGGCCTGGCTGCG  CGGAGTGCAGGGGCGGCGCAGCG
                                                             (INR+)              (MTE-)                           
    273 Human: NOTCH2 (4853, NM_024408) 
Mouse: Notch2 (18129, NM_010928) 
 
           |-50      |-40      |-30      |-20      |-10      |1        |11       |21       |31       |41       
Human     CATCTCTCATTTCTGAAAGAGATAGCATTGTAGATCTGGACGTTTCA  TCA C ATAT  TCCCAGGAAGGC  CCAGCCAAATGCA  GCTGGTAAGTTGCTGAATGT
                                                           (INR+)                   (MTE+)                        
Mouse     GCCATCTCTCATCTCTGACAGAGATAACTGTGGGTTTACATCTGTCATCA G   ACATTCCC  AGAGAAAGCAGAGCC  AGATGC  ACCATTGGCTTTGTGAGTGT
                                                               (INR+)                  (DPE+)                     
    274 Human: NPPA (4878, BC005893) 
Mouse: Nppa (230899, ENSMUST00000048850) 
 
           |-50      |-40      |-30      |-20      |-10      |1        |11       |21       |31       |41       
Human     TGCTTGGAGAGCTGGGGGGC  TATAAAA  AGAGGCGGCACTGGGCAGCTGGG A GACAGGGACAGACGTAGGCCA  AGAGAGGGGAACC  AGAGAGGAACCAGAG
                               (TATA+)                                                   (MTE+)                   
Mouse     CTTCTCAAAGAGCTGGGGGGC  TATAAAA  ACGGGAGATGCTGGCAGCTAGG A G  ACAGTGAC  GGACA  AAAGCTGAGAGAG  AGAGAGAAAGAAACCAGAGTGG
                                (TATA+)                           (INR-)            (MTE+)                          
    275 Human: NPPA (4878, AL021155) 
Mouse: Nppa (230899, ENSMUST00000048850) 
 
           |-50      |-40      |-30      |-20      |-10      |1        |11       |21       |31       |41       
Human     TTCCCCGCACGGGTACCAGCGTCGAGGAGAAAGAATCCTGAGGCACGGCG G TGAGATAACCAAGGACTCTTTTTTACTCTTCTCACACCTTTGAAGTGGG
                                                                                                              
Mouse     CCTGCATGGGTCCTGTTGCCAGG  GAGAAAG  AATCCTGAGGCGAGCGCCCA G GAAGATAACCAAGGACT  CTTTTCTGCTCCT  CTCACACCTTTGAAGTGGG
                                  (TATA+)                                            (MTE-)                       
    276 Human: NRAS (4893, X02751) 
Mouse: Nras (18176, AA114782) 
 
           |-50      |-40      |-30      |-20      |-10      |1        |11       |21       |31       |41       
Human     ACTCAGGCGCCTGG  GGCGCCGA  CTGATTACGTAGCGGGCGGGGCCG  GAAG T GCC  GCTCCTTGGTGGGGGCTGTT  CATGGCGGTTCCG  GGGTCTCCAACAT
                          (BRE-)                            (INR-)                           (MTE-)                 
Mouse     ACTGGGGCGCCTT  GGGCGCCT  AGTGATTACGTAGCGGGTGGGGCCG  GAAG T GCC  GCTCCCTGGCGGGGGCTGTT  CATGGCGGTTTCG  GGGTCTCCAACAG
                         (BRE+)                             (INR-)                           (MTE-)                 
    277 Human: NRAS (4893, AY428630) 
Mouse: Nras (18176, NM_010937) 
 
           |-50      |-40      |-30      |-20      |-10      |1        |11       |21       |31       |41       
Human     TAAAGTTTTACTGATTTTTGAGACACTAGCACCTAGCGCTTTCA  TTATTG A A  ACGTCCCGTGTGGGAGGGGCG  GGTCTG  GGTGCGGCCTGCCGCATGACT
                                                        (INR-)                        (DPE-)                      
Mouse     TAAAGTTTTACTGATTTTTGAGACACTAGCACCTAGCGCTTTCATTATT  G A AATGGG  GGCGGGGCG  GGGCTGGACTGGG  TGCGGCCGGCTGCAAGACTCT
                                                             (INR-)                (MTE-)                         
    278 Human: NRD1 (4898, NM_002525) 
Mouse: Nrd1 (230598, L27124) 
 
           |-50      |-40      |-30      |-20      |-10      |1        |11       |21       |31       |41       
Human     GTCATCGGCGGCCGGGAGGGCGGAGGGACGAGGGAAGTCGGCGAGAGGG  A G ACTGGG  TTGGGGGAGGGGTTCAGGCCTGTTCCCCGCGGCTGCGGCAGCA
                                                             (INR-)                                             
Mouse     AGCGTCATCGGCTGTCAGGAGGGTGGGATGAAGAAAGTCGGTG  TGAGCGA G   GCCGGGTTGGAGGAGGGGTTCAGGCC  TGTCCT  CCCCAGCGGCTGGGGCA
                                                       (INR-)                             (DPE-)                  
    279 Human: NTRK1 (4914, M23102) 
Mouse: Ntrk1 (18211, AK081588) 
 
           |-50      |-40      |-30      |-20      |-10      |1        |11       |21       |31       |41       
Human     GGCCGCTGGCTCCGCCCTTTCCTGGCGGCTGGGTCTTTAACACCGCCCAG C   GCACATGT  CGGGGGAGG  CCTGGCAGCTGCA  GCTGGGAGCGCACAGACGG
                                                               (INR+)                (MTE-)                       
Mouse     GGGCCGTG  GGTGCCGC  CCTCTCCTGGTGGCTAGTCTTTAACACCGCCCCG C G  CACGTGTC  GCGCGAGGCCGGGC  GGCGGCAGCTAGG  AGCGCACGGACGG
                    (BRE-)                                        (INR-)                     (MTE-)                 
    280 Human: DDR2 (4921, NM_006182) 
Mouse: Ddr2 (18214, AK198669) 
 
           |-50      |-40      |-30      |-20      |-10      |1        |11       |21       |31       |41       
Human     CTGGGATCCCTGGCGCTTTCCCAGGAATCCTCAGAATGTGAGGTT  TCTCT C AA  AGGCATCTTGCATCA  GCCTGTGGATGTA  TGCCTACCACCGGGCTCCT
                                                         (INR+)                      (MTE-)                       
Mouse     CTGGGATCCTT  GGCGCAGG  CCCAGGAATCCTCAGAATGTGAGG  TCTCTCT C   AAAGGCATCTTGCATCA  GCCTGTGGATGTA  TGCCTACCACGGGGCTCCT
                       (BRE-)                            (INR+)                        (MTE-)                       
    281 Human: OVGP1 (5016, U58001) 
Mouse: Ovgp1 (12659, AB006193) 
 
           |-50      |-40      |-30      |-20      |-10      |1        |11       |21       |31       |41       
Human     CCAGCGCTATCCACAGGGAA  GCTCCTA  TAAAGGGCTGCATCTCTGGAC  TC A CAGCT  ATCAGACCATTGAGATGTGGAAGCTGTTGCTGTGGGTTGGTGAG
                               (TATA-)                        (INR+)                                              
Mouse     CATGCTGCAGGTGAGCCAACTCC  TATTAAA  GAGTCTTGAGCTTCATCACC A   GAGCTACC  AGGCAGTTGAGATGG  GGAGGCTGCTGCT  GCTGGCTGGTGAG
                                  (TATA+)                        (INR-)                      (MTE-)                 
    282 Human: PAFAH2 (5051, D87845) 
Mouse: Pafah2 (100163, NM_133880) 
 
           |-50      |-40      |-30      |-20      |-10      |1        |11       |21       |31       |41       
Human     GACCAAAGGGCCACGGGTTCATG  GGTCGTT  TCTCATTTCCGTCGAGTTAA A   CGTCTGGG  GCTGCTTCTGA  GGAATCAGCTTGA  CTGGGTAAGTTTGATTG
                                  (TATA-)                        (INR-)                  (MTE-)                     
Mouse     TCCCAGAAGGCCACGGGCTCCTGGGCGGTGTCGGGTTTTAGTCGAGTA  AC C CTTCT  GAGGTTACTTTTCAATGATCAGCG  TGACTG  GGTAAGTTTGATTG
                                                            (INR+)                           (DPE-)               
    283 Human: PRDX1 (5052, NM_002574) 
Mouse: Prdx1 (18477, AA110455) 
 
           |-50      |-40      |-30      |-20      |-10      |1        |11       |21       |31       |41       
Human     TCGCGAGATCCCTACTGGC  TATAAAG  GCAGCGCCCCGGAGAGCTCTTGCG C GTCTTGTTCTTGCCTGGTGTCGGTGGTTAGTTTCTGCGACTTGTGTTGG
                              (TATA+)                                                                           
Mouse     CGCGAGAACTTTGTAGGC  TATAAAA  GCAGCTCCCCGCGCGCGCTGTCGGG C GA  CCTCTTCC  TGCGT  TCTCACGGCTCTT  TCTGTTTGTGAGACCTGTGGC
                             (TATA+)                               (INR+)            (MTE-)                         
    284 Human: PDC (5132, AF076465) 
Mouse: Pdc (20028, AK020798) 
 
           |-50      |-40      |-30      |-20      |-10      |1        |11       |21       |31       |41       
Human     TCATCAATATCTGGAGAT  TTTAAAT  CTGAGCTTAAACCTATTGAAGTTCA A GA  CGAGTTCA  GTAGACAGGGATT  CTCACCCACTCAA  CAAGGACACCAGG
                             (TATA+)                               (INR+)                    (MTE-)                 
Mouse     CATCAATATCCAGGGAT  CTTAAAT  CTGAGCTTAACACTGGTCCAAGTTCA A A  GGAGTTCA  GTGG  ACAGCGATTCTCA  CTCCCTGGACATATTCTAGAACA
                            (TATA+)                               (INR-)           (MTE+)                           
    285 Human: PDZK1 (5174, NM_002614) 
Mouse: Pdzk1 (59020, AI226922) 
 
           |-50      |-40      |-30      |-20      |-10      |1        |11       |21       |31       |41       
Human     CGCAGGGCCAGGCAGGTGGGCCAGAGCTTTTGGTTTGCTGAGGTTTG  TCA G ATTT  TCCAGCTCAGGGC  CCAGCCAGCTGGC  AGGAAGCAGGACAGAGGTC
                                                           (INR+)                    (MTE+)                       
Mouse     ATTGTTAGGCT  GGGGTGGG  GGC  GGGGCTT  GATTTTACTGAGGCCTCTTGA   G CATTTCA  AGCTTAGGACCCTG  CCAGCTGGCCGGG  AGCAGGACAGAGGTC
                       (BRE-)      (TATA-)                        (INR+)                     (MTE+)                   
    286 Human: PFDN2 (5202, NM_012394) 
Mouse: Pfdn2 (18637, AA289888) 
 
           |-50      |-40      |-30      |-20      |-10      |1        |11       |21       |31       |41       
Human     TGGGCGGTAACTCGCAGGCCGGACGTGACGCAAGGCGGGGGCTGAGCTGC G CCGGCCGGAAACCCAGCAGGCGGCGA  AGATGG  CGGAGAACAGCGGTCGC
                                                                                        (DPE+)                  
Mouse     GGAGCAAGTGGGCGGTAACCCCCCGGCCGGATGTGACGCAGTG  AGAGTGC G   CAGGCCGGAAAGCTTGCAGGTGGGGA  AGATGG  CGGACAGCAGCGGTCGT
                                                       (INR-)                             (DPE+)                  
    287 Human: PFKFB2 (5208, CR749442) 
Mouse: Pfkfb2 (18640, AK016729) 
 
           |-50      |-40      |-30      |-20      |-10      |1        |11       |21       |31       |41       
Human     GAGTCAGAAAAAAGTCA  AGCTTTT  TATTAACACCGCCCCCAGCCCTA  TTA G TTAC  CAAGCAACAGTG  CCACCAGGCTCCT  CGGCGCTGGGAAACTCAGGA
                            (TATA-)                          (INR+)                   (MTE-)                        
Mouse     GGGGGGGGGGAAGGGA  AGGCTTT  TAATTCACCCCGCCTCCAGCT  CCATTT G T  TTCCAGGCAACAATGCCAC  CAAGCTCCCCTGG  GCCGGCTGCTTTGCGA
                           (TATA-)                        (INR+)                          (MTE+)                    
    288 Human: PFKFB2 (5208, NM_006212) 
Mouse: Pfkfb2 (18640, AK016729) 
 
           |-50      |-40      |-30      |-20      |-10      |1        |11       |21       |31       |41       
Human     ACGCGCGAGGG  GGCGGGGC  CAGACGGGCTCACATGATTTGCCGG  CGACTG T A  GCGCCGGTCCCGGCCACAA  GCTGTCGGCTCGG  TTCGGTCGCGTTACAG
                       (BRE-)                             (INR-)                          (MTE-)                    
Mouse     CGCCAGCGTG  GGCGGGGC  CAAGCGCCCCACGTGACTCGCCCCAACCCCCG G AGCCCGGACTACTTCAGTCTG  TCTGCCAGCTTTG  GTACCCGGTGCTCCG
                      (BRE-)                                                             (MTE-)                   
    289 Human: PGM1 (5236, NM_002633) 
Mouse: Pgm2 (72157, BB651520) 
 
           |-50      |-40      |-30      |-20      |-10      |1        |11       |21       |31       |41       
Human     AATCAGTAAGGCTCCACCCATTTCACAGGAGGCCTAGAGACCGGCGCT  CC C CTTTC  CCCTCCCGCCGGACCTGCCAGGAGGTGGGCTGGCGCGGAGGGAG
                                                            (INR+)                                              
Mouse     AGATGTGTCAGTCGGGGAGACTCCGCCCCTCTCGACCCCGTGACCCGTC  C C TCTTCC  CAGGCAGCCGGACTTTGGCGAAGTGGGCGGGCCGTGGGCAGGC
                                                             (INR+)                                             
    290 Human: PIGC (5279, NM_002642) 
Mouse: Pigc (67292, AK016394) 
 
           |-50      |-40      |-30      |-20      |-10      |1        |11       |21       |31       |41       
Human     CGTCGCGATGGTTCCAGCCGGGGAAGGGTGCCCTCGCTAAGGAGATTGCG G CGGACCCGGAAGTGCTTGGCCAC  AGTCGC  AGCCCCGGCGCCCCGAAGCG
                                                                                     (DPE+)                     
Mouse     GACCTCGC  GGTGTGGA  GTCTCGGGGCGGGACGCTTTGGCCGCCGAGTGCT G CGGATCCGGAAGTGCTCCTCCACAGT  AGTCCC  CTTCCAAGCCGGCGTCC
                    (BRE-)                                                                (DPE+)                  
    291 Human: PIGR (5284, X95880) 
Mouse: Pigr (18703, Y16523) 
 
           |-50      |-40      |-30      |-20      |-10      |1        |11       |21       |31       |41       
Human     GTGCCTGTGGGAGAGTGGCCC  TTTAAGA  GCCCAGGTGTGGGTCAA  ACACT G AG  CAGAGTTTCAGTTTTG  GCAGCAGCGTCCA  GTGCCCTGCCAGTAGCTC
                                (TATA+)                    (INR-)                       (MTE+)                      
Mouse     CTTGACTATGGGACAACGGCCC  TTTAAGA  GCCAGGTGCGGGTCAAGCCCA G A  AAACAGTT  TCAGTTTTGACAATAGTCACCAGTAGTGCCTTCCTGGAAG
                                 (TATA+)                          (INR-)                                          
    292 Human: PIK3C2B (5287, NM_002646) 
Mouse: C330011J12Rik (240752, AK049181) 
 
           |-50      |-40      |-30      |-20      |-10      |1        |11       |21       |31       |41       
Human     TCCGGGAGATGGCTTCCCCTCTCTCGCCCGCTGCTGCCGCTATTGTCTCA C CCGGGGTGGCCTGAC  TCTGCGAGCTCGC  GCACGCTCTCGGCGGAGAGCA
                                                                                 (MTE-)                         
Mouse     TCCGGGAGATGGCTTCTCCCCGCTCGCTCGCCGTTCGCGCTATTGTCTCA C CCGGGGCTGCCTG  ACTGCGAGCTCGT  GCACGCTCCCGGCAGACAGCACC
                                                                               (MTE-)                           
    293 Human: PIK4CB (5298, BC040300) 
Mouse: Pik4cb (107650, NM_175356) 
 
           |-50      |-40      |-30      |-20      |-10      |1        |11       |21       |31       |41       
Human     CTTGTGGCAGCCTGAAGGCCCCCTCAGGCGGCGCCGCGGGCAGCCCCGCA G CCGGGGCCTGGTGCAGCCTC  CGCGGCCGCTGTC  AGGGAAGCGCAGGCGG
                                                                                      (MTE-)                    
Mouse     CTTGAAGTTGCGTGAAGGCCCCTTCAGGCGGCGCCGCGGGCAGCCCCGCA G CCGGGGCCTGGAGCAGCCGC  CGCGGCCGCTGTC  AGGGAAGCGCAGGCGG
                                                                                      (MTE-)                    
    294 Human: PKP1 (5317, Z98266) 
Mouse: Pkp1 (18772, BQ928617) 
 
           |-50      |-40      |-30      |-20      |-10      |1        |11       |21       |31       |41       
Human     GCCCTGCAGCT  CCGCGTCT  GAGCAGCAGCAGCGCGCGGCCCGGT  GGAGTG G G  TGGTGCAGGGCAGGGGTGGTATATCC  TGTCTG  ACGGAGGGCGGGCCTC
                       (BRE+)                             (INR-)                             (DPE-)                 
Mouse     GGCAGCCA  CAGTGCCC  TGCAGCTCGCGTCTGAGCGGCGGCCTGC  GGAGTG G G  TGGCGCAGGGCAGGGGTGGTATATCC  TGTCTG  ACGGAGGGCGGGTCCG
                    (BRE+)                                (INR-)                             (DPE-)                 
    295 Human: PKP1 (5317, Z34974) 
Mouse: Pkp1 (18772, BQ928617) 
 
           |-50      |-40      |-30      |-20      |-10      |1        |11       |21       |31       |41       
Human     GTGCAGGGCA  GGGGTGG  T  ATATCC  TGTCTGACGGAGGGCGGGCCTCG  CCA G TGCC  AGAGAGGGA  CGAACCAGGGTGG  AAGCGCCAGGAGCAGCTGCAGGG
                      (BRE-)  (TATA+)                          (INR+)                (MTE+)                           
Mouse     GCGCAGGGCA  GGGGTGG  T  ATATCC  TGTCTGACGGAGGGCGGGTCCGG  CCA G TGCC  TACTGAGGGA  CGAGCCGGGGCGG  AGGCGCCAGGAGCAGCAGCCGG
                      (BRE-)  (TATA+)                          (INR+)                 (MTE+)                          
    296 Human: PLA2G4A (5321, U11239) 
Mouse: Pla2g4a (18783, BC003816) 
 
           |-50      |-40      |-30      |-20      |-10      |1        |11       |21       |31       |41       
Human     CTTCTATGAGAAGAGAGCGTTCTCCCTCTTCCCCTTTAATTCCACCTTAA A A  CATCTGCA  AAAGCGCAAGGAGA  CCAGCCCACATTT  TAGCCCCTCCTAC
                                                                (INR-)                     (MTE+)                 
Mouse     ATTTAGAATGTTAGTCTTCACCAGAGGAGCTCGCTTGAATTCCACCT  TAA C ATCC  ACAGAGACCAGCCCACTTC  TTAGCCCCTCCTA  CCAGCCGGAGAAG
                                                           (INR+)                          (MTE+)                 
    297 Human: PLA2G4A (5321, M68874) 
Mouse: Pla2g4a (18783, BC003816) 
 
           |-50      |-40      |-30      |-20      |-10      |1        |11       |21       |31       |41       
Human     AGGAGACCAGCCCACA  TTTTAGC  CCCTCCTACTCAGGATAAGACTTTCTC   T AAGTCCG  GAGCTGAAAAAGGATCC  TGACTG  AAAGCTAGAGGCATTGAGG
                           (TATA+)                              (INR+)                    (DPE-)                    
Mouse     GAGACCAGCCCACTTCTTAGCCCCTCCTACCAGCCGGAGAAGACTTTCTA   G AAGTCGG  AACTGTGAAGGGCTCC  CGACTG  AGAGCCAGAAGGCACAGAGA
                                                              (INR-)                   (DPE-)                     
    298 Human: PLOD1 (5351, NM_000302) 
Mouse: Plod1 (18822, AF046782) 
 
           |-50      |-40      |-30      |-20      |-10      |1        |11       |21       |31       |41       
Human     CGGGGGAG  GGCGTGGA  GGGGCGGGGTGGTGCCGCCCCCGGGGCGGGC  CCA G TGCG  TGGCAGCGGGACCTGCGGCCCCGTCGCGAAGTTTCCAGCCCTGCG
                    (BRE-)                                   (INR+)                                               
Mouse     TCCCCGAGGCGGGCGGA  GGCGTGCG  CGGCACCGCCCCCGGGGCGGGC  TCA G TGCG  TGGCAGCGCCCGCCGGCCCCGTCGCGAAGTTTCCAGTTCAGCTTC
                             (BRE-)                          (INR+)                                               
    299 Human: EXOSC10 (5394, L01457) 
Mouse: Exosc10 (50912, AK081014) 
 
           |-50      |-40      |-30      |-20      |-10      |1        |11       |21       |31       |41       
Human     CAATCGCAGGCTCCCCACAC  TATAATC  AGACGCCCCGCCCGCCGCTTCGA C CTGGCGCATGCGTGGTGCGCACGCGTCC  CGTCTC  CTCGGCCGACAAGCT
                               (TATA+)                                                      (DPE-)                
Mouse     CAAGACCCACAAAGCTACGCTACTTGGTGAGGGGTGGGGGGAG  GATTTAA C   CGCGCGCATGCGTGT  TACGCCCGCCTCG  GGCAGCCGGAACAATCTCGCG
                                                       (INR-)                      (MTE-)                         
    300 Human: PRRX1 (5396, NM_022716) 
Mouse: Prrx1 (18933, L06502) 
 
           |-50      |-40      |-30      |-20      |-10      |1        |11       |21       |31       |41       
Human     TCTCCCTTTCTCTCTAACTCTGATGTTGGCAAAGGGGGTTTTCTTAATC  A G ACTGTT  TTTTGGTCCCAGGGAAAGGAGGAAGAAGGAGATTGTGATGGAG
                                                             (INR-)                                             
Mouse     CTCTCCCTTTCTCTCTGGCTCTGATGTTGGCAAAGGGGTTTTCTTAATC  A G ACTGTT  TTTTCGGTCCGAGGAAAAGGAGGTAGGAGATTGTGATGGAGAA
                                                             (INR-)                                             
    301 Human: POU2F1 (5451, NM_002697) 
Mouse: Pou2f1 (18986, AJ489477) 
 
           |-50      |-40      |-30      |-20      |-10      |1        |11       |21       |31       |41       
Human     AGGGGGGAGGGGAGCCAGAGCGAGGGAGGGTTTATCGACCGGG  CGATTTT G   GTTAAAATATTCAAA  ATGGCGGACGGAG  GAGCAGCGAGTCAAGATGAGA
                                                       (INR+)                      (MTE+)                         
Mouse     GAGGGGGAGG  GGAGCGGG  AGCGAGGGAGGGTTTATCGACCGGG  CGATTTT G   GTTAAAATATTCAAA  ATGGCGGACGGAG  GAGCAGCGAGTCAAGATGAGA
                      (BRE-)                             (INR+)                      (MTE+)                         
    302 Human: PPOX (5498, CR613738) 
Mouse: Ppox (19044, BB100674) 
 
           |-50      |-40      |-30      |-20      |-10      |1        |11       |21       |31       |41       
Human     GGTGAACGTGATCCGGGGCCTTCCAAGTCCCGCCAATCCAGATGTAGGA  G A GGTAGG  GTTAGGCGCGTGCCGCGAGAACAGAGTGGACGGAGCGTAGGAG
                                                             (INR-)                                             
Mouse     TCACCAATCCAGAACCC  GCACTCCA  AGACCCTCCAATCAAGAAGCAAG  AA A GTGAA  ACGCAGTCAC  GCAGGCCGCCAGA  AAGGAATCGACGGAGAGTCGG
                             (BRE+)                           (INR-)                 (MTE-)                         
    303 Human: PPP1R8 (5511, NM_002713) 
Mouse: Ppp1r8 (100336, AX775983) 
 
           |-50      |-40      |-30      |-20      |-10      |1        |11       |21       |31       |41       
Human     CGCTTTTCCCTTCTCGGTCTTCCAGTTTCCCGGCGTGCTTAGGGCGCGC  C A AATGGG  AGGGGGAGACGCAAGATGGCGGCAGCCGCGAACTCCGGCTCTA
                                                             (INR-)                                             
Mouse     CGCCTCTTCCCTCCCGAGCTCTTAGTTTCCCGGCGTGCTTCAGACCCGC  C A AATGGG  AGGGGGAGACGCAAGATGGCGGCAGCCGTGAACTCCGGCTCCA
                                                             (INR-)                                             
    304 Human: PPT1 (5538, NM_000310) 
Mouse: Ppt1 (19063, AK014561) 
 
           |-50      |-40      |-30      |-20      |-10      |1        |11       |21       |31       |41       
Human     CGGCGCCACGCCCA  GGCGGGCG  GAGCGCGGTTCCCGGAGTCTCGCGCCCG C GG  TCATGTGA  CACAGCGAAGATGGCGTCGCCCGGCTGCCTGTGGCTCTT
                          (BRE-)                                   (INR+)                                         
Mouse     AGCCCGGCAGGG  GGCGTGGC  CACCGGAATTACTTTGGTCCACAGTCCCCG C GG  TCATGTGA  TTGCTAAGATGGCGT  CGTCCT  GTTCGCGGAGGCTGCTAG
                        (BRE-)                                     (INR+)                  (DPE-)                   
    305 Human: PRCC (5546, NM_005973) 
Mouse: Prcc (94315, BC027831) 
 
           |-50      |-40      |-30      |-20      |-10      |1        |11       |21       |31       |41       
Human     CGCTGAGATCCCTGCGGCAAAGAACCGGGCTGTGTCCAAAGTGTTCTCTG   G AAGTTGT  AGTTCCTGTATTGGTGAGGCAAGGAGGAGGCGGAGTGACTCG
                                                              (INR-)                                            
Mouse     CACTGAGTTCCCGGAGGTAGAGAATGGGGTTGTTGCTAAAGTCTTCTCTG G   GAATTGTA  GTTCTTAAGTCTGTGAGGTGAGTAGTAGGCGGAGTGACTCG
                                                               (INR-)                                           
    306 Human: PRCC (5546, X99720) 
Mouse: Prcc (94315, BC027831) 
 
           |-50      |-40      |-30      |-20      |-10      |1        |11       |21       |31       |41       
Human     AAGGAGGAGGCGGAGTGACTCGGCGGCCATTAGCTGTGTGTAGTTGCCCG   G GACTAGG  AGCTTAAGT  GAAGAGGTACGCC  TTGTTCGGTGGAAATCAGCC
                                                              (INR-)                (MTE+)                        
Mouse     GAGTAGTAGGCGGAGTGACTCGGCGGCCATTAGGTGTGTGTAG  TTGCTTG G   TGCTAGGAGCTTAAGT  GTAGCGGGAATTC  TTACTCGGTGGAAATCAACC
                                                       (INR+)                       (MTE+)                        
    307 Human: PRELP (5549, NM_002725) 
Mouse: Prelp (116847, AK047480) 
 
           |-50      |-40      |-30      |-20      |-10      |1        |11       |21       |31       |41       
Human     CCCCAATAGGATCTGGTGG  AAGAAAA  GTCAGGCAAACACAAGCACGC  ACA C ACCA  CTGGGAGATCAGATCTT  CTAGCTGGCTCTC  TGCTGCCACAGCTCC
                              (TATA+)                        (INR+)                        (MTE-)                   
Mouse     CCCCAATAGGATCTGGTAG  AAGAAAA  GTCAGGCAGACACACGCAGACACG C AC  CAACTGGG  AGACCAGAACTT  CTAGCTGGCTCTC  TGCTGCCACAGCTC
                              (TATA+)                              (INR-)                   (MTE-)                  
    308 Human: PRKACB (5567, NM_002731) 
Mouse: Prkacb (18749, M21096) 
 
           |-50      |-40      |-30      |-20      |-10      |1        |11       |21       |31       |41       
Human     CGCAGAGAGGGCGGA  CCGCGCGA  AGGGGGAGTGTCTGCCCGCCGCCG  CCA C TGCT  GCTGCCACCGC  CGTCGCCGCCGCC  GCCGCCGCCGCCGCTGCTGCT
                           (BRE+)                            (INR+)                  (MTE-)                         
Mouse     GCGCGTGGAGGGCG  GGCTCGGG  AAGGGGGAGTGTCCGCGCGCCGCCGCTG C CGCCGCCGCCACCGCCG  CCACCGCCGCCAC  CGCCACGGTCCCGGTCTCT
                          (BRE-)                                                     (MTE+)                       
    309 Human: PRKACB (5567, AF538872) 
Mouse: Prkacb (18749, M21096) 
 
           |-50      |-40      |-30      |-20      |-10      |1        |11       |21       |31       |41       
Human     GGGTGCAGACGCGGGAGTTGTCCCAGACTGTGGAGTGGCGGGCACGGCCC C AGCCCCCCTTCCCTTCCC  TGACCCCTTCTTG  CCATCGCCCCAGACATGG
                                                                                    (MTE+)                      
Mouse     AAGCCTAGCCAAAGTTGCTGCCGACTGCGGGGCGGGCGGCGGGACGGCCC C GGTCCCCTCCCCTCCG  TCATCCCTGCTTG  CGGACTCCCGGGTCATGGGG
                                                                                  (MTE+)                        
    310 Human: PROX1 (5629, NM_002763) 
Mouse: Prox1 (19130, BY133648) 
 
           |-50      |-40      |-30      |-20      |-10      |1        |11       |21       |31       |41       
Human     TTGTTCTTGAATGAGAAAGG  AAGAAAA  GAGCCTCCCATTACTCAGACC  CG T GTAAA  CATTATTCCCCCCAGGAGAAAATGGTGTTATTCAAATGAATCAT
                               (TATA+)                        (INR-)                                              
Mouse     TCGTTCTTGAATGGGAAAGAGAGAGAAGAGCCTCCCGTTACTCACACC  CG T GTAAA  CATTCTTTTTCCAAGGGGGAAAATGGTGTTATTTACATGAATCG
                                                            (INR-)                                              
    311 Human: PSEN2 (5664, NM_012486) 
Mouse: Psen2 (19165, AK014706) 
 
           |-50      |-40      |-30      |-20      |-10      |1        |11       |21       |31       |41       
Human     GCGTGGGGCGG  GGCCTGGG  CCGGCGCCGGGTCCGGCCGGGCGCTCAG  CCA G CTGC  GTAAACTCCGCT  GGAGCGCGGCGGC  AGAGCAGGTGAGCGGGCGGT
                       (BRE-)                                (INR+)                   (MTE+)                        
Mouse     ACCGGGGCAGTG  GGCGGGGC  CGGCGCTAGCCCAGGAGAGGCGCTCCGCCC G C  GGCGTAAA  CTCT  ACCCCGCGCCGCG  GCGGAGCAGGTGAGCGGAGGGCG
                        (BRE-)                                    (INR-)           (MTE-)                           
    312 Human: PSEN2 (5664, L44577) 
Mouse: Psen2 (19165, AK014706) 
 
           |-50      |-40      |-30      |-20      |-10      |1        |11       |21       |31       |41       
Human     CAAGCCTAGAGCCGGTTTCTGTTAGCAGCGGTGTTTGGCTGTTTTATCAG   G CATTTCC  AGCAGTGAGGAG  ACAGCCAGAAGCA  AGCTTTTGGAGCTGAAG
                                                              (INR+)                   (MTE+)                     
Mouse     AGGGCCTGAGGCA  GGCGTTCT  GTTACCAGCTGTGTTCAGTGTTTT  GTAAA T CC  CTTAAGGCAGTAG  GGAGACGCCCAGA  ATCAAGCCTCTGAACCGAAGG
                         (BRE-)                            (INR+)                    (MTE+)                         
    313 Human: PSEN2 (5664, U34349) 
Mouse: Psen2 (19165, BY097802) 
 
           |-50      |-40      |-30      |-20      |-10      |1        |11       |21       |31       |41       
Human     GCTGCCCTCTTTGAAAGCCAGGGAGCATCATTCATTTAGCCTGCTGAGAA   G AAGAAAC  CAAGTGTCCGGGATTC  AGACCT  CTCTGCGGCCCCAAGTGTTC
                                                              (INR-)                   (DPE+)                     
Mouse     AAGGTGACCTTTTGAAA  GATAAGG  GTATTGTTCTGTTGGCCCTTTGGA  AA A GTGTC  AGGGTGTCCTAACCTCCCTGC  AGATAG  ACTTGTTTGTGGCCCAG
                            (TATA+)                           (INR-)                        (DPE+)                  
    314 Human: PSMB2 (5690, D26599) 
Mouse: Psmb2 (26445, AK078487) 
 
           |-50      |-40      |-30      |-20      |-10      |1        |11       |21       |31       |41       
Human     TGTACGTCATCCGA  GAGCGCCG  TGGAAGTCGTGCTGCAGGCGTCGCG  CCA A TCTT  CGCTCTGAGGTGCTGTCTCACCGGTG  AGACCT  GGAAGCGGGCGAG
                          (BRE+)                             (INR+)                             (DPE+)              
Mouse     TCATTGGG  CCGCGCCA  GTGACTGGGCCGTCGCGCGTCTTGCGTCACCCCC A GC  TCAGTCCG  GGTGTGCTGTCTCACTGGTGA  GGTCGT  GTACCCGGGAAG
                    (BRE+)                                         (INR+)                        (DPE+)             
    315 Human: PSMB4 (5692, NM_002796) 
Mouse: Psmb4 (19172, BU524742) 
 
           |-50      |-40      |-30      |-20      |-10      |1        |11       |21       |31       |41       
Human     AAGAAAGAAATGACACTGAAGGATCACTTCCGCTTCCGTTGGCGCAAGCG C TT  TCATTTTT  TCTGCTACCGTGACTA  AGATGG  AAGCGTTTTTGGGGTCG
                                                                 (INR+)                   (DPE+)                  
Mouse     AAGAAGAGA  GGCGTGGC  AAAGGACCACTTCCGTTTCCGGTGACGCTAGCG C TT  TCATTTCT  GTCGGCTGAGGTGACTA  AGATGG  AAGCGTTTTGGGAGTC
                     (BRE-)                                        (INR+)                    (DPE+)                 
    316 Human: PSMD4 (5710, NM_002810) 
Mouse: Psmd4 (19185, BI687156) 
 
           |-50      |-40      |-30      |-20      |-10      |1        |11       |21       |31       |41       
Human     TGTGATTGGTGAAGAGCGACGGGCCAATTGGAGGAGTTGTTGTTAGGCCG T CCCGGAGACCCGGTCGGGAGGGAGGAAGGTGGCAAGATGGTGTTGGAAA
                                                                                                              
Mouse     TTTGATTGGTGATGAGCA  CCAGGCCA  ATCGGAGGTGCTGTTGTTTG  TCAG T CCC  GGAGCCGGTCC  GCAGAGGGAAGGA  GGCA  AGATGG  TGTTGGAGAGCA
                              (BRE+)                        (INR+)                  (MTE+)         (DPE+)             
    317 Human: PTGFR (5737, AB041713) 
Mouse: Ptgfr (19220, AK076450) 
 
           |-50      |-40      |-30      |-20      |-10      |1        |11       |21       |31       |41       
Human     GGATCCCAGGAGCCGC  GCGCGCCC  CGCAGTTTCCGCGCTAAGGGAA  CGAG T GCG  CGGAGGGGACGAGCGGCTGGACCACAGCCGGCGCCCGATCAGGATC
                            (BRE+)                          (INR-)                                                
Mouse     CTCTAGGTGCTCTGGAGTTCAGGACACAATAGGTCCTGCGGGTGAT  CCAG T GCT  CAGAAGTGTCCTAGGGAGGAAAGAGAGGTGGAACCCGAGGTTGCGG
                                                          (INR+)                                                
    318 Human: PTGS2 (5743, NM_000963) 
Mouse: Ptgs2 (19225, M64291) 
 
           |-50      |-40      |-30      |-20      |-10      |1        |11       |21       |31       |41       
Human     TGGGCTTGGTTTTCAGTCT  TATAAAA  AGGAAGGTTCTCTCGGTTAGCGA  C C AATTGT  CATACGACTTGCAG  TGAGCGTCAGGAG  CACGTCCAGGAACTCC
                              (TATA+)                          (INR+)                     (MTE+)                    
Mouse     CGTGGAGTCCGCTTT  ACAGACT  TAAAAGCAAGGTTCTCCCCATTAGCAG  C C AGTTGT  CAAACTGCGA  GCTAAGAGCTTCA  GG  AGTCAG  TCAGGACTCTGC
                          (TATA-)                              (INR+)                 (MTE-)       (DPE+)             
    319 Human: QSCN6 (5768, L42379) 
Mouse: Qscn6 (104009, AA023328) 
 
           |-50      |-40      |-30      |-20      |-10      |1        |11       |21       |31       |41       
Human     CCTCCTTGCGGGCGGAGGCAGGCGGTGCCGCGGCGCCGGGACC  CGACTCA T   CCGGTGCTTGCGTGTGGTGG  TGAGCGCAGCGCC  GAGGATGAGGAGGTGC
                                                       (INR+)                           (MTE+)                    
Mouse     CCTCCGGGGCGGAGGCTGTTGGTGCGCGGCAGGCTCCGGATACTGACTAG T C  ACAAACTT  GAAGGAGGTGGACATTCAAGCCGCCTAGGATGAGGAGGTG
                                                                (INR+)                                          
    320 Human: PTPN7 (5778, NM_002832) 
Mouse: Ptpn7 (320139, NM_177081) 
 
           |-50      |-40      |-30      |-20      |-10      |1        |11       |21       |31       |41       
Human     CAAGGAGGGGCGGAGGGAGGAAGCTGGCTTCCTGGAGCCTTCTCAGCCCT C AAAGACAGACCGACAGACAGAC  AGACAG  CTGGCAAGAGGCAGCCTGGGG
                                                                                    (DPE+)                      
Mouse     GCCAGGAGGGGCGGAGGGAGGAAGCTGGCTTCCTGAAACCTCTCAGTCCT C AAAGATAGACGGACTGACAG  ACAGCTGGCAAGA  GGCAGCCTGGGGGACA
                                                                                      (MTE+)                    
    321 Human: PTPN14 (5784, BX538306) 
Mouse: Ptpn14 (19250, AA798138) 
 
           |-50      |-40      |-30      |-20      |-10      |1        |11       |21       |31       |41       
Human     CTCGCATTTCCTGCCGCTCTGGCTCTCCCGGCCCCTCAAAGTTCTTTC  CA A CTTTT  TCTCGGCGGAG  TGAGCGCAGCGGG  CGCAGACTCGGGGGCAGGTT
                                                            (INR+)                  (MTE+)                        
Mouse     CGCTCGCGCTTCCT  GGCGCTCG  GGCTCTCCGGAGGCTCAAGTTCTGCA  CA A CTTCT  CCCGGCTGC  CCAGCGCCTCGGC  CGCGGGCCCGGCCAGCTAGCTG
                          (BRE-)                              (INR+)                (MTE+)                          
    322 Human: PTPRC (5788, M23461) 
Mouse: Ptprc (19264, AK054056) 
 
           |-50      |-40      |-30      |-20      |-10      |1        |11       |21       |31       |41       
Human     TGCAAAGAGGACCCTTA  CAGTATT  TTTGGAGAAGTTAGTAAAACCG  AATC T GAC  ATCATCACCTAGCAGT  TCATGCAGCTAGC  AAGTGGTTTGTTCTTAG
                            (TATA-)                         (INR-)                       (MTE-)                     
Mouse     GCAAAGAGGACCCTTTA  CAGTATT  TTTGGAGAAGTTAGTAAAACCG  AATC T GAC  ATCACCATTTA  GCAGTGCATGTAG  CTAGCAAGTGGTTTGTTCTTAG
                            (TATA-)                         (INR-)                  (MTE+)                          
    323 Human: PTPRC (5788, NM_002838) 
Mouse: Ptprc (19264, AK054056) 
 
           |-50      |-40      |-30      |-20      |-10      |1        |11       |21       |31       |41       
Human     ATCATCACCTAGCAGTTCATGCAGCTAGCAAGTGGTTTGTTCTTAGGG  TA A CAGAG  GAGGAAATTG  TTCCTCGTCTGAT  A  AGACAA  CAGTGGAGAGTATG
                                                            (INR-)                 (MTE-)      (DPE+)               
Mouse     ATCACCATTTAGCAGTGCATGTAGCTAGCAAGTGGTTTGTTCTTAGGGTA A G  AGAGTAGG  AAACTTGCTCCC  CATCTG  ATAAGACAGAGTGCAAAGTATG
                                                                (INR-)               (DPE-)                       
    324 Human: PTPRF (5792, Y00815) 
Mouse: Ptprf (19268, BQ769052) 
 
           |-50      |-40      |-30      |-20      |-10      |1        |11       |21       |31       |41       
Human     GGCAGGGGCGCG  GGCGCGAG  CGCGAGGGGAGCGCGCGGCTGGAGCTGGCG C GGGAGCGGCGGGAGCGGTG  GCGGCGGCAGAGG  CGGCGGCTCCAGCTTCG
                        (BRE-)                                                         (MTE+)                     
Mouse     GGGCAGGGACGCG  GGCGCGGG  CGGGCGGGCGCGCGCGGCTGGAGCTGGCG C GGGAGCGGTGGCGGCGGCGGCG  GCAGAGGCGGCGG  CTCCGGCTCCAGCT
                         (BRE-)                                                           (MTE+)                  
    325 Human: PEX19 (5824, Y09048) 
Mouse: Pex19 (19298, AK029368) 
 
           |-50      |-40      |-30      |-20      |-10      |1        |11       |21       |31       |41       
Human     TGGAGGGCGTGTCGGCACCGAGGAGGTCCCGCCTCCTACGGCAAGTCG  GA G GTAGC  AAGATGGCCGCCGCT  GAGGAAGGCTGTA  GTGTCGGGGCCGAAGC
                                                            (INR-)                      (MTE-)                    
Mouse     GGCGGGGCATGTGG  TGGCGCCA  GTGGTTCCACCTCCTACGGCAAGTCGGA G   GCAGTAAG  ATGGCGGCTGCTGAGGAA  GGTTGC  GGTGTTGGGGTCGAAGA
                          (BRE+)                                 (INR-)                     (DPE+)                  
    326 Human: ABCD3 (5825, BC068509) 
Mouse: Abcd3 (19299, BC009119) 
 
           |-50      |-40      |-30      |-20      |-10      |1        |11       |21       |31       |41       
Human     GGCCGGCCCCGCCCTCTGCTCTCCTCCCAGTCTCCCCCGCGCTGCGT  GCA G TAAG  GTAGCCGCCGCCGC  CGCCGCCGCCGCG  TCCCCTCGCCGGCTCGCT
                                                           (INR-)                     (MTE-)                      
Mouse     CCCTCTCTC  GGCGCCGA  GCTATCCTCCCAGTTTCCCCCGCGGTTCCT  GCA C TGAG  GTTGTCGCCGGCGCGTCCCC  TATCCC  GCGGCTCGCTCGCCCTGCC
                     (BRE-)                                  (INR-)                       (DPE-)                    
    327 Human: RAB3B (5865, NM_002867) 
Mouse: Rab3b (69908, AK082959) 
 
           |-50      |-40      |-30      |-20      |-10      |1        |11       |21       |31       |41       
Human     GAGGGCCGG  GGCGGGGC  GAACACAGACTCCGCCCTTGGGCGGGGCCTGGA T GCGGCCGGAGCGGAGCAGTGCT  GGAGCGGGAGCCT  CAGCCCTCAGGCGC
                     (BRE-)                                                               (MTE+)                  
Mouse     CAGGGGCGGGGCGGATCTTACATGGGTTCCGCCCAGGGGCGGGGCTTGGA C CCAGCGGGAACCCAACCCATCT  TCAGCCAGAGCCT  CAGCCCTCCGTAGC
                                                                                        (MTE+)                  
    328 Human: RAB13 (5872, X75593) 
Mouse: Rab13 (68328, BC027214) 
 
           |-50      |-40      |-30      |-20      |-10      |1        |11       |21       |31       |41       
Human     CCTGGGCT  CCGTGCCG  CTCTGTTTGCCAACCGTCCAGTCCCGCCTA  CCAG T GCC  GGGCGCTCCCCACCCC  TCCCCCGGCTCCC  CCGGTGTCCGCCATGGC
                    (BRE+)                                  (INR+)                       (MTE-)                     
Mouse     CTGGGACTCCGGCGGTTTCTGCCTGCCAACCCTCGGATCCCGCCTA  CCAG T GTT  GGCTCTTCCCGACCCCTC  CCCCGGCGCCCCC  AGTGTCCGCCATGGC
                                                          (INR+)                         (MTE-)                   
    329 Human: RAB13 (5872, NM_002870) 
Mouse: Rab13 (68328, BC027214) 
 
           |-50      |-40      |-30      |-20      |-10      |1        |11       |21       |31       |41       
Human     TCAAAAGGGTGGGCGGGCAGGGGGTGGAGCCCGGAGGGGTGCCGCCTCT  C C TCTTTC  CCGGAGCCTGGGC  GGAGAGGGAGGAA  AACTTCTTCCTGGCCTG
                                                             (INR+)                    (MTE+)                     
Mouse     GGCCTCTCCACTGGCGAAGAGGGGTGGAGCCGAGAGCAGGGCCGCC  TCTC T TCT  TTTCCCGGGGCCTGGGC  GGAGAGGGAGGAA  AACTTCCTGGCTGGGA
                                                          (INR+)                        (MTE+)                    
    330 Human: RABIF (5877, BC037392) 
Mouse: Rabif (98710, BC011166) 
 
           |-50      |-40      |-30      |-20      |-10      |1        |11       |21       |31       |41       
Human     CCGCCCCGCCTCTG  CCAAGCCG  CGCCAGCGCAGTGAATAACGAGCCAGCA G AGACAGCGTCTGC  GCAGCCGTGGCTG  AGGAGCCTGTGGCGGCAGCGGCG
                          (BRE+)                                                 (MTE+)                           
Mouse     GCGGGGCGCCCCCAGGGACCTGCGCCAGCGTAGTTCCGGGTGAGCCAGC  T C AGATCG  CGCGTGCGC  CGTTGTGGCTGAT  GGTGTGGCGGCACGGCGATGG
                                                             (INR+)                (MTE-)                         
    331 Human: RABIF (5877, NM_002871) 
Mouse: Rabif (98710, BC011166) 
 
           |-50      |-40      |-30      |-20      |-10      |1        |11       |21       |31       |41       
Human     ACAAAGCCTGC  CGACACCT  AGCCACTTAGGTTAGGCAATGAATGAAGGCG C TTGCGCAGTAGAGTCC  AGAGCGGGGAAGC  GGGCGCCTGCGCAGAGACAT
                       (BRE+)                                                       (MTE+)                        
Mouse     GATTGTCACAAGTAACTCCTGACACTTCCGTCCGGCAATAGAGAAAGGCG C CTGCGCAATAGAGT  TCAGACGCCTGCG  CAGAGGTACAGCGAGAGGCACG
                                                                                (MTE+)                          
    332 Human: RAP1A (5906, NM_002884) 
Mouse: Rap1a (109905, BC051419) 
 
           |-50      |-40      |-30      |-20      |-10      |1        |11       |21       |31       |41       
Human     GCCGCTCCCGAGGCCCCTGCCGCCGCCGCTCCCGCTGCTGTCGCCGCGCA G AGCCGGAGCAGGAGCCACGGC  CGAGAGGAGGGAG  GAGGAGGAGGAGGAG
                                                                                       (MTE+)                   
Mouse     GGCGCCGCCGCCG  CCGCTCCC  GAGGCCGCTGCTCCTGCCGCCGCCGTGCA G AGCCCGAGCCCGAGCCGCGGC  CGAGAGGAGGGAG  GAGGAGGAGGTGGAG
                         (BRE+)                                                          (MTE+)                   
    333 Human: RAP1GA1 (5909, NM_002885) 
Mouse: Rap1ga1 (110351, AK005063) 
 
           |-50      |-40      |-30      |-20      |-10      |1        |11       |21       |31       |41       
Human     CATGTGACGTCCCCCTCCCCGAACTGCGGCGGCGGCGAGCGCCGGCCG  CA T CTGAG  CAGAGCTGCA  GCGGCGGCCGCGG  GCACCAGAGTGCCGAGCCCAG
                                                            (INR-)                 (MTE+)                         
Mouse     ACGTTCCCCCTCCCCGAACTGCGGCGGCGGTGGCGGCAGCGGCGGCGGCG G C  AGAGAGCG  AGTCC  CGGCAGAGCTGCA  GCGGCAGCCGCGGGCACCGGAG
                                                                (INR-)            (MTE-)                          
    334 Human: RBBP4 (5928, X74262) 
Mouse: Rbbp4 (19646, BY089165) 
 
           |-50      |-40      |-30      |-20      |-10      |1        |11       |21       |31       |41       
Human     GCTGATGTT  GGCGCGAA  GGTGCGCGAGTCAGCCCTCGCGCTGGGGGCGCA G GAAACAATAGAGGCCGCGCGCAC  AGAGCGAGCTCTT  GCAGCCTCCCCGC
                     (BRE-)                                                                (MTE+)                 
Mouse     TCGGTGTT  GGCGCGAA  GGCACGCGAGCCGGCCCCCTACGCACGGGCCGCA G GAAACAATAGAGGCCGCGCGCGC  GGAGTGGAGCGCT  CGTAGCCTCCCCG
                    (BRE-)                                                                 (MTE+)                 
    335 Human: RBBP5 (5929, NM_005057) 
Mouse: Rbbp5 (213464, AK049247) 
 
           |-50      |-40      |-30      |-20      |-10      |1        |11       |21       |31       |41       
Human     GCAGCCCACCTGAAACGAACAAGGAAGGCGGGGGAAGAAAGCGGAAGCCG C GGGGCCTTCTAAGGCCGAAAGT  CTTCGGAGCTTGC  GCCAGTCTCTTCGC
                                                                                        (MTE-)                  
Mouse     GACAGCTACGTAAAACGAACAAGGAAGGCGGGGGAGGAAAGCGGAAGCCG C GGGGCCTTCTAAGTCCGAAAGT  CTCCGGAGCTTGC  GCCAGGCTCTTCGC
                                                                                        (MTE-)                  
    336 Human: RFX5 (5993, NM_000449) 
Mouse: Rfx5 (53970, BC051965) 
 
           |-50      |-40      |-30      |-20      |-10      |1        |11       |21       |31       |41       
Human     TGACAGAGCAGGCCGC  CCGTGTA  TCTAGGCAGATCGCGGCTGTTACAA  CC A TTCAG  AAAAAGGAAAATAAA  GCAACGCACGAGC  CGGCTGGGCCAGGACC
                           (TATA-)                            (INR+)                      (MTE+)                    
Mouse     TCACAGAGCAGGCC  GTGCGTG  TATCCAGGCAGATCGCGGCTGGCATAA  CC A TTCAG  AAAAAGGAAAAATAAAA  GCAACGCACGAGC  CCGCCGGCTGGGCA
                         (TATA-)                              (INR+)                        (MTE+)                  
    337 Human: RGS1 (5996, NM_002922) 
Mouse: Rgs1 (50778, AK089293) 
 
           |-50      |-40      |-30      |-20      |-10      |1        |11       |21       |31       |41       
Human     AGTGCCTGTCTGCATTCTACTA  TATAAAG  CAGCAGAGACGTTGACTAGCG C   ATATTTGC  TAAGAGCACCATGCGCGCAGCAGCCATCTCCACTCCAAAGT
                                 (TATA+)                         (INR+)                                           
Mouse     CAAGTGCCTGTCTGCCTTACGCTA  TATAAAG  CAGCAGTCGTAGACTAACA G   ACACTCAC  TCGTTTTGA  GAAGACCATGAGA  GCGGCAGCCATCTCCATGC
                                   (TATA+)                       (INR+)                (MTE+)                       
    338 Human: RGS2 (5997, L13463) 
Mouse: Rgs2 (19735, AK077922) 
 
           |-50      |-40      |-30      |-20      |-10      |1        |11       |21       |31       |41       
Human     CCCCGCCC  CCAAGCCG  AGGCCT  CATAAAT  GCTGCGACGCACGCCCAGCCG C   AAACAGCC  GGGGCT  CCAGCGGGAGAAC  GATAATGCAAAGTGCTATGTTC
                    (BRE+)         (TATA+)                         (INR-)             (MTE+)                          
Mouse     CGCCCCTAT  GCCGCCG  C  CCGCCT  TAAAAGCCCGCGGCGCGCTCCCGGCC  T C AAACAG  CTGCGGTG  GCCGCGGGAGTCT  GAGAATGCAAAGTGCCATGTTC
                     (BRE-)  (TATA-)                             (INR+)               (MTE+)                          
    339 Human: RGS13 (6003, CR599001) 
Mouse: Rgs13 (246709, AF498319) 
 
           |-50      |-40      |-30      |-20      |-10      |1        |11       |21       |31       |41       
Human     TGTCTCACAAAATTTCAGAA  CCTGATT  TCAAACGGATCATAACAAAGAGG A GA  TCAAATTT  AGCATG  GTGGACTGCTCGA  CA  GGATAT  ATTTGTAAGTAT
                               (TATA-)                             (INR+)             (MTE-)       (DPE+)             
Mouse     TGTCTCGTGCAATTTCAGAG  CCTGATT  TCAAAAGGATCGTAACAAGGAG  A A AATAAA  ATGTAGTTTG  GTAGACTGCTCCA  CG  GGATAT  ATTTGTGAGTAT
                               (TATA-)                         (INR-)                 (MTE-)       (DPE+)             
    340 Human: RGS16 (6004, NM_002928) 
Mouse: Rgs16 (19734, NM_011267) 
 
           |-50      |-40      |-30      |-20      |-10      |1        |11       |21       |31       |41       
Human     CGCCCCGCTCCTCCGA  GGGGATT  TAAAGCAGCGGCCGCTCAGTCTGGGCG C TTGCAGGCTGCTAAACCCAACCGC  AGTTGA  CTAGCACCTGCTACCGCGC
                           (TATA-)                                                      (DPE+)                    
Mouse     CCCCGCCCCGCCCCACGGG  GATAAAA  GCCTTAGTAGCCACCGGCCCGGCG C GTGCCGCTCTCCAGACCTCTTCTGCGCAGCCTGGTACTTGCTACTCGCT
                              (TATA+)                                                                           
    341 Human: RHCE (6006, X54534) 
Mouse: Rhced (19746, AF531096) 
 
           |-50      |-40      |-30      |-20      |-10      |1        |11       |21       |31       |41       
Human     CTCCCCTATCGCTCCCTCAAGCCCTCAAGTAGGTGTTGGAGAGAGGGGTG A TGCCTGGTGCTGGT  GGAACCCCTGCAC  AG  AGACGG  ACACAGGATGAGCT
                                                                                (MTE+)       (DPE+)               
Mouse     CGGGTGTCTCTCCCTATCGCTCCTTCAAGTGGGCGTGGGGGACACCCGTG A AGCCTGCAGCTGGC  AGAACCCGGGCAC  AGCAACAGACACAAGATGGGCT
                                                                                (MTE+)                          
    342 Human: RHD (6007, X63094) 
Mouse: Rhced (19746, AF531096) 
 
           |-50      |-40      |-30      |-20      |-10      |1        |11       |21       |31       |41       
Human     ACAACCAGCCTTGCAGCCTGA  GATAAGG  CCTTTGGCGGGTGTCTCCCCTA T CGCTCCCTCAAGCCCTCAAGTAGGTGTTGGAGAGAGGGGTGATGCCTGG
                                (TATA+)                                                                         
Mouse     AGGCCAGCCCGGCAGCCTGA  GATAAGA  CCTTTGGCGGGTGTCTCTCCCTA T CGCTCCTTCAAGTGGGCGTGGGGGACACCCGTGAAGCCTGCAGCTGGCA
                               (TATA+)                                                                          
    343 Human: RLF (6018, NM_012421) 
Mouse: Rlf (109263, AK045021) 
 
           |-50      |-40      |-30      |-20      |-10      |1        |11       |21       |31       |41       
Human     TGCGCGGGCGGCCGGGGGCAGGGCCAGGGCTGAGTCACGTGGCTTGGTTG C CTACGCGCTGGTGGGCCGTGGGA  AGATGG  CGGACGGAAAGGGAGACGCC
                                                                                     (DPE+)                     
Mouse     TAACACGGGCGATTGGG  GGCGGGGC  CAGGCGGAGTCACGTGGTTTGGTTG C CTACGCGCTGCTGGGCCGTGGGA  AGATGG  CGGACGGAAAGGGAGACGCC
                             (BRE-)                                                    (DPE+)                     
    344 Human: RNF2 (6045, NM_007212) 
Mouse: Rnf2 (19821, BB579288) 
 
           |-50      |-40      |-30      |-20      |-10      |1        |11       |21       |31       |41       
Human     CGGTGGCGCGGGTTCTCCAGGCCAACACGCGGCGGCGCACCCTCCCTCGC G CCGCGGGCCTCCCCGCGCCGTGCA  CGTCCT  CGTCCCGCGCCTCCGCCCC
                                                                                      (DPE-)                    
Mouse     GGGCGTGGCCCGGCGGT  CCGGGCCT  GCACTAGGCACGCCCCTTCCCCTAC G CCAGGGGCCTCCCCGCGCGGTGCGCGTCCG  CGTCCC  GCGCCTCCGCCTC
                             (BRE+)                                                           (DPE-)              
    345 Human: RNF2 (6045, NM_007212) 
Mouse: Rnf2 (19821, BB579288) 
 
           |-50      |-40      |-30      |-20      |-10      |1        |11       |21       |31       |41       
Human     CGGCCCCTGTTGTATTTCCCGAGCTCCATTGCGGAAGCTGAGGCTCG  CCA T ATTG  TGCGGCGGCGCCGGCGTCCGCGGCAGCTGATACCAGAGTCTTGCT
                                                           (INR+)                                               
Mouse     CGGCCCCTGTTGTATTTCCCGAGCTCCATTGCGGAAGCCGAGACTCG  CCA T ATTG  TGCGGCGGC  GCCGGCGGTTGAT  TCTCGAGTCTCGCTCCGGCCACT
                                                           (INR+)                (MTE-)                           
    346 Human: RNPEP (6051, NM_020216) 
Mouse: Rnpep (215615, BC010520) 
 
           |-50      |-40      |-30      |-20      |-10      |1        |11       |21       |31       |41       
Human     CGGCGCTCGG  GGCGGGGC  TCCCCTCGGGTTCGCGGCCCGGCCGGTGAGCA A CGGCTCTGCGGCCATGG  CGAGCGGCGAGCA  TTCCCCCGGCAGCGGCGCG
                      (BRE-)                                                         (MTE+)                       
Mouse     CGGAGCCTG  CCAAGACC  CGAGGAGCGACCGACCTTCCTGGCGGGTGAGCA A CCGCTCAGGAGCCATGG  AGAGCGGCGGACC  AGGCAACTACAGCGCGGCC
                     (BRE+)                                                          (MTE+)                       
    347 Human: RORC (6097, U16997) 
Mouse: Rorc (19885, AF019655) 
 
           |-50      |-40      |-30      |-20      |-10      |1        |11       |21       |31       |41       
Human     CTGCTGGAGGGCCAGGTGCTCCCGCCTTCCACCCTCCGCCCTCCTCCCTC C CCTGGGCCCTGCTCCCTGCCCTCCTGGGCAGCCAGGGCAGCCAGGACGG
                                                                                                              
Mouse     CCCCTGCCTGCTGACGGGCCAGGTGCTCCCTCCCTCTTCCCTCCTCCC  TC C CTTGG  GCCCTGCTCCCTGCCCTCCTGGGCAGCCAGGGCAGCAAGGACGG
                                                            (INR+)                                              
    348 Human: RPA2 (6118, BC021257) 
Mouse: Rpa2 (19891, CN670098) 
 
           |-50      |-40      |-30      |-20      |-10      |1        |11       |21       |31       |41       
Human     GAGCACCGATTGGCTGAAGCGAGCACCCCGGGAGCTGACTGGCTCCG  CCA T TCGC  GGGAAGGCGTTTGTGGTGCCAGAGAAAAGTAGCCAGAGCGGCGCA
                                                           (INR+)                                               
Mouse     GTTCTGATTGGCTGAAGGTAAATGTGTCCAGGGCGCTGATTGGCTG  TCCC T TCG  CGGGAAGGAGTTTGTGGCG  CCAGTGCAAAGTA  GCCAGAAGGCTGCC
                                                          (INR+)                          (MTE+)                  
    349 Human: RPE65 (6121, NM_000329) 
Mouse: Rpe65 (19892, AF432266) 
 
           |-50      |-40      |-30      |-20      |-10      |1        |11       |21       |31       |41       
Human     CTCCTTTTAAGGGATTTAGAAGG  CATAAAA  AGGCCCCTGGCTGA  GAACTT C C  TTCTTCATTCTGCAGTTGGTGCCA  GAACTC  TGGATCCTGAACTGGAAG
                                  (TATA+)                 (INR+)                           (DPE-)                   
Mouse     TCCTCCAATAAGGATTAGATTGCA  TACAAAA  AAGCCCTGGCTAA  GAACTT G C  TTCCTCATCCTACAGCTGGTACCA  GAACTC  TCTCTAATCTTCACTGGA
                                   (TATA+)                (INR+)                           (DPE-)                   
    350 Human: RPL11 (6135, NM_000975) 
Mouse: Rpl11 (67025, BP773178) 
 
           |-50      |-40      |-30      |-20      |-10      |1        |11       |21       |31       |41       
Human     GGGAAAAGA  GCCCGCCT  CCTGGCC  CATAAGG  CCCTCGGCCGGAAGCTCCG C TT  TCTCTTCC  TGCTCTCCA  TCATGGCGGTGAG  TAGCTGGGACCTGGATT
                     (BRE+)          (TATA+)                         (INR+)                (MTE-)                     
Mouse     GAAGAAAA  GCCCGCCT  CCTGACC  CATAAGG  CCACGGGGCCGGAAGTCTCC C TC  TCTCTTCC  TGTTCTCCA  CCATGGCGGTGAG  TAGGCGAGAGTTGGCTT
                    (BRE+)          (TATA+)                          (INR+)                (MTE-)                     
    351 Human: RPL22 (6146, X59357) 
Mouse: Rpl22 (19934, BC007139) 
 
           |-50      |-40      |-30      |-20      |-10      |1        |11       |21       |31       |41       
Human     TTCCGACTGACTGAAACTTCATCTCTGTTATCATTTGTGTATTTTCTTAG   A AAAAGCT  TGTGGTGAAGGGGGGCAAAAAAAAGAAGCAAGTTCTGAAGTT
                                                              (INR-)                                            
Mouse     GGTGGGGGGGACTATCCGAGGCTTCATCCATGTTTTCTGTATACTCCTAG   A AAAAGCT  TGTGGCGAAGGGGGGCAAAAAAAAGAAGCAGGTTTTGAAGTT
                                                              (INR-)                                            
    352 Human: RPL22 (6146, NM_000983) 
Mouse: Rpl22 (19934, BC007139) 
 
           |-50      |-40      |-30      |-20      |-10      |1        |11       |21       |31       |41       
Human     CAAAGTGATTAGTGCGGCCTGGACCCTGCGTGCCTTGCGCGCTGCGG  GCT C TTTG  CGTCTGCGTAGTTCGCTCACCTCCCTTTCTAACTCCGCTGCCGCC
                                                           (INR+)                                               
Mouse     TGATCGATTGAAGTTGCCTGGCTCCTTGCGTGCCCTGTGCGCTGCGGGCT C TTAGCGCCTGCGTAGTGCGCCCCCCTCCCTTTCTGCCTCCTCTGCCGCC
                                                                                                              
    353 Human: RPS8 (6202, NM_001012) 
Mouse: Rps8 (20116, AW550242) 
 
           |-50      |-40      |-30      |-20      |-10      |1        |11       |21       |31       |41       
Human     ACGTTGTTTC  GGCGCTCA  GAAACAACGTAAAGTAAAGGGGCGGGGCAGCG T T  TTACAAAC  CGAACCGTGAAT  CTTTGCGGTTTCT  CTTTCCAGCCAGCGC
                      (BRE-)                                      (INR+)                   (MTE-)                   
Mouse     CCCAGGTCCTTCCCGGCAACCGGAAGAGACGTAAAGAAGGCGGGGC  TGAG T TTT  AGAAACCGGACCGTGAA  GCTTTGCGCTTCC  TCTTTCCAGCCAGCGC
                                                          (INR+)                        (MTE-)                    
    354 Human: RPS27 (6232, NM_001030) 
Mouse: Rps27 (57294, AK088320) 
 
           |-50      |-40      |-30      |-20      |-10      |1        |11       |21       |31       |41       
Human     GTCATTTCCTGTAGTGTGCTC  TATATAA  GGGGCAGGATTTCCGCTTTCGC   T CCTTTCC  GGCGGTGACGACCTACGCACACGAGAACATGCCTGTGAGTGC
                                (TATA+)                         (INR+)                                            
Mouse     GTCACTTCCTGTCCCATATCAGA  TATAAGG  AACAGGATTTCCGCTTTCGC   T CCTTTCC  GGCGGTGACGACCTCCCTACGAGAACATGCCTGTGAGTTCCT
                                  (TATA+)                       (INR+)                                            
    355 Human: RPS27 (6232, M31520) 
Mouse: Rps27 (57294, AK088320) 
 
           |-50      |-40      |-30      |-20      |-10      |1        |11       |21       |31       |41       
Human     AAACGGTAGTTTTGTGTGTTGGCTGCTCCACTGTCCTCTGCCAGCCTACA G GAGGAAAAGCAAGGCTTACAGAAGGTAAATGGTTTACTAATGTGATTTG
                                                                                                              
Mouse     AAACGGTAGTCTTGTGTGTTGGCTGCTCCACTGTCCTCTGTCAGCCTA  CA G GTGGA  AAAGCAAGGCTGACAGAAGGTGAGT  GGTCTC  AGTGTTGGGGTTT
                                                            (INR-)                            (DPE-)              
    356 Human: RXRG (6258, CR596807) 
Mouse: Rxrg (20183, BC058401) 
 
           |-50      |-40      |-30      |-20      |-10      |1        |11       |21       |31       |41       
Human     CGACGGCGGTGGCAAGAGTAGCGGTGACGGCGGCGGCGGCGGCGGCGGCA   G CATTATG  CGTGATTACTGACA  GGCACCAGCTGCT  GCCGCCACAGCCGTC
                                                              (INR+)                     (MTE-)                   
Mouse     GCAGCAGCGAC  GGCGGCGG  CAAGAGGCAAGGAGAGGTAGCAGCGGCGGCA   G CATTATG  CGTGATTACTGACA  GGCACCAGCTGCT  GCGGCCGCCACAGCC
                       (BRE-)                                   (INR+)                     (MTE-)                   
    357 Human: SORT1 (6272, X98248) 
Mouse: Sort1 (20661, BY752668) 
 
           |-50      |-40      |-30      |-20      |-10      |1        |11       |21       |31       |41       
Human     GCGCGTATCCAAGGAGCG  CCGCGCTC  GGCTCGGGGGTGTGGCGCGCGCCG G CGGGGGTGGGCGGGCGCGC  CGGGCGGCAGGTG  TCGGCGTCGGCGGCATT
                              (BRE+)                                                   (MTE+)                     
Mouse     CGCGTATCCAAGC  AGCGCGGG  GCCGAGCTCGGGGGTGTGGCGCAGCGCCG G CGGGGGTGGGCGGG  CGCGCCGGGTGGC  AGGTGTCAGCGGCGTCTGCATT
                         (BRE-)                                                   (MTE-)                          
    358 Human: S100A6 (6277, NM_014624) 
Mouse: S100a6 (20200, AK008241) 
 
           |-50      |-40      |-30      |-20      |-10      |1        |11       |21       |31       |41       
Human     GGACTTGGGCG  GGCGGGGT  GGGCTTGGCCGAGCTGGCCTCCGGGGCACCG A CCGCTATAAGGCCAGTCGGACTGCGACACAGCCCATCCCCTCGACCGCT
                       (BRE-)                                                                                   
Mouse     GGTGGGGTCGG  GGCGTGCC  AGCACTCCCTGGGCGGACCTCACGGATGCTG G   CCACTATA  AGGCCGGCCAGACTGCGACACATTCCATCCCCTCGACCACT
                       (BRE-)                                    (INR+)                                           
    359 Human: S100A8 (6279, M21005) 
Mouse: S100a8 (20201, BC078629) 
 
           |-50      |-40      |-30      |-20      |-10      |1        |11       |21       |31       |41       
Human     AGCTGGCCAAGCCTAACCGC  TATAAAA  AGGAGCTGCCTCTCAGCCCTGCA T G  TCTCTTGT  CAGCTGTCTTTCAGA  AGACCT  GGTAAGTGGGACTGTCTGG
                               (TATA+)                            (INR+)                  (DPE+)                    
Mouse     GCAGCTGGCCAAGCTTTCCTC  TATAAAA  GCAGCTGACACTTAGCC  TCACA T AT  CCTTTGTCAGCTCCGTCTTCA  AGACAT  CGTGTAAGTAGGGCTATGTG
                                (TATA+)                    (INR+)                        (DPE+)                     
    360 Human: S100A9 (6280, NM_002965) 
Mouse: S100a9 (20202, M83219) 
 
           |-50      |-40      |-30      |-20      |-10      |1        |11       |21       |31       |41       
Human     GGCCAAGTGCCCCAGTCAGGAG  CTGCCTA  TAAATGCCGAGCCT  GCACAGC T   CTGGCAAACACTCTGTGTGG  CTCCTCGGCTTTG  GTAAGTGAGCTGCCAG
                                 (TATA-)                 (INR+)                           (MTE-)                    
Mouse     TAACCATGTATCTAACAAGGAG  CTGCCTA  TAAATACTGGGCTTACACT  GC T CTTAC  CAACATCTGTGACTCT  TTAGCCTTGGTAA  GTAAGCTTCATTTTC
                                 (TATA-)                      (INR+)                       (MTE+)                   
    361 Human: S100A13 (6284, NM_005979) 
Mouse: S100a13 (20196, BC005687) 
 
           |-50      |-40      |-30      |-20      |-10      |1        |11       |21       |31       |41       
Human     CTGGCAGGGAGCCCGGGTCCAGACCTGAGGGCTCAGGGCAAGCGGCC  TGA T TGGG  CTGTCTCTGGTCTTCA  GAATCGACCACGG  AAATTTGACACCTCCG
                                                           (INR-)                       (MTE+)                    
Mouse     CTTCCTGATCAGGCTGTGTCCCAGTCGCGTGGCGCTGTGTTGGGATGG  CT A GTGAC  AGCAGGGGCGGTCCCC  TCAGCGGAGGCGG  AAACTTGACACTTCA
                                                            (INR-)                       (MTE+)                   
    362 Human: S100A13 (6284, BC070291) 
Mouse: S100a13 (20196, BC005687) 
 
           |-50      |-40      |-30      |-20      |-10      |1        |11       |21       |31       |41       
Human     GCGGCCCTTGTCTCTTTCCCAGCCTTGCTTGCCCCGTTCCTTTCTCTCCA G GCAGCAGTGGATGGTGCAGGGG  AAAGAGGTGGGAA  GGAGGTCCTGGGAG
                                                                                        (MTE+)                  
Mouse     GCTGTCCTGTGTTTCTTTCCGTTCTTGCTTGCACCACCTCCCTGAGCCTA G CGGGAAGTAGGTGGAAG  GGAAGGAGGTCGA  GGGAAGGGTGCTATGGTGC
                                                                                   (MTE-)                       
    363 Human: SARS (6301, NM_006513) 
Mouse: Sars1 (20226, NM_011319) 
 
           |-50      |-40      |-30      |-20      |-10      |1        |11       |21       |31       |41       
Human     TGCGGGCTCCAG  CTGCGCCT  GCGCAGGAAGGGCGGGTCAGCGCGCCGGC  G C AGTGCG  GCGGTCACAG  GCTGAGTGCTGCG  GCGCGATCCTTGCTTCCCTG
                        (BRE+)                                 (INR-)                 (MTE-)                        
Mouse     TCCCGGGCTGCC  GGGTGCCT  GCGCAGTGAGACCAGGTCAGCGCGCAGGC  G C AGTGCA  TCGTCCACCCGCCGCTT  ACAGTCCTCTCTT  AGTGTTAACTGGT
                        (BRE+)                                 (INR-)                        (MTE-)                 
    364 Human: XCL1 (6375, D63790) 
Mouse: Xcl1 (16963, AK079820) 
 
           |-50      |-40      |-30      |-20      |-10      |1        |11       |21       |31       |41       
Human     GGAGGCATAAAAGAGGTCCTCAAAGAGCCCGATCCTCACTCTCCTT  GCAC A GCT  CAGCAGGACC  TCAGCCATGAGAC  TTCT  CATCCT  GGCCCTCCTTGGC
                                                          (INR+)                 (MTE+)         (DPE-)              
Mouse     AGGGTAATAAAAGGGGCTCCTGGGGAGTCTGCTCCACATTCTTCTT  GCAC A GCC  CAGCAAGACC  TCAGCCATGAGAC  TTCTCCTCCTGACTTTCCTGGGA
                                                          (INR+)                 (MTE+)                           
    365 Human: SDHB (6390, BC007840) 
Mouse: Sdhb (67680, BC051934) 
 
           |-50      |-40      |-30      |-20      |-10      |1        |11       |21       |31       |41       
Human     GACGGGACACCGGCGGAGAGCGACCTCGGGGTTAAGGGGTGGGGCTGACG T CAGGAGCCAAGATGGCGGCGGTG  GTCGCCCTCTCCT  TGAGGCGCCGGTT
                                                                                         (MTE-)                 
Mouse     CCCACCGCGGTGTTCGACGGGATGCCGGAGGGGAAGGGGTGGGGCTGACG T CAGGAGCCAAAATG  GCGGCGACGGTCG  G  GGTCTC  CTTGAAGCGCGGCTT
                                                                                (MTE+)      (DPE-)                
    366 Human: SDHB (6390, U17296) 
Mouse: Sdhb (67680, BC051934) 
 
           |-50      |-40      |-30      |-20      |-10      |1        |11       |21       |31       |41       
Human     CGCTGTGCTTG  CCCCGCCT  TCCCTCCGCCCACCCGGGAAACCGGAAGCCG C CTCCCACTTGGTTGCTC  GTACGCGGCTAGT  G  GGTCCT  CAGTGGATGTAG
                       (BRE+)                                                        (MTE-)      (DPE-)             
Mouse     TGCTGCAC  ACACGCAC  TTCCTGTACATTGGCTCGGAGAAACCG  GAAGCGG C   CTTCCACTCGTTGGCGC  TTAGGCGGCTAGC    GGTCCT  CAGGGTGAGAGGC
                    (BRE+)                               (INR-)                        (MTE-)     (DPE-)              
    367 Human: SDHC (6391, NM_003001) 
Mouse: Sdhc (66052, AK012818) 
 
           |-50      |-40      |-30      |-20      |-10      |1        |11       |21       |31       |41       
Human     CGACCCCCAGCCG  GCGCGCCT  CCGCCCTCGGGTGGCGGGGCCGCCTGGCG   T CACTTCC  GTCCAGACCGGAACCCA  AGATGG  CTGCGCTGTTGCTGAGGTG
                         (BRE+)                                 (INR+)                    (DPE+)                    
Mouse     CGCCGGCT  CCGCCCCC  AAAGGCAGGGCCACACGGGGGGAGGAGCGATGCG   T CATTTCC  GTCCAGGCCGGAACTCA  AGATGG  CTGCGTTCTTGCTGAGGTG
                    (BRE+)                                      (INR+)                    (DPE+)                    
    368 Human: SELL (6402, NM_000655) 
Mouse: Sell (20343, M64440) 
 
           |-50      |-40      |-30      |-20      |-10      |1        |11       |21       |31       |41       
Human     AGGAGGATGTGAGACTGGGTTA  GAGAAAT  GAAAGAAAGCAAGGCTTTCTG T TG  ACATTCAG  TGCAGTCTACCT  GCAGCACAGCACA  CTCCCTTTGGGCAA
                                 (TATA+)                           (INR+)                   (MTE+)                  
Mouse     CGGAGGATGTGAGGCTGGGCTG  GAGAAAT  GAAAGAAAGCAAACC  CCACTG G T  GACATTGGCACATTCTTCC  TGAGCGCAGCACG  CCCTCCTTGTGCAAGA
                                 (TATA+)                  (INR+)                          (MTE+)                    
    369 Human: SELP (6403, NM_003005) 
Mouse: Selp (20344, AF031662) 
 
           |-50      |-40      |-30      |-20      |-10      |1        |11       |21       |31       |41       
Human     TTCTAAACAGCCTGACACTGAGGGGAGGCAGTGAGACTGTAAGC  AGTCTG G G  TTGGGCAGAAGGCAGAAAACCAGCAG  AGTCAC  AGAGGAGATGGTGAGT
                                                        (INR-)                             (DPE+)                 
Mouse     TGTTAGGCAGCCTGACATCAACGGGAGGCAGCAAAAGTATAAGA  AGTGTG G T  TTTGGCTAGAGGGAAGAAAGCC  AGACGG  ATCAGAGAGGACATGGTAAG
                                                        (INR-)                         (DPE+)                     
    370 Human: SELP (6403, AL022146) 
Mouse: Selp (20344, AF031662) 
 
           |-50      |-40      |-30      |-20      |-10      |1        |11       |21       |31       |41       
Human     TACTCTTGCATTATCAACATT  CTAACTT  CATGGGAAGGGCTGTGG  TGAGT T TC  TGGAATGTGAATAGGAAGTTGTTTTTCTAAACAGCCTGACACTGAGG
                                (TATA-)                    (INR+)                                                 
Mouse     TGTTTCTGCAGTATCAACAGG  CCAACTT  TAGGGGAAAGGCCGTGAT  ACAT T TCT  GGAAAGCGAATAGGAAGTTGTTTTGTTAGGCAGCCTGACATCAACG
                                (TATA-)                     (INR+)                                                
    371 Human: SFRS4 (6429, L14076) 
Mouse: Sfrs4 (57317, AK077648) 
 
           |-50      |-40      |-30      |-20      |-10      |1        |11       |21       |31       |41       
Human     GCTCGCGTA  CGTCGCCG  GGGTTCGGCTGCGTCCGTCCCGCCGCCCGCCCG T TGCCGCCGCCGCCGCTGCCGCCG  TGCTCTCGCTTTG  CCCGCCGCCGCCT
                     (BRE+)                                                                (MTE-)                 
Mouse     GCTCGCGTA  CGTCGCCG  GGCTTCGGCTGCGTCCGTCCCGCCGCCCGCCCG T TACCGCCGCCGCCGCGTTTTCCC  TTCGCCGGCCGCC  GCCTGAGGGGGGC
                     (BRE+)                                                                (MTE-)                 
    372 Human: SHC1 (6464, AK096169) 
Mouse: Shc1 (20416, AK036980) 
 
           |-50      |-40      |-30      |-20      |-10      |1        |11       |21       |31       |41       
Human     CCTGGGGAGGAGCCAGAAGCGCTGGGTCCAGCCTGTTCTCTTCTCAGCCA G A  GAAGAGGC  TCCACGTTGGGCGG  GGATGG  GGCCTGAAACTGTCTGGGTC
                                                                (INR-)                 (DPE+)                     
Mouse     CTCTGGGGAGGAGCCAGTAAGCTCTGGGTCCAGCCTGTACTCCTCAG  CCA G TAGA  GGTTTCCTAGCTGGGCTGTGCAA  GGATGT  GCTCTCAAACTGTCTA
                                                           (INR+)                          (DPE+)                 
    373 Human: SHC1 (6464, AF455141) 
Mouse: Shc1 (20416, AK036980) 
 
           |-50      |-40      |-30      |-20      |-10      |1        |11       |21       |31       |41       
Human     AAGGAGGGACCCTGAGGTAGAGGGTCAGGGGTTAGTGAGGCCG  GAAGTGA G   TGTAATAAAGTTTCTCCAGGGAGGCAGGGCCCGGGGAGAAAGTTGGAGC
                                                       (INR-)                                                   
Mouse     AAGGAGGGACAGCGAGGTAGGGGGTCAGGGGTTAGTGAGGCCG  GAAGTGA G   TGTAATAAAGTTTGTCTGGGGA  GGCCCAGGCTGGG  GTGAAAGTTGGGGC
                                                       (INR-)                             (MTE-)                  
    374 Human: ST3GAL3 (6487, AY051143) 
Mouse: St3gal3 (20441, AK005053) 
 
           |-50      |-40      |-30      |-20      |-10      |1        |11       |21       |31       |41       
Human     GGCGCCCGACCGG  GTCGCGGC  CGCGCGCTCCGCCCGCCGCTGCGTCC  CCA C TATG  GCGGCGCCCATGCAGC  CCAGCGCGTTGTG  GGCTCCCGCCGGGGTC
                         (BRE-)                              (INR+)                       (MTE+)                    
Mouse     CGGCCGCCGAGGCGGACCGCAGGATTGGTGGTCCCGCCGCTGCGT  CCCCA C CA  TGGCGGCGCCCGTGCAGCTTGCCG  CGTCCT  GGGCTCCCTCTGGGGTC
                                                         (INR+)                           (DPE-)                  
    375 Human: SIL (6491, Y07540) 
Mouse: Sil (20460, BC049865) 
 
           |-50      |-40      |-30      |-20      |-10      |1        |11       |21       |31       |41       
Human     AGCAGGCGT  GGCGCGGC  CTTCAGTTTCGCGAGCTTGTGTTTGCCGCC  TCA G TTCC  CGCGACCCCAACGTC  CCAGAGGCGGGGC  CGGAGTCGGCGGTGGCG
                     (BRE-)                                  (INR+)                      (MTE+)                     
Mouse     GCAGGCGCG  GCGCGCCC  TTTGGTTCCCTCCAGCGAGCGCTTGCCGCC  TCG G TTCC  CGCGACCCCAACGTC  CCAGAGGCGGGGC  CGCAGTCATCTGAGACG
                     (BRE+)                                  (INR+)                      (MTE+)                     
    376 Human: SLC2A1 (6513, NM_006516) 
Mouse: Slc2a1 (20525, D10229) 
 
           |-50      |-40      |-30      |-20      |-10      |1        |11       |21       |31       |41       
Human     GCCCCGCCCCGAGGTCCTGCCCACACACCCCTGACACACCGGCGTCGCCA G   CCAATGGC  CGGGGTCCTA  TAAACGCTACGGT  CCGCGCGCTCTCTGGCAA
                                                               (INR-)                 (MTE+)                      
Mouse     CGGACGTGCTCCCCAGG  CCCCGCCC  CCTTCGGGCCCACCTACACCCCAGA A   CCAATGGC  GGCGGTCCTATAA  AAAGGCAGCTCCG  CGCGCTCTCCCCCAA
                             (BRE+)                              (INR-)                    (MTE-)                   
    377 Human: SLC2A5 (6518, U05344) 
Mouse: Slc2a5 (56485, AF233337) 
 
           |-50      |-40      |-30      |-20      |-10      |1        |11       |21       |31       |41       
Human     TTCTGACTTAATCTGGC  TATAAAA  ATGCTATTGGCTGTTATTTGGCATGG C C  AAAGTGCA  CCCAGAATGTCTTCTCTCTCCATTCAGTGCACGCGTTACT
                            (TATA+)                               (INR-)                                          
Mouse     TTCTGACTTAGACTAGC  AATAAAA  ATGCAATTGGCTGAGAGTTGGCACGG C C  AAAGTGCA  GCCTAAATATCTTCCCTCCCGAACAGCACAGCTGGCACTT
                            (TATA+)                               (INR-)                                          
    378 Human: SLC6A9 (6536, NM_006934) 
Mouse: Slc6a9 (14664, AK014572) 
 
           |-50      |-40      |-30      |-20      |-10      |1        |11       |21       |31       |41       
Human     GGCGCGGC  GGGGCGGG  CGAACTCGGCGCGGCACAGAGCCTCGGGAGGCTG A TG  CAACTTTC  CCTTTAAG  AAAGCCACCTGGG  CGCACCGCGGTGCGGACC
                    (BRE-)                                         (INR+)               (MTE+)                      
Mouse     AACGCGGC  GGGGCGGG  AGGACTGGCGGCTGCAGAGAGCCTCGGGAGGCTG A TG  CAACTTTC  CCTTTAAG  AAAGCCACCTGGG  CGCACCGCGGTGCGGACC
                    (BRE-)                                         (INR+)               (MTE+)                      
    379 Human: SLC9A1 (6548, M96066) 
Mouse: Slc9a1 (20544, AA184599) 
 
           |-50      |-40      |-30      |-20      |-10      |1        |11       |21       |31       |41       
Human     CCTTCTCAGCCTGGGCTCCCAGCCCCCTCTCTCCTTTTCCTGGACTGGCT C TCACCCCCTTCGGTCCCCTTCCT  TTAGCTCAGGCTC  CCTACCCCTTCCT
                                                                                         (MTE+)                 
Mouse     ATCCCAGGCTGGCCTTCCAGCCTTCTCTCGCCTTTCCCTGCGGAGTGGGT C TC  ACCCTTTT  CCGTCT  TTAGCCCAGGCTC  CTTACCCCTCCCCTTTAGCC
                                                                 (INR+)             (MTE+)                        
    380 Human: SLC9A1 (6548, M96066) 
Mouse: Slc9a1 (20544, NM_016981) 
 
           |-50      |-40      |-30      |-20      |-10      |1        |11       |21       |31       |41       
Human     CGGCTGCTCGCTGGTGCC  TATAAGT  GACAGCGCCGGGCTCAGCTAGGCT  T C AGTCTG  CTGCCGCCGG  GGCCGGGGCTAGG  GTTAGTTCTGGAGCTGGACC
                             (TATA+)                           (INR+)                 (MTE-)                        
Mouse     CTGCTACTCGTTGGTGCC  TATAAAT  GGCTGCGCTGTGCTCAGCGAGGCA  T C AGTCCG  CTACCGCGGG  ACCCGGGGCTGTG  TCAGGTCTGGAAGTGGAGCT
                             (TATA+)                           (INR+)                 (MTE-)                        
    381 Human: SLC16A1 (6566, AJ438943) 
Mouse: Slc16a1 (20501, AK079379) 
 
           |-50      |-40      |-30      |-20      |-10      |1        |11       |21       |31       |41       
Human     TGCTCCGGCCCCGGCGGGT  TATAAGG  CAGCCTCGCTGGCCCGGCCAGAC  A A AGTGGT  GAGCTGCGAC  GTGACTGGCTAGC  TGCGTGGGTACTGGAACAAG
                              (TATA+)                          (INR-)                 (MTE-)                        
Mouse     TGCTCCGGCCCCCGCGGGT  TATAAGG  CAGCCGCGCCTGCCCCGCCAGAC  A A AGTGGC  GAGCTGCGACGTG  ACTGGTCGGTCGT  GTAGGTGCAGCAGCCAA
                              (TATA+)                          (INR-)                    (MTE-)                     
    382 Human: SLC16A1 (6566, AJ438943) 
Mouse: Slc16a1 (20501, AK079379) 
 
           |-50      |-40      |-30      |-20      |-10      |1        |11       |21       |31       |41       
Human     CGCGCGGGCGAGCTAGAG  GGCGCGCG  CGGCTGAAAGCGTGTGGAGGCGCG G GCTGCAGTTCGGATGTCTGT  GTGGCGGGGAGGG  GGCGGCGGCCGGGAGA
                              (BRE-)                                                    (MTE+)                    
Mouse     CGCCCGAGCGAGCGAGCG  GGCACGCG  CAGCCCGCAGCGTGTGGAGGCGC  G A GATGCC  GAGCTGGGAGTATGTATGGCAGGGAGGGGGCGGCGGCCGGGAA
                              (BRE-)                           (INR-)                                             
    383 Human: SNRPE (6635, M21253) 
Mouse: Snrpe (20643, AK019462) 
 
           |-50      |-40      |-30      |-20      |-10      |1        |11       |21       |31       |41       
Human     AGACGCCTCAGTTTGGCCTCCCTCGGAGGCCATGCAGCGAAGAAACG  TGA C TTCA  TGGCTAGAGGGGCATTTCCCAGGAGCCGCCCATGCCGCCGCGTGA
                                                           (INR+)                                               
Mouse     GTTGAGTCGGAAGGCCGCCGGCGGAGTCTATCAGCATAGAGAA  AAAGCGG C   TCCTGGCCTAGAGAGGCGTTCCCCA  GGACCG  GCCCGCGTCGCCGACGAC
                                                       (INR-)                            (DPE+)                   
    384 Human: SNRPE (6635, NM_003094) 
Mouse: Snrpe (20643, AK019462) 
 
           |-50      |-40      |-30      |-20      |-10      |1        |11       |21       |31       |41       
Human     GGCATTTCCCA  GGAGCCGC  CCATGCCGCCGCGTGACCTTCACACTT  CCGC T TCC  GGTTCTTTATTCCGG  AAGTTGCTCTCAG  AGGCAGCGTGCGGGTGTG
                       (BRE-)                               (INR+)                      (MTE-)                      
Mouse     AGGCGTTCCCCAGGACC  GGCCCGCG  TCGCCGACGACCACTGCACTT  CCGT T TCC  GGGTCTTGCTTCC  GGAAGTCGCTTTC  CGAGGCGGCCTTCCTACTCG
                             (BRE-)                         (INR+)                    (MTE-)                        
    385 Human: UAP1 (6675, BC009377) 
Mouse: Uap1 (107652, AK047566) 
 
           |-50      |-40      |-30      |-20      |-10      |1        |11       |21       |31       |41       
Human     CCCGCCCC  GGCGCCCC  GGGCCGCGGCCGCCTCCGCGTCCGCGTCGTCGTC T GTGCTCCCGGCGCTGACGTG  TCTGGGCGGTCGG  CTTCCACTCCTTCAGG
                    (BRE-)                                                              (MTE-)                    
Mouse     GTGACGTAAAG  AGGCTCCT  CCTTCCTGTCGCCGGTGCCCGCCCCTTTG  GC T GTTCT  CGGTCGCTGAC  GTGTCGGGCCGGC  AGGCTTCCGCTCCGCCCCTG
                       (BRE+)                                 (INR+)                  (MTE-)                        
    386 Human: UAP1 (6675, NM_003115) 
Mouse: Uap1 (107652, AK047566) 
 
           |-50      |-40      |-30      |-20      |-10      |1        |11       |21       |31       |41       
Human     TGGCCCCCGCTCCCGGC  CCGCCCCG  CCGCCGCGGCCCCCCGGATGAGGGT   A TATATTC  GGAGCGAGCGCG  GGACGCCGATGAG  TGGCCGCGCGGAAGGAG
                             (BRE+)                             (INR+)                   (MTE-)                     
Mouse     CGGCCGCTGCTCGTGGC  CCGCCCCG  CCGCCCCGGCCCCTCGGATGACAGT   A TATATTC  CAGGCG  GGCGCGGGACACG  GGCGAGTAGCCGAGCCGAAGGAG
                             (BRE+)                             (INR+)             (MTE+)                           
    387 Human: SPRR1A (6698, L05187) 
Mouse: Sprr1a (20753, NM_009264) 
 
           |-50      |-40      |-30      |-20      |-10      |1        |11       |21       |31       |41       
Human     GAGGCAGGGCTCATTCATCT  TATAAAA  GCCAGCTGGCCATTGCCT  TCACA C CA  AACCCAAGGGACCAC  ACAGCCCATTCTG  CTCCGTATACCAGGTAAGT
                               (TATA+)                     (INR+)                      (MTE+)                       
Mouse     TTCCTGGAGGTGTTCTTCT  TATAAAA  AGCCAGCTGCCCAATGCCT  GCACA C AG  AATCCACACCAACAG  AGAACCTGCTCTT  CTCTGAGTATTAGGTAAGT
                              (TATA+)                      (INR+)                      (MTE-)                       
    388 Human: SPRR1B (6699, M84757) 
Mouse: Sprr1b (20754, AK009752) 
 
           |-50      |-40      |-30      |-20      |-10      |1        |11       |21       |31       |41       
Human     GAGGCAGGGCTTGTTCCATG  CATAAAA  AGCCAGTTGGCTGGGAACACTAC A   CCAGTTCT  AAGGGACCATACAGA  GTATTCCTCTCTT  CACACCAGGTGAG
                               (TATA+)                           (INR+)                      (MTE-)                 
Mouse     CAGGCAGGTCTCATTCCTTC  TATAAAA  AGCCAGTGTGAAGAGGATA  CCAC A TCC  GATCCCAGCGACCACACTACC  TGTCCT  CCATATACCAGGTAAGTCT
                               (TATA+)                      (INR+)                        (DPE-)                    
    389 Human: SPRR3 (6707, AF077374) 
Mouse: Sprr3 (20766, NM_011478) 
 
           |-50      |-40      |-30      |-20      |-10      |1        |11       |21       |31       |41       
Human     AAGGCAGGGCTCATTTTCTA  TATAAAT  ACCAGCTAACAAGAGACTCTTCA   C CAGATCC  CAGAGGCTGAACACCTCGACCTTCTCTGCACAGCAGGTGAGT
                               (TATA+)                          (INR+)                                            
Mouse     AGGAAGGGCTCACTTTCTG  TATAAAA  ATCCAGCTGACCAGAAATCCCTCA   A CAAATCC  AAGAACCCAGTGATCTTCAG  CATCTA  AGGTAAGACTTCCCAT
                              (TATA+)                           (INR+)                       (DPE-)                 
    390 Human: SRM (6723, M64231) 
Mouse: Srm (20810, AK028359) 
 
           |-50      |-40      |-30      |-20      |-10      |1        |11       |21       |31       |41       
Human     CGCCGGCAGGCCCCGCC  CCGCGCCC  GGGTTAGGTTGCGGCGCGGGCGGCG G GCGGAGCTGGTCCCGTTGTGCT  GCGGCGCCGCGCG  GCCTGCAGTCCCGG
                             (BRE+)                                                       (MTE+)                  
Mouse     CCCTCCGTAGG  CCCCGCCC  CCGAGCGGGTTAGGTTGCGGCGCGGGCAGCG G GCAGAGCTGGTCACGTTGTGC  TGGGGGGGGTCCG  CGCGCCCTGCAGTCC
                       (BRE+)                                                            (MTE-)                   
    391 Human: SRP9 (6726, NM_003133) 
Mouse: Srp9 (27058, BC039648) 
 
           |-50      |-40      |-30      |-20      |-10      |1        |11       |21       |31       |41       
Human     CCTGACAACATCCCTCCTGCCCATTGGGCGAGCATGGACGGGGCTGT  CCA A TGGA  GCGAGGCGTTGGTGCGAG  GAGGCGCCGCCAT  CTTGGGGCTGCTGG
                                                           (INR-)                         (MTE+)                  
Mouse     CGACATCACGCCTCCTGCCCATTGGACAGGCGCGCGGCCGAGGCTGG  CCA A TGGC  GTGAGCGCTCGTGAGCCTC  CGTCTT  TGCGCCGCCGGAGTGCAGCG
                                                           (INR-)                      (DPE-)                     
    392 Human: SSA2 (6738, NM_004600) 
Mouse: Ssa2 (20822, AF065398) 
 
           |-50      |-40      |-30      |-20      |-10      |1        |11       |21       |31       |41       
Human     CGGGCGCCGTCACCTGCA  GCGCGACT  GAGCGCGGGAGGACGCTCTAGCCA C CCTAGAGACATCGAAACTCTTCCCAGGCCTTGCAGCACCCGTAGCGGAT
                              (BRE+)                                                                            
Mouse     CGGGCGCCGTCACCTGCC  GCGCGCCG  GAGCACAGAAGGACGCTCTAGCCA C CCGGGAGACGTCGAAACGCCTC  TCCTGCCTCTCAG  GATCCGTAGCGCCC
                              (BRE+)                                                      (MTE-)                  
    393 Human: SSA2 (6738, BC036658) 
Mouse: Ssa2 (20822, AF065398) 
 
           |-50      |-40      |-30      |-20      |-10      |1        |11       |21       |31       |41       
Human     CCCGGCCGGACCATTCCTCAGGGTGGGCCCTTTCCGAAGCCGGGACCGCT C CTGCTTGTCGGCATCGCTCCCCACAGGCCGACGTCGAGAGGGCCTGCTT
                                                                                                              
Mouse     GGCCTAGTTGCTCCGGGAGGATGGAATCTTGGGGAACTCAGGTCAACTCT C   CCACTTGC  AAAGCTCCTGG  CGAGCGAACGTGC  CAAGGCCCCTTTTCACT
                                                               (INR+)                  (MTE+)                     
    394 Human: SSR2 (6746, CR600571) 
Mouse: Ssr2 (66256, AK005340) 
 
           |-50      |-40      |-30      |-20      |-10      |1        |11       |21       |31       |41       
Human     GAACTACAACTCCCACAAGGCATTGGGCGGAACACCACGGTTTCCGG  CCA G TGCT  TATTTCCGG  TCTTTCGGATGCT  GACGCTCTCTTCCTGTCTTTGTG
                                                           (INR+)                (MTE-)                           
Mouse     GAACTACAACATCCATAAGGCACAGGAAGGGACATCAGGGTTTCCGG  TCA G ATCC  GTTTCCGGTCTTTCGGAGGCTGACGTTGTTTTCCGGTTGTCTGTG
                                                           (INR+)                                               
    395 Human: STXBP3 (6814, D63506) 
Mouse: Stxbp3 (20912, BB635250) 
 
           |-50      |-40      |-30      |-20      |-10      |1        |11       |21       |31       |41       
Human     CATTCCCCTGCAGCAAGG  GGCGGGGC  CACCCCAACGCCGCTTCTGCGGCC   A AAGTAGG  TTGGGAGTGGAA  GGTGGTGGCTGCT  GCTCCGCAGTGTCGGGA
                              (BRE-)                            (INR-)                   (MTE-)                     
Mouse     CTCCCCACCGCGAGGGGAAGGGCCCGCCCCCGAGCGCAGCTTTCAGGGCC   A AAGTAGG  TTGCGAGTGGGA  GGCGGTGGCTGTA  GCTCCACGGCTGGGGGA
                                                              (INR-)                   (MTE-)                     
    396 Human: XCL2 (6846, D63789) 
Mouse: Xcl1 (16963, AK079820) 
 
           |-50      |-40      |-30      |-20      |-10      |1        |11       |21       |31       |41       
Human     GGAGGCATAAAAGAGATCCTCAAAGAGCCCGATCCTCACTCTCCCT  GCAC A GCT  CAGCGGGACC  TCAGCCATGAGAC  TTCT  CATCCT  GGCCCTCCTTGGC
                                                          (INR+)                 (MTE+)         (DPE-)              
Mouse     AGGGTAATAAAAGGGGCTCCTGGGGAGTCTGCTCCACATTCTTCTT  GCAC A GCC  CAGCAAGACC  TCAGCCATGAGAC  TTCTCCTCCTGACTTTCCTGGGA
                                                          (INR+)                 (MTE+)                           
    397 Human: TAF12 (6883, NM_005644) 
Mouse: Taf12 (66464, AK007811) 
 
           |-50      |-40      |-30      |-20      |-10      |1        |11       |21       |31       |41       
Human     GCGTTTCCGGGTTGGA  GGCGGGGA  GCGGTGCCGGAAGTAGTCGGGGAGGG C CGCGCAGTCGGACCGGCTGCTG  AGACGA  ACGCTTCACTGGGGCAGTCTC
                            (BRE-)                                                    (DPE+)                      
Mouse     GCGTTTCCGGGTTGGA  GGCGGGGA  GCGGTGCCGGAAGTGCTTGGGGAGGG C CGCGCAGTCGGACCGGCTGCTG  AGACGA  AAGCTTCACTGGGGCAGTCTC
                            (BRE-)                                                    (DPE+)                      
    398 Human: TAF13 (6884, NM_005645) 
Mouse: Taf13 (99730, AI530376) 
 
           |-50      |-40      |-30      |-20      |-10      |1        |11       |21       |31       |41       
Human     GGCCCAGCTACGGCC  CCGCGCGT  CACCGGAAGTGAGGCGGCGGAAGT  AGT G TAAC  CAGCTGGGAG  CCAGCCGGCAGGA  CGCTGTGAGTTGGCGTGCTAGT
                           (BRE+)                            (INR-)                 (MTE+)                          
Mouse     CGCCTCTCTGCG  CAACGCTG  GCCAGGCCGGAAGTGACGTACGTATCTTAT A   AAACTCGC  CAGCCGCCGG  TGGGCGGGTTCAG  AAGGCTGGCGAGCAGCTA
                        (BRE+)                                   (INR-)                 (MTE-)                      
    399 Human: CNTN2 (6900, X92681) 
Mouse: Cntn2 (21367, BY782090) 
 
           |-50      |-40      |-30      |-20      |-10      |1        |11       |21       |31       |41       
Human     GAGGAGCACATCTTGATGCAGAGATGCTGCAGTGGCTCCGGGCGCGC  TCA C ACAC  ACGCGCCCTCACCCG  CCACCGCCGCCGC  GGCCGCCGCCGCACCCG
                                                           (INR+)                      (MTE+)                     
Mouse     GAGGAGCGCATCCTGATGCAGAGATGCTGCAGTGGCTCGGGGCGCGCAC  A C ACATAC  GCACCCTCGCC  CGCTGCCGCCACG  CCAGGACAGCCAGTGGCTA
                                                             (INR+)                  (MTE-)                       
    400 Human: TCEB3 (6924, L47345) 
Mouse: Tceb3 (27224, AK036592) 
 
           |-50      |-40      |-30      |-20      |-10      |1        |11       |21       |31       |41       
Human     GCGCTTCCGTCTCCGCCGGGGATGCACGGAGGGCGGAGCTGCGGCCCGAG G ACGCGACGCGAGCCCAGTTCCGGCGAGGAGGCCGCGCCAGTGACAGCGA
                                                                                                              
Mouse     GCGCTTCCGTCTCCG  CCGGGCCC  TCACGGAGGGCGGAGTTGCGGCCCGAG G ACGCGACGCGAGCCCAGTTCCGGCGAGGAGGCCGCGCCAGTGACAGCGA
                           (BRE+)                                                                               
    401 Human: TCFL1 (6944, CR608392) 
Mouse: Tcfl1 (21427, AA110353) 
 
           |-50      |-40      |-30      |-20      |-10      |1        |11       |21       |31       |41       
Human     CTTCTGCCTGTAAACGCCCGACTTCCGCCGCTGGTGGCCACCCGCAG  GTA G TGAT  GTCGAGCGTCGAGCTCCC  AAAACCGAGCTGG  TGAGGGGCTGCAGG
                                                           (INR-)                         (MTE+)                  
Mouse     CTTTTGCTTGCAGACGCTGAACTTCCGTCATTGACGGTCGCCCGCAGGTA   A TAGTGTC  GAGTGCCGATCTGCG  AGAACCCCGAGCT  GGGCCAGGGGCTGA
                                                              (INR-)                      (MTE+)                  
    402 Human: TGFB2 (7042, M87843) 
Mouse: Tgfb2 (21808, NM_009367) 
 
           |-50      |-40      |-30      |-20      |-10      |1        |11       |21       |31       |41       
Human     ACGTGGTTCAGAGAGAACT  TATAAAT  CTCCCCTCCCCGGCAAGATCGTGA T GT  TATCTGCT  GGCAGCAG  AAGGTTCGCTCCG  AGCGGAGCTCCAGAAGCT
                              (TATA+)                              (INR-)               (MTE-)                      
Mouse     CACGTGGTTTGGGGAGCACA  TATAAAA  CTCTGCTCCGGGCAGAATCGTGA T GT  TATCTGCT  GGCAG  CAGGTTTGCTCGG  AGCAGAGCTGCTGAAACTGCC
                               (TATA+)                             (INR-)            (MTE-)                         
    403 Human: TGFB2 (7042, NM_003238) 
Mouse: Tgfb2 (21808, NM_009367) 
 
           |-50      |-40      |-30      |-20      |-10      |1        |11       |21       |31       |41       
Human     GGGCTCGCCCCCAGC  GCGCGCAC  ACGCACACACACACACACAC  ACACACA C   GCACGCACACACGTGTGCG  CTTCTCTGCTCCG  GAGCTGCTGCTGCTCCT
                           (BRE+)                        (INR+)                          (MTE-)                     
Mouse     AGGCTCGCCCTC  GGCGCGCG  CGCACGCACGCGCGCACACGCGC  ACACATC C   ACACGCACACTCATCCACACACGTGTGGAAGGCAGGGCCGAGCCGCTCG
                        (BRE-)                           (INR+)                                                   
    404 Human: TGFB2 (7042, AY438979) 
Mouse: Tgfb2 (21808, NM_009367) 
 
           |-50      |-40      |-30      |-20      |-10      |1        |11       |21       |31       |41       
Human     TTAATATTTCCACTTTTGGAACTACTGGCCTTTTCTTTTTAAAG  GAATTC A A  GCAGGATACGTTTTTCTGTTGGGCATTGACTAGATTGTTTGCAAAAGT
                                                        (INR-)                                                  
Mouse     TAATATCTCCACGTTGGGAACGCGTTGCATTTTCTTTTTTAAA  GGAATCC C   AGCCAGGGACGTTTTTCTATTGGGCAT  TAACTT  TCGACTGCTTTGCAAA
                                                       (INR-)                              (DPE-)                 
    405 Human: THBS3 (7059, NM_007112) 
Mouse: Thbs3 (21827, AY115108) 
 
           |-50      |-40      |-30      |-20      |-10      |1        |11       |21       |31       |41       
Human     CCTGCTCTCTCCGCCTAGCCTTTTCCCCTCCCAGCTGCCTGCCTGCCAGG G   GTAGTGAG  CCGGC  TGAGAGGCATGG  A  GACGC  AGGAACTTCGGGGGGCCC
                                                               (INR-)            (MTE+)    (DPE+)                   
Mouse     TCCTGCTCTCCGCC  TAGTCTT  TTTCCCTCCCAGTCGCCTTGCCTGCCAGG G   GTAGTGAA  CCGGC  TAAGCGGCATGGA  GAAGCCGGAACTTTGGGGTGTCC
                         (TATA-)                                 (INR-)            (MTE+)                           
    406 Human: THBS3 (7059, NM_007112) 
Mouse: Thbs3 (21827, AY115108) 
 
           |-50      |-40      |-30      |-20      |-10      |1        |11       |21       |31       |41       
Human     CGTTGCGGTCAGGG  GGCCTGGG  GAGATCCCCGGGGAGGCGAGG  CTTCTTC T   GGCCCGACTGGCAGCTGAACT  GCGGGGGACTGGG  CCGCGGGCCTCGGGG
                          (BRE-)                         (INR+)                            (MTE-)                   
Mouse     CGCCTCCCTG  GCCGCCGG  GGCTTTGGGGTCTGGGGAGGCGTGGCTCTTGG C   GGCCTGAG  CCGCGGCGGGGCTG  TGAGAGGTGGGGC  ATCGGCCTTCTGGG
                      (BRE-)                                     (INR-)                     (MTE+)                  
    407 Human: TIE1 (7075, NM_005424) 
Mouse: Tie1 (21846, NM_011587) 
 
           |-50      |-40      |-30      |-20      |-10      |1        |11       |21       |31       |41       
Human     CCAAGCCACCTCATTTCCTCTTCCTCCCCAGCACCGACCCACACTGAC  CA A CACAG  GCTGAGCAGTCAGGCCCACAG  CATCTG  ACCCCAGGCCCAGCTCG
                                                            (INR+)                        (DPE-)                  
Mouse     ACCCTAACCATCATTTCCTCTTCCTCCCCAGCACCGAGGTGCACT  GAGCT G GA  CAGGCTGAACAC  TCAGACCCACAG  C  AACTG  ACCCCGGGCCCAGCTGG
                                                         (INR-)                   (MTE+)    (DPE-)                  
    408 Human: TNNI1 (7135, NM_003281) 
Mouse: Tnni1 (21952, AJ242874) 
 
           |-50      |-40      |-30      |-20      |-10      |1        |11       |21       |31       |41       
Human     GGGGGCAGTGGGTCTG  TTCTATT  TTTACCAGCCAGTTGCTGCTGGAC  ACA G TTTT  CATAGCCTCC  CCTCGGCTCTGCC  CCTCACAGTCTGCAGTCTACGG
                           (TATA-)                           (INR+)                 (MTE-)                          
Mouse     GGAGGGGCAGCGGCTG  TTCTATT  TTTACTGGCCAGTTGCCGGAGGCCAC  G G TTTTCA  TAGCCTGCCC  TCAGCTCTGCCCC  CACACTCTGCAGTCTGTGGT
                           (TATA-)                             (INR-)                 (MTE+)                        
    409 Human: TNNT2 (7139, X79859) 
Mouse: Tnnt2 (21956, BM125290) 
 
           |-50      |-40      |-30      |-20      |-10      |1        |11       |21       |31       |41       
Human     TATTGCCTGTTCAGGGGCAAGGAACGAAGTGGACATCAGCAGGTGGCCTT G CTGCCATGTGGGTGTCA  CTATCTCCCCCAG  CAGGGGAGAAAACAGGCTT
                                                                                   (MTE+)                       
Mouse     GGCTTGCCTGCTCGGGGACAGGGAACTGGGGGACACCAGTTGGCTGCCTT   G CTGTGTT  GTTCAGG  TGTCATGGTTGCT  TCTTTGCAGAGGAAGAAATTAG
                                                              (INR-)              (MTE-)                          
    410 Human: TNNT2 (7139, NM_000364) 
Mouse: Tnnt2 (21956, NM_011619) 
 
           |-50      |-40      |-30      |-20      |-10      |1        |11       |21       |31       |41       
Human     CCCCAGCCCACATGCCTG  CTTAAAG  CCCTCTCCATCCTCTGCCTCAC  CCA G TCCC  CGCTGAGAC  TGAGCAGACGCCT  CCAG  GATCTG  TCGGCAGCTGCTG
                             (TATA+)                         (INR+)                (MTE+)         (DPE-)              
Mouse     CCCAGCCCACATGCCTG  CTTAAAG  CTCTCCCCATGCCCTGCCCTCAG  CCA G TCCC  TGCTGAGGC  TGAACAGATACCT  CA  AGACCT  GTGTGCAGTCCCTGT
                            (TATA+)                          (INR+)                (MTE+)       (DPE+)                
    411 Human: TP53BP2 (7159, NM_005426) 
Mouse: Trp53bp2 (209456, BY020445) 
 
           |-50      |-40      |-30      |-20      |-10      |1        |11       |21       |31       |41       
Human     CGGCGCGG  GGGGCGGA  GCCGGCACGGGCTCGGCTGGGGCCCGACCCGGGA   T TAGTTGG  TTTCGGAGCGGAGGAG  GGAGCCCCGACCG  TCACGAGCGTCGA
                    (BRE-)                                      (INR+)                       (MTE+)                 
Mouse     GGCTTCGTGGGG  GGCGGGGC  CAGCGCGCCTCGGTGGGGGCCCGGCCGGGA   T TAGTTGG  TTTCGGCGAGAAGGAGGAGGAGGAGGTGGGAGTCGCGAGCGC
                        (BRE-)                                  (INR+)                                            
    412 Human: TPM3 (7170, CR617822) 
Mouse: Tpm3 (59069, BY264985) 
 
           |-50      |-40      |-30      |-20      |-10      |1        |11       |21       |31       |41       
Human     AGGGCGCGGCAGAGGGACGTCACATCCGGGCGGGTTGGTGAGTTCCG  GTA T TTCA  GGGCGTAGCAGGCGGAAGTAAGGGTGAGAGGAGGCTGCAACGCCG
                                                           (INR+)                                               
Mouse     ACGCGCGGTGGCGGC  GGCGTCAC  ATCCCGGCGTATGAGTGAGTTCCG  GTA T TTCA  GGGCGGAGCAGGCGGAAGTAGGGTGAAAAGCGGCTACAACGCCGA
                           (BRE-)                            (INR+)                                               
    413 Human: TPM3 (7170, AK056997) 
Mouse: Tpm3 (59069, NM_022314) 
 
           |-50      |-40      |-30      |-20      |-10      |1        |11       |21       |31       |41       
Human     GGTGGAGCTATGAGCACA  GATAAAG  ACTCAAGTCTGGGGACCTCCTGG  TC A CTCAG  GCAGCAGCCCCTTCTTTCTTGCCCCAGTCTCCAGTTCTCCAGTG
                             (TATA+)                          (INR+)                                              
Mouse     GGTGGAGCCGCAAGCACA  GATAAAG  GTTCAAACCTGGGGAACTT  TGAGTG A C  CCGGGTGGCAGCCCTTCCTCTCTGGCCCCAGCCTCCAGTTCTCCAGTG
                             (TATA+)                      (INR-)                                                  
    414 Human: TPR (7175, X63105) 
Mouse: Tpr (108989, AK088078) 
 
           |-50      |-40      |-30      |-20      |-10      |1        |11       |21       |31       |41       
Human     CTCTGAGCTCGGGTTCCCAGGGTTCGTAGCTTCCAACGGCTGCGCGC  GCA C TTCG  GTCGCGGGCG  GTGAGGTGCTGTT  GCTGAAACGCTGCCGCTGAGGG
                                                           (INR+)                 (MTE-)                          
Mouse     CTGTGAACTCGCTTTCCC  AGCGTGCC  TAGCTCCCGGTGGCTGCGTGCGC  A G AGAGGG  CGCGGCGGCGC  GGCTCTGGCTGCA  CCTAACGCGGGACTCTCTT
                              (BRE-)                           (INR-)                  (MTE-)                       
    415 Human: CCT3 (7203, CR618496) 
Mouse: Cct3 (12462, AK089548) 
 
           |-50      |-40      |-30      |-20      |-10      |1        |11       |21       |31       |41       
Human     TGCCGATCAATGAGATG  GGTGCGGT  GATTGGCGACTACCTTGAGAGTAGC G GGTTGAGGTGTAAGCCCTGAGG  AGGCAGCGTTTTC  TGGGCTTCTGTCTG
                             (BRE-)                                                       (MTE-)                  
Mouse     TGTCAATCAATAAAATGAGTTTGGTGATTGGCGGCACTTCAGAGGTTAGC A GGTTTCTGTGTCATTCCTGAGG  AGGCAGCGTTTTC  TTCGCTGCTCTTCT
                                                                                        (MTE-)                  
    416 Human: TSHB (7252, M23669) 
Mouse: Tshb (22094, M14499) 
 
           |-50      |-40      |-30      |-20      |-10      |1        |11       |21       |31       |41       
Human     AATCAAATGCAATTG  TATAAAC  AAGAAGATCAGAGGGATTATCCTGAAG  G G TATAAA  ATGAACCAAGAGCTTTTAGTTTGGGTCACCACAGCATCTGCTC
                          (TATA+)                              (INR-)                                             
Mouse     TCGAATGCAATTATATAAAC  AAGAAGA  TCAGAGGGGAATTATCCTGAAG  G G TATAAA  ATGAACGG  AGAGTGGGTCATC  ACAGCAGTAACTCACTCATGCA
                               (TATA+)                         (INR-)               (MTE+)                          
    417 Human: TTC4 (7268, NM_004623) 
Mouse: Ttc4 (72354, AK005923) 
 
           |-50      |-40      |-30      |-20      |-10      |1        |11       |21       |31       |41       
Human     ACGCCCCCAGGCCGGCGGAACCCAAGGCGCGGGGACCTGCGCTGCCGGGA   G GATTGAG  GGAAGG  GAACCCGCGCCGC  CGGAAGGAGCCGCTCGCTTCACG
                                                              (INR-)             (MTE+)                           
Mouse     ACGCCCCCAGGCCGGCGGAACCCGAGAGGCATAGCCTCGCGCAGCCC  GGA C TGCA  AAAGGAAGG  GCATCCCTGCCGC  CGGAAGGAGCGGCGCGTTTCTTG
                                                           (INR-)                (MTE+)                           
    418 Human: TNFSF4 (7292, AB042987) 
Mouse: Tnfsf4 (22164, NM_009452) 
 
           |-50      |-40      |-30      |-20      |-10      |1        |11       |21       |31       |41       
Human     ACGTTCCCCTTTTCCATATCTTCATCTTCCCTCTACCCAGATTGTG  AAGA T GGA  AAGGGTCCAACCCCTGGAAGAGAATGTGGGAAATGCAGCCAGGCCA
                                                          (INR-)                                                
Mouse     CCCAGGTT  CCCCGCCA  CAGCTGTATCTCCTCTGCACCCCGACTGCA  GAGA T GGA  AGGGGAAGGGGT  TCAACCCCTGGAT  GAGAATCTGGAAAACGGATCA
                    (BRE+)                                  (INR-)                   (MTE+)                         
    419 Human: TNFSF4 (7292, NM_003326) 
Mouse: Tnfsf4 (22164, NM_009452) 
 
           |-50      |-40      |-30      |-20      |-10      |1        |11       |21       |31       |41       
Human     TGCCTGCAAAAGTTGCAGC  GTTAAAA  CTGAGAGAGTCCGCTTTGCTCTT  T C AATCGC  CTTTTATCTCTGGCCCTGGGACCTTTGCCTATTTTCTGATTGA
                              (TATA+)                          (INR+)                                             
Mouse     CGCCTGCAAAACCTGCAGA  GTTAAAA  CGAAGAGACTCTGCTCTGCTCCT  T C AATTGC  TTTTTGTCTCCTGTTCTGGGACCTT  TATCTT  CTGACCCGCAGG
                              (TATA+)                          (INR+)                            (DPE-)             
    420 Human: TNFRSF4 (7293, AJ277151) 
Mouse: Tnfrsf4 (22163, NM_011659) 
 
           |-50      |-40      |-30      |-20      |-10      |1        |11       |21       |31       |41       
Human     CCCTCCCCTCCTCCA  CAGTCCT  TATAGCCACACCCCGCAAGGAAAAC  CCA G ACTC  TGGCGACAGCAGAGACGA  GGATGT  GCGTGGGGGCTCGGCGGCTGG
                          (TATA-)                            (INR+)                     (DPE+)                      
Mouse     TGCCCCACCTCCATAGTT  CTTATAG  CCACACCCTGCAAGGAAAAACC  CCA G ACTC  CTGTGAAGGCAGAAAGC  AGACAA  GGATGTATGTGTGGGTTCAGCA
                             (TATA+)                         (INR+)                    (DPE+)                       
    421 Human: UCK2 (7371, CR608443) 
Mouse: Uck2 (80914, CA466474) 
 
           |-50      |-40      |-30      |-20      |-10      |1        |11       |21       |31       |41       
Human     CGCTGCCCGCCCGGATTGGAGCAGCAGCATCACGCTGACCTCTGCCTGG  G A TGTAAA  CCGGACCAGCCGCTGCGGGCAAAGGAAGGCTCTTGGCTCCTTC
                                                             (INR-)                                             
Mouse     CGCAGCCCGCCCGGATTGGAGCAGCCTCATCACGCTGACCTCTGCCTGG  G A TGTAAA  CCCCACGGGCCGCCACGGGCACAGGAGAGCTCTCGGCACCTCC
                                                             (INR-)                                             
    422 Human: UROD (7389, NM_000374) 
Mouse: Urod (22275, BC008109) 
 
           |-50      |-40      |-30      |-20      |-10      |1        |11       |21       |31       |41       
Human     GGACTGGGGGGCAGGCTCAGATT  CAGGTTA  AATTGTGGATTGAGCTC  GCA G TTAC  AGACAGCTGACCATG  GAAGCGAATGGGT  TGGGGTGAGTTCTCCAG
                                  (TATA-)                    (INR+)                      (MTE+)                     
Mouse     GGGGACTGGGGTGTC  GGGGCGGA  GCCTCAGATTAAAGCGGAGACCGC  TCA G TTAC  TGTTTACCATGGAGG  CGAACGGGTTCGG  GTGAGTTTTCTGCTGCA
                           (BRE-)                            (INR+)                      (MTE+)                     
    423 Human: USF1 (7391, AY593992) 
Mouse: Usf1 (22278, BC049784) 
 
           |-50      |-40      |-30      |-20      |-10      |1        |11       |21       |31       |41       
Human     TCCTTTTTCAACATTTTCCTCGGACGCGGTCTTTCCGAGGCTTATCCATT G A  AAATTTTC  CTTGGATAGGAAAGGTTTGGA  GGACCT  TATGGGTAGAGAA
                                                                (INR+)                        (DPE+)              
Mouse     TTGTTAACGTTCTTCTTTGCACACGGTCTTTCCGAGGCGCATTATCCGCT   A AACTAAG  GTTTTTCTTTGATTAGAAAGGTTTGGAAGATCTTGCGGGTAG
                                                              (INR-)                                            
    424 Human: USF1 (7391, NM_007122) 
Mouse: Usf1 (22278, BC049784) 
 
           |-50      |-40      |-30      |-20      |-10      |1        |11       |21       |31       |41       
Human     TGAGTTTGAGTTTATG  GGCGGGGC  GAGAGCGACACTTCCGCCCCTCAC  CA A CATGG  CCGCGGGCTG  GAAGTGCGCATGA  GCAGCTGTCTATGGAGATACC
                            (BRE-)                            (INR+)                 (MTE+)                         
Mouse     GGGCCGTCCGTCGGCTG  GGCGGGTC  GAGGGCACACTTCCGCCCCTCAC  CA A CATGG  CCGCAGGCTG  GGACTGCGCATGA  GCAGCTGTCTATGGAGAGACC
                             (BRE-)                           (INR+)                 (MTE-)                         
    425 Human: USP1 (7398, BC040140) 
Mouse: Usp1 (230484, AK080882) 
 
           |-50      |-40      |-30      |-20      |-10      |1        |11       |21       |31       |41       
Human     GTTCAATCGCAGCGCT  GGCGCGGG  CGGAGGCTAAAACACGGGGGTCCTG  A G ACTGAG  GAAAACGCGCCAAGTTCCCCTCGGTGGCGGAGTGCTAAAGACC
                            (BRE-)                             (INR-)                                             
Mouse     CTCCAGCCGCGACGCT  GGCGCGGG  CGGGGGTTCACTCGCGGGGGTCCTG  A G ACTGAG  GAAAACGCGCCAAGTTCCCCCTCGGCGGCCGTGCGGGGACCCT
                            (BRE-)                             (INR-)                                             
    426 Human: WNT2B (7482, NM_024494) 
Mouse: Wnt2b (22414, AK038400) 
 
           |-50      |-40      |-30      |-20      |-10      |1        |11       |21       |31       |41       
Human     CCGAAGGGGCTGTCCGCACACTAGGCCCGCAGCTCCCTTCAGCGCC  GCAG A CCC  CCTGACACCGCACCCGGTCCTCAGGCAGCGCGCCCCAGACCCCGGG
                                                          (INR+)                                                
Mouse     CCGCAGGGGCTGTC  GGCGCGCT  CTGGCCCCCGCCGCACTCCGCGCCCCGG A G  CCACTGAC  ACCGCACC  CGACCGCCCACAC  CCGGCTCAGCGCTCGTCGG
                          (BRE-)                                  (INR+)               (MTE+)                       
    427 Human: ZBTB17 (7709, NM_003443) 
Mouse: Zbtb17 (22642, AA589413) 
 
           |-50      |-40      |-30      |-20      |-10      |1        |11       |21       |31       |41       
Human     GCCGGAAGTTGCAGCCGGGAAGCCTGCGAGGTCGGTTCCGCCCGACTC  TA A CATGG  CGGCGCCC  TTTGTCTGCTCTG  GAGTGCCGTCCCCGGCCTTCTCG
                                                            (INR+)               (MTE-)                           
Mouse     GACGGAAGTCCCAGCCGGGAAGCCAGCGAGGTCGGTTCCGCCCGGTTC  TA A CATGG  CGGCGCCCTTTGTCTGCT  GTGAAGCGCTGCC  CCCGGCCTTCTCG
                                                            (INR+)                         (MTE-)                 
    428 Human: LUZP1 (7798, NM_033631) 
Mouse: Luzp1 (269593, AK012514) 
 
           |-50      |-40      |-30      |-20      |-10      |1        |11       |21       |31       |41       
Human     GGCCCCGCCCCTCATTGTCAGTCCCGCGGAGCCAGAGGAGGCGCCGGTCG C TGGCAGCTGAGCGGCGGA  ACCCTTCGCTGGC  GGGAGCGCGGTTCGGGGC
                                                                                    (MTE-)                      
Mouse     GGCCCCGCCCCTCATTGTCAGTCTGGGGGAGCCTGAGCAGGCGCCGGCCG C TGGGAGCGGAGCGCCGGATCCT  CCGGCGGCGGGAG  CGCGGGCCGCAGCC
                                                                                        (MTE+)                  
    429 Human: DNALI1 (7802, AF006386) 
Mouse: Dnali1 (75563, NM_175223) 
 
           |-50      |-40      |-30      |-20      |-10      |1        |11       |21       |31       |41       
Human     GCCAAGAAGCGCG  GGGGCGGG  AGTGAGGGGGCGGTTTCCATGGTGACG  GC A AACAA  GGCCCACACTGGACAGG  GCAGCTGCTGGGT  TGCTACTCTCGCCT
                         (BRE-)                               (INR+)                        (MTE+)                  
Mouse     CCCCGCCCCATCCATGG  GGCAAGGA  GAGGAGGGGTTTCCATGGTGATC  GC A AACAA  GACTCCAC  AGGGGCCGCTACG  G  AGTTGT  TACCCCTACTGCCACC
                             (BRE-)                           (INR+)               (MTE-)      (DPE+)                 
    430 Human: DNALI1 (7802, BX647913) 
Mouse: Dnali1 (75563, NM_175223) 
 
           |-50      |-40      |-30      |-20      |-10      |1        |11       |21       |31       |41       
Human     GGCCCACACTGGACAGGGCAGCTGCTGGGTTGCTACTCTCGCCTCCGCCA T GATTCCGCCCGCAG  ACTCTTTGCTCAA  GTACGACACCCCAGTGCTGGTG
                                                                                (MTE-)                          
Mouse     AACAAGACTCCACA  GGGGCCGC  TACGGAGTTGTTACCCCTACTGCCACCA T GATACCCCCAGCAG  ACTCTCTGCTCAA  ATATGACACCCCGGTGTTGGTG
                          (BRE-)                                                  (MTE-)                          
    431 Human: LRP8 (7804, D86389) 
Mouse: Lrp8 (16975, AK038580) 
 
           |-50      |-40      |-30      |-20      |-10      |1        |11       |21       |31       |41       
Human     GCGGCAGGGGG  AGCGTGGG  GCGGAGGCGGCAGCGGGAGGGAGCGCGCGCG C TGGCGGCGGCCGCCCAGGGCCGGGGCCGCGCGCCCAGCCTGAGCCCGCC
                       (BRE-)                                                                                   
Mouse     GCGGCGGGAGG  AGCGTGGG  GCGGAGGCGGCGGCGGGAGGGAGCGCGCGCG C CGGCGGCGGCTGCCCAGGGCCGGGGCCGCGCGCCCAGCCTGAGCCCGCC
                       (BRE-)                                                                                   
    432 Human: LAPTM5 (7805, NM_006762) 
Mouse: Laptm5 (16792, BY204278) 
 
           |-50      |-40      |-30      |-20      |-10      |1        |11       |21       |31       |41       
Human     GTCCCTGCCTGTCTAAGGGGCAGGAAGGAAGTGAGGAGGGCAGCCAGCAG C TT  CCCCTTCT  CTGCCCTGCTCCAGGCACCAGGCTCTTTCCCCTTCAGTG
                                                                 (INR+)                                         
Mouse     TGGCCCCTACCGTCTGAGGGCAAGGCGGAAGTGAGGCAGGCAGCCTGTGG C TT  CCTCTTTT  TTGTTCTGCTGTGGGCCCAAGACTCCTTACTCAGAGCCT
                                                                 (INR+)                                         
    433 Human: LAPTM5 (7805, NM_006762) 
Mouse: Laptm5 (16792, BY204278) 
 
           |-50      |-40      |-30      |-20      |-10      |1        |11       |21       |31       |41       
Human     GAAGGGCATGATGACCGTGTTAGGGTCTGTCCTCTCTGGAACCCCCTCCT G   GGAATGGC  AGAGGGCA  GTGGGGTGCTCTG  AGGCAGGCTGCTGCCCTGTC
                                                               (INR-)               (MTE-)                        
Mouse     AGAGTGCATATAAATG  CGGAGCCC  AGCCCTGGTGAAGCATTGGAGCCCCT G   GGAGTGGT  AGATGCCT  CAGTCGGGCTCCC  TGTGACCCCCCCCCCCCTTC
                            (BRE+)                               (INR-)               (MTE-)                        
    434 Human: BSND (7809, AK129999) 
Mouse: Bsnd (140475, NM_080458) 
 
           |-50      |-40      |-30      |-20      |-10      |1        |11       |21       |31       |41       
Human     TGTGCTCCCTCGCTCAGATTCACACACACACTCCAGGCCAGCCGGGCCCA G AGGGAAGGTGAGAGGGCAAGGAGTAAAGGTGGCTGGGTGTGGGTCCGTT
                                                                                                              
Mouse     CCCACCTCTGTCCCATGTTCAGAGCTACAATCACCAAACAGCTGGGCCCA C AGGGGAGGAGAGTGGCAGGAAGAAAGTT  AGATGG  CTGGGACCTTGGGCC
                                                                                          (DPE+)                
    435 Human: UNR (7812, BX648780) 
Mouse: D3Jfr1 (229663, BC029688) 
 
           |-50      |-40      |-30      |-20      |-10      |1        |11       |21       |31       |41       
Human     CTCGTGCACGCGCC  CGGAGCCC  GCAACTCTCGCGAGAGAAGCGAGAT  TTA T TCCT  ACGTACCGGG  CCGTGCTGCTTAT  GGCGGCGCTGGAGAGGGGGCGC
                          (BRE+)                             (INR+)                 (MTE-)                          
Mouse     CCCGTGCACGCGCT  CGGAGCCT  GCAACTCTCGCGAGAGAAGCGAGAT  TTA T TCCT  ACGTACCGGG  CCGTGCTGCTTAT  GGCGGCGCTGGAGAGGGGGCGC
                          (BRE+)                             (INR+)                 (MTE-)                          
    436 Human: DAP3 (7818, NM_004632) 
Mouse: Dap3 (65111, BC019566) 
 
           |-50      |-40      |-30      |-20      |-10      |1        |11       |21       |31       |41       
Human     GGTGGTTGGAG  GCCGCGGC  GGCTGCGCGTTGAGTCGTTTCCTGCCGGATG A CCCGACCCTTTTTTGCAGTCTCA  GGACGG  GCGCTTTGGAGCCGGCCCCA
                       (BRE-)                                                          (DPE+)                     
Mouse     CTGTGGTTGCA  GCCGCGGC  GACTGCGCGTTGGGTAGCTTCCTG  CCACACG A   CCCGAGCTTGCTTCACAACTGCA  GGACGG  GTGCCTGGGATCCAGCCGCT
                       (BRE-)                            (INR+)                          (DPE+)                     
    437 Human: BTG2 (7832, CR604962) 
Mouse: Btg2 (12227, M64292) 
 
           |-50      |-40      |-30      |-20      |-10      |1        |11       |21       |31       |41       
Human     ACCAATGAG  CGCCGCCG  CCGGCTGCCCCCCTACCTCCCTGGACCTCCTGA   A AAACGCT  GCCCGGGG  AAAGTCCGGGCAG  AGCCCGAGCAGCGGCCAGGGT
                     (BRE+)                                     (INR-)               (MTE+)                         
Mouse     GTCGGCCAAT  GGGCGCCA  CCGACGGCTCCCCGCCTCCCCGAGT  GGTATGA A   AGGCGCAGCCCGGGG  AAAGTCCGGGCAG  AGCCCGAGAGGTGGCCAGACC
                      (BRE+)                             (INR-)                      (MTE+)                         
    438 Human: CDC7 (8317, AY585721) 
Mouse: Cdc7 (12545, AB018575) 
 
           |-50      |-40      |-30      |-20      |-10      |1        |11       |21       |31       |41       
Human     GGCGGCGGGAAGTGTTGCGCAGGCGCATCCGATCGACTCGGTAGGTGGGG A   TCTCTTGG  AGACGGCGACCCAGG  CATCTG  GGGAGCCACAGAAGTCGTAC
                                                               (INR+)                  (DPE-)                     
Mouse     CAGCGCGGGAAGAATCGCGCATGCGCGCCTGGCGGCCCGGCTCAGTGGG  A C GCTCCT  GAGGGAAG  CGACCGCGGACGT  CC  TAACTT  CTGTGAACCGTGAG
                                                             (INR+)               (MTE+)       (DPE-)               
    439 Human: CDC7 (8317, NM_003503) 
Mouse: Cdc7 (12545, AB018575) 
 
           |-50      |-40      |-30      |-20      |-10      |1        |11       |21       |31       |41       
Human     CCCCTTCGGATCCCTCCGACCTGCGGCGGGAAAGTCGCGCGCCTGCGCAC T AAGCATCTGGTTCGGTCTCTGGCCCGAGGGAAGCCGGTCCTTCCCGGCT
                                                                                                              
Mouse     TCGTGTCGCCCTGCGGGCATCCCGCGGCGGGAAGAGGTGCGCCTGC  GCAC T GAG  CTTCCCGTTGGCACTGGCGG  ACAGTCCGCCCTA  CGCACTCGCCCAG
                                                          (INR-)                           (MTE+)                 
    440 Human: PIP5K1A (8394, NM_003557) 
Mouse: Pip5k1b (18720, AK098097) 
 
           |-50      |-40      |-30      |-20      |-10      |1        |11       |21       |31       |41       
Human     CAATGAGTGGGCTGATGCCCCGAGAAGGTCGGGCGCATGCGCAGTGCTC  G G ATTTTT  TGCTTGG  CTACCCGGAGTGA  AGCGGCCGGGTTGGGCGATTAAC
                                                             (INR+)              (MTE+)                           
Mouse     CCGGCTAGATAGAC  GAACGCCG  AGAAGCTGTGGCGCATGCGCAATACCGG G GT  TTCCTTGG  CCGCCCACCC  TGAGCCGAGCGGT  GGAGTTGGGGATAAAC
                          (BRE+)                                   (INR+)                 (MTE+)                    
    441 Human: TAGLN2 (8407, NM_003564) 
Mouse: Tagln2 (21346, AF465519) 
 
           |-50      |-40      |-30      |-20      |-10      |1        |11       |21       |31       |41       
Human     GTCCGCCCCGTCTGAGGCGGGACCCTCCTGAAAATCCCCGGCTGGGCAGG G CGCACCACTTGCAGCT  GCAGCCCTTGCCT  TG  AGTCAG  TGCGCCGCTCTC
                                                                                  (MTE+)       (DPE+)             
Mouse     GTCCGCCCCGTCTGAGGCGGGACCCCCCAGAAAACTCCAAGCCCGGCCGG G TCTTGAGCTCCACTCGCCGCT  GCAGCCCCTGTCG  TGCGTGCGCTCTCAT
                                                                                       (MTE+)                   
    442 Human: BCAR3 (8412, AF124250) 
Mouse: Bcar3 (29815, BC023930) 
 
           |-50      |-40      |-30      |-20      |-10      |1        |11       |21       |31       |41       
Human     GCGGAGCCGCAAATCACCAGTTGAGGGCCGGAGTGCGCGCCGCCGGCTCA   G AGCTGCG  CCTGCTGCTGGCCGGGCGGG  GGACGG  GGCCGGGACCGGAGCC
                                                              (INR-)                       (DPE+)                 
Mouse     GCGAAGCCGCAAATCACCAGTTGAGGGCCAGAGAGCGCGCTGCCGGCTCA   G AGCTGAG  CCTGCAGCCGCCCGGCCAGGCAGCAGCCGGAGCGGGACAGCC
                                                              (INR-)                                            
    443 Human: ANXA9 (8416, NM_003568) 
Mouse: Anxa9 (71790, AA733710) 
 
           |-50      |-40      |-30      |-20      |-10      |1        |11       |21       |31       |41       
Human     GGCGGGGGCTCCACAGGGCTGTTCACCTGCTGCTCTGTGCAGAGACAGCC   T CAAGTCC  AGCTGCTGGGGTTGCATCACCTGCAGCTAAAACAGCCACAGG
                                                              (INR+)                                            
Mouse     GTCCCTGTGGCTGAGAGGGACTGTGCACCTGCTGCTCTGTGCTG  ACAGCC T C  AGGCTCTGCTGTTGGGATTGTATCACTTGCAACCCAAGCAGTCAGGGG
                                                        (INR+)                                                  
    444 Human: ANXA9 (8416, NM_003568) 
Mouse: Anxa9 (71790, BC062140) 
 
           |-50      |-40      |-30      |-20      |-10      |1        |11       |21       |31       |41       
Human     GACTTTGAGACTAGGTCTCTGTGGTCCCACCTGGACCCACAGGGAAG  GCA G TGCA  TAAAAGCCTCCTGT  GTTTGAGGCTGAG  CCGCTGAGAGGCTGAGTG
                                                           (INR-)                     (MTE-)                      
Mouse     CTCAAGAGACAAGGTGTCTGTGGTCCCAACCTGGCCCCACAGGGAAGGCA G CGCACAAAGCCTTCTGTGTCCT  GGGCTGGGCTGAG  AGGTTGAGAGTTCA
                                                                                        (MTE-)                  
    445 Human: NR0B2 (8431, NM_021969) 
Mouse: Nr0b2 (23957, AK004930) 
 
           |-50      |-40      |-30      |-20      |-10      |1        |11       |21       |31       |41       
Human     TCCACTCACTGGGAA  TATAAAT  AGCACCCACAGCGCAGAACACAGAGCCA G A  GAGCTGGA  AGTGAGAGC  AGATCCCTAACCA  TGAGCACCAGCCAACCAG
                          (TATA+)                                 (INR-)                (MTE+)                      
Mouse     ATCAAGGATATAAATAGCACTCACAGTAGAGAGAGAGAGAGAGAGGG  CCA G ATAG  CTGGGAAGAAACAGGAACA  AGATAC  TAACCATGAGCTCCGGCCAG
                                                           (INR+)                      (DPE+)                     
    446 Human: GNPAT (8443, NM_014236) 
Mouse: Gnpat (14712, AK010896) 
 
           |-50      |-40      |-30      |-20      |-10      |1        |11       |21       |31       |41       
Human     CCGCTTCCGGGTT  CCGCGACT  GCGCGCATGCGCTGCAGGGCCCTGCGCGG C TTCCGTCCTGGCTGAGA  TGGCGGCGCCCGG    GATCCT  GTGTAGCGGCTGC
                         (BRE+)                                                      (MTE-)     (DPE-)              
Mouse     ACTTCCGCTTCC  GGCGCGGC  TTCTGCGCGTGCGTCACGGTCCCTGCGCGG C CTCGGGCCTGGTTA  AGATGGCGGTGCC  CGG  GGTCCC  GTGCAGTGGCCGT
                        (BRE-)                                                    (MTE-)        (DPE-)              
    447 Human: TTF2 (8458, NM_003594) 
Mouse: Ttf2 (74044, AK087352) 
 
           |-50      |-40      |-30      |-20      |-10      |1        |11       |21       |31       |41       
Human     TCCTGAACAAAGAGGGCGGAG  TAAAAGG  CGGAAGCAGAATTGGGGGCGGG G C  TTTGTGGA  ACTTGGGGGAC  CCAGCGAAATGGA  AGAAGTTAGGTGTCCA
                                (TATA+)                           (INR-)                  (MTE+)                    
Mouse     GTGGTGGGCGGGAC  GGGGCGGA    GTCAAAG  ACGGAAACCCGGCGGGCCAGG G CTTCCTGGCTGCGGTAT  GGACCTGGTTAAG  TGTCCGGAGCACGGTAAGG
                          (BRE-)   (TATA+)                                             (MTE-)                       
    448 Human: RGS5 (8490, NM_003617) 
Mouse: Rgs5 (19737, NM_009063) 
 
           |-50      |-40      |-30      |-20      |-10      |1        |11       |21       |31       |41       
Human     AGAGGGCTGGGAAAGA  GCTTCTA  TATATACCTCAGGAGGAAAGGCATCCC A G  ACAGTTTT  GAAGTTTTCA  AAGACTGGCTCTG  CTGTTAAGAAGTTGTAC
                           (TATA-)                                (INR+)                 (MTE-)                     
Mouse     CGGAGCTGTGAGAGAGCTTG  TATATAT  TCCTCAAAGGGAAAGGCTGCC  CC A GATTA  TTGAAGTTTCCACAG  ACGGTCAGCTGTT  GAGAGGTTCGTGCTCA
                               (TATA+)                        (INR+)                      (MTE-)                    
    449 Human: RGS5 (8490, AF159570) 
Mouse: Rgs5 (19737, NM_009063) 
 
           |-50      |-40      |-30      |-20      |-10      |1        |11       |21       |31       |41       
Human     GACCTGTGCACAGTTTAGGGCCTAACCCTGCCCTGGCCGGTTAGCAAG  AG A GTGTT  GTGATAAGAGCAGATG  AAAGCCTTATGCC  ACAGTAGTGCCTGTA
                                                            (INR-)                       (MTE+)                   
Mouse     ATCTTATCTCAGTTTAGGCTCCAACACAGCCCTAACTAAACAAACAAG  AG A CTGTG  GGGACAGAAGCCACCAGAAACATGCCCCAGCAGTGCCTGAAGTC
                                                            (INR-)                                              
    450 Human: PIK3R3 (8503, BC021622) 
Mouse: Pik3r3 (18710, BY719658) 
 
           |-50      |-40      |-30      |-20      |-10      |1        |11       |21       |31       |41       
Human     TGTCCCCT  CCGCGCC  G  TTAAAA  TGAAACTCTAGTGGCTGGAGTCCGGGCA G AGCTTGAGGGCAGTTGGTG  CGGTCGGGTTGGT  TCTTACACCCCGGCGGG
                    (BRE+)  (TATA+)                                                      (MTE-)                     
Mouse     GGTCCCCTCAGCGCA  GTTAAAA  TGAAACTCTAGTGGTTGGAGCCAGGGCA G AGCGTGAGGGGAGCCGGCGC  TGTCGTGGTTACC  TTTACTACTCGGGCAC
                          (TATA+)                                                       (MTE-)                    
    451 Human: KCNAB2 (8514, NM_003636) 
Mouse: Kcnab2 (16498, L48983) 
 
           |-50      |-40      |-30      |-20      |-10      |1        |11       |21       |31       |41       
Human     ATCAGAAGCTGGAAGA  GGCGTGGC  CCCGGGCGTCCCACGAGGTG  AAGATG C T  GCTGGCGGAACCTGCAGGAG  CTAGCGGGATTCG  GGGGCGCTAAAGTCG
                            (BRE-)                        (INR-)                           (MTE+)                   
Mouse     ATAATAGGCTGCTAGG  GGCGTGGC  CGAGGGAGTCCCACGAGGTA  AAGATG C T  GCGGGCGGAACCTGCCGGAG  CGAGCGGGATTCG  GGGGTGCTAAAGTCG
                            (BRE-)                        (INR-)                           (MTE+)                   
    452 Human: ITGA10 (8515, AY358325) 
Mouse: Itga10 (213119, AK037222) 
 
           |-50      |-40      |-30      |-20      |-10      |1        |11       |21       |31       |41       
Human     CTTCCAACTTTATTTTTAGCTGCCATTGGGAGGGGGCAGGATGGGAGGG  A A AGTGAA  GAAAACAGAAAA  GGAGAGGGACAGA  GGCCAGAGGACTTCTCAT
                                                             (INR-)                   (MTE+)                      
Mouse     CATCCAACTTTATTTTTAGCTGCCAGTGGGAGGGGGCAGGATAGGAGGG  A A AGTAAC  GAAAACAGCCAA  GGAGAGGGACAGA  GCGACTCAGAGCCTCTCG
                                                             (INR-)                   (MTE+)                      
    453 Human: LMO4 (8543, AY321514) 
Mouse: Lmo4 (16911, AF102817) 
 
           |-50      |-40      |-30      |-20      |-10      |1        |11       |21       |31       |41       
Human     CTGGAGGCG  GGCGGCGG  AGGAGGGGAGGAGGGGGCAGTGGGCGGAGGCGG G GGCTGGGAGGAGGTGC  CGCGAGGGGTGGA  GCGCGCAGCGGAGCCTGCTC
                     (BRE-)                                                         (MTE-)                        
Mouse     GCGGGCGGCAGCGGGA  GGAGCGGG  CGGAGGCGGGCGCTGGGAGGAGGGGG A GGAGGAGGAGGAGGCGC  CGCGAGGGGTGGA  GCGCGCCACAGAGCCTAGC
                            (BRE-)                                                   (MTE-)                       
    454 Human: LMO4 (8543, AY321514) 
Mouse: Lmo4 (16911, NM_010723) 
 
           |-50      |-40      |-30      |-20      |-10      |1        |11       |21       |31       |41       
Human     TGCCGCGA  GGGGTGGA  GCGCGCAGCGGAGCCTGCTCTCGGAGTTTTG  ACA G TACC  GGAGCTGAAATTG  TCAGCGGCGGCAA  GCGCAGGAAAGTTGAGAGG
                    (BRE-)                                   (INR+)                    (MTE+)                       
Mouse     GCCGCGAGGGGTGGA  GCGCGCCA  CAGAGCCTAGCTCTCGGAGTTTTG  ACA G TCCC  CGAGCTGAAATTGTCAGC  AGCAAGCGCTTTG  GAAAGTTGAGAGCA
                           (BRE+)                            (INR+)                         (MTE-)                  
    455 Human: BLZF1 (8548, NM_003666) 
Mouse: Blzf1 (66352, AK006544) 
 
           |-50      |-40      |-30      |-20      |-10      |1        |11       |21       |31       |41       
Human     CTGGGAGTAATCTCAGCTGCGTTCATCCAATCGACAGACCCGCCCCTCCA C GCTGTCGCCTGGGGCTACGAGACGCCATTATTCTGTTTCCGGCAGTTTT
                                                                                                              
Mouse     TGGCTGGGAGTAATTAGG  CCCCGCCC  ATTCACTCAAAGATCCA  CCCTTTG C   CTCATCGCCTGGG  GCAACGCGACGCC  ACCTTCTCCGTTCCAACGCGTTC
                              (BRE+)                     (INR+)                    (MTE+)                           
    456 Human: KMO (8564, NM_003679) 
Mouse: Kmo (98256, NM_133809) 
 
           |-50      |-40      |-30      |-20      |-10      |1        |11       |21       |31       |41       
Human     TGCCGCTGGCTCATGAATGCACTAGGCTTGGGGCAGTATAAAAACTCAGA   G AAATCAG  TGTGTAGGAGACACAGAAATCAGTGTCACTCAGTGACAGAAG
                                                              (INR-)                                            
Mouse     TGCTTCTGGCTCACAAAGGCTCTGGACTTCTGGCAATAAAAAGATCGCA  A A ACATCA  GCATGTAGGACAGAG  AGTTCC  GACCTGAACAGAGGTGTTCTGG
                                                             (INR+)                  (DPE+)                       
    457 Human: YARS (8565, AK125213) 
Mouse: Yars (107271, BC013552) 
 
           |-50      |-40      |-30      |-20      |-10      |1        |11       |21       |31       |41       
Human     CCGGCCGCTGCCGGAGGGGTCCAGGCCGAGTAAGCGGAGCGCCGAGCCCA G CTGATGCAACCTGGCTGGACTCGCGTGAC  AGTTCC  CGGCACGCGGCGGC
                                                                                           (DPE+)               
Mouse     GAGGAGGGGACAGACAGAATCTAGGCCTAGCAAGCGGAGCGACGAGC  TCA G CTGA  TGCAACCCGACGGGACTAGCGTGAC  AGTTCC  CGGCACGCGGAGAC
                                                           (INR+)                            (DPE+)               
    458 Human: MKNK1 (8569, NM_003684) 
Mouse: Mknk1 (17346, AK010697) 
 
           |-50      |-40      |-30      |-20      |-10      |1        |11       |21       |31       |41       
Human     AGCTCCGCCCCCTCACCCTCCTCTCTGATCGGGCCCACCCCCTG  GATCTA G C  ACCGCCTCTTCCGCGTTCTCGGAGGAGC  GATCTG  CAGGTAGGGGTGCG
                                                        (INR-)                               (DPE-)               
Mouse     GACTCCTCCCCC  TCACGCCT  CCTTCTGCTCGGGCCCTCCCCCTG  AGTTTA G C  TCCGCCTCTTCCGCGTTCTCGACGG  AGACCT  GCAGGTGGGGGTGCTCG
                        (BRE+)                            (INR-)                            (DPE+)                  
    459 Human: AKR7A2 (8574, NM_003689) 
Mouse: Akr7a5 (110198, NM_025337) 
 
           |-50      |-40      |-30      |-20      |-10      |1        |11       |21       |31       |41       
Human     CCCCAACTGCCT  CCGCGCTC  CTCCCCGGCATGGGCGGGGCCCCCGGCTCC C AACGCGCAGGCGCCGCTGCTATGCTGAGTGCCGCGTCTCGCGTAGTCTC
                        (BRE+)                                                                                  
Mouse     CTGACCTCCGCGAAGTC  CCGCCCCC  CGCCGTGGGCGGAGCCTGAAGCTT  C G AGTGCG  CAGGCGCAGTCTCCATGCTGCGTGCAGCGTCCAGAGCCGTGGG
                             (BRE+)                            (INR-)                                             
    460 Human: PPAP2B (8613, NM_003713) 
Mouse: Ppap2b (67916, AK011276) 
 
           |-50      |-40      |-30      |-20      |-10      |1        |11       |21       |31       |41       
Human     AGTTGGGGCT  GGCGCTCC  GGAGTTGCTGGGCTCAGCGCAGCTC  CCATTCA T   TAAGGAACCAGCTGCGGAGGAAGGTGGCCGAGCGCCCGCGCTGCCCACT
                      (BRE-)                             (INR+)                                                   
Mouse     GCTAGCTGCA  GGACGCTG  CGGAGTTGCAGGGCTCCGCGCTGCTCCATTCA T   TAAGTAGC  TGCGGAGGATC  GTGGCGGGACGCC  GACGCTGCCCACTCGCA
                      (BRE+)                                     (INR-)                  (MTE+)                     
    461 Human: RTCD1 (8634, NM_003729) 
Mouse: Rtcd1 (66368, AK009245) 
 
           |-50      |-40      |-30      |-20      |-10      |1        |11       |21       |31       |41       
Human     CTCGCCCCGACCGCAGTGTTCCCAGCGCGGCTCCTTTAAGCCGAGCTACG C GCATGCGCACTGACTGCGGAACTGGGCGAACCCGGGGGTTCGTTTCTGC
                                                                                                              
Mouse     GTACACAGTCGCA  CAACGCCC  CGCGCGGCCTCTCTGAGCGCTGGGTGGAG C GCATGCGTACAGCTGCCGGCAGGGTCCCCTGGGGAATTCGTTTCCACAG
                         (BRE+)                                                                                 
    462 Human: EIF3S2 (8668, NM_003757) 
Mouse: Eif3s2 (54709, AI182723) 
 
           |-50      |-40      |-30      |-20      |-10      |1        |11       |21       |31       |41       
Human     TGGCAACGCCGCTGTCTTCTACTTCCGGGAACGAAGGGGCGGAGACCC  AT A ATCCG  GAAGTGACCTCGAAACCTTTTCC  GGTCTT  ACTCACGTTGCGGCC
                                                            (INR+)                          (DPE-)                
Mouse     TGAAAACG  CCAGGCCC  TTCTACTTCCGGGAGCGAGAGGGCGGAGCCC  CCG C ATCC  GGAAGTCGTCCCTAGTCTTTTTCC  GGTCCC  ACTCACATTGCCTCT
                    (BRE+)                                   (INR+)                           (DPE-)                
    463 Human: VAMP4 (8674, AL035296) 
Mouse: Vamp4 (53330, AK018344) 
 
           |-50      |-40      |-30      |-20      |-10      |1        |11       |21       |31       |41       
Human     CTGCGGCGCTGCGGC  CGACGCCG  GGTCCGCACCAACTGTCTCCCCCTCCC A G  CTTCTTAC  CTCGGCTTCCTACTCCTTCCCCCGCCCGCCCAGCACCGCC
                           (BRE+)                                 (INR+)                                          
Mouse     GCAGCAGAGC  GGAGCGGT  GGCGGCTCTGCGCCTTCGGCCTTCCCCCTCCC   A CCCTTCT  TCCTCC  GCTTCCCGCTCCC  TCCCCCGCCTGCCCTGCGCTGCC
                      (BRE-)                                    (INR+)             (MTE-)                           
    464 Human: VAMP4 (8674, BC007019) 
Mouse: Vamp4 (53330, NM_016796) 
 
           |-50      |-40      |-30      |-20      |-10      |1        |11       |21       |31       |41       
Human     CCGTGCCCTGGAC  CCGCCCCC  TCCGCCTGGCGCCAGGCCGCCGAGCGCCG A TCGGCTCGATGAG  CGGAGGCGCTGCT  GCGGCGCTGCGGCCGACGCCGGG
                         (BRE+)                                                  (MTE-)                           
Mouse     NNNNNNNNNNNNNNNNNNNNNNNNNNNNCGCGCCAGGCGGCGGAGCGC  CG A GTCGC  TCGATGAG  CGGGGGCGCTGCA  GCAGAGCGGAGCGGTGGCGGCTC
                                                            (INR-)               (MTE-)                           
    465 Human: PEA15 (8682, AF153272) 
Mouse: Pea15 (18611, AK077421) 
 
           |-50      |-40      |-30      |-20      |-10      |1        |11       |21       |31       |41       
Human     GGCTCCGCGGGCGGAAGA  GGCGGCGG  CGGCGGCAGAAGCGGCGGCGGCGG C GGCGGGAGCCGAGGAGGAGGT  TCCGGACGCTGCT  TAGGAACCGGGGACT
                              (BRE-)                                                     (MTE-)                   
Mouse     CTCTGCTCCACGGGCGGAAGAGGCGGCGGCGGCAGGATCGGCGGCAGCGG T GGCAGGAGCCAAGGAGCAGGCTCC  AGACGC  TGCTTAGGAACCGGGGACC
                                                                                      (DPE+)                    
    466 Human: B4GALT3 (8703, NM_003779) 
Mouse: B4galt3 (57370, BC013619) 
 
           |-50      |-40      |-30      |-20      |-10      |1        |11       |21       |31       |41       
Human     TGGGACCGGGTGCCTAGTCAGCCCGGGTGGCTCCGCCCCGAGGCCCCGCC C CATGACGCGAGACCCCGCCCCC  GCAGCGCCCGCTT  CCAAGATGGCGGCA
                                                                                        (MTE+)                  
Mouse     CAAGCTTGGAGGTGAGGGGCCTCTGCGCCTGAATCTCCCAAGGCTCCGCC C CGTGACGTCAGGCCCCGCC  CCAGCGCCCGCTT  CCAAGATGGCTGCAGCG
                                                                                     (MTE+)                     
    467 Human: B4GALT2 (8704, NM_003780) 
Mouse: B4galt2 (53418, AF142670) 
 
           |-50      |-40      |-30      |-20      |-10      |1        |11       |21       |31       |41       
Human     GTTTCCCTCCCACCCTGCTCAGGCCAGCAGCCGGATGCCCGGGC  CCACTG G G  CGGGCCAGTGGCCGCCTGCG  GGATGA  GCAGACTGCTGGGGGGGACGCT
                                                        (INR+)                       (DPE+)                       
Mouse     ATCTCTCTCTCCCTCTGTTCAGGCTGTCAGCCGGATGCTTGGGC  CCATTG G G  CGGGCCATTGGCCACTTGCG  GGATGA  GCAGACTGCTGGGGGGGACGCT
                                                        (INR+)                       (DPE+)                       
    468 Human: B3GALT2 (8707, BC022507) 
Mouse: B3galt2 (26878, AK036141) 
 
           |-50      |-40      |-30      |-20      |-10      |1        |11       |21       |31       |41       
Human     CAGTGACGTTAAATTCTGCTCTGTCAGAGAGAGCATCTGTCAAGCCC  ACA T TTAA  ACTGCTGCCTGCCTGTGCA  GCAGCTGAGGAAC  CGTGGATTTCATA
                                                           (INR+)                          (MTE+)                 
Mouse     CAGTGACGTTAAATTCTGCTCTGTCAGAGACAGCATCTGTCAAGCCC  ACA T TTAA  ACTGCTGCCTGCCTGT  GCAGCACGGAAGA  ACGGTGCATTTCACAT
                                                           (INR+)                       (MTE+)                    
    469 Human: TNFRSF25 (8718, AY254324) 
Mouse: Tnfrsf25 (85030, AF329969) 
 
           |-50      |-40      |-30      |-20      |-10      |1        |11       |21       |31       |41       
Human     GCCCCGCCTCC  CCCCGCCC  GCCAGGCGGGCCCTTCTCGACGGCGCGGGGC G GGCCCTGCGGGCGCGGGGCTG  AAGGCGGAACCAC  GACGGGCAGAGAGCA
                       (BRE+)                                                            (MTE+)                   
Mouse     ACCCTACCCTAGCCATCCCCCGAGGGGAGTTGTTCTGGATGGCGCGGGGG C GGGCGGGCAGGCAGG  CTAGCTCTAGTCT  AGGAACATAGGGGCTGAGCTG
                                                                                 (MTE+)                         
    470 Human: ADAM15 (8751, AY560593) 
Mouse: Adam15 (11490, AK048901) 
 
           |-50      |-40      |-30      |-20      |-10      |1        |11       |21       |31       |41       
Human     GGCTTGGGGCCGGGTGGGAGGGGGCGGGCCGGGGCGGGGCCTGGTGGCCG C GCGGCGCTGCTGGGTTCTCCGAGG  CGACCT  GGCCGCCGGCCGCTCCTCC
                                                                                      (DPE-)                    
Mouse     GACGGGGC  GGGGCGGA  GCCTCAGACGGGGCGGGGCGGGGCCTAGTTGCTG C GCTGCACCGAGCTGGTGT  CCGGCGGGGCCGT  GGCTGCTCCTCCACGCGT
                    (BRE-)                                                            (MTE+)                      
    471 Human: ADAM15 (8751, NM_003815) 
Mouse: Adam15 (11490, AK048901) 
 
           |-50      |-40      |-30      |-20      |-10      |1        |11       |21       |31       |41       
Human     GTTCTCCGA  GGCGACCT  GGCCGCCGGCCGCTCCTCCGCGCGCTGTTCC  GC A CTTGC  TGCCCTCGCCCGGCCC  GGAGCGCCGCTGC  CATGCGGCTGGCGCT
                     (BRE-)                                   (INR+)                       (MTE+)                   
Mouse     CGAGCTGGTGTCC  GGCGGGGC  CGTGGCTGCTCCTCCACGCGTAGCCCCGC A CCTGCTGCCCCAGT  CCAGCCCGGAGCT  CCGCGGCCATGCGGCTGGCGCT
                         (BRE-)                                                   (MTE+)                          
    472 Human: PABPC4 (8761, U75686) 
Mouse: Pabpc4 (230721, AI594882) 
 
           |-50      |-40      |-30      |-20      |-10      |1        |11       |21       |31       |41       
Human     CGCCTCTCT  CCGCCCCG  GGTCGCTGCCGCCTCCGCCGCTTTCGGGCTTCG C AGCCTGAGGAAAAAAAGAGAAAA  AGATAA  AAAAAATCTGAAAACGCTTC
                     (BRE+)                                                            (DPE+)                     
Mouse     CGCCTCTCT  CCGCCCCG  GGTCGCAGCCATCAGTGTTGCGTTTGGGCTCAG C ACGCCTGAAGCAGAAAAAAGAAAAAGCTTAAAAAAAACACAAAAGCCTC
                     (BRE+)                                                                                     
    473 Human: FPGT (8790, BC020720) 
Mouse: Fpgt (75540, AK045369) 
 
           |-50      |-40      |-30      |-20      |-10      |1        |11       |21       |31       |41       
Human     CACGGAGGGGGCGGGT  CAGAAAC  AACCGGGCGGAGGCGCACCCCAGGGCG C ATGCGTGCTGTGC  GGCGCGGTCTCAG  GGAAGGTGGGGCTATGGCAGCTG
                           (TATA+)                                               (MTE-)                           
Mouse     GAGAGCATGGAGGA  GGCGGGGC  GGGGCGGGGCGGGGCGGGGTTT  GGAGTG C G  CGTGCGTGCTAAGTGTGCTCCCGGA  AGTCGG  CCATGGCGTCTCTCCGC
                          (BRE-)                          (INR-)                            (DPE+)                  
    474 Human: PEX11B (8799, NM_003846) 
Mouse: Pex11b (18632, AK005236) 
 
           |-50      |-40      |-30      |-20      |-10      |1        |11       |21       |31       |41       
Human     GCAGTAGGCGTGACTA  GGGGCGGG  AAGTGGGGCGGGAGCAGGGCCGCGGA G CCTGGGCTGCGGCTGTCATGGACGCCTGGGTCCGCTTCAGTGCTCAGAG
                            (BRE-)                                                                              
Mouse     CCCAGGAGGCGTGACTTGGGGCTGGAAGCAGGCGGGCCGCTGCGCCGTGG G CCCGGGCTGCCGCTGTCATGGACGCCTGGGTCCGCTTCAGTGCTCAGAG
                                                                                                              
    475 Human: CD84 (8832, AF054818) 
Mouse: Cd84 (12523, NM_013489) 
 
           |-50      |-40      |-30      |-20      |-10      |1        |11       |21       |31       |41       
Human     TAAATCAGTTAACATGCACTT  CCTTTCT  TAAATGTCGTTATAAAAAGGAG   G AAGAAAA  CTCAAGTGAAAC  TGACTCTGCTAGA  ACAGTGCCGTGCTTTTC
                                (TATA-)                         (INR-)                   (MTE-)                     
Mouse     AGAAGTAACACATCTTAATCAGCTCACACTTCCTCCCTCCAGCTCCGGAA A CA  CCACACTG  AAGT  GAAAGCAGCTACC  ACACCAGTTATTTTTCCTCAGA
                                                                 (INR+)           (MTE-)                          
    476 Human: FUBP1 (8880, NM_003902) 
Mouse: Fubp1 (51886, AK028418) 
 
           |-50      |-40      |-30      |-20      |-10      |1        |11       |21       |31       |41       
Human     TTTTGTTCCTTCAGTCT  CCGCCCCT  TTACGGCACGATGGTCGCGCA  AGAA T GTA  ATAGAGCTTCGACGGCCGCCATTTTCTTTCTTTCTTAGCTGTTAGC
                             (BRE+)                         (INR-)                                                
Mouse     TTTCCGTCTGCCAGTCT  CCGCCCCT  TTACGGCACGATGGTCGTGCAAG  AA T GTGAT  AGAGCCTCGACGGCCGC  CATCTT  CTTCCTTTCTTAGCAGTTAAC
                             (BRE+)                           (INR-)                    (DPE-)                      
    477 Human: EIF2B3 (8891, AK024006) 
Mouse: Eif2b3 (108067, BQ828648) 
 
           |-50      |-40      |-30      |-20      |-10      |1        |11       |21       |31       |41       
Human     CCATCGAGTTTCTCGGCCATCGCGCGCCTGCGCCATTGGGCTG  TCAGTCA G   AGGCGGCGTGGAGATCGCTG  GGAGCGGTTGCGG  CGTGCCGGGAGCTGAG
                                                       (INR+)                           (MTE+)                    
Mouse     GTATCGAACCGCTCGGCCACCGCGCGCCTGCGCTCTTGGCTTGTCAG  CCA G AGGC  GGCGTGGGGAGCGCT  GGGAGAGGCTGCG  GCGTGCGGATAGCTGAG
                                                           (INR+)                      (MTE-)                     
    478 Human: SELENBP1 (8991, NM_032183) 
Mouse: Selenbp1 (20341, AK002447) 
 
           |-50      |-40      |-30      |-20      |-10      |1        |11       |21       |31       |41       
Human     CTCTGCCCCACCCCCAGCTGGTTG  TATAAAT  TCCCTCCCTTCGCTCCTTC C CCGGAACAGCGGCCTCTGACA  CCAGCACAGCAAA  CCCGCCGGGATCAAA
                                   (TATA+)                                               (MTE+)                   
Mouse     CTCCACCTCTCCCCCAATTGGTTG  CATAAAT  TCCCTCCCTTTG  CCCCTTG C   CTGTGCAGTCCGCACT  TAATACCAGCACT    GGTCTC  TGCTGAGCCTCTGC
                                   (TATA+)               (INR+)                       (MTE+)     (DPE-)               
    479 Human: MPZL1 (9019, AF478448) 
Mouse: Mpzl1 (68481, BY061573) 
 
           |-50      |-40      |-30      |-20      |-10      |1        |11       |21       |31       |41       
Human     AGCGGACAGCGAGGGCC  GGAGTGGG  GCTGAGGCTTCGGTGCAGAGCT  GGA G AGCC  GCGGCTGGGACCGGAGTGG  GGAGCGCGGCGTG  GAGGTGCCACCCG
                             (BRE-)                          (INR-)                          (MTE+)                 
Mouse     GAGCGAGCAGT  GGGGCGGG  CACAGGGGCTGGAGCTGGGCAGAAGGCTGG  C G GTTGGC  CACTGGGGCCG  GGAGCGGGGAGCG  GTGGCGTGGAGGTGTCCTC
                       (BRE-)                                  (INR-)                  (MTE+)                       
    480 Human: SH2D2A (9047, AF106072) 
Mouse: Sh2d2a (27371, AF203343) 
 
           |-50      |-40      |-30      |-20      |-10      |1        |11       |21       |31       |41       
Human     TAAGGCTGTGGGTAAGGCGACATTTCCTGCCCCCGGGGCCAGGGTGAGAG G A  GAGATGAT  GAGTTGCTGAGTGTGCACACCTTTCCGGAACACATACACA
                                                                (INR-)                                          
Mouse     AAGGAGGTGGGTAAAGCGACATTTCCTGTCCCTGGGGGAAGGAGGGAGAG G A  GAGATGAG  CAGCTGCCGAGTGTGCACCTCCTTTGAGAGCACCCACACC
                                                                (INR-)                                          
    481 Human: ARTN (9048, AF120274) 
Mouse: Artn (11876, AK053914) 
 
           |-50      |-40      |-30      |-20      |-10      |1        |11       |21       |31       |41       
Human     TTGACACCGGACGGCTGC  GGCGGCGG  GCAGGAGGCTGCTGAGGGAT  GGAG T TGG  GCCCGGCCCCCAGACAAGG  CCCGGGGGCTCCG  CCAGCAGCAGGTCC
                              (BRE-)                        (INR-)                          (MTE-)                  
Mouse     TGGAATTTGACACCGGAC  GGCGGCGG  GCAGGAGGCTGCTGAGGGAT  GGAG T TGG  GCTCGGCCCCCAGATGCGG  CCCGCGGGCTCTG  CCAGCAACAAGTCC
                              (BRE-)                        (INR-)                          (MTE-)                  
    482 Human: ANGPTL1 (9068, NM_004673) 
Mouse: Angptl1 (72713, AK012888) 
 
           |-50      |-40      |-30      |-20      |-10      |1        |11       |21       |31       |41       
Human     TGGGCTGAACTTGAGTCTACT  GAGAAAG  AGGGAATCACTATTCAGGGGTA C TG  TATATACA  ATC  TGGGTCAGCTGCA  GCTGGTTACTGCATTTCTCCATG
                                (TATA+)                            (INR-)          (MTE-)                           
Mouse     TGTGGGCTGAACTTGAGTCTACTGAGAACGAGAGGGAATCACTCTTC  GGA C TGTG  TGGACAGTCTGTC  AGCTGCGGCTGGT  TTCTGCACATTTCCATGCA
                                                           (INR-)                    (MTE-)                       
    483 Human: TBX19 (9095, AL035297) 
Mouse: Tbx19 (83993, CA558149) 
 
           |-50      |-40      |-30      |-20      |-10      |1        |11       |21       |31       |41       
Human     GGGCGCGCCGCGGTT  GGCGCCTG  GGCGGCTGGGCGGCTGCCTAGCACCCG   G AAGAGCC  GTCAACTTAG  CGAGCGCAACAGG  CTGCCGCTGAGGAGCTGGA
                           (BRE-)                               (INR-)                 (MTE+)                       
Mouse     GGGGCGCGCCGTGGTTGGCAGCGGGCGGAGGGGCGGCGGCTGCGCACCCC G A  AGAGTGGT  AGGC  GGAGCGAACGCCA  AAGGCGGCGGTCGGGACCCTGAC
                                                                (INR-)           (MTE+)                           
    484 Human: SLC16A4 (9122, BC021664) 
Mouse: Slc16a4 (229699, AK049873) 
 
           |-50      |-40      |-30      |-20      |-10      |1        |11       |21       |31       |41       
Human     AAGTCCACTAGCAGGA  GGGCCTA  AATCGTCTGAGCCCTCCTTGGCTCTT  A C AATGCT  CACTTGTTTTCACAAT  GCAGCAAAATGAA  ATGCCTTAGAAAAA
                           (TATA-)                             (INR-)                       (MTE+)                  
Mouse     AAGTCCACAGGCAGGAGCT  GCTAAAC  CTCAGCGCTGCCCCCGCT  TGACTA C A  GTGCCTGGTTGTTTCCACCAA  GCAGCGGAATGGG  AAGCCTTAGAAAGA
                              (TATA+)                     (INR-)                            (MTE+)                  
    485 Human: SLC16A4 (9122, NM_004696) 
Mouse: Slc16a4 (229699, AK049873) 
 
           |-50      |-40      |-30      |-20      |-10      |1        |11       |21       |31       |41       
Human     CCTTCTGAGTGACTGTGGGTGGAGGGAGGGCCTGAGATGACATCAAATC  A C AGTCTT  TCCTCCTCCCCTCAGGAAGTAA  AGTCCA  CTAGCAGGAGGGCCT
                                                             (INR+)                         (DPE+)                
Mouse     GTGTACCTTGTGGGCTGGCTGGAAGGCTGGCCTGAGTTGTTCT  CAAGTTA C   AGCCTTTCCTCCTCCTCCCTCAGGAAGTAA  AGTCCA  CAGGCAGGAGCTG
                                                       (INR-)                                 (DPE+)              
    486 Human: PRPF3 (9129, NM_004698) 
Mouse: Prpf3 (70767, AK014398) 
 
           |-50      |-40      |-30      |-20      |-10      |1        |11       |21       |31       |41       
Human     CGGTGGCGGTAGCGACG  GCACGCCG  TAGGGCGGTCAGAAGGTTTCCGGTT C C  GGTGTAAC  GTTCGGGCTCCGT  CTCAGGGGCTGAA  GTTTGTGAGGTGAG
                             (BRE+)                               (INR-)                    (MTE-)                  
Mouse     GTGTGGCGGTAGCGAC  GGCGGGCC  GTAGGGCAACCGGAAGGTTTCCGGTT C C  GGTGTAAC  GTTCGGGCTCCGT  CTCAGGGGCTGAA  GTTTGTGAGGTGAG
                            (BRE-)                                (INR-)                    (MTE-)                  
    487 Human: ARHGEF2 (9181, NM_004723) 
Mouse: Arhgef2 (16800, AB093254) 
 
           |-50      |-40      |-30      |-20      |-10      |1        |11       |21       |31       |41       
Human     GCCTGGAGTTTTCTTCC  TAAATAG  AGGGGCCTGGGAAACTGAAATGC  CCA C ATCC  AGGAAGTCCTAGGAGGTCA  GGATGG  GGGCTTATCTTTCTCTCCCA
                            (TATA+)                          (INR+)                      (DPE+)                     
Mouse     AGTCTGGAGTTTTCT  CATAAGT  AGAGGGGCCTGGAAAACTTGA  GCATCTT G   ATCCAGAAGAGCCCCAG  AGGTGGGGATTAT  TTCCACTCTCCTATCTCCG
                          (TATA+)                        (INR+)                        (MTE-)                       
    488 Human: DEDD (9191, CR625264) 
Mouse: Dedd (21945, AK088329) 
 
           |-50      |-40      |-30      |-20      |-10      |1        |11       |21       |31       |41       
Human     GTGGGGAGAG  GGAGTCGA  GCGGGACGGGGCGGGGCCGGGCGGGGCCGC  AA A GGGCT  TGGGGAAGG  AAAGTGGAGGGGG  AGGCGGTGGCGACGTCCAGCCT
                      (BRE-)                                  (INR-)                (MTE+)                          
Mouse     GTGGGGAGGG  GGAGTCGA  GTGGGACGGGGCGGGGCCGGGCGGGGCCGCCA A G  GGCTTGGG  GAAGG  AAAGTGGAAGGGG  GAGGCGGTGGCGACGTCCAGCC
                      (BRE-)                                      (INR-)            (MTE+)                          
    489 Human: XPR1 (9213, NM_004736) 
Mouse: Xpr1 (19775, NM_011273) 
 
           |-50      |-40      |-30      |-20      |-10      |1        |11       |21       |31       |41       
Human     GTTACCATGGCGATGACGTCCAGAGGGCGGGGAGGGGCGGGGCTAT  GGAG A GGA  GGAGGAAGATGG  CGGGCGGGCTGCT  CTGAAGAGACCTCGGCGGCGG
                                                          (INR-)                   (MTE-)                         
Mouse     GTTACCATGGCGATGACGTCTAGAGGGCGGGGCGGGGCGGGGCTAT  GGAG A GGA  GGAGGAAGATGGCGG  GAGGGCGGCTCTG  AGGAGACCTCGGCGGCGG
                                                          (INR-)                      (MTE-)                      
    490 Human: FAIM3 (9214, BC006401) 
Mouse: 1810037B05Rik (69169, AI508502) 
 
           |-50      |-40      |-30      |-20      |-10      |1        |11       |21       |31       |41       
Human     ATCGTCAAGCTTTGTTCCTCGTGGGGGCTAGAAATCTCTTTCCAGTTCC  A G ATTGTG  AAGGGTTCCTGAGTAAGCAGCG  TGTCTC  CATCCCCCTCTCTAG
                                                             (INR-)                         (DPE-)                
Mouse     ATTTTAAGTCCTTGCCACTCAAAGAGGTCCAGCATAGCTTTCAAGCAC  CC A GTTGT  AGAAGGCTCCCAGG  TGATCGGTGTGTG  TCAGCCTCACTCCAGGG
                                                            (INR+)                     (MTE+)                     
    491 Human: FAIM3 (9214, NM_005449) 
Mouse: 1810037B05Rik (69169, AI508502) 
 
           |-50      |-40      |-30      |-20      |-10      |1        |11       |21       |31       |41       
Human     TTCCGCTGTGGTTTGTCAGCA  CTCTCTT  TTTAGCCTGAGAATAGTTAGCA A A  CAAGGGAG  GTTGTCATTTCCTCATCGTCAAGCTTTGTTCCTCGTGGGG
                                (TATA-)                           (INR-)                                          
Mouse     TCCGCTATGGTTCGCTGGTGT  TTTCTTT  TTTAGACAAAGACTAGCTA  GCA A ACAA  AACAGATTATCATTTCCTCATTTTA  AGTCCT  TGCCACTCAAAGAG
                                (TATA-)                      (INR+)                            (DPE+)               
    492 Human: DHRS3 (9249, BX648476) 
Mouse: Dhrs3 (20148, BY153191) 
 
           |-50      |-40      |-30      |-20      |-10      |1        |11       |21       |31       |41       
Human     TTTTTTTTTTTTTCTTCCCCTC  CCTAAAC  TCCTCTGTCAGTCT  GTAAACA T   TACCTGAGAATTCC  CCAGCCGAAACGG  CTGCTGGGGCAAGAAACTTCTT
                                 (TATA+)                 (INR+)                     (MTE+)                          
Mouse     TTTTTTTTCGTCCCTGGCCTTG  CCTAAAC  TCTTCTGTCGGTCT  GTAAACA T   TACCTGTGAATTTC  CCAGCCGAAACGG  CTGTTGGGGCAAGAAACTTCTT
                                 (TATA+)                 (INR+)                     (MTE+)                          
    493 Human: SEP15 (9403, AF288992) 
Mouse: MGI:1927947 (93684, AF288740) 
 
           |-50      |-40      |-30      |-20      |-10      |1        |11       |21       |31       |41       
Human     AGGCTCTGGAGT  GGACGCCC  CTAGCTTAGGGGTCCTTCTAGGCAGCCAGA A ACCTGCGGAAAATGGTAGC  GATGGCGGCTGGG  CCGAGTGGGTGTCTGGT
                        (BRE+)                                                         (MTE-)                     
Mouse     TCAGGCAGTGCAGTG  GACGTCCC  CGGCACAGGGGTCCTCAGACCCCTGA  A G ATTGAC  GGCAGACCGCAGGG  ATGGCGGCAGGGC  AGGGTGGGTGGCTGCG
                           (BRE-)                              (INR-)                     (MTE+)                    
    494 Human: SEP15 (9403, AF267982) 
Mouse: MGI:1927947 (93684, AF288740) 
 
           |-50      |-40      |-30      |-20      |-10      |1        |11       |21       |31       |41       
Human     GCCCCTGCGCTTCGCTCTTGGTTCGGTTTACGGTTGGTTTCCTCTG  CCAC A GAG  CTCAATCAAGACGACATTCAATTGGGTAAACTTGGAGAAGAAGGCG
                                                          (INR+)                                                
Mouse     AGGCCGCTTCTCGCCCTTTCGGTTCGATTTACGGTTGGGTTCCTTGTCAC G   GGACTCAA  GCAAGGC  GTTGTCCGATTGG  GTAAACTTGGAGGAGGCGGGG
                                                               (INR-)              (MTE-)                         
    495 Human: HPRP8BP (9410, NM_004814) 
Mouse: 0610009C03Rik (66585, AK002371) 
 
           |-50      |-40      |-30      |-20      |-10      |1        |11       |21       |31       |41       
Human     GAGGAAGGACTAATTTGGAACCAATTGCGTGGCGCCTGAGAGCGGCAGCG C GG  TCAGTGGC  GGCGCTGAA  GAGACCGGTTGCC  GCCATGATAGAACAGCA
                                                                 (INR-)                (MTE-)                     
Mouse     GAGGAAGAACTCAGTTGGAACCAATTGCGCGGCGCCTCCGGTCGGCGGCG C GG  TTAGTGGC  GGCGCCGAG  GAGAGTGGCTGCC  GCCATGATCGAGCAGCA
                                                                 (INR-)                (MTE-)                     
    496 Human: PARG1 (9411, BC067839) 
Mouse: B130017I01Rik (214137, AK044979) 
 
           |-50      |-40      |-30      |-20      |-10      |1        |11       |21       |31       |41       
Human     AGCCCTCGGCGGT  GGCGGCGG  CCGTAGGTGTGGGGCGGGCGTCCGCGTCC G GCACGCGAGATGGAGCGCCGTGGTGAGTGTGAGGAGGTGGCGCTTGCTC
                         (BRE-)                                                                                 
Mouse     CTAGGCGGCACGAGCCGC  CGCCGCCA  GGCTGTGGGCGTTCATCT  GAAGTC G G  CACGGGGGATACAGGAT  GGAGCCCCACGGT  GAGTGAGGGGCGGTGGTG
                              (BRE+)                      (INR-)                        (MTE+)                      
    497 Human: GGPS1 (9453, NM_004837) 
Mouse: Ggps1 (14593, AB118237) 
 
           |-50      |-40      |-30      |-20      |-10      |1        |11       |21       |31       |41       
Human     GACGCCATTTTCCCA  CCAGTTC  TATGGAAACAGAAAGTTACGCCTCAAGG C TTTCTGGGAAATAAAGTCCATACTCTGGGGCCAACGCGCAAATCCTCGT
                          (TATA-)                                                                               
Mouse     GACGCCATTTACCCA  CCAGTTC  TATGGAAACAGAAAGTTATCCCTCA  ATA C TTTC  TGGGAATTGTAGTCCAGTCTTCTAGGCCAATCGGTAAACTGACGG
                          (TATA-)                            (INR+)                                               
    498 Human: GGPS1 (9453, AF057698) 
Mouse: Ggps1 (14593, AB118237) 
 
           |-50      |-40      |-30      |-20      |-10      |1        |11       |21       |31       |41       
Human     GAGGTGAAAGG  GGCGGGGC    AACAAAG  CAGTAGGGAGGCGGCAACGACGCC T GC  GCAGTGTG  ACCGGG  ATGGCGCATTTTC  TTGCACCAACTAATGCGGTG
                       (BRE-)   (TATA+)                              (INR-)             (MTE+)                        
Mouse     AGGGGAAGGA  GGCGCGGC  CATGAGGCAGAGGGGAGGTGGCATACGACGCC T GC  GCAGTGTG  ATAGG  GATGGCGTATTTT  CTTGCACTGAGTGCTGCGGCG
                      (BRE-)                                       (INR-)            (MTE-)                         
    499 Human: RASAL2 (9462, AK075169) 
Mouse: A330066M24Rik (320357, AK039584) 
 
           |-50      |-40      |-30      |-20      |-10      |1        |11       |21       |31       |41       
Human     GTCCCTCCCCGGCTTCGTTACTCGAGTGCACACGCGGCCGGGCTCTCGGC C CTCGATTGCTTCAGCCTATCACCCTG  GGATGC  CGCCTGCCGCTGCCGCC
                                                                                        (DPE+)                  
Mouse     GTTCCTCCTCCGCTCGGTTACTGGATTGCACACGCGGCCGTGAGCTCG  GC A CTCGA  TTGCTTCGGCCTATCACCCTG  GGATGC  CGCCTGCCGCCGCCGCC
                                                            (INR+)                        (DPE+)                  
    500 Human: ADAMTS4 (9507, NM_005099) 
Mouse: Adamts4 (240913, AK076295) 
 
           |-50      |-40      |-30      |-20      |-10      |1        |11       |21       |31       |41       
Human     AATCGCAGAGGCTCA  CCATGCT  TAAAAGAGCTGGCGCGGAGAGAGGCTGG G GAGAACCCACAGGGAGACCCAC  AGACAC  ATATGCACGAGAGAGACAGAG
                          (TATA-)                                                     (DPE+)                      
Mouse     ACAGAGGGCGGTTCT  GGATGCT  TAAAAGAGCTGGAGGGGAGAGAGGCTGG G GAGAACCCGGGGAAGACCCAC  AGATAC  ACAGAAACGAGAGAGACAGAAG
                          (TATA-)                                                    (DPE+)                       
    501 Human: C1orf8 (9528, CR595744) 
Mouse: ORF18 (56374, AF116911) 
 
           |-50      |-40      |-30      |-20      |-10      |1        |11       |21       |31       |41       
Human     CGCCTCCCGCCTACGGAGAACTACATGCCCCAGCCTGCCCCGCGAAGGGA A   GAAGTCAG  GAGGCCCC  GCTTCGCGCTAAC  GCTTGCGATGGTTGAATTCC
                                                               (INR-)               (MTE-)                        
Mouse     TCGCTCCAAGCCGCCGAGAACTACTGGCCCCAGTCGGCCTGGCACGGAG  A A AGGGGC  AGGCGCGGCTC  TGAGCCCAAGGCT  G  AGATGG  TCGGCCGGGTCC
                                                             (INR-)                  (MTE+)      (DPE+)             
    502 Human: C1orf8 (9528, NM_004872) 
Mouse: ORF18 (56374, AF116911) 
 
           |-50      |-40      |-30      |-20      |-10      |1        |11       |21       |31       |41       
Human     GGCCCTCGCGCCTCTGGGCCACAGCGAACCACATTCCCCAGAAT  GCACTG C G  AAGAAATGCGGGCGG  AAGGCGCGCTGAG  GGCGGCTGTAGTTTTCCGAG
                                                        (INR+)                      (MTE-)                        
Mouse     CCAGTTCCTCGCTGGTGACCCGCAGGAACTACACTTCCCAGAAC  GCACTG C G  AGGCGTGCGGAAGCTGTGGTTGAGGGAAGCAACACCCCTCAGCTCGCT
                                                        (INR+)                                                  
    503 Human: HS2ST1 (9653, BC025384) 
Mouse: Hs2st1 (23908, AF060178) 
 
           |-50      |-40      |-30      |-20      |-10      |1        |11       |21       |31       |41       
Human     GGGGGATG  GGCGGGGG  CGGGGATGAGGGCGGCGCAGCCGCAGCGCCGGTG G AGGGGCGCGCGGCCG  CGAGCAAAGGAGG  GAGGGAAGGAAGGAAGAGAGG
                    (BRE-)                                                         (MTE+)                         
Mouse     AGGGGAGGG  GGCGGGCC  GAGGGCGGCGCAGCCGCAGCCGCATCGCCACCG G AGGAGCGCGCGGCCG  CGAGCGACCGAGG  GAGGGAAGGGAGGAAGCGAGG
                     (BRE-)                                                        (MTE+)                         
    504 Human: IPO13 (9670, AB018267) 
Mouse: Ipo13 (230673, NM_146152) 
 
           |-50      |-40      |-30      |-20      |-10      |1        |11       |21       |31       |41       
Human     GGGCAGCCCGCGCCTTGCCGAGGTCCCTGCCCCGTCCCGGCCGGCCTGGC T TGTCTTGTCAGTCACT  GGGGCGGAGGCAG  CGGCTGTAGCGGGGCTGTAG
                                                                                  (MTE+)                        
Mouse     GGACAGCCCGAGTCTTGCTGAGGTCCCCGCCCCGTCCCGGCCGGGCCGGC C   TGTCTGTC  AGTCACT  GGGGCGGAGGCAG  CGACCGTAGCGGGGCCAACGC
                                                               (INR-)              (MTE+)                         
    505 Human: JMJD2A (9682, NM_014663) 
Mouse: Jmjd2a (230674, C78996) 
 
           |-50      |-40      |-30      |-20      |-10      |1        |11       |21       |31       |41       
Human     TGTATGGCTTGCAGCCACCCTTGAATTGGTTGACTCTGTACGGCTGC  GCA G ATGC  CGACTTTAGAGG  AGGCGGAGTTTCG  GCCTTCGCCTGCTGGAAAAG
                                                           (INR+)                   (MTE-)                        
Mouse     CCTAAAGCCATTCTTGGGTTGGTTGACTATGGGCTCTGTGAGGCTGCGCA G AG  TCAGACTT  TAAAG  GAGGCGGAGCTC  T  GTCTT  TCGCCCTCAAGAAAGA
                                                                 (INR+)            (MTE+)    (DPE-)                 
    506 Human: PUM1 (9698, NM_014676) 
Mouse: Pum1 (80912, AK052145) 
 
           |-50      |-40      |-30      |-20      |-10      |1        |11       |21       |31       |41       
Human     GAGCCTATCCCAGGATGCACCGCGCCAACCCAGTCCCCAGTGGGCCGC  CA T GTTGT  CGGAGTGAAAGGTAAGGG  GGAGCGAGAGCGC  CAGAGAGAGAAGA
                                                            (INR-)                         (MTE+)                 
Mouse     GCGCCGCTCCCATGATGCACCGCGTCAGCCCAGTCCCCAGTGGGCCGC  CA T GTTGT  CGGAGTGAAAGGTAAGGG  GGAGCGAGAGCGC  CGGCGAGCGAAGA
                                                            (INR-)                         (MTE+)                 
    507 Human: KIAA0792 (9725, AK022502) 
Mouse: BC014795 (208795, BY185174) 
 
           |-50      |-40      |-30      |-20      |-10      |1        |11       |21       |31       |41       
Human     GAAAGGGGTGGCCGCAGCTCTGCGCATGTGCAGGAGGCGCCGGAGTT  TCA C TTTG  TAACTTTTAAGTGGTCGGAACACGCCCCGCGCTGCTGGGTCCCGC
                                                           (INR+)                                               
Mouse     GTAACGCGTGGACACAGTTCTGCGCATGTGCAAGAGGAGCCGGAGTT  TCA C TTTG  TAACTTTTAAGTGGTGCGGACGCCACCCCGCCTCGCCAGGTACGG
                                                           (INR+)                                               
    508 Human: KIAA0792 (9725, NM_014698) 
Mouse: BC014795 (208795, NM_144794) 
 
           |-50      |-40      |-30      |-20      |-10      |1        |11       |21       |31       |41       
Human     GCCAGGCCGGGGCAGAGGACCGCCGAGGGCCACCGCGGGAGGC  GCAGTCA G   CTGCGTACAGGCTTGCGCCGCCGCGGGCCAAGACTCCAAATTGGGAAAG
                                                       (INR+)                                                   
Mouse     GTGGACCAGCGTTAGAGAACCACATTGGGGTTCACTGCCCGGCGC  ACAGA G CT  CGAGGTCCTGCTGGGG  CCGGCGCGAGCGA  AGAGTCCAAATTAGGTAA
                                                         (INR+)                       (MTE+)                      
    509 Human: RIMS3 (9783, D87074) 
Mouse: Rims3 (242662, NM_182929) 
 
           |-50      |-40      |-30      |-20      |-10      |1        |11       |21       |31       |41       
Human     CCCGCCGGCCCAAGCTTGGGCTTTGCACCCGGCGGCGACTGAAAACGGTG G CTGCTGCGGAGCCG  GCAGCGCAGGCGG  GCGGAGCGGAGCTGCCCCCGTG
                                                                                (MTE+)                          
Mouse     CCCGCCAGCCGT  GGCTTGGG  CTTTGCACCCGGCGGCGACTGAGAACTGTG T GG  CGACTGCG  GAGCCA  GCAGCGCAGGCGG  GCGGAGCGGAGCTGTCCGGC
                        (BRE-)                                     (INR-)             (MTE+)                        
    510 Human: KIAA0494 (9813, NM_014774) 
Mouse: 4732418C07Rik (230648, BB632753) 
 
           |-50      |-40      |-30      |-20      |-10      |1        |11       |21       |31       |41       
Human     GACCAATG  GGAGTGCG  GCTTACTGGCAGGCGGCGCAGGCGGCCGCTGCC  A A AGGGGA  AGTGATCTGGGGCT  GGAGCGATGGCCC  GGGGCTGGAGCTCGCG
                    (BRE-)                                     (INR-)                     (MTE+)                    
Mouse     GCCCAATGGG  GGCGCGGC  TTACTGGCAAGCCTTGCCCGGGGCCGCTGCC  A A AGGGGA  AGTGAGCTAA  GACTGGAGCTATG  GCCCGGGTCTGGAGCCTGCG
                      (BRE-)                                   (INR-)                 (MTE-)                        
    511 Human: DNAJC6 (9829, AB007942) 
Mouse: Dnajc6 (72685, AK122293) 
 
           |-50      |-40      |-30      |-20      |-10      |1        |11       |21       |31       |41       
Human     TTACGTCTGCCTTCATCCCCATCCTTTCCGCTCCAGCATAAGCA  AAATTA T A  ACCCCGCACACCGACTTGCATGCAATTATCATAGCCCGAGTGCTCCTC
                                                        (INR-)                                                  
Mouse     TTACGTCTGCCTTCATCCTCATCCCTTCCGCTTCAGCATAAGCA  AAATTA T A  ACCCTGCACGCTGACTTGTATGCTATTATCATAGCCTGGCTGCTCCTG
                                                        (INR-)                                                  
    512 Human: CAP350 (9857, NM_014810) 
Mouse: 4933409L06Rik (74081, AK044664) 
 
           |-50      |-40      |-30      |-20      |-10      |1        |11       |21       |31       |41       
Human     CAGGGGTGGTCTCTTCTGCG  GATATAC  CTTGGCTCCTCCCAAGTCCTACG   A GTTTAAA  CTTCGACGCACC  AAACATGGCTTCT  TCGCGGTTCTGCTCCTT
                               (TATA+)                          (INR-)                   (MTE-)                     
Mouse     GGGGGTGGTCTCCTCTGCG  GATAGAC  TCTGGCTCCTCCCGAGTCCCGCCG A GCTCCCAAAGCGCCACACCAA  AGATGG  CTTCCCCGCATCCTCTAGCCTC
                              (TATA+)                                                (DPE+)                       
    513 Human: CAP350 (9857, AF287356) 
Mouse: 4933409L06Rik (74081, AK044664) 
 
           |-50      |-40      |-30      |-20      |-10      |1        |11       |21       |31       |41       
Human     GGGAGGCAGCCTTTCCGCCTTGTCTTCCTTCCCAGCGGACCGGCGGATCC C CGGAGCCGGTGCGAGGAG  GGCACCCGGTGCG  TCCCCGGAGCGGGGAGGC
                                                                                    (MTE-)                      
Mouse     GGAAGGCCGCCT  CTGCGCCT  CTCCTTCCTTGCCTGCCGCCCGGCGGATCC C GGGAGCCGGGGCGAGGCG  GGCACCCGGTGCG  TCCCCGGAGCGGGGAGGC
                        (BRE+)                                                        (MTE-)                      
    514 Human: KAB (9859, NM_014812) 
Mouse: 4933426L22Rik (71098, AK038878) 
 
           |-50      |-40      |-30      |-20      |-10      |1        |11       |21       |31       |41       
Human     GGGTCCGGCT  CCTCGCCC  TTTCCCGAGGCGCCTGCGCACTAGGCAGTCGG T CTTTGCCGTTACCGCTATGTGTGGGGCGTGTGTGGAATAACGTTATTGC
                      (BRE+)                                                                                    
Mouse     AGGACCTGGCTCGCTGCCCTTTGCGAGGCGCCTGCGCACTGAGCTGCCGT T CGCTGCCGTTCCCGCTATGTGTGGGGCGTGTGTGGAATAACGTTATTGC
                                                                                                              
    515 Human: LRIG2 (9860, NM_014813) 
Mouse: Lrig2 (269473, AK036116) 
 
           |-50      |-40      |-30      |-20      |-10      |1        |11       |21       |31       |41       
Human     AACCTCATCTTATGGCACCGATTGGCTGGCCCCTGTGCGTGAGGACGTTG C GCCGTGGGGAGGGGCGGA  CGAGAGGTGTCCG  TCAGGCCGTGTGTCCCAG
                                                                                    (MTE+)                      
Mouse     AACCTCATCCCGGGGCTCCGATTGGCTGGCTGCTGAGCGTGAGTACGTTG C GTCGCGGGGCGGGGCGGA  CGAGCGGTGTCTG  TCAGGGTTTCAGCTCTAC
                                                                                    (MTE+)                      
    516 Human: SETDB1 (9869, NM_012432) 
Mouse: Setdb1 (84505, CN526165) 
 
           |-50      |-40      |-30      |-20      |-10      |1        |11       |21       |31       |41       
Human     ACGTGTGCCCCTGGAAGGC  TCTGCCT  TAACGCATGCGTAGTTACC  TTAGT T TT  CGTCGCCTCTGAGGGGCG  CCCCGCGGCTTTG  GATTTGACCCCGTCAG
                              (TATA-)                      (INR+)                         (MTE-)                    
Mouse     GCCCCTTATCTGGAAATGCCCTACTTCACACATGCATACTCTCC  TTAAAT T C  CCATAACCCCTGTACC  GCAGCGCTATGCA  ATAAATTTGGTCCCTTCGG
                                                        (INR+)                       (MTE+)                       
    517 Human: SETDB1 (9869, D31891) 
Mouse: Setdb1 (84505, CN526165) 
 
           |-50      |-40      |-30      |-20      |-10      |1        |11       |21       |31       |41       
Human     CTTCCTCCCCTCCCCCT  CCTCCCT  TATCCCTTCGCTTTCGCTCTTTTCCG T CGAGGCCGACCCCTGAGTTGTGAGTCTGG  GGTCTG  GTTGGTGAAAAAGA
                            (TATA-)                                                          (DPE-)               
Mouse     CACGCTGTTTCCCCTC  CCCCGCCG  TCTCTCCTTCAGTTTGCCTG  GGTTTG G C  AAGGCGGAGCCCTGGAGTAAAACCTGG  GGTCGG  GGTTGTGAGACGCGG
                            (BRE+)                        (INR-)                              (DPE+)                
    518 Human: KIAA0663 (9877, NM_014827) 
Mouse: 5730454B08Rik (70579, AK041148) 
 
           |-50      |-40      |-30      |-20      |-10      |1        |11       |21       |31       |41       
Human     CCCGGAGCAGCCATCTTA  GGCATCGC  CTCATAGAGGAACTACATCTTCCA G AATCCTCGCGCCCT  GCGTCGTGCTGGC  ACTTTTACCGGCCGGGCCTTAG
                              (BRE-)                                              (MTE-)                          
Mouse     CCCAAGAAGCCATTTTAAGG  CTGGCCT  CATAGAGGAAACTACAACTCCC  A G AATGCC  TGCGCTTT  GCGTCGTGCTGGC  ACTTTCACTGGCCCAGCCTTAA
                               (TATA-)                         (INR-)               (MTE-)                          
    519 Human: KIAA0663 (9877, NM_014827) 
Mouse: 5730454B08Rik (70579, AK041148) 
 
           |-50      |-40      |-30      |-20      |-10      |1        |11       |21       |31       |41       
Human     CGGGCCTTAGAG  CGGAGCCT  GTAGCCAGGCGCCATCTTTGACGCTG  GCAG T CTT  GGTTTTCTGC  TAGTGCTGCTGCT  GCTGGGAGGACGACGGACGGCAG
                        (BRE+)                              (INR+)                 (MTE-)                           
Mouse     CCAGCCTTAAA  GGCGAGCC  TGTAGCCGGGCGCCATCTTTGACGCTG  GCAG T CCT  GGGTTTCTGC  TTGTTCGGCTGCT  GTGA  GGACGG  CGGGCGACCGCAG
                       (BRE-)                               (INR+)                 (MTE-)         (DPE+)              
    520 Human: C1orf16 (9887, NM_014837) 
Mouse: 9430023P16Rik (226517, BC082789) 
 
           |-50      |-40      |-30      |-20      |-10      |1        |11       |21       |31       |41       
Human     CCACTCCCTCCC  CCTCGCCT  CATTCACCCCCACCCCCCTGCCGAGCGAGG A GGAGCCGGAGGAGAGGAAGATGGCGGCGGCCGCCAGCACCCGCGGTGCC
                        (BRE+)                                                                                  
Mouse     CCCCCTCGCCTCACTCACTCTCCCCCCCCCCCACCCCGCGCCAAGCGAGG A   GGAGTCGG  AGGAGAGGAAGATGGCGGCGACCGCCAGCACCCGCGGGGCC
                                                               (INR-)                                           
    521 Human: LPPR4 (9890, AF541281) 
Mouse: D3Bwg0562e (229791, NM_177664) 
 
           |-50      |-40      |-30      |-20      |-10      |1        |11       |21       |31       |41       
Human     GTCGCAAGGGCGGTAGAGAGCTGAGGGAGGGTGACAGGGAGGGGAAG  CCA T TGCA  GCAACAGCTTGGA  GGAGGGAGCTGGA  CGTCGTCTCTCGCCAGAAA
                                                           (INR+)                    (MTE-)                       
Mouse     CTTGGGCGGTGGAGAGAGAGCCCAGGGAGAGTGGCAGGGAGGGGAAG  CCA T CTCA  GCAACAGCTTGG  AGAGGGAGCTGC  T  ATCCC  TTGCCCGCAAAACAC
                                                           (INR+)                   (MTE-)    (DPE-)                
    522 Human: UBAP2L (9898, AJ243668) 
Mouse: Ubap2l (74383, AK030218) 
 
           |-50      |-40      |-30      |-20      |-10      |1        |11       |21       |31       |41       
Human     GGGAAGAGGGAATCT  TATATCA  CGTGACAGGGGCGGCGCGGCCCGGGGTG T C  AGTGTGGA  GGAGACTGAGGTAA  AGTTGG  GAGCGCAGCGGCGTTGGCGG
                          (TATA+)                                 (INR-)                 (DPE+)                     
Mouse     GGGAGGAGGGAATCT  TATATCA  CGTGACAGGGGCGGCGCGGCCCGGGGTG T C  AGTGTGGA  GGATACTGAGGTAA  AGTTGG  GAGCGCAGCGGCGTTGGCGG
                          (TATA+)                                 (INR-)                 (DPE+)                     
    523 Human: SV2A (9900, NM_014849) 
Mouse: Sv2a (64051, AK080525) 
 
           |-50      |-40      |-30      |-20      |-10      |1        |11       |21       |31       |41       
Human     TCTCCCGATGCTCAGT  GGCGCGGG  GTAGGGGGGGGCGGAGGCGGGGCT  GG A GTGGT  GGAAGGGGGG  TGGCAGGTCTGCA  TTGCCGCTTCCCTGGTGCCGG
                            (BRE-)                            (INR-)                 (MTE-)                         
Mouse     TCTCCCGCTGCTCACT  GGCGCGGG  GGAGGGAGGGTCGGAGGCGGGGCC  GG A GCGCG  TGCGGGGGGCAGGTCTG  CAGTGCCGCTTCC  CCTGGTACCGGGAG
                            (BRE-)                            (INR-)                        (MTE-)                  
    524 Human: KLHL21 (9903, NM_014851) 
Mouse: Klhl21 (242785, AK198752) 
 
           |-50      |-40      |-30      |-20      |-10      |1        |11       |21       |31       |41       
Human     CTCCCCGCCCATC  CCGCCCCT  CGAGAGCCGCGGCGCCCCGCCC  TATTTAT A   GCAGCGGTGCCTAGCGCGCCG  CGTCTC  TCCGCAGGCCCCGGCCGCGGCG
                         (BRE+)                          (INR-)                        (DPE-)                       
Mouse     CGCCCCAACAGA  CCCCGCCC  ACGGAGGCTGCGGCTGGTCTGCG  TATTTAT A   GAGGCGGTGCCGGACGCGCTGGGG  CGTCCC  TGTGACCCGCAGCTCCGCG
                        (BRE+)                           (INR-)                           (DPE-)                    
    525 Human: RABGAP1L (9910, AL157958) 
Mouse: Rabgap1l (29809, AK034019) 
 
           |-50      |-40      |-30      |-20      |-10      |1        |11       |21       |31       |41       
Human     GCTCCAGCGGTGGCGGAGCGAACGGGACCGGCCCGGCTTCAGAGCGC  GAG G TGGA  GGGTGGAACG  CGGGCGCCTGAAG  G  AGTTGT  TGTCTCGGCAGCGCC
                                                           (INR-)                 (MTE+)      (DPE+)                
Mouse     GCTTCGGCCGCGGCGGAGTGAACGGGACGGGCCCGGCCGCAGA  GCTCTGG G   CGGCGGGTGGGAC  TCGGGCGTCTGAC  AG  AGTTGT  TGTTCCGGCACGGCC
                                                       (INR-)                    (MTE-)       (DPE+)                
    526 Human: LPGAT1 (9926, BC034621) 
Mouse: Lpgat1 (226856, NM_172266) 
 
           |-50      |-40      |-30      |-20      |-10      |1        |11       |21       |31       |41       
Human     TTGTGTTATCATTTGCTA  AATATAT  TGTGACATACTTCGTGCCTCGGTGA   A CAGCTCC  CTTGACC  TTGTGCGGCTCCT  CCTCCCTCGCTCTCTGTCCCCC
                             (TATA+)                            (INR+)              (MTE-)                          
Mouse     ACGGTCTAGCGTTG  GTTAACT  GTGTTCCGCCGTGGCCCGTGCCCCGCAGA A CG  GCTCTCTT  GACC  TTGTGCGGCTCCT  CCCTCCTCACTGTCCCCCTCTG
                         (TATA+)                                   (INR+)           (MTE-)                          
    527 Human: LPGAT1 (9926, D86960) 
Mouse: Lpgat1 (226856, BB865446) 
 
           |-50      |-40      |-30      |-20      |-10      |1        |11       |21       |31       |41       
Human     GGAGCGGCTCCGCCGCAGTCCCGACTCCCGCCGCTTCCTCCCGCCGCCCC T CCCCGCCAGTCCGGG  CGAACGCGGCCGG  GCCCTTGGGGACCGAGTCTCG
                                                                                 (MTE+)                         
Mouse     GCCTTCGTTAGAG  GGCGCTCA  GGGGCGCCGCCGCTTCCTCCCGCCAACCC G CCCCGCCGGCCGGGGCGCAC  GCAGCCACCGGGC  CCTGGGGAGCGAGCGA
                         (BRE-)                                                         (MTE+)                    
    528 Human: MFN2 (9927, D86987) 
Mouse: Mfn2 (170731, AF384100) 
 
           |-50      |-40      |-30      |-20      |-10      |1        |11       |21       |31       |41       
Human     TGAGACGCCGCTCGA  AGCGCCGA  GTCGCGGGGCAGCAGAGGCGTAA  GGAG T AGG  CGGGGCGAGC  CGGCTGGGCTCAG  GGTCCACCAGCTCACCCGGGTCG
                           (BRE-)                           (INR-)                 (MTE-)                           
Mouse     GCGGGGGCTCCTTTTGG  AGCGCGGA  GTTCCTAGACACTGTGGTATA  GGAG T GGT  AAGGTTGAGG  TGACAGCGTTCAG  AGGCCATCGGCTCGTCCGGGCTG
                             (BRE-)                         (INR-)                 (MTE-)                           
    529 Human: MFN2 (9927, D86987) 
Mouse: Mfn2 (170731, AF384100) 
 
           |-50      |-40      |-30      |-20      |-10      |1        |11       |21       |31       |41       
Human     ATCCACTTCCCTCCTCCCGCCTCCCCCTGGGGTGGCGCTCGCTGGTGACG T AG  TGAGTGTG  ATGGCCGCCGCGAGGCCGGGAAGGTGAAGGTGAGAGGGC
                                                                 (INR-)                                         
Mouse     CGCAGCCCCCTCCCCTCCCGCTTCTCTCGGTGTGGCGCCGGCTAGT  GACG T GTT  GGGTGTGATGGCCGCCGCGAGGCCGGGAAGGTGAAGGTGAGAGGGC
                                                          (INR-)                                                
    530 Human: RBM8A (9939, BC071577) 
Mouse: Rbm8a (60365, AK009953) 
 
           |-50      |-40      |-30      |-20      |-10      |1        |11       |21       |31       |41       
Human     GCGACCTTTCCCCTCTGCGACAGTTTCCCGAGGTACCTAGTGTCTGAGCG G CACAGACGAGATCT  CGATCGAAGGCG  A  GATGG  CGGACGTGCTAGATCTT
                                                                                (MTE+)    (DPE+)                  
Mouse     CGTGACGTTTTTCTTCTGCGACAGCTTCCCGAGGTGCTTAGCTTCTAGAG A CTCAGAAAGCGCCGAGAGAAGCA  AGATGG  CGGACGTGCTGGATCTTCAC
                                                                                     (DPE+)                     
    531 Human: THRAP3 (9967, NM_005119) 
Mouse: Thrap3 (230753, BC026580) 
 
           |-50      |-40      |-30      |-20      |-10      |1        |11       |21       |31       |41       
Human     CAGCGGGT  CTACGCCG  TCGCCGTCGTCGGAGAGCGGAGACGCTGGGCGC  G C TGTGGG  GCGGGGGCGA  GGTTCGGGCTGGT  TGTTCCGTTGCGAGCTGCAG
                    (BRE+)                                     (INR-)                 (MTE-)                        
Mouse     CGGAGCGAGGCGGTAGCC  GCCGTCGT  CGGAGAGCGGGGACGCGGGGCGCG C TAGCGGGCGGGGGCGAGCTT  CGGGCCGGTTGTG  CCGTTGCGAGATTGCA
                              (BRE-)                                                    (MTE-)                    
    532 Human: NR1I3 (9970, Z30425) 
Mouse: Nr1i3 (12355, AF009328) 
 
           |-50      |-40      |-30      |-20      |-10      |1        |11       |21       |31       |41       
Human     TGAGAGCAACTGGAGGCCA  CATAAAA  CAGACATCTCTTGTTTT  CCAGATA C   TACGGGTCATAATCC  CTAACTCCAATCA  CTGGCAACTCCTGAGATCAGA
                              (TATA+)                    (INR+)                      (MTE+)                         
Mouse     CGAGGATACCTAGAGGCCCCATGCAAGAGAAGGCCCTTGTTTTCCAG  GCA C TGAG  GACCGCAGTCCCTAATTCCTGGC  AGTTCC  TGAGATCTCAAGGAAA
                                                           (INR-)                          (DPE+)                 
    533 Human: PIGK (10026, BX648473) 
Mouse: Pigk (329777, NM_178016) 
 
           |-50      |-40      |-30      |-20      |-10      |1        |11       |21       |31       |41       
Human     TGTCGCAAAAACCCGCAGCGCTACTTTACGGCACTGCCTCCGCCCCTTCA G GTGCGGGAAGTCT  GAAGCCGGTAAAC  ATGGCCGTCACCGACAGCCTCAG
                                                                               (MTE+)                           
Mouse     CGTGCACGGCAGCGGCAGCGCTGCTTTACGGCACTCGCCCCGCCCCTTT  G C TCTTCG  GGGCGTGA  GAAGCCGGCAAAC  ATGGCGGCCCCCTGCTTCCTCA
                                                             (INR+)               (MTE+)                          
    534 Human: ARPC5 (10092, NM_005717) 
Mouse: Arpc5 (67771, AA591928) 
 
           |-50      |-40      |-30      |-20      |-10      |1        |11       |21       |31       |41       
Human     AGAACGGT  CCGAGCCG  CGGCAGTCGGCGACGCCTCAGAGCGGAAGAGG  GA A GTGAA  TCAGGCGCCGG  GTAGTGGGTTGCT  GGGCTGGGCTTGCTGAGGTA
                    (BRE+)                                    (INR-)                  (MTE-)                        
Mouse     AGAAGCTTGCAAG  CCGCGGCT  GCCGGCGACGCCCCAGAGCGGAAGAGG  GA A GTGAG  TCGGACCGGGC  TGGGCTCGCTAAA  GGAGAGGCACCGCGGAGGGG
                         (BRE+)                               (INR-)                  (MTE-)                        
    535 Human: ARPC5 (10092, NM_005717) 
Mouse: Arpc5 (67771, AA591928) 
 
           |-50      |-40      |-30      |-20      |-10      |1        |11       |21       |31       |41       
Human     GCGCGCTGTGTCCTGC  GCGCTCCT  TCCCTCGCGCGCGCTCTCCGTG  GAAG A GCA  GGGGCAGCGTGG  GAGGCGCCAAGGG  AGCGCGAACCTGAGGAGGAAG
                            (BRE+)                          (INR-)                   (MTE+)                         
Mouse     GTGCGCGGTGCGTC  CCGCGCGC  TCCCCTGCCGACGCGCGCTCCCGGGGGA C GCGGGGGCGGAGCGGGGGCTGAA  GGATGG  CTGCAAGCAGAGGAGGAGGC
                          (BRE+)                                                       (DPE+)                     
    536 Human: TSPAN2 (10100, BC021675) 
Mouse: Tspan2 (70747, AK020982) 
 
           |-50      |-40      |-30      |-20      |-10      |1        |11       |21       |31       |41       
Human     CTCCCCGCCGGAGCCTG  CCGCTCCC  CGCGCTCGTAGCGCGGGCCTGG  GGA C TGGG  GATCCCGCCGCCGGGCCGCAGCATGGGGCGCTTCCGCGGGGGCCT
                             (BRE+)                          (INR-)                                               
Mouse     GCCCCTCCAC  CGGCGCCC  GCCGCTCCCGGGGCTCGGCGCGGGCCTGGG  GA A TCGGG  AGCCCGCCGCCGGGGTCGCAGCATGGGGCGTTTTCGCGGGGGCC
                      (BRE+)                                  (INR-)                                              
    537 Human: TSPAN1 (10103, AF065388) 
Mouse: Tspan1 (66805, AK007428) 
 
           |-50      |-40      |-30      |-20      |-10      |1        |11       |21       |31       |41       
Human     AGGCGGGCAGAAGGAGGC  TTTAAAG  CGCCTACCCTGCCTGCAGGTGAGCA G T  GGTGTGTG  AGAGCCAG  GCGTCCCTCTGCC  TGCCCACTCAGTGGCAACA
                             (TATA+)                              (INR-)               (MTE-)                       
Mouse     GGCCTGCGGCCAGGAGGT  TTTAAAA  CACCTGCCCCACCTGTAGG  TGAGTA G T  GTTTTGCGAGGA  CTAGCCGCTCCTT  CAGCGTTCTGCCAGCACACTGAG
                             (TATA+)                      (INR-)                   (MTE+)                           
    538 Human: ELA3A (10136, M18693) 
Mouse: Ela3b (67868, BC061066) 
 
           |-50      |-40      |-30      |-20      |-10      |1        |11       |21       |31       |41       
Human     CCTGCTTACCTTCTGCCC  TATATTA  GAGCCCCAGGCTCTGTGC  CCTTTTC C   TATCATCACAAAACTCATGA  TGCTCCGGCTGCT  CAGTTCCCTCCTCCTT
                             (TATA+)                     (INR+)                           (MTE-)                    
Mouse     TTCCACCTGCCTCCGGCACTA  TATTAAA  GTCCCGGTTCCACATCTCTTT  C T AATCAC  CCTGAAGCTCACCATGCTC  CGACTG  CTGAGTTCCCTCCTGCTT
                                (TATA+)                        (INR+)                      (DPE-)                   
    539 Human: MAP17 (10158, NM_005764) 
Mouse: Pdzk1ip1 (67182, AK008253) 
 
           |-50      |-40      |-30      |-20      |-10      |1        |11       |21       |31       |41       
Human     TCTCCGACGCCCGTCTTCGTGTCTCCTCCCTCCCTCGCCTTCCTCCTTCC T AGCTCCTCTCCTCCAGGGCCAGACTGAGCCCAGGTTGATTTCAGGCGGA
                                                                                                              
Mouse     CACCAGTCTGCCAGTCCTTCCTCCCCGCCCTCTTTCCACTTCTGCCTTAC   C CAATCCA  ACCTCTCCTTTAGAG  TCAGACTGCTCTC  CAGCTGATCTCATA
                                                              (INR+)                      (MTE-)                  
    540 Human: INADL (10207, AJ224747) 
Mouse: Inadl (12695, BF719028) 
 
           |-50      |-40      |-30      |-20      |-10      |1        |11       |21       |31       |41       
Human     GGGCGAGGGGAGGGCCTGCCAGGTGAGGCGCGGTCACCCTGGGCCTC  TCA C TTCC  GCCCAGGTGAGGCAGGGCCGACACCGAGCCCGCCCGACCCGGGCT
                                                           (INR+)                                               
Mouse     CCAGGTGAGGAGGCGGCCGCACTTCCGTCCAGGTGAGGGCAGCAGCT  GTA C TTCC  GCCAGGTGAGGAGGCCGTCCGTGCCCGCAGCCCCGGGGCTCCCAC
                                                           (INR+)                                               
    541 Human: PRG4 (10216, NM_005807) 
Mouse: Prg4 (96875, NM_021400) 
 
           |-50      |-40      |-30      |-20      |-10      |1        |11       |21       |31       |41       
Human     GCCTAAGTTAGTGGTGAGATGAAAGAGCTGTTTTCTGATACTTTTATT  TT A TTTTC  AGCAAGGGTACCTACGGTACCTGAAAACAACGATGGCATGGAAA
                                                            (INR+)                                              
Mouse     ACGTTATTGAGTGGTGAG  ATTAAAG  AGCGGGTTTCTGATGTTT  TTATTTT A   TCTCAAGCAAGGGCGTT  GCATCCGAGAACC  ATGGGGTGGAAAATACTTC
                             (TATA+)                     (INR+)                        (MTE+)                       
    542 Human: GPA33 (10223, BC069789) 
Mouse: Gpa33 (59290, AK008784) 
 
           |-50      |-40      |-30      |-20      |-10      |1        |11       |21       |31       |41       
Human     GGTGGGTGAGAAGAGGGAAAATTGCAGGGACCTCCAGTTGGGCCAGGCCA G   AAGCTGCT  GTAGCT  TTAACCAGACAGC  TC  AGACCT  GTCTGGAGGCTGCC
                                                               (INR-)             (MTE+)       (DPE+)               
Mouse     CCCAGGTGAGCTGGAGGAGAGTCACAGGGACTACAGGCTGAACCAGG  CCA G AGGC  CATAGCTTTAACCAG  ACAGCCCAGACC  T  GTCCA  GTGCCCTGCCAG
                                                           (INR+)                      (MTE+)    (DPE-)             
    543 Human: GPA33 (10223, BC069789) 
Mouse: Gpa33 (59290, AK008784) 
 
           |-50      |-40      |-30      |-20      |-10      |1        |11       |21       |31       |41       
Human     AGTGCATTTCCCTTTT  TATTATT  TATTTCCTGTCAGTTATAAGAGAGGCC T A  CCCCTTTG  TGAGCA  GTCTAGGACTTTG  TACACTTGTTAAGTAGGGAGA
                           (TATA-)                                (INR+)             (MTE-)                         
Mouse     CGCTCCCTTTTTATTACACAGCAACAACATCCCATCAGTTATGAGAGGT  C C ACACCT  TCTGAGAAGCACAGG  ACTTTGCACTCCT  GCTGAAGAGGGAGAA
                                                             (INR+)                      (MTE-)                   
    544 Human: STX6 (10228, CR591752) 
Mouse: Stx6 (58244, NM_021433) 
 
           |-50      |-40      |-30      |-20      |-10      |1        |11       |21       |31       |41       
Human     GAGGAGCCGGGACTGGAGGCTGCCGAGGGGGCCGGCGCCCGAGTCCGGGA T TCGGCCAGTGGTGC  TGAGCGAGTGCTG  GACCAGCGGCCGTCCTGTGCAC
                                                                                (MTE+)                          
Mouse     CCGGAGGTGCCGGGGGAGGAGGCTGCCGTGGGGGCCGGCTTGGAGTGGGA T TCGACCCGCGGCGCTG  ACCGACTGCTGGA  CCGACGGCCGACGGGCACCC
                                                                                  (MTE-)                        
    545 Human: HNRPR (10236, BC001449) 
Mouse: Hnrpr (74326, BI527056) 
 
           |-50      |-40      |-30      |-20      |-10      |1        |11       |21       |31       |41       
Human     GGGATCGCGTCTCAGTCCATCAGGGGGGGGAGGGGGTGGCGCGCGCG  CCA T TTCT  AGTCGTTTTC  AAAGCGCCTCGCG  CTGATTCTCACGGGCCCGGCTG
                                                           (INR+)                 (MTE+)                          
Mouse     GGATCGCGTCTCGGTCCATGCGAGGGGGGGAGGGGGTGGCGCGCGCG  CCA T TTCT  TGTCGTTTTCAAAGC  GCCTCGCGCTGCT  TCTCACGGGCCAGGCCG
                                                           (INR+)                      (MTE-)                     
    546 Human: HNRPR (10236, NM_005826) 
Mouse: Hnrpr (74326, BI527056) 
 
           |-50      |-40      |-30      |-20      |-10      |1        |11       |21       |31       |41       
Human     CAGGCCGCCTCGCCAAGCGATTGCCCCGTAGCTCGGAGTCCCTGGAA  GCA G TTCC  GGGAAACCCCGCGTG  CTGCCGGGATCG  C  GTCTC  AGTCCATCAGGG
                                                           (INR+)                      (MTE-)    (DPE-)             
Mouse     CATACGGTCTCGTGGGCCGATTGGTCGGCCGCACGGAGTCCCTGGAA  GCA G TTCC  GGGAAACCCCGCGTG  CTGCCGGGATCG  C  GTCTC  GGTCCATGCGAG
                                                           (INR+)                      (MTE-)    (DPE-)             
    547 Human: SRRM1 (10250, NM_005839) 
Mouse: Srrm1 (51796, AK032599) 
 
           |-50      |-40      |-30      |-20      |-10      |1        |11       |21       |31       |41       
Human     CGCCGGTCCCGCCCTCC  GTCGCGGC  GGCGCGGTGTACCCTGGGATAGGGA G CGATCTCCGAGCGAGGCGGCA  AGATGG  ACGCGGGATTTTTCCGCGTAAG
                             (BRE-)                                                  (DPE+)                       
Mouse     TGCCGGTCCCGCCCTCC  GTCGCGGC  GGCGCGGTGCACGCTGGGATAGGGA G CGATCTCCGAGCGAGGCGGCA  AGATGG  ACGCGGGATTCTTCCGCGTAAG
                             (BRE-)                                                  (DPE+)                       
    548 Human: SF3B4 (10262, AK097315) 
Mouse: Sf3b4 (107701, NM_153053) 
 
           |-50      |-40      |-30      |-20      |-10      |1        |11       |21       |31       |41       
Human     CCACCGCTACGTCCGTCTTCGCCCCGGAAGTGGAAGTCGTGCTGAGGTCA G AAGGCGGAACCGCTGCTGGGAGACGGCGG  GATCTC  TTTCGCCATGGCTG
                                                                                           (DPE-)               
Mouse     GGTTAGTGGTTTCATAAACAGGGGATCTGTGCAGAAAACAGAC  AAAGCGA G   GGGGCGGTGCTGCTGCTT  AGAGACGGCGGGA  GCCCCTTTGCCATGGCTG
                                                       (INR-)                         (MTE+)                      
    549 Human: ZMPSTE24 (10269, AB016068) 
Mouse: Zmpste24 (230709, AK083566) 
 
           |-50      |-40      |-30      |-20      |-10      |1        |11       |21       |31       |41       
Human     TGTAGAAACTAGGAGGGACTAGGGAGAGTCTGCAAGGGGAAGAGTG  TGTC T GTG  ACACTGCCAGCAGCGCGCAGAGGGAG  GGATGG  GGGCGGGTATCGGC
                                                          (INR-)                             (DPE+)               
Mouse     GCAGAAATAAGTAGGTCCTTGAAGAGTAAGGCTGGAGGGAATCGTGTC  TT T GTGAC  ACTTCTGGC  AAAGCTGAGGGAA  TGGG  CGTCTG  AAGAAGCCCTCG
                                                            (INR-)                (MTE+)         (DPE-)             
    550 Human: UBE4B (10277, AB028839) 
Mouse: Ube4b (63958, AF260926) 
 
           |-50      |-40      |-30      |-20      |-10      |1        |11       |21       |31       |41       
Human     TGCTCAGGGAACAAGCGGCTGTAGTAGTCTGTGGGGCGACTGGAGTGACC G AAGCCAAGGCAGT  TTAGTGCCTCTCG  TGTTCTTATTTTTTAACCTCTGA
                                                                               (MTE+)                           
Mouse     GCAGCTCTGGGCGCTTGCTGCTGGAGGAGGCTGCGGCGGTGCGAGCGGCC G AAGCCAAGGCGAT  TCAGTGCCTCGTG  TTCTTAGTTTTTAACCTCTGACT
                                                                               (MTE+)                           
    551 Human: BCAS2 (10286, NM_005872) 
Mouse: Bcas2 (68183, AK048527) 
 
           |-50      |-40      |-30      |-20      |-10      |1        |11       |21       |31       |41       
Human     GCGCCGGCGTAATTA  AATATAA  CTGAGTTTACGCAGACGCAGAAAACGCA G   GCAAACCT  GAGGTCCTCAGA  ATGGCGGGCACAG  GTTTGGTGGCTGGAGA
                          (TATA+)                                (INR+)                   (MTE+)                    
Mouse     CGCAGGCGCAGTGG  AATTAGA  ACCGAGGCTGCGCAGACTCAGAAG  GCGCT C TT  GAAGCCTAGTGCCGCGCG  ATGGCGGGCACGG  GCTTGGTAGCCGGAGA
                         (TATA+)                           (INR+)                         (MTE+)                    
    552 Human: AKR1A1 (10327, NM_006066) 
Mouse: Akr1a4 (58810, AY207460) 
 
           |-50      |-40      |-30      |-20      |-10      |1        |11       |21       |31       |41       
Human     CGCGGTGCAATGTGGGCCAGCAAAAGGCGAGGCTGGCCCCGCCCCTTGCA C CGCCCACGTGGCCAGCGCCACCTGCCTCATTGTGCCCAGGAGTTCTCCA
                                                                                                              
Mouse     CCTGCGCGGTGCACTGTGGGCCGGTTGTGGGCGCGGCCAGCCACCCA  AGA C TGTC  CACGTAGCTTGTGCGCATGCACAGTTTCGGGACCCCGGCCCTTCC
                                                           (INR-)                                               
    553 Human: AKR1A1 (10327, NM_006066) 
Mouse: Akr1a4 (58810, AY207460) 
 
           |-50      |-40      |-30      |-20      |-10      |1        |11       |21       |31       |41       
Human     ATGCCCGGCCCCTGCCCCTCCCTCCGGGTGGAACTTCCCCCTCACCG  CCA G ACTT  AAGCTGAGG  ATCGTTGGATCTC  TGGCGGGGTGCAGAACTGAGCCC
                                                           (INR+)                (MTE-)                           
Mouse     GGAGCCGGGCCCTCGCTCCTCCCTGGGGTGGGGCTGCCGCTTCTCCGCC  G G ACTTAA  GTCGGGCCCTGTTGCCTCAGTACTGGAGTGCAGAGCTGAATTC
                                                             (INR-)                                             
    554 Human: WARS2 (10352, NM_015836) 
Mouse: Wars2 (70560, AK036902) 
 
           |-50      |-40      |-30      |-20      |-10      |1        |11       |21       |31       |41       
Human     AGCAGGAGTCGCGAGACTGGGATTGGCTCTCTGAACAGACGTCATT  CCAA A CAA  GACGGCTCCGCCCTTCTCA  AGATGG  CGCTGCACTCAATGCGGAAAG
                                                          (INR+)                      (DPE+)                      
Mouse     GGCGCATGCGTAGTAAGCACAACTCGGCTCGCCCGCAGACGTCATTCCGG G CCGGCGGCCCCGCCCTTCTCA  AGATGG  CGCTGTTCTCTGTGCGGAAGGC
                                                                                   (DPE+)                       
    555 Human: CEPT1 (10390, AK023381) 
Mouse: Cept1 (99712, AA547486) 
 
           |-50      |-40      |-30      |-20      |-10      |1        |11       |21       |31       |41       
Human     CGCTGCTCGTTCAAACCTTGTTCCCCTTTACGGCAATCGCGAAAGTGTCG T G  AACGTGCT  GCCGCCGATCAGTCACCC  AGTCGG  CTGGAGTCGGAGGCGA
                                                                (INR-)                     (DPE+)                 
Mouse     CCTCAACCTTTTCTGAGTCCCCCCTCTTTACGGCAATCGTGAAAGTGTCG T G  AACGTGCT  GCCGCCTATCAGTCACTC  AGTCGC  CTGGAGTCGGAGTTGC
                                                                (INR-)                     (DPE+)                 
    556 Human: PIAS3 (10401, AK027557) 
Mouse: Pias3 (229615, NM_018812) 
 
           |-50      |-40      |-30      |-20      |-10      |1        |11       |21       |31       |41       
Human     GGGCCGGGAAGGGGAGG  TATAGGG  GGCCGGACTGGGCGGAGCAGGCG  GCA T TTGC  GGCCGGCGCCAGGGTGGAG  AGTTGT  GCGCCGGTCCCTGGGCCTGA
                            (TATA+)                          (INR+)                      (DPE+)                     
Mouse     GGGCCGGGAAGGGGAGGTGTAGGGGGCCGGACGGGGCGGAGCAGGCGGCC T TTGCGGCCGGCGCTGGGGCGGAG  AGTTTGCGCCGGT  CCCTGGGCCTGAG
                                                                                         (MTE-)                 
    557 Human: PIAS3 (10401, NM_006099) 
Mouse: Pias3 (229615, NM_018812) 
 
           |-50      |-40      |-30      |-20      |-10      |1        |11       |21       |31       |41       
Human     TTTGCGGC  CGGCGCCA  GGGTGGAGAGTTGTGCGCCGGTCCCTGGGCCTGA G CTCCGGCTCCGGCT  GGGGCGCCTGCGA    TGTCTC  AAGATGGCGGAGCTGG
                    (BRE+)                                                        (MTE+)     (DPE-)                 
Mouse     CTTTGCGGCC  GGCGCTGG  GGCGGAGAGTTTGCGCCGGTCCCTGGGCCTGA G CTCCGGCTCGGGCT  GGGGCGCCTGCGA    TGTCTC  AAGATGGCGGAGCTGG
                      (BRE-)                                                      (MTE+)     (DPE-)                 
    558 Human: TESK2 (10420, AB057597) 
Mouse: Tesk2 (230661, AK047755) 
 
           |-50      |-40      |-30      |-20      |-10      |1        |11       |21       |31       |41       
Human     CTGCTGGGCCGGGTAGGCGTTTCAGTCTTTCGCGGCCCGGGAGCTCAGCA G AGCTACCAGCTGCCCTGT  TGGCTTCGCTGGT  CGGATCGTCCTCCTGGCC
                                                                                    (MTE-)                      
Mouse     CCAGCCCCCTGCCCCCCAGGCGTTTCAGACACTCACCGGCGGGGAGCCG  A G AGTTTC  CAGCTGCCTTCTCAGTC  CCAGCGGTGATCC  GCCTCCGGGCCCC
                                                             (INR-)                        (MTE+)                 
    559 Human: TIMM17A (10440, NM_006335) 
Mouse: Timm17a (21854, BY251099) 
 
           |-50      |-40      |-30      |-20      |-10      |1        |11       |21       |31       |41       
Human     GAAATGGTGAACCAATGCTCA  TAGACCT  TAACGCCCTCCTCTCGGGA  TCA C TTCC  GCCTCTGGGGTCAGGCT  CCGCCCAGCTTGC  CCGGCATCACTCGCG
                                (TATA-)                      (INR+)                        (MTE-)                   
Mouse     GGAAGAGATGCTCCAATCCCCGTCCAGTAGCACGCCTCCGCCTGGGA  TCA C TTCC  GTCGCGGGGATC  AATTTCCGCCCAG  CGTGCCGTAGACTCTCGCGC
                                                           (INR+)                   (MTE-)                        
    560 Human: PPIE (10450, NM_006112) 
Mouse: Ppie (56031, AK008177) 
 
           |-50      |-40      |-30      |-20      |-10      |1        |11       |21       |31       |41       
Human     AGCGTTGGG  GGCGGGGC  GGGACAGTGGGGAAGAGGACGGGTCGAGTGCTG G CTTCCGGCGGAAAAGCGCGCGAGCA  AGATGG  CCACCACCAAGCGCGTCT
                     (BRE-)                                                              (DPE+)                   
Mouse     CGCCCCGACCTAA  GGGGAGGG  GCGACGGCGAGGGGGCCGGCCGAGTGCCG G CTTCCGGCGGAAA  CGGGCGCGAGCA  A  GATGG  CCACCACCAAGCGTGTGC
                         (BRE-)                                                  (MTE+)    (DPE+)                   
    561 Human: MAD2L2 (10459, NM_006341) 
Mouse: Mad2l2 (71890, AK009587) 
 
           |-50      |-40      |-30      |-20      |-10      |1        |11       |21       |31       |41       
Human     CCGTGCGGGAATG  GGCGTGGC  CTGGGCGGGGCGGGCGCTAGGACCCACCG G AGCGCCGTGAACGTCAC  CGAGCGGCGCCGA  GGCCCCGGGTTGAGCGGGA
                         (BRE-)                                                      (MTE+)                       
Mouse     ATTGGACAGCTCCGGGGTAGG  CGTGGCT  TATGGTTGTGCTGGGACCGCGG G AGCACCGCGGTGGTTACTGAG  GCCAGGGGATTCT  GGAGTAGCGCGTTAG
                                (TATA-)                                                  (MTE-)                   
    562 Human: MAD2L2 (10459, AK094316) 
Mouse: Mad2l2 (71890, AK009587) 
 
           |-50      |-40      |-30      |-20      |-10      |1        |11       |21       |31       |41       
Human     GGGAGTGGGCGGGCAGGAGGGAAGGGGGCGGGAGCGCGGGGAGGGGACAG A GGAGGGGCGGCGG  GTGGCGGGCTCGA  CTGCCCCCAGCCGAGGGGCAGCC
                                                                               (MTE-)                           
Mouse     CGAAGGAGGGAGTGAGCGAGGGGCGGGGACGGCGCGCAGGGAGGG  GACGT A GG  AGGGGCCGCGGAGCTCCGCG  ACCTCCCGCCGAG  GGCCAGCCCCTGGC
                                                         (INR-)                           (MTE-)                  
    563 Human: PPIH (10465, NM_006347) 
Mouse: Ppih (66101, AK008394) 
 
           |-50      |-40      |-30      |-20      |-10      |1        |11       |21       |31       |41       
Human     CGTGAGTGGGGTGACCA  TTGACCT  TAACCAAAAAGATGTCAAGGCTTTCC T CCTCCTCACTCCCGCGTCC  CCAGTGCCAGGCA  GAGTCTCTCAGCTAGAC
                            (TATA-)                                                    (MTE+)                     
Mouse     GTCATGCAAGTGGGGAGCCCA  CAGACTT  TGACAAAGATGCCTGCGTTTCC T CCCCTCAGCTCCGACCAGC  TCAGCCAAACAGT  ACCATTCCCAACAACCT
                                (TATA-)                                                (MTE+)                     
    564 Human: CAP1 (10487, NM_006367) 
Mouse: Cap1 (12331, AK053351) 
 
           |-50      |-40      |-30      |-20      |-10      |1        |11       |21       |31       |41       
Human     GTGAGAGCCGGGCCGCTCTCTCCGGGGCGTGGCGAAGAGGGGC  GGAGTCA C   GAGCGGGGCGGTGAGACTTCCTGCCC  AGTCGC  GGGCCAGCCTAGCGCTT
                                                       (INR-)                             (DPE+)                  
Mouse     GCTCCTAAGCCAGATTCTCTCTCCGGTGGCGGGGAATGAGGGC  GGAGTCA C   GAGCGGAGCGGCCAAAC  TTCTTCCTCTCCC  CCGGGCTAGCGTTACAGCC
                                                       (INR-)                        (MTE-)                       
    565 Human: CAP1 (10487, CR611821) 
Mouse: Cap1 (12331, AK053351) 
 
           |-50      |-40      |-30      |-20      |-10      |1        |11       |21       |31       |41       
Human     CGGTGGGGGCG  CCGCGCCC  AGTGAGGGCCCGGAAGTGGGTCGCGCGG  AGA T TGCT  GGGCGGTTCTTGCCG  GAAGCGGAGAGCG  GCTGATCGCAGTCCGGA
                       (BRE+)                                (INR-)                      (MTE+)                     
Mouse     CGGTGGGGGTG  CCGCGCCC  AGTGAGGGCCCGGAAGTGGGTCGCGC  GAAGA C CG  CTGGGCGGTTCTTGCCG  GAAGTGGAGAGCG  GCTGATCGCAGTCCGGA
                       (BRE+)                              (INR+)                        (MTE+)                     
    566 Human: MUF1 (10489, NM_006369) 
Mouse: D630045E04Rik (230654, AK207063) 
 
           |-50      |-40      |-30      |-20      |-10      |1        |11       |21       |31       |41       
Human     GGAGCCGTCCTAACTGCT  GACCTTT  TCAAGATGGCGGTCCGCGGGCG  ACA C TCTC  GGGCTCGCGCACCTCCCCA  AGATGG  CGGCGCCCGAGGCCTGGCGC
                             (TATA-)                         (INR+)                      (DPE+)                     
Mouse     GGAGCGGTCCTAAAAGCT  GACCTCT  TCAAGATGGCGGCCCGCGGGTG  TCA T TCTC  GGGCTCGCGCACCTCCCCA  AGATGG  CGGCGCCCGAGGCCTGGCGC
                             (TATA-)                         (INR+)                      (DPE+)                     
    567 Human: MUF1 (10489, NM_006369) 
Mouse: D630045E04Rik (230654, AK207063) 
 
           |-50      |-40      |-30      |-20      |-10      |1        |11       |21       |31       |41       
Human     GTGCGGCACACTCATCGTGGTCGCGAACACATGCTCAGATTGG  CATGTAC T   ACCAGCCGCAGGCAGA  GGTTAGGGGTAGC  TGATGTTTGCACCCGAGGAG
                                                       (INR-)                       (MTE-)                        
Mouse     GTGCGGCATACTCCTCGTAGTCCCGAACACACGCACAAATTGGGCTGTA  C T ACTCGC  TAGCTACGAGACGT  AGGGGGAGCTGCT  ATATACGCCTGCAAAG
                                                             (INR+)                     (MTE-)                    
    568 Human: SEMA6C (10500, NM_030913) 
Mouse: Sema6c (20360, AF363972) 
 
           |-50      |-40      |-30      |-20      |-10      |1        |11       |21       |31       |41       
Human     GGCGGGCGGGGCCACTGGGGCCGAGCCCAGAGCCCCGGGCGGTC  GCATTG T T  TTCCTCCGCGGATCCGCGGCT  GGACTT  GGACCCAGGGCTCTCCCGACA
                                                        (INR+)                        (DPE-)                      
Mouse     GGCGGGCGGGGCGGCTGGGGCCGAGCCCAGAGCCCCGGGCGGTC  GCATTG T T  TTCCTCCGCCGATCT  GCGGCGGGAATAG  GGGCTCGTACTCGGACCCGA
                                                        (INR+)                      (MTE+)                        
    569 Human: HBXIP (10542, NM_006402) 
Mouse: Hbxip (68576, AK003358) 
 
           |-50      |-40      |-30      |-20      |-10      |1        |11       |21       |31       |41       
Human     TGCACCGTGATCAATTTTGTCCCTTTGGAGGCGCCGTTACGGTCCACGCC C CGCTCGCGTCAAGTGACTGAGGCCTGTGGTGGAGAAGGACGTGCCGTGC
                                                                                                              
Mouse     GGCGCCGCGCTGAATTTCGTCCCTCTGGAGACGCCGTCCAGGCGCCCGCC T CGGTCAAGTCACG  TGATCGAGGTTTG  CGGTGAAGGAGGAAGTGTTTTGC
                                                                               (MTE+)                           
    570 Human: SLC19A2 (10560, AF153330) 
Mouse: Slc19a2 (116914, AF224341) 
 
           |-50      |-40      |-30      |-20      |-10      |1        |11       |21       |31       |41       
Human     GCAAGTATTCCC  GGCGTCCG  CTGTGATTGGTTCCCGGAGTGGAGGCGGTG G CAGAGGGTGGGCCTTAGGACG  GGTCTC  CCTTAAACTGGGCGATCAGGCA
                        (BRE-)                                                       (DPE-)                       
Mouse     AGTGATCGTCTGCTGTGGGTGGGCGTGGCGTGGGGCGTGGCCAGGCGGAG G CGGGCCAGACCGG  GAGGCGGACCTAG  GGAAAGGGCGTTCTGCGGTCGGG
                                                                               (MTE+)                           
    571 Human: IFI44 (10561, BC022870) 
Mouse: Ifi44 (99899, AK085407) 
 
           |-50      |-40      |-30      |-20      |-10      |1        |11       |21       |31       |41       
Human     TTTCTCTGAGTCTTTGAAGCTTCAAGGCTGCTGAATAATTTCCTTCTC  CC A TTTTG  TGCCTGCCTAGCTATCC  AGACAG  AGCAGCTACCCTCAGCTCTAG
                                                            (INR+)                    (DPE+)                      
Mouse     GTTTTCGTTTCCCGAGCAGGATGAGGCAAACAGAGGAAAAGTTTCTCT  TC A TTTTC  TACAGGCTTGGTCACTG  AGACAG  AAGCTGCCCTCCTCGGTGCCA
                                                            (INR+)                    (DPE+)                      
    572 Human: POLR3C (10623, NM_006468) 
Mouse: Polr3c (74414, AK043194) 
 
           |-50      |-40      |-30      |-20      |-10      |1        |11       |21       |31       |41       
Human     AGAAAGGGCGGTGGGCTCCACCTCGGCCTAGAAGGCCAGCGGGAGCCGTA G GAAGCCGTCGCGGGAAGC  TCAGCCGAATTGG    AGTTGG  AGCCCCCGGATT
                                                                                    (MTE+)     (DPE+)             
Mouse     TGGATGGTGCTCTACT  TCTAAGA  AGGGGCGATGGGAAGCTCAT  GAGCTGA G   GCTGCTGGCCTCGGGAGGACAAC  ATTTGGAGCTTCT  AAATTAGCCCCGA
                           (TATA+)                       (INR-)                              (MTE-)                 
    573 Human: POLR3C (10623, CR605615) 
Mouse: Polr3c (74414, AK043194) 
 
           |-50      |-40      |-30      |-20      |-10      |1        |11       |21       |31       |41       
Human     AGCCGCCGAAACTCTGGGGTAGAGTGGCACGCGCTTTTTGCTTTTCCGCG T   CTTCTTCG  GTGGCGATCCGCGTCCTAGAAAGGGCGGTGGGCTCCACCTC
                                                               (INR+)                                           
Mouse     AAACCACCGACACTGTCGGCTAGAGCGGCCCGTGCTCTGACCCTTCCGCG T C  CGCCTGGA  TGGTGCTCTACTTCTAAGAAGGGGCGATGGGAAGCTCATG
                                                                (INR-)                                          
    574 Human: POLR3C (10623, CR605615) 
Mouse: Polr3c (74414, AK043194) 
 
           |-50      |-40      |-30      |-20      |-10      |1        |11       |21       |31       |41       
Human     GGCGCCAACAGCTACCCTGCGGCCCGCCTCCCAGCACCAAAGAGGCGCAG G AA  GGAGAGAC  AAACGGCCCG  CCCGCCGGCTCCA  CAAACAGCCCCTCGCT
                                                                 (INR-)                 (MTE-)                    
Mouse     CCCGCCAGCGG  CCACGCGA  CCCGAGGCCCGCCTCCCGGCACCAAAGGCAG A   GGAGAGCC  CGCCCGCCGGCACCTC  AGACAG  CCCCTGGCCGGTCCTCCAG
                       (BRE+)                                    (INR-)                   (DPE+)                    
    575 Human: T1A-2 (10630, NM_006474) 
Mouse: Gp38 (14726, NM_010329) 
 
           |-50      |-40      |-30      |-20      |-10      |1        |11       |21       |31       |41       
Human     GGAGACCACCTTGCGGCCGACCCCGCTCCCCCGCCTCCTCGGGAGAGA  TA A ATGCT  GACTCCGCTCGGAAAGTTCT  CAACTG  CAAAGTTTGCTGTCCGGC
                                                            (INR-)                       (DPE-)                   
Mouse     TCCGGGACCACC  TGACGCCC  ACCCGCTCCCGCCCCGGGACCGGAGACA  TA A ATGCC  GACTGTGCCGAGAG  GTTGCCAGCTGCC  AAGTTTGCTGTGCTTGC
                        (BRE+)                                (INR-)                     (MTE-)                     
    576 Human: T1A-2 (10630, AJ225022) 
Mouse: Gp38 (14726, NM_010329) 
 
           |-50      |-40      |-30      |-20      |-10      |1        |11       |21       |31       |41       
Human     GCTGCCTAGGGTCTGGGAAGCTCGGGCACCCTCCCTCTCCGGGGCTCCTG C TCCCACCCCTCCGGCCCCCCCA  CCGTCGCGCTCCT  CCAGGCTGGGCCTG
                                                                                        (MTE-)                  
Mouse     GCCTCTGCGAGTCCAGAAAGCCCGGGCACTCTCTGGCGCTGAGACTTTTG C TCAGCGCCTTCCAACCTCCTCC  CGAGCTCTTCCCG  GCTGGGCCTGTGGC
                                                                                        (MTE+)                  
    577 Human: PMVK (10654, L77213) 
Mouse: Pmvk (68603, AA049349) 
 
           |-50      |-40      |-30      |-20      |-10      |1        |11       |21       |31       |41       
Human     GCGGTGTCCCGATTTTAGGGGTAGGGAGAAGTGTCAGCTTCAGGCATCGC G AGGCGTGGCGGCCCCA  TGGCCCCGCTGGG  AGGCGCCCCGCGGCTGGTAC
                                                                                  (MTE-)                        
Mouse     TCGTGCTTGGCTGCATTTCAGGGGTCCGAAGTGTCAGCTTTAGGCCTGGT G AAGCTTGGTGGCACCA  TGGCCCCGCTCGG  AGCCTCCCCGCGTCTGGTGC
                                                                                  (MTE-)                        
    578 Human: PMVK (10654, BC006089) 
Mouse: Pmvk (68603, NM_026784) 
 
           |-50      |-40      |-30      |-20      |-10      |1        |11       |21       |31       |41       
Human     AGGCCAGGCTGTGAGCGAAGGTTCTGGGCGGGGCTGGACTGTTC  TAAGTG A G  TTCGGGTGGGGGAGCTTCACG  AGGGGAGGCTGCT  CTGTGAAGGAACCG
                                                        (INR-)                            (MTE-)                  
Mouse     AAGGCGGGGCCATGACGTCACATCTGGGCGGGCGTGGACTGTTC  TAAGTG C G  TGCTGCGCAGGGGGGGG  AGGGCGGGGGGGG  GCGGGAAAGGTACTTAGA
                                                        (INR-)                        (MTE+)                      
    579 Human: KHDRBS1 (10657, U46751) 
Mouse: Khdrbs1 (20218, AK050520) 
 
           |-50      |-40      |-30      |-20      |-10      |1        |11       |21       |31       |41       
Human     ACTTTGTTCTCCGCG  GAGAAAC  CCAAGGGCGGTGCCGGTGGCTGGCTGCG C ACGCGCGCCGCCTCAT  TTCCGGTGCTCTC  TCTCGCTGGGTCGCTCGGGT
                          (TATA+)                                                   (MTE-)                        
Mouse     ACTTTGTTCTCCGCG  GAGAAAC  TCAAGGGCGGTGCCGGTAGGCGCCTGCG C ACGCGCGCCGCCTCAT  TTCCGGTGCTCTC  TCGCTGGGTCGCTCGGGTCG
                          (TATA+)                                                   (MTE-)                        
    580 Human: GMEB1 (10691, AK000892) 
Mouse: Gmeb1 (56809, BC046983) 
 
           |-50      |-40      |-30      |-20      |-10      |1        |11       |21       |31       |41       
Human     TTAATCCTCGGTGGGACCGGCAATAGGGACGCGTCCCCTTTAAGGCG  CCA G CTCT  CCGGCGGCGGCGGCCGGAAGTGTTGAAGCCCGGCCTGGCGGCGGC
                                                           (INR+)                                               
Mouse     TGTATCCCC  TGCGCGGC  CCGC  AATAAGG  ACGCGTCCCCTTTAAGGCGCCG G CTCGCCCGCCGCGGCGGC  CGGAAGTGCTGGA  GCCCGGCTTGGCGGCGGC
                     (BRE-)       (TATA+)                                               (MTE-)                      
    581 Human: GMEB1 (10691, AK000892) 
Mouse: Gmeb1 (56809, BC046983) 
 
           |-50      |-40      |-30      |-20      |-10      |1        |11       |21       |31       |41       
Human     AGCCCGGCCTGGC  GGCGGCGG  TGGCGGTAGCTGCCGTGGCGGCCTCTGCG C ATGCTCCGTCGCCTGCCCGC  CCTGGCCGCTCGC  CGCCCGCCCGCCCGAC
                         (BRE-)                                                         (MTE-)                    
Mouse     GCCCGGCTTGGC  GGCGGCGG  TGGCGGCGGCCGCCGTGGCGGCTTTGTGCG C ATGCTCCGTCGCCCGCCCGC  CCTGGCCGCTCGC  CGCCCGCCCGCCCGCC
                        (BRE-)                                                          (MTE-)                    
    582 Human: NUDC (10726, BC007280) 
Mouse: Nudc (18221, AK012321) 
 
           |-50      |-40      |-30      |-20      |-10      |1        |11       |21       |31       |41       
Human     AGGAAGGCGGGAAGAGAGGAGGTGGAGGCGGGCCTGGGCAGCCGCGCGCG T GCGTGTTTCCGGCTCCGCTGCGG  AAGGCGGACGACT  AGAGTCGTTGGGC
                                                                                         (MTE+)                 
Mouse     GAGGCGGGAAGAAAGCCGGGAAGGGCGAACCCGCACGGCCGGGCGGCTCC T GCGTGTTTCCGGCTCCGCT  GCGGAAGGCTAGC  GAGGGGAGCCGTTGGGC
                                                                                     (MTE-)                     
    583 Human: PHTF1 (10745, NM_006608) 
Mouse: Phtf1 (18685, NM_013629) 
 
           |-50      |-40      |-30      |-20      |-10      |1        |11       |21       |31       |41       
Human     CCAGGGCAGCGTAAATTCACGC  AAACATT  GATTCGTTCGCTCA  ACAAACA T   TGGGAACCTAAGTGCCAGGCTTGTGCCAGGCCCCAGTCTCTGACCTCAT
                                 (TATA-)                 (INR+)                                                   
Mouse     CCGCGTTCCAGG  CCGCGCAA  ATTCACCAACATTTGTCCATCCAACAA  GAA C GGAG  AACTCGAGCGAAGGCTCGT  GCCCTGCACTAGT  CTCGTCCCCCCAA
                        (BRE+)                               (INR-)                          (MTE-)                 
    584 Human: MASP2 (10747, Y18284) 
Mouse: Masp2 (17175, AJ250369) 
 
           |-50      |-40      |-30      |-20      |-10      |1        |11       |21       |31       |41       
Human     GACAAACAGATCAAAGGTGAGACCAGCGTAGGGCTGCAGACCAGGCCAGG C   CAGCTGGA  CGGGCACACCATGAGGTAGGTGGGCGCCCACAGCCTCCCTG
                                                               (INR-)                                           
Mouse     TGCTGGACAAACAGATCAAAGGTGATAGGCGCTGGACCTGCAGAGCTAGG T G  GCACACCA  TGAGGTGGGTGGGCGACCCATAGCTTCTGGGGAAGCTGAA
                                                                (INR+)                                          
    585 Human: JARID1B (10765, NM_006618) 
Mouse: Jarid1b (75605, NM_152895) 
 
           |-50      |-40      |-30      |-20      |-10      |1        |11       |21       |31       |41       
Human     GAGACGTC  GTCGTCGG  AGGCT  GAAAAAG  CCCAAGGTGCTGCCGTTGCCCG T A  CAACTCGG  ACTTGCTGTTGCTCGAGCCG  CGTCTG  CACGGGTCTCGGAC
                    (BRE-)        (TATA+)                           (INR+)                       (DPE-)               
Mouse     AGACGTCGTCGGAGGAAGGCT  GAAAAAG  CCCTAGGTGTTGCCGTTGCTAG T A  GAAGTCGG  ACCTGT  CGCCCGAGCTGTG    CGTCTG  CACGGGTTTCGGACG
                                (TATA+)                           (INR-)             (MTE-)     (DPE-)                
    586 Human: JARID1B (10765, AY370676) 
Mouse: Jarid1b (75605, NM_152895) 
 
           |-50      |-40      |-30      |-20      |-10      |1        |11       |21       |31       |41       
Human     CCTGGGTTTTACTGCACCTACCATTGTGTACTGACGTGATTGAA  TGATTG G C  TTATGGGACTGATCCTTTCTACCAGACT  TAACTT  CTTCAGGGCAGGAA
                                                        (INR-)                               (DPE-)               
Mouse     GGGTTTTACTAAGCAAATCTTTCGTGCCAAAATATATGACGTGATTGAT  G G ATTGTA  ACTCTGTTCTCCCTACTAGACTGT  TAACTT  CTTGAGAGGAAGA
                                                             (INR-)                           (DPE-)              
    587 Human: FUSIP1 (10772, AY048592) 
Mouse: Fusip1 (14105, AK005295) 
 
           |-50      |-40      |-30      |-20      |-10      |1        |11       |21       |31       |41       
Human     GTCTCGCGAGAAGAGTCGGTTGCCGTAGCAGAGCCCTCTAGCTG  TGTGTG T C  TGAGGCTCGGCCGCC  TGAGCCGCGGACG  GTTTGCTGAGCCCGTTAGTG
                                                        (INR-)                      (MTE+)                        
Mouse     GTCTCGCGAGAAGAGGCAGTTGCCGTAGCGGAGCCCTCTGGGTCTGTG  CG A GTGTG  GTGTGAGTGGATG  TGAGCCGCCGCCG  GAGCTGCGGACGGTTTGC
                                                            (INR-)                    (MTE+)                      
    588 Human: SDCCAG8 (10806, AF161348) 
Mouse: Sdccag8 (76816, AF250729) 
 
           |-50      |-40      |-30      |-20      |-10      |1        |11       |21       |31       |41       
Human     CCCCAAGAGCTCTGTGCGGGATTCTAGGCTCCCCTGTGACAGCCGCGGCA G GAAGCAGGCGGGCGCTCCCCGGCCACAGGCCTGTTGTTCTCGGAAGGGA
                                                                                                              
Mouse     CCCTAGGAGCTCGGTCCGGGGTTCCTGGTTCCCTAGTGACAGCAGCGACA G GAAGCAGGCAGGCATCCCCTCAT  CCCCAGCGCTAGG  CCTGTCCTCGGAG
                                                                                         (MTE-)                 
    589 Human: WDR3 (10885, BC058836) 
Mouse: Wdr3 (269470, AA795258) 
 
           |-50      |-40      |-30      |-20      |-10      |1        |11       |21       |31       |41       
Human     TCCTAGAAGGCAAAGGGAACCTCTGGCGGAAGAGGCTTCCGGAACGGACC C G  GAAGGGGC  AGGTCTT  GTGGGCGGGTCCA  ATCGGCTGGGAGCCTCGTGG
                                                                (INR-)              (MTE-)                        
Mouse     CCGCCTAGAAGGTAAGGGAAACGCCGGGCGGAAGAAACTTCCAATCGGAC C G  GAAGTGGC  GGGTCTCGTAG  GCAGCGCTGTCTG  CTGGAGCCTCGTGGAG
                                                                (INR-)                  (MTE+)                    
    590 Human: JTB (10899, AB016493) 
Mouse: Jtb (23922, BU697939) 
 
           |-50      |-40      |-30      |-20      |-10      |1        |11       |21       |31       |41       
Human     CAACCGCCATTTGCCGTTCTCGCAAAGACTACCAAGACCACAATGCAACG G GGCGCCGAGCTAATTCCCAGTGA  GCAGCAGGCGAGG  CGCCACCGACGCG
                                                                                         (MTE+)                 
Mouse     ACCACCGGTCCCGCCGGCTCTGCAAACACTACAAGGACCACACTGCCGCG C GGCGCCCAGCTGAAGAGTCGCGT  GATCCT  GCGCAAACGCAGTGAGATGC
                                                                                     (DPE-)                     
    591 Human: MAN1A2 (10905, AF053615) 
Mouse: Man1a2 (17156, U03457) 
 
           |-50      |-40      |-30      |-20      |-10      |1        |11       |21       |31       |41       
Human     GGCTTGCCTGATCTACCCCTAGG  AATGAAG  AGGAGGCTTGTAATAATCCG A T  GAAGTACA  GATGTT  GAAGAGGATATCG  CA  GGACCT  AAACTTGTGATCG
                                  (TATA+)                         (INR-)             (MTE+)       (DPE+)              
Mouse     GGCTCGCCCGATCCACTCTCGGG  AATGAAG  AGGAGACTCGCGA  TCATCCG G   TGAGGCACCGAGAGC  CAAATGGCGAGA  A  GATCT  GACGGGACAGAAACCA
                                  (TATA+)                (INR+)                      (MTE+)    (DPE+)                 
    592 Human: SF3A3 (10946, NM_006802) 
Mouse: Sf3a3 (75062, NM_029157) 
 
           |-50      |-40      |-30      |-20      |-10      |1        |11       |21       |31       |41       
Human     TTTGGTGCAGCCTGATGCGCAACGTGGGGACTCAGGCGCGCTGGGCGGCA   G GAGTTGC  TTCCGGCCGTGTTGGT  GGTCTG  AATTGAGAAGCCGCGACTAA
                                                              (INR-)                   (DPE-)                     
Mouse     CTTTGGTGCAGCCGGATGCTGCGCGCTGAGACGCTGGCTGAAAGGCGGCA G GC  GGTGTTTC  CGGCAGTGTTAGT  GGTCCG  CCTTGAAACGCCGTGGCCAA
                                                                 (INR-)                (DPE-)                     
    593 Human: AF1Q (10962, NM_006818) 
Mouse: AI839562 (56772, AB000733) 
 
           |-50      |-40      |-30      |-20      |-10      |1        |11       |21       |31       |41       
Human     GGGATTGGGAGAA  GGGGTGGA  AAAATACTCTGATTCTTAAAAATAC  TTTG T AAC  CTAAAGTCCTTAAATTGTGGAAGAAAGGAATACTCCTCCTTTCCAT
                         (BRE-)                             (INR-)                                                
Mouse     GCAGGCGCTGAGAGGACGATGCGCTGGGGCCCTTTCTGAGTCTCCC  AAAG T CCC  CTTTAGTCCTT  ACAGTGGGAAAGG  AGGGCCATTCTGTTGTTTAACC
                                                          (INR-)                  (MTE+)                          
    594 Human: AF1Q (10962, NM_006818) 
Mouse: AI839562 (56772, AB000733) 
 
           |-50      |-40      |-30      |-20      |-10      |1        |11       |21       |31       |41       
Human     CCTTCTTAGGAGGGGCTGCATTGCAGGGGGAGAGTGAACTGACAGAC  TCA G TCAC  TGAAGAGGGAAAAGGAGTGAGA  AGACAA  AGCCGTCAAAGCCCCAA
                                                           (INR+)                         (DPE+)                  
Mouse     GCCCTTGCTAGGAGGGGCTGCATTGCAGGGGAGACCCAGCGGCAGATTCT G TC  ACAGACGA  GGGAGAAGGCGTGAGG  AGACAA  AGCCGTCACATCCGCGA
                                                                 (INR+)                   (DPE+)                  
    595 Human: EBNA1BP2 (10969, CR605280) 
Mouse: Ebna1bp2 (69072, BU936428) 
 
           |-50      |-40      |-30      |-20      |-10      |1        |11       |21       |31       |41       
Human     CTGGGCGTGGGAGAGGGGGCCTTACACCCCTGGGAGTGACCGTG  ATTGTG T C  CTGCAGTTGCAGGATGCGTTTT  CCCGAGGGCTTCT  GAAGCCAGGCCTC
                                                        (INR-)                             (MTE-)                 
Mouse     AGGGGCTGGGACGGGGACTT  TATTTAG  CTCTTTGGTACTGAGGCTCCCC  G G TCTGTA  GTTGCAGGATGCGTTTT  CCCGCGGACTCCT  GAAGCCAGGTCTC
                               (TATA+)                         (INR-)                        (MTE-)                 
    596 Human: EBNA1BP2 (10969, NM_006824) 
Mouse: Ebna1bp2 (69072, BC054723) 
 
           |-50      |-40      |-30      |-20      |-10      |1        |11       |21       |31       |41       
Human     CCGGACACTTTCAAACGCCGTAG  CGGTTCC  TATAGCAACGACAAACCG  GA A GTATG  GTTTGCCGCCG  GAAGCGGAAGTCC  CAATCAAAAGTTGAGCAGTA
                                  (TATA-)                     (INR-)                  (MTE+)                        
Mouse     CGGGCACGCCCAAACGCTAGAG  CAGTTCC  TATAGCAACGAGAAAACCG  GA A GTGCG  GCTATGAACCCG  GAAGCGGAAGTCC  CTGTTGAAAAGGCAGAGGC
                                 (TATA-)                      (INR-)                   (MTE+)                       
    597 Human: KIF2C (11004, NM_006845) 
Mouse: Kif2c (73804, AK015046) 
 
           |-50      |-40      |-30      |-20      |-10      |1        |11       |21       |31       |41       
Human     TAAGGACGCTTGCGCG  CGGGATT  TAAACTGCGGCGGTTTACGCGGCGTTA   A GACTTCG  TAGGGTTAGCGAAATTGAGGTTTCTTGGTATTGCGCGTTTCT
                           (TATA-)                              (INR+)                                            
Mouse     TAGGGAGGCT  GGCGCGCG  GGA  TTTAAAC  TGCAGCGGTTTAGGCGTTGTTA   A CACAGCG  CAGTATTAGCAG  AGTCGTGGTTTCC  AAGCTTCTTTCATTTGT
                      (BRE-)      (TATA+)                         (INR+)                   (MTE-)                     
    598 Human: IL24 (11009, U16261) 
Mouse: Il24 (93672, NM_053095) 
 
           |-50      |-40      |-30      |-20      |-10      |1        |11       |21       |31       |41       
Human     CCCTCCCCCATCAGC  CCCCATA  TATATGCCCAAATCTCCACAAAGCCTTG C TTGCCTGCAAACCTTTACTTCTGAAA  TGACTT  CCACGGCTGGGACGGGA
                          (TATA-)                                                         (DPE-)                  
Mouse     TCCTCTGCCCCTCCCTGCCAGA  CCCCTTA  TATACAGTTCTCCCAGCCTTG C TTACCCTCAGTCTTTCACTTTTGAAATCATTTCCACAGCTGAGAAGGAG
                                 (TATA-)                                                                        
    599 Human: TDRKH (11022, AF227192) 
Mouse: Tdrkh (72634, AK031051) 
 
           |-50      |-40      |-30      |-20      |-10      |1        |11       |21       |31       |41       
Human     CGAGCAGGGTGTTGATTGGTGCTGTCGGGCTGCGGGGGCGGGGAAGTGGC G GCTGAAGCGCCGC  CGGCGGGGCTCAC  TGTGGTGGTGTGAGTGGGAGGCG
                                                                               (MTE-)                           
Mouse     AAAGTGAGCGTTGATTGGTGAGGATGCGCTGCGGGGGCGGAACAAGCGGC T GCTGAAAAGCGACCCGCTAGG  CGGCTGCGCTGAG  GGAGCGACAGGGCGG
                                                                                       (MTE-)                   
    600 Human: RER1 (11079, NM_007033) 
Mouse: Rer1 (67830, AA003952) 
 
           |-50      |-40      |-30      |-20      |-10      |1        |11       |21       |31       |41       
Human     GCGGAAATGGTTGGAGCCCTTGGCCCCGCCCTCGCGCCATCTTGGGGGCC C TGGAGGCGGCGCCGCGGAGGAC  GGAGCGGAAGTGC  TCGCTGCAGCTTCC
                                                                                        (MTE+)                  
Mouse     AGCGGAAATGGTTGGAGCTTGGGCCCCGCCCCGCGGCCATCTTGGCGGCT G GAGGCGCCGCGGGAC  GGAACGGAAGTGC  CTGTGCAGCTTCCCGGATCCG
                                                                                 (MTE+)                         
    601 Human: DNAJB4 (11080, U40992) 
Mouse: Dnajb4 (67035, BC017161) 
 
           |-50      |-40      |-30      |-20      |-10      |1        |11       |21       |31       |41       
Human     AGGAGGCTGTCTCCTGTGTAGT  GTATATT  TATCTGTAAGTGAGCCGTTGG G GAAGGATTGAATACAGAGACGC  TGTCTG  CTTGCTGCCTTAAGACAGCTA
                                 (TATA-)                                              (DPE-)                      
Mouse     GCTGAGGCCGTCTCCGTGCGGT  GTCTATT  TATCTGTAAGTGAGCTGCCGG G GA  GGAATTAA  ATAGAGAC  GCTGCCTGCTGCC  TCTGGACAGCTGAGCCGA
                                 (TATA-)                           (INR-)               (MTE-)                      
    602 Human: DSCR1L2 (11123, NM_013441) 
Mouse: Dscr1l2 (53902, AK043916) 
 
           |-50      |-40      |-30      |-20      |-10      |1        |11       |21       |31       |41       
Human     AGGGTTGTACCCGCCAGGGCGGGACCCAGCCTGTGCCGTCCCACACCTTG T GCTAGCTCCAGCTCAGCTACCTGCGGCCGCCTCTCCTCGGGCAATGAGG
                                                                                                              
Mouse     TGCTGGGTGCAATGGAGGGAGGGATGGGGCGCGCGCAGTCACACACCTGG T GACACCCATAGCACAGCCACGTCGCCATCGCCTCTACTGGGCGACGGGA
                                                                                                              
    603 Human: FAF1 (11124, BC004970) 
Mouse: Faf1 (14084, BC065098) 
 
           |-50      |-40      |-30      |-20      |-10      |1        |11       |21       |31       |41       
Human     GCGACCTCGATGACAGGCAAAATGTGCGACAGCCGTGGCGCTGGCCAACC A GGGGCGGAGGCGGCGGCCAGGGAGGAAGCGGAGGAGGCGGAGGCGGCCG
                                                                                                              
Mouse     GCGACCTCGATGACAGGCAAAATGTGCGACAGCCCAGGCGCCGGCCAACC A GGGGCGGAGGCGGCGGCCGGGGAGGAAGCGGAGGAGGCGGAGGCGGCTG
                                                                                                              
    604 Human: TNRC4 (11189, AY165003) 
Mouse: Tnrc4 (78784, AK019578) 
 
           |-50      |-40      |-30      |-20      |-10      |1        |11       |21       |31       |41       
Human     TGACATCACTGATGTCAGGGGAAAGGAGGTGGGAGTGGGGAGGGG  GGTGT G GA  GGGGGGAGGTTTTTGTTGAGG  AGAGCGCGGCCGG  AGAGCAGAGCTCC
                                                         (INR-)                            (MTE+)                 
Mouse     GACATCACTGATGTCAGGGGAAAGGAGGTGGGAGTGGGGAGGGGG  GGTGT G GA  GGGGGGGGAGGTTTTTGTTGA  GGAGAGCGCGGCC  GGAGAGCAGAGCT
                                                         (INR-)                            (MTE+)                 
    605 Human: DDX20 (11218, BC034953) 
Mouse: Ddx20 (53975, AF220454) 
 
           |-50      |-40      |-30      |-20      |-10      |1        |11       |21       |31       |41       
Human     CGTGACGCACCGGGCGCAT  CACAAAG  AACGGCGAGAGGGCGGTGGCGCCG G GGGCACGGCTGGG  CGGCTCCGCCCAG  AAGAGGGCCGAGAGGCGGGGCGG
                              (TATA+)                                            (MTE-)                           
Mouse     CGACCGATTCGCGAGCTGCGT  CACAAAG  CGCGTCCTGGGCGCTCG  GGTCT G GA  GCCCCGCCGCGCG  GCCAGGCACTCAG  AGCCGCGGGAGGCCGGCAGCG
                                (TATA+)                    (INR-)                    (MTE-)                         
    606 Human: DUSP10 (11221, NM_007207) 
Mouse: Dusp10 (63953, BC025066) 
 
           |-50      |-40      |-30      |-20      |-10      |1        |11       |21       |31       |41       
Human     AGTCCGCCCCCTCAGAACC  TTACATT  TACAGTGTAGCAAAAGAG  AAAGTA A C  TATGTTGCTGCTGGAGATCAAT  GAAGCCGAGTGAA  TGGGGGCTGAATG
                              (TATA-)                     (INR-)                             (MTE+)                 
Mouse     ACCCCGCCCCCTCCAAAGCTTA  CATTTAC  GCTGTAGCAAAAGAG  AAAGTA A C  TCTGTGTTGCTGCTGG  GGATCAATGAAGC  TGAGTGAATGGGGGCGCAC
                                 (TATA+)                  (INR-)                       (MTE+)                       
    607 Human: PADI2 (11240, BC009701) 
Mouse: Padi2 (18600, AK033760) 
 
           |-50      |-40      |-30      |-20      |-10      |1        |11       |21       |31       |41       
Human     CCCCGCCCACCGGCCGCTG  GATAAGG  CTGCGCGCGGGGCCGTGGGGCGCA G GCTGCTGGAGAAGGCGCACCTGC  TGCAGGTGCTCCC  GGCCGCCCCGGAC
                              (TATA+)                                                      (MTE-)                 
Mouse     CCCGCCCCCGGCTGCTG  CTTAAGG  CCGCCGGCTGCTCGGCAGCGAGCGCA G GCGCAGCAGCGGAGGGCTTACCT  GCAGCAGGTGCTT  ATTCCTGGGAGCA
[truncated: 5,562,637 more chars]
