# Supplementary material for: Genome-wide analysis of core promoter elements from conserved human and mouse orthologous pairs
Source: BMC Bioinformatics. 2006 Mar 7;7:114. doi: 10.1186/1471-2105-7-114 (PMC1475891; doi:10.1186/1471-2105-7-114)
Supplement: Additional File 2 — Supplementary Table 2; Enumeration of core promoter elements in EPD with and without considering conservation in the mouse genome. [file 1471-2105-7-114-S2.doc]

**Supplementary Table 2** Enumeration of core promoter elements in EPD with and without considering conservation in the mouse genome

| Motif  (core, PWM score cutoffs) | Number of promoter elements found in 624 promoter sequences from the EPD | | Number of promoter elements that are conserved in the orthologous mouse promoters | |
| --- | --- | --- | --- | --- |
|  | Real sequences | Randomized sequences | Real sequences | Randomized sequences |
| BRE  (N/A, 0.81) | 179 (28.7%) | 149 (23.9%) | 125 (20.0%) | 86 (13.8%) |
| TATA  (0.73, 0.58) | 222 (35.6%) | 80 (13.0%) | 189 (30.3%) | 40 (6.4%) |
| INR  (0.72, 0.62) | 418 (67.0%) | 334 (53.5%) | 391 (62.7%) | 306 (49.0%) |
| MTE  (0.79, 0.53) | 408 (65.4%) | 371 (59.5%) | 349 (55.9%) | 300 (48.1%) |
| DPE  (0.92, 0.92) | 137 (22.0%) | 134 (21.5%) | 86 (13.8%) | 63 (10.1%) |
