# Supplementary material for: Genome-wide analysis of core promoter elements from conserved human and mouse orthologous pairs
Source: BMC Bioinformatics. 2006 Mar 7;7:114. doi: 10.1186/1471-2105-7-114 (PMC1475891; doi:10.1186/1471-2105-7-114)
Supplement: Additional File 3 — Supplementary Table 3: Composition of orthologous mouse-human core promoters; Alignment of Mouse and Human sequences with highlighted conservation of core promoter elements, in which the TSS of mouse promoter is experimentally supported. [file 1471-2105-7-114-S3.zip › Jin_CorePromoters_Mouse.html]

 
   
   Composition of orthologous human-mouse core promoters 
   Entries are in the following format:  Organism: symbol (GeneID, Accession)   
   Hover cursor over elements to see their scores  
  1 Mouse: Chrnd (11447, X66531) 
Human: CHRND (1144, CR623061) 
 
           |-50      |-40      |-30      |-20      |-10      |1        |11       |21       |31       |41       
Mouse     CTTCTTTCCAAACC  CCTAAAC  CACCAGCACCTGTCCCCTTGCTTGCC  TCA T TCCA  CAGCCAACAGG  CTGAAGGGAAGAC  AAACCCTAGTCAGTCAGAGGT
                         (TATA+)                             (INR+)                  (MTE-)                         
Human     CGTTGACCCCCTCCTCCCCCAACACCTGTCCCCTCTCCCGCCCACCC  TCA T TCCA  CAGCCCTGTAGACAGGAGGGGC  AGATGC  ACGTCCCAGTCAGAGGG
                                                           (INR+)                         (DPE+)                  
    2 Mouse: Chrng (11449, NM_009604) 
Human: CHRNG (1146, X01715) 
 
           |-50      |-40      |-30      |-20      |-10      |1        |11       |21       |31       |41       
Mouse     GCAACCCCCCCCCCCA  GGCCTGTA  GAGCTACTGTCCTGCTCTTCACTGGG C A  GAACTGAG  GCACCATGCAAGGGGGCCAG  AGACCT  CAGCTCCTCTTGCT
                            (BRE-)                                (INR-)                       (DPE+)               
Human     TCTCTCTCTGCCCCAGACCTTGGAGCTGTTGTCCCACCCCTGTCACT  GCA G AGAG  CTGAGGCACCATGCATGGGGGCCAGGGGCCGCTGCTCCTCCTGCT
                                                           (INR+)                                               
    3 Mouse: Adora1 (11539, AF133099) 
Human: ADORA1 (134, AK127752) 
 
           |-50      |-40      |-30      |-20      |-10      |1        |11       |21       |31       |41       
Mouse     GAAACAGGGGCACTACCTC  TTTAAAG  GCGTCCGGGGCTGAGCCTCTGCCA C AC  CATGTGAT  TGCTTGAA  AGGCCGGGCTGGG  AGCGCAGCGGCGGGAGCC
                              (TATA+)                              (INR-)               (MTE-)                      
Human     GAAACAGGGGCGCTACCTC  TTTAAAA  GCGTCCGGGGCTGAGTCTCTGCCG T AC  CATGTGAT  TGCTTGAA  AGGCCGGGCTGGG  AGCGCTGCGGCGGGAGCC
                              (TATA+)                              (INR-)               (MTE-)                      
    4 Mouse: Parp1 (11545, AK028042) 
Human: PARP1 (142, CB998389) 
 
           |-50      |-40      |-30      |-20      |-10      |1        |11       |21       |31       |41       
Mouse     CCGCCCTCCGCGCGCGAGCTTGGGGGGGCGGTCCCGCCGCTAGGCATCAG T   AATCTATC  CTGAGCGGAGGCGGC  TCTGCACGCTTGC  GGGGGAAACCGAC
                                                               (INR-)                      (MTE-)                 
Human     CCCGCCCCGTGGACGCGGGTTCCGTGGGCGTTCCCGCGGCCAGGCATCAG C   AATCTATC  AGGGAACGGCGGTGGCCGGTGCGGCGTGTTCGGTGGCGGCT
                                                               (INR-)                                           
    5 Mouse: Parp1 (11545, AF126717) 
Human: PARP1 (142, CB998389) 
 
           |-50      |-40      |-30      |-20      |-10      |1        |11       |21       |31       |41       
Mouse     CGTTGGGGTTTTCCTTTCTCCCTGTCTGGTTGTAAACAGAGGAGGTGGAG A AGATGAGTAAGAAGATGGAG  GAAGTGAAAGCGG  CCAACGTTCGAGTTGT
                                                                                      (MTE+)                    
Human     AACTTGTATTTTGGTTTTT  TTTCCTT  TCAATTTGAACAGAGGAGGTGGAA A AGATGAATAAGAAGATGGAGGA  AGTAAAGGAAGCC  AACATCCGAGTTGT
                              (TATA-)                                                     (MTE-)                  
    6 Mouse: Adss (11566, AK028060) 
Human: ADSS (159, NM_001126) 
 
           |-50      |-40      |-30      |-20      |-10      |1        |11       |21       |31       |41       
Mouse     GCGGCCCTCGGGGCGGGGGCGGGGCCGGCCTCTGGCTCCTTCTTCCTCCG   C ATGTGGC  TGGTGGCGGCGCAGC  AGTTGG  GTTCACGTTCCGTTCTCCTGC
                                                              (INR-)                  (DPE+)                      
Human     CGGCCCGGCGGGGCGGGGGCGGGGCCGGCCTCTGGCTCCTTCTTCCTCTG   C ATGTGGC  TGGCGGCCGCAGAGCAGTTC  AGTTCG  CTCACTCCTCGCCGGC
                                                              (INR-)                       (DPE+)                 
    7 Mouse: Agxt (11611, NM_016702) 
Human: AGXT (189, M61755) 
 
           |-50      |-40      |-30      |-20      |-10      |1        |11       |21       |31       |41       
Mouse     AGGACAAACATTGATCAGG  GTTAAAT  TGACAATAAAAGGGCTGGAGAAAC A G  GGACTCAT  CAACCAGGCCTGGCCTCTG  AGTTCA  ACGCAGAGCTAGCTG
                              (TATA+)                             (INR-)                      (DPE+)                
Human     CGCAGCACAAGCACAG  ATAAGCCT  CAGGGAACAAAAGGCAGGGCTG  CCAC G GAA  GCCCATCCAC  CAATCCTCACCTC  TCACCTCTGTGTCCGCCCTGCTG
                            (BRE+)                          (INR+)                 (MTE+)                           
    8 Mouse: Akr1b3 (11677, U93230) 
Human: AKR1B1 (231, CR601476) 
 
           |-50      |-40      |-30      |-20      |-10      |1        |11       |21       |31       |41       
Mouse     ACACCAGGGTAAGGTACTCTGACAAGCACCAAGAACCTTAAAAGGG  GCAC A TTT  GCCGGTCCGGGTTTGTAAC  GTGCAGCGATCAT  GGCCAGCCATCTGG
                                                          (INR+)                          (MTE-)                  
Human     TGCCGACCTCACGGGCTA  TTTAAAG  GTACGCGCCGCGGCCAAGGCCGCAC C   GTACTGGG  CGGGGGTCTGG  GGAGCGCAGCAGC  CATGGCAAGCCGTCTCC
                             (TATA+)                             (INR-)                  (MTE+)                     
    9 Mouse: Prdx6 (11758, AF093854) 
Human: PRDX6 (9588, NM_004905) 
 
           |-50      |-40      |-30      |-20      |-10      |1        |11       |21       |31       |41       
Mouse     GCCCACTCGGCCAGCACTGATCTAGGTCTCCGCAGGAGCCCGCCCGCTGC   T CACTGCT  GCGGCTGCGCCTCCT  TGTTCTCAGCGTC  ACCACTGCCGCCAT
                                                              (INR-)                      (MTE+)                  
Human     TCCTCCGCGCGCTGGGACAGGCTGCTTCTTCGCCAGAACCAACCGG  TTGC T TGC  TGTCCCAGCGGCGCCCCC  TCATCACCGTCGC  CATGCCCGGAGGTCT
                                                          (INR+)                         (MTE+)                   
    10 Mouse: Apoa2 (11807, M79361) 
Human: APOA2 (336, CR595207) 
 
           |-50      |-40      |-30      |-20      |-10      |1        |11       |21       |31       |41       
Mouse     GGGGGTGGGGGAGTATATGTG  TATATAG  CCCCTACCTCCAGTCAAGCCCA G A  GTAGACGG  GAAGGACTGC  AGCACAGAATCGG  TATGCTGAGGGAACAGA
                                (TATA+)                           (INR+)                 (MTE-)                     
Human     GGGTGGGTAAACAGACAGG  TATATAG  CCCCTTCCTCTCCAGCCAGGGCAG G C  ACAGACAC  CAAGGACAGA  GACGCTGGCTAGG  TAAGATAAGGAGGCAAG
                              (TATA+)                             (INR+)                 (MTE-)                     
    11 Mouse: Astn1 (11899, NM_007495) 
Human: ASTN (460, NM_207108) 
 
           |-50      |-40      |-30      |-20      |-10      |1        |11       |21       |31       |41       
Mouse     CCCTTGCTTGCTCGCTGCTCCCTCCCTGCTGGCTGCCTCCCCCACCATCG C AGCGCTGGGAGGAAGGC  GGCCAGGGCTCA  A  GATGG  CTTTAGCCGGGCTC
                                                                                   (MTE-)    (DPE+)               
Human     CCGCGCTCGCTCCCTCGCTCCCTCCCCGCTCGCTTCCTCCCCCACCATCG C AGCGCTAGGAGGAAGGC  GGCCGGGGCTCA  A  GATGG  CTTTAGCCGGGCTC
                                                                                   (MTE-)    (DPE+)               
    12 Mouse: Serpinc1 (11905, NM_080844) 
Human: SERPINC1 (462, U11270) 
 
           |-50      |-40      |-30      |-20      |-10      |1        |11       |21       |31       |41       
Mouse     CCCTCCCCCACCTGGCCTCTGGACCTCTCAGATTTAGGGGAAAGAA  CCAG T TTT  CGGAGTGATCGTCTCAGTCAGCAC  CATCTC  TGTAGGAGCATCGGCC
                                                          (INR+)                           (DPE-)                 
Human     GCCCCACCCTGTCCTCTGGAACCTCTGCGAGATTTAGAGGAAAGAA  CCAG T TTT  CAGGCGGATTGCCTCAGATCACAC  TATCTC  CACTTGCCCAGCCCTG
                                                          (INR+)                           (DPE-)                 
    13 Mouse: Atf3 (11910, BC019946) 
Human: ATF3 (467, CR614862) 
 
           |-50      |-40      |-30      |-20      |-10      |1        |11       |21       |31       |41       
Mouse     TGCCAACGCGAGGGC  TTTAAAA  GGGGTGATGCAACGCGCTCCCAGCC  ACA G TCTC  ACTCAGCGAGACGCCGC  GCACGGTGCTTCC  CCAGTGGAGCCAATC
                          (TATA+)                            (INR+)                        (MTE-)                   
Human     CGCCAGCCTGAGGGC  TATAAAA  GGGGTGATGCAACGCTCTCCAAGCC  ACA G TCGC  ACGCAGCCAGGCGCGCA  CTGCACAGCTCTC  TTCTCTCGCCGCCGC
                          (TATA+)                            (INR+)                        (MTE-)                   
    14 Mouse: Atp1b1 (11931, AK010677) 
Human: ATP1B1 (481, BG706419) 
 
           |-50      |-40      |-30      |-20      |-10      |1        |11       |21       |31       |41       
Mouse     TCCCCCTCCTCCCGCTCTGCCTAGGCTGCTCCGCGGCGCGCCTC  GCACTC G G  AGAGCCGCAGCGGCAGCGGCG  CGTCTC  GCTTTCGGAGACAGAGCCGGG
                                                        (INR+)                        (DPE-)                      
Human     CCCCCTCCTCCTGCTCCTGCCTTGGCTCCTCCGCCGCGCGTCTC  GCACTC C G  AGAGCCGCAGCGGCAGCGGCG  CGTCCT  GCCTGCAGAGAGCCAGGCCGG
                                                        (INR+)                        (DPE-)                      
    15 Mouse: Atp1b1 (11931, AK010677) 
Human: ATP1B1 (481, NM_001677) 
 
           |-50      |-40      |-30      |-20      |-10      |1        |11       |21       |31       |41       
Mouse     GACATCTTGACATGTGAGCTGGGTCTTCCCAAGTGCTCTGTCATTCCAA  G A ATTCTA  CAGTATAGGGTAGGGGTGCCCCCAGTGAGACAGAACAGTAAGC
                                                             (INR-)                                             
Human     AACATCTTGCCCTATGCGCTAGGTTTTCCCAAAGGTCCTTTGGCTCCA  AG A ATTTT  ACAGCAGA  ACAAAGGGATCGA  TACCCCCAAGGGGGCATAAAAGC
                                                            (INR+)               (MTE-)                           
    16 Mouse: Bard1 (12021, NM_007525) 
Human: BARD1 (580, NM_000465) 
 
           |-50      |-40      |-30      |-20      |-10      |1        |11       |21       |31       |41       
Mouse     CCATTTCCGGAACTTGGACCGCATTACTAAAGCCCCGCCCTCAGGCCTTG A G  GGCGTGGC  CTAAAGCCGGGTCCTTC  CATCTC  TGAGTTTTACCGCGTTT
                                                                (INR-)                    (DPE-)                  
Human     ACGCCGGGACTTTGGCAAGTTTCAGCCTCCAGCCCCACCCCTAGGTCCCG C   CCACTCGG  CCAGCGGCTGGCTCTCGCGGCCCCGCCCCTGTGCCCTGCGA
                                                               (INR+)                                           
    17 Mouse: Bcl2 (12043, NM_009741) 
Human: BCL2 (596, CD685722) 
 
           |-50      |-40      |-30      |-20      |-10      |1        |11       |21       |31       |41       
Mouse     AAAAAGCAGAAAGGAATTTG  AATAAAA  ATTTCCTGCATCTCATGCCAACG G GGAAACACCAGAAT  CAAGTGTTCGGTG    TAACTA  AAGACACCCCTTCATC
                               (TATA+)                                            (MTE+)     (DPE-)                 
Human     AAAGATCCGAAAGGAATTGG  AATAAAA  ATTTCCTGCATCTCATGCCAAGG G GGAAACACCAGAATCAAGTGT  TCCGCGTGATTGA  AGACACCCCCTCGTC
                               (TATA+)                                                   (MTE-)                   
    18 Mouse: Bcl2 (12043, M16506) 
Human: BCL2 (596, X06487) 
 
           |-50      |-40      |-30      |-20      |-10      |1        |11       |21       |31       |41       
Mouse     GAGGCCGGGCCCCCCGCCCCGCGCCCGCCCCCTTCCTCCCCGGCCCGTCC T CCCGCCCCGCGTCCGGGTCCCCGCCGAGCCGCCGCCGCCAACACCGAGG
                                                                                                              
Human     AGGACAGGCACCACAGCCCCGCTCCCGCCCCCTTCCTCCCGCGCCCGCCC C TCCGCGCCGCCTGCCCGCCCGC  CCGCCGCGCTCCC  GCCCGCCGCTCTCC
                                                                                        (MTE-)                  
    19 Mouse: Bmpr2 (12168, AF003942) 
Human: BMPR2 (659, CD644422) 
 
           |-50      |-40      |-30      |-20      |-10      |1        |11       |21       |31       |41       
Mouse     CTCAGTGCTCCCATCGAGTCCCCGCCCTCCCCCCCCCCCCCCCCCCG  GCA C TCTG  GATATGTTTTCTCCC  AGATCTGGGTATT  TTTTTGATATCGTGAAA
                                                           (INR+)                      (MTE+)                     
Human     TAGTTCTGACCCTCGCCCCCCGACCCCGGATCGAATCCCCGCCCTCCGCA C C  CTGGATAT  GTTTTCTCCC  AGACCTGGATATT  TTTTTGATATCGTGAAA
                                                                (INR+)                 (MTE-)                     
    20 Mouse: Btg2 (12227, M64292) 
Human: BTG2 (7832, CR604962) 
 
           |-50      |-40      |-30      |-20      |-10      |1        |11       |21       |31       |41       
Mouse     TCCCCGCCTCCCCGAGTGG  TATGAAA  GGCGCAGCCCGGGGAAAGTCCGG  G C AGAGCC  CGAGAGGTGGCCAGACCGTCATCATCGTTCTAATACAGCTACT
                              (TATA+)                          (INR+)                                             
Human     CCCCTACCTCCCTGGACCTCCT  GAAAAAC  GCTGCCCGGGGAAAGTCCGG  G C AGAGCC  CGAGCAGCGGCCAGGGTAACGC  TGTCTT  GTGGACCCGCACTTC
                                 (TATA+)                       (INR+)                         (DPE-)                
    21 Mouse: C4bp (12269, M97289) 
Human: C4BPA (722, M62448) 
 
           |-50      |-40      |-30      |-20      |-10      |1        |11       |21       |31       |41       
Mouse     GAAGGAGATTAAATAAACACTGCTGCTCTAGTCTGTTAATCATTCACCGG G   ACAGAGGA  GACTGTCTCCCTGTCCGTCATACTCAACTGCTTAACCAGAA
                                                               (INR+)                                           
Human     GAGCTTAGGTAAACAGT  GCTGCTT  TATTTCTGCTGTTAATCAT  TCATTGG G   CCCGTCAAAAGTTTCTGC  CCATCTATTTCCA  TCAACCGTCCTTGACCAG
                            (TATA-)                      (INR-)                         (MTE+)                      
    22 Mouse: Cab39 (12283, NM_133781) 
Human: CAB39 (51719, NM_016289) 
 
           |-50      |-40      |-30      |-20      |-10      |1        |11       |21       |31       |41       
Mouse     GCCGGGCTCAGGCCGGAGCTGGGCGCGGGGCGCCAGGAGCCGCGCGGCCT G AG  AGAGTCTG  GGGCTTCA  GTAGCCGCGCGAA  GGCTGAGACAACCTCAGA
                                                                 (INR+)               (MTE+)                      
Human     CCGGGCTCGGGCCGCAAGCGGGGCGAGGGGTTCGGGGAGCGGCGC  GGCCT G GG  AGACACAGAGCCTTCAGGCGCCGGGGCGGGGGCACAGGCGAAGACTA
                                                         (INR-)                                                 
    23 Mouse: Cacna1e (12290, NM_009782) 
Human: CACNA1E (777, AF387615) 
 
           |-50      |-40      |-30      |-20      |-10      |1        |11       |21       |31       |41       
Mouse     TGAACTTTTTTTTCTTTTTG  CAGAAAC  CCTGACCTAAATGTCTGGACCCT   A ACCTGGT  TACTACACAG  ATGTGTGGATAAA  CCCAGCCTTGTGCTGCCCC
                               (TATA+)                          (INR-)                 (MTE-)                       
Human     TGAGGTTATTGAACTTTTCCA  CAGAAAC  CCTGACCTAAATTCTTGACC  TC A GGCTG  TTTACAACAC  AAATCCCCGCCTA  AATGAAGCCTCGTGGTGCCCC
                                (TATA+)                       (INR+)                 (MTE+)                         
    24 Mouse: Cacna1s (12292, AF343753) 
Human: CACNA1S (779, BF828872) 
 
           |-50      |-40      |-30      |-20      |-10      |1        |11       |21       |31       |41       
Mouse     GGCTAATTTTACTCGCTGGGAGCAGAGAGAGTAATCCTCCCGCCC  CCACG C CC  GACCCGTCTGCCTGCCTCAGCA  GGATAG  GCTCAGTCAGGTCCAGTCC
                                                         (INR+)                         (DPE+)                    
Human     GGCTAATTTTACTTGCTGGGAGCGAGGAGAGTAATCCTCCTGCCC  CCACT C CT  GCCCCCGCCCC  CTGGCTGGCTCAG  CAGGGCAGGCTCAGCCGACAGCC
                                                         (INR+)                  (MTE-)                           
    25 Mouse: Capn2 (12334, AK053832) 
Human: CAPN2 (824, BC007686) 
 
           |-50      |-40      |-30      |-20      |-10      |1        |11       |21       |31       |41       
Mouse     CGGCCCAGGCGCTTCCCGCCGGTGAATCATCCCCGCAGCAGCGGCTCCC  G C AGTCCG  CTGCAGCGCCCCGGGCCCGGCCGCGCCCCAGCCGAGTGCCGTG
                                                             (INR+)                                             
Human     GGCCGGGCCGCTTCCCTCCGGTGAATCATCGCTCGCAGCGGCGGCGCCC  G C AGTGGC  CGCAGCAGCGCGCCGGGCCCTGGCCGCGCCCCAGCCGAGCGCA
                                                             (INR+)                                             
    26 Mouse: Nr1i3 (12355, AF009328) 
Human: NR1I3 (9970, NM_005122) 
 
           |-50      |-40      |-30      |-20      |-10      |1        |11       |21       |31       |41       
Mouse     ACCCACACCCAGGTCTTTGCCCTGGGTCCAGAGTCTGGGTCCTACCTA  CA T ATGGC  ACCGAGGATACCTAGAGGCCCCATGCAAGAGAAGGCCCTTGTTT
                                                            (INR-)                                              
Human     CCTCCCACCCAGGCCTTTGCCCTGAGTCCAGGGTCTGTGCCCTAACCCC  A C AGTCAC  TGAGAGCAACTGGAGGCCACATAAAACAGACATCTCTTGTTTT
                                                             (INR+)                                             
    27 Mouse: Casq1 (12372, NM_009813) 
Human: CASQ1 (844, NM_001231) 
 
           |-50      |-40      |-30      |-20      |-10      |1        |11       |21       |31       |41       
Mouse     TCCCCGCCCCTGCTCCTATTCCTCCTCCTGACCCTTTTTCTCTTG  GCTCT G TC  GGCAGTTTCTCCAGGACCCAGCAG  TGTCCT  CTGTCCACTGCTCTGGC
                                                         (INR-)                           (DPE-)                  
Human     CCCCGCCCCCTGCTCCTATTCCTCCACCTGACCCTTTTTCCCTTG  GCTCT G TC  GGCAGTTTCTCCAGGACCCA  GCAGTGCCCTCTG  TCCACTGCTCTGGG
                                                         (INR-)                           (MTE+)                  
    28 Mouse: Rb1cc1 (12421, AB050017) 
Human: RB1CC1 (9821, D86958) 
 
           |-50      |-40      |-30      |-20      |-10      |1        |11       |21       |31       |41       
Mouse     CCCGACTCCCATCCTT  CCGGGCCT  CGCCGGGTACTCGGCGGCTGGGCGCC G ACGGTTGTGTCGGTTGGCGGCGCCGCAGGGGCGGTTGATAGCCGCCGCC
                            (BRE+)                                                                              
Human     TCCCTGCCTCCTAGAGTT  CGGGGCCG  CGGCGGGCGGGCGCCCGGGACGCC G   GCGGTTGT  GTCGGC  TTAGCGGTGCCGA  ATGGGCGGTTGGTAACCGCTGC
                              (BRE+)                             (INR+)             (MTE+)                          
    29 Mouse: Cd28 (12487, AK041322) 
Human: CD28 (940, M37812) 
 
           |-50      |-40      |-30      |-20      |-10      |1        |11       |21       |31       |41       
Mouse     CTTCAGTTCACACCACACTCTGCCTTGCTCACAGAGGAGGGGCTGCAGCC C TGGCCCTCATCAGAACAATGA  CACTCAGGCTGCT  GTTCTTGGCTCTCAA
                                                                                       (MTE-)                   
Human     TTTCAGTTCCCCTCACACTTCGGGTTCCTCGGGGAGGAGGGGCTGGAACC C TAGCCCATCGTCA  GGACAAAGATGCT  CAGGCTGCTCTTGGCTCTCAACT
                                                                               (MTE-)                           
    30 Mouse: Cd3z (12503, J04967) 
Human: CD3Z (919, AA355589) 
 
           |-50      |-40      |-30      |-20      |-10      |1        |11       |21       |31       |41       
Mouse     CGAGAGGGGGAGTGAGA  GGGTGTCT  TGCTGGCAGGTGCTGTCTCAAAGGC C C  ACAGTCCT  CCACTTCCTGGGGTGTCAGCCACAGAACAAAGCCAGCAGA
                             (BRE+)                               (INR+)                                          
Human     GGGGGAGTGC  GAATTTCT  TGGCCCTGTCGGCAGGTGCTTTCTCAAAGGCC C C  ACAGTCCT  CCACTTCCTGG  GGAGGTAGCTGCA  GAATAAAACCAGCAGA
                      (BRE+)                                      (INR+)                  (MTE-)                    
    31 Mouse: Cd3z (12503, L03353) 
Human: CD3Z (919, CR614814) 
 
           |-50      |-40      |-30      |-20      |-10      |1        |11       |21       |31       |41       
Mouse     CAGCCACAGAA  CAAAGCCA  GCAGAGACTCCATCAGCGCCTCCTTTTCTCC T CATCCTCCCAGGC  ATAGCTGCCTCTG  CCTCTGCCTCTGGGTACCATCCC
                       (BRE+)                                                    (MTE+)                           
Human     TTCCTGGGGAGGTAGCTGCAG  AATAAAA  CCAGCAGAGACTCCTTTTCTCC   T AACCGTC  CCGGCCACCGCTGCC  TCAGCCTCTGCCT  CCCAGCCTCTTTCT
                                (TATA+)                         (INR-)                      (MTE+)                  
    32 Mouse: Cfh (12628, J02891) 
Human: CFH (3075, NM_000186) 
 
           |-50      |-40      |-30      |-20      |-10      |1        |11       |21       |31       |41       
Mouse     GACAGGGCATAGTAACAACAAGA  GATAAAA  CGCCCATGCTGCTGGACTTG T   GGTCTACT  ATTTTAGTTTACTTTGCAGA  AGTTGC  TCATGGGCGGAGCAA
                                  (TATA+)                        (INR-)                       (DPE+)                
Human     AGTGGGAGTGCAGTGAGAA  TTGGGTT  TAACTTCTGGCATTTCTG  GGCTTG T G  GCTTGTGGTTGATTTT  TTATTTACTTTGC  AAAAGTTTCTGATAGGCGG
                              (TATA-)                     (INR-)                       (MTE+)                       
    33 Mouse: Il8rb (12765, D17630) 
Human: IL8RB (3579, L19593) 
 
           |-50      |-40      |-30      |-20      |-10      |1        |11       |21       |31       |41       
Mouse     GACATTTGGTCCAGAGGGCCTCCAGGACTTCCCCATCGGTAGCCCAGGTC A   GTAGTTTC  CTCATCACGG  CTGCCTCACTTTC  TTCCAGTTCAACCAGCCC
                                                               (INR+)                 (MTE-)                      
Human     GAATACCTCCCCAGGAG  GGCATCCT  GGATTTCCCCCTTGCAAC  CCAGGTC A   GAAGTTTCATCGTCAAGGTTGTTT  CATCTT  TTTTTTCCTGTCTAACAGC
                             (BRE-)                      (INR+)                           (DPE-)                    
    34 Mouse: Cxcr4 (12767, X99582) 
Human: CXCR4 (7852, CD695230) 
 
           |-50      |-40      |-30      |-20      |-10      |1        |11       |21       |31       |41       
Mouse     CCGCCCCGGGA  GCGTGTTT  T  TATAAAA  GTCCGGTTCGGGCCAGAAACT  TC A ATTTT  GTTGCCTGGT  GCAGCAGGTAGCA  GTGAAACCTCTGAGGCGTTTG
                       (BRE+)    (TATA+)                        (INR+)                 (MTE+)                         
Human     CCGCGCTCGG  AGCGTGTT  TT  TATAAAA  GTCCGGCCGCGGCCAGAAACT  TC A GTTTG  TTGGCTGCG  GCAGCAGGTAGCA  AAGTGACGCCGAGGGCCTGAGT
                      (BRE-)     (TATA+)                        (INR+)                (MTE+)                          
    35 Mouse: Cmkor1 (12778, AK031100) 
Human: CMKOR1 (57007, NM_020311) 
 
           |-50      |-40      |-30      |-20      |-10      |1        |11       |21       |31       |41       
Mouse     GGCACTTTCCCGCAGTGTA  TATAAAA  GGCAAGTCCGGAGCCCTGAGAGAT C   TCAGTTGC  TACAAACT  GCTCAGCACTGAA  GGAGCCTGCAGCGCTCACCG
                              (TATA+)                            (INR+)               (MTE-)                        
Human     GAGGCTCCTTTCTGCAGTG  TATATAA  TGCAAGTCTGCAGCCAGCAGAGCT C   ACAGTTGT  TGCAAAGT  GCTCAGCACTAAG  GGAGCCAGCGCACAGCACAG
                              (TATA+)                            (INR+)               (MTE-)                        
    36 Mouse: Col19a1 (12823, NM_007733) 
Human: COL19A1 (1310, AW139403) 
 
           |-50      |-40      |-30      |-20      |-10      |1        |11       |21       |31       |41       
Mouse     CACACTGAAGGGACG  AATAAGG  GCAGAGATGCGTCCCGCTTCCACTCGCT G G  GAGCTGGA  GCGGGCCACT  CCGCGGCGGTGCG  GCAGCCCTGTCCGGACT
                          (TATA+)                                 (INR-)                 (MTE-)                     
Human     ACTGGGAGAGACTAATAAGGGCAGAGATGCGTCCCCCTTCCCCACTCGCA G GGAGCTCACTCCTCGGCGGTGCC  GCAGCCCTGTCCG  GACTCCACTGCGC
                                                                                         (MTE+)                 
    37 Mouse: Col3a1 (12825, X52046) 
Human: COL3A1 (1281, NM_000090) 
 
           |-50      |-40      |-30      |-20      |-10      |1        |11       |21       |31       |41       
Mouse     GGGAAGCCAAACTTTTTCCTA  TTTAAGG  CCAGAGCAGAGGGAAGCGAGCG G C  TGAGTTTT  ATGACGG  GCCCGGTGCTGAA  GGGCAGGGACAACTGATGGT
                                (TATA+)                           (INR+)              (MTE-)                        
Human     GGGAAGCCAAACTTTTTCCTA  TTTAAGG  CCAAAGCAAAGGAATC  TCAGTG G C  TGAGTTTTATGACGG  GCCCGGTGCTGAA  GGGCAGGGAACAACTTGATG
                                (TATA+)                   (INR+)                      (MTE-)                        
    38 Mouse: Col4a3 (12828, AK086939) 
Human: COL4A3 (1285, NM_000091) 
 
           |-50      |-40      |-30      |-20      |-10      |1        |11       |21       |31       |41       
Mouse     GCTGGCCACTCCCCCCACCCTGTGCAGCCACCTCCCTACCGCACACCC  CT A AATTC  CTCACGTTGACC  TGCACCCGCTAGG  CACCGAAGTCCCAGGATCT
                                                            (INR+)                   (MTE-)                       
Human     CTGCGCAGCCACCTCCCCACCGCGCAGCCACCTCCCCACCGCACACCC  CC A AACGC  CCCACCTCCGACC  GCACCCCACTTCC  CCGCCTGGGCCCCCGGAC
                                                            (INR+)                    (MTE-)                      
    39 Mouse: Col4a4 (12829, AF169388) 
Human: COL4A4 (1286, AF218541) 
 
           |-50      |-40      |-30      |-20      |-10      |1        |11       |21       |31       |41       
Mouse     CGGGAGTCTGCAACTCCGGAGGCCAAGTTTGCTGGCAGCTCTAG  GGACTG C C  CATCGGCTGCCGGCCT  TCAACTCGCAGCC  AGCACGCAGACCTGAGGCT
                                                        (INR-)                       (MTE+)                       
Human     CCGGGCGTCTGCACTCGGGAGGCCCCGCTTGTTCCCCGCGCCCGCG  GCGG T CCG  CGTTCCCCGCCT  CCAGCCCGCCTC  C  AACTT  GCGGCCGCCGGGCTTG
                                                          (INR+)                   (MTE+)    (DPE-)                 
    40 Mouse: Col5a2 (12832, AK080811) 
Human: COL5A2 (1290, M31365) 
 
           |-50      |-40      |-30      |-20      |-10      |1        |11       |21       |31       |41       
Mouse     GGCAGCATTCTTCCTA  TTTAAAG  CTGCACCGCTTGAAAAAAGTTTTCGC  A G ACTGTG  CCGGACCTGGTGCT  GAAACAGACTGAG  GCAGCGCGGGGACTGG
                           (TATA+)                             (INR-)                     (MTE-)                    
Human     GGCAGCATTCTTCCTA  TTTAAAG  CTGCATCGCTTGAAAAAAGTTTTCGC  A G ACTGTG  CTGGAGCTGGTGCTGAA  AAAGGGGGTTTGC  AGAGGCTGCCCTG
                           (TATA+)                             (INR-)                        (MTE-)                 
    41 Mouse: Col9a1 (12839, NM_007740) 
Human: COL9A1 (1297, AF036110) 
 
           |-50      |-40      |-30      |-20      |-10      |1        |11       |21       |31       |41       
Mouse     CTTTCCTTTGCTTCAG  CTTAAAG  GGTTATTGCATGCCTCCAAT  TCACTCT T   TTTGCCTTTAGCCCTGAGAGGGGCAAGAAGGAGCAAGCTTGGGGCCAAA
                           (TATA+)                       (INR+)                                                   
Human     CTTTCCTTTGCTTCAG  TTTAAAG  TGTCACGAGATGCCTCTGGTTCTCTC  C C TTTGCT  TTTAGCCCTCACCGGGGGCAGGAG  GGACCA  AGGCTGGGCCCAG
                           (TATA+)                             (INR-)                           (DPE-)              
    42 Mouse: Copa (12847, AK043479) 
Human: COPA (1314, NM_004371) 
 
           |-50      |-40      |-30      |-20      |-10      |1        |11       |21       |31       |41       
Mouse     CATCGTCTCTGTGGCCCA  GCGCTCCA  GAGACCGGAAGTCCTTGTCCGAGG A GCTTCCGGGAGCCGCGACCC  GGGAGTAGCTGAC  GTGGAAGCCTTGGGAG
                              (BRE+)                                                    (MTE-)                    
Human     ACCGTCTCTGTGGCC  CGGAGCCT  AAGAGACCGGAAGTTCGTGTTT  CCAGG C GC  TTCCGGAAACCGCGGGAGAG  GGTCGC  TGACGTGGAGGCGTCCGAAGG
                           (BRE+)                          (INR+)                       (DPE+)                      
    43 Mouse: Cox5b (12859, M77040) 
Human: COX5B (1329, AA393683) 
 
           |-50      |-40      |-30      |-20      |-10      |1        |11       |21       |31       |41       
Mouse     CGGAAGACTGACCG  GGCGTTGT  TAGACTCCCACCAACGGAAGTCCCGCC  C A TCTTGC  TCAGCCT  GTTCCCGGAAGTG    CATCTG  CTTGTCTCGGGCGAGAT
                          (BRE-)                               (INR-)              (MTE-)     (DPE-)                  
Human     CCGAAAGCTGACCGAGA  GGAGAAAG  AAGCCCGCCCCCGGAAGTCCCTCCT G   TCTCTGCA  GCTTGTTCCCG  GAAGTTTTGCTGC  TAGTCGCGGACGCAATG
                             (BRE-)                              (INR-)                  (MTE+)                     
    44 Mouse: Cr2 (12902, U17123) 
Human: CR2 (1380, M56003) 
 
           |-50      |-40      |-30      |-20      |-10      |1        |11       |21       |31       |41       
Mouse     AAACTTTAGCTTTTCT  CAGCTTT  GATATTTCTGTGGTCCTTATTTCTAGG   T CAGTGTA  AGTTGCTGCCAAA  GTATTCTTTTGCT  AAACCTTCTATTGTGA
                           (TATA-)                              (INR+)                    (MTE+)                    
Human     AGCCTCTCTTGGTTT  TTATGTTT  CTGTAGCCTTTGTCTCAGGTCC  TCAGG T CA  GGGTAAGTTTC  TGCCAAGGTTTCC  TTTTGCTAAGCCTAAAATTCAGA
                           (BRE+)                          (INR+)                  (MTE-)                           
    45 Mouse: Creb1 (12912, X92497) 
Human: CREB1 (1385, BX116954) 
 
           |-50      |-40      |-30      |-20      |-10      |1        |11       |21       |31       |41       
Mouse     CGTGAGTGGCCGCTGCGCACTCGGCACTGGGCGGCGCTGGCTGGCTCCCT G GCTGCGGCTCCTCAGTCGGCGGCGGCTGCTGCTGCCTGTGGCCCGGGCG
                                                                                                              
Human     CGTGCGCGGCCGCTGCGCACTCGGCACTGGGCGGCGCTGGCTGGCTCCCT G GCTGCGGCTCCTCAGTCGGCGGCGGCTGCTGCTGCCTGTGGCCCGGGCG
                                                                                                              
    46 Mouse: Crp (12944, X13588) 
Human: CRP (1401, AF449713) 
 
           |-50      |-40      |-30      |-20      |-10      |1        |11       |21       |31       |41       
Mouse     AGCCCCTCCATCTGCTATAGT  TATAAAT  CTGAGGATGGGCTGGGCCCGAG G CAAGCGTTCCAGGACTCCTTGTCCTT  GATCTT  TCAGACAAAACACTGTC
                                (TATA+)                                                   (DPE-)                  
Human     CTCCCTTACTGCTTTGGA  TATAAAT  CCAGGCAGGAGGAGGTAGCTCTAAG G CA  AGAGATCT  AGGACTTCTAGCCCCT  GAACTT  TCAGCCGAATACATCTT
                             (TATA+)                               (INR+)                   (DPE-)                  
    47 Mouse: Cryba2 (12958, AJ272227) 
Human: CRYBA2 (1412, NM_057093) 
 
           |-50      |-40      |-30      |-20      |-10      |1        |11       |21       |31       |41       
Mouse     GGAGAGGCAGCCA  GACATGAC    TATAAAG  CAGCGAACACCAGGCTCGCGC  G C AGCCTC  TCCATTTTCCGTCG  GTGCGGTGCTCAC  TCACTCGGTGAGTACT
                         (BRE-)   (TATA+)                        (INR+)                     (MTE-)                    
Human     GGACAGGGGGACAA  GGCCCAGG    TATAAAG  CTCGGCCCGCCCGGCCGCGC  G C AGCCTC  GCAGCCGGT  CCATTTCCCTCGC  GTGCCGCGCTCACCCACCCGG
                          (BRE-)   (TATA+)                       (INR+)                (MTE+)                         
    48 Mouse: Crygb (12965, M19359) 
Human: CRYGB (1419, M11970) 
 
           |-50      |-40      |-30      |-20      |-10      |1        |11       |21       |31       |41       
Mouse     GCAGCAGTCATGACAGC  TATATAT  ACCAGGGGAGCTCCCCTAG  AGTCTCA C   AGCTCCCAGGGCATCTCTTACTC  TCAGCGAGATGGG  AAAGGTAAGTCCT
                            (TATA+)                      (INR-)                              (MTE-)                 
Human     GGCAGCAGTCAGGGCTGC  TATACAT  ACAGTGACGTTCCCGCAGTC  CCACA C AG  CAACCAGAAAACA  TCTGCTCACTTCC  TTCAAAATGGGAAAGGTAAGT
                             (TATA+)                       (INR+)                    (MTE-)                         
    49 Mouse: Crygc (12966, NM_007775) 
Human: CRYGC (1420, M11972) 
 
           |-50      |-40      |-30      |-20      |-10      |1        |11       |21       |31       |41       
Mouse     TCCTTGCTGGCAGA  AATCACT  TATTTGTCTGGTCCCTTTCTGCGCTT  ACA G ATCA  CCTTCTTCGAG  GACCGCAGCTTCC  AGGGCCGCTGCTATGAGTGCA
                         (TATA-)                             (INR+)                  (MTE-)                         
Human     GGCCTCAGTGGGGGCCAA  TCACTCCA  TGCTCCCACATCTTCCATTTT  TCA G ATCA  CCTTCTACGAGGACAGGG  CCTTCCAGGGCCG  CAGCTACGAATGCA
                              (BRE+)                         (INR+)                         (MTE+)                  
    50 Mouse: Crygd (12967, BC057013) 
Human: CRYGD (1421, NM_006891) 
 
           |-50      |-40      |-30      |-20      |-10      |1        |11       |21       |31       |41       
Mouse     ACGCAGCAGACCTCCTGC  TATATAG  ACCCTGCTCCCAGCCCCACACACTC A ACAGCACCATCCCATCCGACCTGCCAACACCAGCCATGGGGAAGGTGAG
                             (TATA+)                                                                            
Human     GCAGCAGCCCTCCTGC  TATATAG  CCCGCCGCGCCGCAGCCCCACCCGCTC A GCGCCGCCGCCCCA  CCAGCTCAGCACC  GCCGTGCGCCCAGCCAGCCATG
                           (TATA+)                                                (MTE+)                          
    51 Mouse: Crygd (12967, M19359) 
Human: CRYGD (1421, NM_006891) 
 
           |-50      |-40      |-30      |-20      |-10      |1        |11       |21       |31       |41       
Mouse     AACGCAGCAACCCTCCTGC  TATATAG  ACCAGCCACTCTCTGGCTGG  ACAC T GAA  CTCACCACGGGTCAG  CCAGCCATGGGGA  AGGTGAGCAGAACGCAAA
                              (TATA+)                       (INR-)                      (MTE+)                      
Human     CAGCCATCCTGCTA  TATAGAC  TGGCTGTGCAGCCGCAGGCCCCA  TCACAC T G  AACTCGCATCATCCGTGTCAA  CCAGCCATGGGGA  AGGTGAGCAGAACA
                         (TATA+)                          (INR+)                            (MTE+)                  
    52 Mouse: Csrp1 (13007, AK087436) 
Human: CSRP1 (1465, M33146) 
 
           |-50      |-40      |-30      |-20      |-10      |1        |11       |21       |31       |41       
Mouse     GAAACGCCTTCCGGAGGGGGA  AACAAAA  CGGCGCGCACTCCGGCGCAG  CC A GTCAC  CGCTTCCCTGAGAGCTGTCGCCCGTGCGCCTCCCAGCAGCTGCC
                                (TATA+)                       (INR+)                                              
Human     ACACGCCTTCCGC  GGAGCGG  A  ACAAAA  CGGCGCGCAGGCCGGGCGCAC  CC A GCCGC  CACTTCCGAGAGCGCCTGCCGCCCCTGCGCCGCCGAGCCAGCTG
                         (BRE-)  (TATA+)                        (INR+)                                              
    53 Mouse: Daf1 (13136, AK030285) 
Human: DAF (1604, BX376935) 
 
           |-50      |-40      |-30      |-20      |-10      |1        |11       |21       |31       |41       
Mouse     TGCCCCACCCAGGGTGACGAGGGCCCTGCCCCGCCCCGCCACAGCTGCTC A AT  TAACTGCG  GCT  CAAAACAGCTCCA  GCCGCAGCCGGGCAAGGTCTCTT
                                                                 (INR-)          (MTE-)                           
Human     CCCACCCTTGGT  GACGCAGA  GCCCCAGCCCAGACCCCGCCCAAAGCACTC A TT  TAACTGGT  ATTGC  GGAGCCACGAGGC  TTCTGCTTACTGCAACTCGCT
                        (BRE-)                                     (INR-)            (MTE+)                         
    54 Mouse: Dbi (13167, AK078428) 
Human: DBI (1622, BE407606) 
 
           |-50      |-40      |-30      |-20      |-10      |1        |11       |21       |31       |41       
Mouse     AGTTGGAACGCCGGGGTT  GTGCTTT  TAAAGGCGCTAGCTGGTGC  GCTCTG T G  ACTTGATTGCTGCTG  CTTCTGAGCTTGC  TCCCGCGCTTTCGGCATCCG
                             (TATA-)                      (INR-)                      (MTE-)                        
Human     GGTTGGGGCGAGTGGA  CCGCGCC  T  CTAAAG  GCGCTTGCCAGTGC  AATCTG G G  CGATCGCTTCCTGG  TCCTCGCCTCCTC  CGC  TGTCTC  CCTGGAGTTCTT
                            (BRE+)  (TATA+)                 (INR-)                     (MTE+)        (DPE-)             
    55 Mouse: Dbi (13167, AK078428) 
Human: DBI (1622, BC062996) 
 
           |-50      |-40      |-30      |-20      |-10      |1        |11       |21       |31       |41       
Mouse     GGACACGCCCCCGAAAGCAGATCCGACTTCTGATTGGCTGCTGCCGCTCG C CCGAGCTCAGTTGGAACGCCGG  GGTTGT  GCTTTTAAAGGCGCTAGCTGG
                                                                                    (DPE+)                      
Human     GGGCACGCCCCTAGCGCATAGCTGGCTTCTGATTGGCTTTCCGGTGCTCG C CCGAGCAGGGTTGGGGCGAGT  GGACCG  CGCCTCTAAAGGCGCTTGCCAG
                                                                                   (DPE-)                       
    56 Mouse: Degs1 (13244, BC003751) 
Human: DEGS1 (8560, BG720437) 
 
           |-50      |-40      |-30      |-20      |-10      |1        |11       |21       |31       |41       
Mouse     GCCCGCGTCCCCGCGGCTTATCGACTAGAGCCGGGCCTCGAGCTGCCGC  C G TCCGGA  CACGAGCCGGAGGCAGAGCG  GGTCCA  CAGGCCGCAGCCATGGG
                                                             (INR-)                       (DPE-)                  
Human     CGCGCTCCGCCACAGCCGGCCGACACCACACCAGCCGGGGAGCCGCCGCC G CCGCCGCCACCTCTGA  GCAGCCGGCTGGG  AGCGAGAGCCGACAGCTAGT
                                                                                  (MTE-)                        
    57 Mouse: Des (13346, L22550) 
Human: DES (1674, AK097038) 
 
           |-50      |-40      |-30      |-20      |-10      |1        |11       |21       |31       |41       
Mouse     ATGTCAGGAGGGCTACAA  ATAGTGCA  GACAGCTAAGGGGCTCCGTCACCC A TCTTCACATCCACT  CCAGCCGGCTGCC  CGCCCGCTGCCTCCTCTGTGCG
                              (BRE-)                                              (MTE+)                          
Human     GATGTCAGGAGGGA  TACAAAT  AGTGCCGACGGCTGGGGGCCCTGTCTCCC C TCGCCGCATCCACTCTCCGGCCGGCCGCCTGCCCGCCGCCTCCTCCGTG
                         (TATA+)                                                                                
    58 Mouse: Dfy (13349, AF016697) 
Human: FY (2532, Y14873) 
 
           |-50      |-40      |-30      |-20      |-10      |1        |11       |21       |31       |41       
Mouse     ATTAGCCCT  GGGCACTT  ATCTTGGAGCCACAGCTGCTGACAGAGT  CCAGG C CC  TGTACTTCTCTGCCC  TGAGCCTGCAGTG  CCATGGGGAACTGTCTGTA
                     (BRE+)                                (INR+)                      (MTE+)                       
Human     TTAGTCCTT  GGCTCTTA  TCTTGGAAGCACAGGCGCTGACAGCCGTCCCAG C C  CTTCTGTC  TGCGGGCCTGAAC  CAAACGGTGCCAT  GGGGAACTGTCTGC
                     (BRE-)                                       (INR-)                    (MTE+)                  
    59 Mouse: Dfy (13349, AK010883) 
Human: FY (2532, NM_002036) 
 
           |-50      |-40      |-30      |-20      |-10      |1        |11       |21       |31       |41       
Mouse     TGTGACTCTGAATATATA  TATATAT  ACACACACACACCCTGGCTC  TGCCT A GA  ACCCGAGAGAAGTCA  TTAGCCCTGGGCA  CTTATCTTGGAGCCACAGC
                             (TATA+)                       (INR-)                      (MTE+)                       
Human     GCAGGCAGTG  GGCGTGGG  GTAAGGCTTCCTGATGCCCCCTGTCCCTGCCC A G  AACCTGAT  GGCCCTCATTA  GTCCTTGGCTCTT  ATCTTGGAAGCACAGG
                      (BRE-)                                      (INR-)                  (MTE-)                    
    60 Mouse: Dnpep (13437, AK052001) 
Human: DNPEP (23549, AK001777) 
 
           |-50      |-40      |-30      |-20      |-10      |1        |11       |21       |31       |41       
Mouse     TTCCGATCCT  GTACTCCA  GG  GACAAAA  CTGGGTGGAGGTCCTCTTGCCCT G   GGACTGAA  CCTTTGAGC  CGAGAGCGATCGA  GCTAGGAATCCACATCTGT
                      (BRE+)     (TATA+)                           (INR-)                (MTE-)                       
Human     CTCATGATCCTGGGCCGGCGGGCT  GAAGCTT  TCAACGGAGTTTCAGCCCT G   GGTTTGGG  TCTCTCGGTGGGG  ACGGGCGGAAGCA  GAGGTCTGTGCCTTA
                                   (TATA-)                       (INR-)                    (MTE-)                   
    61 Mouse: Ecel1 (13599, NM_021306) 
Human: ECEL1 (9427, AJ130734) 
 
           |-50      |-40      |-30      |-20      |-10      |1        |11       |21       |31       |41       
Mouse     GCCGGCGCGCCCCCTCCTTCGGGGCTGGCGGGCGGCGCGGCGCGGCG  GCA G AGGC  GGCGGGCGCACGAAGAGAGCCGCAGCCAGCCCAGCTCCTCTCCGG
                                                           (INR+)                                               
Human     CCTCCCTCGGGCCGGCAGGCGGGCGGCGCGGCGGGCTCGGCGCGGCG  GCA G AGGA  GGCGGCGGGCGCTGGGAGACACC  GGACGC  CCGCTCGGCTGCGCTG
                                                           (INR+)                          (DPE+)                 
    62 Mouse: Elf3 (13710, AY456682) 
Human: ELF3 (1999, AF517841) 
 
           |-50      |-40      |-30      |-20      |-10      |1        |11       |21       |31       |41       
Mouse     AGCTCCTGCTTTGCTC  TATTTAG  AGCGGGTGGGGGCAGCGCCCTGG  CCAC A CTC  ATCACTGCTACCTGCGGAGCCTT  CGACCG  CTTAGATTTCTTCCCTT
                           (TATA+)                          (INR+)                          (DPE-)                  
Human     AGCTCCCTCCA  GGCTCTA  T  TTAGAG  CCGGGTAGGGGAGCGCAGCGG  CCAG A TAC  CTCAGCGCTACCTGGC  GGAACTGGATTTC  TCTCCCGCCTGCCGGCC
                       (BRE-)  (TATA+)                        (INR+)                       (MTE-)                     
    63 Mouse: Elk4 (13714, NM_007923) 
Human: ELK4 (2005, NM_021795) 
 
           |-50      |-40      |-30      |-20      |-10      |1        |11       |21       |31       |41       
Mouse     GTGACGCTCGCGCTGCTT  CGGCGTCG  AGGAAGGGCCCCGGCAGTGCCGGG G GGGGGGGGTTTGAGAGTGGCGGA  GGCCGCGGAACGA  CGGGAGACGGCGG
                              (BRE+)                                                       (MTE-)                 
Human     AGTGACGTGTAGCGACTA  CGGCGTCT  GGGAGGGACCCAGGAGCAGTCGGG G   GGTTTGAG  AGTGGCGGCGGCCGCGGAGGGCCTGGCAGGCCCCGCCGCTG
                              (BRE+)                             (INR-)                                           
    64 Mouse: En1 (13798, NM_010133) 
Human: EN1 (2019, AA853350) 
 
           |-50      |-40      |-30      |-20      |-10      |1        |11       |21       |31       |41       
Mouse     CTAGACTTTAAAGATTATTTTT  TTCCCTT  TAAGGAAAAAGTCTCGGA  GCT T TAAA  AAAAATTCCT  TTTTCTCTTTTTT  TTTCTCCCCTCTTTTTTTTTTT
                                 (TATA-)                     (INR-)                 (MTE+)                          
Human     TAGACTTTAAATACTATTTTT  TTCCCTT  TAAGAAAAAAATTATTGGAGCT T TTTTTCTTGCTTTCTTTTT  CCTTTTCTTTTTC  TTTTTTTCCTTCATTTT
                                (TATA-)                                                (MTE+)                     
    65 Mouse: En1 (13798, NM_010133) 
Human: EN1 (2019, AA853350) 
 
           |-50      |-40      |-30      |-20      |-10      |1        |11       |21       |31       |41       
Mouse     AAATTCCTTTTTCTCT  TTTTTTT  TCTCCCCTCTTTTTTTTTTTTTCTGAG C CGTGGCTTATCCCCCCAT  TAAGACCAATCAC  TGAAATCTTGTTGCTGAA
                           (TATA-)                                                    (MTE-)                      
Human     TCTTGCTTTCTTTTTCCTT  TTCTTTT  TCTTTTTTTCCTTCATT  TTTTTGG C   CGTGGCTTACTCCCCATT  TAAATCAAATCAT  TGAATCTGGTTGCAGAAA
                              (TATA-)                    (INR-)                         (MTE-)                      
    66 Mouse: Epha4 (13838, S57168) 
Human: EPHA4 (2043, NM_004438) 
 
           |-50      |-40      |-30      |-20      |-10      |1        |11       |21       |31       |41       
Mouse     GGTCCCACCCTCTTGG  CAATGTCT  TCAGACTGCTGCACTCCTAACTTAA  G C ATTTAT  TCCACTGGGATA  GAAGCGGCAGGAG  CAGCGTTGGCACCGGCGA
                            (BRE+)                             (INR+)                   (MTE+)                      
Human     GGGCCCGCCCCCTTGG  CAATGTCT  TCAGCCCGCTGCTCTCCTAACTTAA  G C ATTTAT  TCCACTGGGATA  GAAGCGGCAGGAG  CAGCGTTGGCACCGGCGA
                            (BRE+)                             (INR+)                   (MTE+)                      
    67 Mouse: F13b (14060, D10071) 
Human: F13B (2165, NM_001994) 
 
           |-50      |-40      |-30      |-20      |-10      |1        |11       |21       |31       |41       
Mouse     TTGTTTTGTCAAGA  GAAGTATT    GAGTATT  TAGTAATGAGATCCTGGGTGC T   TTAGAGCT  GTAAAAATCTTCATG  AGACAC  ACTGATGATGACGCTGAGAC
                          (BRE-)   (TATA-)                         (INR+)                  (DPE+)                     
Human     ACTTTTTCCTCAAGA  GAAGTATT  G  TGTACTT  AATGACAAGTTCCTAGTGC T TA  GAATTGTT  AAAATCTTTGT  GAAGCACACCACT  GAAGATGAGGTTGAA
                           (BRE-)    (TATA-)                         (INR-)                  (MTE+)                   
    68 Mouse: Fasl (14103, AK040139) 
Human: FASLG (356, D38122) 
 
           |-50      |-40      |-30      |-20      |-10      |1        |11       |21       |31       |41       
Mouse     TCGTCAGAAATTTCTGGGCGGAAACTTCCTGGGGTTGCTGTGAGC  TTTTT G AG  GCTTCTCAGCTTCAGATG  CAAGTGAGTGGGT  GTCTCACAGAGAAGCA
                                                         (INR-)                         (MTE+)                    
Human     TATCAGAAAATTGTGGGCGGAAACTTCCAGGGGTTTGCTCTGAGCTTCT  T G AGGCTT  CTCAGCT  TCAGCTGCAAAGT  GAGTGGGTGTTTCTTTGAGAAGC
                                                             (INR+)              (MTE+)                           
    69 Mouse: Fcer1a (14125, J05018) 
Human: FCER1A (2205, L14075) 
 
           |-50      |-40      |-30      |-20      |-10      |1        |11       |21       |31       |41       
Mouse     ACCAGATACAACACAGAAGACATTTCCTTCTCCTTTGTGGTTTCAAGC  CT A TTTTC  GAAGCCATAGCTCTCTGGTGC  AGTTAG  CACCTGAAGGTGCAGGG
                                                            (INR+)                        (DPE+)                  
Human     ACCAGATATGATACAGAAAACATTTCCTTCTGCTTTTTGGTTTTAAGCCT A   TATTTGAA  GCCTT  AGATCTCTCCAGC  ACAGTAAGCACCAGGAGTCCATG
                                                               (INR-)            (MTE+)                           
    70 Mouse: Fcer1a (14125, J05018) 
Human: FCER1A (2205, L14075) 
 
           |-50      |-40      |-30      |-20      |-10      |1        |11       |21       |31       |41       
Mouse     AGAAAATGGGTGAC  TATAAAA  TTGCCCAATTTGGCACAACCCTATTATTA   T CTCTGGA  GAAAGC  CAAGCCCAGGCAT  AGCTGATGAGTTAACCAGATACA
                         (TATA+)                                (INR-)             (MTE+)                           
Human     GTAAGTGGGTAAAT  ATTAAAT  TGCCCAGTTGGGCACCATCCTGAATATTA   T CTCTAAA  GAAAGAAGCAAAACC  AGGCACAGCTGAT  GGGTTAACCAGATA
                         (TATA+)                                (INR-)                      (MTE-)                  
    71 Mouse: Fcer1g (14127, BC034163) 
Human: FCER1G (2207, CV328518) 
 
           |-50      |-40      |-30      |-20      |-10      |1        |11       |21       |31       |41       
Mouse     GCAGGAAGGAGGGAACTGTGGTCAGGGAACTGTTCGTGGGCACAGCTGC  G C AGTTCT  GTCAGCGCAGCGCG  ATCACCAGCTCCC  AGCGCCGCAGCCCCCA
                                                             (INR+)                     (MTE-)                    
Human     GGAAGAGGGGGACTCTGTGGTCAGGGAACTGCTCGCTGAGCACAGCTGC  A C AGTGCT  GTCAGAACGGC  CGATCTCCAGCCC  A  AGATGA  TTCCAGCAGTGG
                                                             (INR+)                  (MTE+)      (DPE+)             
    72 Mouse: Fcgr2b (14130, AK089266) 
Human: FCGR2B (2213, CR592864) 
 
           |-50      |-40      |-30      |-20      |-10      |1        |11       |21       |31       |41       
Mouse     TCCCCATTT  GGACTTC  A  CAATTT  TGTCTGTTCTCTGGGTACTATCTG  CCA A GCCG  GGAGGGAAGCCTGTGCCTGCAGC  TGACTC  GCTCCAGAGCTGATGG
                     (BRE+)  (TATA-)                           (INR+)                          (DPE-)                 
Human     TCCCCTTTC  AGACTCCA  G  AATTTGT  TTGCCCTCTAGGGTAGAATCCGCCA A   GCTTTGAG  AGAAGGCTGTGA  CTGCTGTGCTCTG  GGCGCCAGCTCGCTCC
                     (BRE+)    (TATA+)                             (INR-)                   (MTE-)                    
    73 Mouse: Fcgr2b (14130, M63159) 
Human: FCGR2B (2213, CR592864) 
 
           |-50      |-40      |-30      |-20      |-10      |1        |11       |21       |31       |41       
Mouse     CCATAGCAGTGAGAAGAGTAAGTAGTGCAGTGGGGCGGGGGAGGGATTCA   G GACTAGG  GCACAGACC  GATGCTCGATAGG  TACTTACAGAGGAGCGAGTG
                                                              (INR-)                (MTE-)                        
Human     GCAGTGAGAACAGGGATGGAAATGAGGGTGGCAAAAATGACCAAGAT  ACA A AACC  AGGGCACAGATTGGTGCTCAAT  AGATAC  TTATTGGGATATTCATT
                                                           (INR+)                         (DPE+)                  
    74 Mouse: Fmo3 (14262, AK050123) 
Human: FMO3 (2328, NM_001002294) 
 
           |-50      |-40      |-30      |-20      |-10      |1        |11       |21       |31       |41       
Mouse     TGACTCTCCTGCACACACAAACA  TATAAGG  TGCACAACATTTTCCTTT  TC A GAGTC  CCCAGAAG  ACTAGGGGTTTAA  ACACAGAGAAGAAAGGAAGACAA
                                  (TATA+)                     (INR+)               (MTE-)                           
Human     TTTTCAACAATCTTCCT  AATATGT  AGGCTCACTAGAACATTTTCTCTTTC   A AACTGCC  CAGACGGTTGGACAGGACGT  AGACAC  ACAGAAGAAAAGAAGA
                            (TATA+)                             (INR-)                       (DPE+)                 
    75 Mouse: Fmod (14264, AK054183) 
Human: FMOD (2331, BC035281) 
 
           |-50      |-40      |-30      |-20      |-10      |1        |11       |21       |31       |41       
Mouse     AGACTTGCACACTCTCCGTAGGCGCTCACTCCTCTCTTCCCTCTCTG  CCA C ATTC  TCCAACCCAAGGAGACC  AGACAG  AAGGACGTGGTCACTCTGAACA
                                                           (INR+)                    (DPE+)                       
Human     CGCACACTCTCAGT  AGACTCTT  TCACTCCTCTCTCTCTTCCTCTC  TCACA C GT  TCTCCAACCCAAGGAGGCC  AGACAG  AGGGACGTGGTCACTCTCTGAA
                          (BRE+)                           (INR+)                      (DPE+)                       
    76 Mouse: Fn1 (14268, AK054456) 
Human: FN1 (2335, AJ535086) 
 
           |-50      |-40      |-30      |-20      |-10      |1        |11       |21       |31       |41       
Mouse     GGGCGGGAAGGTACTGTCCCA  TATAAGC  CTCTGCTCTTGGGGCTCAACCG C TCGCACCCGCTGCGCTGCACAGGGGGAGAAAAGGAGCCCAGGGTGTGAG
                                (TATA+)                                                                         
Human     GGGCGGGAGGGGACCGTCCCA  TATAAGC  CCCGGCTCCCGGCGC  TCGGACG C   CCGCGCCGGCTGTGCTGCACAGGGGGAGGAGAGGGAACCCCAGGCGCGA
                                (TATA+)                  (INR+)                                                   
    77 Mouse: G0s2 (14373, AK003165) 
Human: G0S2 (50486, M69199) 
 
           |-50      |-40      |-30      |-20      |-10      |1        |11       |21       |31       |41       
Mouse     GGAGGCGGATCCCGGACAAAAGA  TATAAGG  ACTCTGTTCACAGAGCGCTG C AGCTGCGGAGTGTCCCTCCCAAAGAGCAGCAGCTGAGGGAAGAAGAACG
                                  (TATA+)                                                                       
Human     GGCGTGTCTCAGAGAAAAGA  TATAAGC  GGCCCCCGGACGCTAAAGCGGTG C CAGCGGCGGAGTCTCCAAC  TGGGAGAGCTGCA  GCTGCCGAGAGGAGGAG
                               (TATA+)                                                 (MTE-)                     
    78 Mouse: Gbx2 (14472, U74300) 
Human: GBX2 (2637, BG827869) 
 
           |-50      |-40      |-30      |-20      |-10      |1        |11       |21       |31       |41       
Mouse     AGCTGGCAA  AATGTGAA  T  GAGAAAG  AGGAGCGCGATTTAAAGGTGCTGGC G GCGCCCGCCAGAGACAAG  TGCGCCGGCTTCG  CGCGCTCCCCAGCCGCCC
                     (BRE-)    (TATA+)                                                  (MTE-)                      
Human     GGCAGGCAA  AATGTGAA  T  GAGAAAG  AGGAGCGCGATTTAAAGGTGCTGGC T GCGCCCGCCGGAGATAAG  TACGCCGGCTTCG  CGCGCTCCCCAGCGGCCC
                     (BRE-)    (TATA+)                                                  (MTE-)                      
    79 Mouse: Glul (14645, AY044241) 
Human: GLUL (2752, AI064764) 
 
           |-50      |-40      |-30      |-20      |-10      |1        |11       |21       |31       |41       
Mouse     GGCGGGCACAAGCTGTC  AATAAAA  AGTACCGAGCAGCCAGCGACCCT  GCA G AGCG  GAGAATGGGAGTAGAGCAGAG  TGTCTG  AACAGCACGCTCACCCAT
                            (TATA+)                          (INR+)                        (DPE-)                   
Human     GGCGGCCCCGGGCCGCG  GATAAAG  GGTGCGGGGCTGCTGGCGGCTCT  GCA G AGTC  GAGAGTGGGAGA  AGAGCGGAGCGTG  TGAGCAGTACTGCGGCCTCC
                            (TATA+)                          (INR+)                   (MTE+)                        
    80 Mouse: Gls (14660, AF302091) 
Human: GLS (2744, AF327434) 
 
           |-50      |-40      |-30      |-20      |-10      |1        |11       |21       |31       |41       
Mouse     CTCTTTCCAGTCCTCTCCCCGCCGTGCGGGACACGGTTCCGGGA  GCAGAG C G  GCCGCCCACGCCCCAAGCATCCT  CATCTG  ACGAGCGGGCGCCGGCGGT
                                                        (INR+)                          (DPE-)                    
Human     CTCTTCTGTCATCTCACCGCCCCACCACAGACCGCGTTCCCCGAGG  AAAC C GGC  CGCCCACGCCCGGA  GCATCCTCCCCTG  TTGAGCGGGCGCTGACGGA
                                                          (INR-)                     (MTE+)                       
    81 Mouse: Gpc1 (14733, NM_016696) 
Human: GPC1 (2817, CN333565) 
 
           |-50      |-40      |-30      |-20      |-10      |1        |11       |21       |31       |41       
Mouse     CCGCCGTGGGGCCGGGCGGGCGGCGGGGGAGGCGCCGAGCCGGGGCTGCG C GTTCGCCCGCCGCGCTCTAGGCGGCC  CGACTG  AGCGGCCGGACCTCGCG
                                                                                        (DPE-)                  
Human     CCGCCTAGGGGCCGGGCCGGCGGCGGGGGAGGCGCCGAGCCGGGACTGCG C TAGCCCGCCGCGCTCTGGGCTGCCCGAGCGAGCGTTCGGACCTCGCACC
                                                                                                              
    82 Mouse: Lancl1 (14768, Y16518) 
Human: LANCL1 (10314, BC028685) 
 
           |-50      |-40      |-30      |-20      |-10      |1        |11       |21       |31       |41       
Mouse     GTTGATTGGCCA  GGCCTACA  GGAAGTGGGCGGTAAACGCGCCG  CATCTGT T   ATTGTCGCCGCTGCGCGGTTTG  CGGAGCCGCTGGA  GAGGCGTGCGGGTT
                        (BRE-)                           (INR-)                             (MTE-)                  
Human     CTCTGATTGGCCCTCCTGGCAGGAACTGGGCGGTAAAATAGCCCTTCTG  T T ACTGTC  CGGGGCTGCGGGG  TGAGAGGCCAGGG  CCGAGAAGGGCTTCAGG
                                                             (INR-)                    (MTE+)                     
    83 Mouse: Mr1 (15064, AF068691) 
Human: MR1 (3140, NM_001531) 
 
           |-50      |-40      |-30      |-20      |-10      |1        |11       |21       |31       |41       
Mouse     TGAGGTTAAAAAAAAAAATCGGAAAAGCAAAGGACTTCAGCACGGGTTGA T GATGCTCCTGTTACC  TCTGCTCGCTGTA  TTCTTGGTGAAGCGAAGCCAT
                                                                                 (MTE-)                         
Human     CAGTTTTTGGTTAAAAGAACCCGGAAAGAGAAGGACTATGGGG  GAACTGA T   GGCGTTCCTGTTACCTCTCA  TCATTGTGTTAAT  GGTGAAGCACAGCGAT
                                                       (INR-)                           (MTE+)                    
    84 Mouse: Tfb2m (15278, NM_008249) 
Human: TFB2M (64216, NM_022366) 
 
           |-50      |-40      |-30      |-20      |-10      |1        |11       |21       |31       |41       
Mouse     GGAAGCGAGTGAGCAAAGGACTGAGGCTAGGTCGTTCGCATGCG  CCATTC G T  AATGTTTACTTCCGCCCGGC  CTAGTGTGGTGGT  GAAATGTGTGCACGT
                                                        (INR+)                           (MTE+)                   
Human     GGAAGTGAGGGAGAAAAGCAGGAAGGCTCCGCTGTTCGCATGC  GCAGGCT C   TAGTGTTTACTTCCGCTTGACCTGGCCC  GGACGC  CAGAAAATGTTCCAC
                                                       (INR+)                               (DPE+)                
    85 Mouse: Hlx1 (15284, AF172318) 
Human: HLX1 (3142, NM_021958) 
 
           |-50      |-40      |-30      |-20      |-10      |1        |11       |21       |31       |41       
Mouse     CAAGGTCCTACCCGGGGTCTGACTCGAAAGCTCCTGCCAAAACTTTG  GGA G TTTT  TAGAGAGGAGTTTTTTTTTTTTTTTTAATTTTTTTCCTTTTTTTT
                                                           (INR+)                                               
Human     CGAGGTCCTACCCCGGGCCTGACTCGAAAGCTCCTGCCAAAACTTTG  GGA G TTTT  TAGAGACGAGTTTTTTTTTTTTTCTATTACTTTTCCCCCCCCCTA
                                                           (INR+)                                               
    86 Mouse: Hsd11b1 (15483, S75207) 
Human: HSD11B1 (3290, NM_005525) 
 
           |-50      |-40      |-30      |-20      |-10      |1        |11       |21       |31       |41       
Mouse     GTTGGCTAGTGCTGCCTGAGACTACTCCAGCCTCCCCCGTCCCTGATGTC   A CAATTCA  GAGGCTGCTGCCTGCCTGGGA  GGTTGT  AGAAAGCTCTGCAGG
                                                              (INR+)                        (DPE+)                
Human     ATTGGCTAGCACTGCCTGAGACTACTCCAGCCTCCCCCGTCCCTGATGTC   A CAATTCA  GAGGCT  GCTGCCTGCTTAG  GA  GGTTGT  AGAAAGCTCTGTAGG
                                                              (INR+)             (MTE-)       (DPE+)                
    87 Mouse: Hsd17b7 (15490, AK077914) 
Human: HSD17B7 (51478, AF162759) 
 
           |-50      |-40      |-30      |-20      |-10      |1        |11       |21       |31       |41       
Mouse     GACGCAGGAGAGGTGGAGCCGGGCCGAGCCTTGATTGGTCGAGGTG  CGCG T GGT  CACGGATCAA  GGTTCAGGCTAAG  A  GAACCC  CGGTGCAGTTCTACTT
                                                          (INR-)                 (MTE-)      (DPE-)                 
Human     ACGCAACGGGAGGCGG  GGCGTGGC  CGTACTCTGATTGGTGACGGG  TGAGG C GG  CCCGAAATCGTAG  GACTTCCGAAAGC  AGCGGCGGTGTTTGCTTCACT
                            (BRE-)                         (INR+)                    (MTE-)                         
    88 Mouse: Hspd1 (15510, X55023) 
Human: HSPD1 (3329, NM_002156) 
 
           |-50      |-40      |-30      |-20      |-10      |1        |11       |21       |31       |41       
Mouse     TTGAAGCACCGTTGG  ACAAGTT  TGTAGTGATTTTTCTTTTTTCCTTCCCC A   GAAATGCT  TCGACTACCC  ACAGTCCTTCGCC    AGATGA  GACCAGTGTCCC
                          (TATA-)                                (INR-)                 (MTE+)     (DPE+)             
Human     TGCAGCGCACCACGGGTGGTAGTTCTGATGATCTTTTTGCTTCCATCCCC A   GAAATGCT  TCGGTTACCC  ACAGTCTTTCGCC    AGATGA  GACCGGTGTCCA
                                                               (INR-)                 (MTE+)     (DPE+)             
    89 Mouse: Hspd1 (15510, AK088844) 
Human: HSPD1 (3329, NM_199440) 
 
           |-50      |-40      |-30      |-20      |-10      |1        |11       |21       |31       |41       
Mouse     CGGGGCGGGGCGGGGAGGGAGGGGACTCGGGCTCATTGCGGTGCGCGC  CC A GAGCT  GGGTCCCTCACTCGCCGC  AGACGG  CCTGCCTCGCCTCGTGCTCT
                                                            (INR+)                     (DPE+)                     
Human     CGGGAGTAGAGGCGGAGGGAGGGGACACGGGCTCATTGCGGTGTGCGCCC T   GCACTCTG  TCCCTCACTCGCCGCCGA  CGACCT  GTCTCGCCGAGCGCACG
                                                               (INR+)                     (DPE-)                  
    90 Mouse: Hspe1 (15528, AK088121) 
Human: HSPE1 (3336, BC023518) 
 
           |-50      |-40      |-30      |-20      |-10      |1        |11       |21       |31       |41       
Mouse     GGCCCCGCGCGTCCCGTGGGTGAAAGGTCAAGTGGCGTCATTTCCGGGAG G GGGGCTCGTTCTT  TCACCTCGGCGGC  CGGCCTGGAAAAGCCTAGAAAGT
                                                                               (MTE+)                           
Human     AATGCCGCGCTCCCTACGGCTCAAGGGTCAAATCGCGTCATTTCCGGGAG G GGACGAAGGGGTAGTTCTTT  CACCTCGGCTGGG  CGCCTAGAAAAGCCTA
                                                                                      (MTE-)                    
    91 Mouse: Htr2b (15559, AJ012488) 
Human: HTR2B (3357, NM_000867) 
 
           |-50      |-40      |-30      |-20      |-10      |1        |11       |21       |31       |41       
Mouse     GTGGTTTAAAAATTATAAA  TATGAAG  GCAAATTTAATATGGGAGTTG  TCA G TGTA  ATGAACAAATAACA  TAATCTTCCTCTT  AAAGTGCTTCTCAAGGGA
                              (TATA+)                        (INR+)                     (MTE+)                      
Human     TGAAAAATTTACTAATGCTAAAG  GAGAGTT  TTTAAAAACGTGATTTGTA  C A TTTGAT  GAACAAATAAATGGTC  TAATCTTCCTTTT  GGTTTCCCTTTCTT
                                  (TATA-)                      (INR-)                       (MTE+)                  
    92 Mouse: Htr2b (15559, AK033713) 
Human: HTR2B (3357, NM_000867) 
 
           |-50      |-40      |-30      |-20      |-10      |1        |11       |21       |31       |41       
Mouse     CTGCACAACACATTTGTTACACTGCTTACGAGCACCAGCCGCTAAGAC  TC A GAGCA  AGTCAGTGGGGGA  GGATCTGCCAGGA  GAGGGAGTCCAACTGAAC
                                                            (INR+)                    (MTE+)                      
Human     CTATACAACGTATTTGTTTC  ACTGCTT  TCAACCGCCTGTGCTGGAGGCTC   A GAATAAG  TCAATG  GGAGGAGGATTTC    AGTCAC  AGCAGCAAGCAAGTCTA
                               (TATA-)                          (INR-)             (MTE-)     (DPE+)                  
    93 Mouse: Idh1 (15926, NM_010497) 
Human: IDH1 (3417, BC012846) 
 
           |-50      |-40      |-30      |-20      |-10      |1        |11       |21       |31       |41       
Mouse     AGGAGACAAAGCCGGGA  AGAGTTAA  ACAGCCTTGACCCTGCCGCTGGGCT T CCGAGCGAAGGTTGTGGCAGATGCTGATC  TAACTG  GGGCCGGCTTATTA
                             (BRE-)                                                          (DPE-)               
Human     GACAAAGCCGGGAAGA  GGAAAAGC  TCGGACCTACCCTGTGGTCCCGGGTT T CT  GCAGAGTC  TACTTCA  GAAGCGGAGGCAC  TGGGAGTCCGGTTTGGGAT
                            (BRE-)                                 (INR+)              (MTE+)                       
    94 Mouse: Igfbp2 (16008, AK012703) 
Human: IGFBP2 (3485, AL527163) 
 
           |-50      |-40      |-30      |-20      |-10      |1        |11       |21       |31       |41       
Mouse     NNNNNNNNNNNNNCCGGGGGGAAGGGAGTGGTCTCCATAAGGGGGAGGGG A GAAGGCAGGGGGGCGGGGA  GAAGCAGGCTTTT  TAGGACCCGGCAGAGGC
                                                                                     (MTE-)                     
Human     CGCGAGGGAGTGTCGGGGGGGAAGGGAGTGGTCTCCAAAAGGGGGAGGGG A GAAGGCAGGGGGCGGGGA  GAAGCCGGCCCTT  TAGGACCCGGCTGCGGCG
                                                                                    (MTE+)                      
    95 Mouse: Igfbp2 (16008, AK012703) 
Human: IGFBP2 (3485, NM_000597) 
 
           |-50      |-40      |-30      |-20      |-10      |1        |11       |21       |31       |41       
Mouse     GGGGGGCGGGGAGAAGCA  GGCTTTTT  AGGACCCGGCAGAGGCGGGGGAGG A GAAAGAAGGCAAGGAGGCGTCTCCCGCATT  CGTCTG  GGCCGTGCCACCT
                              (BRE-)                                                          (DPE-)              
Human     GGGCGGGG  AGAAGCC  G  GCCCTT  TAGGACCCGGCTGCGGCGGCGAGGGAGG A GGAAGAAGCGGAGGAGGCGG  CTCCCGCGCTCGC  AGGGCCGTGCCACCTG
                    (BRE+)  (TATA-)                                                       (MTE-)                    
    96 Mouse: Igfbp5 (16011, U02023) 
Human: IGFBP5 (3488, NM_000599) 
 
           |-50      |-40      |-30      |-20      |-10      |1        |11       |21       |31       |41       
Mouse     CTGGCAGCCAGGGGCCGTCTA  TTTAAAA  GCGCCTGCTCGACCAGAGCCC  G C AGTCTC  TTTGGAAACTTC  TAAAAGAGCTAGG  AAAGAGCTGCAAAGCTGT
                                (TATA+)                        (INR+)                   (MTE-)                      
Human     TGGCAGCCAGGGGCCGGCTA  TTTAAAA  GCGCCTGCTCTCCCGGAGCCCC  G T AGTCTC  TTTGGAAACTTC  TGCAGGGGAAAAG  AGCTAGGAAAGAGCTGCA
                               (TATA+)                         (INR+)                   (MTE-)                      
    97 Mouse: Ihh (16147, NM_010544) 
Human: IHH (3549, AB010092) 
 
           |-50      |-40      |-30      |-20      |-10      |1        |11       |21       |31       |41       
Mouse     GGGTGCGGTCTGCGCGGGTCCCGAGCCCGGATCTCTTCCATTTCCCCTC  T C ACTCGA  CCCCGGGCTGCGCCGC  AGACGG  CAGCAGCTCCCGCTCTGCCCG
                                                             (INR+)                   (DPE+)                      
Human     GGGTGCGGTCTGCGCGGGGCCCGAGCCCGGATCTCTTCCATTTCCCCTC  T C ACTCGG  CCCCGGGCTGCGCCGC  AGACGG  CAGCAGCTCCCGCTCCGCCCG
                                                             (INR+)                   (DPE+)                      
    98 Mouse: Il10 (16153, M37897) 
Human: IL10 (3586, BC022315) 
 
           |-50      |-40      |-30      |-20      |-10      |1        |11       |21       |31       |41       
Mouse     CCCCACTGAGCCTTCAG  TATAAAA  GGGGGACCAAGAACAGGAGGTCT  ACA T TTAG  AGACTTGCTCTTGCACTACCAAAGCCACAAGGCAGCCTTGCAGAA
                            (TATA+)                          (INR+)                                               
Human     CCTCCCTGAGCTTACAA  TATAAAA  GGGGGACAGAGAGGTGAAGGTCT  ACA C ATCA  GGGGCTTGCTCTTGC  AAAACCAAACCAC  AAGACAGACTTGCAAAA
                            (TATA+)                          (INR+)                      (MTE+)                     
    99 Mouse: Il17 (16171, AK040420) 
Human: IL17 (3605, AY460616) 
 
           |-50      |-40      |-30      |-20      |-10      |1        |11       |21       |31       |41       
Mouse     AGGTAGTAAAACCG  TATAAAA  AGAGAGAAAGGAGCACTACTCTTCATCCA C C  TCACACGA  GGCA  CAAGTGCACCCAG  CACCAGCTGATCAGGACGCGCAA
                         (TATA+)                                  (INR+)           (MTE+)                           
Human     AAGGAGAAAAGCCC  TATAAAA  AGAGAGACGATAGCGCTACATTTTGTC  CA T CTCAT  AGCAGGCACAAACTCAT  CCATCCCCAGTTG  ATTGGAAGAAACAA
                         (TATA+)                              (INR-)                        (MTE+)                  
    100 Mouse: Il1r1 (16177, M20658) 
Human: IL1R1 (3554, BP234951) 
 
           |-50      |-40      |-30      |-20      |-10      |1        |11       |21       |31       |41       
Mouse     TAGAAGTGAGCTGTCTGTCATTCTTGTGCACGCCAGCCCAGTAAT  CATTT G GA  GGCAAAGCAAACTGTAA  GTAATGCTGTCCT  GGGCTGACTTGAGGAGG
                                                         (INR-)                        (MTE+)                     
Human     GCCTCCGTACCAGCTGG  GGCGTCCG  GCAAGATGTGAGTTGTCACTCTGCT G CG  GCACAGAC  CTGAATTAAC  AACTCTAGCTAGG  GCTGACTTCAAAAAGC
                             (BRE-)                                (INR+)                 (MTE-)                    
    101 Mouse: Il1r2 (16178, NM_010555) 
Human: IL1R2 (7850, BC039031) 
 
           |-50      |-40      |-30      |-20      |-10      |1        |11       |21       |31       |41       
Mouse     ACCACAAGCAGGAGCC  TATAAAT  CCCTGAGGGGGCTGCTGGGACA  TCACA G AA  GGTGAAAGTCTGGCCCA  GCAGCCTTCCCC  A  GTCAG  GCAAGAAGCAGC
                           (TATA+)                         (INR+)                        (MTE+)    (DPE+)             
Human     CCCAGCTCCCAAGAGGG  TATAAAT  CCCTGCTTTACTGCTGAGCTCCTGCT G   GAGGTGAA  AGTCTGGCCTG  GCAGCCTTCCCCA  GGTGAGCAGCAACAAGG
                            (TATA+)                              (INR-)                  (MTE+)                     
    102 Mouse: Inha (16322, NM_010564) 
Human: INHA (3623, M13981) 
 
           |-50      |-40      |-30      |-20      |-10      |1        |11       |21       |31       |41       
Mouse     CCCACATTCTTGGCGGGAGTGGGA  GATAAGG  CTCAGGGCCACAGA  CATCT G CG  TCAGAGATAGG  AGGTCTCAATGCC  ATGGGCAGGGGCGACTGGGACTG
                                   (TATA+)                 (INR-)                  (MTE-)                           
Human     CCCACATCCCTGGCGGGAGTGGGA  GATAAGG  CTCATGGCCACAGA  CATCT G CG  TCAGAGATAGG  AGGTCTCAATGCC  ACGGGCAGGGGCAACTCGGACTG
                                   (TATA+)                 (INR-)                  (MTE-)                           
    103 Mouse: Inha (16322, M32754) 
Human: INHA (3623, M13981) 
 
           |-50      |-40      |-30      |-20      |-10      |1        |11       |21       |31       |41       
Mouse     GGGACTGTGG  GGCGTGGG  AAGGACTGGGGGAGACTGGGGTGAGAAGGGTA G AAGAAGGCCAGCAGTGGGATGGGGAGGGGACAGTGGGGAGGTCCTAGAC
                      (BRE-)                                                                                    
Human     TCGGACTGTGG  GGCGTGGA  AAGGACTGGGGAAGACTGGATGAGAAGGGTA G AAGAGGGTGGGTGTGGGATGGGGAGGGGAGAGTGGAAAGGCCCTGGGCA
                       (BRE-)                                                                                   
    104 Mouse: Inhbb (16324, M32756) 
Human: INHBB (3625, BG995292) 
 
           |-50      |-40      |-30      |-20      |-10      |1        |11       |21       |31       |41       
Mouse     CCCGCGTTCCCTTCTGGTCCCCGCGGCGCCCCTTCTCGGCCGCGCGGGAA C CCCCGCGGCCCGGCCGGCCCCCTCCCCCTGCGAGCCGGCGGCGGCGCTC
                                                                                                              
Human     GCCCCTCGGGCCCCCGGGGCCCTCGGCGCCCCTTCCCTGCCGCGCGGGAA C CCCCGAGGCCCGGCCGGCCCCCTCCCCCTGCGAGCCGGCGGCAGCCCTC
                                                                                                              
    105 Mouse: Inpp1 (16329, AK078382) 
Human: INPP1 (3628, AF141325) 
 
           |-50      |-40      |-30      |-20      |-10      |1        |11       |21       |31       |41       
Mouse     ACCTCCAGGCGTTGGCACTCCCTTCCTCCTCTCTCGCCCCTCCTCCGCCT C TTCCGTTTCTCTGGGAAAGGCTG  TATCCT  CCTTGGGGCTCTGCATCCTC
                                                                                     (DPE-)                     
Human     GCCTAACCTCGCGCCCGGGCCGCGCCTCCTCCTCCTCCTGCTCCCCG  CCG C TTCC  GTTTCTCGA  GGGAAAGGCTGCT  GCCTCCTGCTCTGTCCTCATCCC
                                                           (INR+)                (MTE-)                           
    106 Mouse: Irs1 (16367, NM_010570) 
Human: IRS1 (3667, NM_005544) 
 
           |-50      |-40      |-30      |-20      |-10      |1        |11       |21       |31       |41       
Mouse     GAGAGGGAGAGAGACCCGGGCTGGAGCCTCCCGGCGGCGGCCAGGCTGCT G AGCGCAGAGGCTCCGTCACGTGTTTTGCTCCTCTGGAGTGAGACGGCGG
                                                                                                              
Human     GAATGTAGAGACCCGGGC  GGGAGCCT  CCCGGCGGCGGCTGCCCGGCTGCC G AGCGCGGAGGCTCCGTCACGTGT  TTTTCTCCTCCGA  GTGAGACGGCGGC
                              (BRE+)                                                       (MTE+)                 
    107 Mouse: F11r (16456, AK033574) 
Human: F11R (50848, BC057227) 
 
           |-50      |-40      |-30      |-20      |-10      |1        |11       |21       |31       |41       
Mouse     CGCCCACCG  GGCGTGGA    GAGAAAG  GCGGGCCGGGCTTAACCTGGCTGCT  A G AGTCCA  GCCCAGGGCAGT  GGATCGCGAGGCT  GTTCCCATTGGAGTTGCT
                     (BRE-)   (TATA+)                            (INR+)                   (MTE+)                      
Human     GCCAATCA  GGCGCGGA  GGGCGGGGCCGGGCGGGGTTCCACCTGG  CGGCTG G C  TCTCAGTCCCCTCGCTGTAG  TCGCGGAGCTGTG  TCTGTTCCCAGGAGT
                    (BRE-)                                (INR-)                           (MTE-)                   
    108 Mouse: Kcnh1 (16510, NM_010600) 
Human: KCNH1 (3756, NM_002238) 
 
           |-50      |-40      |-30      |-20      |-10      |1        |11       |21       |31       |41       
Mouse     GAGCGCAGCGAGGGCGGCAGCGGGAGCCAGCCGCCGCCCCTGCGCTCCG  G G GCTGCG  GAGCGCATGGGGCGCGT  GGAGCCAGGAACG  CTTGCGGGCTGCG
                                                             (INR-)                        (MTE+)                 
Human     AAGAGGGCGCGAGGGTAGCAGCCAGAGGGAGCCGCCAGCCCTGCCT  GCGG A TCC  CCGCCGGGCGC  ATGGGGCGCTTCG  AGCCGGGACTGCGTGCGGGCCC
                                                          (INR+)                  (MTE-)                          
    109 Mouse: Kcnj10 (16513, AK048864) 
Human: KCNJ10 (3766, AF482709) 
 
           |-50      |-40      |-30      |-20      |-10      |1        |11       |21       |31       |41       
Mouse     CCCCGGCCCCGGCCCCCGGAC  AAACCCT  TATCTGATTCCAGCTCCG  GGTT T AAG  AGTCTTGGCCCTGCCTG  TCGCACAGCTCCG  CTCGCCGCTCCTGCCC
                                (TATA-)                     (INR-)                        (MTE-)                    
Human     CCCCGGCCCCGGCCCCCGGAC  AAGCCCT  TATCTGATCCCAGCTCCG  GGTT T AAG  AGTCCTGGCCCGGCCCG  TCGCACAGCTCTG  CTCCTAACTCCTGCCC
                                (TATA-)                     (INR-)                        (MTE-)                    
    110 Mouse: Kcnj9 (16524, AF403130) 
Human: KCNJ9 (3765, NM_004983) 
 
           |-50      |-40      |-30      |-20      |-10      |1        |11       |21       |31       |41       
Mouse     TTGCCATAGAAACGAGAGAGGAGCAGGGGAACCTGGGAAGTGGGGATGAC   A CAGATAC  CAAGTCCT  AGTCTGAGCTGCC  GTTACATTCAGGAGAAACAGC
                                                              (INR+)               (MTE-)                         
Human     TTGCCGTGGAAACA  AGAGAGGA  GCAGGGGAGCCTGGGAAGTAGGGATGAC   A CAGATAG  CAAGTCCT  AGTCAGAGCTGCC  GCTACATTTAGGAGAAACAGC
                          (BRE-)                                (INR+)               (MTE-)                         
    111 Mouse: Kif1a (16560, BC045161) 
Human: KIF1A (547, BC064906) 
 
           |-50      |-40      |-30      |-20      |-10      |1        |11       |21       |31       |41       
Mouse     GGGGAGGGCCGGGTCGGG  GGCGCGGA  GCCCGTCACGCGGTGCCATCG  TCA C AGCA  CCAGGAGCAGGCAGTAAGAAGCATC  AGTTCC  AGGATCGGGGACCC
                              (BRE-)                         (INR+)                            (DPE+)               
Human     GGGGCGGTCCGGGCCGGG  GGCGCGCA  GCCCGTCACGCGGCGGCGCCG  TCA C AGCG  CAGAGCAGCCGGCGAGC  GGCAGCAGCTCCG  GGCTCGAGAGCCCGC
                              (BRE-)                         (INR+)                        (MTE-)                   
    112 Mouse: Kif21b (16565, NM_019962) 
Human: KIF21B (23046, AK131318) 
 
           |-50      |-40      |-30      |-20      |-10      |1        |11       |21       |31       |41       
Mouse     CTGAGGGGCTGGGGGTGGGTCCTGAGGTGGCTCGGCCCCCGCCCCCTTTG T TCGCGCTGCGGCGGGAGGTGGC  GGTCCG  GGTGGGAGGTGTGTGCGCGCG
                                                                                    (DPE+)                      
Human     GGGGCTGGCTCGGGGCGGGCTCGGGGCCGGATCGGCCCCCGCCCCCTTTG T TCGCGCTGCGGCG  GGAGGTGGCTGCC  GGGTGGGAGGTGTGTGCGCGTGT
                                                                               (MTE-)                           
    113 Mouse: Kifap3 (16579, D50366) 
Human: KIFAP3 (22920, NM_014970) 
 
           |-50      |-40      |-30      |-20      |-10      |1        |11       |21       |31       |41       
Mouse     GGGTCTGGGCTGCTCCAGTGTTTTGGGGCCCAGGAGCAGTGGGAGGAGCC G GCGGGTTCACCGT  GGTAACCGCTGCG  CCGCCGCCGCCGCCACCGCCACC
                                                                               (MTE-)                           
Human     GGGTCTGGG  CCGCTCCA  GTGTTTTGGGGCACAGAAGCTGTGGGAG  GAGCT G GA  GGCTTCACCGTGGTAACC  ACAGCGCCGCTGC  TGCCCCGCCTTGCAGG
                     (BRE+)                                (INR-)                         (MTE+)                    
    114 Mouse: Uhmk1 (16589, AK030152) 
Human: UHMK1 (127933, AK058195) 
 
           |-50      |-40      |-30      |-20      |-10      |1        |11       |21       |31       |41       
Mouse     ATTACATAGCAAA  TGCTTAGT  AAAGTCCAGTTTGCTTAGGTTTTCATCCG   G TAGTTAG  AAAGTGAAA  GGCATGAGTTTGC  CA  GGTTGT  CATGTCTTGAGT
                         (BRE-)                                 (INR+)                (MTE-)       (DPE+)             
Human     CAGGTGTCACAT  AGATGCTC    AGTAAAT  TGTCTTGCTTCGAGTCTCATCGG   A GAATGCC  GCATTCA  TTAGCATACAGAA  A  TATCTT  TTCTAGTGAGAGGAG
                        (BRE+)   (TATA+)                          (INR-)              (MTE+)      (DPE-)                
    115 Mouse: Uhmk1 (16589, AK013347) 
Human: UHMK1 (127933, AK058195) 
 
           |-50      |-40      |-30      |-20      |-10      |1        |11       |21       |31       |41       
Mouse     CTCTAGGATTGGCTTTCCCCCACTTCCGGCTGGGTCCGAGTGCACTCCGG C TTCCGGTGTCATGGC  GGCCTGAGGTCCG  GGG  AGTCGG  TCGGAGCGCTGC
                                                                                 (MTE-)        (DPE+)             
Human     TGATAGGCTCTTCCTCCATTTCCGGCTTCTGGGACTCGGGTGCACCACGG C TTCCGGTGTCATGGCTGCTTGA  AGTCCC  GGGAGTCGGTGAGGCGGCTGC
                                                                                    (DPE+)                      
    116 Mouse: Lad1 (16763, AK083526) 
Human: LAD1 (3898, BC071890) 
 
           |-50      |-40      |-30      |-20      |-10      |1        |11       |21       |31       |41       
Mouse     GGCGCGGGGCCGAGCCGGGCTGA  GATAAGC  GGCCATGTGACCC  TACCTGG C   GCGGGGAGGGGCCGCAGGTGAGCGGCGGAGCAGGGAGCTCGGCACGCGG
                                  (TATA+)                (INR-)                                                   
Human     GCGGCCGGGGCGCGCGGGGGTGA  GATAAGC  GGCCATGTGATCC  CACCTGG G   CTGGAAGGGGAGGGGCGCCAGGTGAGGCGGCGGCCGGCGGGGCGCGGGC
                                  (TATA+)                (INR-)                                                   
    117 Mouse: Lamb3 (16780, NM_008484) 
Human: LAMB3 (3914, U17744) 
 
           |-50      |-40      |-30      |-20      |-10      |1        |11       |21       |31       |41       
Mouse     GTGCAGTGACATCAGATG  GGCGTGGC  AGCTGCCAGATTCCTGAGGCCGCC A   GCAGTGGG  GCTACAC  CCAGCCAGGGAGT  CTCCAGAGGTGAGGCTGTTGT
                              (BRE-)                             (INR+)              (MTE+)                         
Human     GCAGTGACGACAGATG  GGTGTGAC  GGCTGCCAGATTCCTGAGACCC  GCCC T GCG  GTGGGGCTACAC  CCAGCCAGGGAGT  CTCCAGAGGTGAGGCTGTTGT
                            (BRE-)                          (INR-)                   (MTE+)                         
    118 Mouse: Lamc2 (16782, NM_008485) 
Human: LAMC2 (3918, NM_018891) 
 
           |-50      |-40      |-30      |-20      |-10      |1        |11       |21       |31       |41       
Mouse     CTGTCAGGACTGAGTCAGGGAGAAGAGTCGATAAAACACCTGATCGAGGG A AAAGGAAGGCACAGC  AGAGCGAAGACTC  GGCGCCCGGCAGGCACCCGCA
                                                                                 (MTE+)                         
Human     CTGTCAGGACTGAGTCAGG  TAGAAGA  GTCGATAAAACCACCTGATCAAGG A AAAGGAAGGCACAGC  GGAGCGCAGAGTG  A  GAACCA  CCAACCGAGGCGCC
                              (TATA+)                                              (MTE+)      (DPE-)               
    119 Mouse: Lhx9 (16876, BC072623) 
Human: LHX9 (56956, AK097614) 
 
           |-50      |-40      |-30      |-20      |-10      |1        |11       |21       |31       |41       
Mouse     TGGCTTTAGTCTCTT  AACAAAT  TGGACACAGCTCCGGCTGGAGCAGTCCC C AACGCAACCTACAGGCACTGG  GAACTT  GCAGGCAGCCGGGGACCGCTGA
                          (TATA+)                                                    (DPE-)                       
Human     TGGCTTTAGTCTCTT  AACAAAT  TGGACACAGCTCAGGCAGGAGCAGTCCC C AACCCAATCTACAGGCACTGG  GAACTT  GCAAGCAGCCAGGGAACGCTGA
                          (TATA+)                                                    (DPE-)                       
    120 Mouse: Xcl1 (16963, AK079820) 
Human: XCL1 (6375, D63790) 
 
           |-50      |-40      |-30      |-20      |-10      |1        |11       |21       |31       |41       
Mouse     TGCTGACCATTGAGGGT  AATAAAA  GGGGCTCCTGGGGAGTCTGCT  CCACA T TC  TTCTTGCACAGCCCAGCA  AGACCTCAGCCAT  GAGACTTCTCCTCCTG
                            (TATA+)                        (INR+)                         (MTE+)                    
Human     GCACTGACCACTGGA  GGCATAAA  AGAGGTCCTCAAAGAGCCCGATCC  TCA C TCTC  CTTGCACAGC  TCAGCAGGACCTC  AGCCATGAGACTTCTCATCCTG
                           (BRE-)                            (INR+)                 (MTE+)                          
    121 Mouse: Il1rl1 (17082, U38789) 
Human: IL1RL1 (9173, BC012580) 
 
           |-50      |-40      |-30      |-20      |-10      |1        |11       |21       |31       |41       
Mouse     GGCAACTGTATTTG  CATAAAA  CTATTAGAGAAGTCACCTTTTGCAGTTA  A C AAATTC  TGGTGGTTC  GAAGCTGAGGAAT  AA  AGATGG  CTAGGACCTCTGG
                         (TATA+)                               (INR+)                (MTE+)       (DPE+)              
Human     GTATTTAGTAAAGC  TATAAAG  CTGTAAGAGAAATTGGCTTTCTGAGTTGT G   AAACTGTG  GGCAG  AAAGTTGAGGAAG  AAA  GAACTC  AAGTACAACCCAAT
                         (TATA+)                                 (INR-)            (MTE+)        (DPE-)               
    122 Mouse: Il1rl1 (17082, U04318) 
Human: IL1RL1 (9173, BC012580) 
 
           |-50      |-40      |-30      |-20      |-10      |1        |11       |21       |31       |41       
Mouse     GTGGTAAGTTTAGGAATTTCCAGTAAGTGAGACAGCAGCATTTTTGAA  CA A GTCAT  GGATTTTGGCTA  AAACTAAAATTCT  ATGATGGGCGGGTTAAAAA
                                                            (INR-)                   (MTE-)                       
Human     TTGTCAACATCAAGAATTCTTAGTACATGATGCACCAGCATTTTTGAA  CA A GTCAT  AGATTTGGCCA  CAAATCAAATTTC  A  GGATGG  GAGGAGTGTCTCC
                                                            (INR-)                  (MTE-)      (DPE+)              
    123 Mouse: Ly9 (17085, AF244130) 
Human: LY9 (4063, AF244129) 
 
           |-50      |-40      |-30      |-20      |-10      |1        |11       |21       |31       |41       
Mouse     GCGCGTGCACTCACATACTTCAGGAAGTTGTGGTAAGTGCATCTCCTTT  A A TTTGAA  ATCTGGAAGTACACCATCATGGCG  GATCTA  AAGAGATATTGGT
                                                             (INR-)                           (DPE-)              
Human     TGCAGAAGCTGTGACACGTGCGGAAGCTGTGGTAAGTGCATCCTCCTTCA G TC  TCAGTTCT  GAAAATAGA  TCATCATGGTGGC  ACCAAAGAGTCACACAG
                                                                 (INR+)                (MTE+)                     
    124 Mouse: Ly96 (17087, NM_016923) 
Human: LY96 (23643, AB018549) 
 
           |-50      |-40      |-30      |-20      |-10      |1        |11       |21       |31       |41       
Mouse     GAGTATTAAGAAACAG  TATATTC  TAAGTCATTTTTTTCTTCTCTTGCC  TT A CAGGA  AGTGCTCAGATAT  GAAAACAAATGCA  CAGTCACACATGCAAATA
                           (TATA+)                            (INR+)                    (MTE-)                      
Human     ACAGTAAGTGTTTGA  AGAGTACA  T  TCTAAAT  CATTTTTCTCTCTTCCT  TT A CAGGA  AGTGCTCAAATGT  TGAGCACACACAC  ACAAAAAAAGAGGAAACA
                           (BRE-)    (TATA+)                    (INR+)                    (MTE+)                      
    125 Mouse: Mapkapk2 (17164, AY197741) 
Human: MAPKAPK2 (9261, CV373526) 
 
           |-50      |-40      |-30      |-20      |-10      |1        |11       |21       |31       |41       
Mouse     ATCCTCCCCTTTAAGGACTAGCCCAGGACGCCGAGCAGCCTGTGACGCGG C CGCCGCCCCCGGGCTGGT  ACTTCTCGGCGCG  GCGATTCCTCCGGCCGGG
                                                                                    (MTE+)                      
Human     CCCCTCCCCTTTAAGGACCGGCCCCGGACGCAGACGAGGCTGTGACGCGG C CGCCGGCCCGGGGCTGGGT  ACATTGTCGCGCG  GCCGCTTCCCCCCGGCC
                                                                                     (MTE+)                     
    126 Mouse: Mpz (17528, NM_008623) 
Human: MPZ (4359, AA602431) 
 
           |-50      |-40      |-30      |-20      |-10      |1        |11       |21       |31       |41       
Mouse     TCATGCTGGGGATCCAGGGGAT  TTTAAGC  AGGTTCCAGAAAACACAGC  TC A GTTCC  TTGTCCCCCGCTCTCTCCACCCCAC  AGACAC  TCTGGGCCTTTGC
                                 (TATA+)                      (INR+)                            (DPE+)              
Human     CATGCTGGGGCCCTAGGGGAT  TTTAAGC  AGGTTCCAGGAACCCCCCGT  TC A GTTCC  TGGTCCCCCA  CTTTCTCAACCCC  AC  AGATGC  TCCGGGCCCCTGC
                                (TATA+)                       (INR+)                 (MTE+)       (DPE+)              
    127 Mouse: Msc (17681, NM_010827) 
Human: MSC (9242, BC067827) 
 
           |-50      |-40      |-30      |-20      |-10      |1        |11       |21       |31       |41       
Mouse     TTTTCCCTA  AGAGTACA    GATAAAT  AGTCCTGTCAAAAGGCTCAGCTCGCG G CT  GTACTTTT  GGATCCGGAGTCA  AGTTCA  GCAGAGCCCTGGGCTACAGG
                     (BRE-)   (TATA+)                                (INR+)                (DPE+)                     
Human     TGGTTTGTTCCAA  GGAGTACA  GAT  AGCCTTT  TCAAAAGGCGCAGCTTACC G C  GGTGCGCG  CGGATTCTGGACTTGGGCGC  CAACTC  GTAGTCCACGCTCC
                         (BRE-)      (TATA-)                        (INR-)                       (DPE-)               
    128 Mouse: Mybl1 (17864, L35261) 
Human: MYBL1 (4603, AA971128) 
 
           |-50      |-40      |-30      |-20      |-10      |1        |11       |21       |31       |41       
Mouse     CATCTCTGATGCGGATGCGGCGGCGGCGGGCGGGGCGGCGGCGGGCGGGA   G CACGCCT  CCTGACATGTCCAGG  GCATCCCTGGCCG  GGCCCGGCCGCGGC
                                                              (INR+)                      (MTE+)                  
Human     CCAGGGCAGGGAGGCTGACAAGCGGCGGGAGAAGCCGGCGGAGGGCG  GGA T CGCG  CCTCCTGACATGTTGGGG  GTATCCCTGGCCG  GGCCGGGCCGGGGC
                                                           (INR-)                         (MTE+)                  
    129 Mouse: Myl1 (17901, X13130) 
Human: MYL1 (4632, J05027) 
 
           |-50      |-40      |-30      |-20      |-10      |1        |11       |21       |31       |41       
Mouse     GCCCTGTGAAATCTGATGC  TAGATAT  GAGGTCAGTTTGCCCAGAAATAAA A GGAAGCCAGAGAGAGGTTA  AGACTCCACTCAG  GGATTGGAGCTGCCTTC
                              (TATA+)                                                  (MTE-)                     
Human     TCTCTGAAACCTGATGCTAGCTGTGAGGTCAAAGCTTGCCCAGAAATAAA A GGAAGCCTCAGCCAGGGA  TGACCCCACTCAG    GGACCG  GAGCAGCCCTCA
                                                                                    (MTE-)     (DPE-)             
    130 Mouse: Myl1 (17901, K02423) 
Human: MYL1 (4632, J05027) 
 
           |-50      |-40      |-30      |-20      |-10      |1        |11       |21       |31       |41       
Mouse     TCTATTAGGCACTAAGGGAAA  TATATAT  GCATGCCCTTATACCATTTA  AC A CTCTG  GGTCCACCCTCCAG  ACGCTGGGCTGCG  GGTCAAGCGAGTCACAC
                                (TATA+)                       (INR+)                     (MTE-)                     
Human     TCTATTAGGCACTAAGTGAAA  TATATAT  GCATGCCCTTATGCCGTTTA  AC A CTCTG  GGTCCATCTTCAAG  ACACTGGGCTGTG  GATCAACCCAACCACCA
                                (TATA+)                       (INR+)                     (MTE-)                     
    131 Mouse: Myoc (17926, AF093567) 
Human: MYOC (4653, Z97171) 
 
           |-50      |-40      |-30      |-20      |-10      |1        |11       |21       |31       |41       
Mouse     CACAGGGCTGGGTCCCCAGGATA  TATAAAT  GTCTTTGGACTTCA  GGCTTG A G  CCAGCAGGGCCAC  CCATCCAGACACC  TTGCAGGAGAACTTTCCAGAAG
                                  (TATA+)                 (INR-)                    (MTE+)                          
Human     TGAAGGGCTGGCTCCCCAGTATA  TATAAAC  CTCTCTGGAGCTCG  GGCATG A G  CCAGCAAGGCCAC  CCATCCAGGCACC  TCTCAGCACAGCAGAGCTTTCC
                                  (TATA+)                 (INR-)                    (MTE+)                          
    132 Mouse: Myoc (17926, AF049794) 
Human: MYOC (4653, Z97171) 
 
           |-50      |-40      |-30      |-20      |-10      |1        |11       |21       |31       |41       
Mouse     CTCCACACTGCTGTCCTTCTCTGCACGCTGCTGCAGCTGTGGTCC  CAAGA T GC  CAGCTCTCCATCTGCTGTTT  CTGGCCTGCTTGG  TGTGGGGAATGGGG
                                                         (INR+)                           (MTE-)                  
Human     CTCTGCAATGAGGTTCTTCTGTGCACGTTGCTGCAGCTTTGGGCC  TGAGA T GC  CAGCTGTCCAGCTGCT  GCTTCTGGCCTGC  CTGGTGTGGGATGTGGGG
                                                         (INR+)                       (MTE+)                      
    133 Mouse: Myog (17928, BC048683) 
Human: MYOG (4656, NM_002479) 
 
           |-50      |-40      |-30      |-20      |-10      |1        |11       |21       |31       |41       
Mouse     TGGGTTCATGCCAGCAGGGAGGG  TTTAAAT  GGCACCCAGCAGTT  GGTGTG A G  GGGCTGCGGGAGCTTGGGGG  CCAGTGGCAGGAA  CAAGCCTTTTCCGAC
                                  (TATA+)                 (INR-)                           (MTE+)                   
Human     TGGTTCCATGCCAGCGGGGAGGG  TTTAAAT  GGCACCCAGCAGTT  GGCGTG A G  GGGCTGCTGGAG  CTTGGGGGCTGGT  GGCAGGAACAAGCCTTTTCCGAC
                                  (TATA+)                 (INR-)                   (MTE-)                           
    134 Mouse: Nab1 (17936, BC005627) 
Human: NAB1 (4664, NM_005966) 
 
           |-50      |-40      |-30      |-20      |-10      |1        |11       |21       |31       |41       
Mouse     CCCTGCCCCCCTCGATGGGCGCGGGGGCGTCCCGGCCGCCGCAGCCGCCG C AGGAGCCCGGCAGAGCCGTCGTCGCGGAGCGCGCCGCAGCTGCGGGCCG
                                                                                                              
Human     CCCTTCCCCCCTCGATGGGAGCGGGGGCGTCCCGGCTCCTGCAGCCG  CCA G AGGA  GGGAGAGCCGGG  GGCCGTCGCTTCG  G  AGTTGG  GGCTGAGCAGTCC
                                                           (INR+)                   (MTE-)      (DPE+)              
    135 Mouse: Ncf2 (17970, NM_010877) 
Human: NCF2 (4688, AY032997) 
 
           |-50      |-40      |-30      |-20      |-10      |1        |11       |21       |31       |41       
Mouse     GCGTCAGTTCCTGAACGATGGGGAAACTTCCTGACGCTACGAAGTCGC  CC A AAGGG  AGGCTAGACCCCAT  TCTGCGCGCTAGG  CTGGGACCTTGAAGCCT
                                                            (INR+)                     (MTE-)                     
Human     CAAAAGGTGGGGACATTTCCTGATGCATTTGCAACACTGAGAAGT  TATCT T AA  GGGAGGCTGGGCCCCATTCTACT  CATCTG  GCCCAGAAAGTGAACACC
                                                         (INR-)                          (DPE-)                   
    136 Mouse: Nck2 (17974, AK051665) 
Human: NCK2 (8440, AF043119) 
 
           |-50      |-40      |-30      |-20      |-10      |1        |11       |21       |31       |41       
Mouse     CAGACAAAGAGCGGCATCTGGGCGGGCGCAGCGCGGCCGCCGCCCCG  GGA C TCGC  GCTGCTGCCCTCCGGCCTC  GGCGTGCACTGTG  CGGCGAGGTAAGG
                                                           (INR-)                          (MTE-)                 
Human     CAGACAAAGAG  CGGCGCCT  GGGCGGGCGCAGCGCGGCCACCGCCCCGGGA C CCGCGCCGCTGCCCTCCGGCTCCGCGGGCGGCCCACGGCGAGGTAAGCG
                       (BRE+)                                                                                   
    137 Mouse: Ncl (17975, NM_010880) 
Human: NCL (4691, BP363509) 
 
           |-50      |-40      |-30      |-20      |-10      |1        |11       |21       |31       |41       
Mouse     CTCTGGTTGGT  GGAGCCGA  AGTCACGAGGACCCCCTTCGTCGCCTTT  CCA G AGGC  GATTACTGGGCAGGC  TCAGTCTTTTGCC  TCAGACGCTAGCTGTAG
                       (BRE-)                                (INR+)                      (MTE+)                     
Human     GCTTTGGTTGGTC  GGCGCGGA  GTCACGAGGGCGCCGTCGTCGCCTTT  CCA C AGGC  GATTACTGGGCAGGC  TCAGTCTTTCGCC  TCAGTCTCGAGCTCTCG
                         (BRE-)                              (INR+)                      (MTE+)                     
    138 Mouse: Ncoa2 (17978, AK052117) 
Human: NCOA2 (10499, AL703633) 
 
           |-50      |-40      |-30      |-20      |-10      |1        |11       |21       |31       |41       
Mouse     CTGGGGGAGGATCTCCATTGA  AATAAAC  AACCAGGCAGCAGTTATTAACA C GG  GAACATGG  CGGCCGC  AGCCTGGGCTCCC  GCGGCGGCGGCGGAGGTCA
                                (TATA+)                            (INR+)              (MTE-)                       
Human     TTGGGGGAGGATCTCCATTGA  AATAAAC  AACCAGGCAGCAGTTATTAACA C GG  GAACATGG  CGGCCGCAGCCTC  GGCTACAGCTTCG  GCGGCGAAGGTCA
                                (TATA+)                            (INR+)                    (MTE-)                 
    139 Mouse: Nek2 (18005, AK077627) 
Human: NEK2 (4751, NM_002497) 
 
           |-50      |-40      |-30      |-20      |-10      |1        |11       |21       |31       |41       
Mouse     GATGCACCTTCGCTGTGGACC  AATAGAA  TGCGAGGCCGAAGCCCGCGACG G TTGACCCGGTGGCCGGTTGCTCGC  CGACTC  AGGCAGGCAGACGAGGGGC
                                (TATA+)                                                 (DPE-)                    
Human     CGAAGCGGCCCACCGCCTGCC  AATAGAC  TGCGAGATCAGGGCCCGCGACG G   TTAAACGG  GGCCCAAGGCAGGGGTGGCG  GGTCAG  TGCTGCTCGGGGGCT
                                (TATA+)                          (INR+)                       (DPE+)                
    140 Mouse: Cd244 (18106, AK089530) 
Human: CD244 (51744, BM926312) 
 
           |-50      |-40      |-30      |-20      |-10      |1        |11       |21       |31       |41       
Mouse     AACCCACGTGGAGGGACT  GAGGCTT  TCTCGGTAGCCTTGTCAGTGCACAG C A  GAAGTGAA  CAGCCTTCAG  TTGAGGAAATTGT  GGCTGGACAGGCAGTGA
                             (TATA-)                              (INR-)                 (MTE-)                     
Human     AGACCAGCTCCA  GGCGCTGG  GGCTTTCTCAGTGGCCTTGTCAGCTCACA  G C AGGCGT  TAACAGCCTC  TAATTGAGGAAAC  TGTGGCTGGACAGGTTGCAA
                        (BRE-)                                 (INR+)                 (MTE+)                        
    141 Mouse: Npas2 (18143, NM_008719) 
Human: NPAS2 (4862, NM_002518) 
 
           |-50      |-40      |-30      |-20      |-10      |1        |11       |21       |31       |41       
Mouse     GGGTTGGGGCGCGGGGAGGTGAGTCCTGCGCACTCCGGGTCCTGCCGCCC T GGGCTTCGGGTCCT  GCCAACCGCTGCA  GCC  AGACAG  ACGGTGGGCTCCC
                                                                                (MTE-)        (DPE+)              
Human     CGGACGCGGG  GGCGCGGA  GAGGTGGGTTCCCCTCCACAGTCAGGCCGCTG G GGGCTTCGCGTCCTGCAGG  AGGTAGCAATCGC  TGGGCCGGTGGGCTCCG
                      (BRE-)                                                           (MTE-)                     
    142 Mouse: Nppc (18159, U62939) 
Human: NPPC (4880, NM_024409) 
 
           |-50      |-40      |-30      |-20      |-10      |1        |11       |21       |31       |41       
Mouse     ACATCAGCGGCAGGTTGGAT  TATAAAG  GCTCCAGCGGAGCCACGGAC  TCA G AGCG  CACCCAGCCGGCGCCGCACAGCAGTA  GGACCT  GTGCTCGCTTGGC
                               (TATA+)                       (INR+)                             (DPE-)              
Human     ACATCAGCGGCAGGTTGGAT  TATAAAG  GCGCGAGCAGAGTCACGGGC  TCA G AGCG  CACCCAGCCGGCGCCGCGCAGCACTG  GGACCC  TGCTCGCCCTGCA
                               (TATA+)                       (INR+)                             (DPE-)              
    143 Mouse: Slc11a1 (18173, AJ458183) 
Human: SLC11A1 (6556, D50402) 
 
           |-50      |-40      |-30      |-20      |-10      |1        |11       |21       |31       |41       
Mouse     ATGAGGTCTGGAGGGGATGGGAAGGGCGTGGGTTCCCACTCTTAC  TCACT C GG  ACCAGCACCCACAGAA  GGGGACAGATTGA  GGAGCTAGTTGCCAGGCC
                                                         (INR+)                       (MTE-)                      
Human     CAGGGCACTCGGCTGCGGATGGGTAACAGGGCGTGGGCTGGCACAC  TTAC T TGC  ACCAGTGCCCAGAGAGGG  GGTGCAGGCTGAG  GAGCTGCCCAGAGCA
                                                          (INR+)                         (MTE-)                   
    144 Mouse: Ddr2 (18214, NM_022563) 
Human: DDR2 (4921, NM_006182) 
 
           |-50      |-40      |-30      |-20      |-10      |1        |11       |21       |31       |41       
Mouse     GGCATCTTGCATCAGCCT  GTGGATG  TATGCCTACCACGGGGCTCCTTCAC C G  GCAAAGTG  GAAAAA  GAAGTGGTTCCCG  AGAAAGTCTTCTGGGTTGGGG
                             (TATA-)                              (INR+)             (MTE+)                         
Human     GGCATCTTGCATCAGCCT  GTGGATG  TATGCCTACCACCGGGCTCCTTCAC C A  GCAAAGTG  GAAAAA  GAAGCGTTTCACA  ACAAATTCTTCTTTTTGGGTT
                             (TATA-)                              (INR+)             (MTE+)                         
    145 Mouse: Oprk1 (18387, D31663) 
Human: OPRK1 (4986, AY466378) 
 
           |-50      |-40      |-30      |-20      |-10      |1        |11       |21       |31       |41       
Mouse     TGCAGCCCCGCAAGTGCCACCTTCTCGCTTTCCAGCTGCAGCGCTCACCA T   GGAGTCCC  CCATTCAGATCTT  CCGAGGAGATCCA  GGCCCTACCTGCTCT
                                                               (INR+)                    (MTE-)                   
Human     GCGCCGCA  GGTGCCGC  CTGTCCTCGCCTTCCTGCTGCAATCGCCCCACCA T   GGACTCCC  CGATCCAGA  TCTTCCGCGGGGA  GCCGGGCCCTACCTGCGCC
                    (BRE-)                                       (INR-)                (MTE+)                       
    146 Mouse: Pam (18484, AK051649) 
Human: PAM (5066, CB215484) 
 
           |-50      |-40      |-30      |-20      |-10      |1        |11       |21       |31       |41       
Mouse     AGCCGCCCCGGGGGAGGGAGCGGGCGGTCCCGGAGCCCAGCGCGCGGCCC G GGAAGGAAGGAGGCCAGCAGCGGCCGCGGCCGCGTGCTCAGCCTGTCCC
                                                                                                              
Human     CCCCAGGAGTCGAGGGCGAGCGGCGGACCCGGCTGGGCCCGGC  GCACGCC G   AGGAGAGGGAGCCCCCGC  CACCGCCGCTGCC  TCCGCGTGCTCAGCCTGT
                                                       (INR+)                         (MTE-)                      
    147 Mouse: Pax3 (18505, NM_008781) 
Human: PAX3 (5077, AJ007392) 
 
           |-50      |-40      |-30      |-20      |-10      |1        |11       |21       |31       |41       
Mouse     GCCCCGCCTCCCTCTCT  GGCCTTT  TTTGGGGGAGGAGCTCTCCGAGATCC G G  AGAGTTCC  CGAGGGTCAC  CCGCCGCACTGTG  CTCGCTTTTTCGTCTCG
                            (TATA-)                               (INR+)                 (MTE-)                     
Human     CGTCCCTCCCTCTCTCTCCAGCCGTTTTGGGGAGGGGCTCTCCACGCTCC G G  ATAGTTCC  CGAGGG  TCATCCGCGCCGC  ACTCGCCTTTCCGTTTCGCCT
                                                                (INR+)             (MTE+)                         
    148 Mouse: Pax3 (18505, NM_008781) 
Human: PAX3 (5077, AJ007392) 
 
           |-50      |-40      |-30      |-20      |-10      |1        |11       |21       |31       |41       
Mouse     GATCAAGCTTTGGGTGAAAGAACT  AATAAAT  GCTCCCTAGTCCGGATCCC T   GCACTCGG  TGTCACGACG  GGAGGAGACTTGG  GACGTGTTCCTGCCTCGT
                                   (TATA+)                       (INR+)                 (MTE-)                      
Human     GACCAAGCTTTGGGTAAAAGAACT  AATAAAT  GCTCCCGAGCCCGGATCCC C   GCACTCGG  TGTCACCACA  GGAGGAGACTCAG  GCAGGCCGCGCTCCAGCC
                                   (TATA+)                       (INR+)                 (MTE-)                      
    149 Mouse: Pde6d (18582, NM_008801) 
Human: PDE6D (5147, NM_002601) 
 
           |-50      |-40      |-30      |-20      |-10      |1        |11       |21       |31       |41       
Mouse     GCCAAGGCT  GGCTTGTC    ATTGGTT  TCAAGGCGGGAGCTCCACGA  CTAGAG G G  AAGGAGAAGGGATCA  GGAGCGGGAGCTG  AGGGGAGGGAGAGGCCGGTT
                     (BRE-)   (TATA-)                       (INR+)                      (MTE+)                        
Human     CGCCAAGGCT  GGCTTTCC  ATTGGTTCCAGGAGGGCACTGCGAGGCTAGGG G GAAGGAGAAGGGATCAGA  AGCGGGAGCTGAG  GGGAGAGAGAGGCCGGTC
                      (BRE-)                                                          (MTE-)                      
    150 Mouse: Pea15 (18611, AK077421) 
Human: PEA15 (8682, AF153272) 
 
           |-50      |-40      |-30      |-20      |-10      |1        |11       |21       |31       |41       
Mouse     GTGGGGTGAGGCGGTGCTGGGCTCAAGCTCTGCTCCACGGGCGGAAGAGG C GGCGGCGGCAGGATCGGCG  GCAGCGGTGGCAG  GAGCCAAGGAGCAGGCT
                                                                                     (MTE+)                     
Human     GTGGAGTGAGCGGCGCTGGGCTCGGGCTCCGGCTCCGCGGGCGGAAGAGG C GGCGGCGGCGGCA  GAAGCGGCGGCGG  CGGCGGCGGGAGCCGAGGAGGAG
                                                                               (MTE+)                           
    151 Mouse: Per2 (18627, NM_011066) 
Human: PER2 (8864, BF373017) 
 
           |-50      |-40      |-30      |-20      |-10      |1        |11       |21       |31       |41       
Mouse     TGCAAATGAGGTGGCACTCCGACCAATGGCGCGCGCAGGGGCGGGCTCAG C GCGCGCGGTCACGTTTTCCACTATGTGACAGCGGAGGGCGACGCGGCGG
                                                                                                              
Human     GCAGATGAGAC  GGAGTCGC  GGCCAATGGCGGAGGCCGGGGGCGGGCGCGG C GCGCGCGGTCACGTTTTCCACTATGTGACAGCGGCGACTCGGCCGCGGC
                       (BRE-)                                                                                   
    152 Mouse: Pfdn2 (18637, BC049606) 
Human: PFDN2 (5202, NM_012394) 
 
           |-50      |-40      |-30      |-20      |-10      |1        |11       |21       |31       |41       
Mouse     ACCCCCCGGCCGGATGT  GACGCAGT  GAGAGTGCGCAGGCCGGAAAGCTT  G C AGGTGG  GGAAGATGGCGGACAGCAGC  GGTCGT  GTGGGCAAGAGCGGCGG
                             (BRE-)                            (INR+)                       (DPE+)                  
Human     GCCGGACG  TGACGCAA  GGCGGGGGCTGAGCTGCGCCGGCCGGAAACCCA  G C AGGCGG  CGAAGATGGCGGAGAACAGC  GGTCGC  GCCGGCAAGAGCAGCGG
                    (BRE+)                                     (INR+)                       (DPE+)                  
    153 Mouse: Pfkfb2 (18640, AK016729) 
Human: PFKFB2 (5208, CR749442) 
 
           |-50      |-40      |-30      |-20      |-10      |1        |11       |21       |31       |41       
Mouse     GCGCCCCACGTGACTCGCCCCAACCCCCGGAGCCCGGACTACT  TCAGTCT G   TCTGCCAGCTTTGGT  ACCCGGTGCTCCG  GTGGCGACACTGGGTCTACAG
                                                       (INR+)                      (MTE-)                         
Human     GGGCTCACATGATTTGC  CGGCGACT  GTAGCGCCGGTCCCGGCCAC  AAGCT G TC  GGCTCGGTTCGGTCGCG  TTACAGGGCAGGC  GCCGGGGCCAAGGCAGG
                             (BRE+)                        (INR-)                        (MTE-)                     
    154 Mouse: Pigr (18703, Y16523) 
Human: PIGR (5284, X73079) 
 
           |-50      |-40      |-30      |-20      |-10      |1        |11       |21       |31       |41       
Mouse     TGACTATGGGACAACGGCCC  TTTAAGA  GCCAGGTGCGGGTCAAGCCCAGA A A  ACAGTTTC  AGTTTTGACAAT  AGTCAC  CAGTAGTGCCTTCCTGGAAGCT
                               (TATA+)                            (INR+)               (DPE+)                       
Human     GCCTGTGGGAGAGTGGCCC  TTTAAGA  GCCCAGGTGTGGGTCAA  ACACTGA G   CAGAGTTTCAGTTTTGGCAGCAG  CGTCCA  GTGCCCTGCCAGTAGCTCCT
                              (TATA+)                    (INR-)                          (DPE-)                     
    155 Mouse: Pkp1 (18772, NM_019645) 
Human: PKP1 (5317, Z34974) 
 
           |-50      |-40      |-30      |-20      |-10      |1        |11       |21       |31       |41       
Mouse     GGCGCAGGGCA  GGGGTGG  T  ATATCC  TGTCTGACGGAGGGCGGGTCCGG  CC A GTGCC  TACTGAGGGA  CGAGCCGGGGCGG  AGGCGCCAGGAGCAGCAGCCG
                       (BRE-)  (TATA+)                          (INR+)                 (MTE+)                         
Human     GGTGCAGGGCA  GGGGTGG  T  ATATCC  TGTCTGACGGAGGGCGGGCCTCG  CC A GTGCC  AGAGAGGGA  CGAACCAGGGTGG  AAGCGCCAGGAGCAGCTGCAGG
                       (BRE-)  (TATA+)                          (INR+)                (MTE+)                          
    156 Mouse: Lypla1 (18777, BC052848) 
Human: LYPLA1 (10434, AF052112) 
 
           |-50      |-40      |-30      |-20      |-10      |1        |11       |21       |31       |41       
Mouse     GGGACTTTCGGCTGCCGGGAGCCCGAGTTCCCTTCCGCTTCCGAC  GCACT G TC  CGCCAGCCGGTGGATGTGCGGCAACAACA  TGTCCG  CTCCGATGCCCG
                                                         (INR-)                                (DPE-)             
Human     GCACGCCCTTGGGCCGCGGCCGGGCGCCCGCTCTTCCTTCCGCTTGC  GCT G TGAG  CTGAGGCGGTGTAT  GTGCGGCAATAAC  ATGTCAACCCCGCTGCCC
                                                           (INR-)                     (MTE-)                      
    157 Mouse: Pla2g4a (18783, BC003816) 
Human: PLA2G4A (5321, M68874) 
 
           |-50      |-40      |-30      |-20      |-10      |1        |11       |21       |31       |41       
Mouse     TAACATCCACAGAGACCAGCCCACTTCTTAGCCCCTCCTACCAGCCGGAG A   AGACTTTC  TAGAAGTCGGAACT  GTGAAGGGCTCCC  GACTGAGAGCCAGA
                                                               (INR+)                     (MTE-)                  
Human     GCAAAAGCGCAA  GGAGACCA  GCC  CACATTT  TAGCCCCTCCTACTCAGGAT A   AGACTTTC  TCTAA  GTCCGGAGCTGAA  AAAG  GATCCT  GACTGAAAGCTAG
                        (BRE-)      (TATA-)                        (INR+)            (MTE-)         (DPE-)              
    158 Mouse: Serpinb2 (18788, AK081487) 
Human: SERPINB2 (5055, NM_002575) 
 
           |-50      |-40      |-30      |-20      |-10      |1        |11       |21       |31       |41       
Mouse     TGTGTGGGAGGGGCAGAGTCT  TATAAAA  CCAGTCATTACCTTGT  CCACAC A G  TGCCAGCTTTCCAAGAA  GCATTTCCTGTGT  GTCAGCCGCTCAGAAGAT
                                (TATA+)                   (INR+)                        (MTE+)                      
Human     CATGTGGGAGGGGCAAAGCTG  TATAAAA  CCAGTCATTACCATGTCT  GAAC T GTA  ACAACTCTCAGAGGA  GCATTGCCCGTC  A  GACAG  CAACTCAGAGAAT
                                (TATA+)                     (INR-)                      (MTE+)    (DPE+)              
    159 Mouse: Plcd4 (18802, AK083793) 
Human: PLCD4 (84812, NM_032726) 
 
           |-50      |-40      |-30      |-20      |-10      |1        |11       |21       |31       |41       
Mouse     CCCACTCCTCCTCTCTGGCTGTAACTCTGCCAGTTTACAGCAGC  AAACTG C A  GTGGAGGGTGGAAGCAGT  CAAGTGCAAAGGA  GAAAAGGAGTCTTGTTC
                                                        (INR-)                         (MTE+)                     
Human     TTCTTACTGCTTCCCTCCGGC  TATAACT  TGCCAGTCACAGCAG  CCAGCTG C   TGTAGAAGAGGGGAGGAAA  CAAGCCAGTGCAA  GGGGAGCAAAAGAGAAA
                                (TATA+)                  (INR+)                          (MTE+)                     
    160 Mouse: Prrx1 (18933, L06502) 
Human: PRRX1 (5396, NM_022716) 
 
           |-50      |-40      |-30      |-20      |-10      |1        |11       |21       |31       |41       
Mouse     GAAGTAACCGAGGCTGCAGGGAAAGTTACTTGTCCCGAAAGTTCT  ACACT T CA  GAAGGGGTCCCACCCTCT  GTTTCTCTCTCTC  TCTCTCTCTCTCTCTC
                                                         (INR+)                         (MTE+)                    
Human     GGAAATCAACCAATGCTGCGGAAGGCGGCTGGTGCACAACGCTCTGCTCT   A CAGAAGG  GGGTCCCCCACCCT  CTTTTCCAATTTT  TTTTTTTTGGCCTTC
                                                              (INR+)                     (MTE-)                   
    161 Mouse: Pou2f1 (18986, AK028237) 
Human: POU2F1 (5451, NM_002697) 
 
           |-50      |-40      |-30      |-20      |-10      |1        |11       |21       |31       |41       
Mouse     GGGGGAGGGGAGCGGGAGCGAGG  GAGGGTT  TATCGACCGGGCGATTTTGG T T  AAAATATT  CAAAATGGCGGACGGAGGAGCAGCGAGTCAAGATGAGAGT
                                  (TATA-)                         (INR-)                                          
Human     GGGGGAGGGGAGCCAGAGCGAGG  GAGGGTT  TATCGACCGGGCGATTTTGG T T  AAAATATT  CAAAATGGCGGACGGAGGAGCAGCGAGTCAAGATGAGAGT
                                  (TATA-)                         (INR-)                                          
    162 Mouse: Pou3f3 (18993, BC079869) 
Human: POU3F3 (5455, CR599621) 
 
           |-50      |-40      |-30      |-20      |-10      |1        |11       |21       |31       |41       
Mouse     AGTGTGGTGGTGGGT  GCGCGCTT  GCGGCGGAGGGTAAACATTCG  ACAGTC C C  TGCTCTGAGAGGGAGGGACGGAGAGC  GAACTG  TCAGATCCTAGCGGGA
                           (BRE+)                         (INR+)                             (DPE-)                 
Human     AGTGTGGTGGTGGGT  GCGCGCTC  GCGGCGGAGGGTAAACATTCG  ACAGTC C C  CGCTCTGAGAGGGAGGGACAGAGAGC  GAACTG  TCAGATCGGAGCGAGA
                           (BRE+)                         (INR+)                             (DPE-)                 
    163 Mouse: Ppox (19044, NM_008911) 
Human: PPOX (5498, CR613738) 
 
           |-50      |-40      |-30      |-20      |-10      |1        |11       |21       |31       |41       
Mouse     CCTTATCTGCACACGGACAGGATGGACTGCTCCTAGGGCCTCTA  TCAGTC A C  TCACTTTCTCGGGTTCCCC  CTATCTCGTGGGT  AAGTTCTGGCTTCAGA
                                                        (INR+)                          (MTE+)                    
Human     CCCAGCAGAGCGCCGGCGGGGTACGGTCTTAGGACCTCGATCTCCTTCTC C C  TCATTTTC  TCTCATCCCTAC  CTATTGTGGGTGA  GTCCTGGCCCCTGGA
                                                                (INR+)                   (MTE+)                   
    164 Mouse: Ppox (19044, NM_008911) 
Human: PPOX (5498, CR613738) 
 
           |-50      |-40      |-30      |-20      |-10      |1        |11       |21       |31       |41       
Mouse     AAACGCAGTCACGCAGGCCGC  CAGAAAG  GAATCGACGGAGAGTCGGAGGG A CCCTAAAAGTTAG  GGGCGGGGATACC  AGACTTGAACCTGCTCACCCCTG
                                (TATA+)                                          (MTE-)                           
Human     GTAGGGTTA  GGCGCGTG  CCGCGAGAACAGAGTGGACGGAGCGTAGG  AGAG A CCG  AAAAGGCTGGGGGTGGG  AGTAGCGGATTTG  AAGCACTTGTTGGCCT
                     (BRE-)                                 (INR+)                        (MTE-)                    
    165 Mouse: Prei3 (19070, AK034840) 
Human: PREI3 (25843, NM_015387) 
 
           |-50      |-40      |-30      |-20      |-10      |1        |11       |21       |31       |41       
Mouse     CCCGCGCAAGCTGCCGGCCCCCACATCCGGGTACCGACGCCAGCCGC  CCA G ACTC  TGGCACTATGGTCATGGCGGAGGGGACGGCAGTGCTGAGGCGGAA
                                                           (INR+)                                               
Human     CCCCGCGCAGGCTGCCGTCCCTACATCCGGGTACCGACTCCAGCCGC  CTA G ACGC  TGGCACTATGGTCATGGCGGAGGGGACGGCAGTGCTGAGGCGGAA
                                                           (INR+)                                               
    166 Mouse: Prim2 (19076, AK077760) 
Human: PRIM2A (5558, BC017833) 
 
           |-50      |-40      |-30      |-20      |-10      |1        |11       |21       |31       |41       
Mouse     CGCCAGGGGGTGGGACCT  CTGCGCAT  CACTTCCGCTTCCCGTT  CCAAATG A   ACTCTCCCGCCACTGGGAG  AGAGCGGCTGTCA  GCGCGGGTGTTTCTTTC
                              (BRE+)                     (INR+)                          (MTE+)                     
Human     GGGCAGGGCTGGCCTT  CTGTGCTA  GTCACTTCCTCTTCCGGTTT  CATATG A A  CTCTCCCGCCACCCGGG  AACAGTGGCTGCC  ACCGTTTGTGTTTTCCCG
                            (BRE+)                        (INR-)                        (MTE-)                      
    167 Mouse: Ptgs2 (19225, M64291) 
Human: PTGS2 (5743, AF276953) 
 
           |-50      |-40      |-30      |-20      |-10      |1        |11       |21       |31       |41       
Mouse     GTGGAGTCCGCTTT  ACAGACT  TAAAAGCAAGGTTCTCCCCATTAGCAG  CC A GTTGT  CAAACTGCGA  GCTAAGAGCTTCA  GG  AGTCAG  TCAGGACTCTGCT
                         (TATA-)                              (INR+)                 (MTE-)       (DPE+)              
Human     GGGCTTGGTTTTCAGTCT  TATAAAA  AGGAAGGTTCTCTCGGTTAGCGAC  C A ATTGTC  ATACGACTTGCAG  TGAGCGTCAGGAG  CACGTCCAGGAACTCCT
                             (TATA+)                           (INR-)                    (MTE+)                     
    168 Mouse: Ptma (19231, AK088005) 
Human: PTMA (5757, AF452640) 
 
           |-50      |-40      |-30      |-20      |-10      |1        |11       |21       |31       |41       
Mouse     GCTGAGCACCGCCCACTAATCTA  TATTAAA  GCTTCTGGCGCCGCGTGAGT C CC  CCACTGGC  TGCTCCGAAAAGC  CATCTT  TGCATTGTTCCTGGGTCGTG
                                  (TATA+)                          (INR-)                (DPE-)                     
Human     GCCGAGCGCCGCCCACTAATCTA  TATTAAA  GCTTCTGGCGCCGCGTGAGT C CC  CCACTGGC  TGCTCTGAAAAGC  CATCTT  TGCATTGTTCCTCATCCGCC
                                  (TATA+)                          (INR-)                (DPE-)                     
    169 Mouse: Ptpn4 (19258, AK046683) 
Human: PTPN4 (5775, M68941) 
 
           |-50      |-40      |-30      |-20      |-10      |1        |11       |21       |31       |41       
Mouse     GAGGGTGACCGAGGCTCTCCTTTGCACTCTCACACAGAGCCGCCTCCCT  G G AGTCAC  CCGCCGCTTCACGC  CGCGCGCGCTTCC  CTGCTCCGCGGACGCG
                                                             (INR-)                     (MTE-)                    
Human     AGGGGGGTGACT  GAGCTCCT  CTTGCACTCTCACACACAAACGCTGCCCA  G G ATTACC  CGCCAGCTCACGCCGC  GCAGTGCGCTTTT  CCGCTCCTCGCGCC
                        (BRE+)                                 (INR-)                       (MTE-)                  
    170 Mouse: Ptprc (19264, AK054056) 
Human: PTPRC (5788, M23461) 
 
           |-50      |-40      |-30      |-20      |-10      |1        |11       |21       |31       |41       
Mouse     CACCATTTAGCAGTGCATGTAGCTAGCAAGTGGTTTGTTCTTAGGGTAAG   A GAGTAGG  AAACTTGCTCC  CCATCTGATAAGA  CAGAGTGCAAAGTATGCG
                                                              (INR-)                  (MTE+)                      
Human     CATCACCTAGCAGTTCATGCAGCTAGCAAGTGGTTTGTTCTTAGGGTA  AC A GAGGA  GGAAATTGTTC  CTCGTCTGATAAG  ACAACAGTGGAGAGTATGCA
                                                            (INR+)                  (MTE-)                        
    171 Mouse: Ptprc (19264, M23354) 
Human: PTPRC (5788, M23461) 
 
           |-50      |-40      |-30      |-20      |-10      |1        |11       |21       |31       |41       
Mouse     GTCTGAGCTCCTTATGG  TAAATAA  CAGGAGTTGGCAGACATAAGCAGAAA G   ACAGTTGG  TTTGGGTCACTTGGT  CGTCTT  CAACGAACTTCAGAGCCTCG
                            (TATA+)                              (INR+)                  (DPE-)                     
Human     TTGAATGCCCTAAT  GGTAAAT  GATACTGGGTTGCCAAAAATAA  CCAGATT A   GTAGTTTTTTCATTCATTTGGC  CGTCTC  AGTAAGTCAAATATTGATACT
                         (TATA+)                         (INR+)                         (DPE-)                      
    172 Mouse: Ptprn (19275, NM_008985) 
Human: PTPRN (5798, AK054765) 
 
           |-50      |-40      |-30      |-20      |-10      |1        |11       |21       |31       |41       
Mouse     CGCCTAGGCTCCTGGGCTGACTCCCACCAACGTCGCCGAGACTCTGGCCG G AGAGAGAGCTCAGAGC  CGGGAAGGATGAG  GCGCCCGCGGCGGCCCGGGG
                                                                                  (MTE-)                        
Human     CTCAGCCCCTCTGGCA  GGCTCCCG  CCAGCGTCGCTGCGGCTCCGGCCCGG G AGCGAGCGCCCGGAGCTCGGAA  AGATGC  GGCGCCCGCGGCGGCCTGGGG
                            (BRE-)                                                    (DPE+)                      
    173 Mouse: Pex19 (19298, AK029368) 
Human: PEX19 (5824, NM_002857) 
 
           |-50      |-40      |-30      |-20      |-10      |1        |11       |21       |31       |41       
Mouse     CGGGGCATGTGG  TGGCGCCA  GTGGTTCCACCTCCTACGGCAAGTCGGAG  G C AGTAAG  ATGGCGGCTGCTGAGGAA  GGTTGC  GGTGTTGGGGTCGAAGACG
                        (BRE+)                                 (INR+)                     (DPE+)                    
Human     GAGGGCGTGTC  GGCACCGA  GGAGGTCCCGCCTCCTACGGCAAGTCGGAGG T AG  CAAGATGG  CCGCCGCT  GAGGAAGGCTGTA  GTGTCGGGGCCGAAGCGG
                       (BRE-)                                      (INR+)               (MTE-)                      
    174 Mouse: Rab17 (19329, AK075591) 
Human: RAB17 (64284, AK022600) 
 
           |-50      |-40      |-30      |-20      |-10      |1        |11       |21       |31       |41       
Mouse     GCTTACTCTGACCCAGCTGATCACATGATCATGTGGGGACTTTGCACC  GG A GTTTA  CAGGAAGAGT  CCTGCCGGCTGGG  GCCTTGGTGGGAGCAGGGGGC
                                                            (INR+)                 (MTE-)                         
Human     CTCCTGCCCTGCCTCA  GGTGACCA  CATGACCACTGTGGACTTTGCCCTGA A AC  CTTCTGGG  AGGAGAAGAGGCC  TGACCT  TGGCGCTGGGGTCCAGTGGG
                            (BRE-)                                 (INR-)                (DPE-)                     
    175 Mouse: Rab23 (19335, AK047068) 
Human: RAB23 (51715, AF161486) 
 
           |-50      |-40      |-30      |-20      |-10      |1        |11       |21       |31       |41       
Mouse     GCATGCTCCTCAGCGCGGCGGGCGGGAGTCCGGCGCGCCCTCCATCCCCG G CGGCGTCGGCAAGGA  GTAGCTCGGTGAA  CCC  GGATAC  TAATAAAGTTCG
                                                                                 (MTE+)        (DPE+)             
Human     GCGGGTCCACAGATGAGG  GGAGTCGA  GACTCCCGCGCCCCACCACCCCCG G CG  GAGCTGCT  GCTGAGCCACT  CAATCTGAGCCCT  GGCTACTAATAAAGT
                              (BRE-)                               (INR-)                  (MTE+)                   
    176 Mouse: Ren2 (19702, K02597) 
Human: REN (5972, CB044993) 
 
           |-50      |-40      |-30      |-20      |-10      |1        |11       |21       |31       |41       
Mouse     CAGAGCCTGTGA  TACATGGT  GTG  TATAAAA  GAAGGCTCAGGGGGTCTGGG   C TACACAG  CTCTTAGAA  AGCCTTGGCTGAA  CC  AGATGG  ACAGAAGGAGGA
                        (BRE-)      (TATA+)                       (INR+)                (MTE-)       (DPE+)             
Human     TCACCCCA  TGCATGGA  GTG  TATAAAA  GGGGAAGGGCTAAGGGAG  CCACAG A A  CCTCAGTGGATCTCAGAG  AGAGCCCCAGACT  GAGGGAAGCATGGATGG
                    (BRE-)      (TATA+)                     (INR+)                         (MTE+)                     
    177 Mouse: Rgl1 (19731, U14103) 
Human: RGL1 (23179, AF186781) 
 
           |-50      |-40      |-30      |-20      |-10      |1        |11       |21       |31       |41       
Mouse     TCTGCTGCCGCGGGCTCCCGGGCTGCCTGCTCGCTGGCCGGCGCTCC  TTT G TGGC  CCGGTCGCGCGCGGCGGCGGCGGCGGC  AGTCGG  GCAGCAAGGCGC
                                                           (INR-)                              (DPE+)             
Human     TGGGGAGGAGT  CTGCTCCT  CGCTTGCTCGCTCGCTCGCCGCGCTC  CCTTT G TG  GCCCGAGTCGCGCGCACCGGCGGCGGCGGGGGCAGCGCGGCGCGTGT
                       (BRE+)                              (INR-)                                                 
    178 Mouse: Rgs2 (19735, AK077922) 
Human: RGS2 (5997, L13463) 
 
           |-50      |-40      |-30      |-20      |-10      |1        |11       |21       |31       |41       
Mouse     GCCCCTATGCCGCCG  CCCGCCT  TAAAAGCCCGCGGCGCGCTCCCGGCC  TC A AACAG  CTGCGGTGGCCGCGGGA  GTCTGAGAATGCA  AAGTGCCATGTTCC
                          (TATA-)                             (INR+)                        (MTE-)                  
Human     CCCGCCCCCAAGCCGAGGCCT  CATAAAT  GCTGCGACGCACGCCCAGCC  GC A AACAG  CCGGGGCT  CCAGCGGGAGAAC  GATAATGCAAAGTGCTATGTTCT
                                (TATA+)                       (INR+)               (MTE+)                           
    179 Mouse: Xpr1 (19775, NM_011273) 
Human: XPR1 (9213, NM_004736) 
 
           |-50      |-40      |-30      |-20      |-10      |1        |11       |21       |31       |41       
Mouse     TAGGCGGTTACCAT  GGCGATGA  CGTCTAGAGGGCGGGGCGGGGCGGG  GCT A TGGA  GAGGAGGAGGAAGATGGCGGGAGGGCGGCTCTGAGGAGACCTCGG
                          (BRE-)                             (INR-)                                               
Human     TAGGCGGTTACCAT  GGCGATGA  CGTCCAGAGGGCGGGGAGGGGCGGG  GCT A TGGA  GAGGAGGAGGAAGATGG  CGGGCGGGCTGCT  CTGAAGAGACCTCGG
                          (BRE-)                             (INR-)                        (MTE-)                   
    180 Mouse: Rp1h (19888, AF291754) 
Human: RP1 (6101, AF143222) 
 
           |-50      |-40      |-30      |-20      |-10      |1        |11       |21       |31       |41       
Mouse     CTAATAAGGACTCTT  AATAGAG  AGACTTCAGGGCTGTGGCACCCTTGAGG T GAGCTACTTCTGGAGATTAACAT  CACCTTGGATTAA  ATTAATTGGCTGT
                          (TATA+)                                                          (MTE-)                 
Human     TGCAGAGCATGCTAGGAACTG  GTTTGCT  TCTGGCTGTTGTCTCCTTAGGG T GA  GCTCTGTC  TGGTGATTAGCAT  CACCATGGATTAA  ATTAATTGGCTGT
                                (TATA-)                            (INR-)                    (MTE-)                 
    181 Mouse: Rpl7 (19989, M29015) 
Human: RPL7 (6129, BE694494) 
 
           |-50      |-40      |-30      |-20      |-10      |1        |11       |21       |31       |41       
Mouse     CTTCGCGCCCTGAGACC  TTTAAGG  AGACAGTGCGCATGCGCCACCTTTCT C TCTTCTTTTCCGGCT  GGAACCATGGAGG  CTGTTCCGTAAGTCGATTCTT
                            (TATA+)                                                (MTE+)                         
Human     GCTTCTCTCG  CGAAGTC  T  TTAAGT  GGACAGTACGCATGCGCCAACTTCCT C TTTTTCCGGCTGGAAC  CATGGAGGGTGTA  GAGTGAGTTGCTTCTGGTCC
                      (BRE+)  (TATA+)                                                 (MTE-)                        
    182 Mouse: Pdc (20028, AK020798) 
Human: PDC (5132, AF076464) 
 
           |-50      |-40      |-30      |-20      |-10      |1        |11       |21       |31       |41       
Mouse     TCATCAATATCCAGGGAT  CTTAAAT  CTGAGCTTAACACTGGTCCAAGTTC A AA  GGAGTTCA  GTGG  ACAGCGATTCTCA  CTCCCTGGACATATTCTAGAAC
                             (TATA+)                               (INR+)           (MTE+)                          
Human     GTCATCAATATCTG  GAGATTT  TAAATCTGAGCTTAAACCTATT  GAAGTTC A   AGACGAGTTCAGT  AGACAGGGATTCT  CACCCACTCAACAAGGACACCAG
                         (TATA-)                         (INR+)                    (MTE-)                           
    183 Mouse: Rxrg (20183, BC058401) 
Human: RXRG (6258, BI768747) 
 
           |-50      |-40      |-30      |-20      |-10      |1        |11       |21       |31       |41       
Mouse     ATTGGTTCCCAGCT  AATAGAT  TGTGCCACCCGGTAGGAACCTCTGG  GGAC T AGC  GGGGAGCTGG  AAGAGTCGCTCAC  AGCAGCCCAACCCAGAGTTAATC
                         (TATA+)                            (INR-)                 (MTE-)                           
Human     TTGGCTTCCTAGAT  AATAGAT  CGTGCCACCCGGTAGGGACCTCTGGGGAC G CGCCGGGAGCTGGA  AGAGTCGCACGCA  GCAGCCCAACCCTGAGTTAATC
                         (TATA+)                                                  (MTE+)                          
    184 Mouse: Sag (20215, BC016498) 
Human: SAG (6295, M38064) 
 
           |-50      |-40      |-30      |-20      |-10      |1        |11       |21       |31       |41       
Mouse     CCTCACCCTTGCCCTGTGGG  GTTATCT  GATAGGATTGCACCAGGTCCC  TC A GAGCG  TGGGCTGGCTGCTCACTGTCGAAAAGCACAGGTACCCTCTCGTG
                               (TATA-)                        (INR+)                                              
Human     CTCAGCGGTGCCCCT  TCAGGCT  CATCTGGCAAGACGGTACCAGCTTGC  TC A GAACA  GGGGCTGGCTATTCA  TCATCTCAGAGCA  TAGAGACCCTCTCCTT
                          (TATA-)                             (INR+)                      (MTE+)                    
    185 Mouse: Apcs (20219, NM_011318) 
Human: APCS (325, NM_001639) 
 
           |-50      |-40      |-30      |-20      |-10      |1        |11       |21       |31       |41       
Mouse     ATTGTCATCCAAGGTACA  TACAAAA  CCTGAAATCTGAAAAGCATAG  GCAG A CAC  CACACTTTTGTTCCACACCCAAGTAACAGCTGCTGCTGTCATACCC
                             (TATA+)                        (INR+)                                                
Human     AACCCTGAATAACCTGAAG  TCTAAGG  GCATGAATATCAGACGCTAGGGGG   A CAGCCAC  TGTGTTGTCTGCTACCCTCATCCTGGTCACTGCTTCTGCTAT
                              (TATA+)                           (INR+)                                            
    186 Mouse: Apcs (20219, M29535) 
Human: APCS (325, NM_001639) 
 
           |-50      |-40      |-30      |-20      |-10      |1        |11       |21       |31       |41       
Mouse     ATCATTTATTTTCTAACAACAG  CTCTAAT  TATTAGCAGAACGAAGGAG  GA T CTGGG  AGTACCTCACATGGTATT  ACTTCTCTCCACC  CTTCATTGTCATC
                                 (TATA-)                      (INR-)                         (MTE+)                 
Human     TTTCCAGCTCAGGGCTCA  CCACTCCT  TCACCGTAAGCGCCAGGAG  GCAGA C CT  GGAAAATCACTCACATA  TTATTGGTGCTCT  TCCTCCCCCATCCTCAC
                              (BRE+)                       (INR+)                        (MTE+)                     
    187 Mouse: Scg2 (20254, BC014717) 
Human: SCG2 (7857, NM_003469) 
 
           |-50      |-40      |-30      |-20      |-10      |1        |11       |21       |31       |41       
Mouse     AGCGTGGAATGCGGAGTCAGGCGCCCAGGCTCTCTATAAGCCGAG  GAGCT G TC  CGGTGCTGAAACGGCC  CGAGCCCTCACTC  AGCGGCAGAGAGGAGCAT
                                                         (INR-)                       (MTE+)                      
Human     GTGTGGCAGTGCGGAGTCAGGCGCAGCGGCTCCCTATAAGCAGAGGAGCT G TC  CGTGTGCT  GAAACGGCCCGA  GAAGCTCGCCCGG  AGAACGGGGAGGAA
                                                                 (INR-)                   (MTE+)                  
    188 Mouse: Ccl20 (20297, AK079862) 
Human: CCL20 (6364, NM_004591) 
 
           |-50      |-40      |-30      |-20      |-10      |1        |11       |21       |31       |41       
Mouse     TGTACATTCCCAGTATTTTGC  TATAAGA  AGGGCTGGTGCTGGAGCACAGG A   GCACTCGC  AGGGCACTGGGT  ACCCAGCACTGAG  TACATCAACTCCTGGA
                                (TATA+)                          (INR+)                   (MTE-)                    
Human     CCAATATTTGCTATA  AATAGGG  CCATCCCAGGCTGCTGTCAGAATATAAC A   GCACTCCC  AAAGAACTGGGT  ACTCAACACTGAG  CAGATCTGTTCTTTGA
                          (TATA+)                                (INR+)                   (MTE-)                    
    189 Mouse: Sdpr (20324, AK035324) 
Human: SDPR (8436, AK026943) 
 
           |-50      |-40      |-30      |-20      |-10      |1        |11       |21       |31       |41       
Mouse     TCCACTGGGTGGAGCAGGTCC  TTTAAGA  GCAACTGGAATGCAGTTCCCCT G A  TCAGCTTA  GCCAG  TTATTCAAAACCT  CTGCTAGCCCTAGAGAGCAGTG
                                (TATA+)                           (INR+)            (MTE+)                          
Human     CCCACTGGGTGGAGTAGGGCC  TTTAAGA  GCAGCTGGAATGCAGTTCC  CCT G ATCA  GCGTAGCCAGTTGTTGCCTGTCT  GAACCT  CTGCCAGTCCTGGAGA
                                (TATA+)                      (INR+)                          (DPE-)                 
    190 Mouse: Sell (20343, M64440) 
Human: SELL (6402, NM_000655) 
 
           |-50      |-40      |-30      |-20      |-10      |1        |11       |21       |31       |41       
Mouse     AGAGATAGAGAGAGAGAGAGAGAGAAGAGAAAGAGGAAGAATTGGGGGCG G AG  GATGTGAG  GCTGGG  CTGGAGAAATGAA  AGAAAGCAAACCCCACTGGT
                                                                 (INR-)             (MTE-)                        
Human     AAGAGGAAAGA  GAAGTGCA  GGAGGAAGGGGAGGGAAAAGGGGAGGAGGAG G AG  GATGTGAG  ACTGGG  TTAGAGAAATGAA  AGAAAGCAAGGCTTTCTGTT
                       (BRE-)                                      (INR-)             (MTE-)                        
    191 Mouse: Sell (20343, X14772) 
Human: SELL (6402, NM_000655) 
 
           |-50      |-40      |-30      |-20      |-10      |1        |11       |21       |31       |41       
Mouse     CATTCTTCCTGAGCGCA  GCACGCCC  TCCTTGTGCAAGAACTCTGAGCC  CC A GGTGC  AGGAGGCTGAGGCCTG  CAGAGAGACTTGC  AGAGAGACCCAGCAA
                             (BRE+)                           (INR+)                       (MTE-)                   
Human     CAGTCTACCTGCAGCAC  AGCACACT  CCCTTTGGGCAAGGACCTGAGACC  C T TGTGCT  AAGTCAAGAGGC  TCAATGGGCTGCA  GAAGAACTAGAGAAGGAC
                             (BRE-)                            (INR-)                   (MTE-)                      
    192 Mouse: Selp (20344, AF031662) 
Human: SELP (6403, AL022146) 
 
           |-50      |-40      |-30      |-20      |-10      |1        |11       |21       |31       |41       
Mouse     CTAGCTGCCAACACTGTTTC  TGCAGTA  TCAACAGGCCAACTTTAGGGGAA A GGCCGTGATACATTTCTG  GAAAGCGAATAGG  AAGTTGTTTTGTTAGGCA
                               (TATA-)                                                (MTE-)                      
Human     TAAGCTGCCAACA  TTACTCT  T  GCATTA  TCAACATTCTAACTTCATGGGAA G G  GCTGTGGT  GAGTTTCTG  GAATGTGAATAGG  AAGTTGTTTTTCTAAACA
                         (BRE+)  (TATA-)                            (INR-)                (MTE-)                      
    193 Mouse: Selp (20344, M72332) 
Human: SELP (6403, AL022146) 
 
           |-50      |-40      |-30      |-20      |-10      |1        |11       |21       |31       |41       
Mouse     GTTGTTTTGTTAGGCAGCCTGA  CATCAAC  GGGAGGCAGCAAAAGTATAA  G A AGTGTG  GTTTTGG  CTAGAGGGAAGAA  AGCC  AGACGG  ATCAGAGAGGACA
                                 (TATA+)                       (INR-)              (MTE-)         (DPE+)              
Human     GTTGTTTTTCTAAACAGCCTGACACTGAGGGGAGGCAGTGAGACTGTAA  G C AGTCTG  GGTTGGGCAGAAGGCAG  AAAACCAGCAGAG  TCACAGAGGAGAT
                                                             (INR+)                        (MTE+)                 
    194 Mouse: Sema4c (20353, S79463) 
Human: SEMA4C (54910, BQ278955) 
 
           |-50      |-40      |-30      |-20      |-10      |1        |11       |21       |31       |41       
Mouse     GTGCTGGCGGGCG  GGCGCAGT  CTGGAGCGGCGCGGGGGCAGGGGAGGACG G TGGCCGGAGTCAAACGCGAGG  GCAGCGCCCAGGG  ATTGGAGCTGCACGA
                         (BRE-)                                                          (MTE+)                   
Human     GGCTGGCGGGCG  AGCGCGGG  CAGGCGGCGACGCGGGGGCAGGGGTGGACG   G CGGTCAG  AGCCGAACGCGAGGGCGGCGCCCGGGGACTGGAGCTGCGCGC
                        (BRE-)                                  (INR+)                                            
    195 Mouse: St8sia4 (20452, NM_009183) 
Human: ST8SIA4 (7903, NM_175052) 
 
           |-50      |-40      |-30      |-20      |-10      |1        |11       |21       |31       |41       
Mouse     ACGCCCCCGAGCCCAGCTGCAG  AAGAAAC  CGCCACCTCCAATGCACAAGG T G  TCACATTT  GAAAAGAAACC  TGAGCCCCAGGGA  GGCGGCGCAGAGCGAC
                                 (TATA+)                          (INR+)                  (MTE+)                    
Human     ACGCCCCCGAACCCAGCTG  CAGAAGC  TGCCGCCACCTCCAATGCACAAGG   T GTCTCAT  CTGAAAAGAAACC  TGAGCCCCAGGGA  GGCGGCGCGGAGCGAC
                              (TATA+)                           (INR-)                    (MTE+)                    
    196 Mouse: St8sia4 (20452, Y09484) 
Human: ST8SIA4 (7903, NM_175052) 
 
           |-50      |-40      |-30      |-20      |-10      |1        |11       |21       |31       |41       
Mouse     CGGTGATTGGCTGG  CCTTGCT  TATCGTTTCCCTGCTACGGCGAGGAAAGG G   CCAGTGAC  GCCCC  CGAGCCCAGCTGC  AGAAGAAACCGCCACCTCCAATG
                         (TATA-)                                 (INR+)            (MTE+)                           
Human     AGCCTGGTGATT  GGCTTTGC  G  CAGCGTT  TATCTCCTAGCAAGAGGGAAGG G   CCAGTGAC  GCCCCC  GAACCCAGCTGCA  GAAGCTGCCGCCACCTCCAATG
                        (BRE-)    (TATA-)                          (INR+)             (MTE-)                          
    197 Mouse: Vps4b (20479, NM_009190) 
Human: VPS4B (9525, BC009692) 
 
           |-50      |-40      |-30      |-20      |-10      |1        |11       |21       |31       |41       
Mouse     TCATTTGTGGCGAAACGCATCCTGGGTGACTGGTTAGCGCGTGTGCGGAT G AC  GAAGCGGA  AGTGCCCGGGCCAGAGGCG  GGACCA  GAGCCCTAAGGGGC
                                                                 (INR-)                      (DPE-)               
Human     CTGCGCTGTGGCTAGAGAAGGGAGTCTGGTGACTGGGTGCGCGTGCTGAT G A  CGAAATCG  GAAGTGCCC  GGAGCAGAGGCG  G  GACCA  GAACCTAGAGGGC
                                                                (INR+)                (MTE+)    (DPE-)              
    198 Mouse: Sox13 (20668, AB006329) 
Human: SOX13 (9580, CN293459) 
 
           |-50      |-40      |-30      |-20      |-10      |1        |11       |21       |31       |41       
Mouse     CAGAGTGGGTTTATAACTGGGCCATGGGCCTCATTCACTCAGTGT  CCTCT G TC  CCTCCTTCTGCAGGATGTCCATGCAGAGCCCCGTCTCTGTGCAGCTG
                                                         (INR-)                                                 
Human     ACTGCCTCTGGGCAGAAGTTTACGACTGGGGACTCACCTCAGC  TCACTCT G   TCCCTCCCCCAGGATGTCCATGAGGAGCCC  CATCTC  TGCCCAGCTGGCC
                                                       (INR+)                                 (DPE-)              
    199 Mouse: Serpine2 (20720, AK045954) 
Human: SERPINE2 (5270, A03911) 
 
           |-50      |-40      |-30      |-20      |-10      |1        |11       |21       |31       |41       
Mouse     AGGGGCGGGGGCGGCGGCGGC  GGTAAAG  CCCCCGCGCGGCCCGGCCGGCT A GT  GCAGTGGT  TGCACGGG  AGTGCGGGCTGCA  CGCGTCACCGTCACCGCC
                                (TATA+)                            (INR-)               (MTE-)                      
Human     GGGCCCGCGC  GGCGCCGC  C  GATAAAG  CCCCCGCCGCCGCGGCAGCCAGCT T GC  GCTGTGGG  GCTGCCCGGGCTGCGCGG  CGTCTG  CAGGCGCCACCGCTG
                      (BRE-)    (TATA+)                              (INR-)                     (DPE-)                
    200 Mouse: Serpinb8 (20725, AK032659) 
Human: SERPINB8 (5271, NM_002640) 
 
           |-50      |-40      |-30      |-20      |-10      |1        |11       |21       |31       |41       
Mouse     CCTCCCAGATCCCACTGTGGGAAGTGTGAGTCACAGAGTGTTCA  AGTCTG T G  CATTATGTTCTGAGTCACCCGACAGGCGCCTTGCCCCGATCAGGAGGA
                                                        (INR-)                                                  
Human     GCCCACAGACATCCACACAGGCCCAAAGAATCAGGGATTGCACAAGCCAG A   GCAATCGA  ACGGTTCTGAG  TCATCTGCCGGAA  GCCTTGCCCTCAATCAA
                                                               (INR+)                  (MTE+)                     
    201 Mouse: Stat1 (20846, AK039458) 
Human: STAT1 (6772, AK022231) 
 
           |-50      |-40      |-30      |-20      |-10      |1        |11       |21       |31       |41       
Mouse     GAGGCGGAAGGAGGCG  GGACGCCC  TCTAGCCTTTTTTCCTGCCCG  GTACT T TC  GGTTTTCAGCGCCGAGTC  TGTCAAAGCTCCC  TGGAGACCTCCGGGAC
                            (BRE+)                         (INR+)                         (MTE-)                    
Human     GGGCGGAAGGGGGCC  GGGCGCCA  GCGCTGCCTTTTCTCCTGCCGG  GTAGT T TC  GCTTTCCTGCGCAGAGTCTG  CGGAGGGGCTCGG  CTGCACCGGGGGGA
                           (BRE+)                          (INR+)                           (MTE-)                  
    202 Mouse: Stk16 (20872, AK032162) 
Human: STK16 (8576, CR603103) 
 
           |-50      |-40      |-30      |-20      |-10      |1        |11       |21       |31       |41       
Mouse     TTGTTGCTAGGATACAGCGGGCCGTTACTTCGACGCGGACTGAT  GATGTC A T  CCGGGCTTCCGGTCCCTGGC  CCTTCGCACAGGC  CGGAGACCGGTCGGT
                                                        (INR-)                           (MTE+)                   
Human     CGGTTGCTGAGATACCGCGGGCCGTTACGCCGGCGCGGACTGATGATGTC A   GCACTGCT  TCCGGT  CGGTGGCGCTTCT  CTCTGGCCCGAGCCAGGTCAGT
                                                               (INR-)             (MTE-)                          
    203 Mouse: Syt2 (20980, AB036514) 
Human: SYT2 (127833, AK094430) 
 
           |-50      |-40      |-30      |-20      |-10      |1        |11       |21       |31       |41       
Mouse     CTCCGCCCG  GGCGCAGA  CTGCTAAGCTCAGCAAGTCTTGCGGCAAAG  GCA C TTCG  GAGAGGAGTTGGACCCGGGGCGGAGTGGGGACTCCGCGCCCCCAG
                     (BRE-)                                  (INR+)                                               
Human     TGCCGCCTC  GGCGCAGA  CTGCTAAGCTCAGCAAGTCTTGCGGCAAAG  GCA C TTCG  GAGCCGAGTTGGG  GCAGCGGTGGAGC  GGGGACTTCGCGCCCCCAG
                     (BRE-)                                  (INR+)                    (MTE+)                       
    204 Mouse: Tcea1 (21399, NM_011541) 
Human: TCEA1 (6917, AL702853) 
 
           |-50      |-40      |-30      |-20      |-10      |1        |11       |21       |31       |41       
Mouse     GCACGGCCTAGGGGGCG  GAGCGCTT  ATCCCTGCCGCCGCGGGCCG  GGTCT G TG  AGGAAGGCCTAGG  CCAGCGGCTTCGC  GGCTTGTCCAACGTCCGCGCA
                             (BRE+)                        (INR-)                    (MTE+)                         
Human     AGGAGGAGC  CGGAGCCG  CCGCCGCCGCCGCCGCCGCCGCGGGCT  TCGTTC G T  AAGGAAGGGGGCCTAGGCCCG  GGCCTGCGGTGGT  GGGGGTTGCTGCGC
                     (BRE+)                               (INR+)                            (MTE-)                  
    205 Mouse: Tcfap2b (21419, AK017373) 
Human: TFAP2B (7021, AJ278356) 
 
           |-50      |-40      |-30      |-20      |-10      |1        |11       |21       |31       |41       
Mouse     TGTGTGTGTGTGTGTGTGTGTGTGTGTGTGTGTGTGTATGCGTGCGCGTT G   TATCTAAG  TCCTGTGTGCAATAATGTTTTTC  TGTCTG  CTGAGGCTTACT
                                                               (INR-)                          (DPE-)             
Human     TGGGTGTAAATACGGGTTTA  TGATTTT  TATAGTATATATGTCTGTGGGTT G   CATCTAAC  TCCTG  TGTGTGCAATAAT  GTTTTTTCTCTTTGCTGAGGCTT
                               (TATA-)                           (INR-)            (MTE-)                           
    206 Mouse: Terf1 (21749, AK036615) 
Human: TERF1 (7013, BM311009) 
 
           |-50      |-40      |-30      |-20      |-10      |1        |11       |21       |31       |41       
Mouse     GCGATTGGCTGACTT  CCACGCCC  ACCGCGAGCAAATCAGCGCACGGCG  CC A GCTGA  GGCACGGCGAGC  GCTTTCGGTTTAA  CATGGCGGAGACGGTCTCC
                           (BRE+)                             (INR+)                   (MTE-)                       
Human     CGCGGCCACGCCCCGA  GCCCTCGA  ATGCGAGCCAATCGTTGCTCGG  CGCC T GAA  GGGGCAGTACC  CAAGCGAGCCATT  TAACATGGCGGAGGATGTTTCC
                            (BRE-)                          (INR-)                  (MTE+)                          
    207 Mouse: Tgfb2 (21808, NM_009367) 
Human: TGFB2 (7042, AY438979) 
 
           |-50      |-40      |-30      |-20      |-10      |1        |11       |21       |31       |41       
Mouse     GCACGCGCGCACACGCGCACACATCCACACGCACACTCATCCACACA  CGT G TGGA  AGGCAGGGC  CGAGCCGCTCGGT  CTTT  GAACTT  CTCAGTTAGAGCC
                                                           (INR-)                (MTE+)         (DPE-)              
Human     GCACACACACACACACACACACACACGCACGCACACACGTGTGCGCT  TCT C TGCT  CCGGAGCTGCTGC  TGCTCCTGCTCTC  AGCGCCGCAGTGGAAGGCA
                                                           (INR-)                    (MTE-)                       
    208 Mouse: Dedd (21945, AK088329) 
Human: DEDD (9191, AK022531) 
 
           |-50      |-40      |-30      |-20      |-10      |1        |11       |21       |31       |41       
Mouse     CTGGAGCCCGGATGCGGCTCCGTGAGGCGCAGGCCCCGGAGAGCGGCGCG G ATGGATCCAACATGGCGG  CGCCGAGGCTGAG  CCGAGAGTGAGGCGGCGG
                                                                                    (MTE-)                      
Human     TGCTGGAGCCCGGATGC  GGCGCCGT  GAGGCAGGCCCGGGAGAGCGGCGCG G ATGGATCCAACATGGCGGCGC  CGAGCCTGAGCCG  AGAGTGAGGCGGAGG
                             (BRE-)                                                      (MTE+)                   
    209 Mouse: Tnni1 (21952, S66110) 
Human: TNNI1 (7135, CR741181) 
 
           |-50      |-40      |-30      |-20      |-10      |1        |11       |21       |31       |41       
Mouse     GTTGCCGGAGGCCA  CGGTTTT  CATAGCCTGCCCTCAGCTCTGCCCCC  ACA C TCTG  CAGTCTGTGGTGAGGCTCAGGCCAGCCTAGCTCCACGAGGACTAA
                         (TATA-)                             (INR+)                                               
Human     TTGCTGCTGGACACAGTT  TTCATAG  CCTCCCCTCGGCTCTGCCCCTC  ACA G TCTG  CAGTCTACGGCGAGGCACAGGCCAGCCCAGCTCCACGAGGACTGA
                             (TATA+)                         (INR+)                                               
    210 Mouse: Tnnt2 (21956, M80829) 
Human: TNNT2 (7139, N85494) 
 
           |-50      |-40      |-30      |-20      |-10      |1        |11       |21       |31       |41       
Mouse     CCCCAGCCC  ACATGCCT  G  CTTAAAG  CTCTCCCCATGCCCTGCCCTCAG  CC A GTCCC  TGCTGAGG  CTGAACAGATACC  TCA  AGACCT  GTGTGCAGTCCCTG
                     (BRE+)    (TATA+)                          (INR+)               (MTE-)        (DPE+)               
Human     GCCCCAGCCC  ACATGCCT  G  CTTAAAG  CCCTCTCCATCCTCTGCCTCAC  CC A GTCCC  CGCTGAGAC  TGAGCAGACGCCT  CCAG  GATCTG  TCGGCAGCTGCT
                      (BRE+)    (TATA+)                         (INR+)                (MTE+)         (DPE-)             
    211 Mouse: Tnp1 (21958, BC048494) 
Human: TNP1 (7141, BC029516) 
 
           |-50      |-40      |-30      |-20      |-10      |1        |11       |21       |31       |41       
Mouse     TGTGCTCACAATGGCT  AAGGCCT  TAAATACATGGACTCCCGGCCCTGCCG G CCTTGCAAAGCCCC  TCATTTCGGCAGA  AAGTACCATGTCGACCAGCCGC
                           (TATA-)                                                (MTE+)                          
Human     TCCCTGTCACAATGGCC  AAGGCCT  TAAATACCCAGACTCCTGGCCCCCGG G CCTTGCAAAGCCCCTCATTTTGGCA  GAACTT  ACCATGTCGACCAGCCGC
                            (TATA-)                                                      (DPE-)                   
    212 Mouse: Tpp2 (22019, NM_009418) 
Human: TPP2 (7174, NM_003291) 
 
           |-50      |-40      |-30      |-20      |-10      |1        |11       |21       |31       |41       
Mouse     GTACTGGTGGCCA  GGCTCCGC  CCCTTCCCCACCTCCGTCCCGCGGTAGCC G   GGTGTCCT  CGCGCCGCTCG  TCCGCGCGCTGCC  TGGCAGTTTGCCTCTTC
                         (BRE-)                                  (INR-)                  (MTE-)                     
Human     AAGACGGGCCGACCCCGCCCCTCGCCCCCGCCTCCGGCCCGAGGCCGCAC G   GGTGTCCT  CGCGCTG  CTAGTCCGCGCGC  AGCCTGGCAGTTTGCCGCTTC
                                                               (INR-)              (MTE+)                         
    213 Mouse: Tsn (22099, BC004615) 
Human: TSN (7247, NM_004622) 
 
           |-50      |-40      |-30      |-20      |-10      |1        |11       |21       |31       |41       
Mouse     GTGCGCCGCGGGTGTGGGAAGACCCCGGCGCCAGCCTTGGCGCAAGGG  CT A GTGGA  CGCGGCAGTGGCGACGGC  GACGGGCGTTGCG  AGCGAGTCCGCTA
                                                            (INR+)                         (MTE-)                 
Human     GCGCGCCGCGAGCCTCGGAGGACCCTAGCGACGGTCGTGGCGTA  AGACCG G G  GGGACGCGGCGGTAGCGGCGG  CCGTTGCGATTGA  TTGCGCTGGTTGCC
                                                        (INR-)                            (MTE-)                  
    214 Mouse: Tuba4 (22145, AK002427) 
Human: TUBA1 (7277, BQ894018) 
 
           |-50      |-40      |-30      |-20      |-10      |1        |11       |21       |31       |41       
Mouse     GCGCAGGTCCCGCCTCCTGCC  TATAAGA  GCGGTGCGGCACTGCAGCTCC  G C AGATCT  CACCGAGACCCGTGACCC  CGACTC  TACGTGAGACGTACAGCCC
                                (TATA+)                        (INR+)                     (DPE-)                    
Human     GGCCCCGCCCCCCCCCCGCC  TATAAGG  GCGGTGCGGCACTGCAGCTAGC  G C AGTTCT  CACTGAGACCTGTCACCC  CGACTC  AACGTGAGACGCACCGCCC
                               (TATA+)                         (INR+)                     (DPE-)                    
    215 Mouse: Tnfsf4 (22164, NM_009452) 
Human: TNFSF4 (7292, AB042987) 
 
           |-50      |-40      |-30      |-20      |-10      |1        |11       |21       |31       |41       
Mouse     TTCAGATTAGTCACA  TAGAAGT  TCCCCCGCCTGCAAAACCTGC  AGAGTTA A   AACGAAGAGACTCTGCTCTG  CTCCTTCAATTGC  TTTTTGTCTCCTGTTC
                          (TATA+)                        (INR-)                           (MTE-)                    
Human     GATTAGTCACAAAGAAGTTCCCCCGCCTGCCTGCAAAAGTTGCAGCGTTA   A AACTGAG  AGAGTCCGCTTTG  CTCTTTCAATCGC  CTTTTATCTCTGGCCC
                                                              (INR-)                    (MTE-)                    
    216 Mouse: Sumo1 (22218, BC082566) 
Human: SUMO1 (7341, BX460163) 
 
           |-50      |-40      |-30      |-20      |-10      |1        |11       |21       |31       |41       
Mouse     CTCGCCTCTGCGCGCAGCGGAAGTGACGCAAGACGTAGAGGAAGTCCCTG C AGCCGCGGTGTTGTG  CTGTAGAGAAGGG  ACGGATTTGTAAACCTCAGAG
                                                                                 (MTE-)                         
Human     CTCCTCCCTGCGCGAAGCGGAAGTGACGCGAGGCGTAGCGGAAG  TTACTG C A  GCCGCGGTGTTGTGCTGTGGG  GAAGGGAGAAGGA  TTTGTAAACCCCGG
                                                        (INR+)                            (MTE-)                  
    217 Mouse: Usf1 (22278, BC049784) 
Human: USF1 (7391, AY593992) 
 
           |-50      |-40      |-30      |-20      |-10      |1        |11       |21       |31       |41       
Mouse     CTCACCAACATGGCCGCAGGCTGGGACTGCGCATGAGCAGCTGTCTATGG   A GAGACCT  AGATCCAAAGAC  GGAGAAGGCTGCC  GGGGGAAATCGGCGACT
                                                              (INR+)                   (MTE-)                     
Human     CTCACCAACATGGCCGCGGGCTGGAAGTGCGCATGAGCAGCTGTCTAT  GG A GATAC  CTAGGCCGGG  AGAGGGAGAACAC    AGTTGG  AGAAAATCGGCAGCT
                                                            (INR+)                 (MTE-)     (DPE+)                
    218 Mouse: Ush2a (22283, AK020845) 
Human: USH2A (7399, AF091873) 
 
           |-50      |-40      |-30      |-20      |-10      |1        |11       |21       |31       |41       
Mouse     GACTCCCTCCCACTGTACCAGC  TTGAAAG  CCTTGAAAGCCAGGACACC  TC A GTTGC  TGAGGGGCCACCGA  ACAGCCTACACTG  AGCTACAGGTAACCCTT
                                 (TATA+)                      (INR+)                     (MTE+)                     
Human     TCCAGGTCCCTCCCACTGCTTGAGGTACCAGCGGAAAGCTTGGGCAGC  TC A GTTCC  AAGAGGGCCACCAAGC  AGACCA  CGCTCTGAGCTTCAGGTAACCA
                                                            (INR+)                   (DPE+)                       
    219 Mouse: Vil1 (22349, AK027908) 
Human: VIL1 (7429, A07400) 
 
           |-50      |-40      |-30      |-20      |-10      |1        |11       |21       |31       |41       
Mouse     GGGGTGGTGGTGAG  GACAAAG  GTCGTTCGGTCTCCTGCAGCCAGCTTGCC A   CAACTTCC  TAAGATCTCCCA  GGTGGTGGCTGCC  TCTTCCAGACAGGTAA
                         (TATA+)                                 (INR-)                   (MTE-)                    
Human     TAGAGGAGGGGGGGTGGG  GACAAAG  GTCGCTCTTCTGCAGCCAGCTTGCC   A CAATTCC  CTGAGATCTCCCA  GGTGGCAGCTGCC  TCCCCAAGACAGGTAA
                             (TATA+)                            (INR+)                    (MTE-)                    
    220 Mouse: Wnt6 (22420, NM_009526) 
Human: WNT6 (7475, AY009401) 
 
           |-50      |-40      |-30      |-20      |-10      |1        |11       |21       |31       |41       
Mouse     NNNNNNNNNNNNNNNNNNNNNNNNNNNNNNNNNNNNNNNNNNNNNNN  NNT C TCTC  CAGCGAGCTCCGCAGGAGA  CACAGGCGCTGGC  TGCTGGGTCCGCT
                                                           (INR-)                          (MTE-)                 
Human     CCGCCTCGGCCCCGCTCCCCTCCCACCTCCGCCCCCGGCTCTGATTTCTT C TCCCGAGCGAGCTCCGCAGGAGA  CACAGGCGCTGGC  TGCCCCGTCCGCT
                                                                                         (MTE-)                 
    221 Mouse: Ercc5 (22592, U40796) 
Human: ERCC5 (2073, AF255432) 
 
           |-50      |-40      |-30      |-20      |-10      |1        |11       |21       |31       |41       
Mouse     CTTTTAGGCGTGCCAGTACTGGGGCTGAAACCTTTCTGCTCCTCCCA  CCA C ATTT  AAATTCCGCTGTTCTTTCGGG  AGACCG  GGTCCGCTCCGGAAGTGC
                                                           (INR+)                        (DPE+)                   
Human     GGCCTAAGCGCTGGGCGA  GGCGAGGC  CCTGCCCCTCCCCGCCAACGG  CCA T TCTC  TGGACCTGTCTT  TCTTCCGGGAGGC  GGTGACAGCTGCTGAGACGT
                              (BRE-)                         (INR+)                   (MTE+)                        
    222 Mouse: Xrcc5 (22596, AK081633) 
Human: XRCC5 (7520, J04977) 
 
           |-50      |-40      |-30      |-20      |-10      |1        |11       |21       |31       |41       
Mouse     TGAGTTGCGACACTCTAGGTTTCCGCCCGGAAAAAGTAATCAAAT  CACCT G AG  GACCAGCATGGCGTGGTCCGGTAATAAGGTACCGAGAGCAGGGCCTT
                                                         (INR-)                                                 
Human     CGAGTTGCGACACGGCAGGTTCCCGCCCGGAAGAAGCGACCAAAG  CGCCT G AG  GACCGGCAACATGGTGC  GGTCGGGGAATAA  GGTATAAAGAAAGCCAT
                                                         (INR-)                        (MTE-)                     
    223 Mouse: Slc30a1 (22782, BC052166) 
Human: SLC30A1 (7779, NM_021194) 
 
           |-50      |-40      |-30      |-20      |-10      |1        |11       |21       |31       |41       
Mouse     GGTTCCGAGTGCAAAGCAAA  GTTTTTT  TGTAAACCGTCTGCAAAGGAGGG G GGGCGAAGACGGCGGACGCCTCCGGGCCGAGCGCAGTCGCCGCGGACGC
                               (TATA-)                                                                          
Human     GGTTCCGAGTGCAAAGCAAA  GTTTTTT  TGTAAACCGTCTGCAAAGGCGGG G GGGCGAAGAAGGCGCCCGAGACCGGGCCGAGTGCAGCTGCCGTGGCCGC
                               (TATA-)                                                                          
    224 Mouse: Adam23 (23792, AK046677) 
Human: ADAM23 (8745, BE246241) 
 
           |-50      |-40      |-30      |-20      |-10      |1        |11       |21       |31       |41       
Mouse     CCGCGCGTGCCCGTGCCGCTCGCTCCTGCGCGCGCCTCCTCAGCATCC  TT A GGCCC  GGCAGCAACCCCC  GCAGTCACTGGTG  CGGCCGCGCCCGCCACTG
                                                            (INR+)                    (MTE+)                      
Human     TCCCGCGCGTCCCCGCGCCGCTCGCTCCCGCGCGCCGCCTCAGCATCC  TC A GGCCC  GGCGGCAGCCCC  CGCAGTCGCTGAA  GCGGCCGCGCCCGCCGGGG
                                                            (INR+)                   (MTE-)                       
    225 Mouse: Capn10 (23830, AF203031) 
Human: CAPN10 (11132, AF158748) 
 
           |-50      |-40      |-30      |-20      |-10      |1        |11       |21       |31       |41       
Mouse     TCAGAAGACAGGGAGGAACCACCGCTTTGCCAAGTTCCTGCCTCACACCG G AAGGAGCGAAGCACGCTGGGAAGTAGACAACGCCTGTGATCAGCGACGT
                                                                                                              
Human     CTCGGTTCCTCCCAACTACCCGCGGCCACGGCCTCCGCAGCAGAGCGCCG G AAGCAGAGACGCGTTTCGGGAGGAAGGTGCATGCTGGGAGCGGCGGCGC
                                                                                                              
    226 Mouse: Capn10 (23830, AF089089) 
Human: CAPN10 (11132, AF158748) 
 
           |-50      |-40      |-30      |-20      |-10      |1        |11       |21       |31       |41       
Mouse     AGCCGGGTTACCTAGACAACGGAGCTTGCGGCGCTAGGCTGCTCTGCA  CC A GTAGG  TCTCCCGGGCTAAGC  AAACACGGTTTGC  AGTGAAGGCCGCGCAC
                                                            (INR+)                      (MTE-)                    
Human     GGTTACCAAT  GGGAGACT  AGCGGGCCGGCGTACTGGCCTGGTCCAG  CACC T GCG  GGGCCCTCGGGC  TTGGAGGGCTGGG  CCGGGCGGGGAACGGGCGGGG
                      (BRE+)                                (INR-)                   (MTE-)                         
    227 Mouse: Cdh20 (23836, AF007116) 
Human: CDH20 (28316, NM_031891) 
 
           |-50      |-40      |-30      |-20      |-10      |1        |11       |21       |31       |41       
Mouse     AAAGCTGTGACACTTTTGACTTGGGGGGAAGGGGGTGGGGCGGGG  GCACA G AG  GCGAGCTGAGG  CGAAGGGACTCGG  CA  GGACAC  CCGTTCGCCCAATGA
                                                         (INR+)                  (MTE-)       (DPE+)                
Human     AAGCTGTG  ACACTTCT  GACAGAGGGGGTAGGGGGGTGGGGGGCGG  GGACA G CG  CCGCGAGCAGGG  GTGAAGAGACCCA  GCGGGGCACCAGCCGCCCAGTG
                    (BRE+)                                 (INR-)                   (MTE-)                          
    228 Mouse: Farslb (23874, AK010271) 
Human: FARSLB (10056, NM_005687) 
 
           |-50      |-40      |-30      |-20      |-10      |1        |11       |21       |31       |41       
Mouse     GTCGGGTCCT  TCACTCTA  GGGTCGCTGAGGCCCCGCATGTGGCGCAGGC  G C AGTGTC  CACGACTCGCCATGC  CGACTG  TCAGCGTAAAGCGAGACCTGCT
                      (BRE+)                                   (INR+)                  (DPE-)                       
Human     GGTCCTACGCG  CTTCGCTA  GGGAAGCCCGGGTCAGCTCGCTGCGCAGGC  G C AGTGAG  TTCGACACACCATGC  CGACTG  TCAGCGTGAAGCGTGATCTGCT
                       (BRE+)                                  (INR+)                  (DPE-)                       
    229 Mouse: Grem2 (23893, NM_011825) 
Human: GREM2 (64388, BP231127) 
 
           |-50      |-40      |-30      |-20      |-10      |1        |11       |21       |31       |41       
Mouse     ACTGGGGTGTGTGTGTGTGTGTG  AAGTATT  TGTGGGTTAGATGA  GAAGCG A G  GGCTGAGTGAAGCCCGAGAAGGAGGGG  TGACTG  GTGTGATTTGTTTGG
                                  (TATA-)                 (INR-)                              (DPE-)                
Human     GACTGAAGTGTGT  GGTGTGGC  TG  CAATGTT  TGTGGGTTAGATGAG  AAACA A GC  GCTGAGTGAAACAGAGAAG  GGGGGTGGCTGGT  GTGATTTGTTTGGTG
                         (BRE-)     (TATA-)                  (INR-)                          (MTE-)                   
    230 Mouse: Neu2 (23956, AK009828) 
Human: NEU2 (4759, NM_005383) 
 
           |-50      |-40      |-30      |-20      |-10      |1        |11       |21       |31       |41       
Mouse     CTAGGGCTCTGGAGT  TATAAAA  AGGCTCTCCCATGAGTCATCCCCTG  GCA G TCGG  ATAGCTGCAG  AGAGCCACTCAGC  AAGCTCTTCTACTCTACCCAGG
                          (TATA+)                            (INR+)                 (MTE+)                          
Human     TTTCTAGGGCTCTGGAGT  TATAAAA  AGCTCCCATGTGTCACTTTCTGG  CA T TTGGG  CAGCTGCCGAATG  CCACCCGGCCTGC  ACTGCTGTCCTCCCGAGG
                             (TATA+)                          (INR-)                    (MTE+)                      
    231 Mouse: Rgs7 (24012, BC051133) 
Human: RGS7 (6000, NM_002924) 
 
           |-50      |-40      |-30      |-20      |-10      |1        |11       |21       |31       |41       
Mouse     GCCCGGGGGAGCGGGCGCGAGCGGCGGCTGCCGGGGCCGCCTGTGCACCG C GGCGAGGGTGGCGGTGGCCTGA  GCAGCGCGAGTGT  GATGAGGCTGCCCT
                                                                                        (MTE+)                  
Human     AGCGGGCGCGAGTGGCGGCTGCCGGAGCCGCCTGTGCACGGGCGGCGGCG A GGCGAGGGTGGCGGTCGC  CTGAGCAGCTAGA  GCCGGGGCGGCGCGGAAT
                                                                                    (MTE-)                      
    232 Mouse: Avpr1b (26361, AF098866) 
Human: AVPR1B (553, NM_000707) 
 
           |-50      |-40      |-30      |-20      |-10      |1        |11       |21       |31       |41       
Mouse     ACCGTCCCTCCATCCACCTTCCCCTCCTTCTCTCCCTCTAAACCTAACCA T GG  ATTCTGAG  CCTTC  TTGGACTGCTACC  CCCTCCCCTGGGGGCACCCTG
                                                                 (INR-)            (MTE-)                         
Human     CTTGCATCCACACC  CTCCCTT  CATCCTTCCCTCCCAGCAAACCTTGCTCA T GG  ATTCTGGG  CCTCTGTGGGATGC  CAACCC  CACCCCTCGGGGCACCCTC
                         (TATA-)                                   (INR-)                 (DPE-)                    
    233 Mouse: Nr5a2 (26424, AF239709) 
Human: NR5A2 (2494, AF319643) 
 
           |-50      |-40      |-30      |-20      |-10      |1        |11       |21       |31       |41       
Mouse     CCTATTGAA  AGCTTCCT  TTCTTCTGATTAGAAACCATCATGAAACTGG  AG A CATGG  TTTACAGCAGGTCATACATGCTGGAAAAAGTGCAGAGTCCAGGA
                     (BRE-)                                   (INR+)                                              
Human     CCTATCAAAAGTTT  CCATTCTT  CTGATTAGAAACCATCATGAAACTGG  AT A CATGG  TTTACAGCA  GGTCACTAATGTT  GGAAAAAGTACAGAGTCCAGGG
                          (BRE+)                              (INR+)                (MTE-)                          
    234 Mouse: Cul3 (26554, AF129738) 
Human: CUL3 (8452, U58089) 
 
           |-50      |-40      |-30      |-20      |-10      |1        |11       |21       |31       |41       
Mouse     ACAAATACAAAATTATTTTCAACTTGTTCTCCCTTTTCTCTCTTGGATAT T   ACAGATGA  CCATG  GATGAAAAATATG  TAAACAGCATTTGGGACCTTCTG
                                                               (INR+)            (MTE-)                           
Human     CATTGGTTTTTTTTTTTTTT  TTTTTTT  TGTCTCTTCTCTCTCTTGGATAT T   ACAGATGA  CCATG  GATGAAAAATATG  TAAACAGCATTTGGGACCTTCTG
                               (TATA-)                           (INR+)            (MTE-)                           
    235 Mouse: Cops5 (26754, AK012499) 
Human: COPS5 (10987, BX648542) 
 
           |-50      |-40      |-30      |-20      |-10      |1        |11       |21       |31       |41       
Mouse     GCCGCCGCCGCGCCCCAGCGCGCGAATGGTCTGGACCAACGTCACCTCC  G G TCTCAA  GTGTCATGGCTGCCTTG  AGAGTCTAGTGAG  TGTGCACCCTGGT
                                                             (INR-)                        (MTE+)                 
Human     CCGGAAAGGTCCCTGTCTTCCCTGAATGGTCTCTACCAACTTCACCT  CCG G TTCT  AGGTGTCATGGCTGCCCCA  AGAGTCTAGGTAA  GAGTTTGTTCCCG
                                                           (INR+)                          (MTE+)                 
    236 Mouse: B3galt2 (26878, AK036141) 
Human: B3GALT2 (8707, BC022507) 
 
           |-50      |-40      |-30      |-20      |-10      |1        |11       |21       |31       |41       
Mouse     TTAGTCAAAAT  GAACGCAA  ACAATCACGTGGGACTCAGCCAAGCAGTGAC G   TTAAATTC  TGCTCTGTCAGAGACAG  CATCTG  TCAAGCCCACATTTAAAC
                       (BRE+)                                    (INR+)                    (DPE-)                   
Human     TTAGTCAAAAT  GAATGCAA  ACAATCACGTGGGATTCAGCCAAGCAGTGAC G   TTAAATTC  TGCTCTGTCAGAGAGAG  CATCTG  TCAAGCCCACATTTAAAC
                       (BRE+)                                    (INR+)                    (DPE-)                   
    237 Mouse: Cops7b (26895, AK081309) 
Human: COPS7B (64708, AK024273) 
 
           |-50      |-40      |-30      |-20      |-10      |1        |11       |21       |31       |41       
Mouse     CTTCCGGGGGAACAGCACCGGTGACGGGTCGGCGGAGACAGAAAAGTGCT G GACGCCGGGCCGAT  CATGGAAGCTTGA    CAACCT  GCAGGCAGGCTCTGGG
                                                                                (MTE-)     (DPE-)                 
Human     CTTCCGGGGGAGCTGCACGGGCGACGGGTCGGCGGAGACAGAAAAGCG  CC G GACGC  CGGGGTGAT  CATGGACGCTTGA    CAACCT  GCGGGCAGGCGCCGGG
                                                            (INR+)                (MTE-)     (DPE-)                 
    238 Mouse: Exo1 (26909, AK028728) 
Human: EXO1 (9156, BX495976) 
 
           |-50      |-40      |-30      |-20      |-10      |1        |11       |21       |31       |41       
Mouse     AGGACGCCCAG  GGACGTCA  TGTCCGCTGGGCGGGGCTTCCCGATCCCGC  G C AAATCG  AAAGGTCCA  TGGCTGCGCTTGC  AGCAGCCGCTCGCCGCCGTTC
                       (BRE+)                                  (INR+)                (MTE-)                         
Human     GGATACGCGT  TGACGTCA  CATCCTCTGGGCGGGGCCGCAAGGAACCCGCG C   AAATTGAA  AGGTCAGCCT  TTCGCGCGCTGTG  TAGGCAAGTTACCCGTGT
                      (BRE+)                                     (INR-)                 (MTE-)                      
    239 Mouse: Exo1 (26909, AJ238213) 
Human: EXO1 (9156, BX495976) 
 
           |-50      |-40      |-30      |-20      |-10      |1        |11       |21       |31       |41       
Mouse     CAGCCTTGGTTTCATGTT  GCTCTCCT  CCCTTTTTCCTTCATGTAGT  TAAC T TGG  CACGATGGGGA  TTCAAGGGTTACT  TC  AGTTCA  TCCAAGAAGCTTCT
                              (BRE+)                        (INR-)                  (MTE-)       (DPE+)               
Human     TCTTTAAATATTACTGTTCTCCCTGTCTCTTTTCATATCAGGTAGTT  AAT T TGGC  ACCATGGGG  ATACAGGGATTGC  TACAATTTATCAAAGAAGCTTCA
                                                           (INR-)                (MTE-)                           
    240 Mouse: Eif4e2 (26987, AK032965) 
Human: EIF4E2 (9470, BI826265) 
 
           |-50      |-40      |-30      |-20      |-10      |1        |11       |21       |31       |41       
Mouse     CTGACGTTCCGCT  GAGCGCCT  CGCCAGCCAACCGGAAGTAGCCGGGCCCT T AGCGATGGGACCCGGTAGAGCGGA  AGTCAC  TCCGTGAGGCAGTGGCGAC
                         (BRE+)                                                         (DPE+)                    
Human     GCGCCGACGTTCCG  CTGCGCCC  CGCGCAAAACCGGAAGTACCCGGGCCCA   A GGCTGAG  GGACCCGGTGGAGCGGA  AGTCAC  TCCCTGAGGCAGTGGCGAC
                          (BRE+)                                (INR-)                    (DPE+)                    
    241 Mouse: Nit1 (27045, AK032189) 
Human: NIT1 (4817, NM_005600) 
 
           |-50      |-40      |-30      |-20      |-10      |1        |11       |21       |31       |41       
Mouse     GGCCTGCGCACTCTCA  CTGCGTCA  CATCCGGCCGGGGGGTTACCGC  CCAC T TGC  TCCTGCGCTCCTGGCTCCTG  GCAGCTCTTGAGA  TCCGACCCCGTGA
                            (BRE+)                          (INR+)                           (MTE+)                 
Human     CAGCTCAGCCCCCGCCT  TGCGTCAC  GTCCGGCCTGCGAGTTACCGC  CCAC T CGC  TGCGGCGCTTCTGGCTCC  AGACCG  CCCTCCGGATCGGACCCTGCGA
                             (BRE-)                         (INR+)                     (DPE+)                       
    242 Mouse: Srp9 (27058, BC039648) 
Human: SRP9 (6726, BG565815) 
 
           |-50      |-40      |-30      |-20      |-10      |1        |11       |21       |31       |41       
Mouse     ACAGGCGCGCGGCCGAGGCTGGCCAATGGCGTGAGCGCTCGTGAGCCTCC G TCTTTGCGCCGCCGGAGT  GCAGCGCTGTGGG  CGGTTGCGGGAGAGCGGC
                                                                                    (MTE+)                      
Human     TGGACGGGGCTGTCCAATGGAGCGAGGCGTTGGTGCGAGGAGGCGCCGC  C A TCTTGG  GGCTGCTGGGACT  CGCGTCGGTTGG  C  GACTC  CCGGACGTAGGT
                                                             (INR-)                    (MTE-)    (DPE-)             
    243 Mouse: Slamf1 (27218, AK037780) 
Human: SLAMF1 (6504, NM_003037) 
 
           |-50      |-40      |-30      |-20      |-10      |1        |11       |21       |31       |41       
Mouse     TGGGGGTAACAGTAAGCAGCTGTCCTGCCGAGCTGAGCTGAGCTGAGC  TC A CAGCT  GGGGACCC  TGTCTGCGATTGC  TGGCTAATGGATCCCAAAGGATC
                                                            (INR+)               (MTE-)                           
Human     ATGACACGA  AGCTTGCT  TCTGCCTGGCATCTGTGAGCAGCTGCCAGGCTC C GG  CCAGGATC  CCTTCCTTCTCC  TCATTGGCTGATG  GATCCCAAGGGGCT
                     (BRE-)                                        (INR+)                   (MTE+)                  
    244 Mouse: Phlda3 (27280, AF151099) 
Human: PHLDA3 (23612, CN356697) 
 
           |-50      |-40      |-30      |-20      |-10      |1        |11       |21       |31       |41       
Mouse     CGGGGCAGGAA  TGCGCGGC  GGGCGGCGCAGGAGGCGAGCGGTGGAA  CATG T AAG  GGCACATCCCGCCAGCAG  CCGCCCAGCTCGC  AGACGGAGCGCAGGG
                       (BRE-)                               (INR-)                         (MTE-)                   
Human     GTAGGAATGCGCTGCGGGCGGGCGGCGCAGGAGGCGAGCGGCGGAA  CATG T AAG  GGCACATCCCGCGA  GCTGCCGCCCAGC  GCGCAGACAGAGCCCAGGG
                                                          (INR-)                     (MTE+)                       
    245 Mouse: D1Ertd251e (27878, BC027337) 
Human: STAF42 (117143, BC036497) 
 
           |-50      |-40      |-30      |-20      |-10      |1        |11       |21       |31       |41       
Mouse     AGCCGAACCGAGCCGAGCCGAGCCGAGTGGAATCGAGTCGAGTCGAGCCT C CAGCGTCCGGCGCGCAGGCCTT  CCGCCGCGTTGAT  CTTTCGGTTGCTGG
                                                                                        (MTE-)                  
Human     CCAAACAAA  CGACTTCT  GAGAGATTGGGGGCGGGACTGACGGCGGCCGGC T TAGCTTCCAGAGCCAAGGCCTTCCGCCG  AGTTGG  TTTTTGGGTTGTTGA
                     (BRE+)                                                                 (DPE+)                
    246 Mouse: Imp4 (27993, AK008178) 
Human: IMP4 (92856, AF054996) 
 
           |-50      |-40      |-30      |-20      |-10      |1        |11       |21       |31       |41       
Mouse     AATCGAGTGAAGCC  TCTAAAG  GCCTTTTTGGTTGAGGGCGGAAGTGA  TCA C TGCG  TGGTGCCAGCAGCGCCAGAAGAGTTG  GGACTC  GTGAGGGAGCGGC
                         (TATA+)                             (INR+)                             (DPE-)              
Human     CACCAGTGA  AGCGTCTA  AGGGACCATCTTGGTTGAGGGCGGAAGTGA  GGA C TGTG  TCGCGCTGGAGGA  GCCCACAGATTCT  CCCGGACCCACGTGGAAGC
                     (BRE-)                                  (INR-)                    (MTE-)                       
    247 Mouse: Imp4 (27993, AF334609) 
Human: IMP4 (92856, NM_033416) 
 
           |-50      |-40      |-30      |-20      |-10      |1        |11       |21       |31       |41       
Mouse     TTGTGGTCCTCGGTGCTCCGAAGTGGATGGTGACCCTGTGTGT  TCTCTAG C   TCCGTCGGGAGGCTCGCCTGCGCCGCGAGTACCTGTACCGCAAAGCCCG
                                                       (INR-)                                                   
Human     TCCGGGGCTCGGGAGCGAGTAGTGAGTGACTGGGCCTGCTGTTC  CCACAG C T  GCGCCGCGAGGCCCGCCTGCGCCGCGAGTACCTGTACCGCAAGGCCCG
                                                        (INR+)                                                  
    248 Mouse: Rabgap1l (29809, AK034019) 
Human: RABGAP1L (9910, AL157958) 
 
           |-50      |-40      |-30      |-20      |-10      |1        |11       |21       |31       |41       
Mouse     CTCTCCCCTCCCCAGCCCGCTCCCTCCGCCCCGTTCCCTCCGCCCTCCCT G GGCTTCGGCCGCGGCGGAG  TGAACGGGACGGG  CCCGGCCGCAGAGCTCT
                                                                                     (MTE+)                     
Human     CTGCCAGGCGGCCCTTCTCCCCTCCCCTCTCAGTTCCCTCCGCCCTCCTC G GGCTCCAGCGGTG  GCGGAGCGAACG  G  GACCG  GCCCGGCTTCAGAGCGCG
                                                                               (MTE-)    (DPE-)                   
    249 Mouse: Zfp238 (30928, NM_013915) 
Human: ZNF238 (10472, AJ223321) 
 
           |-50      |-40      |-30      |-20      |-10      |1        |11       |21       |31       |41       
Mouse     GACAGCCTGCCATCCCTC  AGCCTTT  TATTCTAACACACAGACAGGGAGTT   A GTGTGTG  CGGATCTG  TGGTGGAGAAGG  T  ATCTC  ATTCCTCTCCAACATC
                             (TATA-)                            (INR-)               (MTE-)    (DPE-)                 
Human     GACAGCCTGCCATCCCTC  AGCCTTT  TATTCTAACACACAGACAGGGAGTT   A GTGTGTG  CGGATCTG  TGGTGGAGAAGG  T  ATCTC  ATTCCTCTCTAACATC
                             (TATA-)                            (INR-)               (MTE-)    (DPE-)                 
    250 Mouse: Usp21 (30941, AK029124) 
Human: USP21 (27005, BF999517) 
 
           |-50      |-40      |-30      |-20      |-10      |1        |11       |21       |31       |41       
Mouse     TTCCGAGTGTG  GGCGCGCA  GGCGCCTAAGAGACTGTGAAGCCGGTGGCCG G TGGCCGGGCGGCAC  CAACAAAGATGGC  GGCGGCCCTTGCGGCGGGAGCA
                       (BRE-)                                                     (MTE-)                          
Human     TTCGGGTGTGGATGCGCA  GGCGCTTT  GAGAGACGGTGAAGCCGGTGGCCG G TGGCCGGGCGGGAC  CAACAAAGATGGC  GGCGGCCCCTGCGGCGGGAGCG
                              (BRE-)                                              (MTE-)                          
    251 Mouse: Cfhl1 (50702, M29008) 
Human: CFHL5 (81494, NM_030787) 
 
           |-50      |-40      |-30      |-20      |-10      |1        |11       |21       |31       |41       
Mouse     TTTACCAAACT  AGCTTCCA  GAAATGCCCTGAAACAGAAAGTCATGCTCA  G C AGTTTT  AACAACAGGGTTCCCA  GAAGCACCACCAG  GATTGGAGTGCTCT
                       (BRE-)                                  (INR+)                       (MTE+)                  
Human     TTACTAAACT  AGCTTCCC  CTTA  GTACATT  GAAATTCAAAGTCATGCTTG  T A ACTGTT  AATGAAAGC  AGATTTAAAGCAA  CACCACCATCACTGGAGTATT
                      (BRE-)       (TATA-)                       (INR-)                (MTE+)                         
    252 Mouse: Rgs1 (50778, AK089293) 
Human: RGS1 (5996, NM_002922) 
 
           |-50      |-40      |-30      |-20      |-10      |1        |11       |21       |31       |41       
Mouse     CCTGTCTGCCTTACGCTA  TATAAAG  CAGCAGTCGTAGACTAACAGACAC  T C ACTCGT  TTTGAGAAGACCATGAGAGCGGCAGCCATCTCCATGCCAAGGT
                             (TATA+)                           (INR+)                                             
Human     TGTCTGCATTCTACTA  TATAAAG  CAGCAGAGACGTTGACTAGCGCA  TATT T GCT  AAGAGCACCATGCGCGCAGCAGC  CATCTC  CACTCCAAAGTTAGACA
                           (TATA+)                          (INR-)                          (DPE-)                  
    253 Mouse: Hs6st1 (50785, NM_015818) 
Human: HS6ST1 (9394, BC001196) 
 
           |-50      |-40      |-30      |-20      |-10      |1        |11       |21       |31       |41       
Mouse     GGGGCGGAGCCGCGACGAACTGCGGAGACGCGGCGGCGCACGGGGCGGT  G C AGAGCG  TTCTCGGCGC  TGAGCTTTGCGGG  CGGGTGCGGGCGCGCGCGGC
                                                             (INR+)                 (MTE+)                        
Human     GCGGGGCGGCGGCGGCGGACTGCGGAGACGCGGCGGCGCGCGGGGCGGC  G C AGAGCG  TTCTCGGCGCGGAGT  GGAGCGGAGCGGG  CTGGTGCTGGGGGCG
                                                             (INR+)                      (MTE+)                   
    254 Mouse: Bok (51800, AF027707) 
Human: BOK (666, BM548648) 
 
           |-50      |-40      |-30      |-20      |-10      |1        |11       |21       |31       |41       
Mouse     TGCTTCAGCTCG  GGTGTGGA  CGGGGCGGGCGCTGGGGCGGGGCGCGCGCT C GCGGGTTTGAATGGAAGGGTCT  AGACCG  CCGGAGACGGCAGCGAGCGGG
                        (BRE-)                                                        (DPE+)                      
Human     TCCCGGCTCG  GGCGTGGA  CGGGGCGGGCGCCGGGGCGGGGCGCGCGTCCT C GCGGGTCTGAATGGAAGGGTCGA  GGTCGT  CGTCGGCGGCGAGCAGATCC
                      (BRE-)                                                           (DPE+)                     
    255 Mouse: Hnrpu (51810, NM_016805) 
Human: HNRPU (3192, AK126868) 
 
           |-50      |-40      |-30      |-20      |-10      |1        |11       |21       |31       |41       
Mouse     CGGCAGGGGGCG  GGGCGCC  T  ATATTA  CGTGCGCGGCGCCGCCCCTGCGAG A GGACGTTGCGTGCTCGCTCGCGCCGGGCGAGTCTCCGCGTCTTGCCTCG
                        (BRE+)  (TATA+)                                                                           
Human     GCGCAGGGGGCG  GGGCGCC  T  ATATTA  CGTGCGCGGCGCCGCTCCTGCGAG A GGACGTTGCGTGCTCGCTCGCGCCAGGCGAGTCTCCGCGTCTCCCTCGC
                        (BRE+)  (TATA+)                                                                           
    256 Mouse: D1Ertd622e (52392, BC023951) 
Human: LOC90355 (90355, BC009203) 
 
           |-50      |-40      |-30      |-20      |-10      |1        |11       |21       |31       |41       
Mouse     GGCGGGGGAGGAGGCGGGACTACGGCGGGCCGTAGCTGGCGCCGAGCGCG G TGGGAGAGCAGCGGGTACCCGGC  TGGCTCCGCTGGC  GGCCTCTGCCCAC
                                                                                         (MTE-)                 
Human     GCGGAGGAGGGGGCGGGGACTAAGCAGGGCGGTAGCTGGCGCCGAGCGCG G TGGGAGAGGAGAGGGTACCCGGC  TGGCTCGGCTGGC  GGCCTCTGCCTGG
                                                                                         (MTE-)                 
    257 Mouse: D1Bwg0212e (52846, AK010503) 
Human: C2orf29 (55571, NM_017546) 
 
           |-50      |-40      |-30      |-20      |-10      |1        |11       |21       |31       |41       
Mouse     GCGACGCT  GTCGCGGA  AGGG  TGCGCTT  TACGGCCGCGGGAGGACGCAG  CG A GTCGG  CGCCTTGGGCCTCT  CGGCCGGGAAGCG  GGGTAGGCGGGCGAGGT
                    (BRE-)       (TATA-)                        (INR+)                     (MTE-)                     
Human     TTGCGACGCT  GTCGTGTA  ACAG  CGCGCTT  TACGGCCGCGGGGAC  GGAGCG A G  CCGGCGCCAGGGCCCCTC  GGGCCGGGAAGAG  GGGAAGGGGAGCGAGGT
                      (BRE-)       (TATA-)                  (INR-)                         (MTE-)                     
    258 Mouse: Vamp4 (53330, NM_016796) 
Human: VAMP4 (8674, AL035296) 
 
           |-50      |-40      |-30      |-20      |-10      |1        |11       |21       |31       |41       
Mouse     CGCGCCAGGCGGCGG  AGCGCCGA  GTCGCTCGATGAGCGGGGGCGCTGCA  G C AGAGCG  GAGCGGTGGCGGCTCTGCGCCTTCGGCCTTCCCCCTCCCACCC
                           (BRE-)                              (INR+)                                             
Human     GGCGCCAGGCCGCC  GAGCGCCG  ATCGGCTCGATGAGCGGAGGCGCTGCTG C GGCGCTGCGGCCGACGCCGGGTCCGCAC  CAACTG  TCTCCCCCTCCCAGC
                          (BRE+)                                                            (DPE-)                
    259 Mouse: Tlr5 (53791, AK036842) 
Human: TLR5 (7100, ENST00000342210) 
 
           |-50      |-40      |-30      |-20      |-10      |1        |11       |21       |31       |41       
Mouse     AAGAGTTGTGGGCGGGGCTCCAGCAGCGGGCGGTGTTTCCCTCTCCTCCC C GCTCTCCTGCTTTCC  CGAGCCCAGCGGC  AGCTTTGGTGCCTGGAGCCGA
                                                                                 (MTE+)                         
Human     GCCGAGACGGG  GGCGTGGC  CTGGCAGGTGGGCGGGCCCTTCCGTGGGCGG G GCCGCGAGGTTTTCA  GGAGCCCGAGCGA  GGGCGCCGCTTTTGCGTCCGG
                       (BRE-)                                                      (MTE+)                         
    260 Mouse: Slc40a1 (53945, AK033987) 
Human: SLC40A1 (30061, BC037733) 
 
           |-50      |-40      |-30      |-20      |-10      |1        |11       |21       |31       |41       
Mouse     CTCAAGCTCCGCCCCCGGCTCCC  TATAAGC  CGCTGCCCTGCCCGCGGCGG T GGCGGCGGCGGCGGCGGCGGCG  AGAGCAGGCTCGG  GGTCTCCTGCGGCC
                                  (TATA+)                                                 (MTE-)                  
Human     GGGAGCTCCGCCCCCGACTCGG  TATAAGA  GCTGGGCCCGGCCCACGGCGG C GGCGGCGGCGGCGGAG  AGAGCTGGCTCAG  GG  CGTCCG  CTAGGCTCGGAC
                                 (TATA+)                                            (MTE+)       (DPE-)             
    261 Mouse: Ngef (53972, AJ238898) 
Human: NGEF (25791, AL157664) 
 
           |-50      |-40      |-30      |-20      |-10      |1        |11       |21       |31       |41       
Mouse     CCCCGGGAGCCTCCA  GGCGCGCG  CTCGTCCGCGGGAGCGCTCTACAGCAG C GGCGGCGGCAGCTCCGGCT  TGAGCCGCGCGCG  CTGCGACCTCACTCAGA
                           (BRE-)                                                      (MTE+)                     
Human     CCCCGGGAGCCTCCA  GGCGCGCG  CCCGCCCGCGGGAGTGCTCGGCAGCGG T GGCGGCGGCGGCTGCAGCTCC  GGTCGA  GCCGCGCGCGCTGCGACCCCGC
                           (BRE-)                                                    (DPE+)                       
    262 Mouse: Irf6 (54139, NM_016851) 
Human: IRF6 (3664, AF027292) 
 
           |-50      |-40      |-30      |-20      |-10      |1        |11       |21       |31       |41       
Mouse     CCGGGGGATGTGG  CCACACCT  GGAGGGCGTGACCAGACCGTGCAGGG  GCT G TGGC  ATCCAGGGCTAGGC  TCTGAGCACTGGG  CACCGAGGTGGGCAGGTG
                         (BRE+)                              (INR-)                     (MTE-)                      
Human     CGGGGGGCGTGG  CCACACCT  GGGAGGCGTGGCCGGGCGGATGCGA  AGGCT G CG  GCGTCCTGGGGCGAGGCGCT  GACGTGAGCTCGG  CGCACCTGGGCTGG
                        (BRE+)                             (INR-)                           (MTE-)                  
    263 Mouse: Icos (54167, BC034852) 
Human: ICOS (29851, AF488346) 
 
           |-50      |-40      |-30      |-20      |-10      |1        |11       |21       |31       |41       
Mouse     TTCCTCTCCAGAAAACTGAAAACAACTGAGATCACGACTTCACTG  ACACT T TT  GAGCACTGGAGGAGAAGACTG  CAACTG  CTCCTGGCAGACATGAAGCC
                                                         (INR+)                        (DPE-)                     
Human     TTCCTTTCCAGCAAA  TAGAAAA  CAACCGAGAGCCTGAATTCACTGTCA  GC T TTGAA  CACTGAACGCGAGGACTGT  TAACTG  TTTCTGGCAAACATGAAGT
                          (TATA+)                             (INR-)                      (DPE-)                    
    264 Mouse: Rassf5 (54354, AK029321) 
Human: RASSF5 (83593, AF445801) 
 
           |-50      |-40      |-30      |-20      |-10      |1        |11       |21       |31       |41       
Mouse     CGCGGGCTGCGGGCCC  TTTAAAA  GCGCGCTCGACCGGAGCCGCCCCTTCT G TTGGCCAGGCTCGGTAGCTGCCGCCGCCTGCGGGCCTTGGCCATGGCTT
                           (TATA+)                                                                              
Human     AGCAGGCTG  CGGTTCC  T  TTAAAG  GCGCGCTGGTGTGGGGCGGCCCCTTCT C TCGGGGCTGGCTCGGGAGTAGCGC  AGTCGC  CAAAGCCGCCGCTGCCAAA
                     (BRE+)  (TATA+)                                                      (DPE+)                    
    265 Mouse: Smarcal1 (54380, AF209773) 
Human: SMARCAL1 (50485, AF432223) 
 
           |-50      |-40      |-30      |-20      |-10      |1        |11       |21       |31       |41       
Mouse     ATCTGCGGCCGTACTGGCTGTTGGGAAATGTAGTTCGACGGTGGCCGAGC C AT  TCAAATGC  CAATGGCCCGCTCCA  AGATGG  GTGAAATTGTAGCGCTTG
                                                                 (INR+)                  (DPE+)                   
Human     AACTGCGGCCG  CGTTGCCT  GCTGGGAAATGTAGTTCAGCGGTGGCCTAGC C CT  TCAGGCCT  GGCCGCTA  CAATAAGGCTGGC    GAACTC  GCAGCGCTTGAA
                       (BRE+)                                      (INR+)               (MTE-)     (DPE-)             
    266 Mouse: Hes6 (55927, AK007480) 
Human: HES6 (55502, AI815695) 
 
           |-50      |-40      |-30      |-20      |-10      |1        |11       |21       |31       |41       
Mouse     ATTGACATGTAAATAAGTTGC  TATAAAA  TGGGCGGGGTCCCGCGGGCCGG A   AGACTCCG  GAGAGCTAGTGGGG  CCTCCGGGCTGCG  ACCCACCTCGCTGA
                                (TATA+)                          (INR-)                     (MTE-)                  
Human     TTGAGATGCAAATAAGCGGC  TATAAAA  GGGGCGGGACCGCGGCGGGCCGG A AGCCGCGAGGAGCGCGGA  CGGCTGGGCTGCT  GCTGGGCGGCCGCGGGGC
                               (TATA+)                                                (MTE-)                      
    267 Mouse: Eef1b2 (55949, BC039635) 
Human: EEF1B2 (1933, NM_001959) 
 
           |-50      |-40      |-30      |-20      |-10      |1        |11       |21       |31       |41       
Mouse     GCCGGGGCGGCGGAGGG  AAACGCCT  CCGTCTCTATATAAGGAGTTTTCCG G AC  GCTCTGGG  TCCTTTTTTTC  CCTCAGCGCTGGC  CGCGGCCTCCCGGCG
                             (BRE+)                                (INR-)                  (MTE-)                   
Human     TCCGGCGCGGTGGAGGG  AAACGCCT  CCGTCTCTATATAAGGAATTTTCC  G G TCTCTT  CGGGTCC  TTTTTCCTCTCTT  CAGCGTGGGGCGCCCACAATTTG
                             (BRE+)                            (INR-)              (MTE+)                           
    268 Mouse: Nol5 (55989, BC076604) 
Human: NOP5/NOP58 (51602, CR618609) 
 
           |-50      |-40      |-30      |-20      |-10      |1        |11       |21       |31       |41       
Mouse     CGACCAGAAGTGACGTTACTTCCCAGAATTCTTAGAGAAGGAGACTCGCA   T ATTTGCT  GCTCCAC  GAGGAGAGGTGTG  GAGCGGCTGAGCAACGTGGTCT
                                                              (INR-)              (MTE-)                          
Human     TGTCGGAAGT  GACGTTGC  TATCCCAGAATCCTCAGAGAAGGAGTAGCGCG T TCGTGCGTCCTAGTTCCAGT  ACAGCGTGGAGGG  TTTAGGCAGCGTGTTC
                      (BRE-)                                                            (MTE+)                    
    269 Mouse: Fmo2 (55990, AF184981) 
Human: FMO2 (2327, NM_001460) 
 
           |-50      |-40      |-30      |-20      |-10      |1        |11       |21       |31       |41       
Mouse     ATTCAATTAGTGCTGCG  TATTAAA  CTACCACCCATCCTGTGGTAAG  TCAG T TAC  CTGACCACAGGGCA  GGATCCTCTTACC  TTTGTCCTTGCTAAGCAGT
                            (TATA+)                         (INR+)                     (MTE+)                       
Human     TTCAATAGTGCTGCT  TATTAAA  TTACCACTGCTACAATGTTTAAAG  CCAA T TAC  CTGAGCACATCATAA  GGATTCTCTTACC    GGTTGT  CCCAGTTAAGTA
                          (TATA+)                           (INR+)                      (MTE+)     (DPE+)             
    270 Mouse: Uchl5 (56207, AK017925) 
Human: UCHL5 (51377, AF157320) 
 
           |-50      |-40      |-30      |-20      |-10      |1        |11       |21       |31       |41       
Mouse     GCGCGCGGCAGGTGA  CGGCGCCC  GGAGGCTGTCGGGAAGTAGGCGGGGT  G A CGTGGG  GTTGACGCTCG  GCGGCGGGTTTTG  CTGAGGTCTGCGGCCGGTG
                           (BRE+)                              (INR-)                  (MTE-)                       
Human     TCGCGCTGCAGGTGA  CGGCGCCC  GGAGGCTGTCGGGAAGTAGGCGGGGTG A   CGTGTGGT  TGACGAGCTCG  GCGGCGGGTTTGC  TGAGATCTGTGGCCGGC
                           (BRE+)                                (INR-)                  (MTE-)                     
    271 Mouse: Rev1l (56210, NM_019570) 
Human: REV1L (51455, AF153594) 
 
           |-50      |-40      |-30      |-20      |-10      |1        |11       |21       |31       |41       
Mouse     GGTGTGGATGGGAGGGGGAGCCGGGCGCGCGCCAGAGGAGCGGCGGGGGA G GGGCGGAGCTGCCGCGGAGGCGCGAGGCGGGCGCAGCGACTGAGGGAGC
                                                                                                              
Human     GCGGAACGCTAGGG  GGCGTGGC  TGCGGCGCGCGCGCTAACCGGAGGGGGA G GGGCGGAGAGCCACCGC  GGAGCGCGCGCGG    GGTTGG  TTGCCGCGAGCGT
                          (BRE-)                                                     (MTE+)     (DPE+)              
    272 Mouse: Tmeff2 (56363, NM_019790) 
Human: TMEFF2 (23671, NM_016192) 
 
           |-50      |-40      |-30      |-20      |-10      |1        |11       |21       |31       |41       
Mouse     CACGTGACCCCGGGCGGGCTAAGGACCTGCTGCTTCCCAAACG  CCAGAGG G   ATGCGGGCGGCAGAGCGCGAGA  GGCGGCGGCTGGG  CTGCGGGGCGCTGT
                                                       (INR+)                             (MTE-)                  
Human     CACGTGACCCCGGGCGGGCTGAGGACCTGCTGCTTCCCAAACG  CCAGAGG G   ATGCGGGCGGCAGAGCTCG  AGAGGCGGCTGCC  GGGCTGCGGGGCGCCTT
                                                       (INR+)                          (MTE-)                     
    273 Mouse: Dpt (56429, AK019890) 
Human: DPT (1805, BE833115) 
 
           |-50      |-40      |-30      |-20      |-10      |1        |11       |21       |31       |41       
Mouse     GAACCACATGTCAGCGGCAGCTG  TATAAAG  TCAGCCCAGCAAACTGAGGG T G  ACATTTTT  TTTGCCA  GAAGCCGGGCAGT  AT  GGACCT  CACTCTTCTGTG
                                  (TATA+)                         (INR+)              (MTE+)       (DPE-)             
Human     CCACATGTCAGCGGCAGCTA  TATAAAA  CGGTCAGACCAGCAATCTT  AGAG T GAC  ATTGTTTGCCA  AAATCCCAGGCAG  CAT  GGACCT  CAGTCTTCTCTGG
                               (TATA+)                      (INR-)                  (MTE+)        (DPE-)              
    274 Mouse: MGI:1930773 (56695, AF318058) 
Human: MR-1 (25953, AF318057) 
 
           |-50      |-40      |-30      |-20      |-10      |1        |11       |21       |31       |41       
Mouse     CTGAAGGGAAACAAGCGAGCGTGCGCGCCTCCAGGGTCTCCTGGGAGGA  G T AGTTCT  CCGGGGCCCGCTACGC  GGACTT  CTGGGAAATGTAGTTTTTGCT
                                                             (INR+)                   (DPE-)                      
Human     CTGGAGGGAAACAAGCGAGCGCGCGCGCCTCCGGGGTCTCCTGGGAAGA  G T AGTTCT  CCTGGGTCCGC  TCTGCGGGCTTCT  GGGAGATGTAGTTTCTGGT
                                                             (INR+)                  (MTE-)                       
    275 Mouse: MGI:1930773 (56695, NM_019999) 
Human: MR-1 (25953, CR625727) 
 
           |-50      |-40      |-30      |-20      |-10      |1        |11       |21       |31       |41       
Mouse     ATGCAAGGA  GGAGCCCG  AGCCCCTCTCCCCTGAGCTAGAATACATTC  CCA G AAAG  AGGGGCAAGAACCCCATG  AAAGCCGTGGGGC  TAGCCTGGTGAGTT
                     (BRE-)                                  (INR+)                         (MTE+)                  
Human     GGGCAAGGA  GGAACCTG  AACCCCTATCCCCGGAGCTGGAATACATTC  CCA G AAAG  AGGGGCAAGAACCC  CATGAAAGCTGTG    GGACTG  GCCTGGTGAGTT
                     (BRE-)                                  (INR+)                     (MTE-)     (DPE-)             
    276 Mouse: Aldh9a1 (56752, BC003297) 
Human: ALDH9A1 (223, CD245902) 
 
           |-50      |-40      |-30      |-20      |-10      |1        |11       |21       |31       |41       
Mouse     GCTATCTCCCCCTCCA  GTAGTAAT  CTGCTGGGGCTTCCGGACGCTTTGCA C GCGTAGCAGATGATCCTCGGTGC  AGTCGG  TTCTGTGCTGACCTCGCTCC
                            (BRE-)                                                     (DPE+)                     
Human     CCGCGGCCCCGCCCCTCCCGCGGCCCGTCAGCCTCTGCCGCGGAGCTG  CG T CCGCC  ACTCATGTTTCTC  CGAGCAGGCCTGG  CCGCGCTCTCCCCGCTTC
                                                            (INR-)                    (MTE+)                      
    277 Mouse: Dnajb10 (56812, BC085162) 
Human: DNAJB2 (3300, NM_006736) 
 
           |-50      |-40      |-30      |-20      |-10      |1        |11       |21       |31       |41       
Mouse     CCCGCCCCCGCCCCCTCGTGTGACCGGCCGAGCCGGCCCTCTGAGTCTCT A   GTAGTTTC  CAGGGCGCTGACGCATGCTTGCGCACACAACCCAGCCTGGC
                                                               (INR+)                                           
Human     GGCCCCCACCCCCCCAGGGGACAGGCGGAGCCGGCCTTCTCCCCGTCTCT A   GTAGTTTC  CGCACCGCTGACGCATGCCTGCGCGCACAGCTGGGCCGGGC
                                                               (INR+)                                           
    278 Mouse: Jph1 (57339, NM_020604) 
Human: JPH1 (56704, CV569417) 
 
           |-50      |-40      |-30      |-20      |-10      |1        |11       |21       |31       |41       
Mouse     CTAGGGGCGGGCGCGGGGCGGTGCCCGGCTCACGTGGGCGGGTGGGAG  CA G CTGAA  AGTCCGCTCTGG  AGACTGGGCTGTC  CTCTCGCGCGCGGCGCTGG
                                                            (INR-)                   (MTE-)                       
Human     CGAGGGGCGGGCGCGGGGCGGTGCCCGGCTCACGTGGGCGGGCGGGAG  CA G CTGAA  GGTCCGCTCGGG  AACCTGGGCTGTC  CTCTCGCGCGCGGCGCTGG
                                                            (INR-)                   (MTE-)                       
    279 Mouse: 1300007B12Rik (57439, AK004922) 
Human: C1orf37 (92703, AF070537) 
 
           |-50      |-40      |-30      |-20      |-10      |1        |11       |21       |31       |41       
Mouse     CAACGGGACCCAGAGCCCTCGCCGAAGTCCTCGCGATAGCCGCGGCCTCG G TGCGGGGATGGCCGC  AGAGCCGGGTGGA  GGTGCTCTGCGGCTCCGGGAG
                                                                                 (MTE+)                         
Human     AACGCGACTCGGAACCCGGACCTATGTTCTCGCGAGAGTTAGCGGCCTCC   G GTGTGGG  ATGGCCGCGGAGCC  GGGCGGAGCTGGC  TTGCGGCTCCCGGGG
                                                              (INR-)                     (MTE-)                   
    280 Mouse: Wdr12 (57750, BC052386) 
Human: WDR12 (55759, CN426769) 
 
           |-50      |-40      |-30      |-20      |-10      |1        |11       |21       |31       |41       
Mouse     GAATAGGTACTTGATGTGTGCGGGCTGGGCGCTGGTGGAAATCGAACCTC G T  GGTATGTT  CTGTA  GGAACCTGAGGGA  GC  TAACTG  AAGCCTAGCTCCTG
                                                                (INR-)            (MTE+)       (DPE-)               
Human     GAGTAGGTAGT  CGATGTTT  GTGGTCTGGGCGTCTGTAGAAGGGC  AACCTC G T  GCTTTCTGCAGAGGAGACCG  GAGGGCAGAAGGC  AGAGTCCAGGCTTAG
                       (BRE+)                             (INR-)                           (MTE-)                   
    281 Mouse: Wdr12 (57750, BC052386) 
Human: WDR12 (55759, AK056092) 
 
           |-50      |-40      |-30      |-20      |-10      |1        |11       |21       |31       |41       
Mouse     TCTGGGGTTTGTAGTATCTGAGCG  CTGAGTC  TAAAGGGTCTGGGTCTAAA G   ACACGCTT  AAAATTTGGCTCAAGCT  AGTTAT  TTTCTTTATTTTCCTAAT
                                   (TATA-)                       (INR+)                    (DPE+)                   
Human     TTCTGAGACTTGTAGTAACTAGGAGCTGTGTTTGAACTATCCAGGCTCAG G   ACAGCCTC  TTGAAAAA  AAATTTTTTATTA  ATAAAGCGGATTTGAGTGGG
                                                               (INR+)               (MTE+)                        
    282 Mouse: Rnf25 (57751, AK002399) 
Human: RNF25 (64320, NM_022453) 
 
           |-50      |-40      |-30      |-20      |-10      |1        |11       |21       |31       |41       
Mouse     CTTTTACTCCGCCCCGCCCTAGTTTTCCGCCTCTAACTTCCCAAAAGGTG T TT  CCGGTTCC  GGCCAAGAGCCGGAGA  AGACAT  GGCGGCGTCTGCGTCGA
                                                                 (INR+)                   (DPE+)                  
Human     GCTAGCTGCCCACCCCTCTTAGTTTTCCGCCTCCGACTTCCCTAAGG  GCA T TTCC  GGTTCCGGCTGCGGGCCGGTGA  AGATAT  GGCGGCGTCTGCGTCTG
                                                           (INR+)                         (DPE+)                  
    283 Mouse: Kcne4 (57814, BC032920) 
Human: KCNE4 (23704, BC014429) 
 
           |-50      |-40      |-30      |-20      |-10      |1        |11       |21       |31       |41       
Mouse     TGACGATGCATTTCTTGAC  TATATAT  CCCAAGGACAGCAGCCGGGCTG  TC A GAGCG  CAGAGCCTGACAGAGAAGAGAAA  CATCCT  CAGATTTGGCCGTTT
                              (TATA+)                         (INR+)                          (DPE-)                
Human     TGACGATGCACTTCTTGAC  TATATAT  CCCAACTGCAGCAGCGGAGTTG  TC A GAGCG  CAGAGCCGGACAGAGCAGAA  GAACCC  TCTTGGACTGGACGATTT
                              (TATA+)                         (INR+)                       (DPE-)                   
    284 Mouse: Rgs20 (58175, NM_021374) 
Human: RGS20 (8601, AK094503) 
 
           |-50      |-40      |-30      |-20      |-10      |1        |11       |21       |31       |41       
Mouse     GGATTGCCCTGCCCCAGGAAAGCGAGCGAGCGCCGGGGCGGAGAGGAG  TC A GAGAA  GGGAAAGGAGGAGGAAGA  GAGAAGAGCTCCG  GAGCTTAGACCCA
                                                            (INR+)                         (MTE-)                 
Human     ACGTGGGATTGCCCGGCCGGG  TAAAAGG  AGTGAGGGGGCGGGGAGGAG  GC A GAGCA  AGGGGAGGAAGAG  GCCGGGAGAAGAG  GGCTAGAGCAAAGACCGA
                                (TATA+)                       (INR+)                    (MTE-)                      
    285 Mouse: Rqcd1 (58184, AK005025) 
Human: RQCD1 (9125, NM_005444) 
 
           |-50      |-40      |-30      |-20      |-10      |1        |11       |21       |31       |41       
Mouse     AACTTTGGCCCTCCTACCATTTTCCTCACGCGCAACCGCTTGAGCATGC  G C AATTCG  TGTCCTTC  CAAGCCTGCTGGC  CTTTTCCCGCCCTTGTCTGGCC
                                                             (INR+)               (MTE+)                          
Human     TTCGACGCCCCCCCACCATTTCCCTCCTACGCAACCCAGTCGGGCATGCG C A  ATAGTTGT  CTTGCCGGGACTGTC  CATCTT  TTCCCGCCGTTCACTGGCT
                                                                (INR+)                  (DPE-)                    
    286 Mouse: Stx6 (58244, NM_021433) 
Human: STX6 (10228, CR591752) 
 
           |-50      |-40      |-30      |-20      |-10      |1        |11       |21       |31       |41       
Mouse     CGCCGGAGGTGCCGGGGGAGGAGGCTGCCGTGGGGGCCGGCTT  GGAGTGG G   ATTCGACCCGCGGCG  CTGACCGACTGCT    GGACCG  ACGGCCGACGGGCAC
                                                       (INR-)                      (MTE-)     (DPE-)                
Human     GGGAGGAGCCGGGACTGGAGGCTGCCGAGGGGGCCGGCGCCCGAGTCCG  G G ATTCGG  CCAGTGGTGC  TGAGCGAGTGCT  G  GACCA  GCGGCCGTCCTGTGC
                                                             (INR-)                 (MTE+)    (DPE-)                
    287 Mouse: Stk25 (59041, BC071218) 
Human: STK25 (10494, BC007852) 
 
           |-50      |-40      |-30      |-20      |-10      |1        |11       |21       |31       |41       
Mouse     GCGGGACTCCTAGTCCCACAGAGCTTCGCGCTCGCCACGACGCCCTGC  TC A GAGCC  GCTGCCTCCAC  CTCACGGGCTGGG  AGGCCTGGGCGTTCCGGGAG
                                                            (INR+)                  (MTE-)                        
Human     GTGGGACTCGGGGTCCCAGAGAGCCGTGCGCGGGCCGCGACGC  CGAGCAC C   GCCCTCGCCGTCG  CCTCCGGGCTTTC  TCC  GGTCGC  TGCCGCCACCACCG
                                                       (INR-)                    (MTE-)        (DPE+)               
    288 Mouse: Nek7 (59125, AK088173) 
Human: NEK7 (140609, CR614163) 
 
           |-50      |-40      |-30      |-20      |-10      |1        |11       |21       |31       |41       
Mouse     ATGCCGGGATGTTTACCCTCCTGACAGCCGCGGCCGCCGGAGGAGGAGCG G AGCCGGGAGGATGGGCCGCCGC  TAACCT  CGCAGTGGGAAGCGCGGGCCG
                                                                                    (DPE-)                      
Human     ATGCCGGGATGTTT  ACACTCCT  GACAGCGGCGGCAGCAGGAGGAGGATCG   G GAGTCGC  GGGAGGATG  GGCCGCCGCTAGG  CTCGCACTCCGGACGCGCCT
                          (BRE+)                                (INR+)                (MTE-)                        
    289 Mouse: Ncstn (59287, AK129102) 
Human: NCSTN (23385, AF240468) 
 
           |-50      |-40      |-30      |-20      |-10      |1        |11       |21       |31       |41       
Mouse     GACAAGGACTTCCGGTCTCTGGAGCGCTGGGCCACAGAGACGATGCTAGC G GA  AGCCTAGA  GAGGCCGCTA  CGAGTCTGGAAGG  GACCTGAGGCTTCCGC
                                                                 (INR-)                 (MTE+)                    
Human     AACACGAACTTCCGGTCTCTTAGGCTCCGGGCCACAGAGACGGTG  TCAGT G GT  AGCCTAGAGAGGCCGCTAAC  AGACAG  GAGCCGAACGGGGGCTTCCGC
                                                         (INR+)                       (DPE+)                      
    290 Mouse: Gpa33 (59290, AK008784) 
Human: GPA33 (10223, BC069789) 
 
           |-50      |-40      |-30      |-20      |-10      |1        |11       |21       |31       |41       
Mouse     TGAGCTGGAGGAGAGTCACAGGGACTACAGGCTGAACCAGGCCAGAGG  CC A TAGCT  TTAACCAG  ACAGCCCAGACCT  GTCCAGTGCCCTGCCAGTTCCAG
                                                            (INR+)               (MTE+)                           
Human     TGAGAAGAGGGAAAATTGCAGGGACCTCCAGTTGGGCCAGGCCAG  AAGCT G CT  GTAGCTTTAACCAGACAGCTC  AGACCT  GTCTGGAGGCTGCCAGTGAC
                                                         (INR-)                        (DPE+)                     
    291 Mouse: Niban (63913, NM_022018) 
Human: C1orf24 (116496, AF288391) 
 
           |-50      |-40      |-30      |-20      |-10      |1        |11       |21       |31       |41       
Mouse     GGCGACGCCTCTCAAGGAAGTT  GGCCCTT  TAATTACAGAGCCAGTTCCCT G   ACACATCC  ACCTTCAGCGG  CAAGCAGCAGGCG  CAGTTGGTGTCAGTCCT
                                 (TATA-)                         (INR+)                  (MTE+)                     
Human     CTCTCGCCTCTCGAA  GGAAGTT  T  GCTCTT  AATTTCAGAGCCGGGTTCGCC G   TCGGATCA  ACCTCCAGGAGCTA  GCAGCGGGCGCGG  ACCGGGCAGTTTCC
                           (BRE+)  (TATA-)                         (INR+)                     (MTE+)                  
    292 Mouse: Ralb (64143, AF174296) 
Human: RALB (5899, BF328742) 
 
           |-50      |-40      |-30      |-20      |-10      |1        |11       |21       |31       |41       
Mouse     GGAGGGCTGCCTCT  GGAGAGGA  CAGGGTTGCCGGGCGGACAGCGGGAACG C GCTCCGGGGGGTGGGGACGCG  CGAGCTCACCACT  CCTCAATGACAAATC
                          (BRE-)                                                         (MTE+)                   
Human     GCGAGGGCGCGCTCTCGAGAGGAGGGGTTGCCTAGGCGACGCCGGAGGCG C GCTCGGGGGGTGGGAAAG  CGAGCCCGGCAGC  TCAATGACAAATCGGTGG
                                                                                    (MTE+)                      
    293 Mouse: Rgs18 (64214, AF302685) 
Human: RGS18 (64407, BC020632) 
 
           |-50      |-40      |-30      |-20      |-10      |1        |11       |21       |31       |41       
Mouse     CATTTCCTTCAACAATTCAG  TACAAGA  CCAATCTTGAGTTCCTCATTT  TC A GACTT  GCAGAGGCAGAAAC  ACAGCTCTTCACT  TTCTTTCATGTTATTTT
                               (TATA+)                        (INR+)                     (MTE+)                     
Human     CATTTCCCTAAACAACTTAGG  ACCAAAT  TGTACTTCATTTCCTCAGTTCT   G CATTTCT  GCAGAGACAGAAAG  AAACGCAGCTCTT  GACTTCTTTTTTGTA
                                (TATA-)                         (INR+)                     (MTE-)                   
    294 Mouse: Itm2c (64294, AK036377) 
Human: ITM2C (81618, AF271781) 
 
           |-50      |-40      |-30      |-20      |-10      |1        |11       |21       |31       |41       
Mouse     GGGCGGGGACAGAGGCGA  GGACTCCG  GAGCCATCCAAACTTCCGGTGCCT G CGGCGCAGCAAGAAGCAG  GCATTGCGATCGA    GGTTGC  ACTGGCAGGGGC
                              (BRE+)                                                  (MTE-)     (DPE+)             
Human     GGGTGGGGTTAGAGAGAG  GGACGCGA  GCGGGATCCAAACTTCCGGTGCCT   G CAGAGCT  CGGAGCGGCGGAGGCAG  AGACCG  AGGCTGCACCGGCAGAGGC
                              (BRE+)                            (INR+)                    (DPE+)                    
    295 Mouse: Mrps14 (64659, AK007673) 
Human: MRPS14 (63931, AU099472) 
 
           |-50      |-40      |-30      |-20      |-10      |1        |11       |21       |31       |41       
Mouse     GCATAAGCCCCGCCTCCACAGAACCTGTTTCCGGTGTTGTACTGCGGCG  G T GGTTGG  CTGGCTGGGCAGACAACATGGCGG  CGTCCG  TGCTAGGCTCGCT
                                                             (INR+)                           (DPE-)              
Human     GCCCCGCCCCTCGTGTCCTGCGCTATTCGGCAGTGTCAATAAAGTTTCAG C   GGTTTGTA  GTTTGTAGCGGACAACATGGCGGCCTTCATGCTGGGCTCGC
                                                               (INR-)                                           
    296 Mouse: Sdhc (66052, AK012818) 
Human: SDHC (6391, NM_003001) 
 
           |-50      |-40      |-30      |-20      |-10      |1        |11       |21       |31       |41       
Mouse     CGGCTCCGCCCCCAAAGGCAGGGCCACACGGGGGGAGGAGCGATGCG  TCA T TTCC  GTCCAGGCCGGAACTCA  AGATGG  CTGCGTTCTTGCTGAGGTGACT
                                                           (INR+)                    (DPE+)                       
Human     CCCCCAGCCG  GCGCGCCT  CCGCCCTCGGGTGGCGGGGCCGCCTGGCG  TCA C TTCC  GTCCAGACCGGAACCCA  AGATGG  CTGCGCTGTTGCTGAGGTGACT
                      (BRE+)                                 (INR+)                    (DPE+)                       
    297 Mouse: Fbxo36 (66153, AK008324) 
Human: FBXO36 (130888, AK123085) 
 
           |-50      |-40      |-30      |-20      |-10      |1        |11       |21       |31       |41       
Mouse     GGCTGCGGCCCCCTGACCCCGGGCAAGCCCCGCCCCTTCCTGGTTGTCCT A GCGACGGCGGTGGC  GTCCCAAGATGGC  GTCGTGGCTGCCGGAGACTCTG
                                                                                (MTE-)                          
Human     GGTTTTGGCCCCTTGACCCCAACCAAGCCCCGCCCCTTCCTGGTTGTCTT A GCGACGGCGGTGGC  GTCCCAAGATGGC  GTCGTGGCTGCCGGAGACTCTC
                                                                                (MTE-)                          
    298 Mouse: 1110060M21Rik (66208, AK002413) 
Human: SCIRP10 (29937, AF173937) 
 
           |-50      |-40      |-30      |-20      |-10      |1        |11       |21       |31       |41       
Mouse     CCCTAGGAGCCGACTGTGCTCCGCCCCGCCCCGCCGTCCTCCCGCCTGCT C CTCGCTGTCTATGGCGCGCCCCGCGCCCTGGTGGCGGCTGCGGCTGCTG
                                                                                                              
Human     GGGCGGGCGTGCTAGGGCCCCGCCGCCCTGGCCCGGCCTTGCCTTGCGCT G CGCGCTCACCATGGTGGGCCCCGCGCCGCGGCGGCGGCTGCGGCCGCTG
                                                                                                              
    299 Mouse: Tmem9 (66241, AK021225) 
Human: TMEM9 (252839, AK094588) 
 
           |-50      |-40      |-30      |-20      |-10      |1        |11       |21       |31       |41       
Mouse     CGGCGACTGTAGCAGCC  AATAGAT  GCTGAAGGGGTGGAGCCTGCTGA  TCA G TGGC  GGCTGCGGCTGAGCTTGCA  GGTTGG  TAGACTCGGCATTGGGGGAG
                            (TATA+)                          (INR+)                      (DPE+)                     
Human     CTGCTCTGGCAACCAA  TAGAAGC  TAGGAGAGGGCGGGGACAACTG  GGTCT T TT  GCGGCTGCAGCGGGCTTGTA  GGTTCG  TGGACGCGGCATTGGGGGAGG
                           (TATA+)                         (INR-)                       (DPE+)                      
    300 Mouse: 1810018L02Rik (66261, AK017209) 
Human: TM4SF20 (79853, AK026453) 
 
           |-50      |-40      |-30      |-20      |-10      |1        |11       |21       |31       |41       
Mouse     ACCAGAACGTTGGGA  TAAAAGG  CCAGGCAGAGAGCTACACGAGCCCAGCC A CCCTGAGGAGGTTT  CTAACTGAACCAG  GAAAACCATGACGTGCTGTGAA
                          (TATA+)                                                 (MTE+)                          
Human     TAACCAGAAGATTGG  GATAAAA  GGCAACATAGTACCATGCAAGCATAG  CC A CTTTG  ACAACGTTTC  TGAGCCAGGGGTG  ACCATGACCTGCTGCGAAGGA
                          (TATA+)                             (INR+)                 (MTE+)                         
    301 Mouse: Ing5 (66262, AK028397) 
Human: ING5 (84289, AI124772) 
 
           |-50      |-40      |-30      |-20      |-10      |1        |11       |21       |31       |41       
Mouse     AGCGCGCGGCGCCGCCCCGCCCCGAGTCCGCGGCACCGCCCGCTCGC  GCA G ACCC  CGAGCGTGGCCG  AGGCGAAGATGG  C  GACTG  CCATGTACTTGGAGC
                                                           (INR+)                   (MTE-)    (DPE-)                
Human     GCGCTGGCACCGCCCCGCCCCCGCCTCCCGCGGCACCGCCCGCCCGC  GCA G ACCC  CGAGCGCGGCCGCGGACGA  AGATGG  CGACCGCCATGTACTTGGAG
                                                           (INR+)                      (DPE+)                     
    302 Mouse: Blzf1 (66352, NM_025505) 
Human: BLZF1 (8548, NM_003666) 
 
           |-50      |-40      |-30      |-20      |-10      |1        |11       |21       |31       |41       
Mouse     AGGGAGGCTTGGGCGGGTTGGCTTCCGAGTGCCAGGGGAGTTTA  CAAGTC A G  CCGGCTGCTGAGCGAGTGA  AGAGCCTGAGCGG  TGGACATTGAAGTAAG
                                                        (INR-)                          (MTE+)                    
Human     GGAGGGACTGGGCGGGTCGGCTTCCGAATGGAAGAGGTCTGTGAGAAGTT   A ACCTGGT  GATACCGATCCGA  AGAGCCTATCAAG  TGAAGCCCCCTGAAAT
                                                              (INR-)                    (MTE+)                    
    303 Mouse: Blzf1 (66352, AK006544) 
Human: BLZF1 (8548, NM_003666) 
 
           |-50      |-40      |-30      |-20      |-10      |1        |11       |21       |31       |41       
Mouse     CGCGACGCCACCTTCT  CCGTTCCA  ACGCGTTCACTTCCGTCTGGCTG  GTA G TGAC  CCGGAAGTTTAAGCGT  CCATACCGCTTGC  ATTATCTATTGGTACT
                            (BRE+)                           (INR+)                       (MTE-)                    
Human     CGAGACGCCATTATTCTGTTTCCG  GCAGTTT  TACTTCCGTTTGTTTAA  CA G CTGGC  CCGGAAGTTTAAGTGA  ATATGCGGCTTGG  GCTCCAAAAGTTGCT
                                   (TATA-)                    (INR-)                       (MTE-)                   
    304 Mouse: Ppp1r7 (66385, AK031983) 
Human: PPP1R7 (5510, BC051689) 
 
           |-50      |-40      |-30      |-20      |-10      |1        |11       |21       |31       |41       
Mouse     CGCAGAACGACTTAGGCAG  CATAACA  AGGGACCAAGAACCAACC  AGACAC C C  TGATGGGCGGACCTCAGTGAAA  GCAAGCAGCTGAG  CGCGCGCAAACAG
                              (TATA+)                     (INR+)                             (MTE-)                 
Human     TCGCACAGCGACT  AGCACCGA  TT  GACAAGC  TCCACGGACCAGCCACTTGC G   CGTATGGG  CGGGAC  TAAGCTAGCCAGA  GTCTAGAAGCCCGCGCTAAGGG
                         (BRE-)     (TATA+)                        (INR-)             (MTE+)                          
    305 Mouse: Ppp1r7 (66385, AF067129) 
Human: PPP1R7 (5510, BC051689) 
 
           |-50      |-40      |-30      |-20      |-10      |1        |11       |21       |31       |41       
Mouse     CCTGGAATCCTGATTGGCCCATGGCCTAAGGCGACAGATTCCGGAAAGGG A AAGAGCAGCCAATATGGCGGC  AGAGCGCGGCGCG  GGGCAGCAACAGTCG
                                                                                       (MTE+)                   
Human     CGGGAGCCCTGATTGGCTGAGGGGTCTGAGGCGACAGATTCCGGAAAGGG G AAGAGCAGCCAACATGGCGGC  GGAACGCGGCGCG  GGGCAGCAACAGTCG
                                                                                       (MTE+)                   
    306 Mouse: Mgst3 (66447, AK003246) 
Human: MGST3 (4259, CB106702) 
 
           |-50      |-40      |-30      |-20      |-10      |1        |11       |21       |31       |41       
Mouse     GGGGCGGGTCCTGCGGGACAGCTAGAGCCGCACCTAGGCACTGCTGTGCT T C  TCAGGTCT  GTACCAGGCGCACGAAGGTGAGCCAGGTAAGTGCTGGCAG
                                                                (INR+)                                          
Human     CATCTAGCCCCGCCCCA  GGCGAGGG  CGCCGCACCCACACCGCGCTGC  GCA G TTTT  GTTCTGCTCCAGCTGTT  CGAAGGTGATCCA  GGTGAGTGCTAGCAC
                             (BRE-)                          (INR+)                        (MTE-)                   
    307 Mouse: Ndufb3 (66495, BC028669) 
Human: NDUFB3 (4709, BC018183) 
 
           |-50      |-40      |-30      |-20      |-10      |1        |11       |21       |31       |41       
Mouse     TCCGTCCTGGAGCAAGACAAAC  ACGGCTT  TATCGTCCTTCTCC  TCAATAT C   TTCTACCTCAAGCA  GACTCCAGCTGCA  AAGTGTGGGAAATAATCTCTAC
                                 (TATA-)                 (INR+)                     (MTE-)                          
Human     CAAGCAAGGACAACA  TATTATC  TTCCCCGCCCTTCTCTTTTCTACAG  TAT C TGTT  ACCTCAGTCA  GATTGAGGATGCA  CATATACTGGGAAAATAATCGA
                          (TATA+)                            (INR-)                 (MTE-)                          
    308 Mouse: 5730449L18Rik (66637, AK017650) 
Human: C1orf19 (116461, AF288394) 
 
           |-50      |-40      |-30      |-20      |-10      |1        |11       |21       |31       |41       
Mouse     CGGGAAGCGGAACTCCCGCGCGCTGGACCGTCTGCTTCTGGCCGAGCGG  G G TTTGCG  GTGCACCACGG  CTTTCTGCGCGGG  CGGCATGGAGGAGCGCAGC
                                                             (INR-)                  (MTE+)                       
Human     GGGGAAGCGGAACCCACA  GGCGCGCG  CGCCGCTGCTTCTGGCCGGGCGCG G GTCGTGGTGCACCACG  GGAGCGCCGCACC  GGCCGGCATGGAGGAGCGCG
                              (BRE-)                                                (MTE+)                        
    309 Mouse: Rpe (66646, BC019126) 
Human: RPE (6120, NM_006916) 
 
           |-50      |-40      |-30      |-20      |-10      |1        |11       |21       |31       |41       
Mouse     TGGGAGGGGGCGGAGCCAGTGCGGCGCGCCCAATCGGGACGGAGTCT  TGG C TGGC  CCGGGCTCTCGTGGG  CTCACTCGCTCTA  GGGAACCGGCACCATGG
                                                           (INR-)                      (MTE-)                     
Human     GGAGAAGGGGCGGGGCCAAAACTGCGCGCCCAATCGGGGTGAC  GCTCTAG C   CTTGCCGGGGACTC  GTGGGTAACTTGC  TTTTGGGAGCCAGCGGTATGGC
                                                       (INR-)                     (MTE-)                          
    310 Mouse: Spag16 (66722, AK015884) 
Human: SPAG16 (79582, BC025379) 
 
           |-50      |-40      |-30      |-20      |-10      |1        |11       |21       |31       |41       
Mouse     GGGTTCTCGCGAGAGTCG  CGACGCCA  GTCGGAGGGGCACCGAGGGGC  GCA G TTGC  AAGCATGGCTGCTCCGTCTGG  GGTCCC  GCCGCTGCGGGTGCTGGA
                              (BRE+)                         (INR+)                        (DPE+)                   
Human     GCTGTTGCCCTTAG  GGACGGCT  GTGGGCCTGCTGGGGGTGGGGGCCCGAA G CG  CCAGAGAT  GGCTGCTC  AGCGAGGGATGCC  CAGCTCCGCCGTGAGGGT
                          (BRE+)                                   (INR+)               (MTE-)                      
    311 Mouse: Bcs1l (66821, NM_025784) 
Human: BCS1L (617, NM_004328) 
 
           |-50      |-40      |-30      |-20      |-10      |1        |11       |21       |31       |41       
Mouse     CGGGGCGGGACTAGCGGCGCTGCCTCTTCGTAGGCCCGGCGAAACCAT  CG A GTGTG  AGGGCCAGAGAGTC  ATGGAGGGAAGGG  GGCGGAGTGACAGATTT
                                                            (INR-)                     (MTE-)                     
Human     GGCGGGGCCGAACGCAGCTTCCCCAAGGTGCAGGCGCGGTGAAACCAT  CG A GACGG  AGGGCCAGAGAG  TCACGGCGGTGAG  AGGGCTGAGTGACGGGTTA
                                                            (INR+)                   (MTE-)                       
    312 Mouse: Ddx18 (66942, AK012706) 
Human: DDX18 (8886, NM_006773) 
 
           |-50      |-40      |-30      |-20      |-10      |1        |11       |21       |31       |41       
Mouse     TTCCGCCCGAAGCATTTCCGCCCCGCGGGTCCGCAGGCTCCAGCGGT  ACA C TTCC  GACTTGCAATTGCGCACGTG  CATCTG  TCTCGGTGCAGACGGCTGG
                                                           (INR+)                       (DPE-)                    
Human     CCGCTCTGGAGCATTTTCGTTCCGCCGGGTGCCAGCGTTCCTGTGACG  CG T TTCCT  GTTGGCCGAGCTGCGCACGTGCGGCCGGAAGGGAAGTAACGTCA
                                                            (INR-)                                              
    313 Mouse: 2310028N02Rik (66950, AK049641) 
Human: FLJ10874 (55248, AA768575) 
 
           |-50      |-40      |-30      |-20      |-10      |1        |11       |21       |31       |41       
Mouse     CGCCCGCGGCGGCAGCAAAAGATGCGCATGCGTTGAGCGCCGCCA  CGCCT G GT  CTTGGGCGGTAGACTAGAGCG  GGTCTT  GCTTAGTAGAGCGGGCACAT
                                                         (INR-)                        (DPE-)                     
Human     GACTCCTGGGCGGCAGCGGCCAGGCGCATGCGCCGAGTATCGCCACGCCT G G  TCTCTGGG  ACGCCCCTC  CGGACCGGTTTCG  CCTCGCGGAGCCGGTAGG
                                                                (INR-)                (MTE-)                      
    314 Mouse: Cdca1 (66977, AK044882) 
Human: CDCA1 (83540, NM_031423) 
 
           |-50      |-40      |-30      |-20      |-10      |1        |11       |21       |31       |41       
Mouse     GGCGGCCGGTTTGAAAAATGACAACGGTCAGCGGCTTTCGGTTGGACGC  T C GGTTTT  TAACTGTTTT  TCCGTGAGCTGCT  AGGCCTCCAGGTGAGTTTGA
                                                             (INR+)                 (MTE-)                        
Human     GGCGGCAAGTTTGA  AAAGTGAT  GACGGTTGACGTTTGCTGATTTT  TGACT T TG  CTTGTAGCTGC  TCCCCGAACTCGC    CGTCTT  CCTGTCGGCGGCCGGCA
                          (BRE-)                           (INR+)                  (MTE-)     (DPE-)                  
    315 Mouse: 1700019D03Rik (67080, AK006523) 
Human: MGC13057 (84281, AK075168) 
 
           |-50      |-40      |-30      |-20      |-10      |1        |11       |21       |31       |41       
Mouse     ACACAAGGCTCTGAGGAAAAA  TATTAAT  AATGTTAAATTTTTGCAGT  CCA T TTTG  AAACGAACAAGGCTTTCATGATA  CATCTT  GTCTTTGAAGATGAGA
                                (TATA+)                      (INR+)                          (DPE-)                 
Human     GATGTGATTCTGTGGGAG  AAGTATT  AATAGCCTTAAACTGGTACAGGTTG T TT  TGAAATTA  AGCCTG  CCAAGACACTTGT  T  TGTCCT  TGAAGATGAGAAC
                             (TATA-)                               (INR+)             (MTE-)      (DPE-)              
    316 Mouse: 2810022L02Rik (67198, NM_144882) 
Human: DNAPTP6 (26010, AF193059) 
 
           |-50      |-40      |-30      |-20      |-10      |1        |11       |21       |31       |41       
Mouse     GGAGGGACAGGAT  CCGTGTCA  GAACGTGCGTGTGAGCGGATACAAAACCC A GGGAGGCGTGATCGGCGGCGTGTGTGCAAGCCCGAGCGAGGGGCGCCGG
                         (BRE+)                                                                                 
Human     GGAGGGACGAGAT  CTGTGTCA  GAACGTGCGTGTGAGCGGATACAAAACCC G AGAGAGGCGTGAGCAGCGCTGTGTTTGCGAGCGGGAGCGAGGGGCGCCG
                         (BRE+)                                                                                 
    317 Mouse: 2810430M08Rik (67223, AK013204) 
Human: CGI-115 (51018, NM_016052) 
 
           |-50      |-40      |-30      |-20      |-10      |1        |11       |21       |31       |41       
Mouse     GTTCCGGAACTAGAGGAGGAAGTTCCGTGTGTTGCAACACGACG  GAATTA T C  AGGTGGCACTTCCGGCGAGCCA  AGATGG  CTGCGGCCGTTCAGGACTCA
                                                        (INR-)                         (DPE+)                     
Human     TTACCGGCACTAAATGCAGAAGTTCCTCTGGTTGCCACCGGACG  CAACTG T C  AGGTGACGCTTCCGGC  GCAGAAAAATGGC  AGCCGCCGCTCCGGACTCA
                                                        (INR-)                       (MTE-)                       
    318 Mouse: 2810484M10Rik (67247, AK013440) 
Human: FLJ20605 (54996, BC015829) 
 
           |-50      |-40      |-30      |-20      |-10      |1        |11       |21       |31       |41       
Mouse     TCCTCCCACACTCC  CGGAGCCT  TAAGCCCCGCCTCGACACCGCGGC  CCAG T AGC  CCAGGCCTGCCCGGG  ATAGTGTAACATG    GGTTCC  TCCAGCTCTACG
                          (BRE+)                            (INR+)                      (MTE+)     (DPE+)             
Human     CTCTCGCCTGCCCGGATC  CTTAAGG  GCCTCCTCGTCCTCCCGGTCT  CCGG T CGC  TGCCGGGTCT  GTGCGCCGGTCCG  CGCCCGCCCTCGCTCTGCCATGG
                             (TATA+)                        (INR+)                 (MTE-)                           
    319 Mouse: Ndufa10 (67273, AK013675) 
Human: NDUFA10 (4705, AI815873) 
 
           |-50      |-40      |-30      |-20      |-10      |1        |11       |21       |31       |41       
Mouse     GGTGACGTCATGGCA  GCGCGCCG  CGGACGAGCTAGGACCCCGTCGCGTT  C A TGTCCC  TCTGGTCCTTGAGCCGGCGC  AGACGG  CGAAGTCATGGCCTTGA
                           (BRE+)                              (INR-)                       (DPE+)                  
Human     CGTGACGTCACGGCA  GCGCGCCG  GCCGCGAGAGAGGGCCCCGTCG  CGACC G CG  TCCCCTTGGGTCCTTG  ATCCTGAGCTGAC  CGGGTAGCCATGGCCTTG
                           (BRE+)                          (INR-)                       (MTE-)                      
    320 Mouse: Pigc (67292, AK016394) 
Human: PIGC (5279, NM_002642) 
 
           |-50      |-40      |-30      |-20      |-10      |1        |11       |21       |31       |41       
Mouse     CGGGACGCTTTGGCCGCCGAGTGCTGCGGATCCGGAAGTGCTCCTCCACA   G TAGTCCC  CTTCCAAGCCGG  CGTCCCGGATGTA  GAAGTAGCTAGGCGGCC
                                                              (INR+)                   (MTE-)                     
Human     GGGTGCCCTCGCTAAGGAGATTGCGGCGGACCCGGAAGTGCTTGGCC  ACA G TCGC  AGCCCCGGCGCCCC  GAAGCGGGAAAAA  GGCTGGGTGCCGCCGTCC
                                                           (INR+)                     (MTE+)                      
    321 Mouse: Unc50 (67387, AK003273) 
Human: UNC50 (25972, AL080115) 
 
           |-50      |-40      |-30      |-20      |-10      |1        |11       |21       |31       |41       
Mouse     GAAGTGCGTGTCCGT  AACGTAAA  TACAGAGCGCCGCAAGTGCGGGC  TACG T AGC  GGGAAGGGAAGTGGCC  CCAGCGGCGGCTG  GGCTCGGCTGCCCTGGG
                           (BRE-)                           (INR-)                       (MTE+)                     
Human     GCGGCGGAAGT  CGGTTCCC  GTGACGCGGCGCGCCCCAAGGGCCGGC  TCCG T TGA  GGGAAGGGAAGCCCGCCC  GGTGGCGGCTGGG  GTCGGCTGCTGGGAG
                       (BRE+)                               (INR+)                         (MTE-)                   
    322 Mouse: Cabc1 (67426, BC030937) 
Human: CABC1 (56997, T04915) 
 
           |-50      |-40      |-30      |-20      |-10      |1        |11       |21       |31       |41       
Mouse     GAGGCGGGGCAAGCGGAGCGCGGCGTGCGCACCCGGGCTAGAAG  GTAAAC A G  AAGCGGAGAGTGT  GCGGAGCGCTCGC  AGCGGGGCGAGCGCGCGGAGGC
                                                        (INR+)                    (MTE-)                          
Human     GGGCGGGGCTTGTGAGCTGGGGCTCCCCCGGAGCGGGCGAGTTGGTAA  AC A GATCC  GGAGCGCGTGGCGGGCG  TCAGCGCGGTGGC  CAGCGCGCAGAGGC
                                                            (INR+)                        (MTE+)                  
    323 Mouse: Pigm (67556, NM_026234) 
Human: PIGM (93183, NM_145167) 
 
           |-50      |-40      |-30      |-20      |-10      |1        |11       |21       |31       |41       
Mouse     GACAGCTGAGAGCCCGCGCGGGGCTGGAGGCCGGAAGTACCGTGGGGGCG   G GACTTGG  GGTGCCG  GAAGTGATCATTC  CTC  GGTTCT  TCCTGTCGGTGCT
                                                              (INR+)              (MTE+)        (DPE+)              
Human     GCGGCTGAGAAGACCGCGCGGGGCTGGAGACAGGTAGCAGTACGGGGGCG G GGCTTCATGCCGGATGTGATAGTCTGC  AGTCGT  TTCGGTTGGCAGCCTG
                                                                                         (DPE+)                 
    324 Mouse: 2310044D20Rik (67698, BC021428) 
Human: UNQ1912 (345757, AY359108) 
 
           |-50      |-40      |-30      |-20      |-10      |1        |11       |21       |31       |41       
Mouse     CGGTTGTCCCGGCAGGCTGCTGCCGCCGCCGCCGCCGAAGCCACCCGGC  G C AGTCTC  AGCCACCCGCTCTCCTCCGGAG  AGTCGG  CTCTGCGGTTCCGGC
                                                             (INR+)                         (DPE+)                
Human     CTTCCGGTTCTCCGGGCAGCTGCCACTGCTGTAGCTTCTGCCACCTGCCA   C GACCGGG  CCTCTCCCTGGCGTTT  GGTCAC  CTCTGCTTCATTCTCCACCG
                                                              (INR-)                   (DPE+)                     
    325 Mouse: Gpatc2 (67769, AK053781) 
Human: GPATC2 (55105, NM_018040) 
 
           |-50      |-40      |-30      |-20      |-10      |1        |11       |21       |31       |41       
Mouse     TGGTCGCTTCCGGTTTCCGGTTTCCGCCTCCAGCCGGCTTCCG  GAAGTTG C   TCTCAAAATGCTGAAGTCC  TGTGTGGAATTGG  CCGGGGCTGCGGGAGTT
                                                       (INR+)                          (MTE-)                     
Human     TGGTTGCTCCGGGCATCCGGTTTCCGCCGCCTGTCGGCTTCCGGAAGCT  C A TCTCAA  AATGCTGA  ACTGCTCTTTGGA    AGTCGC  CGGTGCTGTTGTAGTT
                                                             (INR-)               (MTE+)     (DPE+)                 
    326 Mouse: Arpc5 (67771, NM_026369) 
Human: ARPC5 (10092, NM_005717) 
 
           |-50      |-40      |-30      |-20      |-10      |1        |11       |21       |31       |41       
Mouse     GACGCCCCAGAGCGGAAGAGGGAAGTGAGTCGGACCGGGCTGGGCTCG  CT A AAGGA  GAGGCACCGCGGAGGGGCAGGCCAGCGTCGCGTCGCGATCCGGG
                                                            (INR+)                                              
Human     GAGGGAAGTGAATCA  GGCGCCGG  GTAGTGGGTTGCTGGGCTGGGCTTGCT G AG  GTAGAGGC  AGCGCCAAGAAGAGGCCTTTGCCGCTGGTCGGGATTGGG
                           (BRE-)                                  (INR+)                                         
    327 Mouse: Ubxd2 (67812, AK029431) 
Human: UBXD2 (23190, BC035594) 
 
           |-50      |-40      |-30      |-20      |-10      |1        |11       |21       |31       |41       
Mouse     TGACACAGG  GGCGTACT  TTTCTTGCCAAGCGCGCTCCGGTGCGCGTCCCC A GGGGGTGGCTGTCGGG  CGTACGGGCTGGC  TG  GGTCCT  CAGACCGGGGCC
                     (BRE-)                                                         (MTE-)       (DPE+)             
Human     GGTTGCGACTGT  GACGTGAG  GTGTTCTCGCGCGCGCTAGCGCGCGTCTCC G GGTGCCGCTGACGGG  CGTGCGCGCTTGT  GCGGAGCCGGAGGTGGGGGCC
                        (BRE-)                                                     (MTE-)                         
    328 Mouse: 1500041J02Rik (67876, AK006551) 
Human: FLJ13448 (80219, BC022453) 
 
           |-50      |-40      |-30      |-20      |-10      |1        |11       |21       |31       |41       
Mouse     AGCCGTCCCTCCAGGCCGGCCTGAGGGCGGCGCCTCGTTCCGGGTGCCCG C GTTGGCTACCGGCT  TCACAAAGATGAT  CATGGCAGCTCGGACCAGTCAG
                                                                                (MTE-)                          
Human     CGGGCTCCGGGCCCGACCAGAGGAGGGCGGTGCTGCAGGGCTGGTCCGGG A GGTGACGACCGGCTTCGG  AGAGTCTATCATG  GCAGCTCGGACTGGTCAT
                                                                                    (MTE+)                      
    329 Mouse: Uxs1 (67883, AK076166) 
Human: UXS1 (80146, AK027244) 
 
           |-50      |-40      |-30      |-20      |-10      |1        |11       |21       |31       |41       
Mouse     GGCGCGCGGGGGTGGGGCTGGTGCACGGGGGCGGGGCTCGGGCCGCCGC  G C ATTGTG  CTGCGGCTGGCCGGCGCGGCAGGCCCCGGACCCGAGCGGTTCC
                                                             (INR-)                                             
Human     CGGCGGCAGGAGGCGGGGCTCTCGGGGCCGCTGCCGCCGCCGCCGCCGC  G C ATTGTG  CAGCAGGCGGGCC  CCCGCGCGGTAGG  GCCCTGGACCCGCGCGG
                                                             (INR-)                    (MTE-)                     
    330 Mouse: Tceb1 (67923, AK011757) 
Human: TCEB1 (6921, AL832244) 
 
           |-50      |-40      |-30      |-20      |-10      |1        |11       |21       |31       |41       
Mouse     GGATTTGGTAG  CGATGTCT    CTTAAAT  GGGCCTGTTTTACTGCCCACAAC  G C ACTTCC  TCCAGACGCAAGTTACCGGC  AGATCG  CGTCTCGGCCTTGCCAG
                       (BRE+)   (TATA+)                          (INR+)                       (DPE+)                  
Human     GTTGCGTTCATAGGCT  GGCCTAC  T  ATAGGG  GAAAAGAGTAGGGCG  CTACG C CC  TACCTCCAGACGGAAG  TGAGCGACACACT  CTGCGTCCTCGCCTCACC
                            (BRE-)  (TATA+)                  (INR+)                       (MTE+)                      
    331 Mouse: Fbxo28 (67948, AK077653) 
Human: FBXO28 (23219, NM_015176) 
 
           |-50      |-40      |-30      |-20      |-10      |1        |11       |21       |31       |41       
Mouse     AAGTGACGCACAGGA  AGTGTCCC  G  ATCCCCT  TACATTGGAGGAAGGAA  TC A AACTC  CCAAGATGGCGGCGGCGTCTGAGGAGCGGATGGCTGAGGAAGGA
                           (BRE-)    (TATA-)                    (INR+)                                              
Human     GTGACGCACCGGA  AGTGTCCC  TGTTCCCCTTGCTGTGGGGGTAAGGAA  TC A AGCCC  CCAAGATGGCG  GCAGCGGCGGAGG  AGCGGATGGCAGAGGAAGGA
                         (BRE-)                               (INR+)                  (MTE+)                        
    332 Mouse: Mki67ip (67949, AK077770) 
Human: MKI67IP (84365, AV714163) 
 
           |-50      |-40      |-30      |-20      |-10      |1        |11       |21       |31       |41       
Mouse     CGCTTCCGA  GACGCGAA  GGAAACGTCACTTCCGAAGGCTCCGCGT  GGATT G AC  TTGTTCGGGTTCTC  CAGCATGGCTGGG  TTAGCAGGCCCCGCGAAGCC
                     (BRE-)                                (INR-)                     (MTE-)                        
Human     CGCCTCGGGGGAGCT  GGGAGCCC  GACGTTTCCGGGAGCGCCGCGTGGTTA G CGTCGGCGGCTTTTGGCATGG  CGACTT  TTTCTGGCCCGGCTGGGCCAAT
                           (BRE+)                                                    (DPE-)                       
    333 Mouse: 4921533L14Rik (68187, AK081262) 
Human: KIAA1411 (57579, BC030797) 
 
           |-50      |-40      |-30      |-20      |-10      |1        |11       |21       |31       |41       
Mouse     GGCCGCGGGCCGGGACCTGTTGAACGCAGGTAGCGCTCGGCGGC  CCGGTT C C  CTTGGGACCGACGCGACTGCGCATGCTCGCCGGCGGCTCTGGGCCGGG
                                                        (INR+)                                                  
Human     CGTGCGGCCGGGGACCTGTTGATCGCAGGTATAGCCGGCTGGCCCGGGCT C CCTCGGGACTGGGGCGACTGCG  CATGCTCGCTGGC  CGCGCTGGGCCAGT
                                                                                        (MTE-)                  
    334 Mouse: 1700073K01Rik (68226, BC025062) 
Human: MGC12458 (84288, AK090927) 
 
           |-50      |-40      |-30      |-20      |-10      |1        |11       |21       |31       |41       
Mouse     GGGCTTTCTCCGCCCGCGGGGTCCCGCAGGGTTCGTGCTGGATGTCTCCC G GG  CGACCGGC  TCC  GCACCAAGATGGC  GGAGGAAAGGGACGCGGAAGGGA
                                                                 (INR-)          (MTE-)                           
Human     CGCTGAGCAGGCCGGGACACCGCGGCCGAGGTTATCGTTAGGCATCTC  CC A GGCGA  CCGGCTCC  GCAGCAAGATGGC  GGACGAGAAGGACAGGGAAGGTA
                                                            (INR+)               (MTE-)                           
    335 Mouse: Golt1a (68338, BC024448) 
Human: GOLT1A (127845, NM_198447) 
 
           |-50      |-40      |-30      |-20      |-10      |1        |11       |21       |31       |41       
Mouse     GGGGAATGTGGGAGGGT  GAGAGTT  AATGATTTACCTGCGGCTCTGTCCCT G TTGCCAAGGGTCAGCCACCCTGGAACATGCTCAGGCTCGGTCCTCTGGG
                            (TATA-)                                                                             
Human     GGAGCGGGGGCTGGAGG  GAGAGTT  AATGATTTGCCACAGGCTCATTTCG  C A ACTTAA  CCAAGGG  TCAGCTTCCCGTG  ACCATGTACCAGCTGCGTCCTCT
                            (TATA-)                            (INR-)              (MTE+)                           
    336 Mouse: Arl10b (68724, NM_026823) 
Human: ARL10B (127829, BE070207) 
 
           |-50      |-40      |-30      |-20      |-10      |1        |11       |21       |31       |41       
Mouse     CGGGCGCTGGCCTAGGCCGCGCGCGAGACTGGAGCGCGCTCGGGTGCGAG C AGGACGCGGCCCGGCCCGCGGGAGGGCGGCGGCGGCGGAGGAGGAGGTG
                                                                                                              
Human     CCCCTGGCCCCCCGGCTCCAGCGAGGGAGGAGCGCGCGCTCGGGTGCGAG C AGGACACGGCCCGGCCGCGAGGGAGGGCGGCGGCGGCGGCGGAGGAGGA
                                                                                                              
    337 Mouse: 1110060O18Rik (68818, NM_026846) 
Human: LOC130617 (130617, AI858102) 
 
           |-50      |-40      |-30      |-20      |-10      |1        |11       |21       |31       |41       
Mouse     TCTACTGTTGCCCGGGATGGACCCGCCCAACGTGATGACGTCAGAGCGCG C GAGCCCTCGGGAGGAGG  AGTAGGGGCTGCG  GGCCGGCGGACGCTCAGGC
                                                                                   (MTE-)                       
Human     CCCGGAGA  CTGCGCCG  GACGCGCCAGCCCTGCGGGATGACGTCAGCGCGC C GCGCGCGCCGAGGGA  GGAGCGGGCGCCG  GGGGCCGGCTGGCGCGGGGGC
                    (BRE+)                                                         (MTE+)                         
    338 Mouse: Pdcl3 (68833, BC005601) 
Human: PDCL3 (79031, CN338231) 
 
           |-50      |-40      |-30      |-20      |-10      |1        |11       |21       |31       |41       
Mouse     GGAGAAGACCGGAGGGGGCGGTGCCGCGCGTGCGCGGAGAGGAGCGGAG  G G GCTGGG  GCAGAGGGCTGGTTTGAG  TGACTG  AAGGCAAGATGCAGGTGAG
                                                             (INR-)                     (DPE-)                    
Human     GGGGGAAGA  GGCGTGGC  GGCGCTGTGCGCGTGCACAAAAGAGAGCTGAGG G GCGGGGGCGCTGCGGC  ACAGCTGGTTTGA  G  CAACTG  AACTGGAAACAAG
                     (BRE-)                                                         (MTE+)      (DPE-)              
    339 Mouse: 1500032H18Rik (69028, BC020137) 
Human: LOC129531 (129531, CR608207) 
 
           |-50      |-40      |-30      |-20      |-10      |1        |11       |21       |31       |41       
Mouse     AAGGTTTTTTTTTTTTTTTGTCCCGGCCCATTCACAACGCGCCATAGAAG A CAGCGCAGGCGCAGCGGCGTCA  CGCGTGCGCTTCC  TGGAAGCGCAGTGG
                                                                                        (MTE-)                  
Human     GCATTTTTGTTCCGGCGAAATCCCTCCCACTCAGGAAAGTCCCTAGAAAG A GAGCGCAGGCGCCTGGGTATCA  CATGACCACTTCC  CGGAAGCGCAGCAG
                                                                                        (MTE-)                  
    340 Mouse: Pycr2 (69051, AK007535) 
Human: PYCR2 (29920, NM_013328) 
 
           |-50      |-40      |-30      |-20      |-10      |1        |11       |21       |31       |41       
Mouse     CGTGGCCAACCGGAGGCTGCG  TATTTGG  CGGTGGATGCTCTGGG  ATACTT G G  GGCCTCTCTAGCAGT  TCATCTGGTGCAT  CCCGGTTTGAAAGCTGTTCC
                                (TATA+)                   (INR+)                      (MTE+)                        
Human     CAGCCGCCCGGGAGATATCCGCCGGGGGAGAATAGGGTTGCACCATC  CCA G AAGC  TGCTGTTAGCTCGCCG  GTCCTCGGCACGC  CGCCCGTTCGCCCCTG
                                                           (INR+)                       (MTE-)                    
    341 Mouse: Gmppa (69080, AK007463) 
Human: GMPPA (29926, CN309975) 
 
           |-50      |-40      |-30      |-20      |-10      |1        |11       |21       |31       |41       
Mouse     CGAAAGAGCTCTCGCGGGT  TGTAAGG  AGACACGTACGCGGAAGGAGCAA  G C AGTGGC  AGGCGGC  AGAGCTGGCGTAA  AGGGAGCTAGTGGTAAAAGGATC
                              (TATA+)                          (INR+)              (MTE+)                           
Human     CGAATGAGTTCTCGCGACTTGC  GAGAAGA  CACGTGCGCGGAAGGAGCTT  G C AGTAGC  GGGCGGC  AGAGCTGGAGTGA  AGGGAGCTAGTGGTAAAGGGAGC
                                 (TATA+)                       (INR+)              (MTE+)                           
    342 Mouse: 1810031K17Rik (69171, AK029090) 
Human: C2orf24 (27013, AF151815) 
 
           |-50      |-40      |-30      |-20      |-10      |1        |11       |21       |31       |41       
Mouse     CCCCTCCCCTCAGCG  GATAAAC  AAACACCAGCGCGTGACTTTAA  TGACTT C A  AAGCAGCTTCACCAACTTCGAAA  AGTTGT  TTCTTCCTTCTTGGAGCGT
                          (TATA+)                         (INR+)                          (DPE+)                    
Human     TCCCCTCAGTGGGTA  AACAAAC  ACACACCAGCGCTTGACTCGAC  AGACTC G A  AAACAACATCTACTCAGAAA  AGTTGT  TTCTTTCCTATCTCCTTAAACT
                          (TATA+)                         (INR-)                       (DPE+)                       
    343 Mouse: Wdfy1 (69368, AK007215) 
Human: WDFY1 (57590, AK023415) 
 
           |-50      |-40      |-30      |-20      |-10      |1        |11       |21       |31       |41       
Mouse     CCTAAGCAACCGGCGCGTCCCCGCCCGGCGGTCAGCTGATGGGCCGCCCG C GGGGGAGGCCGCGGCTCGAACATGGCGGCGG  AGATCC  ACTCCAGGCCTC
                                                                                             (DPE+)             
Human     GAAACCGGCGCGTCCCCCGCCCGCCCAGGCGTCAGCTGATGGGC  TGCCTG C C  GAGGAGGCCGCAGCAGTC  GCCGCGCGAACAT  GGCGGCCGAAATCCACT
                                                        (INR-)                         (MTE-)                     
    344 Mouse: Mrps9 (69527, AK049465) 
Human: MRPS9 (64965, NM_182640) 
 
           |-50      |-40      |-30      |-20      |-10      |1        |11       |21       |31       |41       
Mouse     CTGCGCCTGCGCAGTCTCCAGGCTCCACCCCTTTCCCCTCCCGCCAG  GAT G TTTC  TTACGGCCAACATGGCCGCCCCCTGTG  TGTCCT  GCGGCCGAGTGC
                                                           (INR-)                              (DPE-)             
Human     TAGCGCCTGCGCCGTCTCTAGGCCCCGCCCCCTCACCCCTCCG  GTCCTGG A   GCTCCCACAGCTAACATGGCGGCGCCCTGTG  TGTCCT  ACGGCGGAGCAG
                                                       (INR-)                                  (DPE-)             
    345 Mouse: 2310061I09Rik (69668, BC019430) 
Human: MGC12981 (84317, AK054693) 
 
           |-50      |-40      |-30      |-20      |-10      |1        |11       |21       |31       |41       
Mouse     TGGGATGTCACGTGG  TCTAAGA  CCGTCCTGATTAGTTAGTCCGTCTGCGG C TTCACCGGTGCTTGAGCTGGTCAGGCAGCAGGCTAGCGAAGAGTAAAAG
                          (TATA+)                                                                               
Human     GAATGTCACACGTGACTTAGGAC  CGCAGCT  AATAAATATGTCCGC  CCAGG C TT  CTCCATCCTCGTCCTCCCCCCACCACCGCCGTGCAGAATGCAGTTTT
                                  (TATA-)                  (INR+)                                                 
    346 Mouse: 2810422O20Rik (69962, NM_027279) 
Human: MGC9084 (92342, BC008679) 
 
           |-50      |-40      |-30      |-20      |-10      |1        |11       |21       |31       |41       
Mouse     CTCGCCCCCA  AGCGTCGG  GGCCCTCTCTAGCTATTGGAGGACT  CATCTCG T   TGGTGTTCGCCGTAGGCTG  AAGGCGAGTTTCT  CCGGTAGTTTGTTCGCC
                      (BRE-)                             (INR-)                          (MTE-)                     
Human     AAGGCCGGAAA  GAAAGTCG  GGCTTCTCTAGCCCCTGGAGGACTCGAC  TCA C TGGT  GCGCGATTTAGGTCCGG  AGGAGGCGTTGTG  AGGTGAGCTTTTTCA
                       (BRE+)                                (INR-)                        (MTE-)                   
    347 Mouse: Spata3 (70060, BC048516) 
Human: SPATA3 (130560, AK093385) 
 
           |-50      |-40      |-30      |-20      |-10      |1        |11       |21       |31       |41       
Mouse     TGACCTCACAGGAGGTAT  GGGTGACT  CTTGTCTGTGGGACCTT  GCTGTGG T   CAAAGATCAGAGACATACCCACAGGGGAGACTGCCTACCTAGGGAGGGA
                              (BRE+)                     (INR-)                                                   
Human     TGACCTCACAGGA  GGTGTGGG  TCACTGTTCTCTGTGGGGCCTGGGTC  CAG C TGGA  GGCCCAAGCCAGGCTCCTAGGGCAGAGGGCAAACGGCCCCTCCAG
                         (BRE-)                              (INR-)                                               
    348 Mouse: 1110020A09Rik (70397, AK005075) 
Human: FLJ20533 (54968, BC002748) 
 
           |-50      |-40      |-30      |-20      |-10      |1        |11       |21       |31       |41       
Mouse     TCTGCGCACGCGCGCGCACCAATCCGGCCGCGTGTTCCGTTTAAGGGGCG G AGCCTCATCGCTCTTGTGGGGGCCACTGTCGAGCGGAGTCGCTTTTGGG
                                                                                                              
Human     GATTCTGCGCACGCGCAGGCAGCCCAGCTGCCAGTCAGGCGTCCCGGGCT G   GGCATGCG  CCACTTGTGCGGC  AGTCGG  GTGGGAAGCCGTGTCTCGCAGT
                                                               (INR-)                (DPE+)                       
    349 Mouse: 2610300B10Rik (70454, AK053363) 
Human: dJ383J4.3 (91687, BC007923) 
 
           |-50      |-40      |-30      |-20      |-10      |1        |11       |21       |31       |41       
Mouse     CGAGTCGGCCGCAGGGCAAGTCGGGAAATGATTTTAAACTGGAGGGCGTG G CG  TGTGTGGA  AAAGCGCCTAGACGAGCG  GAACTT  TCAAGTCTACCTTTG
                                                                 (INR-)                     (DPE-)                
Human     TGCGTGGGGCCGCAGAGCGAGTCGGGAAACGATTTTAAACTGAAGAGGCG G CGGAGGGCCGAATTCCCTTTT  CTCAACGGCTTGA  TTTCAGAGCTGGGCT
                                                                                       (MTE-)                   
    350 Mouse: 2610205H19Rik (70456, M13094) 
Human: DKFZP564B167 (25874, AL035304) 
 
           |-50      |-40      |-30      |-20      |-10      |1        |11       |21       |31       |41       
Mouse     AGGCCAGCCAATTAGAGCAGGCC  GTTAAGC  TTGCGCAACGCCTCGCCCGC C TA  GCTCTAGC  GGCCCCAGCACGG  CGACCT  CTGTAGCACCTGTTGCCATG
                                  (TATA+)                          (INR-)                (DPE-)                     
Human     GGCGGGCCA  ATCATGGC  GGGCAGCTCGGCCTGCGCAAGCGCGCTGCCA  GC A GGGCG  CAGCGCAGACTTG  GTGAGGTGATTAT  TTTGGCACCTGTTGCCAT
                     (BRE-)                                   (INR+)                    (MTE-)                      
    351 Mouse: 5730454B08Rik (70579, AK041148) 
Human: KIAA0663 (9877, BX484460) 
 
           |-50      |-40      |-30      |-20      |-10      |1        |11       |21       |31       |41       
Mouse     CTGGCCCAGCCTTAAA  GGCGAGCC  TGTAGCCGGGCGCCATCTTTGACGCT G   GCAGTCCT  GGGTTTCTGC  TTGTTCGGCTGCT  GTGAGGACGGCGGGCGAC
                            (BRE-)                               (INR+)                 (MTE-)                      
Human     CCGGCCGGGCCTTAGAG  CGGAGCCT  GTAGCCAGGCGCCATCTTTGACGCT G   GCAGTCTT  GGTTTTCTG  CTAGTGCTGCTGC  TGCTGGGAGGACGACGGAC
                             (BRE+)                              (INR+)                (MTE+)                       
    352 Mouse: Gulp1 (70676, BC032154) 
Human: GULP1 (51454, AK055718) 
 
           |-50      |-40      |-30      |-20      |-10      |1        |11       |21       |31       |41       
Mouse     GCCCTCAGCAACCGGGAGTCCCGACCCAGCCCCTGCCGTCGCCAGCA  CCA G TTTG  GGCTTCAGC  GGATTAGAAGGAG  GGAGA  GGACCA  GAGCCCTAGGGT
                                                           (INR+)                (MTE+)          (DPE-)             
Human     GGGAGCGACCCGGAGTCCCCAGCCCCGCGTCCCAGCTGCCGCCAGCG  CCA G TTTT  GGATTCGGC  GGATTAGGAAGAG  GAGGGAGGGGGGAGAGAGCGCGA
                                                           (INR+)                (MTE+)                           
    353 Mouse: Als2cr3 (70827, AK014647) 
Human: ALS2CR3 (66008, AB038952) 
 
           |-50      |-40      |-30      |-20      |-10      |1        |11       |21       |31       |41       
Mouse     TTGGGAGAGTGGGTCTCC  TTTTTAG  CTTCCCTAGCTGGGTGGGGGCTG  CC A TTGAA  CCGGCGTCACTGCAGCTCCAA  GGTCGC  CCGGTGATGATGTGAGA
                             (TATA+)                          (INR-)                        (DPE+)                  
Human     CGCTGGGAGAGTGGCTCTCCTTTGGCTTCCCCAATTGTGTGGGGGCTG  CC A TTGAC  CCGGTGTCGCCGCAGAACCGA  GGTCGC  CGAGTGATGATGTTGTG
                                                            (INR-)                        (DPE+)                  
    354 Mouse: 4633402D15Rik (70829, AK082581) 
Human: FLJ10996 (54520, BC028609) 
 
           |-50      |-40      |-30      |-20      |-10      |1        |11       |21       |31       |41       
Mouse     AGTGTCAGCTCCTGTGAA  GGAAGTTT  CCGCTTTGCACCCCACCCGGATC  A C AGTTCG  GTTGTAGGGCCCGCT  CTCTCCGGCTCCA  CATCAGCGCATCTGG
                              (BRE+)                           (INR+)                      (MTE-)                   
Human     GGCGGCGG  CGGCGGCA  GAGGGAGTTTCCGCTTTGTACTCCACCCCGGTA  G C AGCTCC  GCGGCAGGG  ACAGCTTCCTCC  G  GACGC  TTGGCGGGCTTCGCTC
                    (BRE+)                                     (INR+)                (MTE+)    (DPE+)                 
    355 Mouse: Sntg1 (71096, NM_027671) 
Human: SNTG1 (54212, NM_018967) 
 
           |-50      |-40      |-30      |-20      |-10      |1        |11       |21       |31       |41       
Mouse     CTCTCTGTCCCCCGTCCCTGTGCGCGCTTGTGCGCGCGCCCGTGTTCGTG C GTTCTTTTTTCAT  TTCTACCATTAGG  AAACCTACTCAGAGCCGCTGTGA
                                                                               (MTE-)                           
Human     CTCTCTCTCTCTGCCTCTCTC  GTGCGTG  TATGTGCGCGCCCCTGTGCGTG C GTTTTCTTTCATTTCTGGCA  TTAGGAAACTCGT  GCAGGGCCACTGAAAA
                                (TATA-)                                                 (MTE-)                    
    356 Mouse: 4930563E19Rik (71227, AK016977) 
Human: FLJ25955 (164781, AK127882) 
 
           |-50      |-40      |-30      |-20      |-10      |1        |11       |21       |31       |41       
Mouse     GTACCCGCCCAGAAAAACTTGCGCACGCGCCTTAGCGTCCTACTGCTGTT T G  TTAGTTTC  CATGGCTAC  GAAGCCCCTCAGG  GTAGGAAAGTAGGAAAAT
                                                                (INR+)                (MTE+)                      
Human     AGGACCGCGCGGTGCAACCTGCGCATGCGCACCCGCGTCCCGCTGC  TGTT T AGC  CGTTTCCAAGGCTACGAAGC  CCATCGGCCGGGG  ATAAGAGAGCAAG
                                                          (INR-)                           (MTE+)                 
    357 Mouse: 5630401D24Rik (71449, AK050492) 
Human: KIAA0859 (51603, NM_015935) 
 
           |-50      |-40      |-30      |-20      |-10      |1        |11       |21       |31       |41       
Mouse     CCGCCTGCCCAATCACGAGACCTCTTACCCGGATATGGTTTCTAGGTTC  T G AGTGTA  GCTGCGGGA  GTGTGTGGCTGCT  GCACGTGGGGCGCGGACCGAG
                                                             (INR-)                (MTE-)                         
Human     CCGGCCGACGAATCACGA  GGCTTCGC  ACCCGGATATGGTTATGGGCTCGG A   AATCTAGT  TCGGGAAAAGT  GTGAGGGGCTCTT  CACGTGGGGAAGGAACA
                              (BRE-)                             (INR-)                  (MTE-)                     
    358 Mouse: Stk11ip (71728, NM_027886) 
Human: STK11IP (114790, BC034051) 
 
           |-50      |-40      |-30      |-20      |-10      |1        |11       |21       |31       |41       
Mouse     GCTTTCCGGTAGGTCCTTTGCCTTCCGGGGCGGGACTTCCTGCCCTAAAA G AGCCCGGCTGCCAAAG  TGATTGGAGTGCG  AATTACGCGGGCCCATGGGC
                                                                                  (MTE+)                        
Human     CCTTGGCTCGATTTTCTTCCGGGGCGGGACTTCCTTTCCATCATTGATAG G CGCCGGGCAGCTGAGCTGGTAGGAGGACC  AGACGG  GGAGGTTCGGTATG
                                                                                           (DPE+)               
    359 Mouse: 2310007B03Rik (71874, NM_172411) 
Human: FLJ22671 (79919, AK026324) 
 
           |-50      |-40      |-30      |-20      |-10      |1        |11       |21       |31       |41       
Mouse     GTGAAGCATGAGGTACACACCCCCTCTTTCGGGTTAAGCCATTG  TGTTTG G G  GCCCAAGATTCAGGAAGG  AGTAGAGACTGAG  GAGAGTGGTGAGTAGGG
                                                        (INR-)                         (MTE-)                     
Human     CAATATTTCTCCAGGGACCCCAAGCCTGGCTGGGACAGGGGCCGTGCCTT G   GGCCTAAG  ATGAG  AGAGCTCTTCTGA  AGGGAGGGAGGAGGGTGGTAACC
                                                               (INR-)            (MTE+)                           
    360 Mouse: Nqo3a2 (72017, BC016266) 
Human: NQO3A2 (51706, AJ710549) 
 
           |-50      |-40      |-30      |-20      |-10      |1        |11       |21       |31       |41       
Mouse     CATAGACCACGCCCCGGAGAGCTCCGCCTCCGGGCCCGCCCCGCGGC  GCA T TGTG  GGATCTTTCCGCCCCACCGGAGCG  GGACGC  ACCTCTAGGTCATGG
                                                           (INR-)                           (DPE+)                
Human     CCCGGCCCCGCCCCCGGCCTGCCCCGCCCTCCGACCCGCCCCGCGGC  GCA T TGTG  GGATCTGTCGGCTTGTCAGGTGGTGGAGGAAAAGGCGCTCCGTCA
                                                           (INR-)                                               
    361 Mouse: Lypdc1 (72585, AB041649) 
Human: LYPDC1 (116372, AK123029) 
 
           |-50      |-40      |-30      |-20      |-10      |1        |11       |21       |31       |41       
Mouse     AGGGGTGGGGGCAGAAGA  GGCGAGAC  TTTTTTGGGTGCTCCGGATCGCCA   G TAGTTCT  TCAAGCCTCAGCAGC  CAACTC  CTCCGGAGGCGCTGCGCTCCG
                              (BRE-)                            (INR+)                  (DPE-)                      
Human     TGGGAGGTGGGGAGAAAA  GGCGAGAC  TTTTGTGGGTGCTCCCGATCGCCA   G TAGTTCC  TTCAGTCTCAGCCGC  CAACTC  CGGAGGCGCGGTGCTCGGCCC
                              (BRE-)                            (INR+)                  (DPE-)                      
    362 Mouse: Angptl1 (72713, AK012888) 
Human: ANGPTL1 (9068, NM_004673) 
 
           |-50      |-40      |-30      |-20      |-10      |1        |11       |21       |31       |41       
Mouse     ACGTAATATGTGGGCTGAACTTGAGTCTACTGAGAACGAGAGGGAA  TCAC T CTT  CGGACTGTGTGGACAG  TCTGTCAGCTGCG  GCTGGTTTCTGCACATT
                                                          (INR+)                       (MTE-)                     
Human     TGATGTAATATGTGGGCTGAACTTGAGTCTACTGAGAAAGAGGGAATCA  C T ATTCAG  GGGTACTGTATATA  CAATCTGGGTCAG  CTGCAGCTGGTTACTG
                                                             (INR+)                     (MTE+)                    
    363 Mouse: Ccnt2 (72949, BC054122) 
Human: CCNT2 (905, NM_001241) 
 
           |-50      |-40      |-30      |-20      |-10      |1        |11       |21       |31       |41       
Mouse     GTGGCCCACCCTACGGAGAGCACATTGGGGCGGGGCCTCGACACATTGGC G C  GGAGTCCC  GGAGGCAGGCGGGGAGGGGGCGGGCTCAGGGCGCGGGCGG
                                                                (INR+)                                          
Human     TGGTGCGTGGTCTACGGCGAGCGGAGTGGGGCGGGGTCGCGCGCCTTGGC G G  GGAGTCCG  CGAGCCAGGAGGGGCGGGGGGTGAATGAAGGAGCGGGCGG
                                                                (INR+)                                          
    364 Mouse: Cnih3 (72978, AK013789) 
Human: CNIH3 (149111, CR595048) 
 
           |-50      |-40      |-30      |-20      |-10      |1        |11       |21       |31       |41       
Mouse     CTGGAGCCGGCGAGGGTG  GGGCGCCG  CGAGGGAGGGGTCCCAGCGCC  GCA T TCCT  CGCGGCGCCCGCGGCA  GCAGCAGAAGCAG  CAGCAGCAGCAGGTGG
                              (BRE+)                         (INR+)                       (MTE+)                    
Human     AGTCGAGGGTGG  GGCGCCGA  GAGGAGGGAATCCCGGGTCGCACCGCT  ACA G TTCT  CGCAGTGGCAAAGGCGGCGGCGGCGGCGGCGGCAGCGGCAGCAGC
                        (BRE-)                               (INR+)                                               
    365 Mouse: Insig2 (72999, AK050394) 
Human: INSIG2 (51141, NM_016133) 
 
           |-50      |-40      |-30      |-20      |-10      |1        |11       |21       |31       |41       
Mouse     GGCGGGGGTGAACTGGGGGTCTCCGGGCAGAGCTCAGGTGAGCT  TATTTG G C  CGGGGACGGCTCGA  GGACGGCGGTGGC  TTTTGGGAAGAGGAGCTGTTT
                                                        (INR-)                     (MTE-)                         
Human     GGCGGGGGTGAGTTGGGGGTCTCCCGGCGAAGCGCGGGTGACGTGGTGCT G AGGAAAGCGGCCTGA  GGAGGAGGGTGGC  CCTTGGGAAGAAGAACTACTT
                                                                                 (MTE-)                         
    366 Mouse: Insig2 (72999, BC024411) 
Human: INSIG2 (51141, NM_016133) 
 
           |-50      |-40      |-30      |-20      |-10      |1        |11       |21       |31       |41       
Mouse     CGTGAAGCCGCCGGGCGGCGCAGGCGCAGTGCGGCTCAGGCGGA  GCTGTG G G  AGGGGAGTAGGTCGGGGGCCGGTCCCTGA  AGATGG  CGGAGGCGGGGGT
                                                        (INR-)                                (DPE+)              
Human     GCGCGGCCTCGTTGGCCGCACAGGCGCAGTGGAGCTCGGGCGGAGTTGTG   G GAGTGGA  GGAGGAAGAGGCGGTAGGGGGTACGGGGGCTGGTCCCAGAAG
                                                              (INR-)                                            
    367 Mouse: 3110009E18Rik (73103, AK014026) 
Human: LOC130355 (130355, BC047365) 
 
           |-50      |-40      |-30      |-20      |-10      |1        |11       |21       |31       |41       
Mouse     AGCGCTCCAACCCTTCCCGGACCCAAGAGGGAGTTTATTGCGCACGCGCG G GGAGGAGCCGCGGGGCACGTTG  GGAGCAGGTGGGA  CGCGTTTCGTTTTT
                                                                                        (MTE+)                  
Human     CGCGGCGCTAGCCCCGCCCGAGCCCACGTGGGACCGCTTGCGCAAACGCC G   GGAGTAGC  CGAAGGGG  ACGCCGGGAACAG  GTGGGGACAACGGAAGTAGG
                                                               (INR-)               (MTE-)                        
    368 Mouse: 1700066M21Rik (73467, AK006906) 
Human: FLJ38973 (205327, AK096292) 
 
           |-50      |-40      |-30      |-20      |-10      |1        |11       |21       |31       |41       
Mouse     CCTCGCTGCCGGGTGAGCGGCCCTGGCGGCTCGAGCCCATGCTGGGGTCG C GG  CGTCTGCG  GTCCCCCGCGC  TGGTGCTGCTGCT  GCTGCGGCCGCTGCT
                                                                 (INR-)                  (MTE-)                   
Human     CACCCTCCACCTCCGAACCGCTCTCGCGGCGGCGACCCATGTGGGGGT  TC A GGCTC  CTGCGGTCGCCGC  CGTTGCTGCTCCT  GCTGCCGCAGCTCGGAAT
                                                            (INR+)                    (MTE-)                      
    369 Mouse: Als2 (74018, AB053307) 
Human: ALS2 (57679, NM_020919) 
 
           |-50      |-40      |-30      |-20      |-10      |1        |11       |21       |31       |41       
Mouse     CCCGCCGCCGGAGGCCCCGCCCCTACCTGGAGGCCCCGCCCCCA  TGAATC A G  CTGATCTCGCGTACCCGCGGTGC  AGTCGG  GCTCGCGCCGGGAGAAGAG
                                                        (INR+)                          (DPE+)                    
Human     TAGTCGCCCTGCAGCCCCGCCCTGTCCGGAGGCCCCGCCCTCCA  TGAATC A G  CTGATCCCGCGGACCCACTG  GGTTGC  CAAGCTCGCGCCGGATGCGGAG
                                                        (INR+)                       (DPE+)                       
    370 Mouse: Npl (74091, AK088859) 
Human: NPL (80896, AW896603) 
 
           |-50      |-40      |-30      |-20      |-10      |1        |11       |21       |31       |41       
Mouse     GTGGTCCCGCAGCCCCTGA  GTTAAAA  GCGCAACCGCGCTGCTCAGA  GGAG A TCG  CGTCTCCAGTGCACA  GCAGCCTAGGTGA  GCGCGGCGCCACCGAGCA
                              (TATA+)                       (INR+)                      (MTE+)                      
Human     CGAGCCGGGCGGCCGCGGA  TATAAAG  CGCGGCGGCGGCTGCCGGGC  GGAG C GGC  TGCACGGACA  AGAGCGGAGGCCT  GGGTGAGCGCGGCCCGCGACGGC
                              (TATA+)                       (INR-)                 (MTE+)                           
    371 Mouse: Abcb6 (74104, AK033668) 
Human: ABCB6 (10058, AK057026) 
 
           |-50      |-40      |-30      |-20      |-10      |1        |11       |21       |31       |41       
Mouse     TGGGAACCCTGGATAGATCTGGTGGGCGGGGCCGCTGCCGTAAAGGACCC A   ACATTTTT  GTGCGGCGGCTCCGAGCCTTAG  GGTCCC  AGCTGCTAGCGAG
                                                               (INR+)                         (DPE+)              
Human     GGGCTGTGGGCGGGGCTACAGCCGTGGAGCGCGGTGCGAGTCCAACACCG A   GCATTCCC  GTGGGCCT  GCAGTTGGCAGGA  G  GGTCCC  GGGCCCAGAGCCA
                                                               (INR+)               (MTE+)      (DPE+)              
    372 Mouse: Iqwd1 (74106, AK004618) 
Human: IQWD1 (55827, AK093970) 
 
           |-50      |-40      |-30      |-20      |-10      |1        |11       |21       |31       |41       
Mouse     CGCATGGTGCTGGTGCCGCTCGGGTGTTGATCGGCCTGTCCCCTCCCTCT C TTCCCCTCCCCACCCCCCGCGGT  GGTCTC  CCCTTTCCCACCCCAGCCCC
                                                                                     (DPE-)                     
Human     CGGATGGTGCCGGTGCGGCTC  GGGTGTT  GAAACGGGTGTCCCCTCCCCCT C CTCCCCTCCCCCACGCGGTGGTCTCCCCTCCCACCCGGCTCAGGCAGAG
                                (TATA-)                                                                         
    373 Mouse: Actr3 (74117, NM_023735) 
Human: ACTR3 (10096, BC044590) 
 
           |-50      |-40      |-30      |-20      |-10      |1        |11       |21       |31       |41       
Mouse     CCGGCCGGCCAGCCTGCTGGGCTTGCGGAAGTACTAGCCGACTGC  TCACT C GC  TCCTGCTCGGGCTTCCC  ACAGCTCCCTCCC  TTCCTCCCTCCCTCCCG
                                                         (INR+)                        (MTE+)                     
Human     CCTGCCTGGGTTGCGGAAGTGATAGCCGCCGACCGAGCCTGCTGCTTTCT T G  CTACTGCT  TCGG  CTTCCCGGCTACC  CCCC  GGACGG  TGAAGGCGGCCCA
                                                                (INR-)           (MTE-)         (DPE+)              
    374 Mouse: 1200013B22Rik (74137, AK034082) 
Human: SNARK (81788, NM_030952) 
 
           |-50      |-40      |-30      |-20      |-10      |1        |11       |21       |31       |41       
Mouse     CGTGGTCAGGACATTCCCCCTCGA  AACGTTT  TATAGCAATTCGGAACGCG G GGCTTTCTAGCGTGCTCGGGTGCGGCTG  TGACCT  CTGAGCCCGCGGCTC
                                   (TATA-)                                                  (DPE-)                
Human     GGCGTGTCCAGGGCCTCGGGCTCA  GGGCGTT  TATAACAACTTGGAAT  GCT G TGCT  TTACTGCGCGCTC  TGGTACTGCTGTG  GCTCCCCGTCCTGGTGCGG
                                   (TATA-)                   (INR-)                    (MTE-)                       
    375 Mouse: Slc35f5 (74150, AK004892) 
Human: SLC35F5 (80255, BC050096) 
 
           |-50      |-40      |-30      |-20      |-10      |1        |11       |21       |31       |41       
Mouse     CGTGGCCTGGCTGGCTGACCGGCCGGGGGCGGGGCCTGGGCCGCGGCC  GC A ATCCG  GGCCTCCAG  GACTCTCGCTGCC  AGCGCCGTCCGGTGGCCTGGGT
                                                            (INR+)                (MTE-)                          
Human     GCGGGGCCGCGCGGAGGGCAAGCCGGGGGCGGGGCCGGGGCCAGA  GCAGA T CT  CCGGGCCTCCGCGGCCATAGC  TGACTG  TGCCGTCCCTTCCCCTCACC
                                                         (INR+)                        (DPE-)                     
    376 Mouse: 1700019B16Rik (74229, AK029772) 
Human: C6orf33 (85315, AY424286) 
 
           |-50      |-40      |-30      |-20      |-10      |1        |11       |21       |31       |41       
Mouse     CCTCCTCCCGGCCGCTCTCGGGTGCAGAGCCCTCGGCGGTCGGGGAGGC  T C AGAAAG  AGCTCTCCCGGAGCTGG  AGTCGG  GAGTGCGGCTGGCGGAGCTG
                                                             (INR+)                    (DPE+)                     
Human     CCTCCTCCCGGCCGCTCCCCCGAGCCGAGCCCGCCGCGGTCACAG  CCACC C GC  GGGAAGCTCGTGGCCGGGACCCCGAGGCGGGAGCGCGGGCTGGGCCG
                                                         (INR+)                                                 
    377 Mouse: D1Bwg1363e (74241, NM_001001566) 
Human: CHPF (79586, AY358403) 
 
           |-50      |-40      |-30      |-20      |-10      |1        |11       |21       |31       |41       
Mouse     TGCCTCTCC  GGCGCTGA  GGAGGGGGCACTTCCGGGGGTCCTTCC  CCTTTA A C  CTCCTCCCCTTCGTC  CCTCTCCGCTAGC  TGACGGCTAGACCCCACCTC
                     (BRE-)                               (INR-)                      (MTE-)                        
Human     TCCCTCTCCCGCACTGAGGAGGGGGCACTTCCGGGGGTCCTTCC  CCTTTA A C  CTCCCCCTTCTCCTC  CCTCTCCGGTAGC  GAGAGCCCGAGACCCCACCC
                                                        (INR-)                      (MTE-)                        
    378 Mouse: 4930513F16Rik (74717, AK015780) 
Human: LOC128153 (128153, AK098591) 
 
           |-50      |-40      |-30      |-20      |-10      |1        |11       |21       |31       |41       
Mouse     GGAAGCCGGCTGGAGGCGGAAACCGGAAACCGGAAGCGACCATTCGCTCA C CTTAGCCTACCCTGACCCTTCCC  ATCAGCAGCTCGA  AATTCCAGGAGAA
                                                                                         (MTE-)                 
Human     GGAAGCCGACAGGCGGCGGAAACCGGATGCCCGGAGCAACCATTAGTTCT C CG  CGTTTACT  GCTTTC  CCCTTCCTATAAT  CCCCTCTTCCTACTCGCAGT
                                                                 (INR-)             (MTE-)                        
    379 Mouse: Slamf7 (75345, AK089525) 
Human: SLAMF7 (57823, AY358512) 
 
           |-50      |-40      |-30      |-20      |-10      |1        |11       |21       |31       |41       
Mouse     ACATAAAAACGTGAGA  TGACTCAT  TGTAACAAGTAAGACTCCTC  CTACTT A A  TCCCAGCTGAGGA  CAAGAGAGCTAAG  ACTACCAGAGTGCTTTCTGTTC
                            (BRE+)                        (INR+)                    (MTE-)                          
Human     ATTTAGCAA  TTACTCAA  TCTCACATGTCTGCGGCGTGACCCCTCCTGCTT C T  TTAAATAT  CAGCTGGGGAAGA  GGTCTG  AGTAATACCTAAGAGGGAAGT
                     (BRE+)                                       (INR+)                (DPE-)                      
    380 Mouse: Spp2 (75396, AK002814) 
Human: SPP2 (6694, AJ308099) 
 
           |-50      |-40      |-30      |-20      |-10      |1        |11       |21       |31       |41       
Mouse     AGTCAGCAGTAAATATTGACACCGGAATCAATCATCTCTACTGAGGTT  CC A GATTG  CTCCAGCCAGCCAGGGCAGCTA  GGTCAC  AGGTGACAAGAATAAG
                                                            (INR+)                         (DPE+)                 
Human     AGTCAGCAGAAAATATTGACCCCAGGATCAATTGTTTCTGTTTAAGTT  AC A GATTG  CTGCAGTCAAAATAA  GCAGCCAGTGTTT  GATAAAGACAGCTCCT
                                                            (INR+)                      (MTE+)                    
    381 Mouse: Jarid1b (75605, NM_152895) 
Human: JARID1B (10765, AK122752) 
 
           |-50      |-40      |-30      |-20      |-10      |1        |11       |21       |31       |41       
Mouse     GCGGAGGAGCACTGCGTGGGGTAGCGGCAAGACGTCGTCGGAGGA  AGGCT G AA  AAAGCCCTAGG  TGTTGCCGTTGCT  AGTAGAAGTCGGACCTGTCGCCC
                                                         (INR-)                  (MTE-)                           
Human     GGCGGAGGCGCAC  GGCGTGGG  CTTGCGGCGAGACGTCGTCGTCGG  AGGCT G AA  AAAGCCCAAGG  TGCTGCCGTTGCC  CGTA  CAACTC  GGACTTGCTGTTG
                         (BRE-)                            (INR-)                  (MTE-)         (DPE-)              
    382 Mouse: 2810421I24Rik (75619, AK035322) 
Human: KIAA0971 (22868, AK021557) 
 
           |-50      |-40      |-30      |-20      |-10      |1        |11       |21       |31       |41       
Mouse     CTGAGAAC  CGATGCTT  TTTGACT  TATAAAA  GAATCCGTAATACGTCA  CCA G ACGC  GCCGCTTGAGGAGGAAC  TGTCATGGCTGCG  CCTATGTGTAGTCAC
                    (BRE+)          (TATA+)                    (INR+)                        (MTE-)                   
Human     CCGCGAAC  ATATGCTT  TT  TGACCTT  TGAGGGAATCCGTAAGATCTCGTGA   G AAGCGCA  GCTTCTCGGG  GAAGCTGTCATGG  CTGCTCCTGTACGTAGTCA
                    (BRE+)     (TATA-)                            (INR-)                 (MTE+)                       
    383 Mouse: 5230400G24Rik (75734, BC059229) 
Human: C2orf33 (56947, AK026137) 
 
           |-50      |-40      |-30      |-20      |-10      |1        |11       |21       |31       |41       
Mouse     CTCGTCACATGACCAGAAACCTACTTCCGCTACGGCTCCTGGAAGGGG  TC A GTCCT  GGCCGTTCTCGCGTCTGTCCCGGCCCGTCGCGGCCGCTCCGCCG
                                                            (INR+)                                              
Human     CGCGTCACATGACCACGA  ACACTCTT  CCGCTACGGCTCCCAGAAGGGG  CC A GCCCG  CGCCTTTCGC  GCTTCTGCCCTGG  CCCTCTGCGGGCCGCTCCGCC
                              (BRE+)                          (INR+)                 (MTE+)                         
    384 Mouse: Mdh1b (76668, BC050786) 
Human: MDH1B (130752, BC033509) 
 
           |-50      |-40      |-30      |-20      |-10      |1        |11       |21       |31       |41       
Mouse     CCAGTTTTAGTACCCGCCGCGGTTGCCAGGAAACCGTGACGCCACGGTC  C C AGTTTC  TAGGTCT  TGCTAGAAATCAT  GGCCAAATTTGTGATCGCGGGTG
                                                             (INR+)              (MTE-)                           
Human     CGCCTTGCCCTTGGC  TGCGTGGT  TGCCAGGAAACCGCGAAGCCGCGGTCC C TG  CCTCTGAG  TCTCTCTCGACC  ATGGCCAAATTCG  TCATCGCGGGTGAG
                           (BRE-)                                  (INR-)                   (MTE-)                  
    385 Mouse: Sdccag8 (76816, AF250729) 
Human: SDCCAG8 (10806, AF161348) 
 
           |-50      |-40      |-30      |-20      |-10      |1        |11       |21       |31       |41       
Mouse     GGCAGGACAGCGGGACTAATCCAAGCGATGCAAAAGGGAAAGGGCGCGGC G CGCCAACCCGTCTCTTGGCA  ACAGCCTCAGGCC  GCTAAACTGTCGGAGT
                                                                                      (MTE+)                    
Human     GGGATGGAGGCGGG  ACTAAAC  CAGGCTGGGCTGGAGGAAAAGGGCGG  GGC C TGCA  AGCCTGTCTCTTG  GCAGCAGGCTCTG  GCAGCCAAACCGTCCAAGC
                         (TATA+)                             (INR-)                    (MTE-)                       
    386 Mouse: 2810047L02Rik (76843, AB095735) 
Human: RAMP (51514, NM_016448) 
 
           |-50      |-40      |-30      |-20      |-10      |1        |11       |21       |31       |41       
Mouse     CGTGACGTCAGTTT  GGCGCGGA  GTTTGGCGGCCGGGGCTCTTAGTGGCG  G G AGTTGG  AGGCGAAACC  GCGCCGCGTTGTG  GAGGGTGCAGACGGCGGCTC
                          (BRE-)                               (INR+)                 (MTE-)                        
Human     CGTGACGTCAGTTT  GGCGCGGA  GTTTGGCGGCCGGGGCTTACAGTGGCG  G G AGTTGG  AGGCGATAA  CGATTTGTGTTGT  GAGAGGCGCAAGCTGCGATTT
                          (BRE-)                               (INR+)                (MTE+)                         
    387 Mouse: 4930418P06Rik (76867, AK029751) 
Human: DKFZp547E052 (84236, AX775823) 
 
           |-50      |-40      |-30      |-20      |-10      |1        |11       |21       |31       |41       
Mouse     GGAATCGGCGT  CCGTGCCG  GAAGTTGAGTCTCCGGATTCGGCGT  CCAGAC G T  AGTGACGCACGAGCGCGGGAGTACGTGA  GGACGC  AAGCGGGTCTTAGA
                       (BRE+)                             (INR+)                               (DPE+)               
Human     CTCCCGCACCCGGCCCCA  GGAACCCG  GGGCGCCCCGGATCGGG  AACGTGC G   TGATGACGCACGTGCGCGCGA  AGACGT  GGGGACGCAGGCGGGTCGTAGA
                              (BRE-)                     (INR-)                        (DPE+)                       
    388 Mouse: Apg16l (77040, AK030983) 
Human: APG16L (55054, NM_017974) 
 
           |-50      |-40      |-30      |-20      |-10      |1        |11       |21       |31       |41       
Mouse     ATTTCCGGCAGAGACCGGAAAATCTTCCCGGATCGCCTCCGGGTGCCC  TG T GTGGG  AAGGTGAGGTTCCG  GCGGCCGGCTTCT  GTTCGCTGGCGGCTGGC
                                                            (INR-)                     (MTE-)                     
Human     TTTCCGGCATGAGCCGGAAGACCGTCCCGGATGGCCTCGGGGACTGCC  AG T GTGTG  GAGGTGAGCTCCG  GGATTGCCGGCAT  TCCCGCTTCTGCTGGTTG
                                                            (INR-)                    (MTE+)                      
    389 Mouse: 5930412E23Rik (77065, AK085362) 
Human: DKFZP434B168 (25896, NM_015434) 
 
           |-50      |-40      |-30      |-20      |-10      |1        |11       |21       |31       |41       
Mouse     CGCCTGCGCGGGGGGGCCCAGAGAGGAACTGGCGAACCGCGGGG  CGACCG A G  GCGCATGCGTACACTGCCTG  ACAGCGCGCGGAG  GGAAAAATCCGCGAG
                                                        (INR-)                           (MTE+)                   
Human     AGCGCCTGCGCAGGGGGTAGCAGAGGCGCGGACAAACCGCGGGGTGACCA G G  GCGCATGC  GTAACAGGGCTTG  ACAGCGCGCGGAA  GAAAAACCAGCAAG
                                                                (INR+)                    (MTE+)                  
    390 Mouse: Zfp142 (77264, AK020384) 
Human: ZNF142 (7701, BC033631) 
 
           |-50      |-40      |-30      |-20      |-10      |1        |11       |21       |31       |41       
Mouse     GGCCACATCAAAGGCCCCCGAGGAGCCACTCCAGTCCCAGTCGGA  CCATA G AG  ATGCAGCTCCCG  CCAGCCCACGGGT  AGGAGGTGAGAGAGCGGCTGTC
                                                         (INR+)                   (MTE+)                          
Human     CGGTCGCACAAAGCGGGCCCCGGGGGCCGTTCCAGCCGCGGCCGACCAT  A G AGATGC  GGCTCCCGCCGGCTCTG  GGTCTG  GAGGTGAGTGAGCGGCTGCG
                                                             (INR+)                    (DPE-)                     
    391 Mouse: 4930544G21Rik (77629, NM_172430) 
Human: SKIP (80309, AK127071) 
 
           |-50      |-40      |-30      |-20      |-10      |1        |11       |21       |31       |41       
Mouse     TTTTCAAGATCTGAGCAGACCACAGGAGGCGCCACCAGGACATCTCTGCA G   CGCTTGGT  GGCCAGGGA  GGAGCGAGCATCA  ATCTCCGCTGCGGGTCTCA
                                                               (INR-)                (MTE+)                       
Human     CAAGATCAGAGAGGACGGCGGGAGGGGACAGCGGGCGGTCGCAGCGC  ACA G AGCT  TGCTGGCCA  GGGAGGAGCTAGT  CTCCGTGGGCGCCGCCGCCGCCC
                                                           (INR+)                (MTE-)                           
    392 Mouse: Ralgps2 (78255, AK036803) 
Human: RALGPS2 (55103, NM_018037) 
 
           |-50      |-40      |-30      |-20      |-10      |1        |11       |21       |31       |41       
Mouse     CCCGCCCGGCGCGCGCCCCCTGCGCGGCCGGGGTGGATCCAGC  GCACTCC C   CTCCCCCGAGCGCCGGC  GGCAGGCGCTGAA  TGAGAGACGCCGACTGCTC
                                                       (INR+)                        (MTE-)                       
Human     CGCTCTCGTCCCCCACCCCTGGCGCGGACGGGGTGGTTCCAGC  TCACTCT C   CTCCCCCGAGCGGCAGCGGCGGCGGCGGCGGCGGCTGCTGCGGGCGCTG
                                                       (INR+)                                                   
    393 Mouse: Ankrd23 (78321, AF492400) 
Human: ANKRD23 (200539, AK092564) 
 
           |-50      |-40      |-30      |-20      |-10      |1        |11       |21       |31       |41       
Mouse     CCAAGAGAGTGCTCTCC  CATAAAA  GAGCTGAGCTCAATTCGCTAGGTGCA T GGAGAGCAAGGGGGG  ACCATGGACTTCA  TCAGCATTGAGCAGTTGGTAA
                            (TATA+)                                                (MTE-)                         
Human     CAGGCGCTCCTATAAAGGGCTGAGCAGTTCAGCTCTTCTCACTGGGG  GGT G TGAG  GAACAGGGGGG  ACCATGGACTTCA  TCAGCATTCAGCAGTTGGTAA
                                                           (INR-)                  (MTE-)                         
    394 Mouse: 4930438O05Rik (78795, AK030307) 
Human: FLJ12584 (80210, BX537956) 
 
           |-50      |-40      |-30      |-20      |-10      |1        |11       |21       |31       |41       
Mouse     GTCGCCTCACGTGACTTCGGCTAGCGGTAGCGGTTGCCCGGGTGACGGCC G GC  GAGCTGGC  GGTCTGGAGGAG  TGAGCGCAGAGCC  CTGCAGCAGGTAAG
                                                                 (INR-)                   (MTE+)                  
Human     CGCCCCCACG  TGACGCCG  GGGTGGCGGCGGCGGTTGCCCGGGTGACGGCT G CG  GAGGTGGC  GGCC  GGGCTGGGATAGC  GCGAGTGTCCGCGGCCGAGCAG
                      (BRE+)                                       (INR-)           (MTE-)                          
    395 Mouse: 1500015O10Rik (78896, BC002254) 
Human: ECRG4 (84417, BC021742) 
 
           |-50      |-40      |-30      |-20      |-10      |1        |11       |21       |31       |41       
Mouse     CAAGGATACTGGAGCTTTCGCCTGCCTCACTGAGCCTGGGTCTCCACT  CC A GTCAT  CCCTCCAGCTACT  TTGCAGCACTCTG  TCGCCATGAGCACCTCGT
                                                            (INR+)                    (MTE-)                      
Human     GGCCGCGCCTGCCCGCTCGCACCCCTCTCCCGCGCCCGGTTCTCCCTCGC A   GCACCTCG  AAGTGCGCCCCTCG  CCCTCCTGCTCGC  GCCCCGCCGCCATG
                                                               (INR+)                     (MTE-)                  
    396 Mouse: Slc19a3 (80721, NM_030556) 
Human: SLC19A3 (80704, NM_025243) 
 
           |-50      |-40      |-30      |-20      |-10      |1        |11       |21       |31       |41       
Mouse     AAAGTTGGGCGCGGCTTTGGTCCTGCGGGACTGACCCGGTGAC  CAAGTCC A   TCTTTACTTGAGATCGCCGAC  CCACATAGCTGAA  AGTGCTACATATCCG
                                                       (INR-)                            (MTE-)                   
Human     GCAAAGCGTGGG  GGCGTGGC  CCCGGGCTCCGGGCCAGGCAGGCTCCGC  CC C CTTCC  CACCAAGAGCGCCTGGAT  CCATCCGACAGCC  GCGGGTGCGTGGT
                        (BRE-)                                (INR+)                         (MTE+)                 
    397 Mouse: Uck2 (80914, NM_030724) 
Human: UCK2 (7371, CR608443) 
 
           |-50      |-40      |-30      |-20      |-10      |1        |11       |21       |31       |41       
Mouse     CATTAAGACGCTGACCTA  GCCGGTT  TCTTCACCCCTCCACCCCACCCCCC G CTCCGTCCCAGAAAGGGT  TAATCCGGCGGGT  GGGGAGGGGAGTGGCGCT
                             (TATA-)                                                  (MTE+)                      
Human     ACCAAATTAAAATACAGAACA  GCCTGTT  TCTTTCCCCCAGGGT  CCACTAG G   GCGCGTCGGAGAAAGGG  TTAACCGGGCGGG  TGGGAGGGAAGGTCGGGCT
                                (TATA-)                  (INR-)                        (MTE+)                       
    398 Mouse: Dusp12 (80915, AF268196) 
Human: DUSP12 (11266, BC006286) 
 
           |-50      |-40      |-30      |-20      |-10      |1        |11       |21       |31       |41       
Mouse     GCTATTCCGACACA  GGAGTCAG  TCACTCTAGCCGGTGACTTGGTGGCGGG C AG  CGGCTGCC  TGG  CTTCCTGGCTCTG  CCATGTTGGAAGCGCAGGGTTCT
                          (BRE-)                                   (INR-)          (MTE-)                           
Human     GCGAGTCCCAGGGGAA  GGATGTTC  TAGCCGGAGTCTACTCGATGGTAGG  G C AGGAAG  CCGCCTTGTCTCTGGGCGCGGCCATGTTGGAGGCTCCGGGCCC
                            (BRE+)                             (INR+)                                             
    399 Mouse: Sf3b1 (81898, AB037890) 
Human: SF3B1 (23451, NM_001005526) 
 
           |-50      |-40      |-30      |-20      |-10      |1        |11       |21       |31       |41       
Mouse     AGAGCGGCCTCTGTTTCTGGCGGCGGCGAGCGGAAGTCCTTGGGAGCG  CC A GTTCC  GTCTGTGTGTT  CGAGTGGACAAAA  TGGCGAAGATCGCCAAGACT
                                                            (INR+)                  (MTE+)                        
Human     GCTATTTTTCTCCGTGGCGGCGGCGACGAGCGGAAGTTCTTGGGAGCG  CC A GTTCC  GTCTGTGTGTT  CGAGTGGACAAAA  TGGCGAAGATCGCCAAGACT
                                                            (INR+)                  (MTE+)                        
    400 Mouse: Il24 (93672, NM_053095) 
Human: IL24 (11009, NM_006850) 
 
           |-50      |-40      |-30      |-20      |-10      |1        |11       |21       |31       |41       
Mouse     CTCCCTGCCAGACCCCT  TATATAC  AGTTCTCCCAGCCTTGCTTACCC  TCA G TCTT  TCACTTTTGAAATCAT  TTCCACAGCTGAG  AAGGAGCTTCCCACCC
                            (TATA+)                          (INR+)                       (MTE-)                    
Human     TCAGCCCCCATATAT  ATGCCCA  AATCTCCACAAAGCCTTGCTTGCCT  GCA A ACCT  TTACTTCTGAAATGAC  TTCCACGGCTGGG  ACGGGAACCTTCCACC
                          (TATA-)                            (INR+)                       (MTE-)                    
    401 Mouse: Lmod1 (93689, AK079173) 
Human: LMOD1 (25802, CR612066) 
 
           |-50      |-40      |-30      |-20      |-10      |1        |11       |21       |31       |41       
Mouse     GGCAGCTGTCAGGT  TTTATTG  GTTGCTGGTTAGACCAAGCAGGGT  CCACT A GC  AGGGAGGCTGCTGTGTCCCCC  GGAGCACCTGTAG  TACAGCAGGGCCC
                         (TATA+)                           (INR-)                            (MTE+)                 
Human     GGCAGTTGTCGGGTCTTATTGGC  CGCTGAT  TAGACCAGCCGGGGT  CCACT A GC  CCTGGGCTTCAGGGAGGCTGCTG  CGTCCA  GTGAACACTTCAGCACCT
                                  (TATA-)                  (INR-)                          (DPE-)                   
    402 Mouse: Klf7 (93691, AF338369) 
Human: KLF7 (8609, AB015132) 
 
           |-50      |-40      |-30      |-20      |-10      |1        |11       |21       |31       |41       
Mouse     AAAAAAAATGCAACCTCCCAA  AATAAAG  AGCAAAGATTGCATTAGGAGCG A ACAGCGCTGCAGATATA  GATGGCAGCTTCG  TGTCAGTGAGTTTGCGTTC
                                (TATA+)                                              (MTE-)                       
Human     GAAAAAAATGCAACCTCCCAA  AATAAAG  AGCAAAGATTGCATTAGGAGCG A ACAGCGCTGCAGAAATA  GATGGCAGCTTCG  TGTCAGTGAGTTTGCATCC
                                (TATA+)                                              (MTE-)                       
    403 Mouse: Ltap (93840, AK046036) 
Human: VANGL2 (57216, AB033041) 
 
           |-50      |-40      |-30      |-20      |-10      |1        |11       |21       |31       |41       
Mouse     CCTTCTGGTCCCCTCCCCCTCTGGTCCCCTCCCCCTCCGCCGCCGGCTCC A   GATCTGAT  TCCTG  ATCCCCGATTGCT  T  GGTTCT  GGGTCCCGCCATGGGA
                                                               (INR-)            (MTE-)      (DPE+)                 
Human     CACCCTCATCCCCTCCCCCTCCGCCCCCTCCCCCCTGCGCCGCCGGCTCC C   GATCTGAT  TCCTG  ATCCTTGATTCCT  T  GATCCT  TGGTCCCGCCATGGGA
                                                               (INR-)            (MTE-)      (DPE-)                 
    404 Mouse: Igsf9 (93842, AK053544) 
Human: IGSF9 (57549, AK056650) 
 
           |-50      |-40      |-30      |-20      |-10      |1        |11       |21       |31       |41       
Mouse     CGACGGGA  GGCGCTCG  GGGCA  TCTAAGG  CGGGGAGGCGGGTCCGCCCCCT A T  TGTGTGGC  TGCGA  GAGCAGAGCTGTG  CTGTGCAGAGCAGGTGAGGGTG
                    (BRE-)        (TATA+)                           (INR-)            (MTE-)                          
Human     CGACGGGA  GGCGCTCG  GGGCATCCGAGGCGGGGAGGCGGGTCCGCCCCCT A T  TGTGTAGC  GGCGAGAGTGGAGC  CGAGCGGTGCGGA  GCAGGTGAGGGAG
                    (BRE-)                                        (INR-)                     (MTE+)                 
    405 Mouse: Pi15 (94227, NM_053191) 
Human: PI15 (51050, CR749657) 
 
           |-50      |-40      |-30      |-20      |-10      |1        |11       |21       |31       |41       
Mouse     CTGGACTTTGCAAACTGACA  TATAAAA  GCTGTTAGCTGTTTCTGTAGCCA G CAGCATTCAAATCTTGCAGA  CTCTTGCACTCTG  AGAGCTTGTAATAAGA
                               (TATA+)                                                  (MTE-)                    
Human     GGACGTTGCAAACTGTGACA  TATAAAA  GCTGTTAGCTGCTCCTCTAGCCA G CAGCATTCAAACC  TTGCAGAGCTTTG  CTCTCAGAGAGTTTGTAAAAAGA
                               (TATA+)                                           (MTE-)                           
    406 Mouse: Igsf4b (94332, AK038917) 
Human: IGSF4B (57863, CN292398) 
 
           |-50      |-40      |-30      |-20      |-10      |1        |11       |21       |31       |41       
Mouse     GCTCCGAGCGCCCTA  TTTAATC  CCGGCGACTGCAGCAGCGCCGGCTCCCT C CCGGTACCCGCCTCG  GCCCCGGGCTTGG  AAGCCGCTCCGGGGTTGCCCC
                          (TATA+)                                                  (MTE-)                         
Human     GCTCCGAGCGCCCTA  TTTAATC  CCCGCGACTGCAGCAGCGCCGGCTCCCT C   CCGGTCCC  CACCTCG  GCCCCGGGCTCCG  AAGCGGCTCGGGGGCGCCCTT
                          (TATA+)                                (INR+)              (MTE-)                         
    407 Mouse: Prg4 (96875, NM_021400) 
Human: PRG4 (10216, NM_005807) 
 
           |-50      |-40      |-30      |-20      |-10      |1        |11       |21       |31       |41       
Mouse     TGAGTGGTGAGATTAAAGA  GCGGGTT  TCTGATGTTTTTATTTTATCTCAA G CAAGGGCGTTGCATCCG  AGAACCATGGGGT  GGAAAATACTTCCCGTCTG
                              (TATA-)                                                (MTE+)                       
Human     TTAGTGGTGAGATGAAAGA  GCTGTTT  TCTGATACTTTTATTTTATTTTCA G CAAGGGTACCTACGGTACCTGA  AAACAACGATGGC  ATGGAAAACACTTC
                              (TATA-)                                                     (MTE-)                  
    408 Mouse: Kmo (98256, NM_133809) 
Human: KMO (8564, NM_003679) 
 
           |-50      |-40      |-30      |-20      |-10      |1        |11       |21       |31       |41       
Mouse     CACAAAGGCTCT  GGACTTCT  GGC  AATAAAA  AGATCGCAAAACATCAG  CAT G TAGG  ACAGAGAGTTCCGACCT  GAACAGAGGTGTT  CTGGAAGCTATGGCA
                        (BRE+)      (TATA+)                    (INR-)                        (MTE-)                   
Human     CATGAATGCACTA  GGCTTGG  G  GCAGTA  TAAAAACTCAGAGAAATC  AGTGT G TA  GGAGACACAGAAAT  CAGTGTCACTCAG  TGACAGAAGCAACAATAATT
                         (BRE-)  (TATA-)                     (INR-)                     (MTE-)                        
    409 Mouse: Txndc9 (98258, AK006170) 
Human: TXNDC9 (10190, CR615367) 
 
           |-50      |-40      |-30      |-20      |-10      |1        |11       |21       |31       |41       
Mouse     TTCTCCGA  GGCTTTCG  TCACTCGCCACCGCCCTTTCCGCGAGTGTC  TCAC A TTG  TATCGCGAGAGGAC  TGTAGCCAATGAA  GTGGCGGAGTTAACAGCCC
                    (BRE-)                                  (INR+)                     (MTE-)                       
Human     CGCAGAACT  GTTCGCCA  CCCGACCGCCCGCCCCTTCCTCTGGCGTCCAAG G   TGATATCG  CGCGAGGTT  CGCAGCCAATAAG  GAGGCGGATGTGACGGCCC
                     (BRE+)                                      (INR+)                (MTE-)                       
    410 Mouse: Stk17b (98267, AK083332) 
Human: STK17B (9262, NM_004226) 
 
           |-50      |-40      |-30      |-20      |-10      |1        |11       |21       |31       |41       
Mouse     GTCTGTGTCGAAGTACAGTGTCCCAGACGCGGCTGCACTTTTCAAACCT  C A ACTGTA  AGAAGCGTCGGTCAG  CGTCTG  TGCGGTCGCCGCCGGGAGTCGC
                                                             (INR-)                  (DPE-)                       
Human     GGATCTGGCGAACGG  CGATGCCC  CAGACGCGGCTGCAGTTTTCAAACCG  C G ACTGCA  AGCTTCGGTAGT  CCTCTCCGCTGCT  GTCGCCAGGAGTCACTTC
                           (BRE+)                              (INR-)                   (MTE-)                      
    411 Mouse: Efhd1 (98363, AK045911) 
Human: EFHD1 (80303, BP212583) 
 
           |-50      |-40      |-30      |-20      |-10      |1        |11       |21       |31       |41       
Mouse     ACCCACGGTTCTTTTCAACCGCCGGGCCCCGAGCGATTCCACCGAGCG  TC A GAAAG  CCACGAGCCCGCAAAGAGCGCA  CAACTC  TCTGTCCCCGTACCGG
                                                            (INR+)                         (DPE-)                 
Human     GTCCTCAGACCCTCCCAACCGCCGGGTCCCCGCCGCCTCGGCGGAGTGTT   G TAGAGCC  TCGAGCCTGCGA  GGAGCGCGCCGCC  CGCCAGCTCCCTGCGTC
                                                              (INR+)                   (MTE+)                     
    412 Mouse: Lbr (98386, BC042522) 
Human: LBR (3930, NM_002296) 
 
           |-50      |-40      |-30      |-20      |-10      |1        |11       |21       |31       |41       
Mouse     CTAGGGGCGGGACCTCGGGGGCGCGAGAGCGCGGCCGGCTGTGTCGCGCG C   GGAGTCGC  CGCTGGCTGCTGG  TGACTG  TCCTGGAGTGGCTGGAGTTCCC
                                                               (INR-)                (DPE-)                       
Human     CGGCTTGCGGGACTGCGGGGCGCGAGAGCGCGGCCGGGTAGCGTCGCG  CG C GTGGA  TCTGCCGCCGGGTTGCTGTG  CGACTA  TTCTCCGGGAGCCGTCCG
                                                            (INR-)                       (DPE-)                   
    413 Mouse: Chst10 (98388, AK050676) 
Human: CHST10 (9486, AF033827) 
 
           |-50      |-40      |-30      |-20      |-10      |1        |11       |21       |31       |41       
Mouse     AGCCCGCCTCCGGGGCTCCGGGGACGCCCACAGGCCCCGCGAGGCCCGA  G C AGAGCG  CCAGCGGCTGGGA  GGAGCGCGAGCGC  CGATGTACGAGGGTGAG
                                                             (INR+)                    (MTE+)                     
Human     CCCAGGACCCC  GCGCGCTT  CGCCCACAGGCCCGGCGAAGCCCGACCCGCG C GGCGCCCCCAGGGCCAGGGGAGGAGCCTAA  GGACCC  GGACGAGCGCCGC
                       (BRE+)                                                                 (DPE-)              
    414 Mouse: Sh3bp4 (98402, NM_133816) 
Human: SH3BP4 (23677, BM147726) 
 
           |-50      |-40      |-30      |-20      |-10      |1        |11       |21       |31       |41       
Mouse     CCCGCCCGCGCTCGCGCCCCGCCCCGGCCCAGACCACCTCTGCGCCCGCT G GCGGGGGCCGAGC  GCTCTGGGCTGTC  GCGG  CGTCTG  GGCACCTCACCAC
                                                                               (MTE-)         (DPE-)              
Human     CCGCCCCCGCCCCCGCCCCCGCCCAGGCGCGGGACCACCCTCCGCCCGCC G AGGCGGGGGCCCAGCGC  GCCCGGCACTCTC  GGCGGTCCGGGCCCCTCGC
                                                                                   (MTE-)                       
    415 Mouse: AI597479 (98404, BC006931) 
Human: MGC5509 (79074, AK127661) 
 
           |-50      |-40      |-30      |-20      |-10      |1        |11       |21       |31       |41       
Mouse     CGCGCTGAGTGACACCAGAGTCCCGCCCCCTCACGTCACTTCCGCG  ACGC T TCC  GCCGCAGTGCCTTGTGGGAGGCCGGCCAGGGGGTCTCCTGGCGACA
                                                          (INR+)                                                
Human     GAGTGACGACACACGCGAGTCTCCGCCCGAGTACGTCACTTCCGCA  ACGC T TCC  TTCGCGGGGCTTTGTGGGTAGC  CGACTG  GGGTCTCCTGGCGACGAC
                                                          (INR+)                         (DPE-)                   
    416 Mouse: Atp1a2 (98660, D90049) 
Human: ATP1A2 (477, AA193018) 
 
           |-50      |-40      |-30      |-20      |-10      |1        |11       |21       |31       |41       
Mouse     GAGGGGGAGAGGGACCTA  TTTAAAG  CTACCCTGTTGCTCAGACTGTCTCT G TC  TGTCTGCC  AGG  GTCTCCAGCTGCC  CC  AGACAG  GCGGTGTGGTCTTGG
                             (TATA+)                               (INR-)          (MTE-)       (DPE+)                
Human     GAGGGGGAGAAGGACCTA  TTTAAAG  CTACCCTGTTGCTTTGGCTTTCTC  T G TCTGCC  AGGGTCTCCGACTGTCCC  AGACGG  GCTGGTGTGGGCTTGGGAT
                             (TATA+)                           (INR-)                     (DPE+)                    
    417 Mouse: 1190005F20Rik (98685, BC061491) 
Human: C1orf25 (81627, AK074993) 
 
           |-50      |-40      |-30      |-20      |-10      |1        |11       |21       |31       |41       
Mouse     TTTTCCTCATTGGACACTGTCCGCCACACGCTTCTGCGCAGTCTGTGGTG G CGTCGTAACGCCTCGGGGAAGGG  GAAGCCGGCGAAG  GGTCGAACCGCTG
                                                                                         (MTE+)                 
Human     CTTACCCAATT  GGACGCCG  TCCCCAGTACGCTTCTGCGCAGTTTGTGGTG G CGTCGTAACGCCTCGG  GGAAAGGGAAGC  G  GACGG  GCATCTGGAATCGCT
                       (BRE+)                                                       (MTE-)    (DPE+)                
    418 Mouse: 1190005F20Rik (98685, BC064024) 
Human: C1orf25 (81627, AK074993) 
 
           |-50      |-40      |-30      |-20      |-10      |1        |11       |21       |31       |41       
Mouse     CGGGGAAGGGGAAGCCGGCGAAGGGTCGAACCGCTGCCTCTGGCTT  TCAG T TTT  TCTGCTGACAGGGTTGGGT  CACTGGAGCTCCG  CCGTTTGTCTGTGG
                                                          (INR+)                          (MTE-)                  
Human     GGGGAAAGGGAAGCGGACGGGCATCTGGAATCGCTGCCTCTGGC  TTTCTG T T  TTCTACTAACAG  GATTTGGTCACT  G  GTTCT  TCATCTTTTGTCTGTTGC
                                                        (INR-)                   (MTE+)    (DPE+)                   
    419 Mouse: Rabif (98710, BC011166) 
Human: RABIF (5877, BC037392) 
 
           |-50      |-40      |-30      |-20      |-10      |1        |11       |21       |31       |41       
Mouse     CCCCAGGGACCTGCGCC  AGCGTAGT  TCCGGGTGAGCCAGCTCAGATCGCG C GTGCGCCGTTGTGGCTGATGGTGTGGCGGCACGGCGATGGAACCCTGCG
                             (BRE-)                                                                             
Human     CTCTGCCAAG  CCGCGCCA  GCGCAGTGAATAACGAGCCAGCAGAGACAG  CG T CTGCG  CAGCCGTGGCTGA  GGAGCCTGTGGCG  GCAGCGGCGATGGAACCA
                      (BRE+)                                  (INR-)                    (MTE+)                      
    420 Mouse: Rdh10 (98711, NM_133832) 
Human: RDH10 (157506, AF456765) 
 
           |-50      |-40      |-30      |-20      |-10      |1        |11       |21       |31       |41       
Mouse     TTCCCGGCGGTGGGT  TATATAG  CGCGGAGTGTGGAGCTACCTCAGAGC  CG G CTGGG  AACGCTCAGACGCTGCGGGGCCCCGTGCCCGAGTGACAGCGGCG
                          (TATA+)                             (INR-)                                              
Human     TCCCGGTCGCGGGGT  TATATAG  CGCGGAGCGTGGAGCCCGCTCAGAGCCG G CCCGGAGCGCTCTGACTTG  CAAGCGGGCTGCG  CTGCGGAGCCCAGTGCC
                          (TATA+)                                                      (MTE-)                     
    421 Mouse: AW822216 (98733, AK031074) 
Human: KIAA0657 (23363, BC007201) 
 
           |-50      |-40      |-30      |-20      |-10      |1        |11       |21       |31       |41       
Mouse     AGGGGGCTGGAAAGAGA  GACTCCCA  AGAGCAGGAAGCCCGGCCGCGG  GCA G TCTA  GGCTGTAGG  GCCCCTCGCTGAG  CAGGCAAACAGGAAGGACAGCCC
                             (BRE-)                          (INR+)                (MTE-)                           
Human     AGGGGGCTGGGAAGAGA  GGCTCCTA  AGAGCAGGAAGCCAGGCAGCGG  GCA G GGGA  GGCTGCGGG  GCCACTCGCTGGA  GAGGCAAACAGGAAGGACTGCCC
                             (BRE-)                          (INR+)                (MTE-)                           
    422 Mouse: BB219290 (98752, BC064708) 
Human: FREB (84824, AF531423) 
 
           |-50      |-40      |-30      |-20      |-10      |1        |11       |21       |31       |41       
Mouse     AGGTTTATTTCCTCTTTTCTAATTTGAGAGGTTTCATGTTGAAGAAAATC   A GTATGTT  GGGGTTG  CAGGAGGTATAAA  CCC  AGTCAC  CATGAAGCTGAGC
                                                              (INR-)              (MTE-)        (DPE+)              
Human     AAATTTACTTCCTCTTTTCTAATCTGAGAGGTTTCATGTTGAAGAAAA  TC A GTGTT  GGGGTTGCA  GGAGACCTAAACA  C  AGTCAC  CATGAAGCTGGGCTG
                                                            (INR+)                (MTE+)      (DPE+)                
    423 Mouse: Qscn6 (104009, BC076590) 
Human: QSCN6 (5768, CB137990) 
 
           |-50      |-40      |-30      |-20      |-10      |1        |11       |21       |31       |41       
Mouse     CCTCCTCCTCCGGGGCGGAGGCTGTTGGTGCGCGGCAGGCTCCGGATAC  T G ACTAGT  CACAAACT  TGAAGGAGGTGGA  CATTCAAGCCGCCTAGGATGAG
                                                             (INR-)               (MTE-)                          
Human     GTGGCTCCTCCTTGCGGGCGGAGGCAGGCGGTGCCGCGGCGCCGGGACC  C G ACTCAT  CCGGTGCTTGCGTGTGGTGGTGAGCGCAGCGCCGAGGATGAGG
                                                             (INR-)                                             
    424 Mouse: Uap1 (107652, AK047566) 
Human: UAP1 (6675, BC009377) 
 
           |-50      |-40      |-30      |-20      |-10      |1        |11       |21       |31       |41       
Mouse     CGCGCTCCCCTCGGCCGCTGCTCGTGGCCCGCCCCGCCGCCCCGGCCCC  T C GGATGA  CAGTATATATTCCAGGCGGGCGCG  GGACAC  GGGCGAGTAGCCG
                                                             (INR+)                           (DPE+)              
Human     GGCGCTCCACTTGGCCCCCGCTCCCGGCCCGCCCCGCCGCCGCGGCCCC  C C GGATGA  GGGTATATATTC  GGAGCGAGCGCG  G  GACGC  CGATGAGTGGCCG
                                                             (INR+)                   (MTE+)    (DPE+)              
    425 Mouse: Uap1 (107652, AK047566) 
Human: UAP1 (6675, BC009377) 
 
           |-50      |-40      |-30      |-20      |-10      |1        |11       |21       |31       |41       
Mouse     CTTCCTGTCGCCGGTGCCCGCCCCTTTGGCTGTTCTCGGTCGCT  GACGTG T C  GGGCCGGCAGGCTTCCGCTCCGCCCCTGGGCCGCAGCCACTCACCGCG
                                                        (INR-)                                                  
Human     GCGGCCGCCTCCGCGT  CCGCGTCG  TCGTCTGTGCTCCCGGCGCTGACG  TG T CTGGG  CGGTCGGCTTCCACTCCTTCAGGCGTCGGCAGCCACTAGTCGTG
                            (BRE+)                            (INR-)                                              
    426 Mouse: Mrpl30 (107734, AK009074) 
Human: MRPL30 (51263, NM_016503) 
 
           |-50      |-40      |-30      |-20      |-10      |1        |11       |21       |31       |41       
Mouse     CGGCGTGGTTTGAATGTTGCACTGGGTCCACCTGCCTCTGTTCCTCTCCT T CCGGCTGCGGGGACGTTC  GGAGTGGACACAG  CGGTCGCCGGTGAGTGCG
                                                                                    (MTE+)                      
Human     ACGGCAGGCTTTGAGTGTAGCACTTGGTAGTTCTTCCTCTGCTCTGCTTC C CTTCGGAGGAAAATTTCAG  GCTGAAGGTTTAG  CGGGTGCCGGTGAGTGG
                                                                                     (MTE-)                     
    427 Mouse: Mgat5 (107895, AK030402) 
Human: MGAT5 (4249, NM_002410) 
 
           |-50      |-40      |-30      |-20      |-10      |1        |11       |21       |31       |41       
Mouse     GGCCCCGACAGGGCTGGTAGTTTTCAGACCGTCCCTGCCGAGGCCCGCTC G CCCGGCAGCCCTGCGG  CTCCCGCGGTGGC  GGCGGCGGCCCCAGGCTCGA
                                                                                  (MTE-)                        
Human     GGCCCCGCCAGGGCTGGTAGTTTGCAGACCGTCCCTGCCGCGGCCCGCTC G GCGGCGGCCCTGC  GGCTCCCGCGGCG  GCGGCCCCGGGCGCGATCCAGCC
                                                                               (MTE+)                           
    428 Mouse: Atic (108147, BC039925) 
Human: ATIC (471, BG705531) 
 
           |-50      |-40      |-30      |-20      |-10      |1        |11       |21       |31       |41       
Mouse     CCTCGGGGTCACGTGGGGTCGACACGCCCTGCCCACTGCTTCCT  GAACCG C G  GGGTCCGGCCTGCCTGCCTACGCACGTGGCCTGTCCGTCCCTTGACTC
                                                        (INR-)                                                  
Human     GGCCACGTGATAAGCCCGGAAACAGCTCCGCCCCCTCGCTTCCTGAGCCG   C CACATCC  CGGCAGCCCTCCTACCTGCGCACGTGGTGCCGCCGCTGCTGC
                                                              (INR+)                                            
    429 Mouse: Rnpepl1 (108657, BC031789) 
Human: RNPEPL1 (57140, AF158748) 
 
           |-50      |-40      |-30      |-20      |-10      |1        |11       |21       |31       |41       
Mouse     CGCTGGCCTTCAGGCTGGACCCGTTCACCGACTATGGCTCCTCTCTGACC G   TCACACTG  CCTCCCGAGGTGCAGGCGCACCAGCCCTTCCAGGTCATCCT
                                                               (INR+)                                           
Human     CGCTGGCCTTCAGGGTGGACCCGTTCACCGACTACGGCTCCTCGCTCACC G   TCACGCTG  CCGCC  CGAGCTGCAGGCG  CACCAGCCCTTCCAGGTCATCCT
                                                               (INR+)            (MTE+)                           
    430 Mouse: Cops8 (108679, AF482000) 
Human: COPS8 (10920, BC036499) 
 
           |-50      |-40      |-30      |-20      |-10      |1        |11       |21       |31       |41       
Mouse     AAGTGACGACACGCTCGCCCATGCGTACTGAGAGGCAGCTCCTTTACCTC A TCGCGGGCGCGACGCCCCG  AGGGCCAGCTTGT  GCTTTGGTCTGGCAGAG
                                                                                     (MTE-)                     
Human     GACGGTCTGGTACGCAGGGGCGCTCGGCGGCAACGGCGGCTTTAAACGTC A TCGCGGGCGCGACGCCTGAGGG  ACAGTCTGGGGTT  TGGCTGTCCGGACG
                                                                                        (MTE+)                  
    431 Mouse: Ppp1r15b (108954, BC006897) 
Human: PPP1R15B (84919, NM_032833) 
 
           |-50      |-40      |-30      |-20      |-10      |1        |11       |21       |31       |41       
Mouse     GGTCCTCCGTCTCGCCCTGCAGCTTCCGGGTGTGCGGCTGCGG  CCATTTT G   AGCTTCGCTTCTTTGCGCCCTCGCCTGCCACCCAGCCACCCTTTCCGCC
                                                       (INR+)                                                   
Human     CCGGCGTCTAGGGGGGTGTCCTGCCGGCGCGCGGGCCCTGCGG  CCATTTT G   GGCTTCGCTTCCACCGCA  CCAGCCGGCCTAC  CCAGTCCTTCCGGTATCG
                                                       (INR+)                         (MTE+)                      
    432 Mouse: Tpr (108989, AK088078) 
Human: TPR (7175, BP314877) 
 
           |-50      |-40      |-30      |-20      |-10      |1        |11       |21       |31       |41       
Mouse     TAACGCGGGACTCTCTTTCCCAGGGTCCCGCCGCGGGAGTCTCAGGCGG  G C AGGCGC  GCGAGAGACCG  CGAGCGAGGCGGC    GGACGG  GGCGGCTCCGGCG
                                                             (INR+)                  (MTE+)     (DPE+)              
Human     TGAGGGTGGACTCGATTTCCCAGGGTCCCGCCGCGGGAGTCTCCGGCGGG C GGGCGCGCGCGAGCCAC  CGAGCGAGGTGAT  AGAGGCGGCGGCCCAGGCG
                                                                                   (MTE+)                       
    433 Mouse: Hdlbp (110611, AK048780) 
Human: HDLBP (3069, BP337189) 
 
           |-50      |-40      |-30      |-20      |-10      |1        |11       |21       |31       |41       
Mouse     ATTGGAGCCTCCGCCC  TATATAG  CAGCGGCGGCCCGGGCGCCGCGCCTCG G AGCGTCCCGGCTTCTCCCGC  GCAGCGGCAGCGA  CAGACGGACAGACTCT
                           (TATA+)                                                      (MTE+)                    
Human     GATTGGGCCGCCGCCC  TATATAG  CAGCCGGGGCCCGGGCGCCGCGCCTCG G AGCGTCCCGGCTTCTCCCGCGC  GGGGGGCGAGTAA  GCCAGCGGCAGGAC
                           (TATA+)                                                        (MTE-)                  
    434 Mouse: Hdlbp (110611, BC025648) 
Human: HDLBP (3069, BM465586) 
 
           |-50      |-40      |-30      |-20      |-10      |1        |11       |21       |31       |41       
Mouse     GCGGTTGGCCATATAGAGGCTGGGGGCGGGGGGAGGTCAAGCGTAGCCTC T TCTCCTTTACCAAGATGGCGTCTTGTTCCTGTTTCGCCACAGTTCCTAC
                                                                                                              
Human     AGTTGGCCATATAGAGGCTGGGGGTGGGGGGGGAGGTCAAGCGTAGCCTC T TCTCCTTTACCAA  GATGGCGGCTTGT  CCCTGTTTCGCCACAGTTCCTAC
                                                                               (MTE-)                           
    435 Mouse: Lmx1a (110648, AK044944) 
Human: LMX1A (4009, AK122800) 
 
           |-50      |-40      |-30      |-20      |-10      |1        |11       |21       |31       |41       
Mouse     GTTAGGGGGCGGAGGCCTTAGCA  CATAACG  CGTTGACTTCAAGTGAAA  TC A GAGCC  GTCCGAGCAGTTCACGGTGGCTCTTCTCTCTCCTCCCACCTTAC
                                  (TATA+)                     (INR+)                                              
Human     GTTGGGGGGCGGAGGCCTAAG  TACATAA  CGTGTTGACTTCAAGTGAAA  TC A GATCA  GCCAGAGC  AGTTCGCTGTGAC  T  GATCTC  TCCTCCCACCCTACAT
                                (TATA+)                       (INR+)               (MTE+)      (DPE-)                 
    436 Mouse: Rpl31 (114641, AK075964) 
Human: RPL31 (6160, CR600452) 
 
           |-50      |-40      |-30      |-20      |-10      |1        |11       |21       |31       |41       
Mouse     AGTTGTACGGCT  ACGCGACT  TTCCCTCCCACAAACCCTCGCGCCCTTCCT T TC  CTACTTGG  GCCCGGCAGAGT  GAGTATGGCTGCG  GGGTCGCGGCTCTG
                        (BRE+)                                     (INR+)                   (MTE-)                  
Human     GTACTTGCAACTGC  GGCTTTCC  TTCTCCCACAATCCTTCGCGCTCTTCCT T TC  CAACTTGG  ACGCTGC  AGAGTGAGTATGG  GTGGCGGAGTCTGGGCTCC
                          (BRE-)                                   (INR-)              (MTE+)                       
    437 Mouse: Prelp (116847, AK047480) 
Human: PRELP (5549, NM_002725) 
 
           |-50      |-40      |-30      |-20      |-10      |1        |11       |21       |31       |41       
Mouse     GATCTGCCCCTCCCTCCCCAAACTAACCTCCCCACCCCAATAG  GATCTGG T   AGAAGAAAAGTCAGGCAGACACACGC  AGACAC  GCACCAACTGGGAGACC
                                                       (INR-)                             (DPE+)                  
Human     TCTGCCCCTCCCTCCCCCCAAGCTAACCTCCCCACCCCAATAG  GATCTGG T   GGAAGAAAAGTCAGGCAAACA  CAAGCACGCACAC  ACCACTGGGAGATCA
                                                       (INR-)                            (MTE+)                   
    438 Mouse: Serpinb7 (116872, NM_027548) 
Human: SERPINB7 (8710, NM_003784) 
 
           |-50      |-40      |-30      |-20      |-10      |1        |11       |21       |31       |41       
Mouse     CTGGAGAATCTTCCCAGCATTTTGGCAGAAGTGTGCAGGGAGGAGCAG  AG A GTCTT  AGGAGGAAGGAGGCAGGAG  GAACTC  GAGCCCAGCAGCGAAGTCC
                                                            (INR+)                      (DPE-)                    
Human     CTTGGGAATTTTCCCTGAACCTTGGTATAGGTGTGCTGGGAGGAGTGA  GA A GTCTT  AGGAGGTAGGAGGCAGGAG  GAACTG  AAGCCCAGCTGTGAAGGCC
                                                            (INR-)                      (DPE-)                    
    439 Mouse: Slc19a2 (116914, AF224341) 
Human: SLC19A2 (10560, AF153330) 
 
           |-50      |-40      |-30      |-20      |-10      |1        |11       |21       |31       |41       
Mouse     GGGCGGGGCGTGGGGGCGGGGCCAGGCGGGGTGGCGGGCCAGACCCGGAG G C  GGACTTAG  GGAAAGG  GCGTTCGGCTAGC  GAGCTGTGGCCGCCGGCGGG
                                                                (INR-)              (MTE-)                        
Human     CCTTAGGACGGGTCTCC  CTTAAAC  TGGGCGATCAGGCAGCGACCCTAGAG G   CGTCTGTA  GGGTAAAGCTGGG  GGTTCT  GTAGCCGGAGGCGGCGGCGAGT
                            (TATA+)                              (INR-)                (DPE+)                       
    440 Mouse: Ivns1abp (117198, NM_028582) 
Human: IVNS1ABP (10625, AB020657) 
 
           |-50      |-40      |-30      |-20      |-10      |1        |11       |21       |31       |41       
Mouse     CGTGCGAGGGAGTGCGGCGCGAGCGCGCGCGGCGGCGACGGCGGTGGC  AG T GTGTT  CGGGACGCGCGTGAGGTCGGGNNNNNNNNNNNNNNNNNNNNNNN
                                                            (INR-)                                              
Human     GGTGACGTGCGAGGGGGTGCGGCGCGAGCGGTCGGCGGCGGCGGAG  GCAG T GTC  TCCCGGTCGCGCGTGGAGGTC  GGTCGC  TCAGAGCTGCTGGGCGCAG
                                                          (INR+)                        (DPE+)                    
    441 Mouse: Nphs2 (170484, AY050309) 
Human: NPHS2 (7827, AJ279246) 
 
           |-50      |-40      |-30      |-20      |-10      |1        |11       |21       |31       |41       
Mouse     TCCAGACAGCAAGGTCCTTAGGGCCTTGGGGTCGCTCCCCATCGGC  TCAG A AAG  CTGGGGCTGCGACTCTGCCA  GCAGCTGGCTCCG  GGGTTGCACCGCT
                                                          (INR+)                           (MTE+)                 
Human     CCCCCACCCGACGGTCTTTAGGGTCCCCCGGGCACGCCACGCGGACCCGC A G  CGACTCCA  CAGGG  ACTGCGCTCCCGT  GCCCCTAGCGCTCCCGCGCTGC
                                                                (INR-)            (MTE+)                          
    442 Mouse: Sgk3 (170755, AK031133) 
Human: SGKL (23678, BI598588) 
 
           |-50      |-40      |-30      |-20      |-10      |1        |11       |21       |31       |41       
Mouse     CCGCCCGCCGCC  CGGCGCTT  CGCGGGAGGAGCCGCCGTGACGCGTCAGGG A GGAAACGCCGGCGCCCGGCCGCG  CCGCCGGGAAGAA  GAAACTGCCAGCG
                        (BRE+)                                                             (MTE-)                 
Human     CTCCCGCGCGCTCCGCGGGAGGAGCCGCCAGCGGCCGTGACGCGTCAAGG A GGAAACGCCGGCGCCCGGCGGCC  CTGCCGGGAAGGA  GGAAGCGCAGTGC
                                                                                         (MTE-)                 
    443 Mouse: Acbd3 (170760, AF501319) 
Human: ACBD3 (64746, AB043587) 
 
           |-50      |-40      |-30      |-20      |-10      |1        |11       |21       |31       |41       
Mouse     CCGCCTTGGCGGCGGAAGTTCCGGTGTCGGCGAGCGGAGGTGAGAGGTCA G CAGGAAGTCGATACGTGGCCGCCGCCTGTCCCCGCCGAGGAGGCGCTGC
                                                                                                              
Human     CCTCCTCAGCAGCGGAAGTTCCGGTGTCGGTAAGCGGAGGTCAGAGGTCA G CAGGAAGTCGATACGTGGCTGC  CGTCTG  TCCCCGCTGAGGAGGTGCAGC
                                                                                    (DPE-)                      
    444 Mouse: Khdrbs2 (170771, AK078160) 
Human: KHDRBS2 (202559, BC034043) 
 
           |-50      |-40      |-30      |-20      |-10      |1        |11       |21       |31       |41       
Mouse     GGGTTGGGCCGCACG  GGCATCCT  CTCCAGCTGAGGCCACGGCCGG  AGCCT G GC  TGGGGCGCGGGAGAGGCAGGTACCAGCGC  GGTCTG  CGCAGCCCCAGG
                           (BRE-)                          (INR-)                                (DPE-)             
Human     CGTCCTGTTCCCG  CCTCTCCA    GTTAAGG  CCGCTGGTGTGAGCCGGG  GCTC T GCG  CGAGCGAGGGACGACGGAAG  GGACGG  GCAGGTGTGGGCGCGGGGCC
                         (BRE+)   (TATA+)                     (INR-)                       (DPE+)                     
    445 Mouse: Nme7 (171567, AK043515) 
Human: NME7 (29922, AF241238) 
 
           |-50      |-40      |-30      |-20      |-10      |1        |11       |21       |31       |41       
Mouse     GGCGGCGTCTTCGAAGCAGTGGCTGGAGGAAGGCGGGGCTTGCGTTGCC  A G AGTAAC  CGGATCATTGGTGGTGGTGTAGTCTTCCTGTGTACAGGAATCT
                                                             (INR-)                                             
Human     GGCGGTGGCGTC  CCACGCCT  CGTGCGACAGTGGGCGGGGCTTTGTTGCC  T G AGTAAC  CGTATGATGGTGGTGGTGGTGG  TGTCTT  CCTGTCTCAACGATA
                        (BRE+)                                 (INR-)                         (DPE-)                
    446 Mouse: BC031781 (208768, AK078485) 
Human: FLJ35382 (163859, NM_152608) 
 
           |-50      |-40      |-30      |-20      |-10      |1        |11       |21       |31       |41       
Mouse     TCATCGAGATGCGACCGGATGGCTGTGTCGCCTAGTGCCTGTC  GCAGTCC A   GAGGTTCCGTCAC  CAGACGAAATGGC  GGAGGCTGCGGTGGTGGCGTGGG
                                                       (INR+)                    (MTE-)                           
Human     TCATCAAGA  GGCGCCGG  GCGAGTTGCGGCGCCTGGTGTCTCAGAGA  GGCT T GAG  GTTCGGGTAGTCGGTGACATGGCGGAGGCCGCGGCGCTGGTGTGGA
                     (BRE-)                                 (INR-)                                                
    447 Mouse: BC014795 (208795, NM_144794) 
Human: KIAA0792 (9725, AB007958) 
 
           |-50      |-40      |-30      |-20      |-10      |1        |11       |21       |31       |41       
Mouse     AAATTAGGTAA  CGCGTGGA  CACAGTTCTGCGCATGTGCAAGAGGAGCC  GG A GTTTC  ACTTTGTAACTTTTAAGTGGTGC  GGACGC  CACCCCGCCTCGCCA
                       (BRE-)                                 (INR+)                          (DPE+)                
Human     AAATTGGGAAA  GGGGTGGC  CGCAGCTCTGCGCATGTGCAGGAGGCGCC  GG A GTTTC  ACTTTGTAACTTTTAAGT  GGTCGG  AACACGCCCCGCGCTGCTGG
                       (BRE-)                                 (INR+)                     (DPE+)                     
    448 Mouse: Trp53bp2 (209456, NM_173378) 
Human: TP53BP2 (7159, NM_005426) 
 
           |-50      |-40      |-30      |-20      |-10      |1        |11       |21       |31       |41       
Mouse     GCGCGCCTCGGTGGGGGCCCGGCCGGGATTAGTTGGTTTCGGCGAGAAGG A GGAGGAGGAGGTGGGAGTCG  CGAGCGCCGAGAC  AAAGCCGCGTCCCGGA
                                                                                      (MTE+)                    
Human     GGCACGGGCTCGGCTGGGGCCCGACCCGGGATTAGTTGGTTTCGGAGCGG A GGAGGGAGCCCCGACCGTCA  CGAGCGTCGAAGA  GACAAAGCCGCGTCAG
                                                                                      (MTE+)                    
    449 Mouse: 9430069J07Rik (211383, BC070435) 
Human: FLJ38377 (205147, AK095696) 
 
           |-50      |-40      |-30      |-20      |-10      |1        |11       |21       |31       |41       
Mouse     GCTGGCCGTGGTGCTGACGCGAGGCTCGGGCGCGCACTCGTTCCTGGCTC G GGCACGGCCGCGCC  CTGCGACACTCCC  CAGCCCCGGCGGTAAGTGGCTG
                                                                                (MTE-)                          
Human     GCTGACCGTGGTGCTGAGCGCGGCTCGCGCTCCGACGCGGTGCCCG  AGCC T GTC  GCGGCCGCGCC  CTGCTGCACTGCG  GGCCCCCAGCGGTAAGTCGCCA
                                                          (INR-)                  (MTE-)                          
    450 Mouse: Tsga10 (211484, BC066782) 
Human: TSGA10 (80705, AK094208) 
 
           |-50      |-40      |-30      |-20      |-10      |1        |11       |21       |31       |41       
Mouse     CGCGAGAG  GGCGTGTC  GAGACAGGTACGCGTGCGCACGGGTGCTAGGGCG C G  CCGGACGG  AAGT  GGAGCGTAGGCGC  CGGGGAGGTGGAAGAAGAGCCCC
                    (BRE-)                                        (INR+)           (MTE+)                           
Human     GCGAGAAGGAAGG  TCGAGCCA  GGTACGCGTGCGCACCGGGCCTGGGGG  CG A GCGGG  ACGGAAGC  AAAGCTGGGGCGC  CGGGGAGGTGGAAGACGAGCCCC
                         (BRE+)                               (INR-)               (MTE+)                           
    451 Mouse: Tsga10 (211484, AK030254) 
Human: TSGA10 (80705, AK094208) 
 
           |-50      |-40      |-30      |-20      |-10      |1        |11       |21       |31       |41       
Mouse     GCTCAGCCCTGGGCAATAGCCTTCGCGCCTGCGCGGGCTAGAGGATGG  TT A CTAGG  AGACCGGACGCAGGGGTCGGC  CATCTC  GGCGCTGCGTGGCCGTG
                                                            (INR-)                        (DPE-)                  
Human     CAGGCAGAGATTAAGAAA  TGAGTCGT  GGAAAAGGGGGCTAGCGGCTGG  TT A CTAGG  AGACGGGACGCTCGGCTCGGG  CGTCTA  GGCGCTGCGTGGGCGTG
                              (BRE-)                          (INR-)                        (DPE-)                  
    452 Mouse: Satb2 (212712, NM_139146) 
Human: SATB2 (23314, AJ438951) 
 
           |-50      |-40      |-30      |-20      |-10      |1        |11       |21       |31       |41       
Mouse     GAGAGAGAGAGAGAGAGAGAGAGAGAGAGAGAGAGAGAGAGAAGCATAGG   T AACCAAC  AAACCCAA  TTACTAAGCTTTA  TTGATACTAAAAGGAAAAGTT
                                                              (INR-)               (MTE-)                         
Human     GAGAAAGAGAGAGAGAGAGAGAGACTGAGAAAGTCGAACTCGAGC  TTAGG T AA  CCAACAAACCCAA  TTACTAGGCTTTA  TTGATACTAAAAGGAAAAGTT
                                                         (INR+)                    (MTE-)                         
    453 Mouse: Pcanap6 (212980, AK035428) 
Human: PCANAP6 (85414, AW939762) 
 
           |-50      |-40      |-30      |-20      |-10      |1        |11       |21       |31       |41       
Mouse     CCCCACTGAGTAACCTGGAGA  TTTAAAA  GGCGCCCGCTGGCGCGCGTTGG T GAAGCAGGGGTCCGAGCTCGCACGCGCCAGCCCCAGGTGACAGCCGCAC
                                (TATA+)                                                                         
Human     CCCCGCGGAGTAACCTGGAGA  TTTAAAA  GCCGCCGGCTGGCGCGCGTGGG G GGCAAGGAAGGGGGGGCGGAA  CCAGCCTGCACGC  GCTGGCTCCGGGTGA
                                (TATA+)                                                  (MTE+)                   
    454 Mouse: A930031D07Rik (213006, AK048692) 
Human: DKFZp761N1114 (148808, AK090831) 
 
           |-50      |-40      |-30      |-20      |-10      |1        |11       |21       |31       |41       
Mouse     GGGCGGGGCAGCCGCGCGGCGCTCCCCTCCTCTCCTCTCCTCCCCGCCCG G CCTCCGGTTGGCCGCGGCACTCG  GCCCTGGGCTCGC  TGCGAGCTGCCGG
                                                                                         (MTE-)                 
Human     GGGGCCCCTCGCTGGGGCGGCCCCTCCCCGCCCCTCCCTCCCCGCCGCGG G CCTCCCCAGCCCGGCCGG  CCCTCCCGCGGGC  GGCACTCGGGCACCGGGC
                                                                                    (MTE+)                      
    455 Mouse: BC049806 (213056, AK052638) 
Human: MGC39518 (285172, AK124184) 
 
           |-50      |-40      |-30      |-20      |-10      |1        |11       |21       |31       |41       
Mouse     TGCTGTCTTGGGGGGGTTGCGGGGGGGGGGGCGCACGGTGCCGGGTGAAG A   GGAATTTC  TCCTCCG  GCTTCGGACGCCG  T  AGTCGG  GAGCTGACGAAGGT
                                                               (INR+)              (MTE+)      (DPE+)               
Human     ACAGGTAGGCGGCGGTGCTGTCTTTGGGGGAGGCAAAGTGCTAGATGAAG A GG  GGTTTTCT  TCTCCTTCGGC  CGCGGAAACTACA  GACGGGAGCTGGCGA
                                                                 (INR-)                  (MTE-)                   
    456 Mouse: BC049806 (213056, BC049806) 
Human: MGC39518 (285172, NM_173822) 
 
           |-50      |-40      |-30      |-20      |-10      |1        |11       |21       |31       |41       
Mouse     TGTTATTAAGCGCGTGGGCCCACTCAATGAGAAGGCTGGTTGTTATGCT  G C AGTTGG  CAGGCTGCTGC  GGGAGGCGGTGGC  GGTGGCGGTGGCGGTAGGA
                                                             (INR+)                  (MTE-)                       
Human     TGTTGTTGAGAGCGTGTTCTCACCCAATGAGAGGGTTGGTTGTTGCGCT  G C AGTTGG  CAGGCTGCTGCGGGAGG  CGGCGGCGGTAGG  AAGCCGGAGACAG
                                                             (INR+)                        (MTE-)                 
    457 Mouse: Rbbp5 (213464, AK049247) 
Human: RBBP5 (5929, NM_005057) 
 
           |-50      |-40      |-30      |-20      |-10      |1        |11       |21       |31       |41       
Mouse     TAAAACGAACAAGGAAGGCGGGGGAGGAAAGCGGAAGCCGCGGGGCCTTC   T AAGTCCG  AAAGTCTCC  GGAGCTTGCGCCA  GGCTCTTCGCGGCGCCCCCC
                                                              (INR+)                (MTE+)                        
Human     TGAAACGAACAAGGAAGGCGGGGG  AAGAAAG  CGGAAGCCGCGGGGC  CTTC T AAG  GCCGAAAGTCTTC  GGAGCTTGCGCCA  GTCTCTTCGCGGCGTCCACC
                                   (TATA+)                  (INR-)                    (MTE+)                        
    458 Mouse: Bag2 (213539, BC016230) 
Human: BAG2 (9532, NM_004282) 
 
           |-50      |-40      |-30      |-20      |-10      |1        |11       |21       |31       |41       
Mouse     GTCCGCGTCAG  CGGCGCCC  GTGGTGACGGCGACGTGCGGCTCGCG  CCACT G CT  GCCCGCGGACGCCGAGGAC  CCCCGGAGCTCAT  GGCCCAGGCGAAGAT
                       (BRE+)                              (INR-)                          (MTE-)                   
Human     GGGCGCCCGCGTGGTGAC  GGCGACGC  CTGCAGCCCAAGGAGCGCT  CCACT C GC  TGCCGCCGGAGGGGCCGGTGA  CCTCTTGGCTACC  CCGCGTCGGAGGC
                              (BRE-)                       (INR+)                            (MTE-)                 
    459 Mouse: Rcor3 (214742, AK050287) 
Human: RCOR3 (55758, AK057163) 
 
           |-50      |-40      |-30      |-20      |-10      |1        |11       |21       |31       |41       
Mouse     GAGGCGGGGCCGGGGCGGGCCGGTGTGGGGCGACTGCGCTACGGCT  CGAC C GGG  GCGGTGATGGC  GGCTCCATATTAA  CACCTCCTCCTCCTCCTCCGCG
                                                          (INR-)                  (MTE-)                          
Human     GAGGCGGGGCCGGGGCGGGTTGTTGTGAGGCGACTGCGCTACTGCC  GGAG C GGG  GCGGTTATGGC  GGCTCCATATTAA  CAGCCTCCTCCTCCTCCGCCGC
                                                          (INR-)                  (MTE-)                          
    460 Mouse: Arid5a (214855, AK085015) 
Human: ARID5A (10865, BC047390) 
 
           |-50      |-40      |-30      |-20      |-10      |1        |11       |21       |31       |41       
Mouse     TCCATGGGCGGGTCCGCGGGGGAGGAGGCAGTCGAGCGGCGCCGGTAG  TC A GTCGC  GCCGAGAGCGCTGGGACA  GGACGG  CTGTGCGTGTAGAGGGTCTC
                                                            (INR+)                     (DPE+)                     
Human     GGGACGGGGCGGGACTGCGGGGAGGTGGCCGGCGGGCCGCGCCGCGAGCC A G  TATCTCAG  AGAGCGCGGGGTCC  GGACAG  CCGCGCGCTGAGGGTCTCGG
                                                                (INR-)                 (DPE+)                     
    461 Mouse: C530043G21Rik (215015, BC023737) 
Human: FAM20B (9917, AL701403) 
 
           |-50      |-40      |-30      |-20      |-10      |1        |11       |21       |31       |41       
Mouse     GGCGTGGGGCTGCCCCTCCCCGGAGGCGGCGGGGGCGGCCGGGGCCGCGC C TCACCGCACCGCGC  GGGCGGCTATGGA  GCGAGCCTGAGGCCCGCCAGGT
                                                                                (MTE-)                          
Human     GGCGTGGGGCTGCCCCTCCCCGGAGGCGGCGGGGGCGGCCGGGGCCGCGC C GCACCGCACCGCGCGGGCGGCC  ATGGAGCGAGCCT  AGGGCCCGACAGGT
                                                                                        (MTE-)                  
    462 Mouse: AA408296 (215193, AK052355) 
Human: MGC29875 (27042, BC022964) 
 
           |-50      |-40      |-30      |-20      |-10      |1        |11       |21       |31       |41       
Mouse     CTGGGCGTAAAGCAGC  TTTGCTT  TACGGCTCCCGCCTCCGCCGTAAAGCC   A GCGTGCC  CACGTGCGACAGCGA  CAACTT  GCCACTGGGAACCTTGTGTTT
                           (TATA-)                              (INR-)                  (DPE-)                      
Human     GTAAAGCGGATCTGC  TTTATGG  CACCTTGCTTTCGCCGTAAAGC  GCAGTC A G  CGAGCCCACGTGCT  TGTGTTGACTGGA    CAACTT  CCTGGTGGAAAACCG
                          (TATA+)                         (INR+)                     (MTE-)     (DPE-)                
    463 Mouse: 6030423D04Rik (215243, AK041994) 
Human: T3JAM (80342, AK125601) 
 
           |-50      |-40      |-30      |-20      |-10      |1        |11       |21       |31       |41       
Mouse     TGGGTGCCTGGGTGGACTTCCTCACCAGCCCTGGAGGCCCCAAGAGTGCA G GGGCCCTCTCAAATTGCTAAGGGCAAGGAGAGGAACTGAGGTTTGTGAA
                                                                                                              
Human     CTACTGATGGGAGGAGCTCCTGAGCCCAGCCCTGGAGGCCTCAGAGTTCA G GGGCCCACTTAGA  TGATACCAGAGAG  CAAGAGAGAGGAACTGGGGTTTG
                                                                               (MTE+)                           
    464 Mouse: Camk1g (215303, NM_144817) 
Human: CAMK1G (57172, BQ086330) 
 
           |-50      |-40      |-30      |-20      |-10      |1        |11       |21       |31       |41       
Mouse     TACAGTCCTGGGAAAGAAA  ACCCCTG  TATTCCTCCCCTTTAATTG  GGTTT A TT  GTTAAAGCACTGGG  GCAGCTAATCCTC  AC  AGACCT  GTAGGAGCAGGC
                              (TATA-)                      (INR-)                     (MTE+)       (DPE+)             
Human     TACGGTCCTGGGAAAGA  AAACTCCT  GCATTCCTCCCCTTTAATTG  GGTTT A TT  GTTAAAGCACTGGA  GCAGCTAATCCTC  AC  AGACCT  GTAGGAGCTGGA
                             (BRE+)                        (INR-)                     (MTE+)       (DPE+)             
    465 Mouse: B830045N13Rik (215378, AK046878) 
Human: FAM5C (339479, NM_199051) 
 
           |-50      |-40      |-30      |-20      |-10      |1        |11       |21       |31       |41       
Mouse     GCCTAAATCAGTGCTGCTGCTCTCTGAAGATGACTACAGCCTAGCTCA  CT A GTTGT  CTACTCCTGTTTACAACA  CATCCT  TTCACTTCTCTTTGGAGATC
                                                            (INR+)                     (DPE-)                     
Human     AAATCATTCTTGCTGC  TATATCT  ATCAGGATGACTACAGCCTTGCTCA  CT A GTTCT  CTCTTTATCTCGTTGTCTTCAACA  CATCCT  TTTACTTCTCTTTG
                           (TATA+)                            (INR+)                           (DPE-)               
    466 Mouse: Rnpep (215615, BC010520) 
Human: RNPEP (6051, BQ948696) 
 
           |-50      |-40      |-30      |-20      |-10      |1        |11       |21       |31       |41       
Mouse     GGCGGAGCCTGCCAAGACCCGAGGAGCGACCGACCTTCCTGGCGGGTGAG C AACCGCTCAGGAGCCATGGAGAGCGGC  GGACCA  GGCAACTACAGCGCGG
                                                                                         (DPE-)                 
Human     CGCGGCGCTCGGGGCGGGGCTCCCCTCGGGTTCGCGGCCCGGCCGGTGAG C AACGGCTCTGCGGCCATGG  CGAGCGGCGAGCA  TTCCCCCGGCAGCGGCG
                                                                                     (MTE+)                     
    467 Mouse: Epb4.1l5 (226352, BC003937) 
Human: EPB41L5 (57669, AK023019) 
 
           |-50      |-40      |-30      |-20      |-10      |1        |11       |21       |31       |41       
Mouse     CGACGGCCAATGGGGCTG  GGCGCTGA  GTTTCGGGCGATACCGCGCGGTGG C CGCTGAGGGCCGGGCGCGTCGCCGG  GGTCTC  GCGCGTTGTTGGGGGGAA
                              (BRE-)                                                     (DPE-)                   
Human     GGCGGCCAATGAGGCAGGGCCGCCGAGTTTCGGTCGATACCGCGCGACGG G CCGGGGCGGAGGGCCCGGGGCAGCCG  GGTTAA  TGTTTGCCGAGCGGACG
                                                                                        (DPE+)                  
    468 Mouse: Epb4.1l5 (226352, AK040354) 
Human: EPB41L5 (57669, AK023922) 
 
           |-50      |-40      |-30      |-20      |-10      |1        |11       |21       |31       |41       
Mouse     GGGGCGGACCTTGGATGGGGC  TTTAAGG  GGCGTGCCCTCGGGCGGCCGGG G TCCTCCGGGGATTAGCGC  AGGGTGGGCTCGC  GCCTTGCGCCGCCATTTC
                                (TATA+)                                               (MTE-)                      
Human     GGGGCGGAGCTGGGAGGGAGC  TTTAAGG  GGTGGACGGGCGGGAGGTCGGG G TCCTCCGGGGATTAGAG  CCGGTGGGCTCGT  TGTGGGCGCCATTTCTCGG
                                (TATA+)                                              (MTE-)                       
    469 Mouse: R3hdm (226412, NM_181750) 
Human: R3HDM (23518, NM_015361) 
 
           |-50      |-40      |-30      |-20      |-10      |1        |11       |21       |31       |41       
Mouse     AGCGTATGAGGTAGGCTGCTTTCCGTCAACGCGACGGAGAGGCGGGG  CCA G TGCG  TGGTGGGAA  GGGGCGGGATTCT  GCCGCCGCGGCTGCCGCTGGAGC
                                                           (INR+)                (MTE-)                           
Human     AACCTATGAATAGTCGGCTGCCGCTCAAAGAGAACGAAGAGGCGGGG  CTA G TGCG  TGGTGGGAA  GGGGCGGGATTCT  GCCAGCCGCGGCTGCCGCTGGAG
                                                           (INR+)                (MTE-)                           
    470 Mouse: Lct (226413, L04635) 
Human: LCT (3938, AJ001492) 
 
           |-50      |-40      |-30      |-20      |-10      |1        |11       |21       |31       |41       
Mouse     AGCCTTGGCTGTGCT  CATAAAG  TCAGGATTCCTTCCACACCGTTCTA  GCA G TTCC  TTGACCATGGAGCTGCCTT  GGACAGCACTCTT  CCTCAGTACCTTC
                          (TATA+)                            (INR+)                          (MTE-)                 
Human     TACAACCTCAGTTGCAGT  TATAAAG  TAAGGGTTCCACATACCTCCTA  ACA G TTCC  TAGAAAATGGAGC  TGTCTTGGCATGT  AGTCTTTATTGCCCTGCTA
                             (TATA+)                         (INR+)                    (MTE-)                       
    471 Mouse: Dars (226414, AK077820) 
Human: DARS (1615, AA355758) 
 
           |-50      |-40      |-30      |-20      |-10      |1        |11       |21       |31       |41       
Mouse     GGAGCTGGCGGCGGCGCGCGAGAGCTCGCTGTGGCTCTCGCGATATTTCC G AAGCCAAGGTTCCACGCGGAG  ACTGGGGGCTTAG  AACCCGGGCTGGGAG
                                                                                       (MTE-)                   
Human     CCGCCAGCGCTCCGGGATCTCGAGATAGCCGCAGCTCTCGCGATC  TTTCT G GA  GCCGCACCTCCACGCGGAGTC  CGAGCGCGTGTGC  TGAGACCCCAGGG
                                                         (INR-)                            (MTE+)                 
    472 Mouse: 9830132G07Rik (226470, NM_172643) 
Human: FRBZ1 (360023, CD699546) 
 
           |-50      |-40      |-30      |-20      |-10      |1        |11       |21       |31       |41       
Mouse     CGGACCCGGAAGTGGGTGGGCGCGGGCGCGAGCGCGCGGGGATCG  GAGCT G GG  CGGCCCCGAGCCGGCGCG  GGAGCCCGGGCTT  CCCGGTAATGGGCCTG
                                                         (INR-)                         (MTE+)                    
Human     TGAACCCGGAAGTGGGTA  GGCGCAGG  AGCGAGCGCGCCCAGAGTGGAGC  T C AGCTGC  TGGGAGCCAGCGGGGGAGCCTTG  GGTTCC  CGGTAATGGATCTG
                              (BRE-)                           (INR+)                          (DPE+)               
    473 Mouse: 9430023P16Rik (226517, BC082789) 
Human: C1orf16 (9887, NM_014837) 
 
           |-50      |-40      |-30      |-20      |-10      |1        |11       |21       |31       |41       
Mouse     CACCCCGCGCCA  AGCGAGGA  GGAGTCGGAGGAGAGGAAGATGGCGG  CGAC C GCC  AGCACCCGCGGGGCCG  CGGGGCCGCTCCG  AGGAGCCTGAGAGACCC
                        (BRE-)                              (INR-)                       (MTE-)                     
Human     ACCCCCCTGCCG  AGCGAGGA  GGAGCCGGAGGAGAGGAAGATGGCGGCGGC C GCCAGCACCCGCGGTGCCG  CGGGGCCGCTCCG  AGGAGCCTGAGAGACCC
                        (BRE-)                                                         (MTE-)                     
    474 Mouse: Lamc1 (226519, J03749) 
Human: LAMC1 (3915, M55217) 
 
           |-50      |-40      |-30      |-20      |-10      |1        |11       |21       |31       |41       
Mouse     GGCAGACCCCACCCCCTTCTCCTTCCTCCTGGGGCGCGCTCTCGAGTGCG C GCTCGGAAGTCGGGGGTCGGCGC  ACAGTGCAGGCTG  CGCACCGGGAGGT
                                                                                         (MTE+)                 
Human     CCGACCCCACCCCCTGCTCCTTCCTCCCCGGGGGCGCGCACTCGG  GCACG C GC  TCGGAAGTCGGGGGTCGGCGC  GGAGTGCAGGCTG  CTCCCGGGGTAGG
                                                         (INR+)                            (MTE+)                 
    475 Mouse: 5830468K18Rik (226539, BC057943) 
Human: FLJ10514 (55157, BC045173) 
 
           |-50      |-40      |-30      |-20      |-10      |1        |11       |21       |31       |41       
Mouse     GGAGGCGCCCTCGTCCTGATTGGCCGGAATCCTTGCGTAACTTTGGCGCT C GCCGGGCGTCAGAGGGACTG  GCTTGTGGATGTG  GTTTGTGTCTGGGTTT
                                                                                      (MTE-)                    
Human     GGCACCTCACCACTGA  TTCGTTGA  ATTCCTTCCCGGTAATCTTGG  GCACT A GC  GGGCGGAGTTGAAGGGCGCTT  GGACCC  CAGCGGCGATCTGTGTTTGG
                            (BRE-)                         (INR-)                        (DPE+)                     
    476 Mouse: Klhl20 (226541, BC019571) 
Human: KLHL20 (27252, NM_014458) 
 
           |-50      |-40      |-30      |-20      |-10      |1        |11       |21       |31       |41       
Mouse     TCTGGGCGGGTGCAGGGAAAGGAAATCGCTGCAGGAGCCGCATCCTGCCT G CGCTAGCGGCTCCAGAGGTGGAGGCAGAGAGGGCGGAGAAGGCAGAGAG
                                                                                                              
Human     TATGGGCCGGTGAAGGGAAAGGAAATAGCTCTACGAGCGGCATCCTGCCT G CGTTAGCGGCGGTGGAGGAGGAGGCAGAGAGGAGTGGAGGGCGGAGTAG
                                                                                                              
    477 Mouse: Fmo4 (226564, NM_144878) 
Human: FMO4 (2329, AL031274) 
 
           |-50      |-40      |-30      |-20      |-10      |1        |11       |21       |31       |41       
Mouse     TCACCTGAT  CAGTGCTT  GCAAGGTAAGTGCCCTGGATTTTTTCCCCCTTC A AGAGGAAACTTCCCTA  GAAACTGCTCTTG  GGAAGGTGAACAGAAAGTGT
                     (BRE+)                                                         (MTE+)                        
Human     AAAGAATCTGCT  CTATGCTA  ACCAAGGTAAGTGTCCTGGATTTTTTTT  TT A ATGGG  GAAATGATCCA  GAAACTGCTTTCA  CAAAAGATGAATGTTTTTCT
                        (BRE+)                                (INR+)                  (MTE+)                        
    478 Mouse: 1810011K17Rik (226591, AK049784) 
Human: MGC3794 (261726, AL049670) 
 
           |-50      |-40      |-30      |-20      |-10      |1        |11       |21       |31       |41       
Mouse     GGCGGCAGGGGGCGGGGCCGAGATGGTAACGGACGCGCCCAGGAGCCGCG C TGCAGAGAGGGCCATCG  TGCGACCGCTGCC  GCAGGCGCTTGCTCCGAGT
                                                                                   (MTE-)                       
Human     CAGGGGGCGGGGCCG  GGCATGGT  AACGGCTCGGAAGCCTAGGAGGCTGGG C CGGAGGGAGGCGGA  GGAACCGGTGTTC  GCCGCCGCCGCTGCTTCAGCTT
                           (BRE-)                                                 (MTE+)                          
    479 Mouse: C030014K22Rik (226610, AK047703) 
Human: LOC149297 (149297, AL580399) 
 
           |-50      |-40      |-30      |-20      |-10      |1        |11       |21       |31       |41       
Mouse     NNNNNNNNNNNNNNNNNNNNNNNNNNNNNNNNNNNNNNNNNNNNN  NNTTT G CC  AGGCGCGGATCCGGCGCGA  GAACTT  GTGGTGTACGATGTGTGCGCCA
                                                         (INR-)                      (DPE-)                       
Human     CGCCGTGCCGGCACCGGGCTGCAGGATGGGCTGTATCCAAAGCAT  CACCT G CA  AGGCGCGGATCCGGCGCGAGA  ACATCGTGGTGTA  CGATGTGTGCGCC
                                                         (INR-)                            (MTE+)                 
    480 Mouse: Ndufs2 (226646, AK078474) 
Human: NDUFS2 (4720, AW753468) 
 
           |-50      |-40      |-30      |-20      |-10      |1        |11       |21       |31       |41       
Mouse     GCCCACCAGGGGGCGCGCGGGAGCGACTTCCGCCCGGCTCTCCTTCCT  AC A GTCTG  CAGCCCGGTAAAGATGGCGGCCCTGAGGGCGCTGCGCTGCCTCC
                                                            (INR+)                                              
Human     GCCAAGAAGGA  GGCGCGCT  GGAGTTACTTCCGCCCGGTTCTCCTTCCC  GC A GTCTG  CAGCCGGAGTAAGA  TGGCGGCGCTGAG  GGCTTTGTGCGGCTTCC
                       (BRE-)                                 (INR+)                     (MTE-)                     
    481 Mouse: MGI:1915033 (226747, NM_026375) 
Human: ELYS (25909, NM_015446) 
 
           |-50      |-40      |-30      |-20      |-10      |1        |11       |21       |31       |41       
Mouse     GTAGTCTTTCGCCTAT  CTGCTCCT  AGGAGGCGGAGAGACCCTGCTGTACG   T AAATGAA  TCTTTCTTCCCGGCGT  CGTCTCGGTTTTC  TCCCTTTGCGAAA
                            (BRE+)                              (INR-)                       (MTE-)                 
Human     AGTCTTTCTCCCCAC  CAGCTCCT  CGGAGGCGGAGAGACCCGATTTTTTTG   T AAATGAT  CCGCCCTTCC  AGGCCCTGCTTCT  CTTTCCTCCCTTTCCCTCC
                           (BRE+)                               (INR-)                 (MTE-)                       
    482 Mouse: Wdr26 (226757, NM_145514) 
Human: WDR26 (80232, NM_025160) 
 
           |-50      |-40      |-30      |-20      |-10      |1        |11       |21       |31       |41       
Mouse     CTTTTTTTTTCTTTGCAGGCAGAGAATGACCTGAATGAGCTAAAGCCTTT A GT  GCATTCTC  CTCACG  CTATTGTGGTAAG  AGGCGCACTTGAAATCTCTC
                                                                 (INR+)             (MTE+)                        
Human     TTATACCAATTTTTACAGG  CAGAAAA  TGACCTGAATGAACTAAAGCCTTT A GT  GCATTCTC  CTCATG  CTATTGTGGTAAG  AGGCGCACTTGAAATCTCTC
                              (TATA+)                              (INR+)             (MTE+)                        
    483 Mouse: Lyplal1 (226791, BC027340) 
Human: LYPLAL1 (127018, AK123628) 
 
           |-50      |-40      |-30      |-20      |-10      |1        |11       |21       |31       |41       
Mouse     CGCACACCCATGCTGGTGCTGCTTGCCTGCGCGCGCGGCCCCGTGACGCT A GG  GACGTGGC  CGCGCGCCTTG  CCATCTCTCCTCC  GCCATGGCTGCTGTG
                                                                 (INR-)                  (MTE+)                   
Human     GTGCTCCCGC  GCGTGCCT  GCG  TATTTGC  GTGCGCGGCCCCGCGCGGCAAC G CAGGGGCGGAACCGCATGACTG  GCAGTGGCATCAG  CGATGGCGGCTGCG
                      (BRE+)      (TATA+)                                                   (MTE+)                  
    484 Mouse: Lpgat1 (226856, NM_172266) 
Human: LPGAT1 (9926, BC034621) 
 
           |-50      |-40      |-30      |-20      |-10      |1        |11       |21       |31       |41       
Mouse     TCAGGGGCGCCGCCGCTTCCTCCCGCCAACCCGCCCCGCCGGCCGGGGCG C ACGCAGCCACCGGGCCCTGG  GGAGCGAGCGAGT  CTCCACGCCGCCCGGG
                                                                                      (MTE+)                    
Human     TCCCGACTCCCGCCGCTTCCTCCCGCCGCCCCTCCCCGCCAGTCCGGGCG A ACGCGGCCGGGCCCTTGGGGAC  CGAGTCTCGGCGC  CGCCGGGGAACGGG
                                                                                        (MTE+)                  
    485 Mouse: 4632411B12Rik (226976, AK030079) 
Human: FLJ10081 (55683, AK023813) 
 
           |-50      |-40      |-30      |-20      |-10      |1        |11       |21       |31       |41       
Mouse     AGAGCCCAA  GGCGCCGG  GAGGTAAGGCGCACGCGCATTGGGGTCCGGCTC G GGCGAGCGCGCGGCCGACAACTGCGGGTG  AGACGC  GGTTTTGCCCCGGC
                     (BRE-)                                                                  (DPE+)               
Human     GCGCCGGGGCGTGGA  GGCGTTAA  CGCGCACGCGCTTAGGGATCCGGCCGT G GCCGAGCGCGCGGCCGTA  AGACCGCGGGTGA  GAGCAGCGTTGGCTTCGA
                           (BRE-)                                                     (MTE+)                      
    486 Mouse: Actr1b (226977, AK088839) 
Human: ACTR1B (10120, BC010090) 
 
           |-50      |-40      |-30      |-20      |-10      |1        |11       |21       |31       |41       
Mouse     GAGGGGAGC  GGCGCGGT  GTCTGGAGTCGCATCCGTCCAGGTCTCCCCGCG A ACCTTGTAGCCCGCCGGTCCTCCTGCAGCCCGCCTGCTGGGCAGGGCCG
                     (BRE-)                                                                                     
Human     GAGGGCAGC  GGCGCCGC  GTCGGGAGCCGCCGCCGTCCCGGTCCTCCCGCC C GCCCGCCCATCCGGTGCCTCCT  GCAGCCCGCCTGC  TGGGCAGGGCCGGC
                     (BRE-)                                                               (MTE+)                  
    487 Mouse: Eif5b (226982, BC040746) 
Human: EIF5B (9669, NM_015904) 
 
           |-50      |-40      |-30      |-20      |-10      |1        |11       |21       |31       |41       
Mouse     GCGGCGGGGCGATGCGCT  TGCGTACT  GAGGGCTCACACCATATGTGCCCT G TC  CCAGTGCG  CGGGTCTGTGG  AGAGCCGGGTGCG  AGAGGCGGCGGCGCG
                              (BRE-)                               (INR+)                  (MTE+)                   
Human     GCGGCTGGGCG  ATGCGCTT  GCGCACTGAGAACTCACACCATATGTGTCCT G TT  CCAGTGCG  CGGGTCTGTGG  AGAGCCGGGTGCG  AGCGGCGGCAGCACG
                       (BRE+)                                      (INR+)                  (MTE+)                   
    488 Mouse: Slc9a2 (226999, L11236) 
Human: SLC9A2 (6549, BC035787) 
 
           |-50      |-40      |-30      |-20      |-10      |1        |11       |21       |31       |41       
Mouse     GCTGAGCGCGGCCGAGGGACCGCGCAGTGGCCTCCTGTCGCTGCGCGCTC C CCTCCGGGCCCTGCGAGGTTCACACCCACC  GAACTG  GTTGGCGACAGGG
                                                                                            (DPE-)              
Human     TGAGGGCTGCTGAGGGTACGCGCAGCGGCCTCTCGTCGCCCTG  CACGTGC C   TCGCCAGGCAGTGCGCCTGCTCGCAGCGA  GGACCT  AGCCCTCTGGTTGC
                                                       (INR-)                                (DPE-)               
    489 Mouse: Pms1 (227099, NM_153556) 
Human: PMS1 (5378, NM_000534) 
 
           |-50      |-40      |-30      |-20      |-10      |1        |11       |21       |31       |41       
Mouse     CGATTGGCTGCCAG  CCGCGCCA  ATCCCACTTCCTGCTGTGGGCGGGCC  TT A GAGGA  GCGCGAGCGC  GGGTCCTGCTGAG  CGCAGCCGTGCTTCGGTCCGA
                          (BRE+)                              (INR+)                 (MTE-)                         
Human     TGGCTGCGAG  CAGCGCCA  ATCTCACGTTGCCCCCGGGCGAGGCGGGAC  TC A GTGCC  GCGCTCTC  TGCACCCGCTCTG  CCGCGCGCGTGCGTGCTGGGTGC
                      (BRE+)                                  (INR+)               (MTE-)                           
    490 Mouse: Ormdl1 (227102, AK052927) 
Human: ORMDL1 (94101, NM_016467) 
 
           |-50      |-40      |-30      |-20      |-10      |1        |11       |21       |31       |41       
Mouse     ATCGGCTGCTCGCTAGAGCAGCTTGGCAGCCTTCCGGAAGCGGTGGGGCG C A  CCAGTAGC  TCTAGCCAGAGGACCTACTGTGGCTGCCGCGGGAATTGTC
                                                                (INR+)                                          
Human     ATCCGCTGGCA  GCGAGTCT  GTCCGGGAAAGCTTCCGCAAGCTGCGCAGCG C CG  GGCCTGAG  GGCGT  GTATCCGCGGCCG  TAGCAGCCGGGCTGGTCCTGC
                       (BRE+)                                      (INR-)            (MTE+)                         
    491 Mouse: Mpp4 (227157, AK044600) 
Human: MPP4 (58538, AF316032) 
 
           |-50      |-40      |-30      |-20      |-10      |1        |11       |21       |31       |41       
Mouse     TCAGGTGACTGGCAGCTAGCAGCCTCCTGGTGCTTCCTGCTTGCTTCTCT C TTGCCAGGAGCAACACTGTGTTT  TCATCCTTGCAGG  TAAGAGTCTGCTC
                                                                                         (MTE+)                 
Human     AGGTGACTTTGGCAGCTAACAGGCCACTAGTATCCTACTAAAGCTTT  TGT C TGGA  TAGGAGCAACATGCATGTTTACAGTCTTGCAGGTAAGAGACCTTG
                                                           (INR-)                                               
    492 Mouse: BC010584 (227195, BC010584) 
Human: FLJ20309 (54891, NM_017759) 
 
           |-50      |-40      |-30      |-20      |-10      |1        |11       |21       |31       |41       
Mouse     TAAGCAGAGGCGGCCCCAGGCCCGGCCCACATCCCCGGGCTCCCGTAGGG C GGCGGGCTGGGCCGCGTAGGCAGGGCGGGCGGCGAGCGCGCTTACGTGA
                                                                                                              
Human     TAAGCAGAGGCGGCCCCAGGCCCGGCCCACATCCCCGGGCTCCCGTAGGG C GGCGGGCTGGGCCGCGCAGGCTGGGCGGGCGGCGAGCGCGCTTACGTGA
                                                                                                              
    493 Mouse: Ndufs1 (227197, BC006660) 
Human: NDUFS1 (4719, NM_005006) 
 
           |-50      |-40      |-30      |-20      |-10      |1        |11       |21       |31       |41       
Mouse     AGACGGGGAAAAACAGTCGAAT  CTGTGTT  TCTAGTCCCTCCTGGCTTTCC G TCCTCCAGGTCGGGCTGACAGA  GGTTGC  CGTGGCCGCCATATTGACTTA
                                 (TATA-)                                              (DPE+)                      
Human     TTGCCGACAAACTTGTCGAATG  CAAGGAT  TCTAGTCCCTCCGGGCTTTCC G TTCTCCAGGCCCGGCTGACAG  AGTTAG  CCGAGGCCGCCATATTGAATAA
                                 (TATA-)                                             (DPE+)                       
    494 Mouse: Cps1 (227231, AF134407) 
Human: CPS1 (1373, AY166970) 
 
           |-50      |-40      |-30      |-20      |-10      |1        |11       |21       |31       |41       
Mouse     AAGGAGGAGCTGTAGATA  GGTTATT  TAATGGCAGAATGAATGG  GGAGTCA A   AGATCGCTGTGCAG  TCAGCCTACAGCC  T  CAACTG  TACTGTCGCCACACA
                             (TATA-)                     (INR+)                     (MTE+)      (DPE-)                
Human     AGGAGGAGCTGTGGCTGA  AGACATT  TAATGGCAGAATGAATGG  AAATTCA A   AGATCGCTGTGCAG  TCAGCCTTAAACA  C  TGACTG  CACCCCTCCCAGATT
                             (TATA-)                     (INR-)                     (MTE+)      (DPE-)                
    495 Mouse: Aamp (227290, BC080766) 
Human: AAMP (14, CR590201) 
 
           |-50      |-40      |-30      |-20      |-10      |1        |11       |21       |31       |41       
Mouse     CTTTTAACGCTTCTCTCAAAGCAGATGATCTGGCCCAGGAGATGGAAGAC G T  GGACTTTG  AGGAAGAGGAAGAAGAAGAAGAGGGCAATGACGAGGGCTG
                                                                (INR+)                                          
Human     CTTCTAACCCTCTCCCCAAAGCAGATGACCTGGCCCAGGAGATGGAA  GAT G TGGA  CTTTGAGGAAGAAGAGGAGGAAGAGGGCAACGAAGAGGGCTGGGT
                                                           (INR-)                                               
    496 Mouse: Aamp (227290, NM_146110) 
Human: AAMP (14, CR590201) 
 
           |-50      |-40      |-30      |-20      |-10      |1        |11       |21       |31       |41       
Mouse     TACTTCCTCCCGGGAGGAGGGGCTCGAGTTCCGCGTCGTCGCGCAGAGCT G   ACTCTGGG  AGGCGTTTGG  GCCCAGAGAAGT  G  GATCG  GTAGCTTTCGCCG
                                                               (INR-)                 (MTE-)    (DPE+)              
Human     TACTTCCTGCCGGGAGGAGGGGCTCGAGTTCCGCGTCGTCGCGCAGAGCT G   ACTCTGGG  AGGCGTTTGG  GCCCAGAGAAGTG    GATCCG  CCGCTTGCGCCG
                                                               (INR-)                 (MTE-)     (DPE-)             
    497 Mouse: Ctdsp1 (227292, NM_153088) 
Human: CTDSP1 (58190, AF258582) 
 
           |-50      |-40      |-30      |-20      |-10      |1        |11       |21       |31       |41       
Mouse     GAAGCTCGCGGGGATCCCTCCCTCCCACCCTCCCCTCCCCCCCGCGCC  CC G ATTCC  GGCCCGAGCCGGGGGGGAGGCCGGGCGCCGGGGCCAGAGTCCGG
                                                            (INR+)                                              
Human     GAGCTCGCGGGGATCCCTCCCTCCCACCCCTCCCCTCCCCCCCGCGCCC  C G ATTCCG  GCCCCAGCCGGGGGGGAGGCCGGGCGCCCGGGCCAGAGTCCGG
                                                             (INR-)                                             
    498 Mouse: BC038286 (227298, AK034667) 
Human: C2orf17 (79137, AK074983) 
 
           |-50      |-40      |-30      |-20      |-10      |1        |11       |21       |31       |41       
Mouse     ACGTGACGCTTGACCGC  AGGTTTT  TTTGCTCGCCCGTGCGTGCGCCCCCT T C  CGTCTGAC  GCGCCCCCGGCGGCGGCCGCGCAGCCCTGGCTCCTCGCGG
                            (TATA-)                               (INR-)                                          
Human     AGTCACGTGACGCTCGTCCGCAACCTCTGCTGTCCTCCGCGGCGCCCCCT T C  CGCCTGAC  GCGCCCCCGGCGGCGGCCGCGCAGCCCTGGCTCCTCGCGG
                                                                (INR-)                                          
    499 Mouse: Dner (227325, BC034634) 
Human: DNER (92737, NM_139072) 
 
           |-50      |-40      |-30      |-20      |-10      |1        |11       |21       |31       |41       
Mouse     CAGCCTCCGTGGGCCCCGCCCCCGGCCCCGCCCGCGGTTGCCCGGG  GAAC C ACG  GGCTCCCGCAGCTGCAGGAGGCGTCC  GGTCCC  GCTGAGCAGCACTA
                                                          (INR-)                             (DPE+)               
Human     GCCCCGCCCCGCCCCGCCCCGCAGGCCCCGCCCGCCGTTGCCCGGG  GAAC C GCG  GATCCCGCAGCTGCAGGAGGCGCCCGGCCCGGCGGAGCAGCCCTAG
                                                          (INR-)                                                
    500 Mouse: Slco4c1 (227394, AK049253) 
Human: SLCO4C1 (353189, BI764421) 
 
           |-50      |-40      |-30      |-20      |-10      |1        |11       |21       |31       |41       
Mouse     GGAGACTAGTCCGGCCTTAGGGCGCTGTCACCTGCCCAGGGGCGGAG  CCA G AAAA  GAAACCAGC  CTTTGCCGATCGT  ACTCACACAGGAGTCTAGAGAAC
                                                           (INR+)                (MTE-)                           
Human     CGGGAAGCTTCCCGCACGGGGGCGCTGTCACCTGCCTGTGGGAGGAG  CCA G AGAG  GGACCTGGCTCT  GCTGCTCTGAAGC  ACCGGAGTCGGGAGAACCCA
                                                           (INR+)                   (MTE+)                        
    501 Mouse: AW555814 (227399, AK129140) 
Human: KIAA0433 (23262, AB007893) 
 
           |-50      |-40      |-30      |-20      |-10      |1        |11       |21       |31       |41       
Mouse     CCTGTGGAGGGCGGGCAGGGAAGAGGCGTGTCGGAGGCTCCTGA  GGTGTG C C  AGTGCCAGCGGTTCCGCGGTGGCCGGCGAGGGAAGCTTCAGGTCCACG
                                                        (INR-)                                                  
Human     GAGACCCGAGGAGGGCGGGGAAGAGGCGTGACGGAGATTCCTGA  GGTGTA G T  AGCCTGAGGTTCCCTTATGTGG  CCCTATAGCTGTT  ACTGAAGGAAGTA
                                                        (INR-)                             (MTE-)                 
    502 Mouse: 2310035C23Rik (227446, AK035724) 
Human: KIAA1468 (57614, AK091160) 
 
           |-50      |-40      |-30      |-20      |-10      |1        |11       |21       |31       |41       
Mouse     GTGGGTGGGGCTGAGGCGGGGACGGGGCGGGGCGAAGATGGGACCAAG  CC A GTTCT  CGCGAGACTAGGGACCAGGA  AGACGC  CTGCAGAGTCGGCTACTG
                                                            (INR+)                       (DPE+)                   
Human     GGCGGGGCTGGGGCGGGGCTAGATCGGGGAGAGGGGCAGGGGCACGCT  CC A GTTCT  CGCGAGACTGGAGACCAGGA  AGACGC  CTGCAGAGCCGGGCTGCT
                                                            (INR+)                       (DPE+)                   
    503 Mouse: Zcchc2 (227449, AK046562) 
Human: ZCCHC2 (54877, CD652412) 
 
           |-50      |-40      |-30      |-20      |-10      |1        |11       |21       |31       |41       
Mouse     CGTTGATTGGCT  GGAGTGCG  GTGCGCGGAGCCCGCCAGCGTCTCCGC  CCA G CCCC  GCTGCTCCTCCTCCTCCTCGCCAAGCCGCTCTGCCAGCGCCGGCT
                        (BRE-)                               (INR+)                                               
Human     TGCTGATTGGCGGA  GGTGAGGT  GCGCGGAGCCCGCAAGCTTCTCCGCCCG G CC  CGGCTGCT  CCACCTCCTCA  CCCAGCCGCTCCG  TCCTCACCGGCTCGC
                          (BRE-)                                   (INR-)                  (MTE-)                   
    504 Mouse: Plekha6 (240753, AK173074) 
Human: PLEKHA6 (22874, BE158366) 
 
           |-50      |-40      |-30      |-20      |-10      |1        |11       |21       |31       |41       
Mouse     TGCCCTCCACAGCTGATGGCAGCTCTCTCCCCCCTTTTTTTTCT  CCACAG C C  ACAATGAAAGACGCAAC  ACATTTCGACATC  CAGTGACTGGCCAGATCC
                                                        (INR+)                        (MTE+)                      
Human     TCTCTGCCTTCTTCATCTTTACCACAACTCTTCTGTTTTTCCCTCTACAG C C  ACAATGAA  AGACGGAAC  ACATTTCTACACC  CAGTGACTGGCCAGGTCC
                                                                (INR-)                (MTE+)                      
    505 Mouse: E430019B13Rik (240754, AK040834) 
Human: LAX (54900, AK000347) 
 
           |-50      |-40      |-30      |-20      |-10      |1        |11       |21       |31       |41       
Mouse     TGTTTCCATCAACTGG  CAGAAAC  TAGAGAGTGGCCCTACTGCT  TCACACT G   GGAGCACAGACAGC  TGGGTGAGAAAGA  GCACGGCTCCTGCAGTGGGCAT
                           (TATA+)                       (INR+)                     (MTE-)                          
Human     CCAGAAACGTGAAGGCAGAGGCCACAGATTCTCCCTGAGCCACC  TCACTT G G  AAGCACCATGTCCGGATG  AGATCGCACTTCC  TGCAGTGGGCATTAGCC
                                                        (INR+)                         (MTE+)                     
    506 Mouse: A130072J07 (240832, AK080959) 
Human: LOC163590 (163590, AF464140) 
 
           |-50      |-40      |-30      |-20      |-10      |1        |11       |21       |31       |41       
Mouse     CCGCGGGTTCCTCCCTGCGGAGCTCACGTCACGTGGGCGGTACAGGCG  AC A TTCTG  CCGTGGCG  GCTGGGGGTTGC  A  GACCA  GCGCTGTGGAGCTGGAGG
                                                            (INR+)               (MTE-)    (DPE+)                   
Human     GCGCCTTCCGGCGGAAAGTG  TTTGTCA  TATGGGCGGGGACCCTGGACG  AC A TTCGG  GCCGCGGCG  GCCGCTGGGTGCA  GCCGAGCGGTGTGGAGCGGAGA
                               (TATA-)                        (INR+)                (MTE-)                          
    507 Mouse: Zbtb37 (240869, AK078748) 
Human: ZBTB37 (84614, AK057310) 
 
           |-50      |-40      |-30      |-20      |-10      |1        |11       |21       |31       |41       
Mouse     GGTGACTGATTTCCTTCCGGCACGTCGCTCGCTCCGGAGGAGGGGGCGGG G   GGTTTCAC  TTCCGGGACGG  TGTTCCGGCCCAT  TCCGGCCCATTTCCACC
                                                               (INR-)                  (MTE+)                     
Human     GCTGAGTGATTTCCTTCCGGCACGTCGCCCGCTCCGTGGGAGGGGGTGGG G   GATTTCAC  TTCCGGGACGG  TGTTCCGGCCCAT  TCCGGCCCATTTCCACC
                                                               (INR-)                  (MTE+)                     
    508 Mouse: Scyl3 (240880, AK030824) 
Human: SCYL3 (57147, AL117233) 
 
           |-50      |-40      |-30      |-20      |-10      |1        |11       |21       |31       |41       
Mouse     CCGCCCCCTTACTTGTCTGAAGCCAGAGGTGGCCATTTTGGATTCGGGCG   G AAATGAA  CTCAAAA  GCGGCTGGTTAAA  AACCCAAACATGAAAACCGGGA
                                                              (INR-)              (MTE-)                          
Human     CACCTCTACTGTTTGC  TACAAGT  GGCCAGCAGCCATTTTGGATTTGGGCG   G AAATGAA  ATTAAAAC  TGTGCTGTTAAAA  GCCTAAAAATTCAAGTCAAGA
                           (TATA+)                              (INR-)               (MTE+)                         
    509 Mouse: Adamts4 (240913, NM_172845) 
Human: ADAMTS4 (9507, AA578965) 
 
           |-50      |-40      |-30      |-20      |-10      |1        |11       |21       |31       |41       
Mouse     CACAGAGGGCGGTTCT  GGATGCT  T  AAAAGA  GCTGGAGGGGAGAG  AGGCTG G G  GAGAACCCGGGGAAG  ACCCACAGATACA  CAGAAACGAGAGAGACAGAA
                            (BRE+)  (TATA+)                 (INR-)                      (MTE-)                        
Human     CAATCGCAGAGGCTCA  CCATGCT  T  AAAAGA  GCTGGCGCGGAGAG  AGGCTG G G  GAGAACCCACAGGGAGACCCAC  AGACAC  ATATGCACGAGAGAGACAGA
                            (BRE+)  (TATA+)                 (INR-)                         (DPE+)                     
    510 Mouse: Carf (241066, AK044738) 
Human: ALS2CR8 (79800, AB053309) 
 
           |-50      |-40      |-30      |-20      |-10      |1        |11       |21       |31       |41       
Mouse     CGACGGTGGCTGAGAAGGGTCTAATCTGATGGACCTGCGCTGCGCCTCCA G GAGGGGTGCGCCTGCGCATTGTGCT  GGTCTC  CACGGCGCGGCCTCGGAG
                                                                                       (DPE-)                   
Human     ACGGTGGCCGATAGTGGTCCTACTGTCGTGGGACTGCTCTGCGCGCTCCC G GTGGGGCGCGCCTGCGCATTATGCT  GGTCTC  CATGGCGGGGCCTCGGAG
                                                                                       (DPE-)                   
    511 Mouse: Prkag3 (241113, NM_153745) 
Human: PRKAG3 (53632, NM_017431) 
 
           |-50      |-40      |-30      |-20      |-10      |1        |11       |21       |31       |41       
Mouse     AGAGAACCCGATCTCTCAAGGGCCTGCCGCCAGGGACCAGCCC  AGAATAG T   GCCACCCTGCCGCCGCCT  GTCGCGCACTTGC  TAACGGACTAGAGCTGGC
                                                       (INR-)                         (MTE-)                      
Human     GAGAAACCGATCCTGGCAGGGCAGGGTGCCCGGGGCCGGGCCC  AGAATAG T   GCAGCCCAGCCACAGT  GTCGCACACTTGC  TCTCAGTTGGTCTGGGGCTG
                                                       (INR-)                       (MTE-)                        
    512 Mouse: Accn4 (241118, AJ242554) 
Human: ACCN4 (55515, AJ271643) 
 
           |-50      |-40      |-30      |-20      |-10      |1        |11       |21       |31       |41       
Mouse     CGCTTGCTCCCTCGCTCACTCGCTTGCTCGCAGGGACACACGCAG  GGGCT G AC  AGCTGTGCGGGTGCT  GATAAGGGAAGCC  ACAAGGAGACGATCGAGGA
                                                         (INR-)                      (MTE-)                       
Human     CGCTCGCTCCCTCGCTCACTCGCTCGCTCGCAGGGACACACGCAGGGGCT G   ACAGCTGT  GCTGGTGCT  GATAAGGGAAGCC  ACAAGGAGACGATCGAGGA
                                                               (INR+)                (MTE-)                       
    513 Mouse: A830043J08Rik (241128, NM_173425) 
Human: FLJ22746 (79843, NM_024785) 
 
           |-50      |-40      |-30      |-20      |-10      |1        |11       |21       |31       |41       
Mouse     ACTGGGCGTGGGTC  TCAGCAT  TATAGGTTTTTAGGTCAAACCAAAACCCT G G  GGAATGCC  CGAG  AAAGCCAAGCAAA  T  AGTTAC  ATCTGCTCAGACCGGA
                         (TATA-)                                  (INR-)           (MTE+)      (DPE+)                 
Human     CATTTGGGTGTTTC  TCAGGAT  TATAGGTTTTTAGGTCACGCAGAA  ACCCT G GG  GAACGCCTGAAAAGGCTAAACACAAGC  AGTTAC  ATTATGCACAAGCT
                         (TATA-)                           (INR-)                              (DPE+)               
    514 Mouse: Serpinb13 (241196, AK030670) 
Human: SERPINB13 (5275, AJ001696) 
 
           |-50      |-40      |-30      |-20      |-10      |1        |11       |21       |31       |41       
Mouse     AGGCAGAGAGCACTCAC  TATAAAT  TAAAGACCTCAGCTACTCACTCTAC  A T AGATTC  CTAGTCTCTG  TCCAGCCACTGC  T  GTCTC  TTCAGAGCCAGGTAA
                            (TATA+)                            (INR+)                 (MTE-)    (DPE-)                
Human     AAGCAGATGTGGAGAAC  TATAAAT  TAAGGATCCCAGCTACTTAAT  TGACT T AT  GCTTCCTAGTTC  GTTGCCCAGCCAC  CAC  CGTCTC  TCCAAAAACCCGA
                            (TATA+)                        (INR+)                   (MTE+)        (DPE-)              
    515 Mouse: Cdh7 (241201, NM_172853) 
Human: CDH7 (1005, NM_004361) 
 
           |-50      |-40      |-30      |-20      |-10      |1        |11       |21       |31       |41       
Mouse     GAGTTTCCCGT  GGCGTTCC  CAAGTTACTGCGTGTTGTGTTGAGACGTGCT T C  TGCCTGGC  AGGACAGGCTTGAGGACTG  AGTTCT  CCCGAGATCCTCCAG
                       (BRE-)                                     (INR-)                      (DPE+)                
Human     GAGTTTCCCTT  GGCGTCCC  CAAGTTACTGCGTGTTGCAGTGAGATGTG  GC T CTGCA  AGGCAGGACAGGCTTGT  GGACGG  AGGCGGGGGCGTTTGGCCCGG
                       (BRE-)                                 (INR-)                    (DPE+)                      
    516 Mouse: Fcgr3a (246256, AK076532) 
Human: FCGR3A (2214, M90743) 
 
           |-50      |-40      |-30      |-20      |-10      |1        |11       |21       |31       |41       
Mouse     CCTCTCTTCTAGTCAAATAGGGCC  CTTAAGG  GTTCCGGATATCTGTGGTG   A CATTTTC  TATCTGCT  TCAGCAGCATGTG  GCAGCTACTACTACCAACAGC
                                   (TATA+)                      (INR+)               (MTE+)                         
Human     CCTCCTGTCTAGTCGGTTTG  GTCCCTT  TAGGGCTCCGGATATCTTTGG  TG A CTTGT  CCACTCCAGTGTG  GCATCATGTGGCA  GCTGCTCCTCCCAACTGC
                               (TATA-)                        (INR+)                    (MTE+)                      
    517 Mouse: Rgs13 (246709, AF498319) 
Human: RGS13 (6003, CR599001) 
 
           |-50      |-40      |-30      |-20      |-10      |1        |11       |21       |31       |41       
Mouse     TTTGCATAGA  TACGTGTC    TGTAAAA  CTAGAATTTCATAGCACAGGGACCC   G GAGTGCC  ATAGAAGGTAATTATAC  AGACAG  TGGCGTGTTTTTATGCCAG
                      (BRE-)   (TATA+)                            (INR-)                    (DPE+)                    
Human     CTCTTTGCATAGA  TACGTGTG    TGTAAAA  ACAGAACATTCATACTGAGGCC   A GAGTGCC  ATCGAAGGTAATTATAG  AGACAG  TAAAATCCTTTTACTCTGG
                         (BRE-)   (TATA+)                         (INR-)                    (DPE+)                    
    518 Mouse: 4930429M06Rik (252876, AK014287) 
Human: FLJ20125 (54826, CR621255) 
 
           |-50      |-40      |-30      |-20      |-10      |1        |11       |21       |31       |41       
Mouse     ACGACCCGCCTGAGCCTTCCTGTTCCACAAGCGCCACCAGAACTCCGACC C CCGGCAGTTCCGCTTCCGGT  AGCACCAGATAAA  TCGCGAGAGGAGAGTT
                                                                                      (MTE-)                    
Human     CTTTCTGACCGTGGTTCCAAGGTTCCCCAAAGGACTGCCCTCTAAAGGAC C CGGGTAGTTCCGCTTCCGGC  AGCGCGAGATAAA  TCACGAGAGGAAGCTT
                                                                                      (MTE-)                    
    519 Mouse: Fev (260298, AY049086) 
Human: FEV (54738, NM_017521) 
 
           |-50      |-40      |-30      |-20      |-10      |1        |11       |21       |31       |41       
Mouse     CGGAAATTGCGGCCCCTGCCCGCGCGGGGCCGGAGGGTTGCAAGCGGCCT T G  TAGCTGGG  AGCATCGCTCCA  GGACCC  CCTCCCTCCCCGCCACACCCTG
                                                                (INR-)               (DPE-)                       
Human     GGAAATTGCGGCCCCGGGCCGCGCGGGGGCCGGAGGGTTCCAAGCGGCCC C T  TAGCTGGA  AGCGTTTCTCCA  GGACCC  CCCCGCAACCCCCGCCACGCCC
                                                                (INR-)               (DPE-)                       
    520 Mouse: Creg2 (263764, AB046110) 
Human: CREG2 (200407, NM_153836) 
 
           |-50      |-40      |-30      |-20      |-10      |1        |11       |21       |31       |41       
Mouse     CGCAGGGGGGCGCCCCTGGCTGGCCGCTGCTTGGTCAGTGGTCCCCG  CCA C AGGG  CGCGTCGCCT  CCAAGAAGATGTC  GC  TGTCCG  GCAGGGAGCGTCCT
                                                           (INR+)                 (MTE-)       (DPE-)               
Human     TGGGGCGCTGCCGGGCTCCCCGGCACTAGCGCTGCTGGCGGCCCCGGCGG C CG  GGCGTGCT  GCCTG  CAAGATGTCCGTG  CGCCGCGGCCGGCGGCCGGCG
                                                                 (INR-)            (MTE+)                         
    521 Mouse: Optc (269120, AF333981) 
Human: OPTC (26254, BF995083) 
 
           |-50      |-40      |-30      |-20      |-10      |1        |11       |21       |31       |41       
Mouse     GTGTGTGTGTGTGTGTGTGTGTGTGTGTTCACCCCCACACAAA  GAATTCC T   GTTCATTAAGTGAA  GAAATGAGATTAG    GGACCC  CTGAGGAAAAAGGAAG
                                                       (INR-)                     (MTE-)     (DPE-)                 
Human     GAGCAGGAGCTGGAGGTGGGGGGTGAAGCGGAAGGTGGGGGGA  GAATTCT C   TTTTGTCAAATACA  GGAATGGGGCAGG    GGACCC  CCTGAGGAAAGAGGAG
                                                       (INR-)                     (MTE+)     (DPE-)                 
    522 Mouse: Optc (269120, NM_054076) 
Human: OPTC (26254, BF995083) 
 
           |-50      |-40      |-30      |-20      |-10      |1        |11       |21       |31       |41       
Mouse     GTGGGTTCTGCTAAGGCCATCTGTTCTGAGGGTAGAGTCACTGTGTTCTC A   GGATTCCT  GAGAT  GAAGTTGTAAGAA  GGTGAGCCTTGTGAACTCAGAAT
                                                               (INR-)            (MTE+)                           
Human     ATGAGTTCTG  CCAAGCCA  TCTGCTCTGAGGGCCTGGAAACACACGGATTC A   GGACTCCT  GGGGAAGA  AGGTGGAAATGGG  GAGGGTCATATGAACTAAAG
                      (BRE+)                                     (INR-)               (MTE-)                        
    523 Mouse: Inpp4a (269180, AK089671) 
Human: INPP4A (3631, AK057550) 
 
           |-50      |-40      |-30      |-20      |-10      |1        |11       |21       |31       |41       
Mouse     TGGGGCGCGCA  TGCGCGGT  GAGGGGGCGGAACCGGGGCGGGGTTGTGCGC T GT  GTTCTGAG  AGAAGGCG  GTGGCAGGATTCG  GGCTGCGCGGGGACGGCG
                       (BRE-)                                      (INR-)               (MTE-)                      
Human     CTCAGCGCGCAT  GCGCGACA  AGGGGGCGGGGCCAGGGCGGGGCTGCGCCC G G  CGTCTAGA  GCGGCGGCGGCT  GGCTAGGGCTGCG  GCGCGCGTGGAGGGT
                        (BRE+)                                    (INR-)                   (MTE-)                   
    524 Mouse: Inpp4a (269180, NM_030266) 
Human: INPP4A (3631, NM_001566) 
 
           |-50      |-40      |-30      |-20      |-10      |1        |11       |21       |31       |41       
Mouse     CGCTGGCCGCGTCTTTGGAGCGCGATCCTAGACTTGCGGTCCCCTTGCCG C GGGCTTCTCGGGGA  GCCGTGCGATGCT  ATACAACGCGCCCGCCGGCTGC
                                                                                (MTE-)                          
Human     CTGCGGTGGCGTCTTTTGAGAACAATCCGAGCCCGGCCGACCC  CCAGCCC C   GTGAAGACGCGGCCGCC  CCAGCAGGGAGCC  GGGTGCCGAGCGGGCGCGT
                                                       (INR+)                        (MTE+)                       
    525 Mouse: B3gat2 (280645, AB055903) 
Human: B3GAT2 (135152, AY070108) 
 
           |-50      |-40      |-30      |-20      |-10      |1        |11       |21       |31       |41       
Mouse     ACCGGAGCACTAGGA  GGACGCCC  GAGTCTGCAGCGCCCCCGCCCCC  TCGC T TTT  TCTTTCTTTCCCT  GCTTTGGGATCTT  GC  TGTCTG  GAGCCGGGAGAG
                           (BRE+)                           (INR+)                    (MTE-)       (DPE-)             
Human     CAGGAGGACTTGGGA  GGACGCTG  GATTCTGCAGCGCCCCCGCCCCC  TCGC T TTT  TCTTTCTTTCCTT  GCTTTGGGATCTT  GCTGCTGGATCCGGAGAGGT
                           (BRE+)                           (INR+)                    (MTE-)                        
    526 Mouse: Usp37 (319651, AK035640) 
Human: USP37 (57695, AL832645) 
 
           |-50      |-40      |-30      |-20      |-10      |1        |11       |21       |31       |41       
Mouse     TGCGTGCGTCCGTACGTGTTTGCGTGTAGTCATAGTTCCCGCCATCAGCG   G GTTTAGG  TGTGCTAGGAGAGAGACCGGCCTCGCTCTCTGGGGCTAGTAG
                                                              (INR-)                                            
Human     TGCGTGCGTGCGT  GCGTGTTT  GCGTGTGGTCACAGTTCCCGCCA  TCAGCC G C  TCTCCCTGACTAGGGGTAG  CTAGCGGCCTCCC  TCCTTTGGGTTCGGTA
                         (BRE+)                           (INR+)                          (MTE+)                    
    527 Mouse: Usp37 (319651, AK030013) 
Human: USP37 (57695, AL832645) 
 
           |-50      |-40      |-30      |-20      |-10      |1        |11       |21       |31       |41       
Mouse     CCAAAGAATGTGTTAGAACCTAAGTGTTCCAAGTCTTGGTGTTCCCGCTA G   TGTCTGAA  AGGAAGGATGGT  TACAACGGATGCA  GGTGCAGTGGGGCGAG
                                                               (INR-)                   (MTE-)                    
Human     AGACAATGTGAGTTAGAACCTAGGTATTCTAGGTCTTGCTGTTTCCTCTG G   TGTCTAAT  GGTAGAATGGGAA  GGATGC  CCACAGCTGATGCAGGTGCAAT
                                                               (INR-)                (DPE+)                       
    528 Mouse: A230078I05Rik (319998, NM_177056) 
Human: MGC99813 (130612, BC080573) 
 
           |-50      |-40      |-30      |-20      |-10      |1        |11       |21       |31       |41       
Mouse     CAGGATGGTGCACCCTGAGTGACGTCAGGAGCAGAGGCCGGAGCTGTCCA T CAGCACCAAAGGCCG  CCAGCGGGCTCAG  GGCATGGGGCCGCGGCTCCGG
                                                                                 (MTE-)                         
Human     CTGGATGGTGCGCCCTGAGTGACGTCAGGAGCAGAGGCCGGAGCTGTCCA T CAGCACCAAAGGCCG  CGGGCGGGCTCAG  GGCATGGGGCCGCGGTTCTGG
                                                                                 (MTE-)                         
    529 Mouse: Ptpn7 (320139, NM_177081) 
Human: PTPN7 (5778, NM_002832) 
 
           |-50      |-40      |-30      |-20      |-10      |1        |11       |21       |31       |41       
Mouse     GGGCGGAGGGAGGAAGCT  GGCTTCCT  GAAACCTCTCAGTCCTCAAAGATA G AC  GGACTGAC  AGACAGCTGGCAAGAGGCAGCCTGGGGGACATAGCTTCT
                              (BRE-)                               (INR-)                                         
Human     GGCGGAGGGAGGAAGCT  GGCTTCCT  GGAGCCTTCTCAGCCCTCAAAG  ACA G ACCG  ACAGACAGACAG  ACAGCTGGCAAGA  GGCAGCCTGGGGGCCACAGC
                             (BRE-)                          (INR+)                   (MTE+)                        
    530 Mouse: B230209C24Rik (320705, BC042757) 
Human: C6orf65 (221336, BC036119) 
 
           |-50      |-40      |-30      |-20      |-10      |1        |11       |21       |31       |41       
Mouse     GCGGCGGCAGCGGCGGCGCGGGTGCGGGCGCGCGCGCATGGCTGGGGCCG   G GACCGAG  CGGCGCGCGCCCTCCAGCAGGTC  GGTTCG  GTGCGCGCGCGGC
                                                              (INR-)                          (DPE+)              
Human     CGCGCGGACATGGCTGCGGCGGTTTCGGCGGCGTCCGCGGGCTGCACCCG   G GCCTGAG  AGCCCAGCGCCCTCCCGCGGGGCCGCCCGCCAGTCCGCGCCG
                                                              (INR-)                                            
    531 Mouse: Arl7 (320982, AJ623276) 
Human: ARL7 (10123, BM918203) 
 
           |-50      |-40      |-30      |-20      |-10      |1        |11       |21       |31       |41       
Mouse     CGAGAAGACCCCGCCTGACCCCGGGAGACCGCGGGGCGTGGCGGGCGAGG   G GCGTGGC  CTGTGGGCGGGACTAGAGGGGCGGGGCCGGCGCCCTCCGGCC
                                                              (INR-)                                            
Human     ACCGCGCCTGACCCCCGGCGGCGGGCGGGGCGGGGCCCGGGGCG  GGTCCG G G  GGCGGGTCCGGGGCGGGGCGCGAGGGGGCG  GGTCCG  GGAGCTCTCCGG
                                                        (INR-)                                 (DPE+)             
    532 Mouse: MGC65590 (381306, NM_201364) 
Human: FLJ10706 (55732, AL354614) 
 
           |-50      |-40      |-30      |-20      |-10      |1        |11       |21       |31       |41       
Mouse     CCTCTCTATGGTTTGGTGGGGGCGGAGCTCTCTCAAAGCCTGCTGGGACG G CGTTCCAAGCTGTGGCGGGAG  TCGTGCGGATTCG  TTCGTCTCTCGGGGG
                                                                                       (MTE-)                   
Human     CTCTCTATGGTTTTGGAGCCGGCGGAGAGCGCGCAAGGGTTGGCG  GGACT G CG  AGTTTCCGGTCTGGGCTTTGGCG  GGTCTG  GTTTGAAGCTCTCCTGTT
                                                         (INR-)                          (DPE-)                   
    533 Mouse: 2010002H18Rik (381314, AK048653) 
Human: FLJ10326 (55699, NM_018060) 
 
           |-50      |-40      |-30      |-20      |-10      |1        |11       |21       |31       |41       
Mouse     AGACTTCCAAGCCGGAAAACCCCGCCCTTGCTCTTGCCCCGCCCCTTCCG C CCCGGAGCGCGTGC  GCAGTTCCTCGCC  GCCGAGCTCTTCAAGGGTCTCT
                                                                                (MTE+)                          
Human     TGCCCACCGCCCCGCCCCCACTACCCGCCGCTCCCTCCCCGCCCCTTCCG C TCCCGGAGCGCGTGCGCCCTC  TTACTCGGCTCCC  CTTGGTTTCCTGGGG
                                                                                       (MTE-)                   
    534 Mouse: 4833432M17Rik (381318, NM_198654) 
Human: C1orf48 (25936, NM_015471) 
 
           |-50      |-40      |-30      |-20      |-10      |1        |11       |21       |31       |41       
Mouse     GCGTTCCAGGGAGGGA  AGCATCCT  TCCGGTCTCAGCGATTCCCGCC  TTAG T TTA  AACCGGGAAGATGGCGGC  TGTCTC  TGAAACAGTGCTCGTCTCCGCC
                            (BRE-)                          (INR+)                     (DPE-)                       
Human     TTACACCAGTGTGAAC  CCGCTTTT  CCGCTCCCAGAGTGCCCCGCCC  ACAG T TCC  GACGAAAAATGGCGGGGTCTCCTG  AGTTGG  TGGTCCTTGACCCTCC
                            (BRE+)                          (INR+)                           (DPE+)                 
    535 Mouse: Abca2 (11305, NM_007379) 
Human: ABCA2 (20, CV322806) 
 
           |-50      |-40      |-30      |-20      |-10      |1        |11       |21       |31       |41       
Mouse     CGCGCAGAGGACAGGGCCGCGGCGCTGAAGCGGCGGAGCGCGGCCCGGCC A TGGGCTTCCTGCACCAGCT  GCAGCTGCTGCTC  TGGAAGAACGTGACGCT
                                                                                     (MTE+)                     
Human     CGCGCAGA  GGAGCGGG  CCGCGGCGCTGAGGCGGCGGAGCGTGGCCCCGCC A TGGGCTTCCTGCACCAGCT  GCAGCTGCTGCTC  TGGAAGAACGTGACGCT
                    (BRE-)                                                             (MTE+)                     
    536 Mouse: Abi1 (11308, AF420251) 
Human: ABI1 (10006, BX476599) 
 
           |-50      |-40      |-30      |-20      |-10      |1        |11       |21       |31       |41       
Mouse     TGGTCTGTGCGGAGCTCGGGTCCCCGGCGGACTCAGCTTCCTCTGTCTCT   T TAATGCG  AGAGGAAGCGATGCGGAGGGGTGGAAAATGGCAGAGCTGCAG
                                                              (INR+)                                            
Human     CGGGCTGTGAGGTGCT  CGGAGCCT  CGGCGGACCTTGCTGCCTC  TGTCTCT T   TAACGCGAGAGGAAGCGATGCAGAGGGGTGGAAAATGGCAGAGCTGCAG
                            (BRE+)                       (INR-)                                                   
    537 Mouse: Abl1 (11350, X07541) 
Human: ABL1 (25, CN284745) 
 
           |-50      |-40      |-30      |-20      |-10      |1        |11       |21       |31       |41       
Mouse     CTGAGAGGCGTCGGAGCTGGAACCGGGTGCCGGGTCCTGCGGCTGA  GGAC T CCT  AACGCGGCACACACC  CTCACTGGCTGGA  GAAGCCGGGTTCCTGGAC
                                                          (INR-)                      (MTE-)                      
Human     CTGAGCAGCGCTGGAGCCGGAGCCGGTTCCCGGGTCCTGCGGCTGAGGAG C CCCTCCGCTGTCCACGGCCC  CTACCGGCGGGGG  GCGGCTGGGTCCCTCG
                                                                                      (MTE+)                    
    538 Mouse: Abl1 (11350, U13835) 
Human: ABL1 (25, CN284745) 
 
           |-50      |-40      |-30      |-20      |-10      |1        |11       |21       |31       |41       
Mouse     CTGAAAAGTCTTGAACCCTCTTCTGGAAAGGGGCACCTATTATTGCTTTA T GGGGCAGCAGCCTGGAAAAGTTCTTGG  GGACCA  AAGAAGGCCTAGTTTG
                                                                                         (DPE-)                 
Human     CTGGAAGATCTTGAACCCTCTTCTGGAAAGGGGTACCTATTATTACTTTA T GGGGCAGCAGCCTGGAAAAGTACTTGG  GGACCA  AAGAAGGCCAAGCTTG
                                                                                         (DPE-)                 
    539 Mouse: Abl1 (11350, U14720) 
Human: ABL1 (25, AA461345) 
 
           |-50      |-40      |-30      |-20      |-10      |1        |11       |21       |31       |41       
Mouse     CGGGGCTGGGGGCCCGGGGTGGTGGGGCCTTCGCCGTGGCCACGGGA  CCA T GTTG  GAGATCTGTTTGAAG  TTGGTGGGCTGCA  AATCCAAGAAGGGGCTC
                                                           (INR+)                      (MTE-)                     
Human     GACCGAGCTGGGAGAGGGGTTCCGGCCCCCGACGTGCTGGCGCGGG  AAAA T GTT  GGAGATCTGCCTGAAG  CTGGTGGGCTGCA  AATCCAAGAAGGGGCTG
                                                          (INR-)                       (MTE-)                     
    540 Mouse: Acp2 (11432, AK128963) 
Human: ACP2 (53, NM_001610) 
 
           |-50      |-40      |-30      |-20      |-10      |1        |11       |21       |31       |41       
Mouse     CCGGGCGGCTGCAGCAGGTACCGCCCAAGGCGTTCCGGCCTCTTG  CTACA C AG  CCTACAACAGTGATGGCCGGC  AGACAG  ACTGGTTGGAGCCAGGCGGC
                                                         (INR+)                        (DPE+)                     
Human     GGGCGGCTGCTTGCAGGTGCCACCCAGCGGGTTCCAGCTTGTTTGCTGC  A T AGATTA  CAACGGTGATGGCGGGCAAGC  GGTCCG  GCTGGAGCCGGGCGGC
                                                             (INR+)                        (DPE+)                 
    541 Mouse: Chrna1 (11435, M17640) 
Human: CHRNA1 (1134, BC006314) 
 
           |-50      |-40      |-30      |-20      |-10      |1        |11       |21       |31       |41       
Mouse     CCAAGCTCCCTAAGCCAGGGTGGAGTAGGACCGGCAGCAAGCCGCTGGCG G   CCACAGCG  GCACCCACAGC  CCATGGAGCTCT  C  GACTG  TTCTCCTGCTGC
                                                               (INR+)                  (MTE-)    (DPE-)             
Human     GCACCCAGCCAGAGTGCCAGTGAGAAGCACAGGCCACCACTCTGCCCTGG T   CCACACAA  GCTCCG  GTAGCCCATGGAG  CCCTGGCCTCTCCTCCTGCTCT
                                                               (INR+)             (MTE+)                          
    542 Mouse: Chrna4 (11438, AK034228) 
Human: CHRNA4 (1137, X87629) 
 
           |-50      |-40      |-30      |-20      |-10      |1        |11       |21       |31       |41       
Mouse     CCCACAACCTCGAGCTCCCGCCCCGCCGCCGCACCCCGCGGGAC  GAGCTG C G  GAGCCCGGTTTGGGGACGAGCGGCCGCGACACGGGGCATGAAGTTGGG
                                                        (INR-)                                                  
Human     GCCCGGCGCTCCCTG  CCGCGCCG  CCGCCGCACCGCGCCCCACAGGAGAAG A C  GAACCGGG  CCCGGCGGCC  GAAGCGGCCCGCG  AGGCGCGGGAGGCATGA
                           (BRE+)                                 (INR-)                 (MTE+)                     
    543 Mouse: Chrna4 (11438, AB010002) 
Human: CHRNA4 (1137, NM_000744) 
 
           |-50      |-40      |-30      |-20      |-10      |1        |11       |21       |31       |41       
Mouse     CCTCCACATACCCTCGGGGCCCGGTCCGCCCCCTCCTCCCGCTCTCTCCC T CCTCCCTTCAGTCCCTCCCCCTCCTCCCCTCTCGCCGGCGGCTGGAGGC
                                                                                                              
Human     CCCCTGGGCGCGCGGTCCATCCCTGCTCATTCTCCCTGGCCCTTGCTCCC T CCTCCCTTTTCACTCTC  CGTTCCCTCCCCC  TCCCCTGCTCCCCCTCCTC
                                                                                   (MTE+)                       
    544 Mouse: Actc1 (11464, M59866) 
Human: ACTC (70, BC009978) 
 
           |-50      |-40      |-30      |-20      |-10      |1        |11       |21       |31       |41       
Mouse     CCCCGTCCATCAGAGAGC  TATAAAG  CTGCGCTCCAGGCGACTGACAC  CCA G TGCC  TGCCACCAGCG  CCAGCCCAGCTGA  ATCCAGCCGCCCCTAGCACGG
                             (TATA+)                         (INR+)                  (MTE+)                         
Human     CCCTGTCCATCAGCGTTC  TATAAAG  CGGCCCTCCTGGAGCCAGCCAC  CCA G AGCC  CGCTGCCGCCGGAGCCGAGC  CGACCC  GCCCCGCCGACGGTGAGTC
                             (TATA+)                         (INR+)                       (DPE-)                    
    545 Mouse: Acvr1 (11477, NM_007394) 
Human: ACVR1 (90, AL700573) 
 
           |-50      |-40      |-30      |-20      |-10      |1        |11       |21       |31       |41       
Mouse     TGCAGCTCCC  GGTCGCCT  CGAGCTCTCCGCCTCCCCGGGTTCAGCACC  CG A CCGCC  GCTGGACCAGAGGTGAGAGCA  GGTCGC  CGGCCACGCCGCCGGAG
                      (BRE+)                                  (INR-)                        (DPE+)                  
Human     CCTGCAGCGCCCGGCTGCCTCGCACTCCGCCTCCCCCGGCTCAGCCCCCG G CCGCGGCGGGACCCGAGGTGAGCGCA  GGTCGC  CGGCCGCGCCTGCTGGA
                                                                                        (DPE+)                  
    546 Mouse: Acvr1 (11477, L15436) 
Human: ACVR1 (90, AL700573) 
 
           |-50      |-40      |-30      |-20      |-10      |1        |11       |21       |31       |41       
Mouse     GCTCCCGGTCGCCGCCGCCGCCGCCGCCGCCCCCAAGCGCTGGGG  GAACT T TG  CCGCCGCCGGCTCCCCGCCGCCCCCAGCCGAGCCCCGCGCCGCCGCA
                                                         (INR-)                                                 
Human     TTTGCAGCCGCCGCCGCCGCCGCAGCCTCCCCCTCGGCGCAGGGGGAACT T TT  CTACTCTC  TGTGACGGTCTCCCCCGCCGCCCCCGGGGGCAAGCCCAG
                                                                 (INR+)                                         
    547 Mouse: Acvr2 (11480, AK041246) 
Human: ACVR2 (92, BC069707) 
 
           |-50      |-40      |-30      |-20      |-10      |1        |11       |21       |31       |41       
Mouse     GCTTCAGGACGGAACCGGAAGTGTAGGGGGCGGGGCCGGCGGAGGCCA  GG A GACCG  AAAACGCGGC  CGAGCCGGGAGCC  GGGAGCCGGAGCCACAGCCTG
                                                            (INR+)                 (MTE+)                         
Human     CTTCGGGCCGGAACCGGAAGTTTGGGGGGCGGAGCCCGGCGGAGGCCA  GG A GACCG  AAAACGCGGCCGAGCCC  GGAGCCCGGAGCT  GGAGCCAGAGCCTG
                                                            (INR+)                        (MTE+)                  
    548 Mouse: Ada (11486, U73107) 
Human: ADA (100, NM_000022) 
 
           |-50      |-40      |-30      |-20      |-10      |1        |11       |21       |31       |41       
Mouse     GCGGGGCTGTGCCGGGGCAG  CCCGGTA  AAAAAGAGCGTGGCGGGCCGCGG   T CTCTGAG  AGCCATCGG  GAAGCGACCCTGC  CAGCGAGCCAACGCAGACCC
                               (TATA-)                          (INR-)                (MTE+)                        
Human     GGGGCGGGAGGCGGGGCCCGGCCC  GTTAAGA  AGAGCGTGGCCGGCCGCGG C CACCGCTGGCCCCAGGGAAAGC  CGAGCGGCCACCG  AGCCGGCAGAGACC
                                   (TATA+)                                                (MTE+)                  
    549 Mouse: Ak1 (11636, AK089270) 
Human: AK1 (203, NM_000476) 
 
           |-50      |-40      |-30      |-20      |-10      |1        |11       |21       |31       |41       
Mouse     CATGCGTGCGCGCTGACAGCCT  TATAAAT  AGTCGCCTTTGCAGG  CGGCTG A G  AGGACAGGCAGGGCACTCACTGGCCCCAGCGCCCACCACACCCTCCCC
                                 (TATA+)                  (INR-)                                                  
Human     GCATGCCGCGCGCTGACAGCCT  TATAAAT  AGTCGCCTTTGCCGGCCGCCG C GA  GGACGGGC  AGGGCACGCACTGGCCCCGGCGCCCACCCGCACCCCTCC
                                 (TATA+)                           (INR-)                                         
    550 Mouse: Api5 (11800, BC007133) 
Human: API5 (8539, NM_006595) 
 
           |-50      |-40      |-30      |-20      |-10      |1        |11       |21       |31       |41       
Mouse     GACGCGCCAGTCTC  TGCGTAGA  CGTTGGGGGCGGTACCGTGCTGTACGCG G   TGACTGGT  GGCGGCCCGGCGGCGGCTCCCGGCGTACTAGTGCGGTCGAC
                          (BRE-)                                 (INR-)                                           
Human     GACGTGCGCGTCTT  TACGTAGA  CGTCGGGGGCGCAGCCGCGCTGTGCGCG G   TGACTGGC  GGCTGCACTGGCG  GCAGCTGGAGGTG  TAATAGTGCGGGTAG
                          (BRE-)                                 (INR-)                    (MTE+)                   
    551 Mouse: Ass1 (11898, BC002074) 
Human: ASS (445, BE298580) 
 
           |-50      |-40      |-30      |-20      |-10      |1        |11       |21       |31       |41       
Mouse     CCCCCCAGGCCCTGTGCT  TATAACC  CTGGATGCGCGCCTCTCTCAGCCCT G CTCCGCCGTCTGCCACTGC  CGCCTGGGCTCAC  TGGTAAGAAGTTCCGGG
                             (TATA+)                                                   (MTE-)                     
Human     GCCCCCGGGCCCTGTGCT  TATAACC  TGGGATGGGCACCCCTGCCAGTCCT   G CTCTGCC  GCCTGCCACCGCTGCC  CGAGCCCGGTAAG  GAGCCCTCGGCCC
                             (TATA+)                            (INR-)                       (MTE+)                 
    552 Mouse: Atp5c1 (11949, BC048777) 
Human: ATP5C1 (509, NM_001001973) 
 
           |-50      |-40      |-30      |-20      |-10      |1        |11       |21       |31       |41       
Mouse     AGACCGAGGACGCAGGCGGAGCGGGCCGGGGCGCCTGAGCTCCCGCGCAC G C  GCACTGAG  TCCCGTCTGCCCGACCTTCAAGGCTCCACCATGTTCTCGC
                                                                (INR-)                                          
Human     CTGAAGAAGAGAGCAAGGTGGGAGGGGCGCGCTGGGGAGCTTCG  GCGCAT G C  GCGCTGAGGCCTGCCTGACCGACCTTCAGCAGGGCTGTGGCTACCATG
                                                        (INR+)                                                  
    553 Mouse: Atrn (11990, NM_009730) 
Human: ATRN (8455, NM_012070) 
 
           |-50      |-40      |-30      |-20      |-10      |1        |11       |21       |31       |41       
Mouse     GATATGGACCCGCCGGGCCACCGTCGCCCGGCCCCGCCTAGCACGG  CCAG G CGA  AGGGGAGCCGGCGTGCGGGGTGTGTATGTGTTCGCTGGGCGCCGGC
                                                          (INR+)                                                
Human     CTTTGGACCGCACGAGCCACCGTCCGCACAGCCCCGCCCCGCACGG  CCAG G CGA  AGCGGAGCCG  GCCGTGCGGTGTG  TGTGTATGTGTTCGCGGGGCGCC
                                                          (INR+)                 (MTE-)                           
    554 Mouse: B2m (12010, X01838) 
Human: B2M (567, AV713115) 
 
           |-50      |-40      |-30      |-20      |-10      |1        |11       |21       |31       |41       
Mouse     TGGCTGTGAGTTCAGGAAC  TATATAA  GAGCGCGCGCCCTGGCTGGCTC  TC A TTTCA  GTGGCTGCT  ACTCGGCGCTTC  A  GTCGC  GGTCGCTTCAGTCGTCA
                              (TATA+)                         (INR+)                (MTE-)    (DPE+)                  
Human     GATTGGCTGG  GCACGCGT  TTAA  TATAAGT  GGAGGCGTCGCGCTGGCGG  GC A TTCCT  GAAGCTGACAGCATTC  GGGCCGAGATGTC  TCGCTCCGTGGCCTT
                      (BRE+)       (TATA+)                      (INR+)                       (MTE-)                   
    555 Mouse: Bcl2l1 (12048, U10100) 
Human: BCL2L1 (598, AA488236) 
 
           |-50      |-40      |-30      |-20      |-10      |1        |11       |21       |31       |41       
Mouse     AGCAGGTGTTTTGGACAATGGACTGGTTGAGCCCATCTCTATTATA  AAAA T GTC  TCAGAGCAACCG  GGAGCTGGTGGT  C  GACTT  TCTCTCCTACAAGCTT
                                                          (INR-)                   (MTE+)    (DPE-)                 
Human     AGCAGGTGTTTTGGACAATGGACTGGTTGAGCCCATCCCTATTATA  AAAA T GTC  TCAGAGCAACCG  GGAGCTGGTGGT  T  GACTT  TCTCTCCTACAAGCTT
                                                          (INR-)                   (MTE+)    (DPE-)                 
    556 Mouse: Bcl2l1 (12048, U10102) 
Human: BCL2L1 (598, BG419161) 
 
           |-50      |-40      |-30      |-20      |-10      |1        |11       |21       |31       |41       
Mouse     AATGGGGGTGACTTTTG  GAGAAAG  GCATTTCGGAGAAAAGGGGGTGGGTG G TTGTAGTAAAAAGAGGCGG  ATGAAACAATTCA  AAGCTGGCTGGTCCTTT
                            (TATA+)                                                    (MTE-)                     
Human     GCGGGGGGGACTGCCCAGGGAGTGACTTTCCGAGGAAGGCATTTCGGAGA A GACGGGGGTAGAAAAGGCT  GGTGGGAGATTCA  GAGTCCACTGGTGCTTT
                                                                                     (MTE-)                     
    557 Mouse: Bcl2l1 (12048, L35049) 
Human: BCL2L1 (598, AA488236) 
 
           |-50      |-40      |-30      |-20      |-10      |1        |11       |21       |31       |41       
Mouse     TGCCTACCA  GGTCGCAT  GATCCCTCCGGCCGGGGCTGGTTTTTTTTTTTT T TTTTTTTTTTTGCTGAGTTACCGG  CGACCC  AGCCACCACCTCCTCCCCG
                     (BRE+)                                                             (DPE-)                    
Human     AGCCTGCCG  GGTCGCAT  GATCCCTCCGGCCGGAGCTGGTTTTTTTG  CCAG C CAC  CGCGAGGCCGGCTGAGTTACCGG  CATCCC  CGCAGCCACCTCCTCTC
                     (BRE+)                                 (INR+)                          (DPE-)                  
    558 Mouse: Bcl2l1 (12048, AF133277) 
Human: BCL2L1 (598, AA488236) 
 
           |-50      |-40      |-30      |-20      |-10      |1        |11       |21       |31       |41       
Mouse     AGGGGGTTGGGCTCCCGGGTGGCTGGAGCCTGCGGAGCAGAGAGAGGCCG C CCTCGATCTGGTCGATGGAGGAACCA  GGTTGT  GAGGGGGCAGGTTCCTA
                                                                                        (DPE+)                  
Human     GAGGGGGCTGGGCTCCCGGGTGGCAGGAGGCCGCGGCTGCGGAGCGGCCG C CCTCGATCCGGGCGATGGAGGAGGAAGCAAGCGAGGGGGCTGGTTCCTG
                                                                                                              
    559 Mouse: Bcl2l1 (12048, AF088904) 
Human: BCL2L1 (598, AA488236) 
 
           |-50      |-40      |-30      |-20      |-10      |1        |11       |21       |31       |41       
Mouse     GGGGGAAATTACACTAAACCCATACCTCCGGGAGAGTTCTCCTGACTC  CC A GTAGG  AGGCGGAGAGCCAA  GGGGCGTGCTAGA  GCGAGGGGGTTGGGCTC
                                                            (INR+)                     (MTE-)                     
Human     AACCAACTAAAT  CCATACCA  GCCACCTCCGGGAGAGTACTCCTGGCTC  CC A GTAGG  AGGCGGAGAGCCAAGGGGCGTGCAAGAGAGAGGGGGCTGGGCTC
                        (BRE+)                                (INR+)                                              
    560 Mouse: Bcl2l1 (12048, NM_009743) 
Human: BCL2L1 (598, AA488236) 
 
           |-50      |-40      |-30      |-20      |-10      |1        |11       |21       |31       |41       
Mouse     CCCTTTCT  GGCGCGCA  CTCCTTTTGCGTCTCGGGCTCGCGCGCGCTGCCG C GGCACCGGAAGTGGCTGCGCTTGCA  AGTTCC  CCCGGTCTCTTCAGGGGA
                    (BRE-)                                                               (DPE+)                   
Human     TCACTCAA  GGCGCGCA  CTCCCTTTGCGTCTCGGGCTCGCGCGCGTTGCCG C GGCACCGGAAGTGACTGAGCTTGCA  AGTTCC  CCTGTCTCTTCAGGGGAA
                    (BRE-)                                                               (DPE+)                   
    561 Mouse: Bdnf (12064, AY057907) 
Human: BDNF (627, NM_170734) 
 
           |-50      |-40      |-30      |-20      |-10      |1        |11       |21       |31       |41       
Mouse     GGCCCCCTCCCCTCGCCCCCTCCCCGCTGCGCTTTTCTGGTATTCTTATT A AA  GCAGTAGC  CGGC  TGGTGCAGAAAAG  CAACAAGTTCCCCAGCGGTCTT
                                                                 (INR-)           (MTE-)                          
Human     CGTTGCCGCCCCCCACCCCCTCCCTGCTGCGCTTTTCTGGTATTATTATT   A AAGCGGT  AGTCTGC  CGGCGCTGATAAG  CAACAAGTTCCCCAGCGGTCTT
                                                              (INR-)              (MTE-)                          
    562 Mouse: Bdnf (12064, S71196) 
Human: BDNF (627, AY054393) 
 
           |-50      |-40      |-30      |-20      |-10      |1        |11       |21       |31       |41       
Mouse     CGGCTGGTGCAGAAAAGCAACAAGTTCCCCAGCGGTCTTCCCGCCCT  AGC T TGAC  AAGGCGAAGGGTTTCTTACCTGGCGACAGGGAAATCTCCTGAGCC
                                                           (INR-)                                               
Human     CTGCCGGCGCTGATAAGCAACAAGTTCCCCAGCGGTCTTCCCGCCCT  AGC C TGAC  AAGGCGAAGGTTTTCTTACCTGGCGACAGGGAAATCTCCCGAGCC
                                                           (INR-)                                               
    563 Mouse: Bdnf (12064, AY057914) 
Human: BDNF (627, BU506938) 
 
           |-50      |-40      |-30      |-20      |-10      |1        |11       |21       |31       |41       
Mouse     CGAAGCTCAACCGA  AGAGCTA  AATAATGTCTGACCCCAGTGCCTG  GCTCT G GC  TGAGCTCTGGGT  GCCCGTCGCTGCT  GCCGTGCCGGGGCGCACCCGCT
                         (TATA-)                           (INR-)                   (MTE-)                          
Human     CGAAGCTCAACCGA  AGAGCTA  AATAATGTCTGACCCGGGCGCAAGGCGC  A G CCTGGA  GCTCCGGGT  CCCCGACGCTGCC  GCCGCCGCGCCCGGGCGCACC
                         (TATA-)                               (INR-)                (MTE-)                         
    564 Mouse: Bcl2l11 (12125, AY369780) 
Human: BCL2L11 (10018, AY305716) 
 
           |-50      |-40      |-30      |-20      |-10      |1        |11       |21       |31       |41       
Mouse     CGGTGATTGGGCGCGGGGGCGGGGGCTCAACTACCGCAGAGTCTCAAGA  G C AGGCTG  CTTTCACTTCGCTCCC  CGCAGGCACTGGG  TCACCAGCTGGTTG
                                                             (INR+)                       (MTE-)                  
Human     AGTGATTGGGCGTA  GGAGCGGG  GCCGCCAGCCAGAGCTGGGCTGCAGGGC C GC  GCAGGTTT  CACTTCGCTCCGC  GCAGCCGCCTGGT  CTGCAGTTTGTTG
                          (BRE-)                                   (INR+)                    (MTE+)                 
    565 Mouse: Bmi1 (12151, NM_007552) 
Human: PCGF4 (648, BX491444) 
 
           |-50      |-40      |-30      |-20      |-10      |1        |11       |21       |31       |41       
Mouse     GCAGAGGTGGCC  GGATGCCA  AG  TGTAAGT  GTAAGTTGCTATGGAAACCCC A ACGGAGGCGAGTTCC  GAATCCGGAACA  A  GACCT  AGCCCGGGGCACTTCG
                        (BRE+)     (TATA+)                                           (MTE+)    (DPE+)                 
Human     GCAGCGGTGGCC  GGATGCCA  AG  TGTAAGT  GTAAGTTGCTATGGAAACCC  C G ACCGAG  GCGAGTTCC  GAATCCGGAGCG  A  GACGG  AGCCCCGGGCGCCGCC
                        (BRE+)     (TATA+)                       (INR-)                (MTE+)    (DPE+)                 
    566 Mouse: Bmp2 (12156, L25602) 
Human: BMP2 (650, AF040249) 
 
           |-50      |-40      |-30      |-20      |-10      |1        |11       |21       |31       |41       
Mouse     CCGACGACAGCAGCAG  CCTTGCCT  CAGCCTTCCCTTCCCGTCCCGGCCCC   G CACTCCT  CCCCCT  GCTCGAGGCTGTG  TGTCAGCACTTGGCTGGAGACTT
                            (BRE+)                              (INR+)             (MTE-)                           
Human     AGGAGGACGA  CAGCACCA  GCT  TCTCCTT  TCTCCCTTCCCTTCCCTGCCCC   G CACTCCT  CCCCCT  GCTCGCTGTTGTT  GTGTGTCAGCACTTGGCTGGGGA
                      (BRE+)      (TATA-)                         (INR+)             (MTE-)                           
    567 Mouse: Bmp7 (12162, BC010771) 
Human: BMP7 (655, AF210054) 
 
           |-50      |-40      |-30      |-20      |-10      |1        |11       |21       |31       |41       
Mouse     CTCGCTCTCTTGCTCGCTCTCTGGAGTTGCTGTGCTAGCCTTGCCGTGCG T CCTGGCGAGTGCGGGCCGAGGGGCCCCGGGCCAGAACTGAGTAAAGGAC
                                                                                                              
Human     CTCACTCGCCTTTTCGTTCGCCGGGGCTGCTTTCCAAGCCCTGCGGTGCG C CCGGGCGAGTGCGGGGCGAGGGGCCCGGGGCCAGCACCGAGCAGGGGGC
                                                                                                              
    568 Mouse: Bub1b (12236, AF107296) 
Human: BUB1B (701, AF310192) 
 
           |-50      |-40      |-30      |-20      |-10      |1        |11       |21       |31       |41       
Mouse     AAACTTGGCGGCCCGTGCGTGATGGGGGCGTGAGGAGGCTATTCTGAGAA G   GAATCGGG  TGCGTGGTTTTGTTTAGGAGCACGGGGTGACGATCTTCGCA
                                                               (INR-)                                           
Human     TTTGAAACTTGGCGGCTAGGGGTGTGGGCTTGAGGTGGCCGGTTTGTTAG   G GAGTCGT  GTACGTGCCTTGGTC  GCTTCTGTAGCTC  CGAGGGCAGGTTGC
                                                              (INR+)                      (MTE+)                  
    569 Mouse: Commd3 (12238, BC038077) 
Human: COMMD3 (23412, BI461776) 
 
           |-50      |-40      |-30      |-20      |-10      |1        |11       |21       |31       |41       
Mouse     CGCGCCCCGAGTCACGTGGTGTGCGTGTCGAAGGTCACGGCGCGCTC  ACA A TGGA  GCTCTCGGAGTCTGTGCAAAGAGG  CATCCA  GACGCTGGCGGATCC
                                                           (INR-)                           (DPE-)                
Human     CGCGGCGCGTGTCACGTGGTGTGCGTGTCGAAGGTCACGGCGCGCTC  ACA A TGGA  GCTCTCGGAGTCTGTG  CAGAAAGGCTTCC  AGATGCTGGCGGATCC
                                                           (INR-)                       (MTE-)                    
    570 Mouse: Serping1 (12258, NM_009776) 
Human: SERPING1 (710, NM_000062) 
 
           |-50      |-40      |-30      |-20      |-10      |1        |11       |21       |31       |41       
Mouse     GACCCCTGAGCTCCTA  GGCTCCAA  GGCTGGCTCTGAGGCTAACTGGCTTC G TAGGACGCAGCTG  ACATCGCTGCCC  A  GATGG  CCTCCAGGCTGACCCCAC
                            (BRE-)                                               (MTE+)    (DPE+)                   
Human     GGGCCCCTG  GGCTCCCA  GGGTGGGAGCTGGCTCCGAGGCTGGCTGGCTCC   G CAGGTCC  GCTGACGTCGCCGCCC  AGATGG  CCTCCAGGCTGACCCTGCTG
                     (BRE-)                                     (INR+)                   (DPE+)                     
    571 Mouse: Serping1 (12258, AF052039) 
Human: SERPING1 (710, NM_000062) 
 
           |-50      |-40      |-30      |-20      |-10      |1        |11       |21       |31       |41       
Mouse     GCTGAGAGTGCCCCCCCCCCACACACTGATTCTCTGCGACTGTCTGC  TCA G TCTG  TCCTGAAGCTGCCTAGTGACCAA  GAACTT  GGACCAGGTTTGGTAA
                                                           (INR+)                          (DPE-)                 
Human     CTGCCCCCCCCGCACCCCACCCTCCCTGACCCTGGGGGACTCTCTAC  TCA G TCTG  CACTGGAGCTGCCTGGTG  ACCAGAAGTTTGG  AGTAGGTTTGGTGC
                                                           (INR+)                         (MTE-)                  
    572 Mouse: Cacnb4 (12298, AK038633) 
Human: CACNB4 (785, AW134825) 
 
           |-50      |-40      |-30      |-20      |-10      |1        |11       |21       |31       |41       
Mouse     GCCGCTTTCCTAGCTCTGTCACCAGTCTGTGCTTGAGGAAGGCTTTTGCT T TTTATGCTCTGTATTGG  AGAGGCAGATAAA  ATGCCAGGTCTGCATGTCT
                                                                                   (MTE-)                       
Human     GCCGCTTTCCTAGCTCAGTCACCAGTCTGTGGTTGAGGAAGGGTTTTGCT T TTTGTGCTCTGTATTGG  GAAGGCAGATAAA  ATGCCAGGTCTGGACTGCC
                                                                                   (MTE-)                       
    573 Mouse: Capn3 (12335, AB117943) 
Human: CAPN3 (825, AB117940) 
 
           |-50      |-40      |-30      |-20      |-10      |1        |11       |21       |31       |41       
Mouse     CTGAGAGTGCTGCTGTGTTACAGATCCCAGTGTTTCAGCGAGGTG  GAAGT G TG  GTACCAGTAAAGACC  ACTGTGGGAACGT  CCACTGGCTGGATGGCTGA
                                                         (INR-)                      (MTE-)                       
Human     CTGAGCGTGCTGCTCTGTTACAGATTCCAGTGTTTCAGCGAGGTG  GAAGT G TG  ATACCAATAAAGACAACT  GTAGGAAAATCCA  CAGGCTGGATGACTGA
                                                         (INR-)                         (MTE-)                    
    574 Mouse: Cat (12359, AK075853) 
Human: CAT (847, L13609) 
 
           |-50      |-40      |-30      |-20      |-10      |1        |11       |21       |31       |41       
Mouse     GGCGGTGCTGATTGG  TGGAGCCT  GAAGTCACCACTCCAGCGGGCCTGGCC A A  CAAGATTG  CCTTCTCCGGGTGG  AGACCG  CTGCGTCCGTCCCTGCTGTC
                           (BRE+)                                 (INR+)                 (DPE+)                     
Human     GGGTGGTGCTGATTGG  CTGAGCCT  GAAGTCGCCACGGACTCGGGGCAACA G   GCAGATTT  GCCTGCTGAGGGTGG  AGACCC  ACGAGCCGAGGCCTCCTGCA
                            (BRE+)                               (INR+)                  (DPE+)                     
    575 Mouse: Entpd2 (12496, AK002553) 
Human: ENTPD2 (954, BC011003) 
 
           |-50      |-40      |-30      |-20      |-10      |1        |11       |21       |31       |41       
Mouse     GCCCCGCCC  CGGAGCCT  AACTCTGGGGCCCCGGCCACCGGGCCTCCCGCT G CCTGCTGTCCGGGGTCCCTGCTGTGTTCTCCCGCGTGCTCCCATGGCTG
                     (BRE+)                                                                                     
Human     GGGGCCCCTACCCCGCGGTCCCGCGGCCCCGCCCGCCTCCGCCTCCGGCT C CCCGCACTCTCCGGGTCCAC  GCATCGTCCTCCC  GCGCGCCCGCCCGCCC
                                                                                      (MTE+)                    
    576 Mouse: Entpd6 (12497, AK030789) 
Human: ENTPD6 (955, AF039916) 
 
           |-50      |-40      |-30      |-20      |-10      |1        |11       |21       |31       |41       
Mouse     CGCATCAACGCCGTCCTGCCCACCCGGTGACGCAAAGTGCACGTAGGCGG A G  ACAGCTTC  TCGGCGTCTGCTGGGGTTTGCTTCCACGCGTTAAGGCTCT
                                                                (INR+)                                          
Human     GGCTGGGTGCGCCCCCCCGCCCAGCCTGTGACGCCACGTGCATGGGGCGG A GC  CCAGGCCC  TAGGGAATCGTGG  GGTCGT  ATCCCGCGGGTGGAGGCCGG
                                                                 (INR+)                (DPE+)                     
    577 Mouse: Cd44 (12505, U96138) 
Human: CD44 (960, CD105061) 
 
           |-50      |-40      |-30      |-20      |-10      |1        |11       |21       |31       |41       
Mouse     CACTCTTT  CAAAGCCT  GG  AATAAAA  ACCACAGCCAACTTCCGAAGC  GGTC T CAT  TGCCCAGCAGCCC  CCAGCCAGTGACA    GGTTCC  ATTCACCCTCGTTG
                    (BRE+)     (TATA+)                        (INR-)                    (MTE+)     (DPE+)               
Human     TCACTGTTTTCAACCTCG  AATAAAA  ACTGCAGCCAACTTCCGAGGCAGCC   T CATTGCC  CAGCGGACC  CCAGCCTCTGCCA    GGTTCG  GTCCGCCATCCTCG
                             (TATA+)                            (INR-)                (MTE+)     (DPE+)               
    578 Mouse: Cdc25b (12531, AK043730) 
Human: CDC25B (994, AY494082) 
 
           |-50      |-40      |-30      |-20      |-10      |1        |11       |21       |31       |41       
Mouse     GGCCCATTGCTCTTCCTCCCTCCCTTACCTCCCTCCTTCCCCTCACCCCA G GC  TCACTCTC  GGAGCTGAGCCAGCTG  GGTCGG  CGTCTGCTGGCCGCTGT
                                                                 (INR+)                   (DPE+)                  
Human     CCTGTGGCTCTTCCTCCCTCCCTCCTTCCCCCCCCCCCCACCCCTCGCCC G C  TGCCTCCC  TCGGC  CCAGCCAGCTGTG  CCGGCGTTTGTTGGCTGCCCTG
                                                                (INR-)            (MTE-)                          
    579 Mouse: Cebpb (12608, X62600) 
Human: CEBPB (1051, AF350408) 
 
           |-50      |-40      |-30      |-20      |-10      |1        |11       |21       |31       |41       
Mouse     CAGCCCGTTGCCA  GGCGCCGC  CT  TATAAAC  CTCCCGCTCGGCCGCCGCCG C GC  CGAGTCCG  AGCCGCGCACG  GGACCG  GGACGCAGCGGAGCCCGCGGGC
                         (BRE-)     (TATA+)                          (INR+)              (DPE-)                       
Human     AGCGGTTGCTACGGGCCGCCCT  TATAAAT  AACCGGGCTCAGGAGAAACTT T AG  CGAGTCAG  AGCCGCGCACG  GGACTG  GGAAGGGGACCCACCCGAGGGT
                                 (TATA+)                           (INR+)              (DPE-)                       
    580 Mouse: Cel (12613, BC006872) 
Human: CEL (1056, CN265348) 
 
           |-50      |-40      |-30      |-20      |-10      |1        |11       |21       |31       |41       
Mouse     ACCTTGGCCCCAGCTC  CATAAAT  ACTGGAGAAGAGGAGAGAAGGCCGC  CT A GAGGC  AGACACTCACCATGGGGCGCCTGGA  GGTTCT  ATTTCTTGGCCTC
                           (TATA+)                            (INR+)                            (DPE+)              
Human     ACCTTGGATCCAGCTC  CATAAAT  ACCCGAGGCCCAGGGGGAGGGCCAC  CC A GAGGC  TGATGCTCACCATGGGGCGCCTG  CAACTG  GTTGTGTTGGGCCTC
                           (TATA+)                            (INR+)                          (DPE-)                
    581 Mouse: Chgb (12653, AK019893) 
Human: CHGB (1114, NM_001819) 
 
           |-50      |-40      |-30      |-20      |-10      |1        |11       |21       |31       |41       
Mouse     CTGCACCCCGCCTGCCCTT  CATAAGA  GCCAGGCCTGCGCCCACCGCG  CCA C AGCT  GCTCTGCGGAGCCCGCAC  CCGCCGAGCTCCT  CCTACACTCAGCTG
                              (TATA+)                        (INR+)                         (MTE-)                  
Human     GCGGCCCCCGCCTGCCC  TTTAAAA  GAGCGGGGCCTGCGCCGGCCGCG  CCA C ACCG  CGGGGACCAG  GAGGCACGCTGGT  TTTCCGGGGCCGCTCCATCGCG
                            (TATA+)                          (INR+)                 (MTE-)                          
    582 Mouse: Chrm4 (12672, NM_007699) 
Human: CHRM4 (1132, NM_000741) 
 
           |-50      |-40      |-30      |-20      |-10      |1        |11       |21       |31       |41       
Mouse     GAGAACCCGCGGAAGCACATTGCCCACGCCCAGAGACTACAGGAACTGCG   T GCCTGGG  GGTTGATGGGAGGGCGGGGCATCGGAGAACCTTGGCCCTTTC
                                                              (INR-)                                            
Human     CGAGGACTCGGGAAACACAGTGCCCACGCCCAGAGGCTGCAGGAAC  TGCG T GCT  AGGGGCTTGGAGGGAGGGCGGGGCACCCGAGAGCCAGGGCTCCTTC
                                                          (INR-)                                                
    583 Mouse: Ckmt1 (12716, NM_009897) 
Human: CKMT1 (1159, AK094322) 
 
           |-50      |-40      |-30      |-20      |-10      |1        |11       |21       |31       |41       
Mouse     ACCACACCCTCCTC  CTCTCTT  TATATTCCCCCTCCAGTCCAGGAACTG  CC A GCTGC  AGAGAAGA  AGGAAGAGAAGGA  AGAAGAAACTCACCATTCCCTGC
                         (TATA-)                              (INR+)               (MTE-)                           
Human     CACCACACCCTTCAC  CTCACTT  TACCTTCTCCTCCAGCACAGGAACTAG  G A ACTACG  GAGAGAGAAGCCAAG  GGAGAGGAGGAGG  AGGAAACTAACGATT
                          (TATA-)                              (INR-)                      (MTE+)                   
    584 Mouse: Col5a1 (12831, NM_015734) 
Human: COL5A1 (1289, AK057231) 
 
           |-50      |-40      |-30      |-20      |-10      |1        |11       |21       |31       |41       
Mouse     CCACAGCCTCCGCGTTGTCTGCCAGCAGCGAGGGAGGGAGGGAAAGGGGG G A  AAAGTGCT  CAGCGCCGAAGGCGA  GGTCCG  CACTCCTGGTCCCCGCGGC
                                                                (INR-)                  (DPE+)                    
Human     GACAGCCT  CCGCGGCT  GCCTTCCAGGAGAGAGGGAGGGAGGAAAAGGGGG A AA  AAAGTGCT  CCGCGCCGAAGGCGA  GGTCCG  CACTCTCCGTCCCCGCGG
                    (BRE+)                                         (INR-)                  (DPE+)                   
    585 Mouse: Crat (12908, AK041431) 
Human: CRAT (1384, AL521455) 
 
           |-50      |-40      |-30      |-20      |-10      |1        |11       |21       |31       |41       
Mouse     CGGCCGGGGACT  GCGAGCCA  CCAAAGCTCCGCCCCGTGAGTCCTGCCCCA C CG  GCAGCCCG  CCGGGCCCGGTGGCCGCGGGGCCGCCTCTACTGGCTAGC
                        (BRE+)                                     (INR+)                                         
Human     CGCCCGGGGCCCGCTACCGGCCCAGGCCCCGCCTCGCGAGTCCTCCTCCC   C GGGTGCC  TTCCCGCAGCCCGCTCGGCCCAGAGGGTGGGCGCGGGGCTGC
                                                              (INR-)                                            
    586 Mouse: Cry2 (12953, NM_009963) 
Human: CRY2 (1408, BC041814) 
 
           |-50      |-40      |-30      |-20      |-10      |1        |11       |21       |31       |41       
Mouse     GCCAGGGAGAGGCTGCATCATAGGTCGCTGGGCGGGCGCTGGGCGGGACC A ACGGGGCGGTCGGGCGGAGCGGC  AGACCG  AGACCCAGTCCAGGCGGCGA
                                                                                     (DPE+)                     
Human     AGGGTGGAGTTGC  GGCGTCAT  AGGTCACTGGGCGGGCTATGGGCGGGGT  C C ACGTCG  CCTACCGGGGC  GGAGCGGGGGTGG  CTGGAGCAGTCTGGACAGT
                         (BRE-)                                (INR+)                  (MTE+)                       
    587 Mouse: Csnk2a1 (12995, AK031617) 
Human: CSNK2A1 (1457, AF011920) 
 
           |-50      |-40      |-30      |-20      |-10      |1        |11       |21       |31       |41       
Mouse     TCCGCTTCCGGCAGCGGAGGCTGCAGCCTCGCTCTGGTCCCTG  CGGCTGG C   GGCCGAGCCGTGTGTCTCCTCCTCCGCCGCCGCCATATTGTCTGTGTGA
                                                       (INR-)                                                   
Human     TCCGCTTCCGGCAGCAGCGGCTGCAGCCTCGCTCTGGTCCCTG  CGGCTGG C   GGCCGAGCCGTGTGTCTCCTCCT  CCATCGCCGCCAT  ATTGTCTGTGTGA
                                                       (INR-)                              (MTE+)                 
    588 Mouse: Cst3 (13010, AK014368) 
Human: CST3 (1471, AA738018) 
 
           |-50      |-40      |-30      |-20      |-10      |1        |11       |21       |31       |41       
Mouse     GGGCGGGTCCCTAACGCTCGCATTTGGGTAAAAGTCGCACGGAGTAGCAG   C GTCTGTT  CTGCACCAACTC  AGAGTCTTGTTGG  AGCTTTATCCCTTTGTC
                                                              (INR-)                   (MTE+)                     
Human     ACCGCAGTCGCCGGCCTCGCGGGGCTCACGGCCTCGCCTCGGTATCGCAG C GGGTCCTCTCTAT  CTAGCTCCAGCCT  CTCGCCTGCGCCCCACTCCCCGC
                                                                               (MTE+)                           
    589 Mouse: Cst7 (13011, AK004420) 
Human: CST7 (8530, AJ510167) 
 
           |-50      |-40      |-30      |-20      |-10      |1        |11       |21       |31       |41       
Mouse     CTTGTGGCCCCAAGTCCTGAA  GATGAAG  CGCTTCTGGTTAAAA  TGATTCA G   CAAGAAACCACGCCCCACT  GAAGCTACCCCAC  CATGCCCTGGTCCTGGA
                                (TATA+)                  (INR+)                          (MTE+)                     
Human     TTTGTGGCTGATAGATCTGAAGGTGAAGAGCTTCTGGCCAAAA  TGATTCA G   CAGCAAAACACTCCCCTCC  CCAGCACCACGCA  GGCAGCCCCCTAGCCCC
                                                       (INR-)                          (MTE+)                     
    590 Mouse: Cyp24a1 (13081, D49438) 
Human: CYP24A1 (1591, NM_000782) 
 
           |-50      |-40      |-30      |-20      |-10      |1        |11       |21       |31       |41       
Mouse     ATCAGACCTGGC  GGCATCCC  TTCGACCCTCCTTGACCCCTCGTGGCT  TTA G ACCT  TAGAAGTCAAATTTGC  AAAGCCACAGAGT  ACTCGGGCCAAACCCC
                        (BRE-)                               (INR+)                       (MTE+)                    
Human     AGCGCCAGC  AGCATCTC  ATCTACCCTCCTTGACACCTCCCCGTGGCTCCA G   CCAGACCC  TAGAGGTCAGCCTTGC  GGACCA  ACAGGAGGACTCCCAGCTT
                     (BRE-)                                      (INR+)                   (DPE-)                    
    591 Mouse: Cyp24a1 (13081, D89669) 
Human: CYP24A1 (1591, NM_000782) 
 
           |-50      |-40      |-30      |-20      |-10      |1        |11       |21       |31       |41       
Mouse     TCCAGCATGCCCTGTTCC  CATAAAT  ACGAGGTCCCTAAGCCCGGGAGCTC G GGGAGGCACAAGGAGGTACATC  AGACCT  GGCGGCATCCCTTCGACCCTC
                             (TATA+)                                                  (DPE+)                      
Human     CAGGGCATGCTCTGTCTC  CATAAAT  GCATGGTCCCTGGGCATAGG  AACAT G GA  GAGGGACAGGAGGAAAC  GCAGCGCCAGCAG  CATCTCATCTACCCTCC
                             (TATA+)                       (INR-)                        (MTE+)                     
    592 Mouse: Dbh (13166, NM_138942) 
Human: DBH (1621, X13257) 
 
           |-50      |-40      |-30      |-20      |-10      |1        |11       |21       |31       |41       
Mouse     AACCCCACTGGACA  GGCATAAA  TGGCAGAGTGGGGTTGGGGTGCTCATCC C TG  CCATGCAA  GCTCATC  TCAGCCACCAGCC  TTGTTGGAGCAGCCTCCCA
                          (BRE-)                                   (INR+)              (MTE+)                       
Human     CCATTCAGGACCAG  GGCATAAA  TGGCCAGGTGGGACCAGAGAGCTCACCC C AG  CCATGCCC  GCC  CTCAGTCGCTGGG  CCAGCCTGCCCGGCCCCAGCATG
                          (BRE-)                                   (INR+)          (MTE-)                           
    593 Mouse: Dlx1 (13390, NM_010053) 
Human: DLX1 (1745, AY257976) 
 
           |-50      |-40      |-30      |-20      |-10      |1        |11       |21       |31       |41       
Mouse     TTTCCTGGGTGGAGA  AGCGTGGG  TTCCTGGCTCTCCACGCGCACTGCTTT G A  TCAGACCC  GTGCA  GCCTCGAGCTGGA  GTGGCCAGCTGGGCCTGGAGAG
                           (BRE-)                                 (INR+)            (MTE-)                          
Human     TTTCCTGGGAGGAGA  AGCGCGGG  TTCCTGGCTCTCCACGCGCACTGCTTT A A  TCAGACCC  GTGCA  GCCTCGAGCTGGA  GTGGCCAGCTGGGCCTGGAGCA
                           (BRE-)                                 (INR+)            (MTE-)                          
    594 Mouse: Dlx1 (13390, U51000) 
Human: DLX1 (1745, AY257976) 
 
           |-50      |-40      |-30      |-20      |-10      |1        |11       |21       |31       |41       
Mouse     GCGCTCCGCACCGGACTAGGTGACCTCAAGAAGAAGTGGTCCGGGGTC  CC A GGCGT  TGGGGGCGGGCGAAGGGTGGGGACAGCAAGGGGGAGGGCGACGG
                                                            (INR+)                                              
Human     TAGCTCTGCGCCGAACTGAGTGGCAGCGACGAGAAGCGGTCCCG  GGTGTC C G  GTGTTAGGGACCGCTGAAGGGTGGGGACAGTGGTAGGGGGGCGGCGGC
                                                        (INR-)                                                  
    595 Mouse: Dlx2 (13392, M80540) 
Human: DLX2 (1746, BC032558) 
 
           |-50      |-40      |-30      |-20      |-10      |1        |11       |21       |31       |41       
Mouse     AAAGCTGCTCGGGGGTCGCTCACCTGCTCCCGCCTCCTGGAGCCCCTACC G   GGTCTGAC  CGCTCTTGCCCCCT  CCATTGCCCGCCC  CCCGCCCGAGCCGG
                                                               (INR-)                     (MTE+)                  
Human     TAAAGCCGCTCGGGGCCGCTCACCTGCTCCCGCCTCCCAGAGC  CCCGACC C   GGCCCAGCCACCCCTGC  CCCCTCCACTGCC  CGCCCCCCGCCAGAGCCCG
                                                       (INR+)                        (MTE-)                       
    596 Mouse: Dlx2 (13392, NM_010054) 
Human: DLX2 (1746, BC032558) 
 
           |-50      |-40      |-30      |-20      |-10      |1        |11       |21       |31       |41       
Mouse     CGCGGTCACGTGATAAGT  GTTAAAT  GCCCACCCTGCAGGAGCCTGCCCG  G C AGACAA  TCGTAAAGCTGCTCGGG  GGTCGC  TCACCTGCTCCCGCCTCCTG
                             (TATA+)                           (INR+)                    (DPE+)                     
Human     AGCGCTCACGTGACGAGT  GTTAAAT  GCCCACACTTCAGGAGCCCGCCTG  G C AGACAA  TCGTAAAGCCGCT  CGGGGCCGCTCAC  CTGCTCCCGCCTCCCAG
                             (TATA+)                           (INR+)                    (MTE-)                     
    597 Mouse: Dnajc1 (13418, AK012505) 
Human: DNAJC1 (64215, NM_022365) 
 
           |-50      |-40      |-30      |-20      |-10      |1        |11       |21       |31       |41       
Mouse     CACCGGAAG  CGAGGCCT  GGGATAGGCTGAGGCCTGGGGAAACCTCTC  CCA C ACGG  CCGCTTGGG  TGCCAGCGCTGGC  TCTCCAGCTGTTCCTAGGCCGGG
                     (BRE+)                                  (INR+)                (MTE-)                           
Human     CACCGGAAG  CGAGGCCT  GGGATAGGCTGAGGCGAAGGGAAGCCTCAC  CCA C TGTG  CAGCCCAGGC  GCCGGGCGCTGCC  TCTACAGCTGTGTGTAGGCCTG
                     (BRE+)                                  (INR-)                 (MTE-)                          
    598 Mouse: Dncic2 (13427, AK088015) 
Human: DNCI2 (1781, AK096579) 
 
           |-50      |-40      |-30      |-20      |-10      |1        |11       |21       |31       |41       
Mouse     GCCATTTGTCCCTCGGCTCTCGCCGTAGCGGCCGCCGCCCATCCCTCTTT G TG  TGCTTGGG  ACGCCGC  GGATCTGCGGCGG  CGACAGTTGGAGAGGGACG
                                                                 (INR-)              (MTE+)                       
Human     GCTATTTGTCCCTAAGCTCTCGCCGTAGCAGCCGCCGCCCATCCCTCTT  T G TGTGCT  TTGGAAAG  CCGCGGAGCTGGT  GGTGGCTACAGTTGGTGTTGGG
                                                             (INR-)               (MTE-)                          
    599 Mouse: Dnm1 (13429, AK011651) 
Human: DNM1 (1759, NM_004408) 
 
           |-50      |-40      |-30      |-20      |-10      |1        |11       |21       |31       |41       
Mouse     CGGCGGCCTGGAGA  AGCGCTCC  GCTCGGATCCCCGAGGGGCGGGGGACCC G CGGCGCACGCAGTCGGG  ATATTCGGCTGCA  GCCGCGAAGCCGGGGGCCA
                          (BRE-)                                                     (MTE-)                       
Human     GCGGCGTCCG  GGAGCGGT  GCTCGCTCCGATCCCCGAGGGGCGGGGGCCCC G CGGCGCAGGCAGTCTGG  GCGCGCGGCTGCA  GCGGCGGAGCCGGAGTCGG
                      (BRE-)                                                         (MTE-)                       
    600 Mouse: Dnmt2 (13434, AK029136) 
Human: DNMT2 (1787, NM_004412) 
 
           |-50      |-40      |-30      |-20      |-10      |1        |11       |21       |31       |41       
Mouse     GGAACGGAGCGGACATGGGGGAGTAAGAGACGCAGAGAACCGGTAGGC  CT A GTTTC  CAGGCCGC  GGTCGCGGTTGCG  AGA  GGATGG  AACCTCTGCGTGTC
                                                            (INR+)               (MTE-)        (DPE+)               
Human     CTGAGGGAGC  GGCGCGAT  GGAGGGAGGAGGAGCGACGGACCGGCA  GGCCT A GC  TCCGGGGCTGCGGCGGCTGAG  GCGCGGGGATGGA  GCCCCTGCGGGTG
                      (BRE-)                               (INR-)                            (MTE-)                 
    601 Mouse: Dpm1 (13480, AK004834) 
Human: DPM1 (8813, BC007073) 
 
           |-50      |-40      |-30      |-20      |-10      |1        |11       |21       |31       |41       
Mouse     TCCGAGATTTGGTACGGCCCGTGTCGGATGACGTAAGTCCGCTTCCGGCG C CTCTCGCAGAGCCGTCATGGCTTCCACGGGGGCGAGTCGCAGTCTTGCT
                                                                                                              
Human     GGTCCGACCCGTCCTTTT  CCGCGCCA  CATTACGTAATTCCGCTTCCGG  CA T CTGGC  TCAGTTCCGCCATGGCCTCCTTGGA  AGTCAG  TCGTAGTCCTCGC
                              (BRE+)                          (INR-)                            (DPE+)              
    602 Mouse: Dpm2 (13481, BC008256) 
Human: DPM2 (8818, BG420520) 
 
           |-50      |-40      |-30      |-20      |-10      |1        |11       |21       |31       |41       
Mouse     CACGTGATTT  ACAAGCCG  GAAAGG  CCTAAAC  CAGGAACCGGAT  GTATTTT G   TTGCTCCGGCGGAACG  CTGGAGAGATGGT  GAGCCTGGGGTGGAGTACAT
                      (BRE+)         (TATA+)               (INR+)                       (MTE-)                        
Human     CTCCGCAGTC  CAAAGCCG  GAAGAGCGTGGACCCGGAACCGGATGT  GGCTT G CG  GCTCGGGTGGC  TGAGCGCGCGGGG  AAATGGTGAGATTGGCACCGTGT
                      (BRE+)                               (INR-)                  (MTE+)                           
    603 Mouse: Dpp4 (13482, U12599) 
Human: DPP4 (1803, BC065265) 
 
           |-50      |-40      |-30      |-20      |-10      |1        |11       |21       |31       |41       
Mouse     CACCGAAGCCCGCCTTGCGCTAAC  TAATGTT  TAACTCAGGCCG  AAACTTG G   CGGCGAGCTCAGGGTGACTGCGTGCAGAGCAGCCGCGCAGGACGTCCGT
                                   (TATA-)               (INR-)                                                   
Human     TACCCAGCGGGCTC  GGCGCTCA  C  TAATGTT  TAACTCGGGGCCG  AAACTTG C   CAGCGGCGAGTGACT  CCACCGCCCGGAG  CAGCGGTGCAGGACGCGCGTC
                          (BRE-)    (TATA-)                (INR-)                      (MTE+)                         
    604 Mouse: Dusp2 (13537, AK088059) 
Human: DUSP2 (1844, BQ271986) 
 
           |-50      |-40      |-30      |-20      |-10      |1        |11       |21       |31       |41       
Mouse     CGAGGGGCCAGCCCTCGAGTA  TTTAAGC  CCGGGCTCGACGAAGAGCCC  CC A GAACC  GTCAGCTCCCGCCACAC  AGACAC  CGCGCCGCCGGAGCCGAGGGA
                                (TATA+)                       (INR+)                    (DPE+)                      
Human     CCGGAGGCCGGCCCAC  GGGTACT  TAACCCGGGCCGCCGCGGAGGGCGCCC   G GAGTCGA  CCGCTCGG  GCAGCGCCACCGC  CACGAGAGCCCGGGACGCGGG
[truncated: 4,901,410 more chars]
